# Rapid (≤25 °C) cycloisomerization of anhydride-tethered triynes to benzynes – origin of a remarkable anhydride linker-induced rate enhancement†

**DOI:** 10.1039/d4sc07232d

**Published:** 2025-01-07

**Authors:** Dorian S. Sneddon, Paul V. Kevorkian, Thomas R. Hoye

**Affiliations:** a Department of Chemistry, University of Minnesota 207 Pleasant St. SE Minneapolis MN 55455 USA hoye@umn.edu

## Abstract

The hexadehydro-Diels–Alder (HDDA) reaction is a cycloisomerization between a conjugated diyne and a tethered diynophile that generates *ortho*-benzyne derivatives. Considerable fundamental understanding of aryne reactivity has resulted from this body of research. The multi-yne cycloisomerization substrate is typically pre-formed and the (rate-limiting) closure of this diyne/diynophile pair to produce the isomeric benzyne generally requires thermal input, often requiring reaction temperatures of >100 °C and times of 16–48 h to achieve near-full conversion. We report here that diynoic acids can be dimerized and that the resulting substrate, having a 3-atom anhydride linker (*i.e.*, O=COC=O), then undergoes HDDA cyclization within minutes at or below room temperature. This allows for the novel *in situ* assembly and cyclization of HDDA benzyne precursors in an operationally simple protocol. Experimental kinetic data along with DFT computations are used to identify the source of this surprisingly huge rate acceleration afforded by the anhydride linker: >10^7^ faster than the analogous multi-yne having, instead, a CH_2_OCH_2_ ether linker.

## Abstract 2

An anhydride linker between a 1,3-diyne and tethered diynophile greatly accelerates the cycloisomerization that produces a HDDA benzyne: (*t*_1/2_ = 9 min for a triyne and even shorter for a tetrayne).

## Graphical abstract

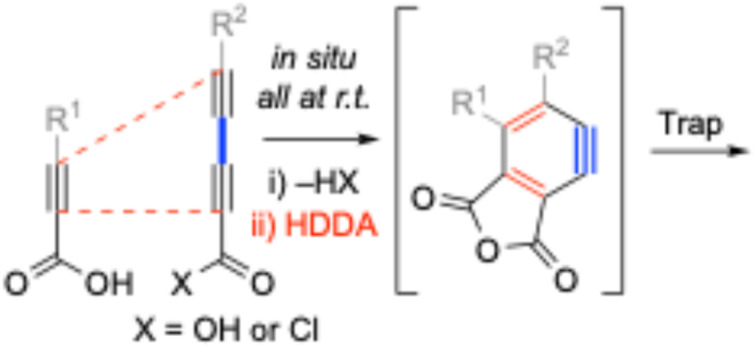

## Introduction

**Fig. 1:**
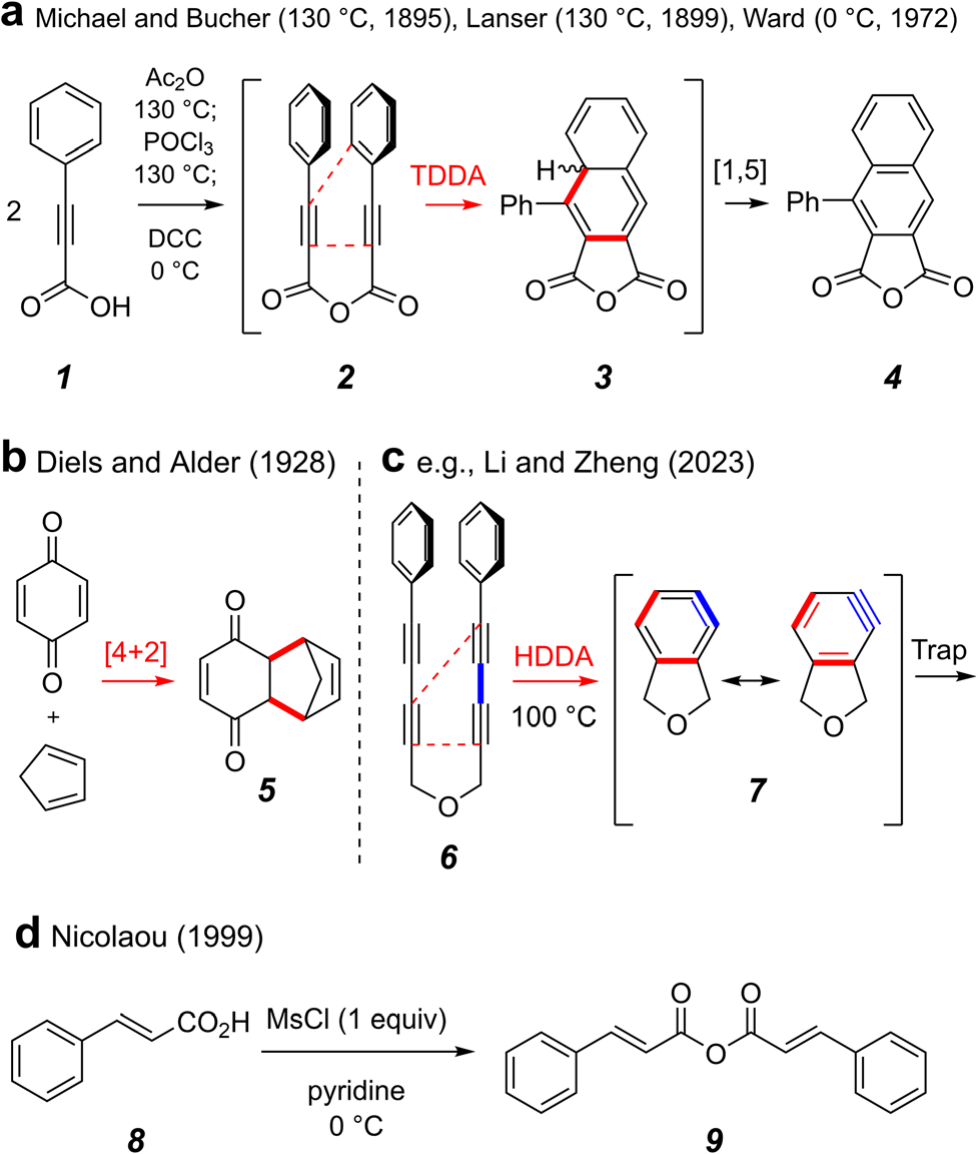
(a) Reports of the tetradehydro-Diels–Alder (TDDA) reaction of phenylpropiolic acid. (b) A classical Diels–Alder reaction (c). A hexadehydro-Diels–Alder (HDDA) reaction to form an *ortho*-benzyne (and its subsequent trapping). (d) Facile formation of a carboxylic acid anhydride mediated by methanesulfonyl chloride.

The Diels–Alder [4 + 2] cycloaddition is one of the most, arguably the most, venerated reaction in the field of organic chemistry. Its ability to merge two reactive substrates, a diene and a dienophile, to simultaneously introduce up to four contiguous stereocenters affords it considerable utility and versatility. In light of more recent understanding in the realm of cycloaddition chemistry, we can classify other processes as dehydro variants of the classical Diels–Alder reaction, wherein the alkenes that comprise either the diene or the dienophile are instead alkynes.^1^ By this definition, the first disclosure of any Diels–Alder-like process is the 1895 report by Michael and Bucher of the condensation and subsequent tetradehydro-Diels–Alder (TDDA) reaction of two equivalents of phenylpropiolic acid (1) in refluxing acetic anhydride to efficiently produce the naphthalene–anhydride derivative 4 (Fig. 1a).^2,3^ In 1899, Lanser reported a similar transformation using phosphoryl chloride as the dehydrating agent.^4^ Baddar and coworkers later carried out a series of experiments in which they established the intermediacy of phenylpropiolic acid anhydride (2) in this transformation.^5^ It is now recognized that TDDA reactions of substrates containing tethered conjugated enyne to alkyne subunits like that present in 2 proceed *via* 1,2,4-cyclohexatrienes 3,^6^ which then undergo net 1,5 H-atom migration to afford 4.

It was nearly 30 years later that Diels and Alder published their seminal work producing adducts such as 5, in what we regard today as the classical Diels–Alder reaction (Fig. 1b).^7^ A more recent variant of a net cycloaddition reaction to produce six-membered carbocycles is the hexadehydro-Diels–Alder (HDDA) reaction (Fig. 1c).^8^ For example, a poly-yne substrate joined by a three-atom tether such as the ether linker in 6 undergoes net intramolecular [4 + 2] cycloaddition to form highly reactive, yet selective, *ortho*-benzyne derivatives such as 7 under purely thermal conditions.^9^ Subsequent experiments done by the Ward group^10^ in which phenylpropiolic acid (1) was converted to the anhydride 2 using DCC were shown to produce the known TDDA adduct 4 even at 0 °C.^11^ Considering that most TDDA and HDDA cyclizations proceed by stepwise mechanisms,^12^ we reasoned that an anhydride linked multi-yne substrate could potentially produce an HDDA benzyne also at ambient temperature. To date, there are few examples of HDDA substrates that cyclize at or below room temperature.^8,13^ One final reaction that influenced our thinking and design of the project we describe here is shown in Fig. 1d. Nicolaou and coworkers described the formation of symmetrical anhydrides under very mild conditions *via* transient, mixed carboxylic–sulfonic anhydrides.^14^

## Results and discussion

**Fig. 2:**
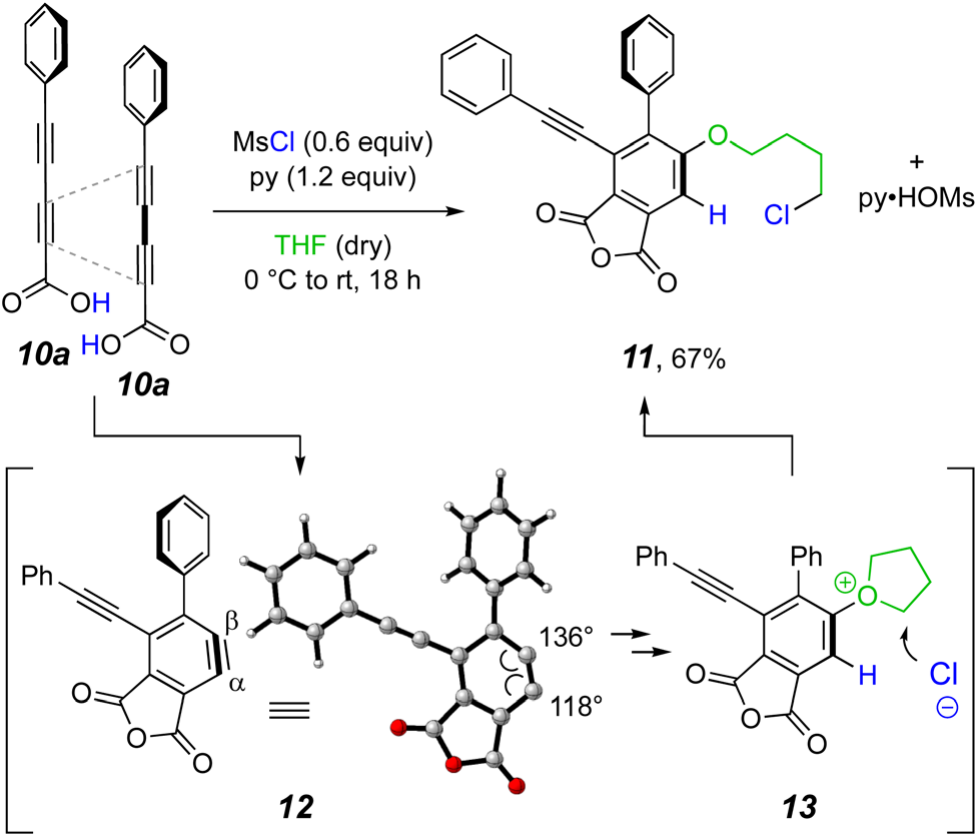
Our first example of *in situ*, MsCl-promoted condensation of the diynoic acid 10a (1.0 equiv.) to form an anhydride, which underwent rapid HDDA cycloisomerization. The intermediate electrophilic benzyne 12 was immediately trapped by a THF solvent molecule and the oxonium ion 13 was ring-opened by chloride ion.

Two aspects of any HDDA reaction are (i) that it requires the creation of a substrate containing a linked diyne/diynophile pair and (ii) that the rate of generation of the benzyne is fast relative to that of competing side reactions of the substrate and (trapped) benzyne-derived products. In light of the mild reaction conditions sufficient to induce the cyclization of the anhydride 2, we wondered what temperature would be required to induce HDDA cyclization of an anhydride such as that derived from dehydrative coupling of the conjugated diynoic acid 10a. In the first experiment (Fig. 2) a THF solution of phenylpentadiynoic acid (10a) was treated with a slight excess of MsCl and pyridine (because one molecule of the dehydrating agent MsCl converts two molecules of acid to anhydride, 0.6 molar equivalents is actually a 1.2 stoichiometrically equivalent ratio and, hence, a “slight excess.”) Within minutes even at 0 °C a precipitate as well as a blue, fluorescent TLC spot appeared. The latter was shown to be the product 11 following its isolation, indicating that we were in fact producing an HDDA benzyne at sub-ambient conditions! There was no evidence for the presumed intermediate anhydride. Also, there was no noticeable change in the TLC when the reaction mixture was allowed to further incubate overnight. The 4-chlorobutoxy group in 11 can be accounted for by the ring-opening of the oxonium ion 13.^15^ This, in turn, can arise from initial trapping of the HDDA benzyne 12 by THF at the more electrophilic carbon (C_β_) followed by protonation at the carbon atom of the initially formed zwitterionic oxonium-carbanion.

**Fig. 3:**
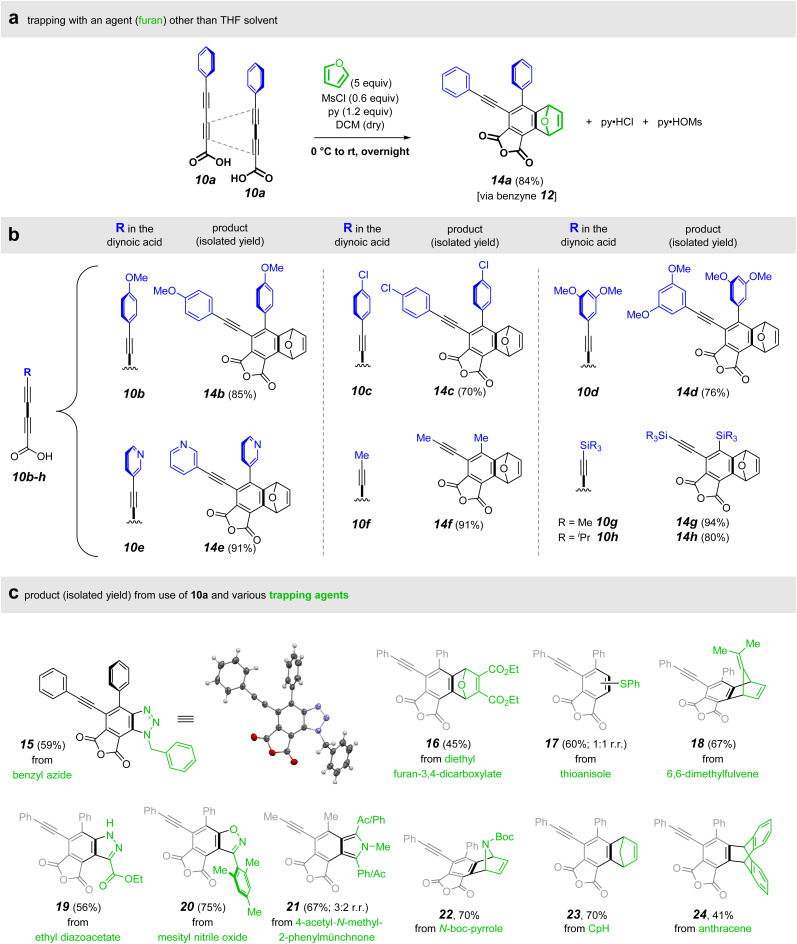
(a) Furan trapping of the *in situ*-generated benzyne 12 derived from 10a. A variety of (b) substituted diynoic acids 10b–h (blue) and (c) benzyne trapping agents (green) are compatible with the *in situ* anhydride formation/HDDA cyclization conditions.

By incubating 10a with MsCl and pyridine in a non-interacting solvent (DCM instead of the THF used in Fig. 2), we were able to establish reaction conditions that allowed for generalized benzyne trapping. The first example employing these conditions used furan as an external trap (Fig. 3a). This led to formation of the oxanorbornadiene derivative 14a in excellent yield. We observed that trifluoroacetic anhydride and methanesulfonic anhydride also were effective dehydrating agents, although we did not further explore this. As shown in Fig. 3b, this anhydride-HDDA process tolerates the presence of electron-rich and -poor aryl (10b–d), heteroaryl (10e), alkyl (10f), and silyl (10g–h) substituents on the terminus of the diyne carboxylic acid substrate.

We also used several other trapping agents (Fig. 3c): benzyl azide provided the triazole 15; thioanisole gave the sulfides 17; ethyl diazoacetate led to the pyrazole 19; mesityl nitrile oxide gave the isoxazole 20; a munchnone derivative gave rise to the isoindoles 21; and the simple dienes *N*-Boc-pyrrole, cyclopentadiene, and anthracene afforded the [4 + 2] cycloadducts 22, 23, and 24 respectively. We note that the tetrayne-derived products that contain an arylethynyl substituent on the phthalic anhydride scaffold show blue fluorescence, which represents further opportunity for investigation.

**Fig. 4:**
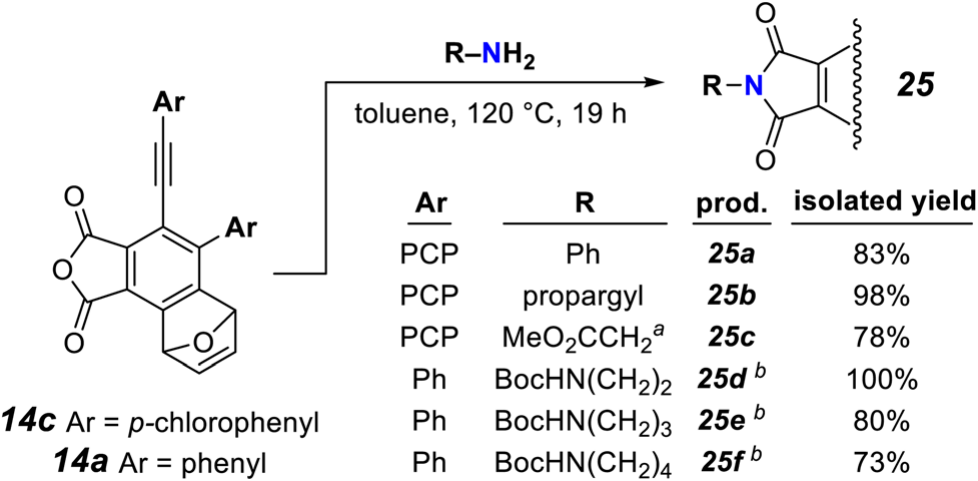
Facile condensations of the phthalic anhydride moiety in 14a/c with aniline, propargylamine, glycine methyl ester, and mono-boc diamines give imides 25a–f. ^*a*^Glycine methyl ester hydrochloride was used. ^*b*^Yield is of the deprotected primary amine following subsequent Boc removal (TFA, rt, 30 min).

Not surprisingly, the phthalic anhydride moiety in the products of these one-pot, *in situ*, anhydride assembly, cycloisomerization, and trapping cascades could be efficiently derivatized with primary amines to afford the phthalimide derivatives 25a–f (Fig. 4).^16^ Phthalic anhydrides are substrates for a considerable array of additional types of transformation.^16^ A recent report of anti-bacterial activities of similar phthalimide derivatives against plant pathogens is an example of contemporary interest in this class of compound.^17^

**Fig. 5:**
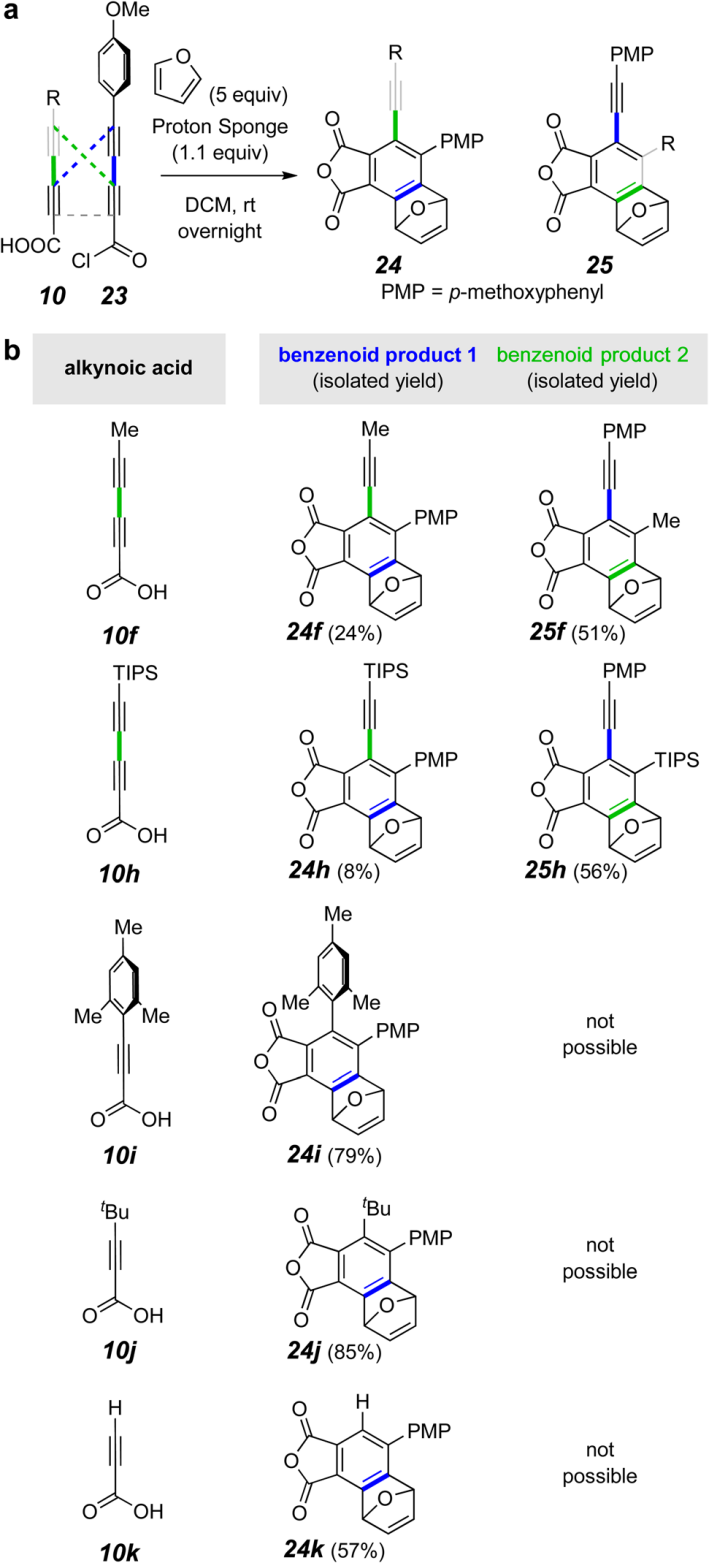
(a) Generic representation of the preparation of mixed anhydrides using the acid chloride 26 to produce more varied product structure motifs. (b) Examples include unsymmetrical anhydride HDDA substrates of both tetrayne (f, h) and triyne (i–k) classes. ^*a*^2.5 equiv. of 10k used to minimize the amount of 14b that was competitively formed.

Recognizing that the applicability of this strategy would be enhanced if we could selectively make and cyclize mixed (*i.e.*, unsymmetric) anhydrides, we explored conditions that might allow this. Using the MsCl-mediated conditions for anhydride formation from a mixture of two different diynoic acids, one would expect, of course, a relatively non-selective formation of products from the three possible homo- and hetero-anhydrides. This was confirmed in a preliminary experiment (^1^H NMR analysis of the crude product mixture^18^). Thus, we needed to find more-controlled, milder conditions for selectively forming a mixed anhydride that would persist sufficiently long for its HDDA cycloisomerization to take place. That is, conditions were needed that would not induce anhydride exchange of the unsymmetric anhydride, resulting in formation of unwanted symmetric anhydrides. We explored the use of an acid chloride/acid pair of substrates to generate the mixed anhydride. First, a dichloromethane solution of the preformed acid chloride 26 (structure in Fig. 5a) and the diyne acid 10f was treated with a slight excess of pyridine. Again^18^ we observed a more or less statistical mixture of the crossed and symmetrical products 27f, 28f, 14b, and 14f. This implied that pyridine and/or pyridinium chloride was catalyzing an unwanted anhydride metathesis event. We also examined the use of a silver(i) carboxylate salt to couple with the acid chloride 26.^19^ However, anhydride formation was thwarted by decarboxylation to give a terminal alkyne. We deemed other nucleophilic amine bases (*e.g.*, DBU, DMAP, or Et_3_N) to be poor choices because of the susceptibility of ynoyl chlorides or anhydrides to undergo either 1,2- or 1,4-addition to the ynoyl moiety by species containing a basic nitrogen atom.

Finally, we chose to explore the use of Proton-sponge® [1,8-bis(dimethylamino)naphthalene], a less nucleophilic, yet more basic amine than pyridine.^20^ This choice proved very rewarding (Fig. 5). Now the products derived from the unsymmetrical mixed anhydrides, arising from reaction of the acid chloride 26 with the acid 10f or 10h, were produced in good yield (Fig. 5b). Likewise, the triynes from 26 + 10i–k were efficiently formed as shown by product formation. These results indicate that scrambling of the initial mixed anhydride was slower than its HDDA cyclization using Proton-sponge®. In the case of the mixed tetraynes, two different benzynes were formed, accounting for the formation of product pairs 27f/28f and 27h/28h.

The triynes derived from the propiolic acid derivatives 10i–k also cyclized, now each to the furan adduct 27i–k from trapping of the only possible benzyne intermediate. Notice that formation of the mesityl-containing product 27i arises from an HDDA process even though a TDDA adduct could have been formed from the diradical intermediate common to both pathways. The presence of non-hydrogen substituents on C2 and C6 of the mesityl group significantly slows the TDDA mode of cyclization.^21^

**Fig. 6:**
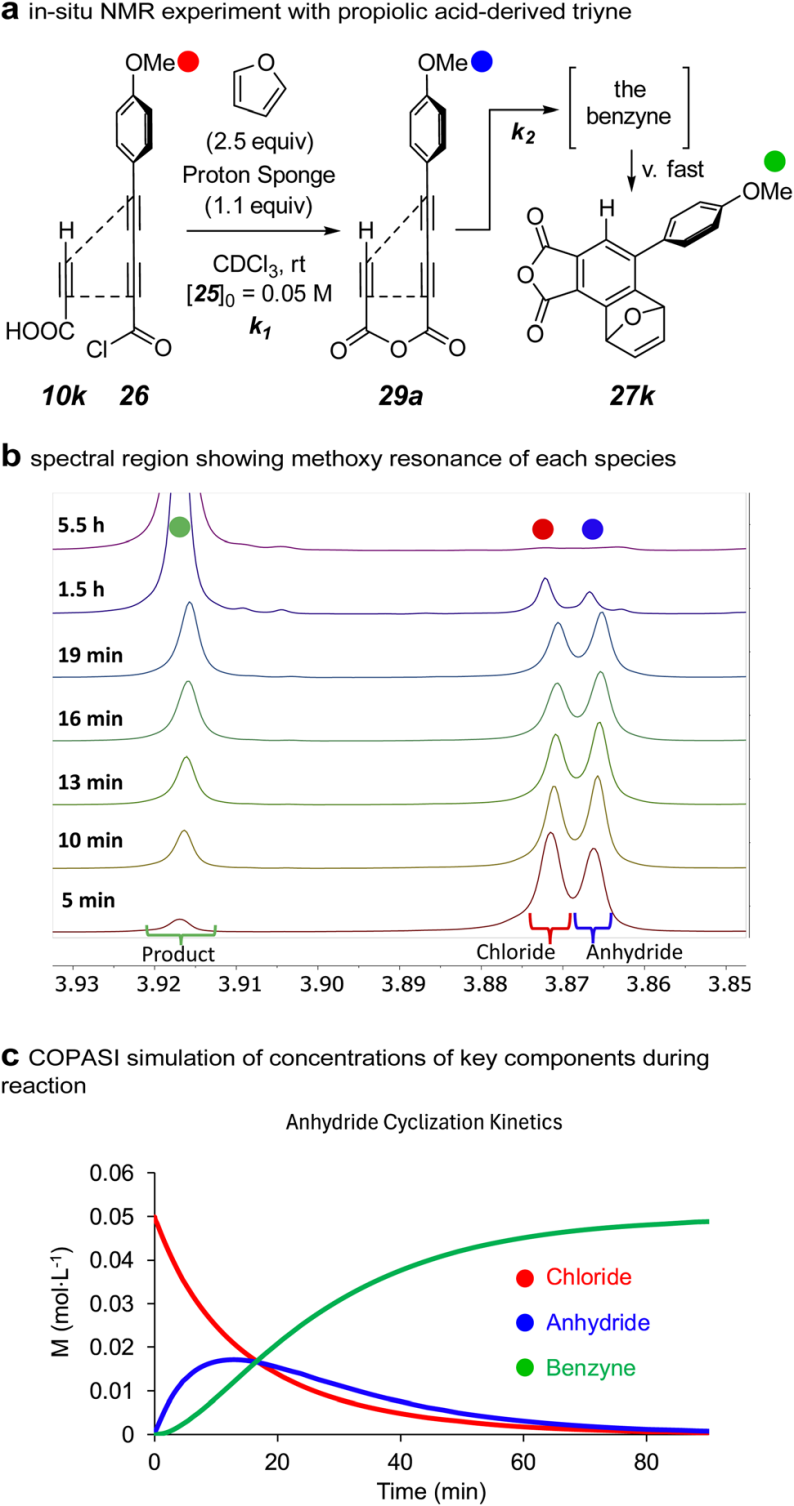
(a) The reaction used to measure the half-life of HDDA cyclization of the anhydride intermediate 29a. (b) *In situ* NMR data showing competitive formation and cyclization of the anhydride 29a. (c) COPASI simulation of the first 90 min of data shown in panel b.

A particularly striking result from the series of reactions involving of triyne anhydrides was the formation of 27k from propiolic acid. To the best of our knowledge, this is the first example of a triyne containing a terminal alkyne to undergo an HDDA cyclization at room temperature. The additional remote alkyne in a tetrayne enhances the rate of a HDDA cycloisomerization compared with an analogous triyne substrate.^22–24^ This is because the additional radical stabilizing energy (RSE) of the alkyne lowers the barrier in the rate-limiting, benzyne-forming event.^25^ Therefore, we wondered if the ability to make mixed anhydrides would provide an opportunity to now observe the formation of the triyne anhydride by *in situ*^1^H NMR analysis. Indeed, reaction of 10k and 26 in CDCl_3_ containing furan and Proton-sponge® allowed us to observe an acyclic anhydride (see 29a, Fig. 6a) for the first time. This species appeared and competitively disappeared as a transient intermediate enroute to the furan-trapped product 27k.

The ability to observe the mixed anhydride in the relatively less reactive triyne anhydride 29a (from 10k and 26, Fig. 6a) meant that it should be possible to assess the magnitude of the rate enhancement afforded by the anhydride linkage. A stack plot of spectra over time shows changes in the methoxy resonances of the PMP groups in the acid chloride, mixed anhydride, and final product 26, 29a, and 27k, respectively (Fig. 6b). Under the indicated conditions, the rate of formation of the anhydride, a bimolecular process, was observed to compete with that of its cyclization to the benzyne, which is not observed, of course, because of its extremely fast reaction with furan. The changes in the relative amounts of the three PMP-containing species over time were simulated with COPASI,^26^ which provided the data represented in Fig. 6c. From this the extracted half-life for the HDDA cyclization of 29a to the benzyne was 9 minutes.

**Fig. 7:**
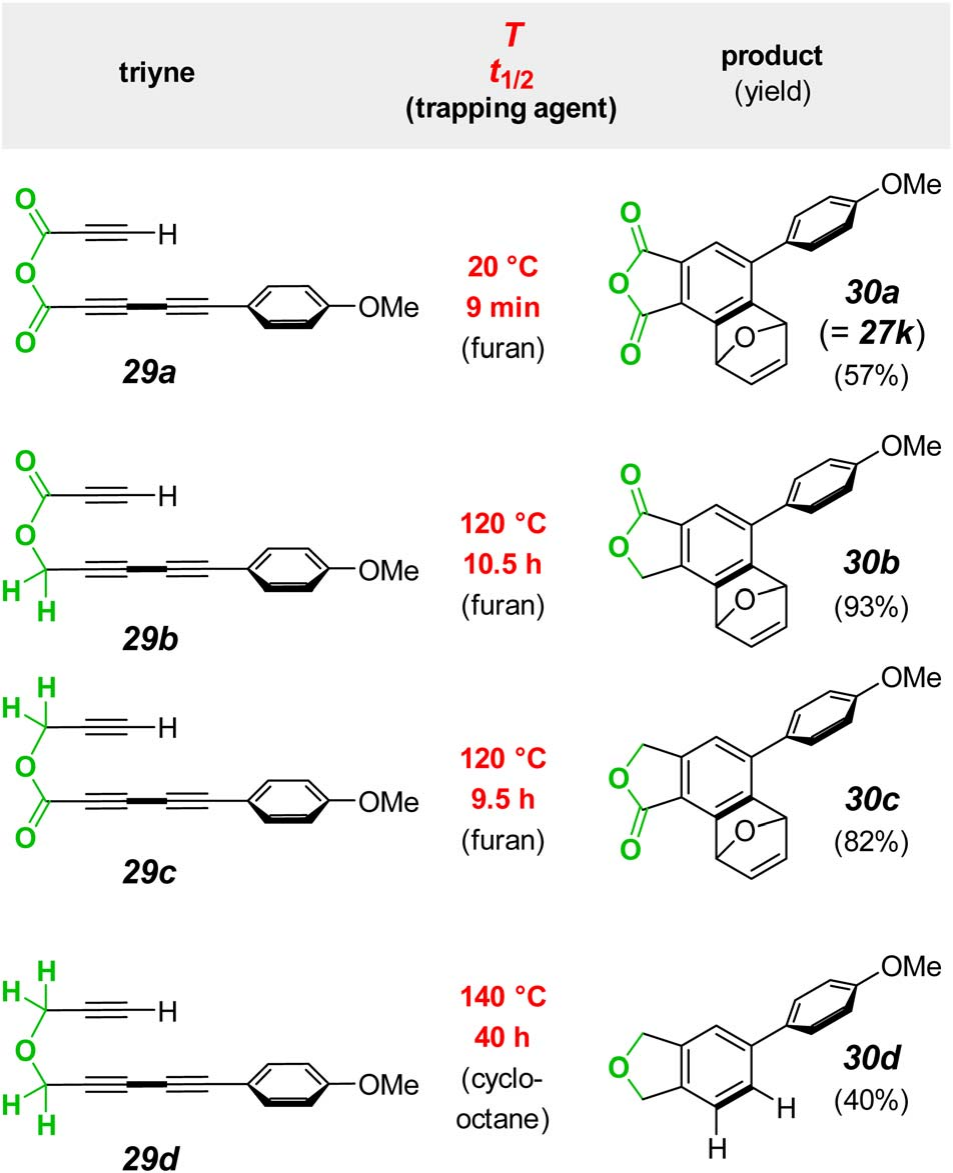
Large rate enhancement (red) for the HDDA cyclization of the triyne 29a having an anhydride tether compared to the rates of substates 29b–d having ester or ether tethers.

We next asked how the rate of the anhydride linked substrate 29a compared with that of the analogous ester- and ether-containing substrates 29b/c and 29d (Fig. 7). Because these latter three all cycloisomerize much more slowly than the anhydride analog, each was easily synthesized and purified. Each was then first converted preparatively into the HDDA benzyne-trapped products 30b/c (from reaction with furan) and 30d (from reaction with cyclooctane^27^).

**Fig. 8:**
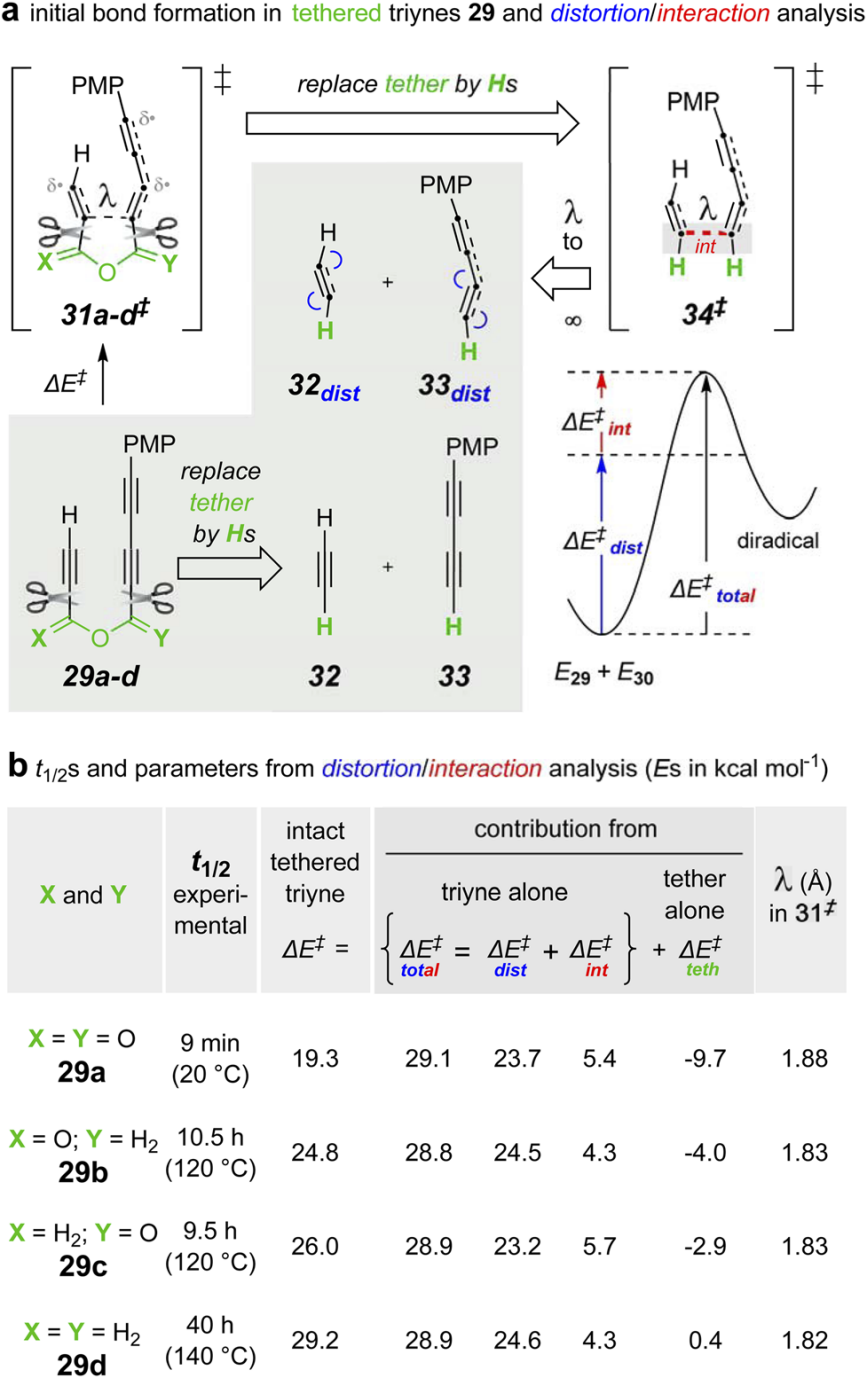
(a) Distortion/interaction analysis performed on the series of tethered triynes 29a–d. (b) Experimental half-lives and DFT^*a*^-computed parameters^*b*^ separating the energetic contributions from the triyne *vs.* those from the tether components to the overall barrier height for the initial, rate-limiting C–C bond formation in each of the triynes 29a–d. ^*a*^[(U)B3LYP-GD3BJ/6-311+G(d,p), SMD: chloroform]. ^*b*^Δ*E*^‡^ = *E*_31‡_ − *E*_32_‖Δ*E*^‡^_total_ = *E*_34‡_ – (*E*_32_ + *E*_33_)‖Δ*E*^‡^_dist_ = (*E*_32dist_ + *E*_33dist_) − (*E*_32_ + *E*_33_)‖Δ*E*^‡^_int_ = Δ*E*^‡^_total_ − Δ*E*^‡^_dist_‖Δ*E*^‡^_teth_ = Δ*E*^‡^ − Δ*E*^‡^_total_.

With NMR data for authentic samples of products 30b–d in hand, we proceeded to measure the half-life for the rate-limiting cycloisomerization of each of the triynes 29b–d in CDCl_3_ (see ESI† for the detailed protocol). Using a convenient temperature for the reaction rate of these much less reactive substrates (relative to the anhydride 29a), we used NMR spectroscopy to monitor the disappearance of each. The half -lives at 120 °C for 29b and 29c were very similar to one another (10.5 and 9.5 h, respectively) as well as to that seen for an analog of 29b in an earlier study.^13,23^ Substrate 29d having the ether linkage proved to be even less reactive. (All of the measured half-lives for 29a–d are also listed in column two of the tabulated data in Fig. 8b).

Clearly, the anhydride linker present in substrate 29a and, by extension, all of the other anhydrides involved in the reactions in Fig. 2, 3, and 5 are imparting a dramatic rate-acceleration on the HDDA cycloisomerization. Why? To address this question, we carried out a revealing distortion/interaction (aka, activation strain) analysis (Fig. 8a).^28^ Generally speaking and in the well-stated words of Bickelhaupt and Houk, “the activation strain or distortion/interaction model is a tool to analyze activation barriers that determine reaction rates. For bimolecular reactions, the activation energies are the sum of the energies to distort the reactants into geometries they have in transition states plus the interaction energies between the two distorted molecules. The energy required to distort the molecules is called the activation strain or distortion energy. This energy is the principal contributor to the activation barrier. The transition state occurs when this activation strain is overcome by the stabilizing interaction energy.”^28^

The specific approach used here involved first computing the energetic barrier for the initial C–C bond formation in each of 29a–d leading to the corresponding diradical (see ESI†) enroute to each benzyne. These computed activation energies (Δ*E*^‡^) are given in column 3 of Fig. 8b. They correlate well with the approximate relative rates of reaction of each of these four substrates (extrapolated from their reactions at different temperatures^29^). The tethering atoms in each computed structure of 31a–d^‡^ were then excised and replaced by a hydrogen atom to give rise to the four different structures of 34^‡^.^30^ In each of these, the internuclear distance *l* in 31^‡^ is maintained. The two “halves” of 31^‡^ were then separated to infinite distance, giving 32_dist_ and 33_dist_, the distorted but non-interacting, free alkyne components. To identify the distortion energy developed in the transition structures (TSs) 31^‡^, the tether was also excised from 32 to produce, following optimization, the undistorted alkyne components 32 and 33. The difference in (the single point) energies between 32_dist_*vs.*32 and 33_dist_*vs.*33 comprise the total distortion energy (Δ*E*^‡^_dist_) Likewise, the difference in energy between 34^‡^ and 32_dist_ + 33_dist_ gives the interaction energy for each of the four analogs.

Notably, the values of both the Δ*E*^‡^_dist_ and the Δ*E*^‡^_int_ [as well as, therefore, their sum Δ*E*^‡^_total_] were nearly identical for all four sets of structures (columns 4–6, Fig. 8b). Moreover, the interaction energy in 34^‡^ for each of the four reactions was destabilizing. This is an uncommon, but not unprecedented,^31^ phenomenon, including an example seen for a computed, stepwise HDDA reaction.^32^ The implication is that at the distances and geometries between the alkyne moieties in 34^‡^ (and 31^‡^), the alkynes have more destabilizing, repulsive interaction than stabilizing, bonding interaction. The energetic contribution imposed/provided by the tether Δ*E*^‡^_teth_ is, therefore, the difference in activation energies between the intact tethered substrates 29a–d proceeding to 31a–d^‡^ (Δ*E*^‡^) *vs.* the untethered analogs 32 and 33 proceeding to 34a–d^‡^ Δ*E*^‡^_total_.

These are significantly different across the series of tethers (column 7, Fig. 8b). Relative to the ether in 29d, the anhydride in 29a provides *ca.* 10 kcal mol^−1^ greater stabilization to its transition structure.

**Fig. 9:**
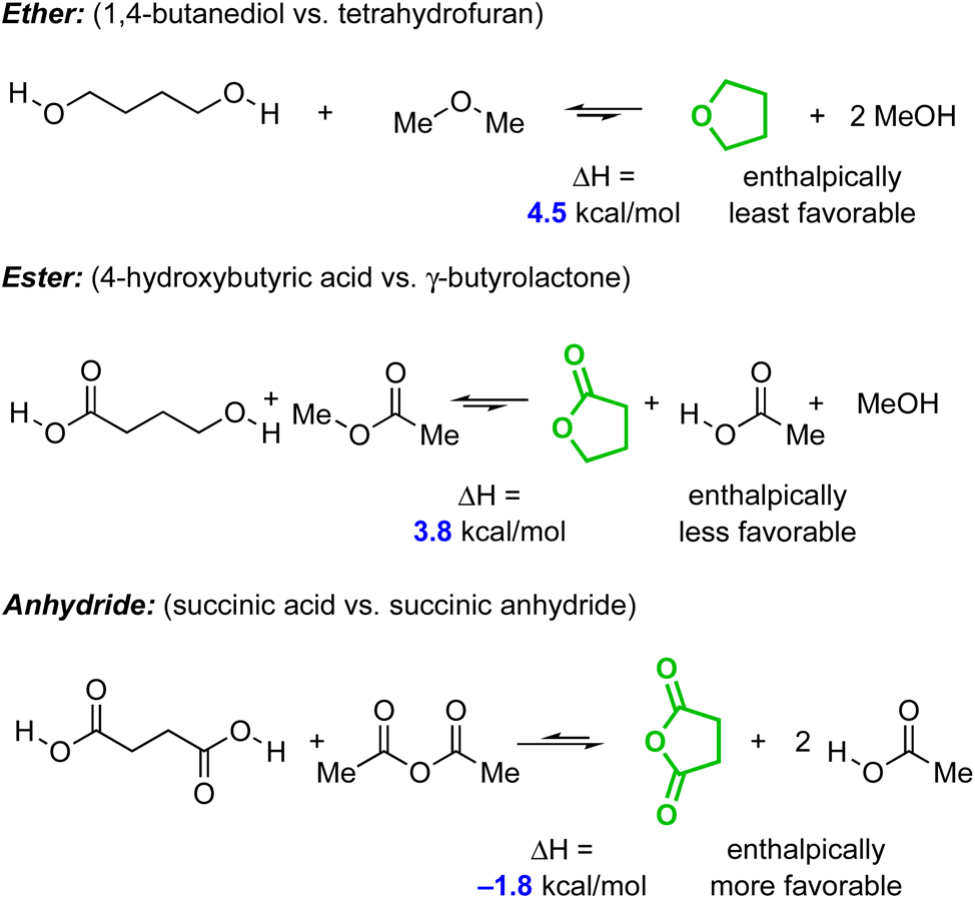
Computed^*a*^ enthalpic change for a series of homodesmotic reactions showing the unique favorability (−1.8 kcal mol^−1^) afforded by conversion of the acyclic to cyclic anhydride compared to the unfavorable enthalpies of cyclization for the ether and ester analogs (+4.5 and +3.8 kcal mol^−1^, respectively). ^*a*^[(MN15/6-311++G(d,p), SMD : water].

At a more fundamental level, the question remains: why is the anhydride so different? Two factors may be contributing. First, nearly all acyclic anhydrides, including the HDDA substrates here, are composed (as judged by computation as well as microwave and rotational–vibrational spectroscopies) of a family of non-coplanar conformations^33^ (the exception being formic anhydride^34^). In the phthalic anhydride products produced here, the enforced planarity of the anhydride could lead to a greater degree of stabilization (resonance and/or strain?)^35^ relative to the ester-to-lactone and ether-to-ether cyclizations. To probe that possibility, we examined the simple set of homodesmotic reactions (note the same number and type of functional groups on each side of the reaction equation) shown in Fig. 9. The enthalpy change between the (all-anti conformer of the) ring-opened *vs.* the ring-closed forms for each of the three functional groups (*i.e.*, ether, *vs.* ester, *vs.* anhydride) was computed with DFT. The magnitudes of the differences in the Δ*H*'s across this series of cyclizations correlate well with the differences in the rates of the HDDA cyclizations. This reinforces the conclusions from the distortion/interaction analysis. That is, that the primary differentiating factor in the large differences in reactivity for the series 29a–d is the enthalpy change associated with each tether as it undergoes the cyclization, the anhydride being uniquely favorable.

**Fig. 10:**
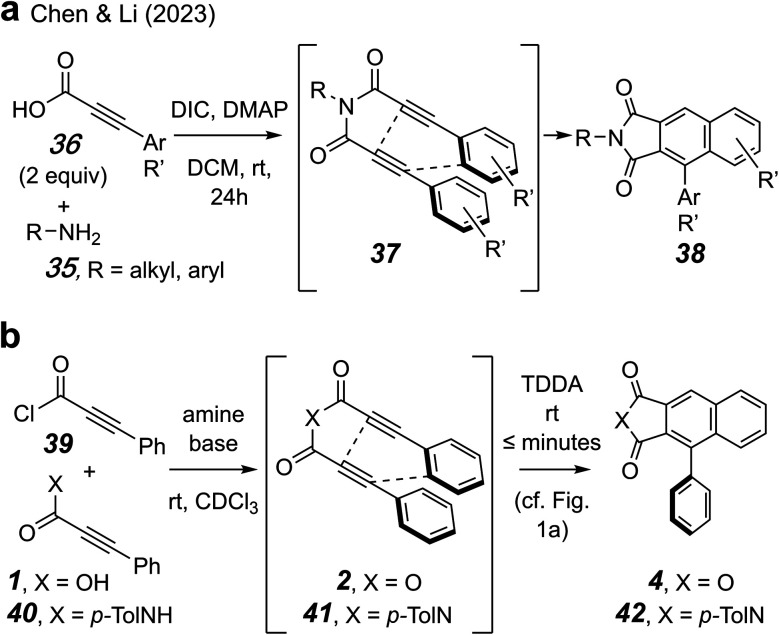
Tetradehydro-Diels–Alder (TDDA) reactions of propiolic acid chloride-derived anhydride- and imide-linked TDDA substrates 2 and 37. The imide cyclizes faster than the anhydride.

Finally, a 2023 report from the Chen and Li groups at Guizhou University describes a robust method for preparing symmetrical *N*,*N*-bis-(3-aryl)propiolylimides that cyclize at ambient temperature to give naphthalimide derivatives 38 (Fig. 10a).^17^ Precursor amines 35 were acylated with two equivalents each of an arylpropiolic acid 36 and *N*,*N*′-diisopropylcarbodiimide (DIC). The intermediate imides 37 spontaneously underwent a TDDA reaction (*cf.*Fig. 1a) to produce products 38 in very good to excellent yields. Over four dozen examples were reported. All reactions were performed at ambient temperature for 24 h.

A reviewer of our manuscript asked whether there was any other type of linker that would accelerate a cyclization by virtue of the planarization phenomenon seen with the anhydride. Because the imide-templated reaction from Chen and Li represents such a linker and especially because it bears considerable similarity to the anhydride work disclosed here, we were curious about the actual extent to which an imide would accelerate this type of cycloisomerization. Toward that end, we prepared, *in situ*, the symmetrical TDDA substrates, phenylpropiolic anhydride (2) and *N*-(*p*-tolyl)-*N*,*N*-bis-(3-phenylpropiolyl)imide (41) (Fig. 10b). We used ^1^H NMR spectroscopy to monitor reactions in CDCl_3_ between phenylpropiolic acid chloride (39) with either 3-phenylpropiolic acid (1) in the presence of Proton-sponge® or 3-phenyl-*N*-(*p*-tolyl)propiolamide (40) in the presence DMAP/Et_3_N.^36^ The rate of the TDDA cycloisomerization of the imide 41 to produce the naphthalimide derivative 42 was significantly faster than that of the anhydride 2 to give 4 (see ESI†). Further studies to more fully evaluate and quantify the extent of this even greater rate acceleration by an imide are planned. We note in closing that this strategy for imide formation is readily amenable to the preparation of unsymmetrical *N*,*N*-bis-(propiolyl)imides.

## Conclusion

We have demonstrated here the first example of HDDA cycloisomerization of a poly-yne precursor possessing an anhydride linker *via* dehydration of shelf-stable carboxylic acid diynes. Substrates containing the anhydride have proven to show much higher than expected cyclization rates. In accordance with, a perhaps underappreciated, 50^10^–125^2^ year-old precedent of the facile TDDA reaction of phenylpropiolic acid anhydride (Fig. 1a), the HDDA variant produces benzyne at or below room temperature (including, for the first time, a substrate having a terminal propargylic alkyne serving as the diynophile). This expands the scope of conditions under which HDDA-benzyne formation and trapping can be performed. Use of MsCl as a dehydrating agent at room temperature leads to formation of symmetric anhydrides derived from dimerization of a variety of diynoic acids. These quickly proceed to benzyne formation and *in situ* trapping to form various phthalic anhydride derivatives, including a number with fused heterocyclic moieties. Post-HDDA modifications of several of the phthalic anhydride products to their phthalimide derivatives demonstrate the ease with which potential other polar moieties can be introduced. By employing a diynoic acid chloride, we further identified conditions that allowed for selective formation of the benzyne derived from mixed anhydrides, further expanding the modularity of the approach.

We also deduced the reason for the rate enhancement afforded by the anhydride linker. By synthesizing a series of triyne substrates that varied only in their linker structure (ether, ester, and anhydride), we found that the rate of HDDA cycloisomerization increased, dramatically, in the stated order (estimated to be >10^7^ compared to the analogous ether-linked substrate^29^). To rationalize this trend, we performed a distortion/interaction analysis that revealed that: (i) the distortion/interaction within the diyne–diynophile reacting atoms was nearly identical for all three classes of substrate, and (ii) all four substrates have a destabilizing (*i.e.*, positive) interaction energy at the internuclear distance present in the computed transition structure of each.

A series of simple homodesmotic calculations were performed to assess the enthalpy difference between ring-opened *vs.* -closed forms for THF, γ-butyrolactone, and succinic anhydride. This showed that it is the energetic contribution associated with the formation of the fused, cyclic five-membered anhydride in the benzyne intermediate that is the principal contributor to the substantially higher rate of the HDDA cycloisomerization. Thus, the differences in stabilization of the transition structures across the series of four substrate classes arise nearly entirely from changes within the reorganization of the tethering atoms. This understanding explains why that, in this setting, the anhydride tether is a kinetically privileged linker. Finally, we have observed that an *N*-aryl imide analog of the anhydride linker induces an even greater rate acceleration.

## Data availability

The data upon which the conclusions in this manuscript are based are provided in the ESI† document and in a .zip file of a master Mnova file of all NMR spectra.

## Author contributions

D. S. S. conceived the project, effected the initial examples, and carried out the computational studies, D. S. S. and P. V. K. carried out similar amounts of the experimentation, and all three authors analyzed the data and co-wrote the manuscript.

## Conflicts of interest

There are no conflicts to declare.

## Supplementary Material

SC-016-D4SC07232D-s001

SC-016-D4SC07232D-s002

SC-016-D4SC07232D-s003
