# Supplementary material for: Rapid (≤25 °C) cycloisomerization of anhydride-tethered triynes to benzynes – origin of a remarkable anhydride linker-induced rate enhancement
Source: Chem Sci. 2025 Jan 7;16(6):2898–906. doi: 10.1039/d4sc07232d (PMC11734507; doi:10.1039/d4sc07232d)
Supplement: SC-016-D4SC07232D-s001 [file SC-016-D4SC07232D-s001.pdf]

Supplementary Information (SI) for Chemical Science.
This journal is © The Royal Society of Chemistry 2025

Supporting Information for

Rapid (≤25 °C) Cycloisomerization of Anhydride-tethered Triynes to Benzynes – Origin of a Remarkable Anhydride Linker-induced Rate Enhancement

Dorian S. Sneddon, Paul V. Kevorkian, and Thomas R. Hoye*

Department of Chemistry, University of Minnesota, 207 Pleasant St. SE, Minneapolis, Minnesota 55455

Email: hoye@umn.edu

Table of Contents

I. General Experimental Protocols ···· **S4**

II. General Experimental Procedures ···· **S5-S7**

III. Experimental Procedures and Characterization Data for All New Compounds ···· **S8-S59**

IV. Kinetics Data for Compounds 27k (=29a), 29b, 29c, and 29d ···· **S60-S70**

V. Discussion of Computational Results ···· **S71-S110**

VI. X-Ray Crystallographic Data for Compound 15 ···· **S111-S113**

VII. References for the Supporting Information ···· **S114-S116**

VIII. Copies of 1D and 2D NMR Spectra ···· **S117-S302**

- **S1** ^{1}H ···· S117
- **S2** ^{1}H ···· S118
- **S3** ^{1}H ···· S119
- **S4** ^{1}H ···· S120
- **S5** ^{1}H ···· S121
- **S6** $^{1}H/^{13}C\{^{1}H\}$ ···· S122-S123
- **S7** ^{1}H ···· S124
- **S8** $^{1}H/^{13}C\{^{1}H\}$ ···· S125-S126
- **S10** ^{1}H ···· S127
- **S11** $^{1}H/^{13}C\{^{1}H\}$ ···· S128-S129
- **S12** ^{1}H ···· S130
- **S14** ^{1}H ···· S131

S15 ^{1}H ········ S132
S16 ^{1}H ········ S133
S17 ^{1}H ········ S134
S18 ^{1}H ········ S135
S19 ^{1}H ········ S136
S20 ^{1}H ········ S137
S21 ^{1}H ········ S138
10a ^{1}H ········ S139
10b $^{1}H/^{13}C\{^{1}H\}$ ········ S140-S141
10c $^{1}H/^{13}C\{^{1}H\}$ ········ S142-S143
10d $^{1}H/^{13}C\{^{1}H\}$ ········ S144-S145
10e $^{1}H/^{13}C\{^{1}H\}$ ········ S146-S147
10f $^{1}H/^{13}C\{^{1}H\}$ ········ S148-S149
10g $^{1}H/^{13}C\{^{1}H\}$ ········ S150-S151
10h $^{1}H/^{13}C\{^{1}H\}$ ········ S152-S153
11 $^{1}H/^{13}C\{^{1}H\}$/COSY/NOESY/HSQC/HMBC ········ S154-S159
14a $^{1}H/^{13}C\{^{1}H\}$ ········ S160-S161
14b $^{1}H/^{13}C\{^{1}H\}$/NOESY/HSQC/HMBC ········ S162-S166
14c $^{1}H/^{13}C\{^{1}H\}$/COSY/NOESY/HSQC/HMBC ········ S167-S172
14d $^{1}H/^{13}C\{^{1}H\}$/NOESY/HSQC/HMBC ········ S173-S177
14e $^{1}H/^{13}C\{^{1}H\}$/COSY/NOESY/HSQC/HMBC ········ S178-S183
14f $^{1}H/^{13}C\{^{1}H\}$ ········ S184-S185
14g $^{1}H/^{13}C\{^{1}H\}$ ········ S186-S187
14h $^{1}H/^{13}C\{^{1}H\}$ ········ S188-S189
15 $^{1}H/^{13}C\{^{1}H\}$/COSY/NOESY/HSQC/HMBC ········ S190-S195
16 $^{1}H/^{13}C\{^{1}H\}$ ········ S196-S197
17a $^{1}H/^{13}C\{^{1}H\}$/NOESY/HSQC/HMBC ········ S198-S202
17b $^{1}H/^{13}C\{^{1}H\}$/NOESY/HSQC/HMBC ········ S203-S207
18 $^{1}H/^{13}C\{^{1}H\}$ ········ S208-S209
19 $^{1}H/^{13}C\{^{1}H\}$/COSY/NOESY/HSQC/HMBC ········ S210-S215
20 $^{1}H/^{13}C\{^{1}H\}$/NOESY/HSQC/HMBC ········ S216-S220

21a $^{1}H/^{13}C\{^{1}H\}$/NOESY/HSQC/HMBC S221-S225
21b $^{1}H/^{13}C\{^{1}H\}$/NOESY/HSQC/HMBC S226-S230
22 $^{1}H/^{13}C\{^{1}H\}$/NOESY/HSQC/HMBC S231-S235
23 $^{1}H/^{13}C\{^{1}H\}$/NOESY/HSQC/HMBC S236-S240
24 $^{1}H/^{13}C\{^{1}H\}$/NOESY/HSQC/HMBC S241-S245
25a $^{1}H/^{13}C\{^{1}H\}$ S246-S247
25b $^{1}H/^{13}C\{^{1}H\}$ S248-S249
25c $^{1}H/^{13}C\{^{1}H\}$ S250-S251
25d $^{1}H/^{13}C\{^{1}H\}$ S252-S253
25e $^{1}H/^{13}C\{^{1}H\}$ S254-S255
25f $^{1}H/^{13}C\{^{1}H\}$ S256-S257
26 $^{1}H/^{13}C\{^{1}H\}$ S258-S259
27f $^{1}H/^{13}C\{^{1}H\}$ S260-S261
27h $^{1}H/^{13}C\{^{1}H\}$/NOESY/HSQC/HMBC S262-S266
27i $^{1}H/^{13}C\{^{1}H\}$/NOESY/HSQC/HMBC S267-S271
27j $^{1}H/^{13}C\{^{1}H\}$/NOESY/HSQC/HMBC S272-S276
27k $^{1}H/^{13}C\{^{1}H\}$ S277-S278
28f $^{1}H/^{13}C\{^{1}H\}$ S279-S280
28h $^{1}H/^{13}C\{^{1}H\}$/NOESY/HSQC/HMBC S281-S285
29b $^{1}H/^{13}C\{^{1}H\}$ S286-S287
29c $^{1}H/^{13}C\{^{1}H\}$ S288-S289
29d $^{1}H/^{13}C\{^{1}H\}$ S290-S291
30b $^{1}H/^{13}C\{^{1}H\}$ S292-S293
30c $^{1}H/^{13}C\{^{1}H\}$ S294-S295
30d $^{1}H/^{13}C\{^{1}H\}$ S296-S297
42 $^{1}H/^{13}C\{^{1}H\}$/NOESY/HSQC/HMBC S298-S302

I. General Experimental Protocols

^{13}C and ^{1}H NMR spectra were recorded on a Bruker Avance III (HD-500) spectrometer. Chemical shifts for spectra in $CDCl_3$ are referenced to TMS at δ 0.00 ppm; spectra in DMSO-d_6 are referenced to 2.50 for the residual proton in C_2D_5HOS. "A non-first order multiplet, doublet, or doublet of doublets in a ^{1}H NMR spectrum are denoted as 'nfom', 'nfod', or 'nfodd,' respectively. Multiplets are described by: chemical shift (ppm) [multiplicity, coupling constant(s) in Hz, integral value to the nearest integer, and assignment of the environment within the structure by indicating neighboring atoms or by numbering of the carbon atom to which the proton is attached]. Analysis of coupling constants was done using methods published previously.[1,2] ^{13}C NMR chemical shifts are those measured in the 1D spectrum. Carbon chemical shifts in $CDCl_3$ are referenced to δ 77.16 ppm.

Infrared spectra were taken on a Bruker Alpha II Spectrometer in the attenuated total reflectance (ATR) mode. Absorption maxima are given in cm^{-1}. The samples were prepared as thin films made by evaporation of a DCM solution on a diamond window.

Medium pressure liquid **chromatography** (MPLC) was used to purify most new compounds. Hand-packed silica gel columns (Teledyne RediSep Rf Gold®; normal-phase, 20–40 μm, 60 Å pore size) were used. The apparatus was constructed with a HPLC pump (Waters model 510), differential refractive index detector (Waters R401), and UV detector (Gilson 111 UV). Preparative flash chromatography was done on Agela silica gel (230–400 mesh). Thin layer chromatography (TLC) was carried out using silica-gel coated, aluminum-backed plates that were visualized first by UV light and, then, by staining with a solution of $KMnO_4$ and heating.

Reaction temperatures refer to the temperatures of an external heating oil bath or block heater. HDDA reactions, including reactions at temperatures higher than the that of the boiling point of the solvent, were done in a screw-top culture tube that was capped with an inert Teflon®-lined closure."[3]

High-resolution **mass spectrometry** (HRMS) was done in ESI-TOF ionization mode on a Thermo Orbitrap Velos instrument that has a mass accuracy of ≤3 ppm. The external calibrant was Pierce™ LTQ. The samples were introduced directly into the ion source. Compounds of lower molecular weight were analyzed on an Agilent 7200 GC/QTOF-MS in electron ionization (EI) mode; $(C_4F_9)_3N$ was used as an external calibrant.

Melting points were recorded as a range with the first number representing the initial point of liquification (or degradation) of the crystal and the final point being that at which full liquification or degradation was observed. For assessing crystallinity and taking melting points up to 212 °C, a Bristoline Bristolscope microscope with polarizing filter and Kofler hot-stage coupled to a Variac and Traceable digital thermometer was used. For melting points between 212 °C and 400 °C, a Mel-Temp apparatus was employed.

II. General Experimental Procedures

Protocol for synthesis of aryl carboxylic acid diynes

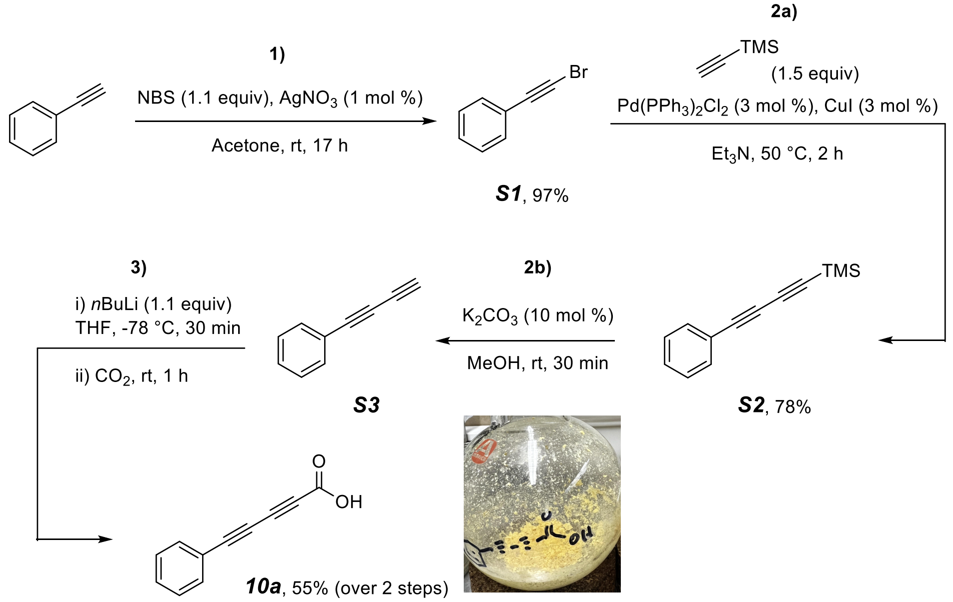

A three-step synthetic protocol was devised to synthesize aryl carboxylic acid diynes, exemplified by the scheme for the phenyl derivative **10a**. NBS bromination of commercial phenylacetylene (Oakwood Chemical) furnished the bromoalkyne **S1**.[4] Subsequent Sonogashira cross-coupling with TMS-acetylene afforded the silylated diyne in 78% yield.[5] The terminal diyne **S2** is reported to be unstable upon concentration and/or heating, so care was taken to carry it forward into the next transformation.[4] The methanolic reaction solution was rotary evaporated in an ambient temperature water bath to ca. 25% of its initial volume, water was added, and the mixture was extracted with diethyl ether three times. The combined organic extracts were dried over brine and sodium sulfate. The ethereal solution was then evaporated under vacuum, again at ambient temperature, and taken up in dry tetrahydrofuran for the subsequent transformation. Finally, a reported carboxylation protocol afforded the aryl carboxylic acid diyne **10a** (for apparatus setup, see Figure S1).[6] Copies of the proton NMR spectra for the bromoalkyne **S1**, the terminal diyne **S2**, and aryl diyne carboxylic acid **10a** matched with those reported in the literature and have been provided for readers' convenience. For (hetero)aryl acetylenes that proved recalcitrant to bromination, an alternative three-step synthesis protocol was devised involving Cadiot-Chodkiewicz coupling to the protected heteroaryl butadiyne followed by basic deprotection in refluxing toluene to access the corresponding terminal diyne.

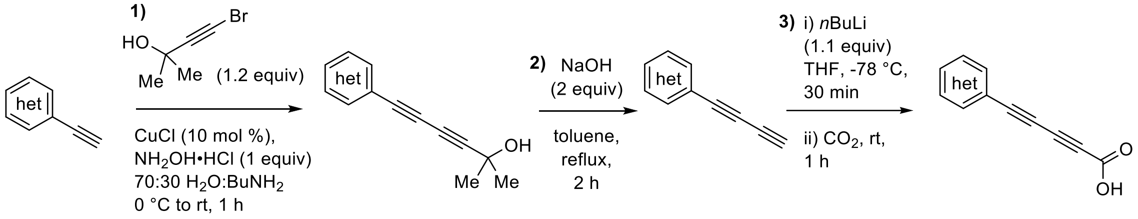

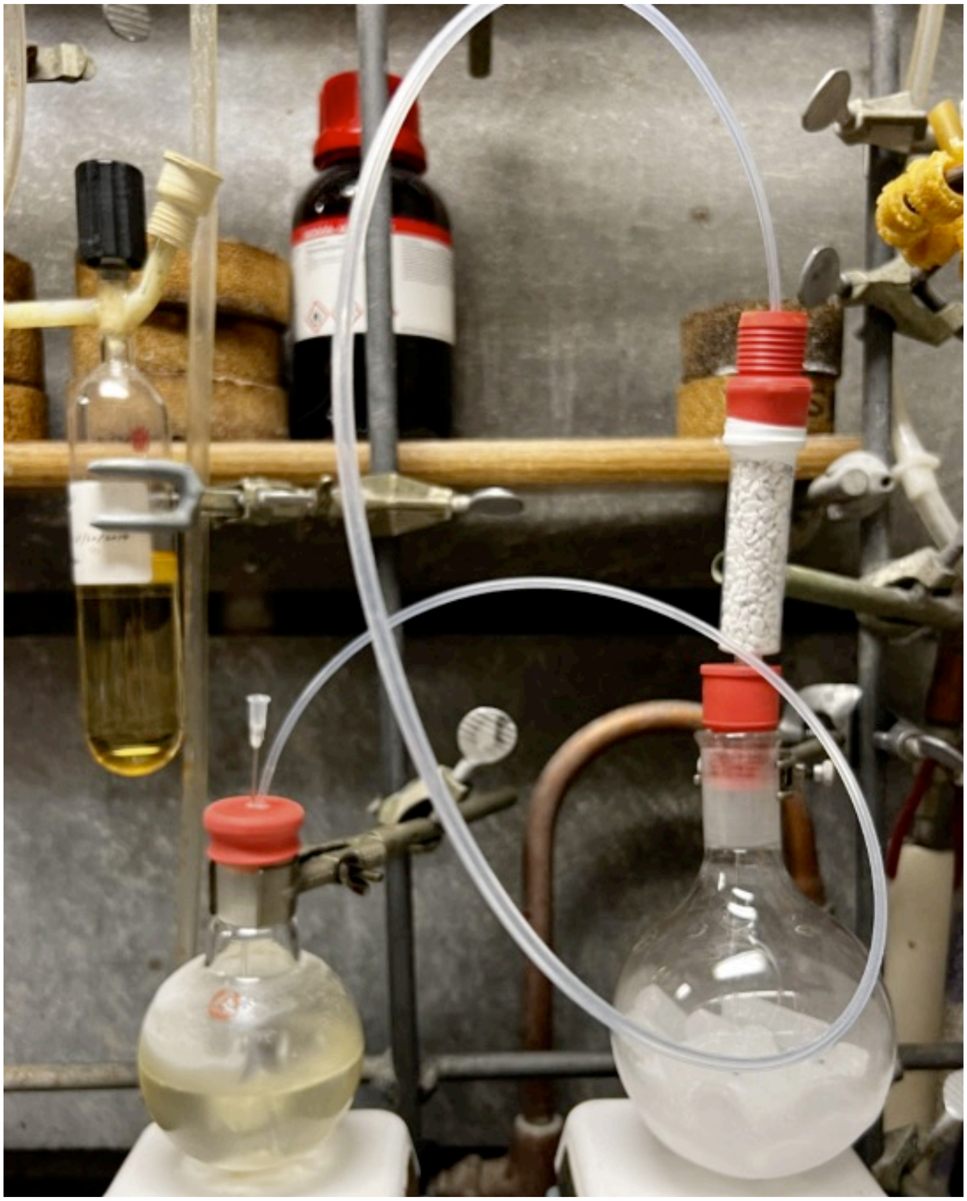

Figure S1. An oven dried round-bottom flask was capped with a septum and taken to a storage cooler in which blocks of dry ice was kept. Chunks of dry ice were taken from underneath the top layer (*Caution*: Carbon dioxide inhalation in large amounts can lead to problems; it is advisable that the researcher be accompanied by a second person in case there is any such issue.) To perform the carboxylation of terminal diynes, the flange was cut from a 20 mL plastic syringe. The syringe was filled with desiccant and capped with a rubber septum and sealed with Teflon tape. The luer fitting was attached to a needle and inserted into a flask containing dry ice (right). A length of 1/8" d. Teflon tubing was bevel-cut with a razor blade at both ends. The septa on the flange side of the plastic syringe and on the reaction vessel was pierced, and the Teflon cannula was used to connect the reaction flask (left) with the CO_2 generator flask. An outlet vent needle was inserted into the the reaction vessel septum, which allowed for a steady and efficient bubbling of dry carbon dioxide through the reaction solvent.

A. General procedure for synthesis of carboxylic acid-derived HDDA benzenoids

A round-bottom flask was flame-dried and evacuated/backfilled with dry nitrogen three times. The diynyl carboxylic acid was added to the vessel and dry dichloromethane was added (0.05 M). A benzyne trapping agent of choice (5 equiv) was added, a nitrogen balloon was affixed to the flask, and the DCM solution or suspension was cooled to 0 °C. Methanesulfonyl chloride (0.6 equiv) was added dropwise to the stirring solution or suspension, followed by dropwise addition of distilled pyridine (1.2 equiv). The reaction vessel was then warmed to ambient temperature; a precipitate began to appear as the reaction mixture warmed. As a matter of course, the reaction mixture was typically allowed to stir overnight, but depending on the substrate structure the reaction was often complete in a matter of ≤ a few hours. Pyridinium salts were filtered by passing the reaction suspension through a silica gel plug (1:1 Hex:EtOAc or 100% EtOAc eluent), the solvent was removed by rotary evaporation, and the crude material was purified by MPLC.

B. General procedure for synthesis of phthalimides from HDDA-derived phthalic anhydrides

The HDDA-derived phthalic anhydride derivative was added to a screw-cap culture tube and dissolved in toluene (0.05 M). A primary amine was added (1.2–2.4 equiv) and the reaction vessel was heated at 120 °C overnight. The reaction solvent was removed by rotary evaporation, and the crude material was purified by MPLC.

C. General procedure for synthesis of alkylamino phthalimides from HDDA-derived phthalic anhydrides

The HDDA-derived phthalic anhydride derivative was added to a screw-cap culture tube and dissolved in toluene (0.05 M). A mono-Boc-protected alkyl diamine was then added (1.1 equiv) and the reaction vessel was heated at 120 °C until completion of the reaction was observed by thin-layer chromatography. The reaction solvent was removed by rotary evaporation and the crude material was taken up in 1:1 DCM:TFA (0.05 M). After 10 minutes, aqueous NaOH (2 M) was added until the aqueous layer remained basic. The aqueous layer was extracted with DCM and dried over brine and $MgSO_4$. The crude material was then passed through a silica plug, eluting impurities first with EtOAc, and eluting the product alkylamine with MeOH.

D. General procedure for preparation of unsymmetrical anhydrides.

A round-bottom flask was flame-dried and evacuated/backfilled with dry nitrogen three times. The diynoic acid chloride (1.1 equiv), Proton-sponge® (1.1 equiv), and furan (5 equiv) were added to the vessel and dissolved in DCM (to achieve 0.04 M). The carboxylic acid (1 equiv) was added to the mixture in one portion and stirred at room temperature for 17 h. The resulting mixture was filtered through a plug of silica (100% EtOAc) and purified by MPLC. **Note**: Carboxylic acids suspected to contain moisture, namely **10f** and **10h**, generally resulted in higher levels of scrambling to form symmetric HDDA adducts. This was mitigated by incubating a solution of the acid in DCM over activated 3 Å molecular sieves overnight.

III. Experimental Procedures and Characterization Data for All New Compounds

1-(Buta-1,3-diyn-1-yl)-4-chlorobenzene (S6)

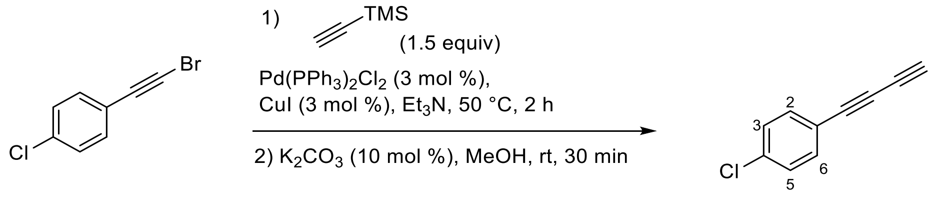

S5 ***S6***, 73%

1-(Bromoethynyl)-4-chlorobenzene (**S5**, 6.00 g, 27.8 mmol, 1 equiv) was prepared according to a literature procedure from commercial 1-chloro-4-ethynylbenzene (Oakwood Chemical);[7] the proton NMR spectrum of **S5** has been provided in the SI.

1) To a 500 mL round-bottom flask, *bis*(triphenylphosphine)palladium(II) dichloride (586 mg, 0.835 mmol, 0.03 equiv), copper (I) iodide (159 mg, 0.835 mmol, 0.03 equiv), and a magnetic stir bar were added. The flask was then fitted with a rubber septum, evacuated, and backfilled with nitrogen three times. Triethylamine (200 mL) was degassed by gently bubbling nitrogen through it for ca. 5 minutes. This solution was then added to the reaction solids via syringe and the resultant solution was degassed again for ca. 5 minutes. Next, TMS-acetylene (5.95 mL, 41.7 mmol, 1.5 equiv) was added via syringe to the reaction solution, and a nitrogen balloon was attached. The reaction mixture was allowed to stir at 50 °C for 2 hours, at which point the reaction was deemed complete by GC-MS analysis. The crude reaction solution was passed through a bed of Celite and concentrated. The crude material was then purified by flash chromatography (hexanes) and carried forward directly to the desilylation of the terminal diyne.

2) The TMS-alkyne was dissolved in 100 mL of MeOH. To this solution was added K_2CO_3 (385 mg, 2.79 mmol, 0.1 equiv) and the mixture was allowed to stir for 30 min at ambient temperature, after which time, the reaction was judged to be complete by GC-MS analysis. After evaporation of the solvent, the crude material was taken up in ca. 100 mL of diethyl ether and washed with ca. 50 mL of water, then dried over ca. 50 mL brine and $MgSO_4$. The supernatant was obtained via gravity filtration and concentrated to give the terminal diyne **S5** (3.27 g, 20.4 mmol, 73%) as a gold crystalline solid. This material was sufficiently pure for use in the following reaction.

Data for the terminal diyne S6:

^{1}H NMR ($CDCl_3$, 500 MHz): δ 7.44 (nfod, J_{app} = 8.7 Hz, 2H, *H2* and *H6*), 7.31 (nfod, J_{app} = 8.7 Hz, 2H, *H3* and *H5*), and 2.50 (s, 1H, C≡C-*H*).

^{13}C{^{1}H} NMR ($CDCl_3$, 126 MHz): δ 135.9, 134.1, 129.0, 119.7, 77.4, 74.6, 74.2, and 72.0.

HRMS (ESI-TOF) m/z: $[M+H^+]^+$ Calcd for $C_{10}H_6{}^{35}Cl^+$ 161.0153; Found 161.0149.

IR (neat): 3272, 2202, 1587, 1469, 1264, 1090, 1013, 823, 736, 626, and 523 cm^{-1}.

mp: 95–97 °C (with decomposition).

1-(Buta-1,3-diyn-1-yl)-3,5-dimethoxybenzene (S8)

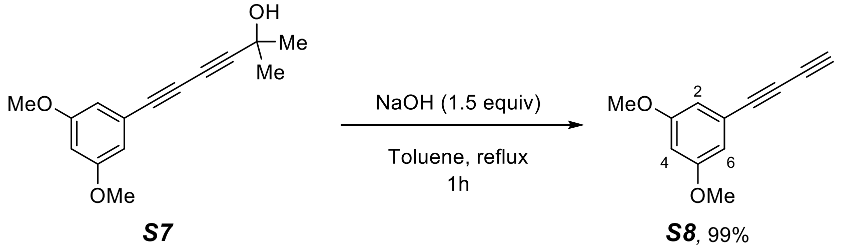

6-(3,5-Dimethoxyphenyl)-2-methylhexa-3,5-diyn-2-ol (**S7**) was prepared according to a literature procedure;[8] A copy of the proton NMR spectrum of **S7** has been provided.

To a stirred solution of 6-(3,5-dimethoxyphenyl)-2-methylhexa-3,5-diyn-2-ol (**S7**, 100 mg, 0.41 mmol, 1 equiv) in toluene (5 mL) was added NaOH (25 mg, 0.61 mmol, 1.5 equiv); the resulting mixture was refluxed for 1 h. The solvent was removed *in vacuo* and the residue was purified by flash column chromatography (10:1 hexanes:EtOAc) to afford 1-(buta-1,3-diyn-1-yl)-3,5-dimethoxybenzene (**S8**, 75 mg, 0.403 mmol, 99%) as an off-white solid which became darker upon full concentration, presumably due to slow degradation of the terminal diyne at high concentration. The terminal diyne was used within a matter of hours after isolation but appear stable for several months at -10 °C, even when stored as a neat sample.

Data for the terminal diyne S8:

^{1}H NMR ($CDCl_3$, 500 MHz): δ 6.66 (d, *J* = 2.4 Hz, 2H, *H2* and *H6*), 6.49 (t, *J* = 2.4 Hz, 1H, *H4*), 3.77 (s, 6H, ArOCH_3), and 2.47 (s, 1H, C≡C-*H*).

^{13}C{^{1}H} NMR ($CDCl_3$, 126 MHz): δ 160.7, 122.3, 110.6, 103.4, 75.5, 73.1, 71.4, 68.2, and 55.6.

HRMS (ESI-TOF) *m/z*: $[M+H^+]^+$ Calcd for $C_{12}H_{11}O_2^+$ 187.0754; Found 187.0745.

IR (neat): 3281, 3093, 3002, 2965, 2838, 2205, 1589, 1448, 1418, 1355, 1304, 1201, 1158, 1065, 1018, 989, 928, 850, 820, 672, 646, 634, 603, and 535 cm^{-1}.

mp: 49–50 °C.

2-Methyl-6-(pyridin-3-yl)hexa-3,5-diyn-2-ol (S11)

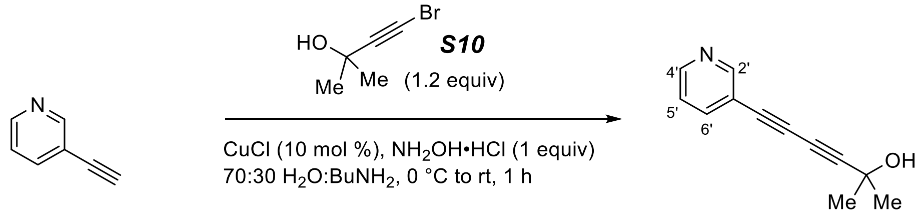

S9 ***S11***, 50%

3-Ethynylpyridine (**S9**) was purchased from Ambeed. 4-Bromo-2-methylbut-3-yn-2-ol (**S10**) was prepared according to a literature procedure;[9] a copy of the proton NMR spectrum of **S10** has been provided.

To a 250 mL round-bottom flask, copper (I) chloride (370 mg, 0.374 mmol, 0.1 equiv), hydroxylamine hydrochloride (259 mg, 37.3 mmol, 1.00 equiv), and a magnetic stir bar were added. The reaction solids were dissolved in 100 mL of 70/30 *n*-butylamine/water solution, placed in an ice bath, and stirred under nitrogen. 3-Ethynylpyridine (**S9,** 3.85 g, 37.3 mmol, 1 equiv) and 4-bromo-2-methylbut-3-yn-2-ol (**S10**, 7.30 g, 44.8 mmol, 1.2 equiv) were taken up in 50 mL of DCM and placed in an addition funnel over the reaction flask. The solution of alkynes was added dropwise over ca. 15 min. The presence of a bright yellow precipitate indicated copper acetylide formation. After addition of the solution of alkynes, the reaction was allowed to come to room temperature. After 1 h, the reaction was determined to be complete by TLC and GC-MS analysis. The reaction was quenched with 50 mL of saturated aqueous NH_4Cl and washed twice more until dissipation of any blue coloration of the aqueous layer, indicative of residual copper (II). The organic layer was then dried with brine (50 mL) and $MgSO_4$. The organic layer was then evaporated and the crude material purified by flash chromatography (1:1 Hex:EtOAc) to afford 2-methyl-6-(pyridin-3-yl)hexa-3,5-diyn-2-ol (**S11**, 3.49 g, 18.8 mmol, 50%) as a yellow crystalline solid.

Data for the diynol S11:

^{1}H NMR ($CDCl_3$, 500 MHz): δ 8.77 (dd, J = 2.2, 0.8 Hz, 1H, *H2'*), 8.56 (dd, J = 4.9, 1.8 Hz, 1H, *H6'*), 7.77 (ddd, J = 8.0, 2.2, 1.7 Hz, 1H, *H4'*), 7.28 (ddd, J = 8.0, 4.9, 0.9 Hz, 1H, *H5'*), 3.32 (br s, 1H, O*H*), and 1.58 (s, 6H, $(CH_3)_2$).

^{13}C{^{1}H} NMR ($CDCl_3$, 126 MHz): δ 153.2, 149.1, 139.7, 123.3, 119.4, 88.7, 76.7, 75.2, 66.5, 65.5, and 31.2.

HRMS (ESI-TOF) *m/z*: $[M+H^+]^+$ Calcd for $C_{12}H_{12}NO^+$ 186.0913; Found 186.0906.

IR (neat): 3246 (br), 2981, 2933, 1409, 1210, 1169, 955, 805, and 701 cm^{-1}.

mp: 97–99 °C.

5-(4-Methoxyphenyl)penta-2,4-diynoic acid (10b)

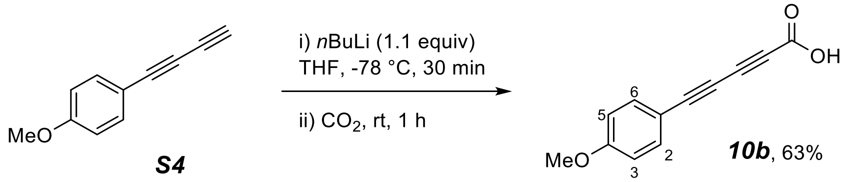

1-(Buta-1,3-diyn-1-yl)-4-methoxybenzene (448 mg, 2.87 mmol, 1 equiv) was prepared according to a literature procedure;[10] A copy of the proton NMR spectrum of **S4** has been provided here.

To a stirred solution of diyne **S4** in THF at -78 °C, *n*-BuLi (2.5 M, 1.37 mL, 3.44 mmol, 1.2 equiv) in hexane was added dropwise under N_2 atmosphere. After addition, stirring was continued for 30 minutes. The dry ice bath was removed and subsequently after, CO_2 was bubbled into the reaction mixture. The stirring was continued for an additional 1 h at room temperature. The reaction mixture was quenched with aqueous HCl (1M) and extracted with EtOAc. The combined organic extracts were washed with water and brine, dried over $MgSO_4$ and concentrated under reduced pressure. The crude material was purified by flash column chromatography (1:1 hexanes:EtOAc) to afford the carboxylic acid **10b** (362 mg, 1.8 mmol, 63%) as a red, crystalline solid.

Data for the carboxylic acid diyne 10b:

^{1}H NMR (DMSO-d_6, 500 MHz): δ 7.63 (nfod, J_{app} = 9.0 Hz, 2H, *H2* and *H6*), 7.02 (nfod, J_{app} = 9.0 Hz, 2H, *H3* and *H5*), ~5.1–2.6 (br s, 1H, -CO_2H), and 3.81 (s, 3H, -OCH_3).

^{13}C{^{1}H} NMR (DMSO-d_6, 126 MHz): δ 161.3, 153.3, 135.1, 114.8, 110.4, 84.1, 73.3, 70.6, 69.3, and 55.5.

HRMS (ESI-TOF) *m/z*: [M+H$^+$]$^+$ Calcd for $C_{12}H_9O_3^+$ 201.0546; Found 201.0541.

IR (neat): 3600–2500 (br), 3459, 2955, 2930, 2861, 2200, 1674, 1661, 1596, 1327, 1246, 831, and 534.

mp: 146–150 °C (with gas evolution, presumable loss of CO_2, and subsequent decomposition).

Note: Decarboxylation is also observed of an NMR sample in DMSO (ca. 50% overnight) but not of a sample in $CDCl_3$).

5-(4-Chlorophenyl)penta-2,4-diynoic acid (10c)

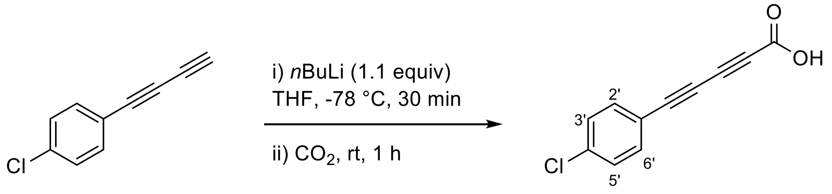

S6 ***10c***, 72%

To a flame-dried 250 mL round-bottom flask charged with a magnetic stir bar was added 1-(buta-1,3-diyn-1-yl)-4-chlorobenzene (**S6**, 2.00 g, 12.5 mmol, 1 equiv). The flask was evacuated and backfilled with dry nitrogen three times. Dry tetrahydrofuran (100 mL) was added and the solution was cooled to -78 °C. To the cooled reaction solution, *n*-butyllithium (2.5 M, 5.5 mL, 14 mmol, 1.1 equiv) was added dropwise over the course of 15 min. The reaction solution was allowed to stir for an additional 15 min at -78 °C, during which time, a gray suspension formed. The vessel was removed from the dry ice bath and allowed to come to room temperature as carbon dioxide was allowed to bubble through the solution (see Figure S1 for apparatus setup). The suspension had then become a gold-colored solution. Bubbling was allowed to continue for one hour at ambient conditions. Upon reaction completion as determined by TLC, an equal volume of 5 M aqueous HCl was added and the resulting mixture was extracted three times with 50 mL portions of ethyl acetate. The combined organic layers were dried with brine and $MgSO_4$ and evaporated. The crude material was purified by flash chromatography. Because of poor solubility in the eluent (3:1 hexanes:EtOAc) and streaking on silica, higher rf impurities were eluted first followed by elution with pure EtOAc to afford the carboxylic acid diyne **10c** as an off-white crystalline solid (1.83 g, 8.94 mmol, 72%).

Data for the carboxylic acid diyne 10c:

^{1}H NMR (DMSO-d_6, 500 MHz): δ 14.25 (s, 1H, CO_2H), 7.71 (nfod, J_{app} = 8.6 Hz, 2H, *H2′* and *H6′*), and 7.55 (nfod, J_{app} = 8.6 Hz, 2H, *H3′* and *H5′*).

^{13}C{^{1}H} NMR (DMSO-d_6, 126 MHz): δ 153.1, 136.0, 134.8, 129.3, 117.8, 82.0, 74.0, 72.4, and 68.3.

HRMS (ESI-TOF) *m/z*: [M+H$^+$]$^+$ Calcd for $C_{11}H_6{}^{35}ClO_2{}^+$ 205.0051; Found 205.0041.

IR (neat): 3600–2500 (br), 2232, 1667, 1433, 1089, and 826 cm^{-1}.

mp: 148–150 °C (with gas evolution and subsequent decomposition).]

5-(3,5-Dimethoxyphenyl)penta-2,4-diynoic acid (10d)

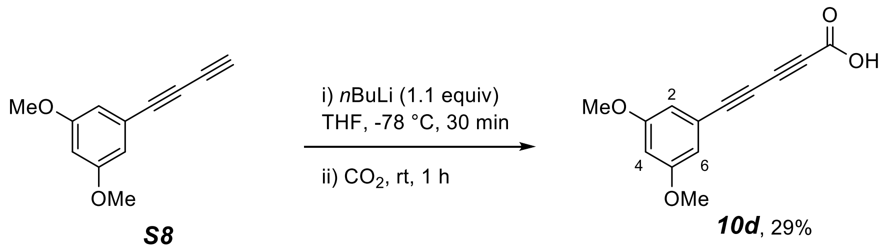

To a flame-dried 250 mL round-bottom flask charged with a magnetic stir bar was added 1-(buta-1,3-diyn-1-yl)-3,5-dimethoxybenzene (**S8**, 4.57 g, 24.6 mmol, 1 equiv). The flask was evacuated and backfilled with nitrogen three times. Dry tetrahydrofuran (100 mL) was added and the solution was cooled to -78 °C. To the cooled solution, *n*-butyllithium (2.5 M, 10.8 mL, 27.1 mmol, 1.1 equiv) was added dropwise over 15 min and stirring was continued for 30 min at -78 °C. The vessel was removed from the dry ice bath and allowed to warm to room temperature as CO_2 was bubbled into the solution. Bubbling was continued for an additional 1 h at room temperature. The reaction was quenched by the addition of aqueous HCl (5M) and extracted three times with 50 mL portions of ethyl acetate. The combined organic extracts were washed with water and brine, dried over $MgSO_4$, and concentrated under reduced pressure. The crude mixture was purified by flash chromatography (3:1 hexanes:EtOAc) to afford the diynyl carboxylic acid **10d** as an orange crystalline solid (1.64 g, 7.13 mmol, 29%).

Data for the carboxylic acid diyne 10d:

^{1}H NMR (DMSO-d_6, 500 MHz): δ 14.34 (br s, 1H, -CO$_2$*H*), 6.88 (d, *J* = 2.3 Hz, 2H, *H2* and *H6*), 6.72 (t, *J* = 2.3 Hz, 1H, *H4*), and 3.80 (s, 6H, -OC*H$_3$*).

^{13}C{^{1}H} NMR (DMSO-d_6, 126 MHz): δ 160.5, 153.1, 120.2, 110.6, 104.3, 83.2, 73.5, 71.0, 68.4, and 55.6.

HRMS (ESI-TOF) *m/z*: $[M+H^+]^+$ Calcd for $C_{13}H_{11}O_4^+$ 231.0652; Found 231.0639.

IR (neat): 3600–2500 (br), 3490, 3087, 3066, 2998, 2936, 2870, 2840, 2215, 2147, 1671, 1589, 1450, 1412, 1361, 1283, 1203, 1156, 1043, and 827 cm^{-1}.

mp: 88–92 °C (gas evolution with subsequent decomposition).

5-(Pyridin-3-yl)penta-2,4-diynoic acid (10e)

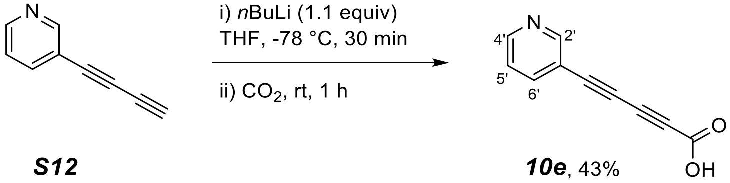

3-(Buta-1,3-diyn-1-yl)pyridine (**S12**, 850 mg, 6.67 mmol, 1 equiv) was prepared according to a literature procedure;[11] A copy of the proton NMR spectrum of **S12** has been provided.

To a flame-dried 250 mL round-bottom flask charged with a magnetic stir bar was added 3-(buta-1,3-diyn-1-yl)pyridine. The flask was evacuated and backfilled with dry nitrogen three times. Dry tetrahydrofuran (100 mL) was added and the solution was cooled to -78 °C. To the cooled reaction solution, *n*-butyllithium (2.5 M, 3.0 mL, 7.5 mmol, 1.1 equiv) was added dropwise over the course of 15 min. The reaction solution was allowed to stir for a further 15 min at -78 °C, during which time, the solution became black. The vessel was removed from the dry ice bath and allowed to come to room temperature as carbon dioxide was allowed to bubble through (see Figure S1 for apparatus setup). The solution then became a gold-colored suspension. Bubbling was allowed to continue for one hour at ambient conditions. Upon reaction completion as determined by TLC, an equal volume of deionized water was added, solubilizing the reaction solids. Approximately 5 mL of 5M aqueous HCl was added and the neutral pyridyl carboxylic acid was extracted three times into 50 mL portions of diethyl ether. The combined organic layers were dried with brine and $MgSO_4$ and evaporated. The crude material was not amenable to silica gel chromatography due to low solubility, even in polar organic solvents. Therefore, the product was recrystallized from hot EtOAc:hexanes, and 5-(pyridin-3-yl)penta-2,4-diynoic acid (**10e**, 490 mg, 2.86 mmol, 43%) was isolated via vacuum filtration as a brown crystalline solid.

Data for the carboxylic acid diyne 10e:

^{1}H NMR (DMSO-d_6, 500 MHz): δ 12.35 (br s, 1H, CO_2*H*), 8.86 (dd, *J* = 2.2, 0.8 Hz, 1H, *H2'*), 8.69 (dd, *J* = 4.9, 1.7 Hz, 1H, *H6'*), 8.11 (ddd, *J* = 7.9, 2.2, 1.6 Hz, 1H, *H4'*), and 7.51 (ddd, *J* = 7.9, 4.9, 0.9 Hz, 1H, *H5'*).

^{13}C{^{1}H} NMR (DMSO-d_6, 126 MHz): δ 153.2, 153.0, 150.7, 140.4, 123.7, 116.5, 80.0, 74.5, 74.3, and 67.8.

HRMS (ESI-TOF) *m/z*: $[M+H^+]^+$ Calcd for $C_{10}H_6NO_2^+$ 172.0393; Found 172.0386.

IR (neat): 3600–2500 (br), 2233, 1692, 1332, 1243, 1052, 1041, 810, 749, 691, 649, 571, and 511 cm^{-1}.

mp: 97–99 °C (gas evolution with subsequent decomposition).

Hexa-2,4-diynoic acid (10f)

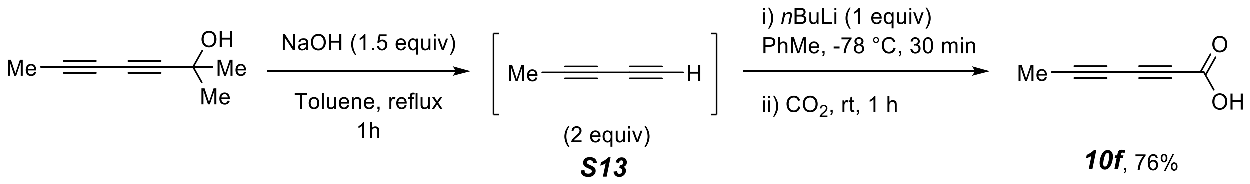

From an earlier report from our laboratory:

"**Caution**: low molecular weight, terminal 1,3-diynes have been reported to detonate; hence we developed the following procedure to avoid handling of neat 1,3-pentadiyne. 2-Methylhepta-3,5-diyn-2-ol (9.76 g, 80 mmol), sodium hydroxide (4.80 g, 120 mmol), and toluene (350 mL, 0.23 M) were placed into a 500 mL round-bottom flask equipped with a stir bar and heated to reflux for 1 hour. The solution was cooled, washed with water (100 mL x 2) and brine (100 mL), and dried over $MgSO_4$. This solution was passed through a plug of silica gel (to remove acetone-derived dimers) with additional toluene as eluant to give a solution of 1,3-pentadiyne in toluene (~ 500 mL total volume)."[12]

In this study:

An oven dried 100 mL round-bottom flask fitted with a stir bar was evacuated and backfilled with N_2 three times. To the flask was added a freshly prepared toluene solution of 1,3-pentadiyne (**S13**, 0.2 M, 15 mmol of the diyne in ca. 75 mL of PhMe, 2 equiv) and the solution was cooled to -78 °C. *n*-BuLi (2.5 M, 3.0 mL, 7.4 mmol, 1 equiv) in hexane was added dropwise under N_2 atmosphere. After addition, stirring was continued for 30 minutes. The dry ice bath was removed and subsequently after, CO_2 was bubbled into the reaction mixture. The stirring was continued for an additional 1 h at room temperature. The reaction was quenched by the addition of aqueous HCl (5M) and extracted three times with 50 mL portions of ethyl acetate. The combined organic extracts were washed with water and brine and dried over $MgSO_4$. The majority of the solvent was evaporated under reduced pressure and the product crystallized out of solution over 30 minutes. The crystalline solid was washed with toluene, decanted and collected via vacuum filtration. The mother liquors were combined for a second recrystallization to afford carboxylic acid **10f** (600 mg, 5.6 mmol, 76%) as a white, crystalline solid.

Data for the carboxylic acid diyne 10f:

^{1}H NMR (DMSO-d_6, 500 MHz): δ 14.08 (br s, 1H, -CO_2*H*), and 2.09 (s, 3H).

^{13}C{^{1}H} NMR (DMSO-d_6, 126 MHz): δ 154.1, 86.0, 70.7, 66.9, 62.9, and 5.0.

HRMS (EI) *m/z*: $[M\cdot]^+$ Calcd for $C_6H_4O_2^{\bullet+}$ 108.0211; Found 108.0203.

IR (neat): 3200–2400 (br), 3043, 2834, 2567, 2235, 1668, 1417, 1285, 1192, and 906.

mp: 108–110 °C (gas evolution with subsequent decomposition).

5-(Trimethylsilyl)penta-2,4-diynoic acid (10g)

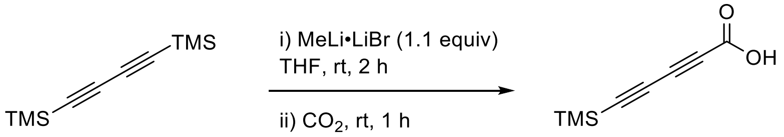

S14 ***10g***, 48%

1,4-bis(Trimethylsilyl)buta-1,3-diyne (**S14**, 108 mg, 0.55 mmol, 1 equiv) was prepared according to a literature procedure and its proton NMR spectrum has been provided.[13]

To a flame-dried 25 mL round-bottom flask charged with a magnetic stir bar was added 1,4-bis(trimethylsilyl)buta-1,3-diyne. The flask was evacuated and backfilled with dry nitrogen three times. Dry tetrahydrofuran (10 mL) was added and the solution was stirred at ambient temperature. To the cooled reaction solution, methyllithium lithium bromide complex (1.5 M in Et_2O, 0.4 mL, 0.6 mmol, 1.1 equiv) was added dropwise over the course of 5 min. The reaction solution was allowed to stir for 2 h at ambient temperature. Subsequently, CO_2 was bubbled directly into the solution (cf. Figure S1), which immediately turned dark brown. Bubbling was allowed to continue for one hour at ambient conditions. Upon reaction completion as determined by TLC, an equal volume of 5 M aqueous HCl was added and the mixture was extracted three times with 15 mL portions of ethyl acetate. The combined organic layers were dried with brine and $MgSO_4$ and the solvent was evaporated. The crude material was purified by flash chromatography (4:1 hexanes:EtOAc) to afford the carboxylic acid diyne **10g** as an amber oil (44 mg, 0.27 mmol, 48%).

Data for the diyne carboxylic acid 10g:

^{1}H NMR ($CDCl_3$, 500 MHz): δ 9.17 (br s, 1H, CO_2H) and 0.24 [(s, 9H, $Si(CH_3)_3$].

^{13}C{^{1}H} NMR ($CDCl_3$, 126 MHz): δ 156.4, 96.2, 85.5, 73.3, 66.1, and 0.69.

HRMS (EI) *m/z*: $[M\text{-}CH_3]^+$ Calcd for $C_7H_7O_2Si^+$ 151.0215; Found 151.0208.

IR (neat): 3600–2500 (br), 2960, 2925, 2856, 2203, 2106, 1688, 1409, 1375, 1250, 1112, 842, 760, 637, 595, and 558.

5-(Triisopropylsilyl)penta-2,4-diynoic acid (10h)

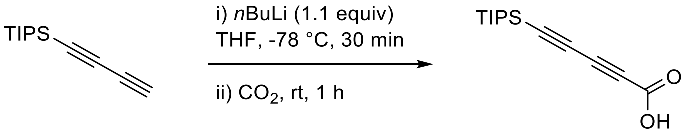

S15 ***10h***, 84%

Buta-1,3-diyn-1-yltriisopropylsilane (**S15**, 2.00 g, 9.69 mmol, 1 equiv) was prepared according to a literature procedure and its proton NMR spectrum has been provided.[14]

To a flame-dried 250 mL round-bottom flask charged with a magnetic stir bar was added buta-1,3-diyn-1-yltriisopropylsilane. The flask was evacuated and backfilled with dry nitrogen three times. Dry tetrahydrofuran (100 mL) was added and the solution was cooled to -78 °C. To the cooled reaction solution, *n*-butyllithium (2.5 M, 4.3 mL, 11 mmol, 1.1 equiv) was added dropwise over the course of 15 min. The reaction solution was allowed to stir for a further 15 min at -78 °C. The vessel was removed from the dry ice bath and allowed to come to room temperature as carbon dioxide was allowed to bubble directly into the reaction solution (see Figure S1 for apparatus setup). Bubbling was allowed to continue for one hour at ambient conditions. Upon reaction completion as determined by TLC, an equal volume of 5 M aqueous HCl was added and the mixture was extracted three times with 50 mL portions of ethyl acetate. The combined organic layers were dried with brine and $MgSO_4$ and the solvent was evaporated. The crude material was purified by flash chromatography (3:1 hexanes:EtOAc to pure EtOAc) to afford the carboxylic acid diyne **10h** as an amber oil (2.05 g, 8.19 mmol, 84%).

Data for the carboxylic acid diyne 10h:

^{1}H NMR ($CDCl_3$, 500 MHz): δ 6.56 (br s, 1H, CO_2H) and 1.11–1.08 (overlapped m, 21H, $SiCH(CH_3)_2$ and $SiCH(CH_3)_2$).

^{13}C{^{1}H} NMR ($CDCl_3$, 126 MHz): δ 155.9, 94.1, 87.3, 73.6, 65.2, 18.6, and 11.3.

HRMS (ESI-TOF) *m/z*: $[M+H^+]^+$ Calcd for $C_{14}H_{23}O_2Si^+$ 251.1462; Found 251.1456.

IR (neat): 3600–2500 (br), 2892, 2867, 2203, 2104, 1463, 1273, 1242, 1117, 997, 882, 774, 679, and 662 cm^{-1}.

6-(4-Chlorobutoxy)-5-phenyl-4-(phenylethynyl)isobenzofuran-1,3-dione (11)

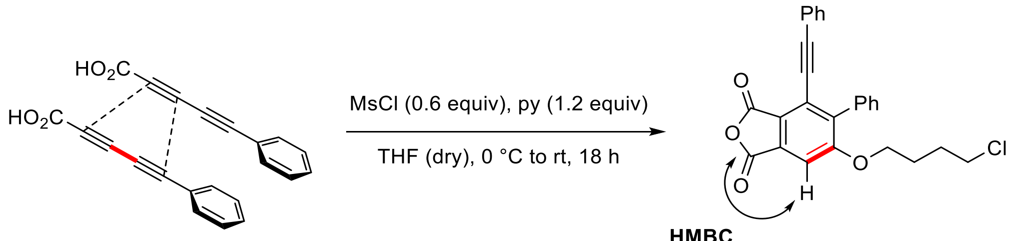

10a **11**, 67%

Following general procedure A, 5-phenylpenta-2,4-diynoic acid (**10a**, 50 mg, 0.29 mmol, 1 equiv), methanesulfonyl chloride (14 µL, 0.18 mmol, 0.6 equiv), pyridine (28 µL, 0.35 mmol, 1.2 equiv), and dry tetrahydrofuran (5.8 mL) were used to prepare the phthalic anhydride derivative **11**. Purification by MPLC (3:1 hexanes:EtOAc) yielded the phthalic anhydride derivative **11** (42 mg, 0.098 mmol, 67%) as a white crystalline solid.

Data for the phthalic anhydride derivative 11:

^{1}H NMR ($CDCl_3$, 500 MHz): δ 7.53–7.45 (overlapped m, 3H, ArPhH_m and ArPhH_p), 7.42 (s, 1H, ArH), 7.42–7.40 (nfom, 2H, ArPhH_o), 7.36–7.27 (overlapped m, 5H, C≡C-PhH_o, C≡C-PhH_m, and C≡C-PhH_p), 4.15 (t, J = 6.0 Hz, 2H, OCH_2), 3.43 (t, J = 6.4 Hz, 2H, ClCH_2), 1.89 (tt, J = 7.8, 6.0 Hz, 2H, OCH_2CH_2), and 1.77 (tt, J = 7.8, 6.4 Hz, 2H, $ClCH_2$CH_2).

^{13}C{^{1}H} NMR ($CDCl_3$, 126 MHz): δ 162.9, 162.5, 161.1, 141.5, 134.2, 133.2, 132.3, 130.1, 129.7, 128.7, 128.5, 128.1, 123.7, 122.7, 122.1, 106.9, 102.2, 83.3, 69.1, 44.4, 29.1, and 26.2.

HRMS (ESI-TOF) *m/z*: $[M+H^+]^+$ Calcd for $C_{26}H_{20}{}^{35}ClO_4^+$ 431.1045; Found 431.1034.

IR (neat): 3084, 3060, 2956, 2940, 2881, 2852, 2217, 1840, 1775, 1330, 729, and 692 cm^{-1}.

mp: 178–180 °C.

(±)-5-Phenyl-4-(phenylethynyl)-6,9-dihydro-6,9-epoxynaphtho[1,2-*c*]furan-1,3-dione (14a)

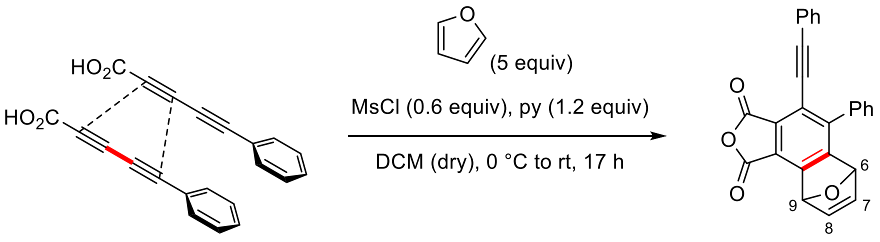

Following general procedure A, 5-phenylpenta-2,4-diynoic acid (**10a**, 50 mg, 0.29 mmol, 1 equiv), methanesulfonyl chloride (14 µL, 0.18 mmol, 0.6 equiv), pyridine (28 µL, 0.35 mmol, 1.2 equiv), furan (100 mg, 1.47 mmol, 5 equiv), and dry dichloromethane (5.8 mL) were used to prepare the phthalic anhydride derivative **14a**. Purification by MPLC (3:1 hexanes:EtOAc) yielded **14a** (48 mg, 0.12 mmol, 84%) as a pale yellow crystalline solid.

Data for the phthalic anhydride derivative 14a:

^{1}H NMR ($CDCl_3$, 500 MHz): δ 7.60–7.53 (overlapped m, 3H, ArPhH_m and ArPhH_p), 7.47–7.43 (br m, 2H, ArPhH_o), 7.39–7.28 (overlapped m, 5H, C≡C-PhH_o, C≡C-PhH_m, and C≡C-PhH_p), 7.19 (d, J = 5.8 Hz, 1H, *H7* or *H8*), 7.18 (d, J = 5.6 Hz, 1H, *H7* or *H8*), 6.30 (s, 1H, *H9*), and 5.67 (s, 1H, *H6*).

^{13}C{^{1}H} NMR ($CDCl_3$, 126 MHz): δ 161.5, 161.2, 157.5, 148.7, 143.4, 142.7, 142.6, 135.3, 132.3, 129.8, 129.42 (2x), 128.7, 128.6, 126.9, 122.06, 122.04, 120.7, 102.4, 84.0, 81.9, and 80.7.

HRMS (ESI-TOF) *m/z*: $[M+H^+]^+$ Calcd for $C_{26}H_{15}O_4^+$ 391.0965; Found 391.0956.

IR (neat): 3059, 3028, 2957, 2924, 2215, 1839, 1766, 900, 873, and 732 cm^{-1}.

mp: 222–224 °C.

(±)-5-(4-Methoxyphenyl)-4-((4-methoxyphenyl)ethynyl)-6,9-dihydro-6,9-epoxynaphtho[1,2-*c*]furan-1,3-dione (14b)

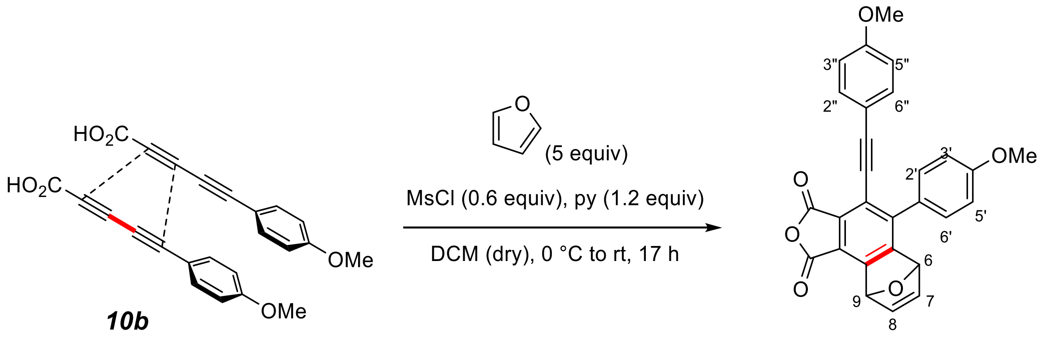

14b, 85%

Following general procedure A, 5-(4-methoxyphenyl)penta-2,4-diynoic acid (**10b**, 20 mg, 0.1 mmol, 1 equiv), methanesulfonyl chloride (4.5 µL, 0.06 mmol, 0.6 equiv), pyridine (10 µL, 0.12 mmol, 1.2 equiv), furan (36 µL, 0.5 mmol, 5 equiv), and dry dichloromethane (2 mL) were used to prepare the phthalic anhydride derivative **14b**. Purification by a silica plug (100% EtOAc) yielded **14b** (19 mg, 0.042 mmol, 85%) as an orange crystalline solid.

Data for the phthalic anhydride derivative 14b:

^{1}H NMR ($CDCl_3$, 500 MHz): δ 7.40 (br nfod, 2H, *H2′* and *H6′*), 7.38 (nfod, J_{app} = 8.8 Hz, 2H, *H2″* and *H6″*), 7.19 (d, 1H, J = 5.8, *H7* or *H8*) 7.17 (d, 1H, J = 5.8, *H7* or *H8*), 7.09 (nfod, J_{app} = 8.9 Hz, 2H, *H5′* and *H3′*), 6.85 (nfod, J_{app} = 8.9 Hz, 2H, *H3″* and *H5″*), 6.29 (dd, J = 2.2, 1.1 Hz, 1H, *H9*), 5.69 (dd, J = 2.0, 0.9 Hz, 1H, *H6*), 3.93 (s, 3H, C4'OCH_3), and 3.82 (s, 3H, C4″OCH_3).

^{13}C{^{1}H} NMR ($CDCl_3$, 126 MHz): δ 161.6, 161.4, 160.9, 160.5, 157.2, 148.0, 143.3, 142.7, 142.0, 134.0, 130.9, 127.6, 126.5, 121.7, 121.2, 114.3, 114.2, 114.1, 103.0, 83.5, 82.0, 80.6, 55.6, and 55.5.

HRMS (ESI-TOF) *m/z*: $[M+H^+]^+$ Calcd for $C_{28}H_{19}O_6^+$ 451.1176; Found 451.1165.

IR (neat): 2917, 2838, 2199, 1837, 1767, 1605, 1594, 1509, 1432, 1289, 1249, 1228, 1176, 1166, 1025, 834, 717, and 693 cm^{-1}.

mp: 215–218°C.

(±)-5-(4-Chlorophenyl)-4-((4-chlorophenyl)ethynyl)-6,9-dihydro-6,9-epoxynaphtho[1,2-*c*]furan-1,3-dione (14c)

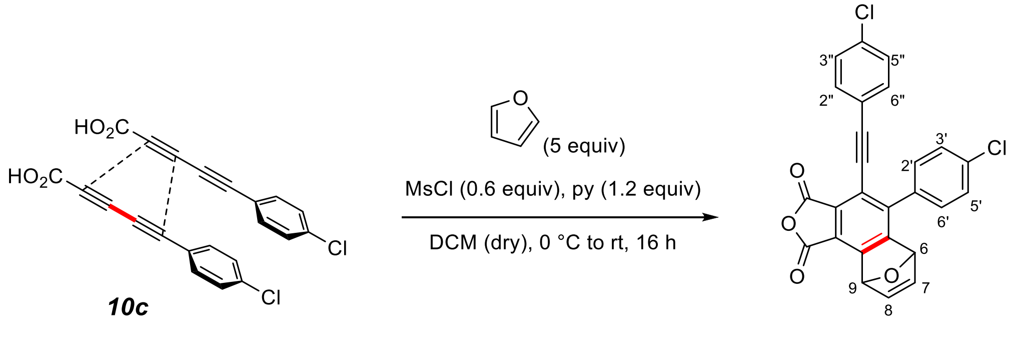

Following general procedure A, 5-(4-chlorophenyl)penta-2,4-diynoic acid (**10c**, 50 mg, 0.24 mmol, 1 equiv), methanesulfonyl chloride (11 µL, 0.14 mmol, 0.6 equiv), pyridine (23 µL, 0.29 mmol, 1.2 equiv), furan (83 mg, 1.22 mmol, 5 equiv), and dry dichloromethane (5.8 mL) were used to prepare the phthalic anhydride derivative **14c**. Purification by MPLC (3:1 hexanes:EtOAc) yielded the phthalic anhydride derivative **14c** (39 mg, 0.086 mmol, 70%) as a white crystalline solid.

Data for the phthalic anhydride derivative 14c:

^{1}H NMR ($CDCl_3$, 500 MHz): δ 7.56 (nfod, J_{app} = 8.8 Hz, 2H, *H3′* and *H5′*), 7.38 (br nfod, J_{app} = 8.0 Hz, 2H, *H2′* and *H6′*), 7.33 (nfod, J_{app} = 8.6 Hz, 2H, *H3″* and *H5″*), 7.31 (nfod, J_{app} = 8.6 Hz, 2H, *H2″* and *H6″*), 7.20 (dd, *J* = 5.5, 2.0 Hz, 1H, *H8*), 7.16 (dd, *J* = 5.5, 2.0 Hz, 1H, *H7*), 6.31 (dd, *J* = 1.9, 0.8 Hz, 1H, *H9*), and 5.66 (dd, *J* = 1.9, 0.8 Hz, 1H, *H6*).

^{13}C{^{1}H} NMR ($CDCl_3$, 126 MHz): δ 161.2, 161.0, 157.7, 149.0, 143.2, 142.9, 141.1, 136.2, 135.8, 133.6, 133.4, 130.8, 129.09, 129.08, 127.2, 122.3, 120.3, 120.1, 101.4, 84.5, 81.8, and 80.7.

HRMS (ESI-TOF) *m/z*: $[M+H^+]^+$ Calcd for $C_{26}H_{13}{}^{35}Cl_2O_4^+$ 459.0185; Found 459.0180.

IR (neat): 3088, 3055, 3030, 2954, 2930, 2857, 2215, 1839, 1774, 1738 (sh), 1490, 1228, 1167, 1090, 901, 872, and 830 cm^{-1}.

mp: 240–242 °C.

1 mmol scale reaction

To demonstrate the scalability of the synthetic method, the above reaction was also performed on an ca. 1 mmol scale. Following general procedure A, 5-(4-chlorophenyl)penta-2,4-diynoic acid (**10c**, 205 mg, 1.00 mmol, 1 equiv), methanesulfonyl chloride (46.5 µL, 0.601 mmol, 0.6 equiv), pyridine (101 µL, 1.25 mmol, 1.25 equiv), furan (341 mg, 5.01 mmol, 5 equiv), and dry dichloromethane (20.0 mL) were used to prepare the phthalic anhydride derivative **14c**.

Purification by MPLC (3:1 hexanes:EtOAc) yielded the phthalic anhydride derivative **14c** (199 mg, 0.433 mmol, 87%) as an off-white crystalline solid that matched the spectral and physical properties given above.

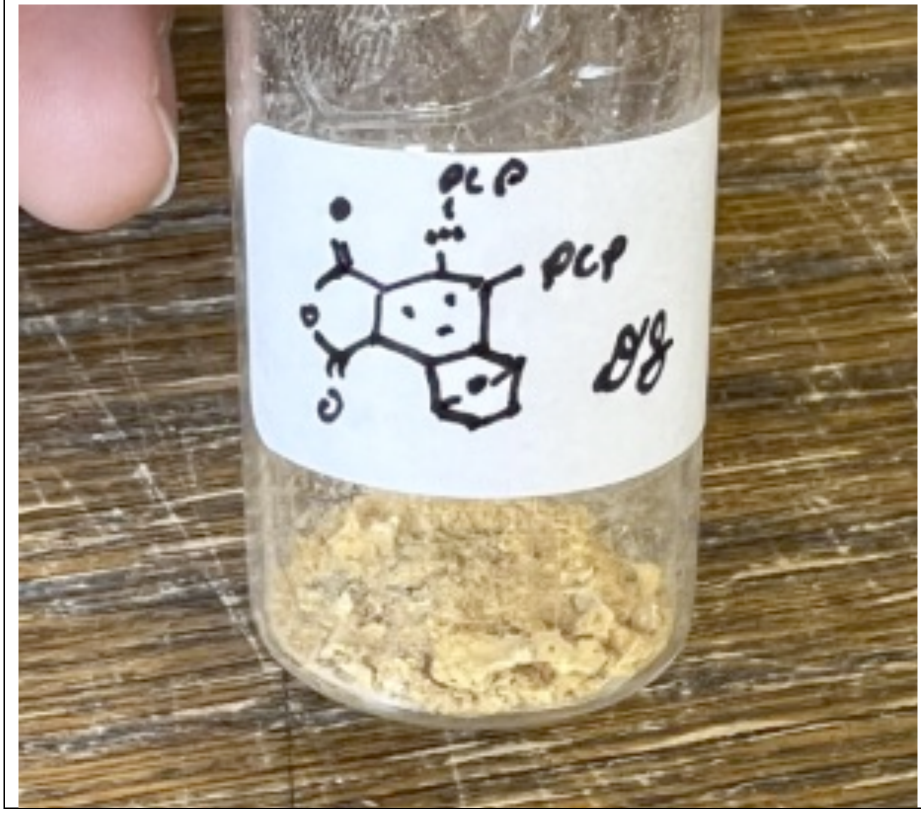	The solid sample of **14c** obtained from reaction scale-up.<br><br>(Photograph taken by Dorian Sneddon, one of the authors of this manuscript.)

(±)-5-(3,5-Dimethoxyphenyl)-4-((3,5-dimethoxyphenyl)ethynyl)-6,9-dihydro-6,9-epoxynaphtho[1,2-*c*]furan-1,3-dione (14d)

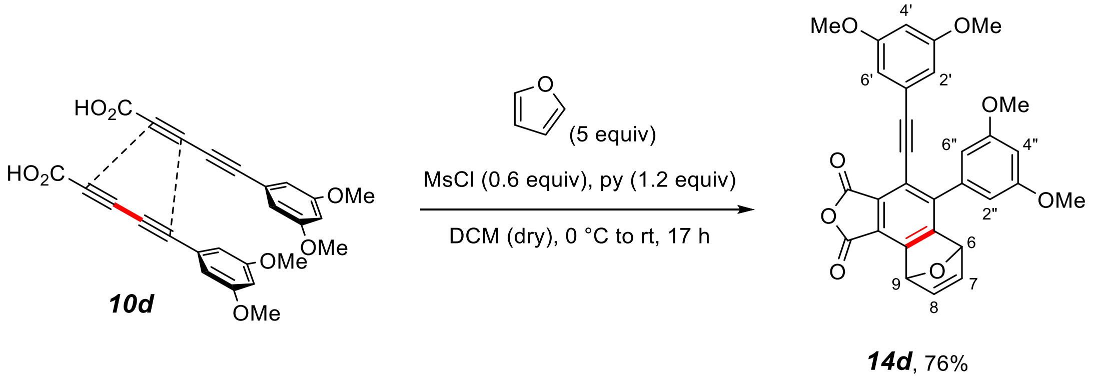

Following general procedure A, 5-(3,5-dimethoxyphenyl)penta-2,4-diynoic acid (**10d**, 50 mg, 0.22 mmol, 1 equiv), methanesulfonyl chloride (10 µL, 0.13 mmol, 0.6 equiv), pyridine (21 µL, 0.26 mmol, 1.2 equiv), furan (80 µL, 1.1 mmol, 5 equiv), and dry dichloromethane (4 mL) were used to prepare the phthalic anhydride derivative **14d**. Purification by MPLC (3:7 hexanes:EtOAc) yielded **14d** (43 mg, 0.082 mmol, 76%) as an orange crystalline solid.

Data for the phthalic anhydride derivative 14d:

^{1}H NMR ($CDCl_3$, 500 MHz): δ 7.18 (d, *J* = 5.3 Hz, 1H, *H7* or *H8*), 7.17 (d, *J* = 5.3 Hz, 1H, *H7* or *H8*) 6.61 (t, *J* = 2.2 Hz, 1H, *H4'*), 6.59–6.51 (br m, 2H, *H2'', H6''*), 6.55 (d, *J* = 2.2 Hz, 2H, *H2', H6'*), 6.47 (t, *J* = 2.2 Hz, 1H, *H4''*), 6.29 (nfom, 1H, *H9*), 5.74 (nfom, 1H, *H6*), 3.84 (s, 6H, C3'OCH_3 and C5'OCH_3), and 3.77 (s, 6H, C3''OCH_3 and C5''OCH_3).

^{13}C{^{1}H} NMR ($CDCl_3$, 126 MHz): δ 161.4, 161.1, 160.9, 160.7, 157.5, 148.7, 143.3, 142.7, 142.6, 137.0, 126.8, 123.3, 122.1, 120.4, 109.8, 107.6, 103.6, 102.6, 101.1, 83.5, 82.0, 80.6, 55.7, and 55.6.

HRMS (ESI-TOF) *m/z*: $[M+H^+]^+$ Calcd for $C_{30}H_{23}O_8^+$ 511.1387; Found 511.1363.

IR (neat): 3089, 3024, 2972, 2941, 2841, 2212, 1837, 1776, 1584, 1453, 1417, 1352, 1303, 1269, 1204, 1155, 1058, 856, and 712 cm^{-1}.

mp: 196–200 °C.

(±)-5-(Pyridin-3-yl)-4-(pyridin-3-ylethynyl)-6,9-dihydro-6,9-epoxynaphtho[1,2-*c*]furan-1,3-dione (14e)

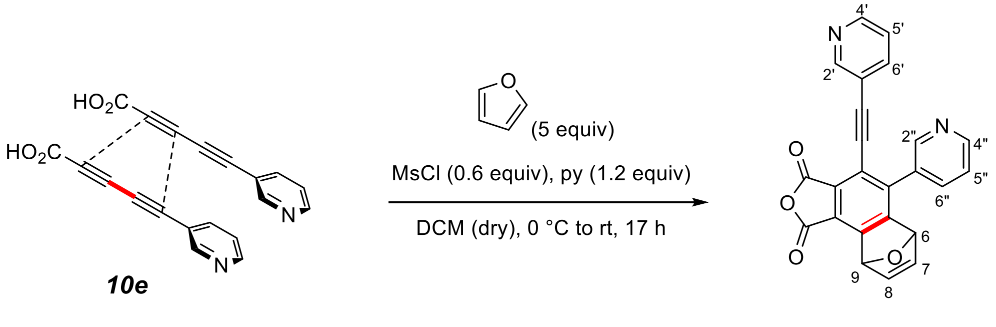

Following general procedure A, 5-(pyridin-3-yl)penta-2,4-diynoic acid (**10e**, 50 mg, 0.29 mmol, 1 equiv), methanesulfonyl chloride (14 µL, 0.18 mmol, 0.6 equiv), pyridine (29 µL, 0.37 mmol, 1.2 equiv), furan (99 mg, 1.45 mmol, 5 equiv), and dry dichloromethane (5.8 mL) were used to prepare the phthalic anhydride derivative **14e**. Purification by MPLC (1:1 hexanes:EtOAc) yielded **14e** (52 mg, 0.13 mmol, 91%) as a pale yellow crystalline solid.

Data for the phthalic anhydride derivative 14e:

^{1}H NMR ($CDCl_3$, 500 MHz): δ 8.82 (dd, *J* = 4.8, 1.4 Hz, 1H, *H4″*), 8.67 (br s, 1H, *H2″*), 8.59 (dd, *J* = 4.9, 1.6, 0.6 Hz, 1H, *H4′*), 8.57 (dd, *J* = 2.0, 0.6 Hz, 1H, *H2′*), 7.89 (br d, *J* = 7.8 Hz, 1H, *H6″*), 7.74 (ddd, *J* = 7.9, 2.0, 1.6 Hz, 1H, *H6′*), 7.56 (ddd, *J* = 7.9, 4.9, 0.7 Hz, 1H, *H5″*), 7.29 (dd, *J* = 7.9, 4.9, 0.7 Hz, *H5′*), 7.23 (dd, *J* = 5.5, 1.9 Hz, 1H, *H8*), 7.20 (dd, *J* = 5.5, 1.9 Hz, 1H, *H7*), 6.34 (dd, *J* = 1.9, 0.9 Hz, 1H, *H9*), and 5.70 (dd, *J* = 1.9, 0.9 Hz, 1H, *H6*).

^{13}C{^{1}H} NMR ($CDCl_3$, 126 MHz): δ 161.0, 160.8, 158.2, 152.6, 150.8, 150.1, 149.7, 143.3, 142.9, 139.2, 138.7, 136.9, 131.2, 127.6, 123.50, 123.47, 123.3, 122.7, 119.8, 118.9, 99.1, 86.2, 81.7, and 80.7.

HRMS (ESI-TOF) *m/z*: $[M+H^+]^+$ Calcd for $C_{24}H_{13}N_2O_4^+$ 393.0870; Found 393.0860.

IR (neat): 3359 (br), 3224 (br), 2962, 2925, 2855, 2210, 1842, 1773, 1478, 1231, 1187, 1171, 1025, 903, 873, 818, 740, 712, and 640 cm^{-1}.

mp: >400 °C (decomposes prior to melting).

(±)-5-Methyl-4-(prop-1-yn-1-yl)-6,9-dihydro-6,9-epoxynaphtho[1,2-*c*]furan-1,3-dione (14f)

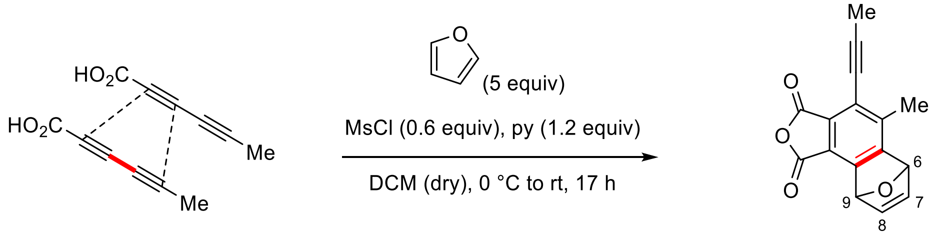

10f ***14f***, 91%

Following general procedure A, hexa-2,4-diynoic acid (**10f**, 20 mg, 0.19 mmol, 1 equiv), methanesulfonyl chloride (8.5 µL, 0.11 mmol, 0.6 equiv), pyridine (19 µL, 0.23 mmol, 1.2 equiv), furan (67 µL, 0.93 mmol, 5 equiv), and dry dichloromethane (4 mL) were used to prepare the phthalic anhydride derivative **14f**. Purification by a silica plug (100% EtOAc) yielded **14f** (22 mg, 0.083 mmol, 91%) as a white crystalline solid.

Data for the phthalic anhydride derivative 14f:

^{1}H NMR ($CDCl_3$, 500 MHz): δ 7.11 (dd, 1H, J = 5.1, 1.5 Hz, *H7* or *H8*), 7.10 (dd, 1H, J = 5.1, 1.4 Hz, *H7* or *H8*), 6.23 (dd, J = 2.2, 1.0 Hz, 1H, *H9*), 5.90 (dd, J = 2.2, 1.0 Hz, 1H, *H6*), 2.51 (s, 3H, ArCH_3), and 2.24 (s, 3H, C≡C-CH_3).

^{13}C{^{1}H} NMR ($CDCl_3$, 126 MHz): δ 161.7, 161.5, 157.1, 147.7, 142.9, 142.8, 139.7, 126.6, 122.8, 121.0, 101.5, 81.0, 80.6, 73.7, 17.3, and 5.3.

HRMS (ESI-TOF) *m/z*: Calculated for $C_{16}H_{10}O_4^+$ [M+H$^+$] 267.0652, found 267.0642.

IR (neat): 3104, 3019, 2959, 2916, 2232, 1836, 1771, 1438, 1378, 1283, 1270, 1249, 1226, 1180, 1093, 1034, 988, 891, 876, 861, 836, 748, 713, and 628.

mp: 218–220 °C.

(±)-5-(Trimethylsilyl)-4-((trimethylsilyl)ethynyl)-6,9-dihydro-6,9-epoxynaphtho[1,2-*c*]furan-1,3-dione (14g)

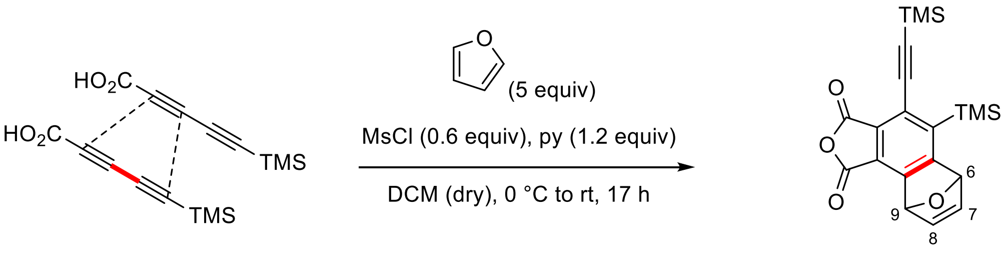

10g ***14g***, 94%

Following general procedure A, 5-(trimethylsilyl)penta-2,4-diynoic acid (**10g**, 28 mg, 0.17 mmol, 1 equiv), methanesulfonyl chloride (8 µL, 0.1 mmol, 0.6 equiv), pyridine (16 µL, 0.2 mmol, 1.2 equiv), furan (62 µL, 0.85 mmol, 5 equiv), and dry dichloromethane (2 mL) were used to prepare the phthalic anhydride derivative **14g**. Purification by MPLC (10:1 hexanes:EtOAc) yielded **14g** (30 mg, 0.079 mmol, 94%) as a pale yellow crystalline solid.

Data for the phthalic anhydride derivative 14g:

^{1}H NMR ($CDCl_3$, 500 MHz): δ 7.09 (dd, *J* = 5.1, 1.4 Hz, 1H, *H7* or *H8*), 7.07 (dd, *J* = 1.4, 5.1 Hz, 1H, *H7* or *H8*), 6.22 (dd, *J* = 2.1, 1.0 Hz, 1H, *H9*), 6.05 (dd, *J* = 2.1, 1.0 Hz, 1H, *H6*), 0.51, [s, 9H, Ar-Si(CH_3)$_3$], and 0.31 [s, 9H, C≡C-Si(CH_3)$_3$].

^{13}C{^{1}H} NMR ($CDCl_3$, 126 MHz): δ 164.8, 161.8, 161.0, 147.5, 143.2, 143.0, 142.5, 126.9, 125.7, 123.0, 110.3, 100.4, 82.6, 79.46, 0.95, and -0.60.

HRMS (ESI-TOF) *m/z*: $[M+H^+]^+$ Calcd for $C_{20}H_{23}O_4Si_2^+$ 383.1129; Found 383.1110.

IR (neat): 2955, 2889, 2157, 1846, 1773, 1375, 1325, 1248, 1209, 1181, 837, and 710 cm^{-1}.

mp: 138–140 °C.

(±)-5-(Triisopropylsilyl)-4-((triisopropylsilyl)ethynyl)-6,9-dihydro-6,9-epoxynaphtho[1,2-*c*]furan-1,3-dione (14h)

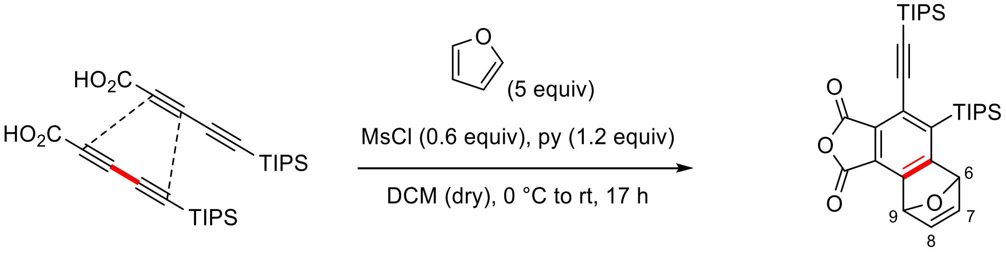

10h ***14h***, 80%

Following general procedure A, 5-(triisopropylsilyl)penta-2,4-diynoic acid (**10h**, 50 mg, 0.20 mmol, 1 equiv), methanesulfonyl chloride (9 µL, 0.12 mmol, 0.6 equiv), pyridine (19 µL, 0.24 mmol, 1.2 equiv), furan (68 mg, 1.0 mmol, 5 equiv), and dry dichloromethane (4.0 mL) were used to prepare the phthalic anhydride derivative **14h**. Purification by MPLC (19:1 hexanes:EtOAc) yielded the phthalic anhydride derivative **14h** (44 mg, 0.080 mmol, 80%) as a white crystalline solid.

Data for the phthalic anhydride derivative 14h:

^{1}H NMR ($CDCl_3$, 500 MHz): δ 7.10 (dd, *J* = 5.5, 1.7 Hz, 1H, *H8*), 7.08 (dd, *J* = 5.5, 1.9 Hz, 1H, *H7*), 6.24 (dd, *J* = 1.8, 0.8 Hz, 1H, *H9*), 6.11 (dd, *J* = 1.7, 0.9 Hz, 1H, *H6*), 1.98 (sept., *J* = 7.5 Hz, 3H, C5Si[C*H*(CH_3)$_2$]$_3$), and 1.27–1.14 (overlapped m, 39H, C5Si[CH(C*H*$_3$)$_2$]$_3$, C≡C-Si[C*H*(CH_3)$_2$]$_3$, and C≡C-Si[CH(C*H*$_3$)$_2$]$_3$).

^{13}C{^{1}H} NMR ($CDCl_3$, 126 MHz): δ 166.3, 161.8, 160.9, 147.0, 143.3, 142.3, 141.4, 128.2, 127.2, 123.0, 108.4, 102.0, 83.7, 79.4, 19.4, 18.7, 13.6, and 11.7.

HRMS (ESI-TOF) *m/z*: [M+H$^+$]$^+$ Calcd for $C_{32}H_{47}O_4Si_2^+$ 551.3007; Found 551.3016.

IR (neat): 2944, 2890, 2865, 1846, 1777, 1192, 908, 884, 806, 714, and 669 cm^{-1}.

mp: 123–125 °C.

1-Benzyl-4-phenyl-5-(phenylethynyl)-1*H*-isobenzofuro[4,5-*d*][1,2,3]triazole-6,8-dione (15)

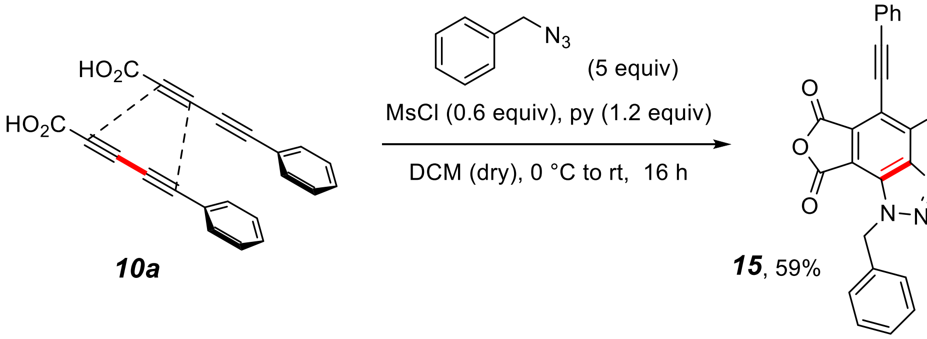

Following general procedure A, 5-phenylpenta-2,4-diynoic acid (**10a**, 50 mg, 0.29 mmol, 1 equiv), methanesulfonyl chloride (14 µL, 0.18 mmol, 0.6 equiv), pyridine (28 µL, 0.35 mmol, 1.2 equiv), benzyl azide (196 mg, 1.47 mmol, 5 equiv), and dry dichloromethane (5.8 mL) were used to prepare the phthalic anhydride derivative **15**. Purification by MPLC (6:1 hexanes:EtOAc) yielded **15** (39 mg, 0.086 mmol, 59%) as a yellow crystalline solid, solutions of which showed a chartreuse fluorescence in ambient room light. This was more intense when the solution was irradiated with a handheld, short wavelength UV lamp.

Data for the phthalic anhydride derivative 15:

^{1}H NMR ($CDCl_3$, 500 MHz): δ 7.98–7.95 (nfom, 2H, ArPhH_o), 7.65–7.58 (overlapped m, 3H, ArPhH_m and ArPhH_p), 7.50–7.45 (overlapped m, 4H, C≡C-PhH_o and CH_2PhH_o), 7.39–7.29 (overlapped m, 6H, C≡C-PhH_m, C≡C-PhH_p, CH_2PhH_m, CH_2PhH_p), and 6.39 (s, 2H, CH_2).

^{13}C{^{1}H} NMR ($CDCl_3$, 126 MHz): δ 161.0, 160.7, 150.0, 145.5, 134.8, 133.0, 132.1, 131.9, 131.2, 130.4, 129.6, 129.2, 128.9, 128.6, 128.4, 128.3, 127.6, 122.3, 115.4, 114.2, 100.3, 82.8, and 54.6.

HRMS (ESI-TOF) *m/z*: [M+H^{+}]$^{+}$ Calcd for $C_{29}H_{18}N_3O_3^{+}$ 456.1343; Found 456.1334.

IR (neat): 3060, 3034, 2955, 2929, 2856, 2214, 1847, 1768, 1492, 1264, 1187, 909, 751, 732, and 692 cm^{-1}.

mp: 227–230 °C.

UV (λ_{max}, 10^{-5} M in $CHCl_3$): 272 (ε/dm^{3} mol^{-1} cm^{-1} 28 000), 309 (34 600), and 420 (7 900) nm.

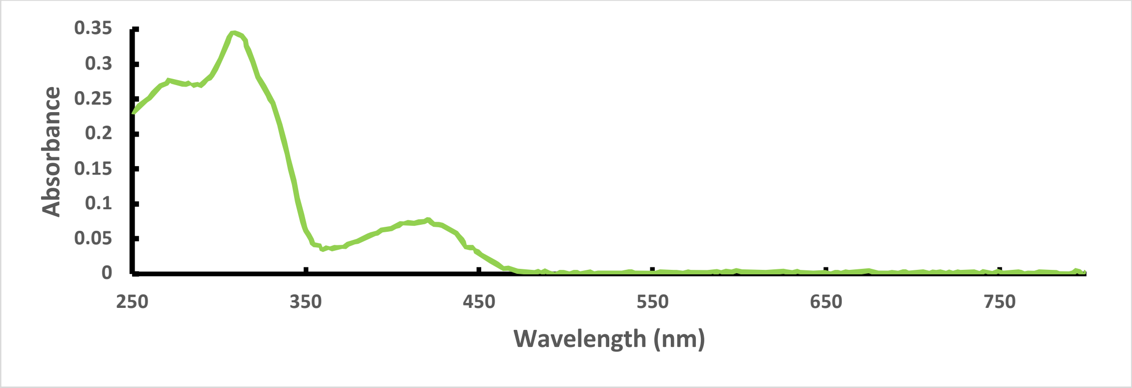

(±)-Diethyl 1,3-Dioxo-5-phenyl-4-(phenylethynyl)-1,3,6,9-tetrahydro-6,9-epoxynaphtho[1,2-*c*]furan-7,8-dicarboxylate (16)

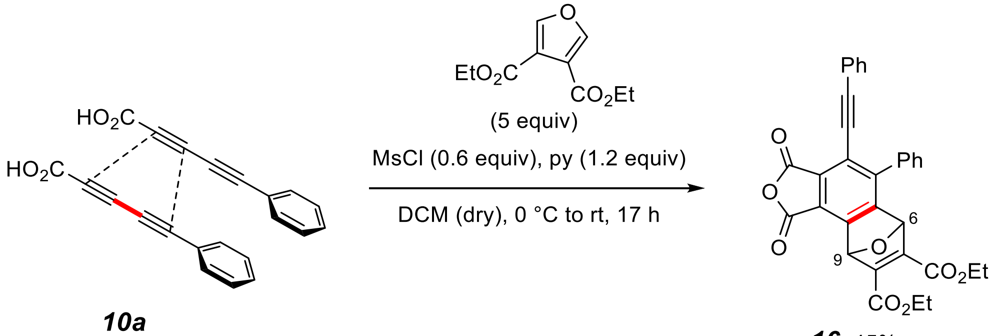

10a ***16***, 45%

Following general procedure A, 5-phenylpenta-2,4-diynoic acid (**10a**, 50 mg, 0.29 mmol, 1 equiv), methanesulfonyl chloride (14 µL, 0.18 mmol, 0.6 equiv), pyridine (28 µL, 0.35 mmol, 1.2 equiv), diethyl furan-3,4-dicarboxylate (311 mg, 1.47 mmol, 5 equiv), and dry dichloromethane (5.8 mL) were used to prepare the phthalic anhydride derivative **16**. Purification by MPLC (6:1 hexanes:EtOAc) yielded the phthalic anhydride derivative **16** (36 mg, 0.067 mmol, 45%) as a yellow crystalline solid.

Data for the phthalic anhydride derivative 16:

^{1}H NMR ($CDCl_3$, 500 MHz): δ 7.59–7.51 (overlapped m, 5H, ArPhH_o, ArPhH_m, and ArPhH_p), 7.38–7.29 (overlapped m, 5H, C≡C-PhH_o, C≡C-PhH_m, and C≡C-PhH_p), 6.52 (d, 1H, J = 1.2 Hz, 1H, *H9*), 6.01 (d, 1H, J = 1.2 Hz, 1H, *H7*), 4.34 (dq, J = 11.0, 7.4 Hz, 1H, CH_aCH$_b$Me), 4.28 (dd, J = 11.0, 7.4 Hz, 1H, CH$_a$CH_bMe), 4.21 (dq, J = 10.8, 7.3 Hz, 1H, CH_aCH$_b$Me'), 4.13 (dd, J = 10.8, 7.3 Hz, 1H, CH$_a$CH_bMe'), 1.35 (t, 3H, J = 7.3 Hz, CH_3), and 1.18 (t, 3H, J = 7.3 Hz, CH_3').

^{13}C{^{1}H} NMR ($CDCl_3$, 126 MHz): δ 161.5, 161.3, 160.9, 160.6, 154.5, 150.8, 150.3, 145.3, 144.7, 134.9, 132.4, 129.9, 129.6, 129.5, 128.61, 128.58, 128.1, 123.2, 122.1, 121.9, 103.3, 83.9, 83.8, 82.9, 62.4, 62.1, 14.2, and 14.1.

HRMS (ESI-TOF) *m/z*: $[M+H^+]^+$ Calcd for $C_{32}H_{23}O_8^+$ 535.1387; Found 535.1369.

IR (neat): 3058, 2983, 2939, 2907, 2215, 1844, 1779, 1750 (br), 1212, and 901 cm^{-1}.

mp: 145–148 °C.

5-Phenyl-4-(phenylethynyl)-7-(phenylthio)isobenzofuran-1,3-dione (17a) and 5-Phenyl-4-(phenylethynyl)-6-(phenylthio)isobenzofuran-1,3-dione (17b)

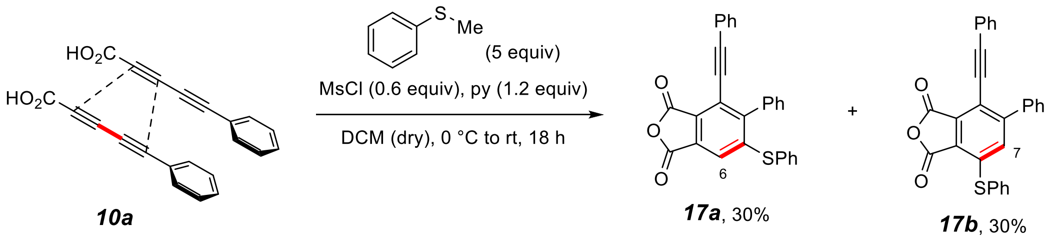

Following general procedure A, 5-phenylpenta-2,4-diynoic acid (**10a**, 50 mg, 0.29 mmol, 1 equiv), methanesulfonyl chloride (14 µL, 0.18 mmol, 0.6 equiv), pyridine (28 µL, 0.35 mmol, 1.2 equiv), thioanisole (182 mg, 1.47 mmol, 5 equiv), and dry dichloromethane (5.8 mL) were used to prepare the phthalic anhydride derivatives **17a** and **17b**. Purification by MPLC (9:1 hexanes:EtOAc) yielded in order of elution **17a** (19 mg, 0.044 mmol, 30%) as a white crystalline solid and **17b** (19 mg, 0.044 mmol, 30%) as a yellow crystalline solid.

Data for the phthalic anhydride derivative 17a (first to elute):

^{1}H NMR ($CDCl_3$, 500 MHz): δ 7.61–7.56 (overlapped m, 3H, ArPhH_m and ArPhH_p), 7.53–7.48 (m, 5H, SPhH_o, SPhH_m, SPhH_p), 7.46 (nfod, J_{app} = 8.1 Hz, 2H, ArPhH_o), 7.33 (tt, J_{app} = 7.6, 1.5 Hz, 1H, C≡C-PhH_p), 7.28 (nfodd, J_{app} = 7.6, 7.6 Hz, 2H, C≡C-PhH_m), 7.23 (nfod, J_{app} = 7.1 Hz, 2H, C≡C-PhH_o), and 7.21 (s, 1H, *H6*).

^{13}C{^{1}H} NMR ($CDCl_3$, 126 MHz): δ 162.5, 161.1, 152.1, 148.4, 136.3, 135.7, 132.3, 131.3, 130.72, 130.69, 129.8, 129.6, 129.5, 129.4, 128.9, 128.5, 126.0, 122.7, 121.9, 120.5, 103.1, and 83.2.

HRMS (ESI-TOF) *m/z*: $[M+H^+]^+$ Calcd for $C_{28}H_{17}O_3S^+$ 433.0893; Found 433.0882.

IR (neat): 3056, 2918, 2204, 1840, 1762, 1575, 1440, 1380, 1338, 1288, 1217, 1165, 906, 751, and 687 cm^{-1}.

mp: 214–218 °C.

Data for the phthalic anhydride derivative 17b (second to elute):

^{1}H NMR ($CDCl_3$, 500 MHz): δ 7.66–7.62 (m, 2H, ArPhH_o), 7.53–7.49 (m, 3H, ArPhH_m and ArPhH_p), 7.46–7.41 (overlapped m, 7H, C≡C-PhH_o , SPhH_o, SPhH_m, and SPhH_p), 7.36–7.29 (m, 3H, C≡C-PhH_m and C≡C-PhH_p), and 7.06 (s, 1H, *H7*).

^{13}C{^{1}H} NMR ($CDCl_3$, 126 MHz): δ 161.4, 161.3, 151.5, 142.4, 137.7, 135.8, 132.5, 132.3, 132.1, 130.7, 130.6, 129.6, 129.31, 129.28, 128.6, 128.4, 127.9, 124.4, 122.3, 117.2, 101.5, and 83.5.

HRMS (ESI-TOF) *m/z*: $[M+H^+]^+$ Calcd for $C_{28}H_{17}O_3S^+$ 433.0893; Found 433.0882.

IR (neat): 3068, 3054, 2918, 2214, 1853, 1834, 1780, 1769, 1578, 1490, 1299, 1216, 1143, 883, 753, 724, 690, and 630 cm^{-1}.

mp: 210–216 °C.

(±)-5-Phenyl-4-(phenylethynyl)-10-(propan-2-ylidene)-6,9-dihydro-6,9-methanonaphtho[1,2-*c*]furan-1,3-dione (18)

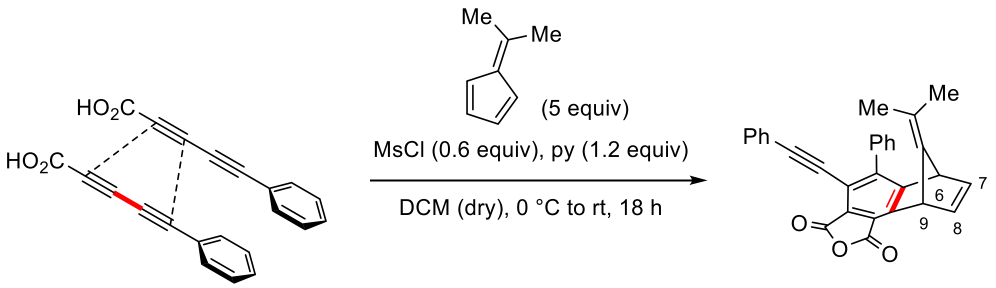

10a ***18***, 67%

Following general procedure A, 5-phenylpenta-2,4-diynoic acid (**10a**, 50 mg, 0.29 mmol, 1 equiv), methanesulfonyl chloride (14 µL, 0.18 mmol, 0.6 equiv), pyridine (28 µL, 0.35 mmol, 1.2 equiv), 6,6-dimethylfulvene (156 mg, 1.47 mmol, 5 equiv), and dry dichloromethane (5.8 mL) were used to prepare the phthalic anhydride derivative **18**. Purification by MPLC (6:1 hexanes:EtOAc) yielded the phthalic anhydride derivative **18** (41 mg, 0.096 mmol, 67%) as a yellow crystalline solid.

Data for the phthalic anhydride derivative 18:

^{1}H NMR ($CDCl_3$, 500 MHz): δ 7.60–7.52 (overlapped m, 3H, ArPhH_m and ArPhH_p), 7.50–7.40 (br m, 2H, ArPhH_o), 7.35–7.25 (overlapped m, 5H, C≡C-PhH_o, C≡C-PhH_m, and C≡C-PhH_p), 7.03 (ddd, 1H, J = 5.4, 3.3, 0.7 Hz, 1H, *H8*), 6.96 (ddd, 1H, J = 5.3, 3.3, 0.8 Hz, 1H, *H7*), 5.13 (ddd, 1H, J = 3.4, 1.7, 0.9 Hz, *H6*), 4.40 (ddd, 1H, J = 3.4 1.7, 0.9 Hz, *H9*), 1.60 (s, 3H, CH_3), and 1.48 (s, 3H, CH_3')).

^{13}C{^{1}H} NMR ($CDCl_3$, 126 MHz): δ 162.5, 161.7, 159.4, 159.2, 151.1, 143.1, 142.8, 142.7, 136.3, 132.2, 129.7, 129.4, 128.9, 128.5 (2 overlapping resonances), 126.2, 122.4, 121.9, 119.5, 108.4, 101.1, 84.3, 50.3, 48.9, 19.3, and 19.2.

HRMS (ESI-TOF) *m/z*: $[M+H^+]^+$ Calcd for $C_{30}H_{21}O_3^+$ 429.1485; Found 429.1469.

IR (neat): 3059, 3021, 2978, 2914, 2858, 2213, 1835, 1766, 902, 887, 756, 733, 719, 701, and 689 cm^{-1}.

mp: 192–194 °C.

Ethyl 6,8-Dioxo-4-phenyl-5-(phenylethynyl)-6,8-dihydro-3*H*-furo[3,4-*e*]indazole-1-carboxylate (19)

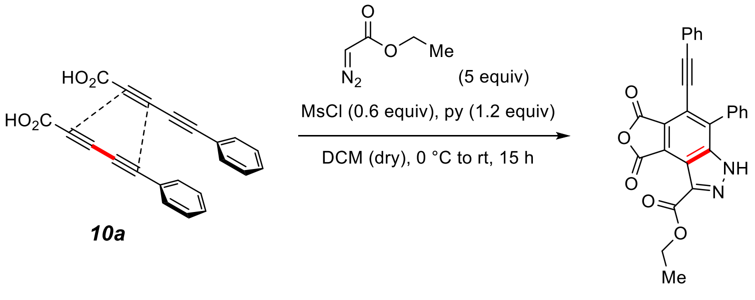

19, 56%

Following general procedure A, 5-phenylpenta-2,4-diynoic acid (**10a**, 50 mg, 0.29 mmol, 1 equiv), methanesulfonyl chloride (14 µL, 0.18 mmol, 0.6 equiv), pyridine (28 µL, 0.35 mmol, 1.2 equiv), ethyl diazoacetate (168 mg, 1.47 mmol, 5 equiv), and dry dichloromethane (5.8 mL) were used to prepare the phthalic anhydride derivative **19**. Purification by MPLC (3:1 hexanes:EtOAc) yielded the phthalic anhydride derivative **19** (36 mg, 0.082 mmol, 56%) as a yellow crystalline solid, solutions of which showed a yellow-chartreuse fluorescence in ambient room light. This was more intense when the solution was irradiated with a handheld, short wavelength UV lamp.

Data for the phthalic anhydride derivative 19:

^{1}**H NMR** ($CDCl_3$, 500 MHz): δ 10.83 (br s, 1H, N*H*), 7.71 (nfod, 2H, J_{app} = 8.2 Hz, ArPhH_o), 7.67 (nfodd, J_{app} = 7.4, 7.4 Hz, 2H, ArPhH_m), 7.36 (tt, *J* = 7.2, 1.6 Hz, 1H, ArPhH_p), 7.41 (nfod, 2H, J_{app} = 8.1 Hz, C≡C-PhH_o), 7.36 (tt, *J* = 7.4, 1.6 Hz, 1H, C≡C-PhH_p), 7.32 (nfodd, J_{app} = 7, 7 Hz, 2H, C≡C-PhH_m), 4.59 (q, *J* = 7.3 Hz, 2H, CH_2), and 1.47 (t, *J* = 7.3 Hz, 3H, CH_3).

^{13}**C{^{1}H} NMR** ($CDCl_3$, 126 MHz): δ 161.69, 161.65, 159.8, 144.2, 140.1, 135.3, 132.9, 132.2, 130.5, 129.8, 129.6, 128.8, 128.6, 128.4, 124.8, 122.0, 117.9, 114.4, 101.6, 82.8, 62.8, and 14.3.

HRMS (ESI-TOF) *m/z*: $[M+H^+]^+$ Calcd for $C_{26}H_{17}N_2O_5^+$ 437.1132; Found 437.1124.

IR (neat): 3282, 3059, 2996, 2937, 2214, 1848, 1779, 1708, 1443, 1296, 1188, 901, 748, and 684 cm^{-1}.

mp: 253–256 °C.

UV (λ_{max}, 10^{-5} M in $CHCl_3$): 280 (ε/dm^3 mol^{-1} cm^{-1} 23 400) and 312 (24 300) nm.

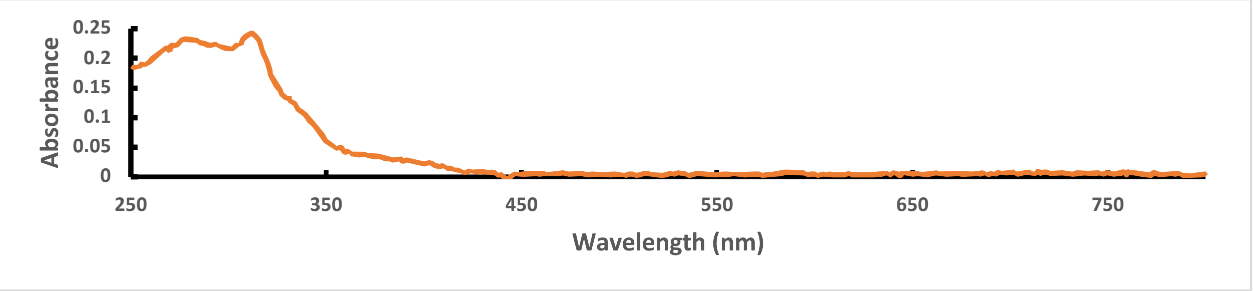

3-Mesityl-4-phenyl-5-(phenylethynyl)isobenzofuro[5,4-*d*]isoxazole-6,8-dione (20)

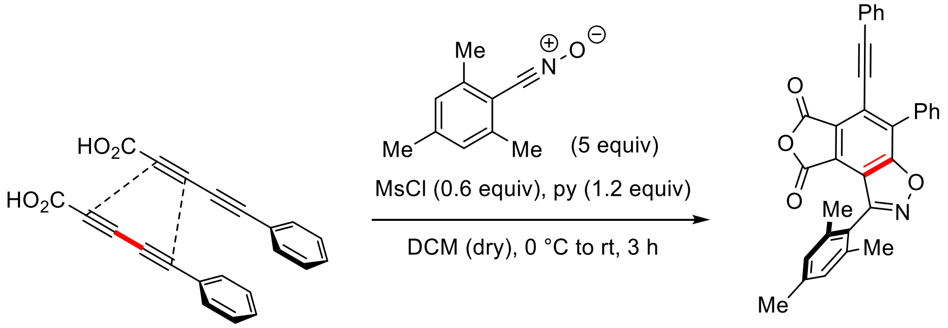

10a ***20***, 75%

Following general procedure A, 5-phenylpenta-2,4-diynoic acid (**10a**, 20 mg, 0.12 mmol, 1 equiv), methanesulfonyl chloride (5.6 µL, 0.072 mmol, 0.6 equiv), pyridine (11.6 µL, 0.14 mmol, 1.2 equiv), mesitylenecarbonitrile oxide[15] (95 mg, 0.6 mmol, 5 equiv), and dry dichloromethane (3 mL) were used to prepare the benzisoxazole derivative **20**. Purification by MPLC (10:1 hexanes:EtOAc) yielded **20** (21 mg, 0.043 mmol, 75%) as a neon yellow crystalline solid.

Data for the phthalic anhydride derivative 20:

^{1}H NMR ($CDCl_3$, 500 MHz): δ 7.94 (nfod, J_{app} = 8.0 Hz, 2H, ArPhH_o), 7.68–7.60 (overlapped m, 3H, ArPhH_m, ArPhH_p), 7.50 (nfod, J_{app} = 8.0 Hz, 2H, C≡C-PhH_o), 7.42–7.35 (overlapped m, 3H, C≡C-PhH_m and C≡C-PhH_p), 7.04 (s, 2H, MesC3/5*H*), 2.40 (s, 3H, Mes4CH_3), and 2.10 (s, 6H, Mes2/6CH_3).

^{13}C{^{1}H} NMR ($CDCl_3$, 126 MHz): δ 166.1, 161.1, 159.2, 157.9, 140.3, 137.5, 134.7, 132.4, 131.3, 130.6, 130.5, 130.1, 129.4, 128.69 (HSQC shows correlation with three distinct aryl protons), 125.3, 123.2, 121.9, 121.3, 118.0, 103.5, 83.1, 21.5, and 20.4.

HRMS (ESI-TOF) *m/z*: $[M+H^+]^+$ Calcd for $C_{26}H_{17}N_2O_5^+$ 437.1132; Found 437.1124.

IR (neat): 2917, 2209, 1846, 1774, 1610, 1280, 1218, 906, and 698 cm^{-1}.

mp: 232–235 °C.

8-Acetyl-5,7-dimethyl-6-phenyl-4-(prop-1-yn-1-yl)-1*H*-furo[3,4-*e*]isoindole-1,3(7*H*)-dione (21a) and 6-Acetyl-5,7-dimethyl-8-phenyl-4-(prop-1-yn-1-yl)-1*H*-furo[3,4-*e*]isoindole-1,3(7*H*)-dione (21b)

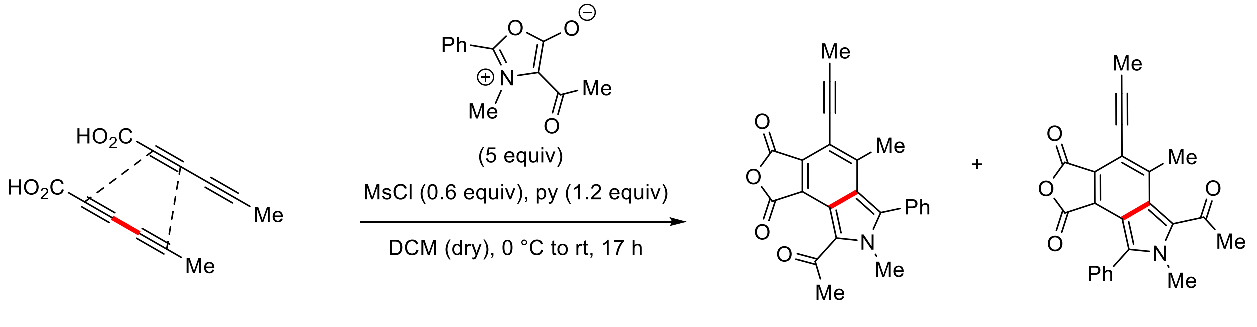

10f ***21a***, 26% ***21b***, 41%

Following general procedure A, hexa-2,4-diynoic acid (**10f**, 20 mg, 0.185 mmol, 1 equiv), methanesulfonyl chloride (8.6 µL, 0.112 mmol, 0.6 equiv), pyridine (18 µL, 0.22 mmol, 1.2 equiv), the indicated munchnone derivative (4-acetyl-3-methyl-2-phenyloxazol-3-ium-5-olate)[16] (200 mg, 0.92 mmol, 5 equiv), and dry dichloromethane (3 mL) were used to prepare the isoindole derivatives **21a** and **21b**. Purification by MPLC (7:3 hexanes:EtOAc) yielded, in order of elution, **21a** (9 mg, 0.024 mmol, 26%) as an orange crystalline solid, and **21b** (14 mg, 0.038 mmol, 41%) as an crystalline orange solid.

Data for the isoindole derivative [first to elute] 21a:

^{1}H NMR ($CDCl_3$, 500 MHz): δ 7.62–7.52 (m, 3H), 7.38–7.35 (nfom, 2H, PhH_m), 3.78 (s, 3H, *N*-CH_3), 2.71 (s, 3H, C(O)CH_3), 2.24 (s, 3H, ArCH_3), and 2.19 (s, 3H, C≡C-CH_3).

^{13}C{^{1}H} NMR ($CDCl_3$, 126 MHz): δ 193.8, 162.7, 162.6, 146.0, 132.9, 131.3, 131.1, 130.5, 130.1, 128.9, 125.4, 124.7, 121.7, 115.2, 112.4, 96.4, 73.4, 35.6, 33.0, 18.8, and 4.9.

HRMS (ESI-TOF) *m/z*: [M+H$^+$]$^+$ Calcd for $C_{23}H_{18}NO_4^+$ 372.1230; Found 372.1218.

IR (neat): 2950, 2922, 2852, 2235, 1833, 1764, 1688, 1447, 1396, 1375, 1362, 1221, 1157, 1113, 894, and 703 cm^{-1}.

mp: 282–288 °C.

Data for the isoindole derivative [second to elute] 21b:

^{1}H NMR ($CDCl_3$, 500 MHz) : δ 7.61–7.51 (m, 3H), 7.37–7.34 (nfom, 2H, PhH_m), 3.78 (s, 3H, *N*-CH_3), 2.78 (s, 3H, C(O)CH_3), 2.73 (s, 3H, ArCH_3), and 2.25 (s, 3H, C≡C-CH_3).

^{13}C{^{1}H} NMR ($CDCl_3$, 126 MHz): δ 194.7, 162.9, 161.2, 142.4, 131.34, 131.29, 131.2, 130.1, 129.9, 128.4, 127.3, 126.6, 123.9, 114.3, 114.2, 97.6, 73.9, 35.6, 33.9, 20.9, and 5.0.

HRMS (ESI-TOF) *m/z*: [M+H$^+$]$^+$ Calcd for $C_{23}H_{18}NO_4^+$ 372.1230; Found 372.1219.

IR (neat): 3057, 2964, 2917, 2847, 2235, 1835, 1768, 1663, 1549, 1510, 1360, 1243, 1215, 1105, 979, 898, and 701 cm^{-1}.

mp: 158–162 °C.

(±)-*tert*-Butyl (6R)-1,3-dioxo-5-phenyl-4-(phenylethynyl)-1,3,6,9-tetrahydro-6,9-epiminonaphtho[1,2-c]furan-10-carboxylate (22)

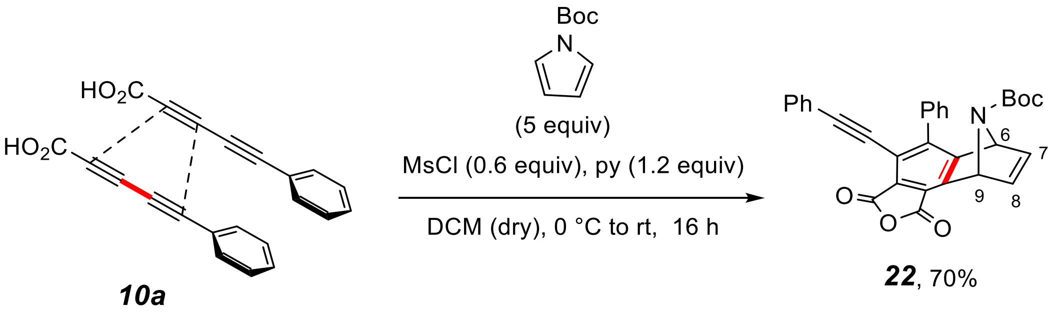

Following general procedure A, 5-phenylpenta-2,4-diynoic acid (**10a**, 25 mg, 0.15 mmol, 1 equiv), methanesulfonyl chloride (7 µL, 0.09 mmol, 0.6 equiv), pyridine (15 µL, 0.18 mmol, 1.2 equiv), *N*-Boc-pyrrole (123 mg, 0.74 mmol, 5 equiv), and dry dichloromethane (3 mL) were used to prepare the phthalic anhydride derivative **22**. Purification by MPLC (4:1 hexanes:EtOAc) yielded **22** (25 mg, 0.05 mmol, 70%) as a light yellow crystalline solid.

Data for the phthalic anhydride derivative 22:

^{1}H NMR ($CDCl_3$, 500 MHz): δ 7.60–7.53 (m, 3H, overlapped m, 3H, ArPhH_m and ArPhH_p), ~7.6–7.4 (vbr s, 2H, ArPhH_o), 7.38–7.33 (overlapped m, 3H, C≡C-PhH_o and C≡C-PhH_p), 7.30 (nfodd, J_{app} = 7.5, 7.5 Hz, 2H, C≡C-PhH_m), 7.11–7.05 (overlapped br m, 2H, *H7* and *H8*), 6.12 (nfod, J_{app} = 3.2 Hz, 1H, *H9*), 5.53 (br s, 1H, *H6*), and 1.37 [s, 9H, COC(CH_3)$_3$].

^{13}C{^{1}H} NMR ($CDCl_3$, 126 MHz): δ 161.5 (br, C1), 161.2 (C3), 156.9, 154.5 (br), 147.8 (vbr), 143.6 (vbr), 142.4 (vbr), 135.2 (C9b), 132.3 (C≡C-Ph$_o$), 129.8 (ArPh$_p$), 129.6 (ArPh$_o$), 129.4 (C≡C-Ph$_p$), 128.7 (ArPh$_m$), 128.6 (C≡C-Ph$_m$), 126.7, 122.1 (C≡C-Ph$_{ipso}$), 120.6 (C4), 102.4 (alkyne), 84.0 (Me$_3$*C*O), 82.1 (alkyne), 65.8 (br, C6), 64.9 (br, C9), and 28.2 (CH_3). (Resonance for two carbon atoms not observable).

HRMS (ESI-TOF) *m/z*: $[M+H^+]^+$ Calcd for $C_{31}H_{24}NO_5^+$ 490.1649; Found 490.1646.

IR (neat): 3064, 3010, 2976, 2929, 2872, 2208, 1839, 1782, 1698, 1491, 1391, 1365, 1320, 1279, 1251, 1176, 1155, 1091, 1076, 893, and 714 cm^{-1}.

mp: 200–205 °C.

(±)-5-Phenyl-4-(phenylethynyl)-6,9-dihydro-6,9-methanonaphtho[1,2-c]furan-1,3-dione (23)

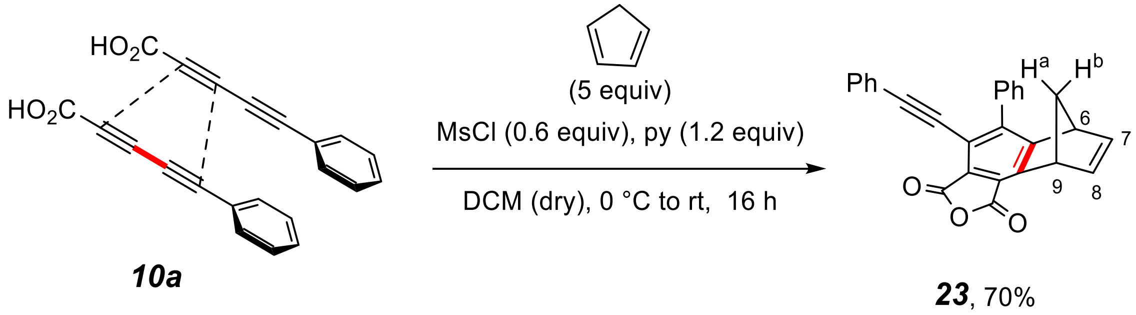

Following general procedure A, 5-phenylpenta-2,4-diynoic acid (**10a**, 25 mg, 0.15 mmol, 1 equiv), methanesulfonyl chloride (7 µL, 0.09 mmol, 0.6 equiv), pyridine (15 µL, 0.18 mmol, 1.2 equiv), freshly cracked cyclopentadiene (61 µL, 0.74 mmol, 5 equiv), and dry dichloromethane (3 mL) were used to prepare the phthalic anhydride derivative **23**. Purification by MPLC (9:1 hexanes:EtOAc) yielded **23** (25 mg, 0.052 mmol, 70%) as a white crystalline solid.

Data for the phthalic anhydride derivative 23:

^{1}H NMR ($CDCl_3$, 500 MHz): δ 7.58–7.49 (overlapped m, 3H, ArPhH_m and ArPhH_p), ~7.5–7.3 (vbr m, 2H, ArPhH_o), 7.36–7.31 (overlapped m, 3H, C≡C-PhH_o and C≡C-PhH_p), 7.29 (nfodd, J_{app} = 7.2, 7.2 Hz, 2H, C≡C-PhH_m), 6.93 (dddd, J = 5.2, 3.1, 0.7, 0.7 Hz, 1H, *H7*), 6.90 (dddd, J = 5.2, 3.1, 0.7, 0.7 Hz, 1H, *H8*), 4.67 (ddddd, J = 3.1, 1.6, 1.6, 1.6, 0.7 Hz, *H9*), 3.96 (ddddd, J = 3.1, 1.6, 1.6, 1.6, 0.7 Hz, *H6*), 2.46 (ddd, J = 8.0, 1.6, 1.6 Hz, H^b), and 2.36 (ddddd, J = 8.0, 1.6, 1.6, 0.7, 0.7 Hz, H^a).

^{13}C{^{1}H} NMR ($CDCl_3$, 126 MHz): δ 162.4 (anhydride), 161.7 (anhydride), 160.8 (C9a), 152.8, 143.8, 143.5, 143.1, 136.3, 132.2 (C3a), 129.8 (br, C9b), 129.4, 128.8, 128.5, 128.4, 126.6, 123.0, 122.4, 119.5, 101.0, 84.4, 69.4, 50.0, and 48.5.

HRMS (ESI-TOF) *m/z*: $[M+H^+]^+$ Calcd for $C_{27}H_{17}O_3^+$ 389.1172; Found 389.1171.

IR (neat): 3070, 3052, 2988, 2974, 2941, 2863, 2204, 1831, 1764, 1489, 1441, 1423, 1384, 1362, 1317, 1299, 1278, 1241, 1208, 1189, 1164, 1136, 898, 873, 742, 686, 621, and 522 cm^{-1}.

mp: 219–222 °C.

(±)-5-Phenyl-4-(phenylethynyl)-6,11-dihydro-6,11-[1,2]benzenoanthra[1,2-c]furan-1,3-dione (24)

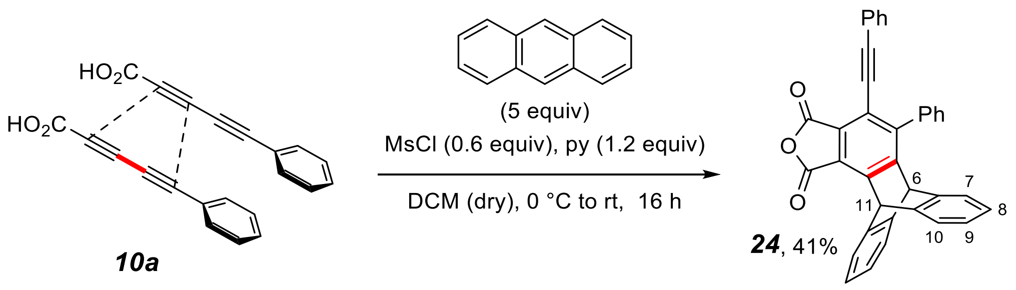

Following general procedure A, 5-phenylpenta-2,4-diynoic acid (**10a**, 25 mg, 0.15 mmol, 1 equiv), methanesulfonyl chloride (7 µL, 0.09 mmol, 0.6 equiv), pyridine (15 µL, 0.18 mmol, 1.2 equiv), anthracene (132 mg, 0.74 mmol, 5 equiv), and dry dichloromethane (3 mL) were used to prepare the phthalic anhydride derivative **24**. Purification by MPLC (9:1 hexanes:EtOAc) yielded **24** (15 mg, 0.03 mmol, 41%) as a white crystalline solid.

Data for the phthalic anhydride derivative 24:

^{1}H NMR ($CDCl_3$, 500 MHz): δ 7.68–7.61 (overlapped m, 3H, ArPhH_m and ArPhH_p), 7.56 (dd, *J* = 7.2, 1.5 Hz, 2H, *H10* and *H12*), 7.40 (nfod, J_{app} = 8.1 Hz, 2H, *H7* and *H15*), 7.32–7.21 (overlapped m, 7H, C≡C-Ph*Hs* and ArPhH_o), 7.10 (ddd, *J* = 7.5, 7.5, 1.4 Hz, 2H, *H9* and *H13*), 7.06 (ddd, *J* = 7.3, 7.3, 1.4 Hz, 2H, *H8* and *H14*), 6.56 (s, 1H, *H11*), and 5.52 (s, 1H, *H6*).

^{13}C{^{1}H} NMR ($CDCl_3$, 126 MHz): δ 162.7, 161.1, 153.2, 146.5, 145.5, 143.4, 143.0, 136.4, 132.2, 129.7, 129.5, 129.0, 128.8, 128.4, 126.5, 126.4, 126.4, 125.0, 124.4, 123.8, 122.2, 101.8, 84.0, 51.8, and 48.9. (Resonance for one carbon atom not observable).

HRMS (ESI-TOF) *m/z*: $[M+H^+]^+$ Calcd for $C_{36}H_{21}O_3^+$ 501.1485; Found 501.1486.

IR (neat): 3053, 2926, 2208, 1839, 1769, 1490, 1459, 1443, 1426, 1401, 1361, 1286, 1232, 1169, 1155, 904, 887, 874, 746, 699, 689, 613, and 522 cm^{-1}

mp: 288–294 °C.

5-(4-Chlorophenyl)-4-((4-chlorophenyl)ethynyl)-2-phenyl-6,9-dihydro-1H-6,9-epoxybenzo[e]isoindole-1,3(2H)-dione (25a)

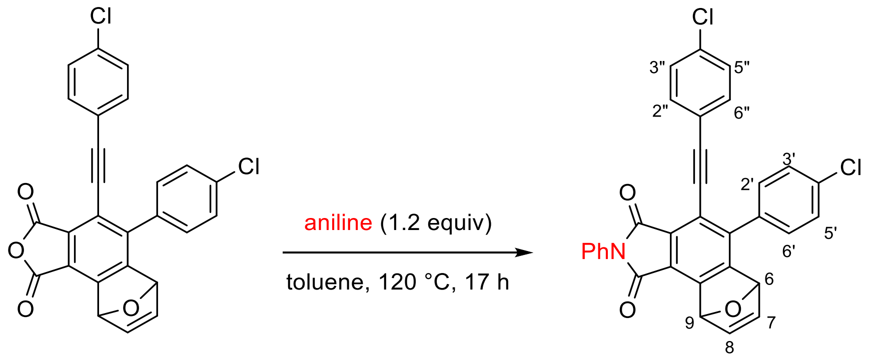

14c ***25a***, 83%

Following general procedure B, 5-(4-chlorophenyl)-4-((4-chlorophenyl)ethynyl)-6,9-dihydro-6,9-epoxynaphtho[1,2-*c*]furan-1,3-dione (**14c**, 20 mg, 0.044 mmol, 1 equiv), aniline (5 mg, 0.054 mmol, 1.2 equiv), and toluene (0.9 mL) were used to prepare the phthalimide derivative **25a**. Purification by MPLC (3:1 hexanes:EtOAc) yielded the phthalimide derivative **25a** (19 mg, 0.036 mmol, 83%) as a yellow crystalline solid.

Data for the phthalimide derivative 25a:

^{1}H NMR ($CDCl_3$, 500 MHz): δ 7.55 (nfod, J_{app} = 8.7 Hz, 2H, *H3′* and *H5′*), 7.55–7.51 (overlapped m, 2H, PhH_o), 7.46–7.41 (overlapped m, 3H, PhH_m and PhH_p), 7.40 (br nfod, J_{app} = 7.8 Hz, 2H, *H2′* and *H6′*), 7.31 (nfod, J_{app} = 8.6 Hz, 2H, *H3″* and *H5″*), 7.27 (nfod, J_{app} = 8.6 Hz, 2H, *H2″* and *H6″*), 7.19 (dd, *J* = 5.4, 2.0 Hz, 1H, *H8*), 7.13 (dd, *J* = 5.5, 1.9 Hz, 1H, *H7*), 6.38 (dd, *J* = 1.9, 0.7 Hz, 1H, *H9*), and 5.64 (dd, *J* = 1.9, 0.8 Hz, 1H, *H6*).

^{13}C{^{1}H} NMR ($CDCl_3$, 126 MHz): δ 165.8, 165.4, 155.4, 146.9, 142.9, 142.8, 139.7, 135.7, 135.3, 134.4, 133.3, 131.7, 130.9, 129.4, 129.0, 128.9, 128.5, 128.1, 126.9, 123.2, 120.8, 117.7, 99.4, 85.5, 81.6, and 80.7.

HRMS (ESI-TOF) *m/z*: $[M+H^+]^+$ Calcd for $C_{32}H_{18}Cl_2NO_3^+$ 534.0658; Found 534.0649.

IR (neat): 3063, 2961, 2928, 2856, 2211, 1767, 1716, 1499, 1389, 1365, and 1090 cm^{-1}.

mp: 201–203 °C.

(±)-5-(4-Chlorophenyl)-4-((4-chlorophenyl)ethynyl)-2-(prop-2-yn-1-yl)-6,9-dihydro-1*H*-6,9-epoxybenzo[*e*]isoindole-1,3(2*H*)-dione (25b)

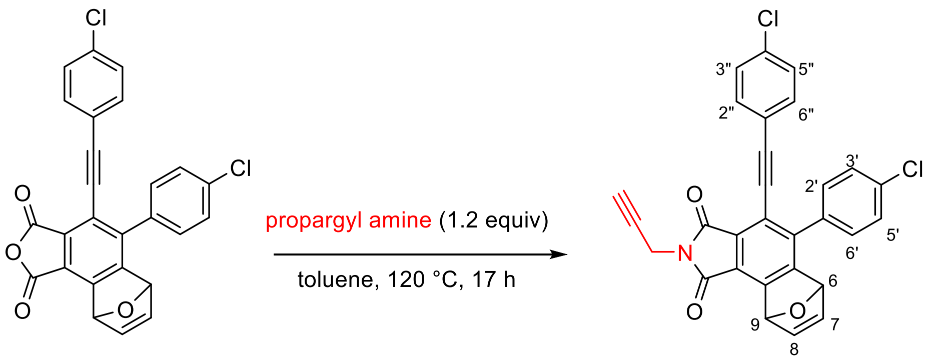

Following general procedure B, 5-(4-chlorophenyl)-4-((4-chlorophenyl)ethynyl)-6,9-dihydro-6,9-epoxynaphtho[1,2-*c*]furan-1,3-dione (**14c**, 20 mg, 0.044 mmol, 1 equiv), propargylamine (3 mg, 0.054 mmol, 1.2 equiv), and toluene (0.9 mL) were used to prepare the phthalimide derivative **25b**. Purification by MPLC (3:1 hexanes:EtOAc) yielded **25b** (21 mg, 0.044 mmol, 98%) as a white crystalline solid.

Data for the phthalimide derivative 25b:

^{1}H NMR ($CDCl_3$, 500 MHz): δ 7.53 (nfod, J_{app} = 8.7 Hz, 2H, *H3′* and *H5′*), 7.36 (br nfod, J_{app} = 7.8 Hz, 2H, *H2′* and *H6′*), 7.33 (nfod, J_{app} = 8.7 Hz, 2H, *H3″* and *H5″*), 7.30 (nfod, J_{app} = 8.7 Hz, 2H, *H2″*, and *H6″*), 7.16 (dd, *J* = 5.5, 1.9 Hz, 1H, *H8*), 7.11 (dd, *J* = 5.4, 1.8 Hz, 1H, *H7*), 6.34 (dd, *J* = 1.8, 0.8 Hz, 1H, *H9*), 5.61 (dd, *J* = 1.7, 0.8 Hz, 1H, *H6*), 4.48 (d, *J* = 2.5 Hz, 2H, CH_2), and 2.24 (t, *J* = 2.5 Hz 1H, C≡C-*H*).

^{13}C{^{1}H} NMR ($CDCl_3$, 126 MHz): δ 165.4, 165.1, 155.2, 146.7, 142.9, 142.7, 139.5, 135.7, 135.3, 134.3, 133.3, 130.9, 129.0, 128.9, 128.3, 123.2, 120.8, 117.6, 99.3, 85.3, 81.5, 80.6, 71.8, and 27.2. (The internal alkyne carbon for the propargyl group not observed; that carbon in HC≡CCH_2NPhth is at 77.3 ppm; presumably this carbon is superimposed under the upfield most line of the $DCCl_3$ solvent.)[17]

HRMS (ESI-TOF) *m/z*: $[M+H^+]^+$ Calcd for $C_{29}H_{16}{}^{35}Cl_2NO_3^+$ 496.0502; Found 496.0487.

IR (neat): 3297, 3085, 3055, 3031, 2963, 2929, 2217, 1768, 1716, 1490, 1392, 1343, 1132, 1090, 1014, 871, 830, and 705 cm^{-1}.

mp: 213–215 °C.

(±)-Methyl 2-(5-(4-chlorophenyl)-4-((4-chlorophenyl)ethynyl)-1,3-dioxo-1,3,6,9-tetrahydro-2*H*-6,9-epoxybenzo[*e*]isoindol-2-yl)acetate (25c)

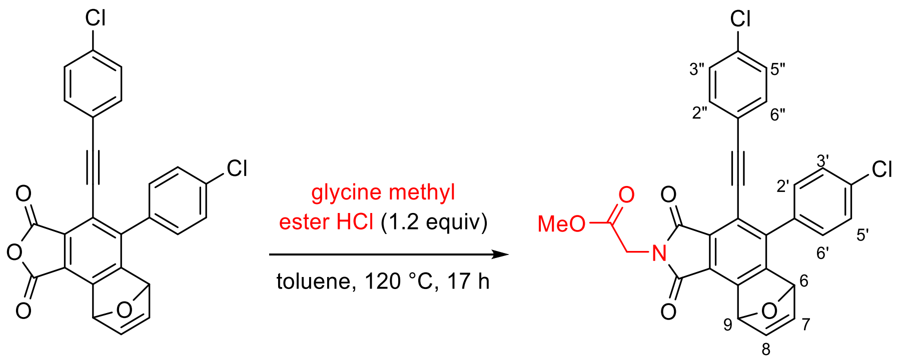

14c ***25c***, 78%

Following general procedure B, 5-(4-chlorophenyl)-4-((4-chlorophenyl)ethynyl)-6,9-dihydro-6,9-epoxynaphtho[1,2-*c*]furan-1,3-dione (**14c**, 20 mg, 0.044 mmol, 1 equiv), glycine methyl ester hydrochloride (7 mg, 0.056 mmol, 1.2 equiv), and toluene (0.9 mL) were used to prepare the phthalimide derivative **25c**. Purification by MPLC (3:1 hexanes:EtOAc) yielded the phthalimide derivative **25c** (18 mg, 0.034 mmol, 78%) as a white crystalline solid.

Data for the phthalimide derivative 25c:

^{1}H NMR ($CDCl_3$, 500 MHz): δ 7.53 (nfod, J_{app} = 8.6 Hz, 2H, *H3′* and *H5′*), 7.37 (br nfod, J_{app} = 7.3 Hz, 2H, *H2′* and *H6′*), 7.31–7.29 (overlapped m, 4H, *H3″*, *H5″*, *H2″*, and *H6″*), 7.16 (dd, *J* = 5.5, 1.9 Hz, 1H, *H8*), 7.11 (dd, *J* = 5.4, 1.8 Hz, 1H, *H7*), 6.33 (dd, *J* = 1.9, 0.7 Hz, 1H, *H9*), 5.62 (dd, *J* = 1.9, 0.9 Hz, 1H, *H6*), 4.48 (s, 2H, CH_2), and 3.79 (s, 3H, CH_3).

^{13}C{^{1}H} NMR ($CDCl_3$, 126 MHz): δ 167.8, 165.9, 165.5, 155.2, 146.8, 142.9, 142.7, 139.5, 135.7, 135.3, 134.3, 133.3, 130.9, 129.0, 128.9, 128.4, 123.3, 120.9, 117.7, 99.3, 85.4, 81.6, 80.6, 52.9, and 39.0.

HRMS (ESI-TOF) *m/z*: $[M+H^+]^+$ Calcd for $C_{29}H_{18}Cl_2NO_5^+$ 530.0557; Found 530.0546.

IR (neat): 3091, 3054, 2954, 2926, 2856, 2206, 1755, 1718, 1490, 1414, 1218, 1092, 1015, 872, 833, and 705 cm^{-1}.

mp: 212–214 °C.

(±)-2-(2-Aminoethyl)-5-phenyl-4-(phenylethynyl)-6,9-dihydro-1*H*-6,9-epoxybenzo[*e*]isoindole-1,3(2*H*)-dione (25d)

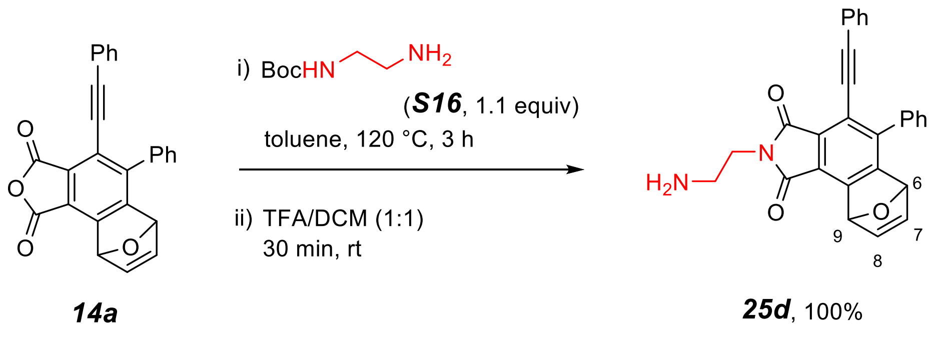

tert-Butyl (2-aminoethyl)carbamate (**S16**, 9 mg, 0.056 mmol, 1.1 equiv) was prepared according to a literature procedure and its proton NMR spectrum has been provided here.[18]

Following general procedure C, 5-phenyl-4-(phenylethynyl)-6,9-dihydro-6,9-epoxynaphtho[1,2-*c*]furan-1,3-dione (**14a**, 20 mg, 0.051 mmol, 1 equiv), *tert*-butyl (2-aminoethyl)carbamate, and toluene (1.0 mL) for step i) and 1:1 DCM/TFA (1.0 mL) for step ii) were used to prepare the phthalimide derivative **25d**. Purification by passage through a silica gel plug (EtOAc to MeOH) yielded the phthalimide derivative **25d** (22 mg, 0.051 mmol, 100%) as the free base and a pale yellow amorphous solid.

Data for the phthalimide derivative 25d:

^{1}H NMR ($CDCl_3$, 500 MHz): δ 7.48–7.42 (m, 3H, ArPhH_m and ArPhH_p), 7.36–7.28 (br m, 2H, ArPhH_o), 7.30–7.22 (m, 5H, C≡C-PhH_o, C≡C-PhH_m, and C≡C-PhH_p), 7.04 (dd, *J* = 5.7, 1.7 Hz, 1H, *H8*), 6.99 (dd, *J* = 5.4, 1.6 Hz, 1H, *H7*), 6.20 (d, *J* = 1.6 Hz, 1H, *H9*), 5.52 (d, *J* = 1.6 Hz, 1H, *H6*), 3.90 (t, *J* = 6.8 Hz, 2H, N-CH_2CH$_2$NH$_2$), and 3.16 (t, *J* = 6.5 Hz, 2H, N-CH$_2$CH_2NH$_2$). *NH_2 resonance not observed.

^{13}C{^{1}H} NMR ($CDCl_3$, 126 MHz): δ 167.2, 166.8, 154.9, 146.5, 142.7, 142.5, 140.8, 135.8, 132.0, 129.5, 129.4, 128.8, 128.6, 128.4, 127.72, 122.69, 122.3, 117.6, 99.7, 85.1, 81.5, 80.5, 39.5, and 36.9.

HRMS (ESI-TOF) *m/z*: $[M+H^+]^+$ Calcd for $C_{28}H_{21}N_2O_3^+$ 433.1547; Found 433.1526.

IR (neat): 3392 (br), 3056, 3026, 2928, 2207, 1765, 1707, 1673, 1436, 1398, 1357, 1200, 1182, 1132, 870, 757, and 722 cm^{-1}.

(±)-2-(3-Aminopropyl)-5-phenyl-4-(phenylethynyl)-6,9-dihydro-1*H*-6,9-epoxybenzo[*e*]isoindole-1,3(2*H*)-dione (25e)

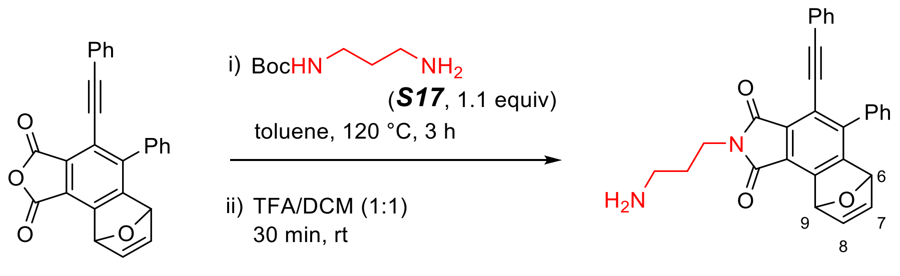

tert-Butyl (3-aminopropyl)carbamate (**S17**, 10 mg, 0.057 mmol, 1.1 equiv) was prepared according to a literature procedure and its proton NMR spectrum has been provided here.[18]

Following general procedure C, 5-phenyl-4-(phenylethynyl)-6,9-dihydro-6,9-epoxynaphtho[1,2-*c*]furan-1,3-dione (**14a**, 20 mg, 0.051 mmol, 1 equiv), *tert*-butyl (3-aminopropyl)carbamate, and toluene (1.0 mL), and 1:1 DCM/TFA (1.0 mL) were used to prepare the phthalimide derivative **25e**. Purification by passage through a silica gel plug (EtOAc to MeOH) yielded the phthalimide derivative **25e** (18 mg, 0.041 mmol, 80%) as the free base and as a pale yellow amorphous solid.

Data for the phthalimide derivative 25e:

^{1}H NMR ($CDCl_3$, 500 MHz): δ 7.52–7.44 (m, 3H, ArPhH_m and ArPhH_p), 7.39–7.33 (br m, 2H, ArPhH_o), 7.32–7.22 (m, 5H, C≡C-PhH_o, C≡C-PhH_m, and C≡C-PhH_p), 7.10 (dd, J = 5.4, 1.8 Hz, 1H, *H8*), 7.05 (dd, J = 5.4, 1.8 Hz, 1H, *H7*), 6.24 (d, J = 1.9 Hz, 1H, *H9*), 5.56 (d, J = 1.8 Hz, 1H, *H6*), 3.81 (t, J = 6.8 Hz, 2H, N-CH_2CH$_2$CH$_2$NH$_2$), 3.04 (t, J = 7.4 Hz, 2H, N-CH$_2$CH$_2$CH_2NH$_2$), and 2.07 (tt, J = 6.6, 6.6 Hz, 2H, N-CH$_2$CH_2CH$_2$NH$_2$). *NH_2 resonance not observed.

^{13}C{^{1}H} NMR ($CDCl_3$, 126 MHz): δ 167.1, 167.0, 155.0, 146.5, 142.9, 142.5, 140.9, 135.8, 132.0, 129.6, 129.4, 128.9, 128.48, 128.45, 127.7, 122.6, 122.4, 118.0, 100.3, 84.8, 81.6, 80.5, 37.3, 34.3, and 26.7.

HRMS (ESI-TOF) *m/z*: [M+H$^+$]$^+$ Calcd for $C_{29}H_{23}N_2O_3^+$ 447.1703; Found 447.1692.

IR (neat): 3469 (br), 3060, 3028, 2946, 2360, 2342, 2208, 1764, 1705, 1678, 1472, 1440, 1204, 1185, 1136, 758, 723, and 702 cm^{-1}.

(±)-2-(4-Aminobutyl)-5-phenyl-4-(phenylethynyl)-6,9-dihydro-1*H*-6,9-epoxybenzo[*e*]isoindole-1,3(2*H*)-dione (25f)

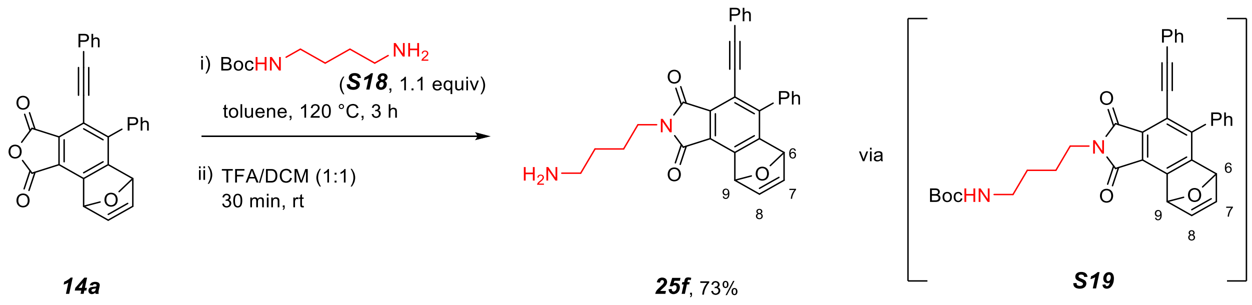

tert-Butyl (4-aminobutyl)carbamate (**S18**, 11 mg, 0.058 mmol, 1.1 equiv) was prepared according to a literature procedure and its proton NMR spectrum has been provided here.[18]

Following general procedure C, 5-phenyl-4-(phenylethynyl)-6,9-dihydro-6,9-epoxynaphtho[1,2-*c*]furan-1,3-dione (**14a**, 20 mg, 0.051 mmol, 1 equiv), *tert*-butyl (4-aminobutyl)carbamate, and toluene (1.0 mL), and 1:1 DCM/TFA (1.0 mL) were used to prepare the phthalimide derivative **25f** (17 mg, 0.037 mmol, 73%) as the free base and as a pale yellow amorphous solid. [Prior to treatment by TFA, a portion of the 4-*N*-Bocbutylimide **S19** was passed through a silica gel plug and the ^{1}H NMR spectrum of this material is provided.]

Crude NMR ^{1}H NMR spectrum for S19:

^{1}H NMR ($CDCl_3$, 500 MHz): δ 7.54 (nfodd, J = 7.1, 7.1 Hz, 2H, ArPhH_m), 7.51 (tt, J = 7.2, 1.7 Hz, 1H, ArPhH_p), 7.46–7.40 (br m, 2H, ArPhH_o), 7.36 (nfod, J = 8.0 Hz, 2H, C≡C-PhH_o), 7.34–7.27 (m, 3H, C≡C-PhH_m and C≡C-PhH_p), 7.15 (dd, J = 5.5, 1.9 Hz, 1H, *H8*), 7.12 (dd, J = 5.5, 1.9 Hz, 1H, *H7*), 6.32 (dd, J = 1.8, 0.8 Hz, 1H, *H9*), 5.62 (dd, J = 1.8, 0.8 Hz, 1H, *H6*), 4.67 (br s, 1H, N*H*), 3.73 (t, J = 7.1 Hz, 2H, N-C$H_2$$CH_2CH_2CH_2$NHBoc), 3.18 (m, 2H, N-$CH_2CH_2CH_2CH_2$NHBoc), 1.77–1.71 (m, 2H, N-CH_2CH_2C$H_2$$CH_2$NHBoc), 1.59–1.54 (m, 2H, N-CH_2C$H_2$$CH_2CH_2NH_2$), and 1.43 [s, 9H, NH(CO)C(CH_3)$_3$]. [Excess of the mono-Boc protected diamine is present in this sample]

Data for the phthalimide derivative 25f:

The sample of this free amine **25f** in $CDCl_3$ gave ^{1}H NMR resonances in that solvent that were broadened such that coupling constants were difficult to discern; therefore, the ^{1}H NMR spectrum and associated line listing are for the spectrum in MeOD-d_3.

^{1}H NMR (MeOD-d_3, 500 MHz): δ 7.62 (nfodd, J = 7.3, 7.3 Hz, 2H, ArPhH_m), 7.58 (tt, J = 7.3, 1.5 Hz, 1H, ArPhH_p), 7.52–7.47 (br m, 2H, ArPhH_o), 7.41–7.32 (m, 5H, C≡C-PhH_o, C≡C-PhH_m, and C≡C-PhH_p), 7.30 (dd, J = 5.6, 1.9 Hz, 1H, *H8*), 7.21 (dd, J = 5.6, 1.9 Hz, 1H, *H7*), 6.29 (dd, J

= 1.9, 0.8 Hz, 1H, *H9*), 5.62 (dd, *J* = 1.9, 0.8 Hz, 1H, *H6*), 3.80 (t, *J* = 6.6 Hz, 2H, N-C$H_2$$CH_2CH_2CH_2NH_2$), 3.02 (t, *J* = 7.6 Hz, 2H, N-$CH_2CH_2CH_2$C$H_2$$NH_2$), 1.88–1.80 (m, 2H, N-$CH_2CH_2$$CH_2CH_2NH_2$), and 1.80–1.71 (m, 2H, N-CH_2CH_2C$H_2$$CH_2NH_2$).

$^{13}C\{^1H\}$ NMR ($CDCl_3$, 126 MHz): δ 167.1, 166.7, 154.7, 146.2, 142.8, 142.5, 140.7, 135.9, 132.0, 129.6, 129.3, 128.8, 128.5, 128.4, 127.9, 122.8, 122.5, 117.5, 99.8, 85.0, 81.6, 80.5, 39.6, 37.2, 25.4, and 24.8.

HRMS (ESI-TOF) *m/z*: $[M+H^+]^+$ Calcd for $C_{30}H_{25}N_2O_3^+$ 461.1860; Found 461.1848.

IR (neat): 3453 (br), 3057, 3028, 2927, 2206, 1764, 1707, 1679, 1441, 1401, 1203, 1184, 1137, 870, 758, 722, and 702 cm^{-1}.

5-(4-Methoxyphenyl)penta-2,4-diynoyl chloride (26)

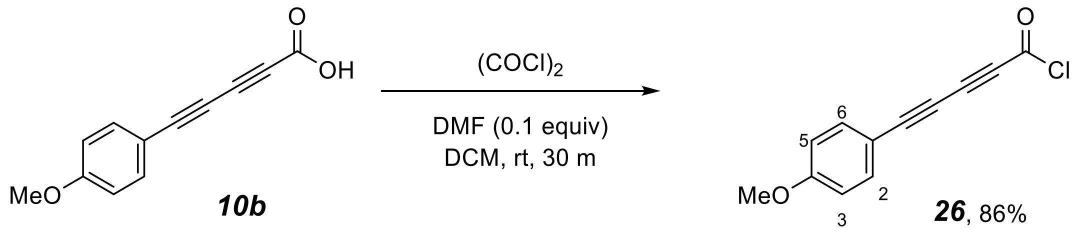

To a stirred solution of oxalyl chloride (64 µL, 0.75 mmol, 3 equiv) in DCM (1 mL) was added DMF (2 µL, 0.025 mmol, 0.1 equiv) at room temperature. Once bubbling of the solution had subsided (~1 minute), 5-(4-methoxyphenyl)penta-2,4-diynoic acid (**10b**, 50 mg, 0.25 mmol, 1 equiv) was added in one portion and the mixture was stirred for 30 minutes. The solvent was subsequently evaporated *in vacuo* and the residue was purified by passing through a silica gel plug (9:1 hexanes:EtOAc) to afford the acid chloride **26** as an orange solid (47 mg, 0.216 mmol, 86%). NOTE: The acid chloride appears to degrade if left on silica for extended time, so it is helpful to purify it quickly on a short silica gel plug. The compound showed reasonably good stability and was routinely stored in a freezer between uses.

Data for the acid chloride derivative 26:

^{1}H NMR ($CDCl_3$, 500 MHz): δ 7.54 (nfod, J_{app} = 8.8 Hz, 2H, *H2* and *H6*), 6.90 (nfod, 2H, *H3* and *H5*), and 3.85 (s, 3H, -OCH_3).

^{13}C{^{1}H} NMR ($CDCl_3$, 126 MHz): 162.3 (MeO*C*), 148.8 (*C*O), 135.6 (C2/6), 114.8 (C3/5), 111.1 (C1), 91.9, 80.1, 73.9, 71.2, and 55.7 (*C*H_3).

HRMS (ESI-TOF) *m/z*: $[M+H^+]^+$ Calcd for $C_{12}H_8{}^{35}ClO_2{}^+$ 219.0207; Found 219.0202.

IR (neat): 2922, 2841, 2212, 2178, 2130, 1718, 1680, 1595, 1507, 1336, 1253, 1176, 1118, 1025, 868, 826, 788, 665, 633, 561, 532, 493, 471, and 429 cm^{-1}.

mp: 66–68 °C.

(±)-5-(4-Methoxyphenyl)-4-(prop-1-yn-1-yl)-6,9-dihydro-6,9-epoxynaphtho[1,2-*c*]furan-1,3-dione (27f) and

(±)-4-((4-Methoxyphenyl)ethynyl)-5-methyl-6,9-dihydro-6,9-epoxynaphtho[1,2-*c*]furan-1,3-dione (28f)

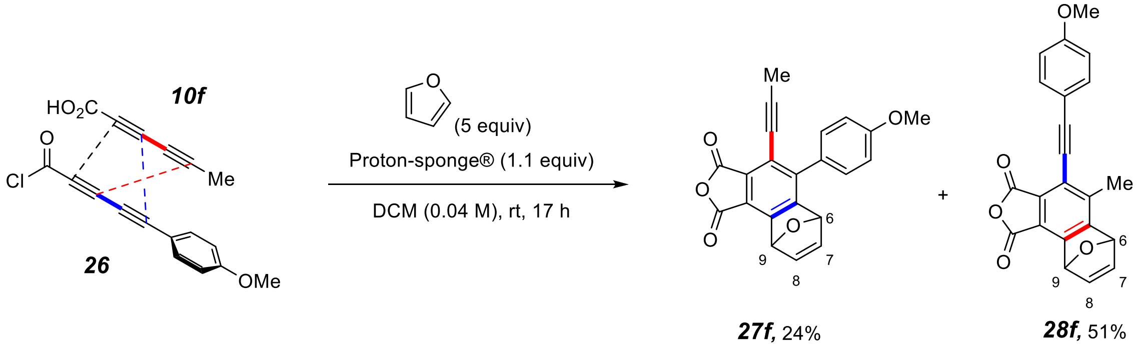

Following general procedure D, 5-(4-methoxyphenyl)penta-2,4-diynoyl chloride (**26**, 11 mg, 0.05 mmol, 1.1 equiv), furan (16.4 µL, 0.225 mmol, 5 equiv), and Proton-sponge® (10.7 mg, 0.05 mmol, 1.1 equiv) were added to a flame dried round-bottom flask and dissolved in dry DCM (0.7 mL). A solution of hexa-2,4-diynoic acid (**10f**, 5 mg, 0.045 mmol, 1 equiv) in DCM (0.7 mL), which had been incubated on 3 Å molecular sieves overnight, was added to the mixture in one portion and stirred overnight. The solvent was evaporated *in vacuo* and the crude mixture was purified by MPLC (7:3 hexanes:EtOAc) to afford, in order of elution, **27f** (3.8 mg, 0.011 mmol, 24%) as a light brown solid and **28f** (8.2 mg, 0.023 mmol, 51%) as an off-white solid.

Data for the phthalic anhydride derivative [first to elute] 27f:

^{1}H NMR ($CDCl_3$, 500 MHz): δ 7.30 (br d, *J* = 8.4 Hz, 2H, *H*C=CHC-OMe), 7.16 (br d, *J* = 5.4 Hz, 1H, *H8*), 7.15 (br d, *J* = 5.4 Hz, 1H, *H7*), 7.04 (nfod, J_{app} = 9.0 Hz, 2H, *H*C=C-OMe), 6.27 (nfom, 1H, *H9*), 5.64 (nfom, 1H, *H6*), 3.90 (s, 3H, ArOCH_3) and 2.05 (s, 3H, C≡C-CH_3).

^{13}C{^{1}H} NMR ($CDCl_3$, 126 MHz): δ 161.54, 161.46, 160.3, 157.4, 148.1, 143.3, 142.8, 142.7, 130.7, 127.6, 127.3, 121.6, 121.5, 114.2, 100.8, 82.0, 80.6, 74.3, 55.5, and 5.2.

HRMS (ESI-TOF) *m/z*: $[M+H^+]^+$ Calcd for $C_{22}H_{15}O_5^+$ 359.0914; Found 359.0903.

IR (neat): 3013, 2920, 2846, 2228, 1838, 1766, 1606, 1515, 1432 and 1174.

mp: 215–220 °C.

Data for the phthalic anhydride derivative [second to elute] 28f:

^{1}H NMR ($CDCl_3$, 500 MHz): δ 7.61 (nfod, J_{app} = 9.0 Hz, 2H, *H*C=CHC-OMe), 7.13–7.10 (m, 2H, *H8* and *H7*), 6.92 (nfod, J_{app} = 9.0 Hz, 2H, *H*C=C-OMe), 6.24 (ddd, *J* = 1.0, 1.0, 1.0 Hz, 1H, *H9*), 5.93 (ddd, *J* = 1.0, 1.0, 1.0 Hz, 1H, *H6*), 3.86 (s, 3H, C≡C-ArOCH_3), and 2.60 (s, 3H, ArCH_3).

^{13}C{^{1}H} NMR ($CDCl_3$, 126 MHz): δ 161.7, 161.5, 161.0, 157.0, 147.7, 142.9, 142.8, 138.7, 134.1, 126.1, 122.4, 121.2, 114.4, 114.2, 104.0, 82.4, 81.0, 80.6, 55.6, and 17.3.

HRMS (ESI-TOF) *m/z*: $[M+H^+]^+$ Calcd for $C_{22}H_{15}O_5^+$ 359.0914; Found 359.0903.

IR (neat): 3018, 2966, 2941, 2844, 2216, 1835, 1771, 1596, 1509, 1245 and 834.

mp: 218–224 °C.

(±)-5-(4-Methoxyphenyl)-4-((triisopropylsilyl)ethynyl)-6,9-dihydro-6,9-epoxynaphtho[1,2-*c*]furan-1,3-dione (27h)
and
(±)-4-((4-Methoxyphenyl)ethynyl)-5-(triisopropylsilyl)-6,9-dihydro-6,9-epoxynaphtho[1,2-*c*]furan-1,3-dione (28h)

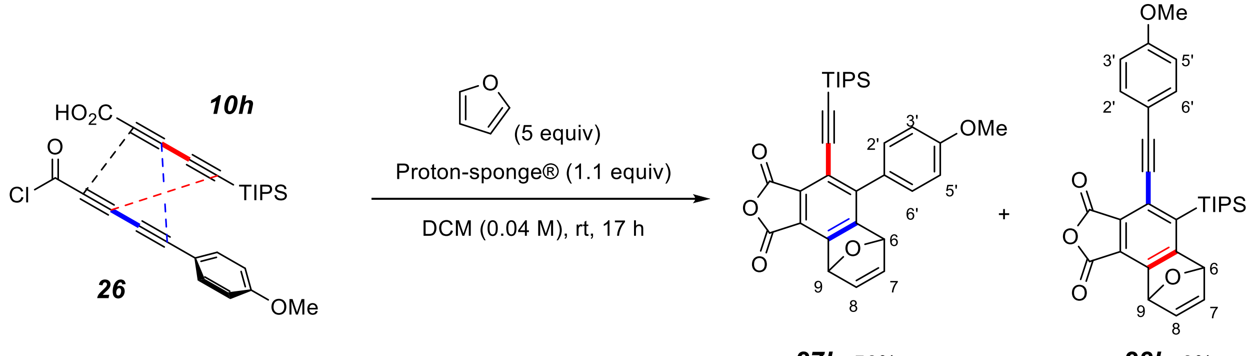

Following general procedure D, 5-(4-methoxyphenyl)penta-2,4-diynoyl chloride (**26**, 12.6 mg, 0.058 mmol, 1.1 equiv), furan (18.9 mL, 0.26 mmol, 5 equiv), and Proton-sponge® (12.3 mg, 0.058 mmol, 1.1 equiv) were added to a flame dried round-bottom flask and dissolved in dry DCM (0.7 mL). A solution of 5-(triisopropylsilyl)penta-2,4-diynoic acid (**10h**, 13.2 mg, 0.052 mmol, 1 equiv) in DCM (0.7 mL), which had been incubated on 3 Å molecular sieves overnight, was added to the mixture in one portion and stirred overnight. The solvent was evaporated *in vacuo* and the residue was purified by MPLC (4:1 hexanes:EtOAc) to afford, in order of elution, **28h** (2 mg, 0.004 mmol, 8%) as a bright yellow oil and **27h** (14.5 mg, 0.029 mmol, 56%) as a bright yellow oil.

Data for the phthalic anhydride derivative [first to elute] 28h:

^{1}H NMR ($CDCl_3$, 500 MHz): δ 7.58 (nfod, J_{app} = 8.7, 2H, *H2'/6'*), 7.11 (br d, *J* = 5.6 Hz, 1H, *H7* or *H8*), 7.10 (br d, *J* = 5.6 Hz, 1H, *H7* or *H8*), 6.94 (nfod, J_{app} = 8.7 Hz, 2H, *H3'/5'*), 6.25 (nfom, 1H, *H9*), 6.12 (nfom, 1H, *H6*), 3.85 (s, 3H, OC*H*$_3$), 1.95 (sept, *J* = 7.6 Hz, 3H, C5Si[C*H*(CH_3)$_2$]$_3$), 1.24 (d, *J* = 7.6 Hz, 9H, C5Si[CH(C*H*$_3$)(CH_3)']$_3$), 1.18 (d, *J* = 7.6 Hz, 9H, C5Si[CH(CH_3)(C*H*$_3$)']$_3$).

^{13}C{^{1}H} NMR ($CDCl_3$, 126 MHz): δ 166.5, 161.9, 161.6, 161.0, 146.4, 143.1, 142.4, 140.3, 133.5, 128.0, 126.5, 123.0, 114.6, 114.5, 102.5, 86.5, 83.7, 79.4, 55.6, 19.34, 19.31, 18.7, and 13.7.

HRMS (ESI-TOF) *m/z*: [M+H$^+$]$^+$ Calcd for $C_{30}H_{33}O_5Si^+$ 501.2092; Found 501.2080.

IR (neat): 2945, 2892, 2866, 2201, 1841, 1774, 1604, 1569, 1510, 1464, 1373, 1294, 1250, 1193, 1174, 1144, 1030, 1022, 979, 902, 885, 883, and 753 cm^{-1}.

Data for the phthalic anhydride derivative [second to elute] 27h:

^{1}H NMR ($CDCl_3$, 500 MHz): δ 7.29 (nfod, J_{app} = 8.2 Hz, 2H, *H2'/6'*), 7.15 (dd, *J* = 5.6, 1.9 Hz, 1H, *H7* or *H8*), 7.14 (dd, *J* = 5.6, 1.9 Hz, *H7* or *H8*), 7.00 (nfod, J_{app} = 8.8 Hz, *H3'/5'*), 6.28 (dd, *J* = 1.7, 0.9 Hz, 1H, *H9*), 5.64 (dd, *J* = 1.7, 0.9 Hz, 1H, *H6*), 3.87 (s, 3H, OC*H*$_3$), and 1.03-0.98 (overlapped m, 21H, C≡C-Si[CH(C*H*$_3$)$_2$]$_3$ and C≡C-Si[C*H*(CH_3)$_2$]$_3$).

$^{13}C\{^{1}H\}$ NMR ($CDCl_3$, 126 MHz): δ 161.6, 160.8, 160.4, 157.4, 148.7, 143.4, 143.3, 142.7, 130.6, 127.8, 127.6, 121.7, 120.7, 114.2, 107.2, 99.6, 81.9, 80.7, 55.6, 18.6, and 11.3.

HRMS (ESI-TOF) *m/z*: $[M+H^+]^+$ Calcd for $C_{30}H_{33}O_5Si^+$ 501.2092; Found 501.2082.

IR (neat): 2941, 2892, 2864, 1844, 1774, 1608, 1516, 1462, 1430, 1381, 1365, 1295, 1249, 1210, 1176, 1145, 1111, 1074, 1032, 996, 974, 949, 901, 882, 874, 835, 808, and 742 cm^{-1}.

(±)-4-Mesityl-5-(4-methoxyphenyl)-6,9-dihydro-6,9-epoxynaphtho[1,2-*c*]furan-1,3-dione (27i)

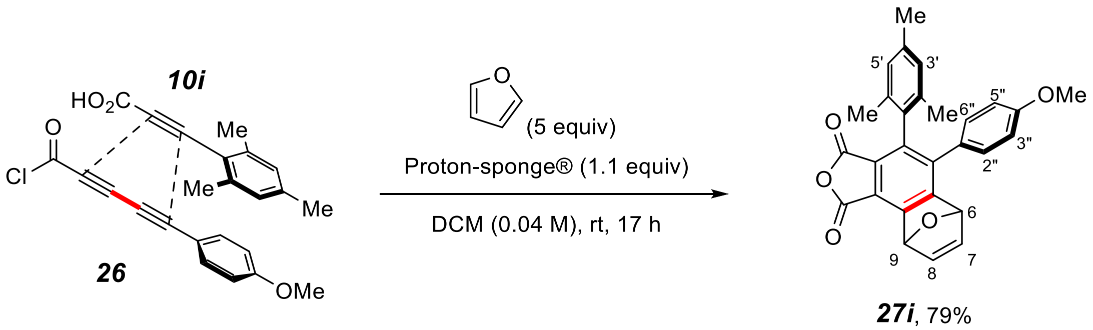

3-Mesitylpropiolic acid (**10i**) was prepared according to a literature procedure.[19]

Following general procedure D, 5-(4-methoxyphenyl)penta-2,4-diynoyl chloride (**26**, 13.3 mg, 0.061 mmol, 1.1 equiv), furan (20 µL, 0.28 mmol, 5 equiv), and Proton-sponge® (13 mg, 0.061 mmol, 1.1 equiv) were added to a flame dried round-bottom flask and dissolved in dry DCM (1.4 mL). To this, 3-mesitylpropiolic acid (**10i**, 10.3 mg, 0.055 mmol, 1.0 equiv) was added in one portion and stirred overnight. The solvent was evaporated *in vacuo* and the crude mixture was purified by MPLC (4:1 hexanes:EtOAc) to afford the sample of the phthalic anhydride derivative **27i** (19 mg, 0.043 mmol, 79%) as a bright red solid.

Data for the phthalic anhydride derivative 27i:

^{1}H NMR ($CDCl_3$, 500 MHz): δ 7.25 (dd, J = 5.5, 1.7 Hz, 1H, *H8*), 7.23 (dd, J = 5.5, 1.7 Hz, 1H, *H7*), 6.87 (dqqq, J = 1.8, 0.6, 0.6, 0.6 Hz, 1H, *H3'*), 6.82 (nfod, J_{app} = 8.8 Hz, 2H, *H2"* and *H6"*), 6.78 (nfod, J_{app} = 8.8 Hz, 2H, *H3"* and *H5"*), 6.71 (dqqq, J = 1.8, 0.6, 0.6, 0.6 Hz, 1H, *H3'*), 6.35 (dd, J = 1.8, 0.9 Hz, 1H, *H9*), 5.64 (dd, J = 1.8, 1.0 Hz, 1H, *H6*), 3.78 (s, 3H, ArOCH_3), 2.25 (dd, J = 0.7, 0.7 Hz, 3H, C4'CH_3), 2.00 (dd, J = 0.7, 0.7 Hz, 3H, C2'CH_3), and 1.66 (dd, J = 0.7, 0.7 Hz, 3H, C6'CH_3).

^{13}C{^{1}H} NMR ($CDCl_3$, 126 MHz): δ 162.1, 161.5, 159.7, 158.5, 148.8, 143.5, 143.0, 141.3, 140.1, 138.1, 135.9, 134.8, 130.4, 130.2, 128.6, 128.4, 127.8, 124.6, 121.7, 113.9, 82.4, 80.8, 55.4, 21.3, 20.6, and 20.4.

HRMS (ESI-TOF) *m/z*: $[M+H^+]^+$ Calcd for $C_{28}H_{23}O_5^+$ 439.1540; Found 439.1529.

IR (neat): 2953, 2921, 2854, 1838, 1769, 1608, 1515, 1249, 1176, 1027, 897, 874, 852, 725, 702, and 646.

mp: 85–90 °C.

(±)-4-(*tert*-Butyl)-5-(4-methoxyphenyl)-6,9-dihydro-6,9-epoxynaphtho[1,2-*c*]furan-1,3-dione (27j)

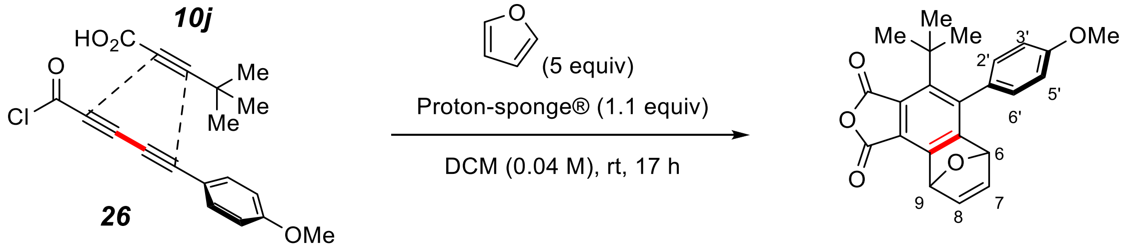

27j, 85%

4,4-Dimethylpent-2-ynoic acid (**10j**) was prepared according to a literature procedure.[20]

Following general procedure D, 5-(4-methoxyphenyl)penta-2,4-diynoyl chloride (**26**, 10 mg, 0.046 mmol, 1.1 equiv), furan (15.3 mL, 0.21 mmol, 5 equiv), and Proton-sponge® (9.8 mg, 0.046 mmol, 1.1 equiv) were added to a flame dried round-bottom flask and dissolved in dry DCM (1.1 mL). To this, 4,4-dimethylpent-2-ynoic acid (**10j**, 5.3 mg, 0.042 mmol, 1 equiv) was added in one portion at room temperature and stirred overnight. The solvent was evaporated *in vacuo* and the crude mixture was purified by MPLC (4:1 hexanes:EtOAc) to afford the phthalic anhydride derivative **27j** (13.4 mg, 0.036 mmol, 85%) as an off-white, crystalline solid.

Data for the phthalic anhydride derivative 27j:

^{1}H NMR ($CDCl_3$, 500 MHz): δ 7.21 (dd, *J* = 8.4, 2.2 Hz, 1H, *H2'* or *6'*), 7.10 (dd, *J* = 5.4, 1.9 Hz, 1H, *H8*), 7.00 (dd, *J* = 8.4, 2.7 Hz, 1H, *H3'* or *5'*), 6.95 (dd, *J* = 8.4, 2.7 Hz, 1H, *H5'* or *3'*), 6.91 (dd, *J* = 5.5, 1.9 Hz, 1H, *H7*), 6.85 (dd, *J* = 8.4, 2.3 Hz, 1H, *H6'* or *2'*), 6.30 (dd, *J* = 1.8, 0.8 Hz, 1H, *H9*), 5.20 (dd, *J* = 1.8, 0.8 Hz, 1H, *H6*), 3.89 (s, 3H, OCH_3), and 1.30 [s, 9H, $C(CH_3)_3$].

$^{13}C\{^1H\}$ NMR ($CDCl_3$, 126 MHz): δ 162.9, 162.4, 161.4, 159.5, 152.6, 146.2, 143.2, 143.0, 142.0, 132.2, 130.1, 129.6, 125.4, 123.9, 114.0, 113.9, 82.3, 80.9, 55.5, 38.4, and 32.5.

HRMS (ESI-TOF) *m/z*: $[M+H^+]^+$ Calcd for $C_{23}H_{21}O_5^+$ 377.1384; Found 377.1373.

IR (neat): 3007, 2960, 2914, 2872, 2837, 1840, 1822, 1775, 1607, 1511, 1483, 1469, 1283, 1246, 1226, 1155, 1122, 1110, 1029, 920, 878, 866, and 836.

mp: 170–173 °C.

Electrophile inversion experiment

To establish that this methodology for making the unsymmetric anhydride intermediate was also effective using the monoyne as the acid chloride, the following experiment was performed:

i)

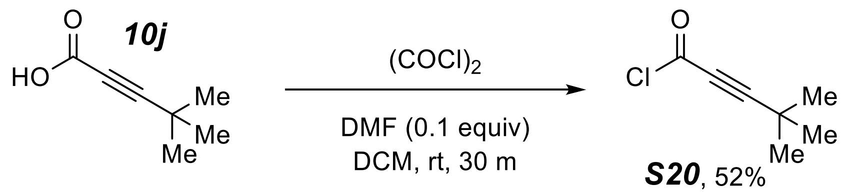

ii)

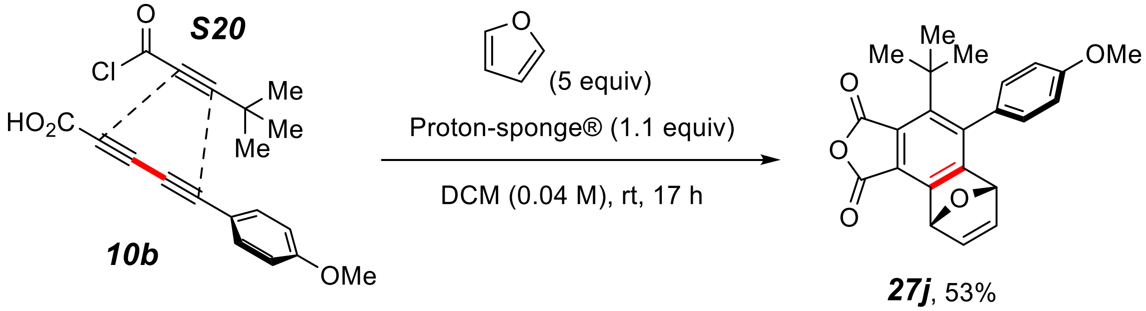

i) To a stirred solution of oxalyl chloride (207 µL, 0.75 mmol, 3 equiv) in DCM (2 mL) was added DMF (6 µL, 0.079 mmol, 0.1 equiv) at room temperature. Once bubbling of the solution had subsided (~1 minute), 4,4-dimethylpent-2-ynoic acid (**10j**,[20] 100 mg, 0.79 mmol, 1 equiv) was added in one portion and the mixture was stirred for 30 minutes. The resultant solution was filtered through a silica plug (100% hexanes) and the solvent was removed *in vacuo* using an ambient temperature water bath to afford 4,4-dimethylpent-2-ynoyl chloride (**S20**,[20] 59 mg, 0.41 mmol, 52%) as a clear liquid. A copy of its proton NMR spectrum has been provided.

ii) Following general procedure D, 4,4-dimethylpent-2-ynoyl chloride (**S20**, 8 mg, 0.055 mmol, 1.1 equiv), furan (18 µL, 0.25 mmol, 5 equiv), and Proton-sponge® (11.8 mg, 0.055 mmol, 1.1 equiv) were added to a flame dried round-bottom flask and dissolved in dry DCM (1.3 mL). To this, 5-(4-methoxyphenyl)penta-2,4-diynoic acid (**10b**, 10 mg, 0.05 mmol, 1 equiv) was added in one portion at room temperature and stirred overnight. The solvent was evaporated *in vacuo* and the crude mixture was purified by MPLC (4:1 hexanes:EtOAc) to afford phthalic anhydride derivative **27j** (10 mg, 0.027 mmol, 53%) as an off-white, crystalline solid.

(±)-5-(4-Methoxyphenyl)-6,9-dihydro-6,9-epoxynaphtho[1,2-*c*]furan-1,3-dione (27k)

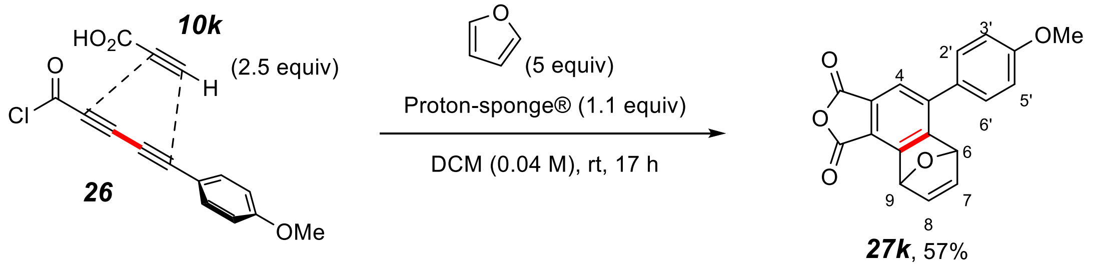

Following general procedure D, 5-(4-methoxyphenyl)penta-2,4-diynoyl chloride (**26**, 15 mg, 0.069 mmol, 1 equiv), furan (25 µL, 0.345 mmol, 5 equiv), and Proton-sponge® (16.3 mg, 0.069 mmol, 1.1 equiv) were added to a flame dried round-bottom flask and dissolved in dry DCM (1.7 mL). To this solution, propiolic acid (**10k**, 10.7 µL, 0.172 mmol, 2.5 equiv) was added in one portion at room temperature and the resulting solution was stirred overnight. The solvent was evaporated *in vacuo* and the crude mixture was purified by MPLC (4:1 hexanes:EtOAc) to afford the phthalic anhydride derivative **27k** (12.5 mg, 0.039 mmol, 57%) as a white, crystalline solid.

Data for the phthalic anhydride derivative 27k:

^{1}H NMR ($CDCl_3$, 500 MHz): δ 7.76 (s, 1H, *H4*), 7.32 (nfod, J_{app} = 8.6 Hz, 2H, *H2′/6′*), 7.27 (dd, *J* = 5.4, 1.8 Hz, 1H, *H7* or *H8*), 7.22 (dd, *J* = 5.4, 1.8 Hz, 1H, *H7* or *H8*), 7.08 (nfod, J_{app} = 8.6 Hz, 2H, *H3′/5′*), 6.30 (nfom, 1H, *H9*), 5.93 (nfom, 1H, *H6*), and 3.90 (s, 3H, OCH_3).

^{13}C{^{1}H} NMR ($CDCl_3$, 126 MHz): δ 162.9, 162.1, 160.7, 156.7, 150.9, 143.4, 143.1, 141.0, 129.5, 129.3, 127.8, 124.1, 120.8, 115.0, 81.9, 80.5, and 55.7.

HRMS (ESI-TOF) *m/z*: $[M+H^+]^+$ Calcd for $C_{19}H_{13}O_5^+$ 321.0757; Found 321.0750.

IR (neat): 3585, 3067, 3011, 2962, 2938, 2916, 2838, 1830, 1762, 1605, 1519, 1454, 1409, 1294, 1262, 1241, 1233, 1182, 1169, 1148, 1124, 1028, 963, 926, 876, 836, and 827.

mp: 274–277 °C.

5-(4-Methoxyphenyl)penta-2,4-diyn-1-yl propiolate (29b)

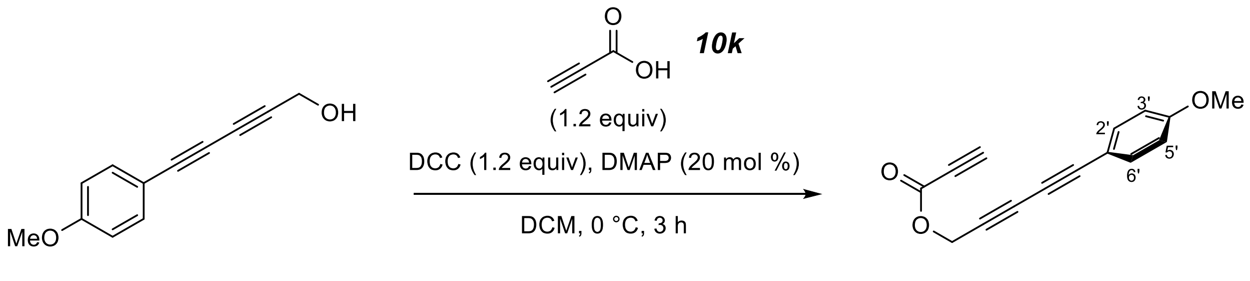

S21 **29b**, 94%

5-(4-Methoxyphenyl)penta-2,4-diyn-1-ol (**S21**) was prepared according to a literature procedure and its proton NMR spectrum has been provided.[21]

An oven-dried 50 mL round-bottom flask was evacuated and backfilled with N_2 three times. The flask was charged with diynol (**S21**, 100 mg, 0.537 mmol, 1 equiv), DCC (133 mg, 0.645 mmol, 1.2 equiv), DMAP (13 mg, 0.11 mmol, 0.2 equiv), and DCM (5 mL) at 0 °C. A solution of propiolic acid (**10k**, 48 mg, 0.648 mmol, 1.2 equiv) in DCM (3 mL) was added slowly to the flask and the resulting, initially homogenous solution was allowed to stir at 0 °C for 3 h. The suspension was directly loaded onto a silica gel column and purified by flash chromatography (3:1 Hex:EtOAc) to afford **29b** (121 mg, 0.508 mmol, 94%) as a white crystalline solid that became brown over time with no significant change in spectral purity.

Data for the triyne 29b:

^{1}H NMR ($CDCl_3$, 500 MHz): δ 7.44 (nfod, J_{app} = 8.8 Hz, 2H, *H2′* and *H6′*), 6.84 (nfod, J_{app} = 8.8 Hz, 2H, *H3′* and *H5′*), 4.92 (s, 2H, C*H*$_2$), 3.82 (s, 3H, OC*H*$_3$), and 2.96 (s, 1H, C≡C-*H*).

^{13}C{^{1}H} NMR ($CDCl_3$, 126 MHz): δ 160.8, 151.9, 134.5, 114.3, 113.0, 79.9, 76.2, 74.1, 74.0, 72.7, 71.9, 55.5, and 54.3.

HRMS (ESI-TOF) *m/z*: [M+H$^+$]$^+$ Calcd for $C_{15}H_{11}O_3^+$ 239.0703; Found 239.0708.

IR (neat): 3280, 3008, 2936, 2840, 2246, 2224, 2121, 1718, 1602, 1509, 1250, 1203, 1174, and 832 cm^{-1}.

mp: 51–53 °C.

Prop-2-yn-1-yl 5-(4-methoxyphenyl)penta-2,4-diynoate (29c)

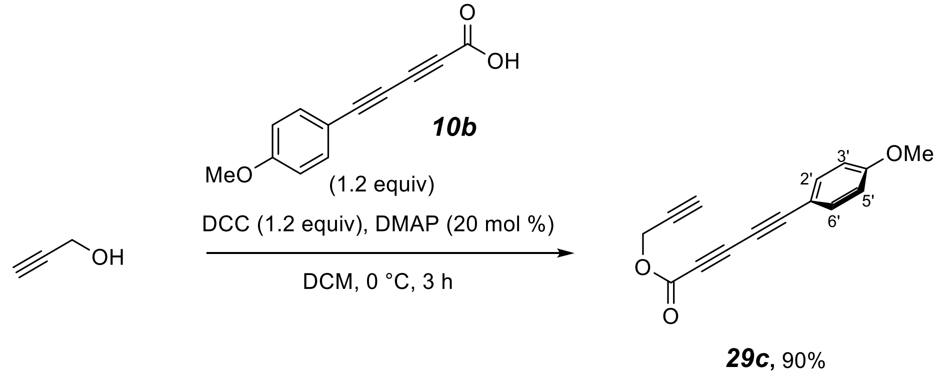

An oven-dried 50 mL round-bottom flask was evacuated and backfilled with N_2 three times. The flask was charged with propargyl alcohol (23 mg, 0.41 mmol, 1 equiv), DCC (103 mg, 0.499 mmol, 1.2 equiv), DMAP (10 mg, 0.082 mmol, 0.2 equiv), and DCM (5 mL) at 0 °C. A solution of 5-(4-methoxyphenyl)penta-2,4-diynoic acid (**10b**, 100 mg, 0.500 mmol, 1.2 equiv) in DCM (3 mL) was added slowly to the flask and the resulting, initially homogenous solution was allowed to stir at 0 °C for 3 h. The suspension was directly loaded onto a silica gel column and purified by flash chromatography (3:1 Hex:EtOAc) to afford **29c** (88 mg, 0.37 mmol, 90%) as an orange crystalline solid.

Data for the triyne 29c:

^{1}H NMR ($CDCl_3$, 500 MHz): δ 7.50 (nfod, J_{app} = 8.8 Hz, 2H, *H2′* and *H6′*), 6.87 (nfod, J_{app} = 8.8 Hz, 2H, *H3′* and *H5′*), 4.79 (d, *J* = 2.5 Hz, 2H, C*H*$_2$), 3.84 (s, 3H, OC*H*$_3$), and 2.54 (t, *J* = 2.5 Hz, 1H, C≡C-*H*).

^{13}C{^{1}H} NMR ($CDCl_3$, 126 MHz): δ 161.6, 152.4, 135.2, 114.6, 111.7, 85.1, 76.6, 76.1, 73.3, 71.3, 70.9, 55.6, and 53.6.

HRMS (ESI-TOF) *m/z*: $[M+H^+]^+$ Calcd for $C_{15}H_{11}O_3^+$ 239.0703; Found 239.0708.

IR (neat): 3290, 3002, 2958, 2932, 2845, 2219, 1705, 1603, 1372, 1200, 1030, and 828 cm^{-1}.

mp: 78–80 °C.

1-Methoxy-4-(5-(prop-2-yn-1-yloxy)penta-1,3-diyn-1-yl)benzene (29d)

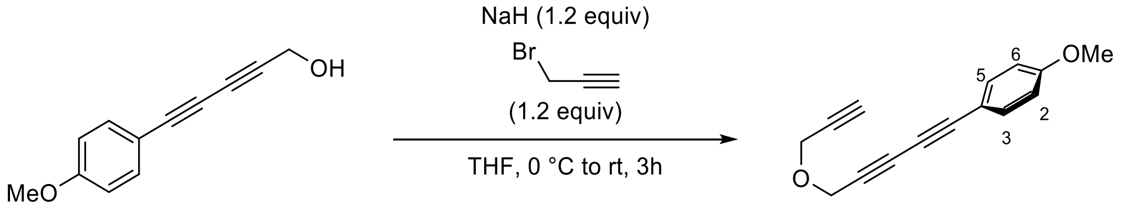

S21 ***29d,*** 73%

An oven-dried 25 mL round-bottom flask was evacuated and backfilled with N_2 three times. The flask was charged with the diynol (**S21**, 200 mg, 1.07 mmol, 1 equiv) and dry THF (10 mL) at 0 °C. To this stirred solution was added NaH in 60% mineral oil (52 mg, 1.29 mmol, 1.2 equiv) and the mixture was allowed to warm to ambient temperature. After 0.5 h, propargyl bromide (80% in toluene, 0.14 mL, 1.29 mmol, 1.2 equiv) was added in one portion and the mixture was stirred for an additional 2 h. Upon reaction completion as monitored by TLC, the solution was diluted with Et_2O (15 mL) and quenched by the addition of saturated aqueous $NaHCO_3$ solution (15 mL). The aqueous layer was extracted with Et_2O (2 x 15 mL) and the combined organics were washed with brine (15 mL), dried with $MgSO_4$, and concentrated in vacuo. The crude mixture was purified by MPLC (19:1 Hex:EtOAc) to afford **29d** (176 mg, 0.79 mmol, 73%) as a light yellow crystalline solid.

Data for the triyne 29d:

^{1}H NMR ($CDCl_3$, 500 MHz): δ 7.44 (nfod, J_{app} = 8.8 Hz, 2H, *H3* and *H5*), 6.84 (nfod, J_{app} = 8.8 Hz, 2H, *H2* and *H6*), 4.42 (s, 2H, C≡C-C≡CCH_2), 4.30 (d, *J* = 2.4 Hz, 2H, HC≡CCH_2), 3.82 (s, 3H, OCH_3), and 2.47 (t, *J* = 2.4 Hz, 1H, C≡C-*H*).

^{13}C{^{1}H} NMR ($CDCl_3$, 126 MHz): δ 160.7, 134.5, 134.4, 114.3, 113.3, 78.8, 78.7, 77.2, 75.4, 72.2, 72.0, 57.3, 56.7, and 55.5.

HRMS (ESI-TOF) *m/z*: $[M+H^+]^+$ Calcd for $C_{15}H_{13}O_2^+$ 225.0910; Found 225.0904.

IR (neat): 3287, 3070, 3010, 2962, 2935, 2889, 2840, 2242, 2217, 1600, 1568, 1506, 1439, 1357, 1290, 1249, 1173, 1071, 1023, 941, 822, and 632 cm^{-1}.

mp: 34–37 °C.

(±)-5-(4-Methoxyphenyl)-6,9-dihydro-6,9-epoxynaphtho[1,2-*c*]furan-3(1*H*)-one (30b)

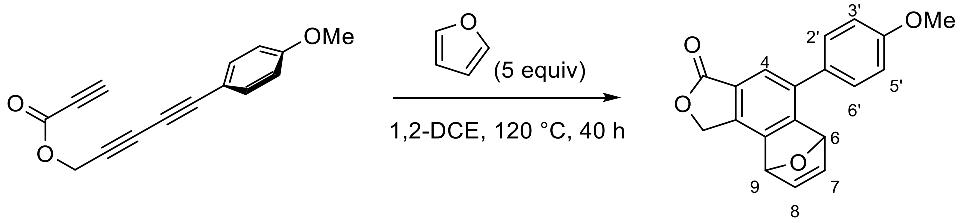

29b ***30b***, 93%

The ester triyne (**29b**, 20 mg, 0.084 mmol, 1 equiv) was placed in a screw-capped culture tube. 1,2-Dichloroethane (1.7 mL) was added, followed by furan (29 mg, 0.43 mmol, 5 equiv). The solution was heated to 120 °C for 40 h. The crude reaction mixture was then passed through a silica gel plug, the elution solvent was removed, and the residue was purified by MPLC (1:1 Hex:EtOAc) to afford **30b** (24 mg, 0.078 mmol, 93%) as a white crystalline solid.

Data for the phthalide 30b:

^{1}H NMR ($CDCl_3$, 500 MHz): δ 7.69 (s, 1H, *H4*), 7.29 (nfod, J_{app} = 8.7 Hz, 2H, *H2′* and *H6′*), 7.25 (dd, *J* = 5.5, 1.9 Hz, 1H, *H8*), 7.17 (dd, *J* = 5.5, 1.9 Hz, 1H, *H7*), 7.03 (nfod, J_{app} = 8.7 Hz, 2H, *H3′* and *H5′*), 5.89 (dd, *J* = 1.9, 0.9 Hz, 1H, *H9*), 5.87 (dd, *J* = 1.9, 0.9 Hz, 1H, *H6*), 5.41 (d, *J* = 14.8 Hz 1H, CH_a), 5.29 (d, *J* = 14.8 Hz 1H, CH_b), and 3.88 (s, 3H, OCH_3).

^{13}C{^{1}H} NMR ($CDCl_3$, 126 MHz): δ 170.6, 159.8, 154.2, 144.0, 143.3, 143.0, 135.9, 135.0, 130.5, 129.3, 123.8, 123.7, 114.6, 82.1, 80.5, 68.3, and 55.5.

HRMS (ESI-TOF) *m/z*: [M+H$^+$]$^+$ Calcd for $C_{19}H_{15}O_4^+$ 307.0965; Found 307.0971.

IR (neat): 3014, 2959, 2937, 2838, 1755, 1518, 1251, 1024, and 830 cm^{-1}.

mp: 203–205 °C.

(±)-5-(4-Methoxyphenyl)-6,9-dihydro-6,9-epoxynaphtho[1,2-*c*]furan-1(3*H*)-one (30c)

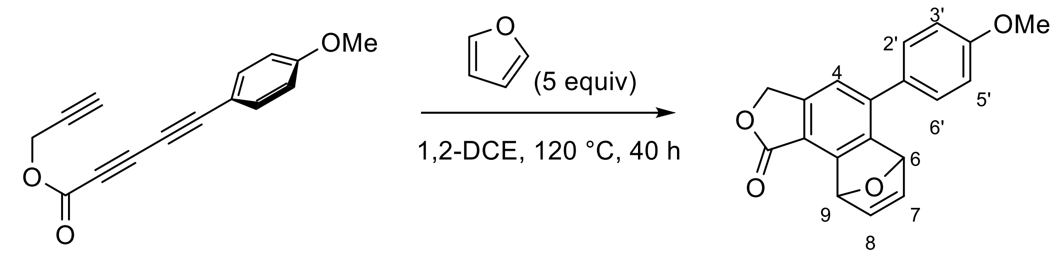

29c ***30c***, 82%

The ester triyne (**29c**, 20 mg, 0.084 mmol, 1 equiv) was placed in a screw-capped culture tube. 1,2-Dichloroethane (1.7 mL) was added, followed by furan (29 mg, 0.43 mmol, 5 equiv). The solution was heated at 120 °C for 40 h. The crude reaction mixture was then passed through a silica gel plug, the elution solvent was removed, and the residue was purified by MPLC (1:1 Hex:EtOAc) to afford **30c** (21 mg, 0.069 mmol, 82%) as a white crystalline solid.

Data for the phthalide 30c:

^{1}H NMR ($CDCl_3$, 500 MHz): δ 7.31 (nfod, J_{app} = 8.7 Hz, 2H, *H2'* and *H6'*), 7.24 (dd, *J* = 5.5, 1.9 Hz, 1H, *H8*), 7.19 (dd, *J* = 5.5, 1.9 Hz, 1H, *H7*), 7.69 (dd, *J* = 1.1, 1.1 Hz, 1H, *H4*), 7.04 (nfod, J_{app} = 8.7 Hz, 2H, *H3'* and *H5'*), 6.37 (dd, *J* = 2.0, 0.9 Hz, 1H, *H9*), 5.88 (dd, *J* = 2.0, 0.9 Hz, 1H, *H6*), 5.37 (dd, *J* = 14.9, 1.1 Hz 1H, CH_a), 5.33 (dd, *J* = 14.9, 1.1 Hz 1H, CH_b), and 3.89 (s, 3H, OCH_3).

^{13}C{^{1}H} NMR ($CDCl_3$, 126 MHz): δ 169.9, 160.1, 150.4, 149.9, 143.9, 143.3, 142.6, 139.6, 130.9, 129.5, 118.2, 117.8, 114.7, 81.8, 80.5, 70.4, and 55.6.

HRMS (ESI-TOF) *m/z*: [M+H$^+$]$^+$ Calcd for $C_{19}H_{15}O_4^+$ 307.0965; Found 307.0969.

IR (neat): 3022, 2934, 2840, 1758, 1641, 1608, 1252, and 831 cm^{-1}.

mp: 184–187 °C.

5-(4-Methoxyphenyl)-1,3-dihydroisobenzofuran (30d)

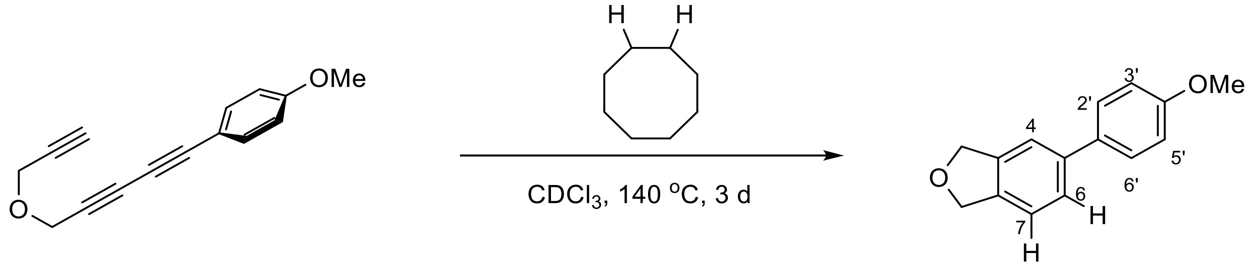

29d ***30d***, 40%

The ether triyne (**29d**, 10 mg, 0.045 mmol, 1 equiv) was placed in a screw-capped culture tube and dissolved in $CDCl_3$ (50 µL). Cyclooctane (1 mL) was added to the solution as a cosolvent The solution was heated at 140 °C for 3 days. The crude reaction mixture was then passed through a silica gel plug, the elution solvent was removed, and the residue was purified by MPLC (9:1 Hex:EtOAc) to afford **30d** (4 mg, 0.018 mmol, 40%) as a white crystalline solid.

Data for the isobenzofuran 30d:

^{1}H NMR ($CDCl_3$, 500 MHz): δ 7.51 (nfod, J_{app} = 8.8 Hz, 2H, *H2'* and *H6'*), 7.45 (ddd, J = 7.8, 2.4, 0.7 Hz, 1H, *H6*), 7.40 (dd, J = 1.6, 0.8 Hz, 1H, *H4*), 7.28 (dd, J = 7.8, 0.8 Hz, 1H, *H7*), 6.98 (nfod, J_{app} = 8.8 Hz, 2H, *H3'* and *H5'*), 5.16 (nfom, 2H, *H1* or *H3*), 5.15 (nfom, 2H, *H1* or *H3*), and 3.86 (s, 3H, OCH_3).

^{13}C{^{1}H} NMR ($CDCl_3$, 126 MHz): δ 159.4, 140.6, 140.07, 137.7, 133.7, 128.4, 126.3, 121.3, 119.4, 114.4, 73.72, 73.59, and 55.52.

HRMS (ESI-TOF) *m/z*: $[M+H^+]^+$ Calcd for $C_{15}H_{15}O_2^+$ 227.1067; Found 227.1060.

IR (neat): 3033, 2999, 2933, 2903, 2836, 1607, 1517. 1486, 1463, 1439, 1360, 1290, 1244, 1179, 1038, 1022, 901, 889, 839, 813, 797, 705, 587, 578, 537 and 526 cm^{-1}.

mp: 64–66 °C.

4-Phenyl-2-(p-tolyl)-1H-benzo[f]isoindole-1,3(2H)-dione (42)

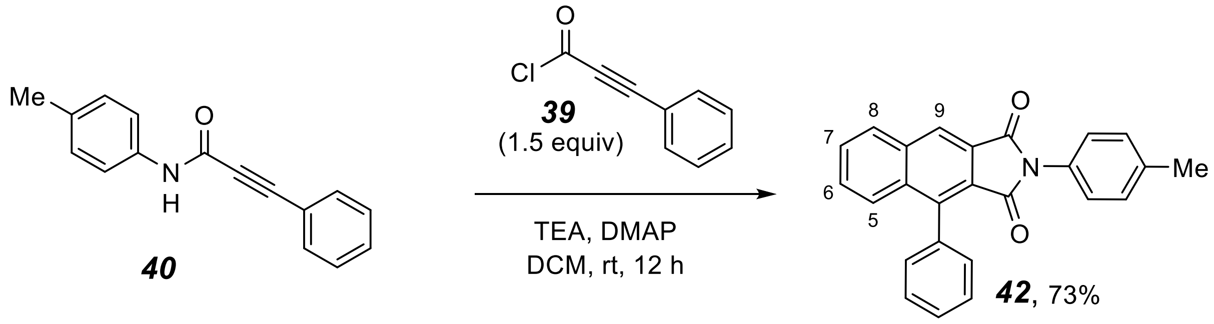

3-Phenyl-*N*-(p-tolyl)propiolamide[22] (**40**, 25 mg, 0.106 mmol, 1 equiv) and 3-phenylpropioloyl chloride (**39**) [23] (26 mg, 0.159 mmol, 1.5 equiv) were prepared according to literature procedures.

The propiolamide **40**, triethylamine (30 µL, 0.21 mmol, 2 equiv), and DMAP (1.3 mg, 0.01 mmol, 0.1 equiv) were added to a flame-dried, round-bottom flask and dissolved in DCM (3 mL). A DCM solution (2 mL) of 3-phenylpropiolyl chloride (**39**) was added to the mixture dropwise at room temperature and the resulting mixture was allowed to stir overnight. The DCM was removed and the resulting residue was purified by MPLC (4:1 Hex:EtOAc) to afford the naphthalimide derivative **42**[24] (28 mg, 0.077 mmol, 73%) as a white crystalline solid.

Data for the naphthalimide 42:

^{1}H NMR ($CDCl_3$, 500 MHz): δ 8.48 (d, *J* = 0.8 Hz, 1H, *H9*), 8.12 (ddd, *J* = 8.1, 2.0, 0.8 Hz, 1H, *H8*), 7.85 (ddd, *J* = 8.5, 2.0, 0.9 Hz, 1H, *H5*), 7.71 (ddd, *J* = 8.1, 6.9, 1.3 Hz, 1H, *H7*) 7.62 (ddd, *J* = 8.5, 6.9, 1.4 Hz, 1H, *H6*), 7.56–7.51 (m, 3H, ArPhH_m and ArPhH_p), 7.43 (nfodd, J_{app} = 7.7 Hz, 2H, ArPhH_o), 7.31 (nfod, J_{app} = 8.5 Hz, 2H, -NArH_o), 7.25 (nfod, J_{app} = 8.5 Hz, 2H, -NArH_m), and 2.37 (t, *J* = 0.8 Hz, 3H, NArCH_3).

^{13}C{^{1}H} NMR ($CDCl_3$, 126 MHz): δ 167.1, 166.6, 140.9, 138.2, 135.83, 135.79, 134.5, 132.7, 130.5, 130.0, 129.7, 129.3, 129.2, 128.8, 128.7, 128.3, 127.7, 126.6, 124.8, 123.6, and 21.3.

IR (neat): 3056, 3036, 1764, 1716, 1682, 1607, 1511, 1443, 1361, 1205, 1179, 1116, 1097, 943, 909, and 764 cm^{-1}.

mp: 206–208 °C.[24]

IV. Kinetics Data for Compounds 29a-d

Kinetics Data for Cyclization of the Anhydride Derived from Precursors 10k and 26

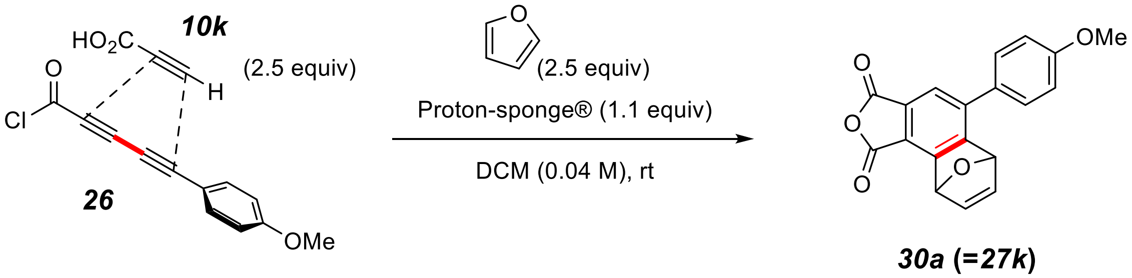

The diynoic acid chloride (**26**, 5 mg, 0.023 mmol, 1 equiv), Proton-sponge® (5.4 mg, 0.025 mmol, 1.1 equiv), and furan (4.2 μL, 0.058 mmol, 2.5 equiv) were dissolved in deuterochloroform (600 μL) in a vial and transferred to an NMR tube. Propiolic acid (3.6 μL, 0.058 mmol, 2.5 equiv) was added directly to the NMR tube, and the reaction progress was monitored by periodically taking a ^{1}H NMR spectrum of the reaction mixture. A sufficient number of spectra were obtained to deduce the experimental half-life at ambient temperature.

The following scheme shows the observable species in the reaction. These are the **acid chloride** **26**, the **anhydride** **29a**, and the **product** **30a.** Their appearance and disappearance over time was modeled as a combination of two elementary reaction steps for the purposes of kinetic analysis. First, the acid chloride reacts with propiolic acid to form the intermediate anhydride. The anhydride then cyclizes to the benzyne, which is then rapidly trapped by furan to give the final benzenoid product. The trapping step need not be considered in the rate law because it is much faster than the previous two elementary steps. A stack of ^{1}H NMR spectra showing the relative concentrations of these species over the course of the reaction is provided in Figure S2.

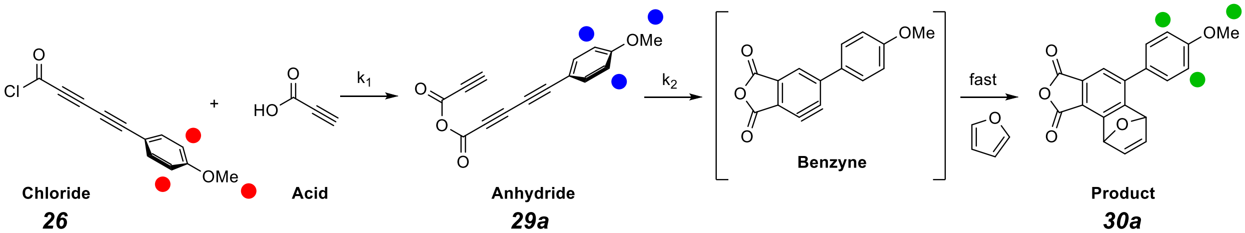

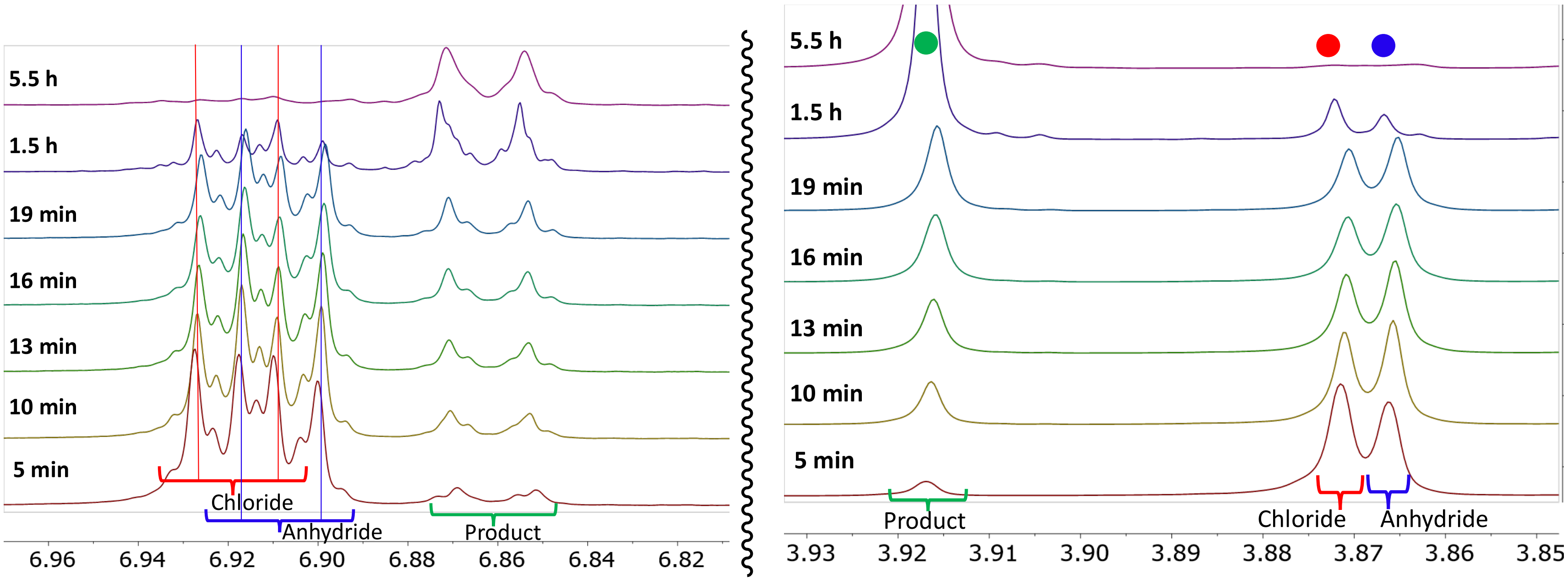

Figure S2. Representative portions of the ^{1}H NMR spectra of the condensation to form and cycloisomerization of the **anhydride 29a**, derived from the **acid chloride 26**, from which the half-life was determined by monitoring the appearance of the **product 30a** formed by in situ (and very rapid) furan trapping of the intermediate benzyne. The resonances in the 6.84–6.94 ppm range are of the protons ortho to the methoxy group in each of the distinct products. They appear as non-first-order doublets due to magnetic inequivalence [see 5-HMR-15 The AA'BB' Pattern" in the Hans Reich series of NMR resources maintained by the Division of Organic Chemistry of the American Chemical Society: https://organicchemistrydata.org/hansreich/resources/nmr/?page=05-hmr-15-aabb%2F (accessed 2-24-24)]. The overlapped resonances for the chloride and anhydride were challenging to quantify, so we used the methoxy singlet resonances (3.86–3.92 ppm) to measure the relative amounts of **acid chloride 26**, the **anhydride 29a**, and the **product 30a** over time.

Because NMR analysis (Figure S2) showed that the rates of formation and cycloisomerization of the anhydride are comparable (and we presume that first step is irreversible), we could not apply a steady-state or rapid pre-equilibrium approach to solve for the half-life of the anhydride's cyclization to benzyne. Thus, we opted to model this reaction using COPASI.[25]

The following rate laws represent the elementary steps that were entered into the COPASI software:

$\frac{d[Anhydride]}{dt} = k_1[Chloride][Acid]$ Irreversible, bimolecular formation of anhydride

$\frac{d[Benzyne]}{dt} = k_2[Anhydride]$ Irreversible, unimolecular cycloisomerization to benzyne

The following optimized values for the kinetic parameters were used in COPASI to generate the plot shown in Figure S3:

$$k_1 = 0.0111 \frac{l}{mol * s}$$

$$k_2 = 0.00130\, s^{-1}$$

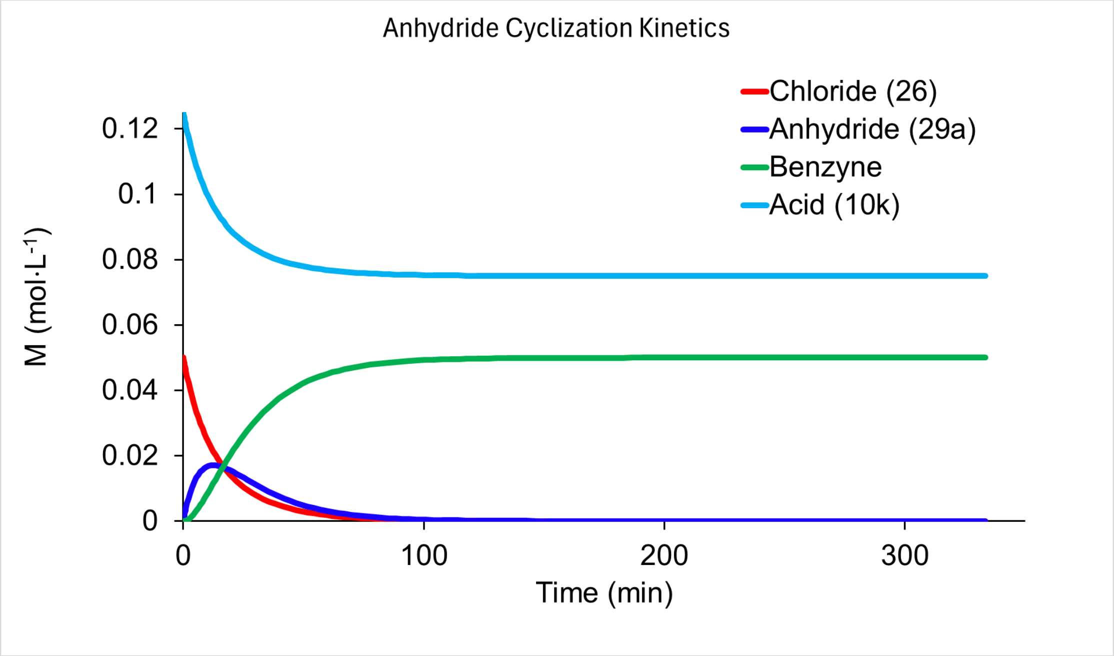

Figure S3. COPASI simulations using the equations given above with the optimal kinetic parameters. The simulation assumes an initial concentration of 0.05 M for the stoichiometrically limiting reagent.

The COPASI analysis of the data gives a half-life of 533 seconds (8.9 minutes) for the cycloisomerization of the anhydride-containing triyne to form the benzyne. The benzyne is, of course, not directly observed because it is trapped rapidly by the furan. That is, the rate of formation of the product **30a** serves as a proxy for the rate of benzyne formation.

Kinetics Data for HDDA Cyclization of Ester Triyne 29b

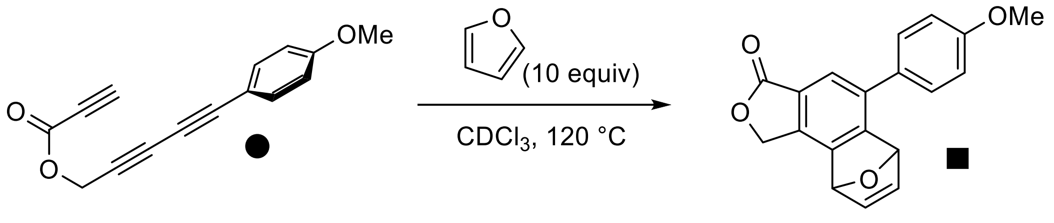

The ester-containing triyne (**29b**, 10 mg, 0.042 mmol, 1 equiv) was placed in a screw-capped culture tube. Deuterochloroform (1.0 mL) was added, followed by furan (29 mg, 0.43 mmol, 10 equiv). The solution was heated to 120 °C. The solution was periodically removed from the heating block, cooled, and the ^{1}H NMR spectrum recorded. A sufficient number of spectra were obtained to deduce the experimental half-life.

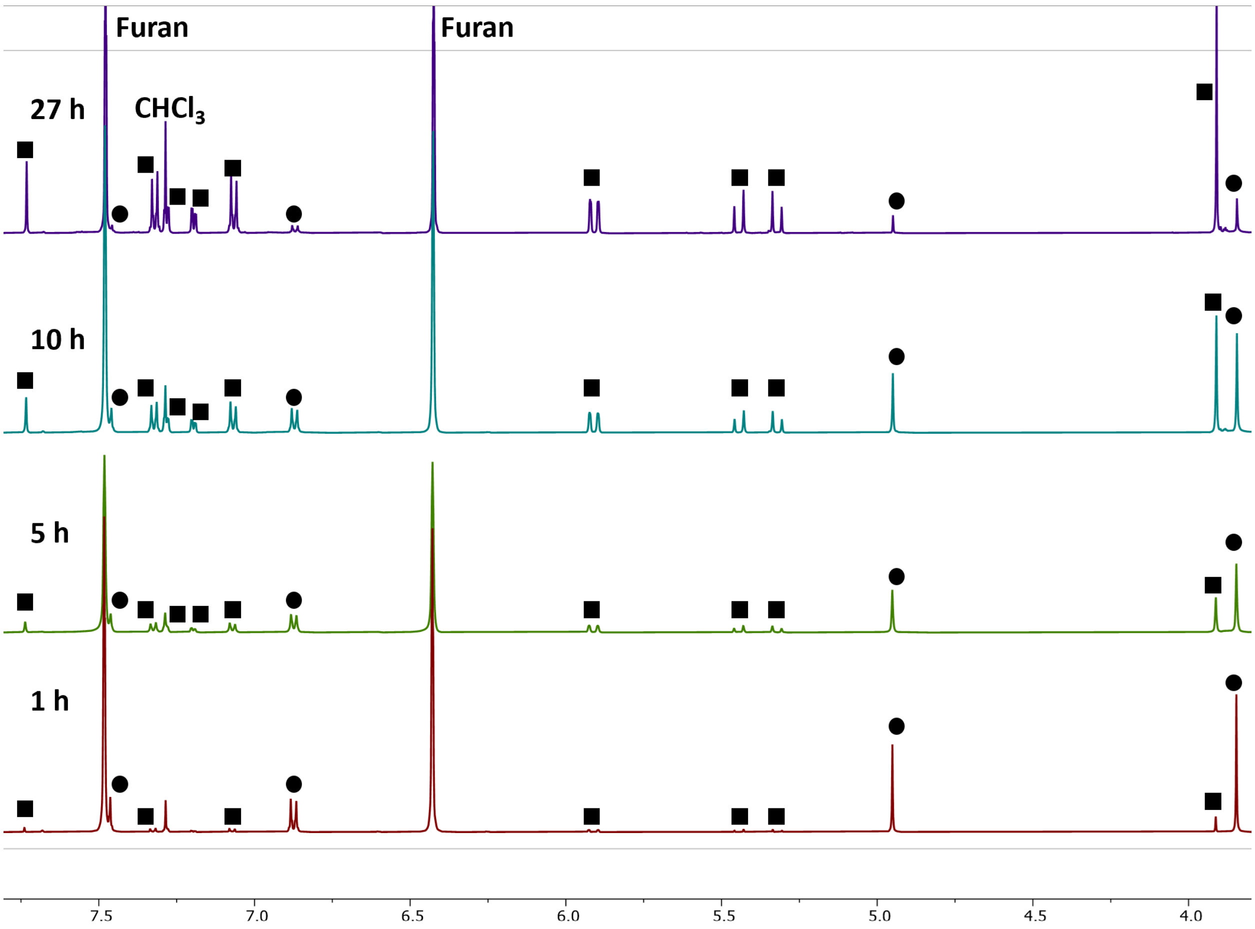

Figure S4. ^{1}H NMR spectra of the cycloisomerization of ester **29b** from which the half-life was determined via in situ (and rapid) furan trapping of the intermediate benzyne.

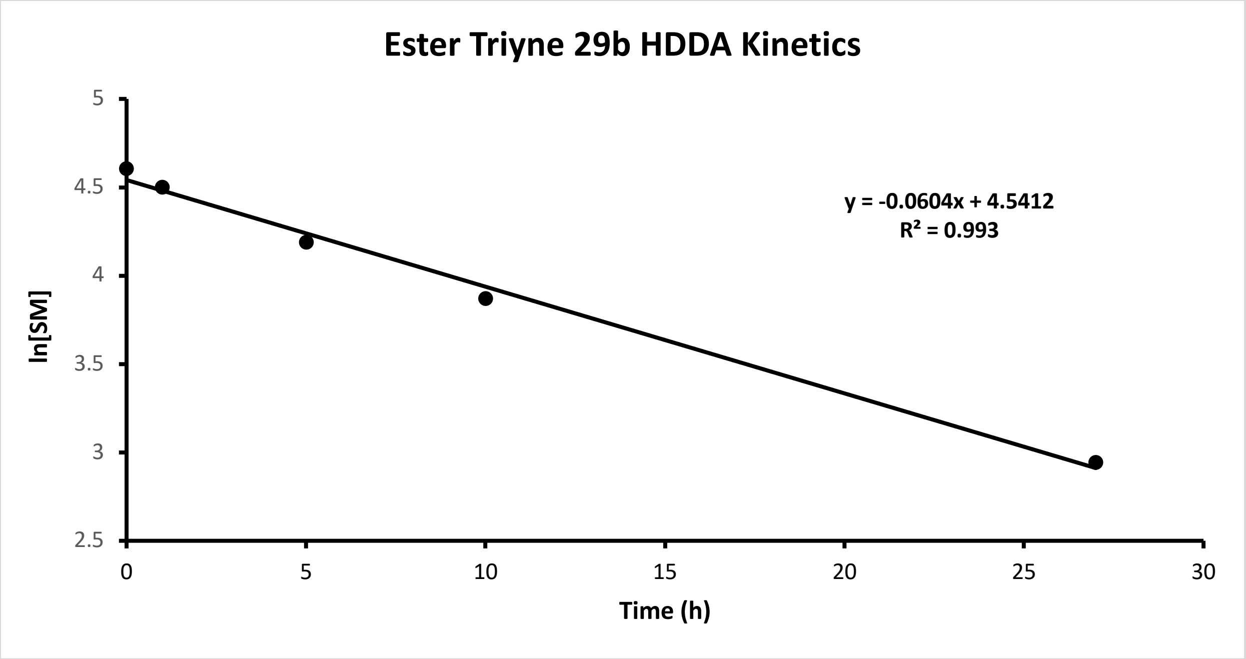

Figure S5. Fit of the above data to the first-order integrated rate law. The linear fit suggests a half-life for the cycloisomerization of **29b** of 10 h 25 min at 120 °C.

Kinetics Data for HDDA Cyclization of Ester Triyne 29c

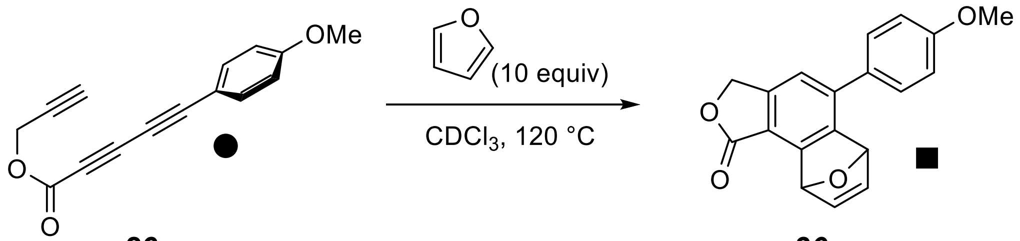

29c ***30c***

The ester-containing triyne (**29c**, 10 mg, 0.042 mmol, 1 equiv) was placed in a screw-capped culture tube. Deuterochloroform (1.0 mL) was added, followed by furan (29 mg, 0.43 mmol, 10 equiv). The solution was heated to 120 °C. The solution was periodically removed from the heating block, cooled, and the ^{1}H NMR spectrum recorded. A sufficient number of spectra were obtained to deduce the experimental half-life.

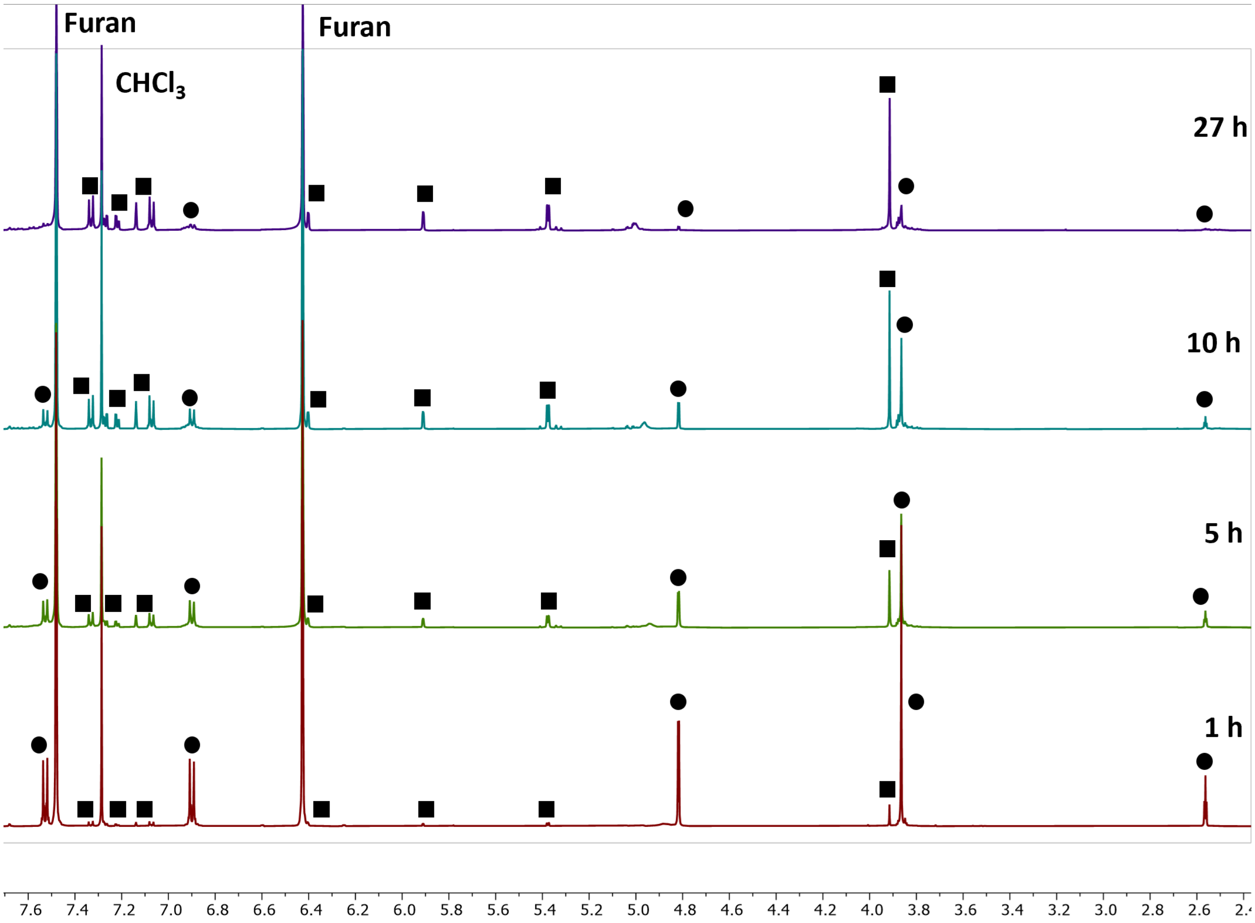

Figure S6. ^{1}H NMR spectra of the cycloisomerization of ester **29c** from which the half-life was determined via in situ (and rapid) furan trapping of the intermediate benzyne.

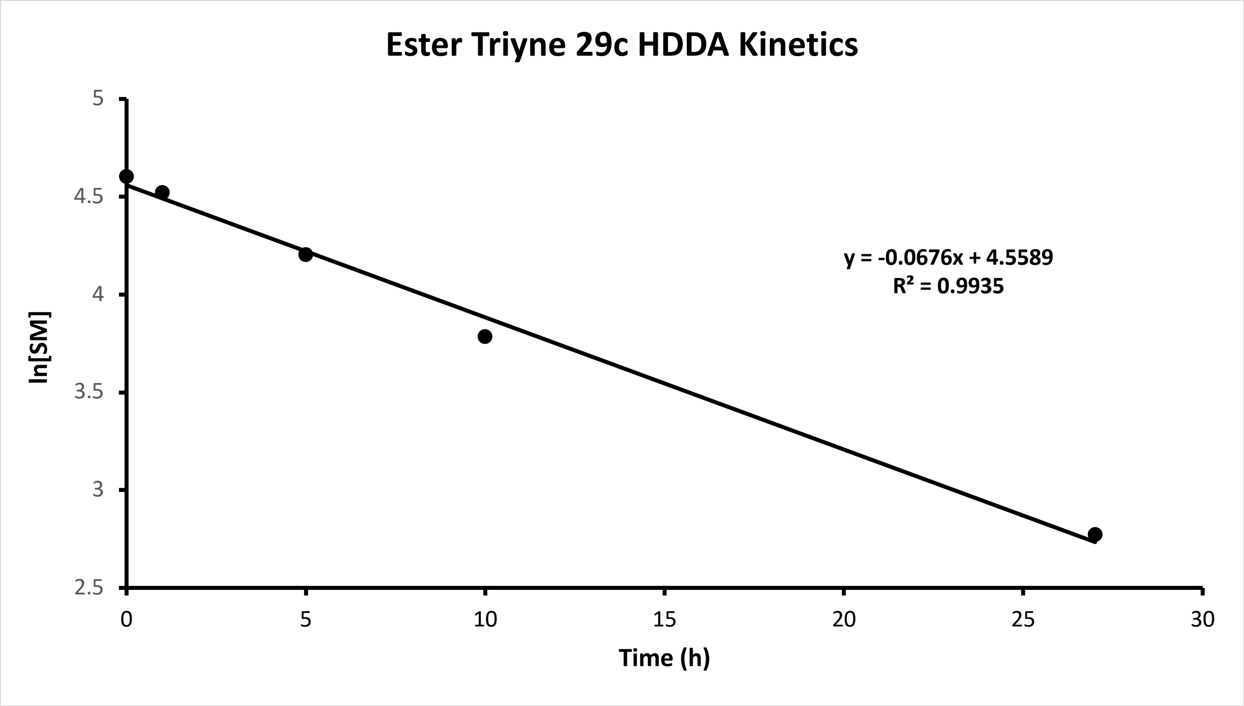

Figure S7. Fit of the above data to the first-order integrated rate law. The linear fit suggests a half-life for the cycloisomerization of **29c** of 9 h 35 min at 120 °C.

Kinetics Data for HDDA Cyclization of Ether Triyne 29d

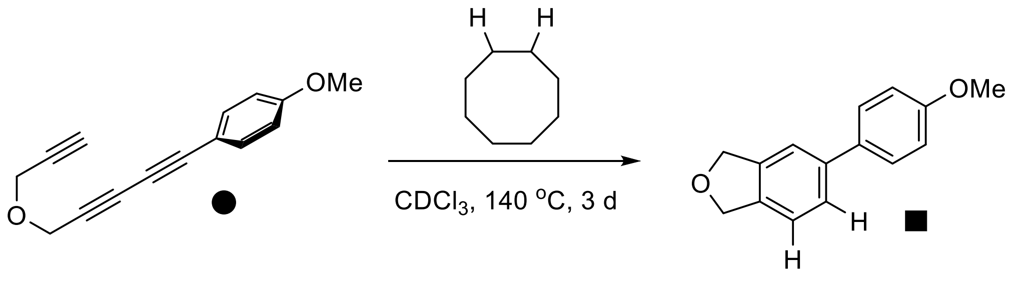

The ether-containing triyne (**29d**, 15 mg, 0.067 mmol, 1 equiv) was placed in a screw-capped culture tube. Deuterochloroform (50 µL) was added to aid in dissolution, followed by cyclooctane (1 mL). The solution was heated to 140 °C. The solution was periodically removed from the heating block, cooled, and the ^{1}H NMR spectrum recorded. A sufficient number of spectra were obtained to deduce the experimental half-life.

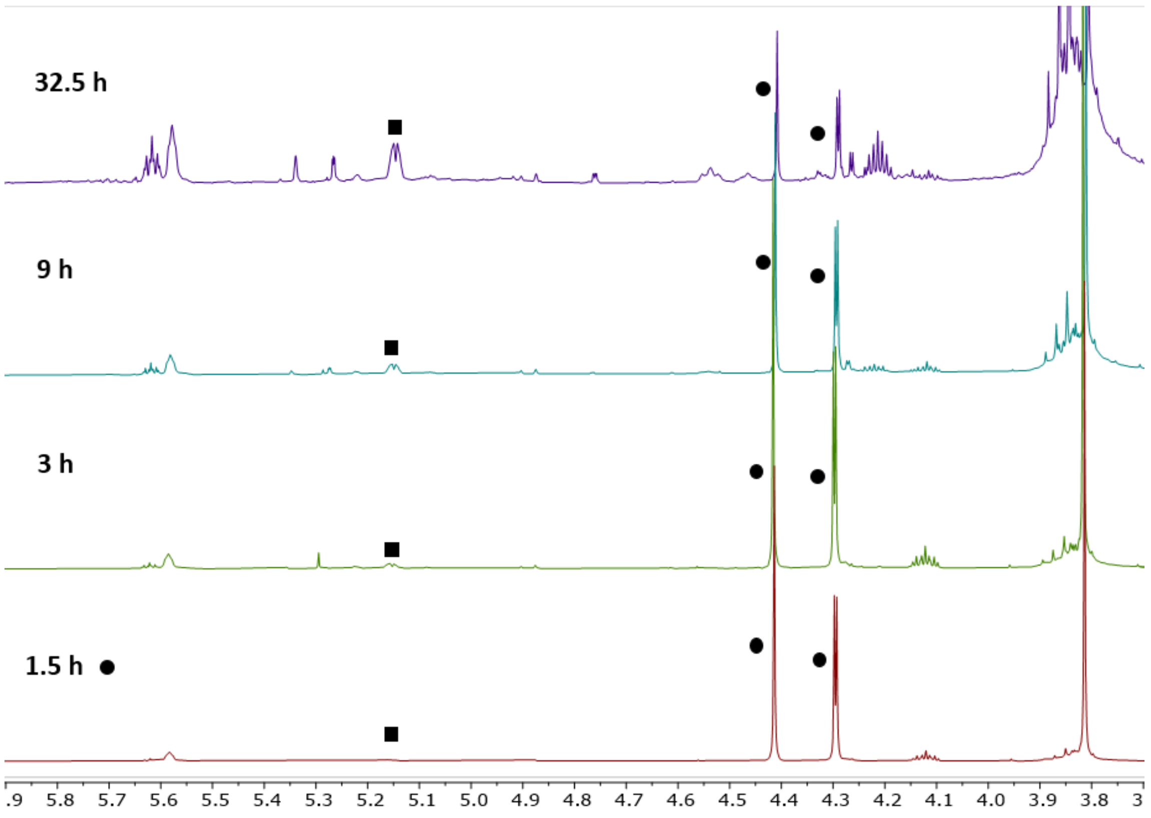

Figure S8. ^{1}H NMR spectra of the cycloisomerization of ether **29d** from which the half-life was determined via in situ (and rapid) trapping of the intermediate benzyne by dihydrogen transfer from cyclooctane.

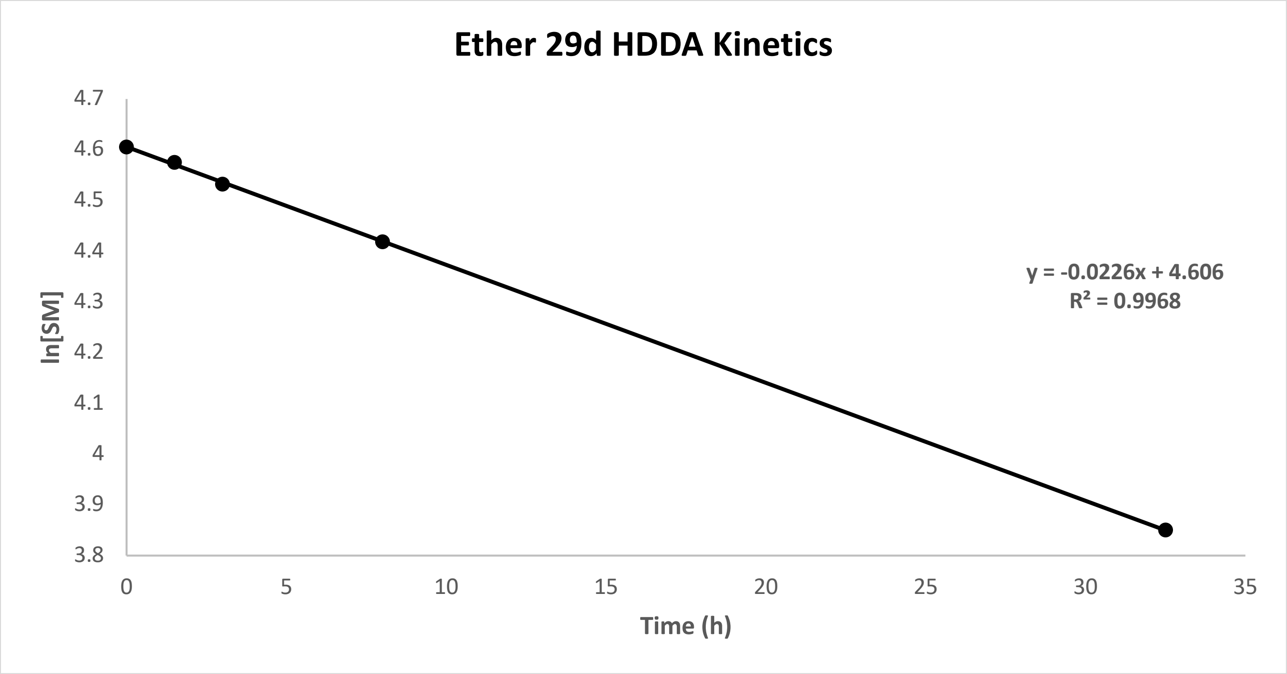

Figure S9. Fit of the above data to the first-order integrated rate law. The linear fit suggests a half-life for the cycloisomerization of **29d** of 40 h at 140 °C.

In situ NMR Data for TDDA Cyclization of Anhydride 2 Derived from Precursors 39 and 1

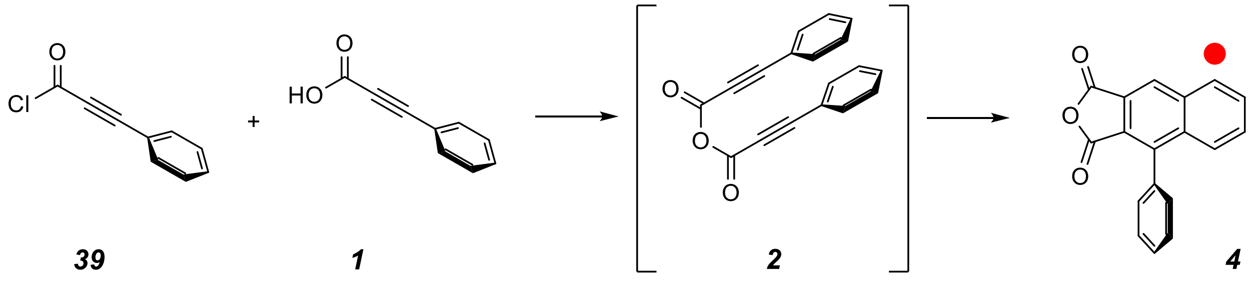

Phenylpropioloyl chloride (**39**, 10 mg, 0.061 mmol, 1 equiv) was placed in a screw-capped culture tube. Proton-sponge® (16 mg, 0.073 mmol, 1.2 equiv) and deuterochloroform (1.0 mL) were added and an initial time point was recorded. Phenylpropiolic acid (**1**, 10 mg, 0.062 mmol, 1 equiv) in $CDCl_3$ (1.0 mL) was added. The ^1H NMR spectrum was recorded at various intervals to monitor conversion to the known naphthyl product **4**.[26]

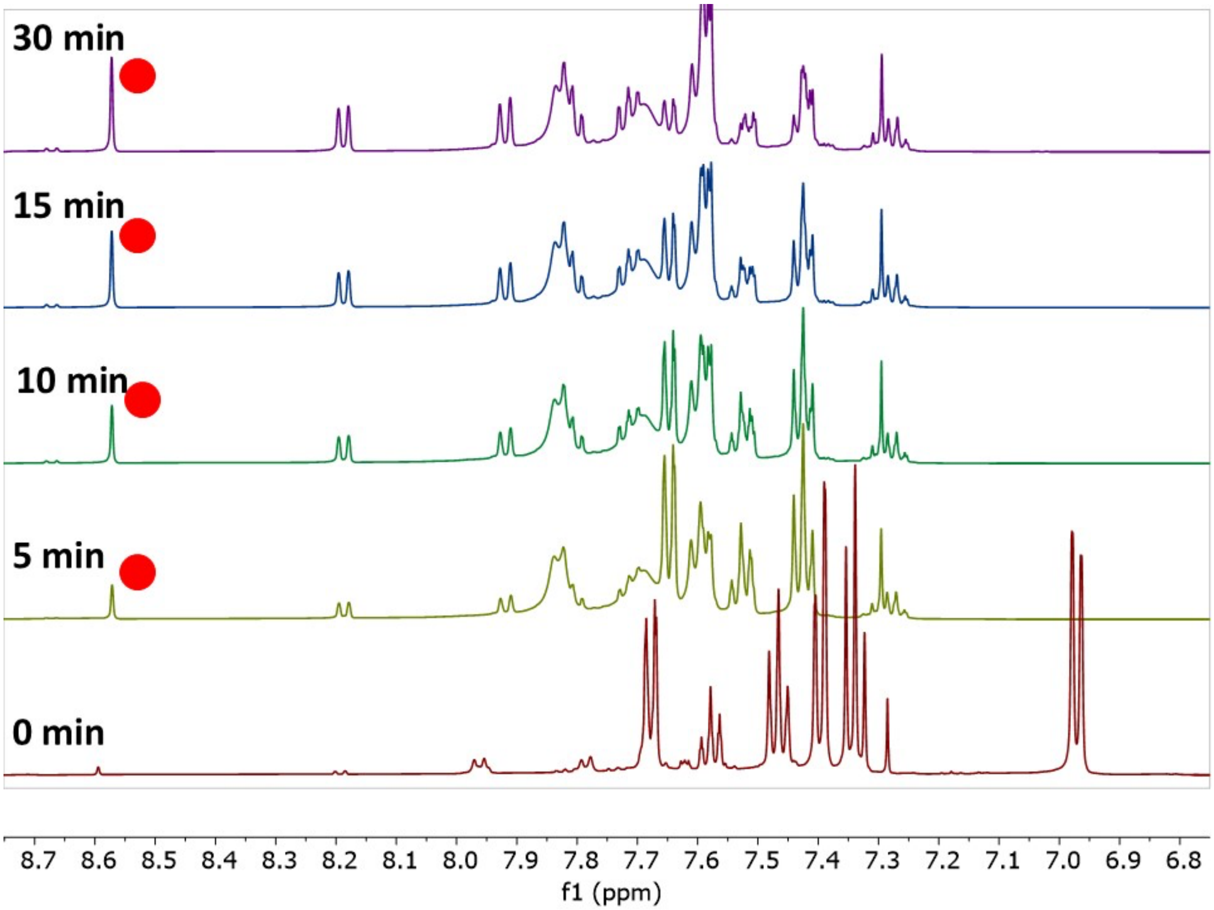

Figure S10. ^1H NMR spectra of the TDDA cycloisomerization of anhydride **2**.

Kinetics Data for HDDA Cyclization of Phthalimide 42 Derived from Precursors 39 and 40

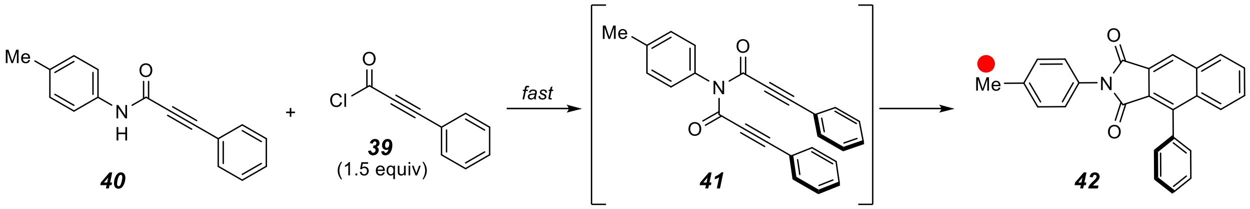

3-Phenyl-n-(p-tolyl)propiolamide (**40**, 5 mg, 0.02 mmol, 1 equiv), triethylamine (4.5 µL, 0.032, 1.5 equiv) and DMAP (0.5 mg, 0.002 mmol, 0.1 equiv) were dissolved in deuterochloroform (600 µL) and added to an NMR tube and an initial time point was recorded. 3-Phenylpropiolyl chloride (**39**, 5.2 mg, 0.032 mmol, 1.5 equiv) was added in one portion. The ^{1}H NMR spectrum was recorded at various intervals to monitor conversion to the phthalimide product **42**.

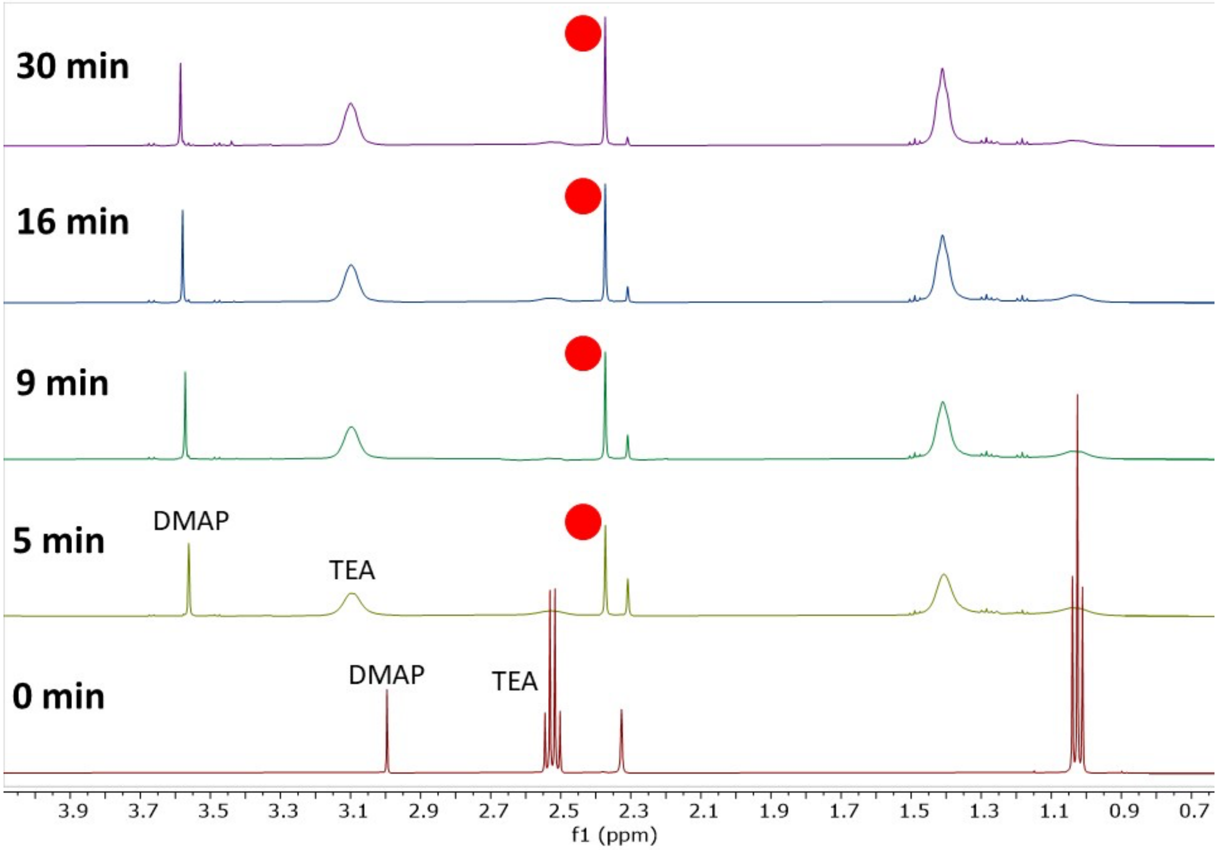

Figure S11. ^{1}H NMR spectra of the cycloisomerization of imide **41**.

V. Discussion of Computational Results

All DFT calculations were performed at 298 K in Gaussian 16.[27] Structure **12** was optimized in and characterized by a frequency calculation at the MN15/6-311++G(d,p) level of theory with solvation treatment (SMD) using tetrahydrofuran. For the complete structures in Figure 8 (**29a-d**, **31**‡, **32**, **33**, and the diradical intermediates), an optimization and frequency calculation were performed at the (U)B3LYP-GD3BJ[28]/6-311+G(d,p) level of theory with solvation treatment (SMD) using chloroform. For the distorted and interacting halves ($\mathbf{32}_{\mathbf{dist}}$, $\mathbf{33}_{\mathbf{dist}}$, and **34**‡), a single point calculation using the same level of theory, basis set, and solvation model was performed. For the structures in Figure 9, an optimization and frequency calculation were performed at the MN15/6-311++G(d,p) level of theory with solvation treatment (SMD) using water.

For all open-shell calculations (i.e., diradicals and the stepwise transition structures leading to or from a diradical), spin symmetry was broken during the optimization using the keyword "guess=(mix, always)" in Gaussian. To search for each transition structure, we used a "Scan" calculation varying the distance between the interacting carbon atoms forming the initial five-membered ring. The structure at the maximum energy from the scan was used as the initial guess of the geometry of the transition structure.

Given on the following pages are the energy values (in Hartree atomic units) and the Cartesian coordinates for each of the computed structures. 3D representations (generated in CYLview20) for each of the structures are also shown.

Energy and Geometry for Intermediate 12

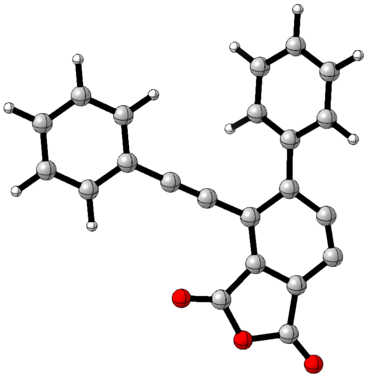

E(RMN15): -1068.91488221 a.u.

Number of imaginary frequencies: 0

Center Number	Atomic Number	Atomic Type	Coordinates (Ångstroms) X	Y	Z
1	6	0	-3.49079	-0.36314	-0.01414
2	6	0	-3.65203	1.01150	-0.04484
3	6	0	-2.63120	1.72223	-0.03505
4	6	0	-1.27132	1.50514	0.04387
5	6	0	-1.04845	0.08038	0.05294
6	6	0	-2.16140	-0.78955	0.03722
7	6	0	-0.23872	2.55326	0.06837
8	6	0	0.27182	-0.44560	0.02470
9	6	0	1.39666	-0.88931	-0.02639
10	6	0	2.73094	-1.39422	-0.07350
11	6	0	3.81525	-0.50423	-0.13156
12	6	0	5.11422	-0.99615	-0.17347
13	6	0	5.34273	-2.37207	-0.15793
14	6	0	4.26822	-3.25973	-0.10022
15	6	0	2.96553	-2.77797	-0.05801
16	6	0	0.92244	2.44209	0.84308
17	6	0	1.85317	3.47631	0.85318
18	6	0	1.63831	4.62355	0.09133
19	6	0	0.47677	4.74581	-0.67045
20	6	0	-0.46044	3.72015	-0.67578
21	6	0	-4.36416	-1.55095	-0.00889
22	8	0	-3.53226	-2.65243	0.03213
23	6	0	-2.19854	-2.26990	0.05920
24	8	0	-1.32831	-3.08546	0.09600
25	8	0	-5.55291	-1.65592	-0.03540
26	1	0	3.62610	0.56389	-0.14694
27	1	0	5.94935	-0.30671	-0.21974
28	1	0	6.35739	-2.75224	-0.19207

29	1	0	4.44648	-4.32880	-0.08895
30	1	0	2.12367	-3.45958	-0.01368
31	1	0	1.08400	1.56577	1.45872
32	1	0	2.74414	3.38857	1.46441
33	1	0	2.36879	5.42457	0.09899
34	1	0	0.30062	5.63960	-1.25758
35	1	0	-1.36673	3.80976	-1.26685

Energy and Geometry for Intermediate 29a

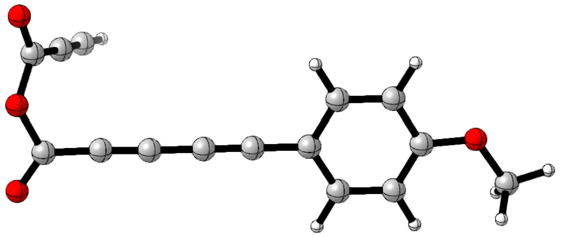

E(UB3LYP): -877.416132434 a.u.

Number of imaginary frequencies: 0

Center Number	Atomic Number	Atomic Type	Coordinates (Ångstroms) X	Y	Z
1	8	0	-5.40493	1.91231	0.97998
2	6	0	-4.97214	1.04604	0.27149
3	6	0	-4.35455	1.27617	-1.00517
4	6	0	-3.87717	1.51713	-2.08130
5	8	0	-5.14325	-0.26855	0.63747
6	6	0	-4.32790	-1.31757	0.18728
7	6	0	-2.94101	-1.03818	0.17286
8	8	0	-4.84535	-2.37200	-0.06413
9	6	0	-1.73894	-0.84875	0.16263
10	6	0	-0.41539	-0.60980	0.14749
11	6	0	0.78469	-0.39538	0.13452
12	6	0	2.16884	-0.14419	0.12205
13	6	0	3.07156	-1.13184	-0.31282
14	6	0	4.43726	-0.89329	-0.32460
15	6	0	4.92768	0.34872	0.10347
16	6	0	4.03567	1.34414	0.53814
17	6	0	2.67821	1.10360	0.54816
18	8	0	6.23541	0.67891	0.13383
19	6	0	7.20490	-0.28852	-0.28768
20	1	0	-3.44491	1.72255	-3.03522
21	1	0	2.69137	-2.09103	-0.64172
22	1	0	5.10769	-1.67006	-0.66353
23	1	0	4.43574	2.29635	0.86404
24	1	0	1.99267	1.87112	0.88516
25	1	0	8.17148	0.19743	-0.17020
26	1	0	7.16469	-1.18166	0.34148
27	1	0	7.05596	-0.55975	-1.33642

Energy and Geometry for Intermediate 29b

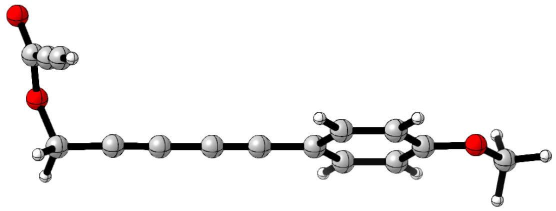

E(UB3LYP): -803.375028802 a.u.

Number of imaginary frequencies: 0

Center Number	Atomic Number	Atomic Type	Coordinates (Ångstroms) X	Y	Z
1	8	0	6.08079	0.19934	1.85286
2	6	0	5.48710	0.13198	0.80315
3	6	0	5.06904	1.30424	0.06894
4	6	0	4.73784	2.30791	-0.50345
5	8	0	5.20195	-1.07089	0.27018
6	6	0	4.45465	-1.16780	-0.97974
7	6	0	3.03866	-0.92072	-0.79984
8	6	0	1.84667	-0.74145	-0.67311
9	6	0	0.51673	-0.52127	-0.51707
10	6	0	-0.67617	-0.32738	-0.38320
11	6	0	-2.06405	-0.10118	-0.22221
12	6	0	-2.92756	-1.15345	0.12338
13	6	0	-4.29087	-0.93945	0.28435
14	6	0	-4.81722	0.34483	0.10000
15	6	0	-3.96551	1.40530	-0.24553
16	6	0	-2.61096	1.18683	-0.40427
17	8	0	-6.12825	0.65857	0.23293
18	6	0	-7.04869	-0.37905	0.58379
19	1	0	4.44014	3.19831	-1.01038
20	1	0	4.88509	-0.48773	-1.71839
21	1	0	4.63158	-2.19222	-1.30913
22	1	0	-2.52230	-2.14761	0.26683
23	1	0	-4.92885	-1.77019	0.55129
24	1	0	-4.39003	2.39236	-0.38330
25	1	0	-1.95817	2.00913	-0.67011
26	1	0	-8.02485	0.09968	0.63566
27	1	0	-7.06575	-1.16300	-0.17897
28	1	0	-6.80080	-0.81099	1.55764

Energy and Geometry for Intermediate 29c

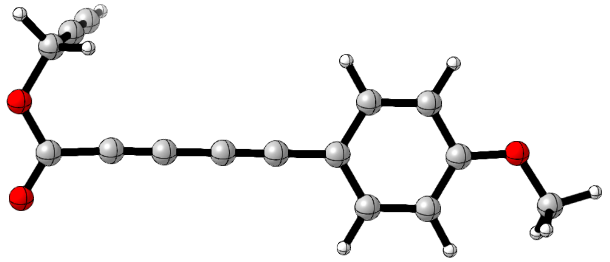

E(UB3LYP): -803.377856525 a.u.

Number of imaginary frequencies: 0

Center Number	Atomic Number	Atomic Type	Coordinates (Ångstroms) X	Y	Z
1	6	0	4.97000	1.04503	-0.83823
2	6	0	4.61614	1.87963	0.30073
3	6	0	4.33250	2.57810	1.23650
4	8	0	5.48462	-0.24998	-0.43003
5	6	0	4.63421	-1.21789	-0.01819
6	6	0	3.23286	-0.92821	-0.05237
7	6	0	2.03069	-0.74843	-0.04763
8	6	0	0.69907	-0.53112	-0.04928
9	6	0	-0.50352	-0.33923	-0.05297
10	6	0	-1.89646	-0.12008	-0.05788
11	6	0	-2.42691	1.14405	-0.39783
12	6	0	-3.79068	1.35277	-0.40364
13	6	0	-4.66793	0.30705	-0.07199
14	6	0	-4.15704	-0.95266	0.26852
15	6	0	-2.78478	-1.15790	0.27432
16	8	0	-5.98444	0.60925	-0.10877
17	6	0	-6.93619	-0.41359	0.20602
18	8	0	5.08056	-2.28272	0.34822
19	1	0	4.11791	0.91384	-1.50915
20	1	0	5.78773	1.49200	-1.40342
21	1	0	4.07571	3.19002	2.07043
22	1	0	-1.75343	1.95227	-0.65459
23	1	0	-4.20567	2.31907	-0.66289
24	1	0	-4.81532	-1.76960	0.52760
25	1	0	-2.38946	-2.13125	0.53745
26	1	0	-7.91314	0.05532	0.10535
27	1	0	-6.85735	-1.24977	-0.49424
28	1	0	-6.80454	-0.76947	1.23158

Energy and Geometry for Intermediate 29d

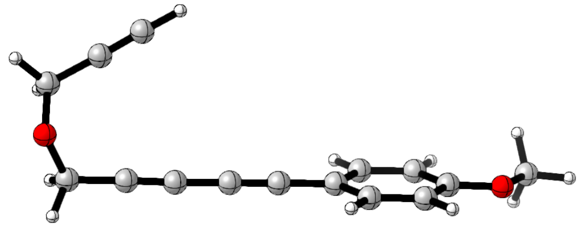

E(UB3LYP): -729.319909074 a.u.

Number of imaginary frequencies: 0

Center Number	Atomic Number	Atomic Type	Coordinates (Ångstroms) X	Y	Z
1	6	0	-5.80690	0.88989	-0.08740
2	6	0	-4.67140	1.71719	0.32617
3	6	0	-3.76210	2.42096	0.67867
4	8	0	-5.72690	-0.46863	0.34530
5	6	0	-4.94551	-1.32167	-0.49281
6	6	0	-3.50480	-1.11799	-0.38886
7	6	0	-2.30400	-0.97321	-0.30650
8	6	0	-0.96439	-0.77602	-0.20438
9	6	0	0.23508	-0.59947	-0.11198
10	6	0	1.63244	-0.39732	0.00365
11	6	0	2.32993	-0.83054	1.15119
12	6	0	3.69293	-0.63492	1.26342
13	6	0	4.40398	-0.00181	0.23293
14	6	0	3.72796	0.43484	-0.91239
15	6	0	2.35628	0.23642	-1.01908
16	8	0	5.73687	0.14152	0.43571
17	6	0	6.51997	0.77266	-0.58072
18	1	0	-6.72125	1.28783	0.35892
19	1	0	-5.92100	0.93423	-1.17784
20	1	0	-2.94062	3.02624	0.98500
21	1	0	-5.19456	-2.33941	-0.18177
22	1	0	-5.25785	-1.20651	-1.53978
23	1	0	1.78654	-1.32092	1.94951
24	1	0	4.23203	-0.96569	2.14295
25	1	0	4.25555	0.92558	-1.71836
26	1	0	1.83537	0.57520	-1.90635
27	1	0	7.54234	0.77190	-0.20689
28	1	0	6.47399	0.21118	-1.51843
29	1	0	6.19291	1.80328	-0.74642

Energy and Geometry for Transition Structure 31a‡

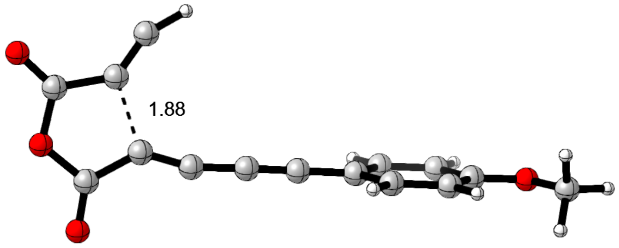

E(UB3LYP): -877.385307043 a.u.

Number of imaginary frequencies: 1

Center Number	Atomic Number	Atomic Type	Coordinates (Ångstroms) X	Y	Z
1	6	0	5.26550	0.78240	-0.06298
2	6	0	3.86623	1.24984	-0.06759
3	6	0	3.30800	2.36736	-0.09167
4	1	0	2.46358	3.02621	-0.10475
5	8	0	6.24810	1.46409	-0.07150
6	8	0	5.38520	-0.58900	-0.04863
7	6	0	4.20156	-1.29764	-0.03519
8	6	0	3.02974	-0.42966	-0.02590
9	6	0	1.78394	-0.60418	0.01344
10	6	0	0.46962	-0.44772	0.05119
11	6	0	-0.75289	-0.32852	0.08941
12	6	0	-2.14393	-0.18973	0.13514
13	6	0	-2.90654	-0.20392	-1.05084
14	6	0	-2.81248	-0.03067	1.37365
15	6	0	-4.28383	-0.06379	-1.01191
16	1	0	-2.40515	-0.32689	-2.00265
17	6	0	-4.18192	0.10853	1.41301
18	1	0	-2.23726	-0.01975	2.29082
19	6	0	-4.93132	0.09471	0.22290
20	1	0	-4.84408	-0.07895	-1.93596
21	1	0	-4.70281	0.23083	2.35465
22	8	0	-6.26300	0.23992	0.36806
23	6	0	-7.09351	0.24020	-0.80058
24	1	0	-7.01032	-0.70781	-1.33835
25	1	0	-8.11060	0.36485	-0.43461
26	1	0	-6.83636	1.07230	-1.46138
27	8	0	4.22646	-2.49553	-0.03298

Energy and Geometry for Transition Structure 31b‡

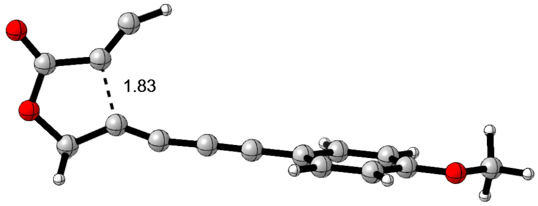

E(UB3LYP): -803.335477035 a.u.

Number of imaginary frequencies: 1

Center Number	Atomic Number	Atomic Type	Coordinates (Ångstroms) X	Y	Z
1	6	0	-5.43666	0.44488	0.31198
2	6	0	-4.06486	0.97425	0.07933
3	6	0	-3.55778	2.12315	0.08951
4	1	0	-2.70471	2.75837	-0.04834
5	8	0	-5.59716	-0.81625	-0.12534
6	6	0	-4.43130	-1.39081	-0.74791
7	6	0	-3.23021	-0.59246	-0.38381
8	6	0	-1.98517	-0.75234	-0.33527
9	6	0	-0.67096	-0.58740	-0.21344
10	6	0	0.54615	-0.46137	-0.10984
11	6	0	1.93562	-0.31429	0.01802
12	6	0	2.70037	0.25389	-1.02047
13	6	0	2.60138	-0.73149	1.19578
14	6	0	4.07318	0.40405	-0.89844
15	1	0	2.20397	0.57818	-1.92675
16	6	0	3.96594	-0.58262	1.32044
17	1	0	2.02683	-1.16995	2.00218
18	6	0	4.71525	-0.01372	0.27620
19	1	0	4.63242	0.84393	-1.71219
20	1	0	4.48161	-0.89852	2.21909
21	1	0	-4.35062	-2.41593	-0.38912
22	1	0	-4.58955	-1.38795	-1.82959
23	8	0	-6.34138	1.04728	0.82925
24	8	0	6.04378	0.08928	0.49508
25	6	0	6.86861	0.67311	-0.51987
26	1	0	6.57278	1.70722	-0.71636
27	1	0	7.88094	0.65265	-0.12107
28	1	0	6.82695	0.08853	-1.44299

Energy and Geometry for Transition Structure 31c‡

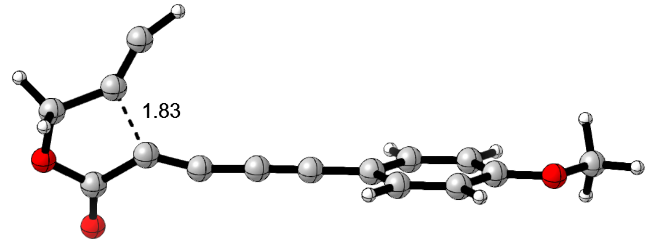

E(UB3LYP): -803.336450455 a.u.

Number of imaginary frequencies: 1

Center Number	Atomic Number	Atomic Type	Coordinates (Ångstroms) X	Y	Z
1	6	0	-5.46587	1.07049	0.22140
2	6	0	-4.13530	1.38043	-0.39645
3	6	0	-3.59463	2.34412	-0.98073
4	1	0	-2.77683	2.85275	-1.44816
5	8	0	-5.68646	-0.35272	0.21770
6	6	0	-4.56514	-1.08544	0.08880
7	6	0	-3.36255	-0.24716	-0.07884
8	6	0	-2.12118	-0.44915	0.03015
9	6	0	-0.80219	-0.31281	0.06838
10	6	0	0.42255	-0.22155	0.10970
11	1	0	-6.27560	1.52051	-0.34875
12	1	0	-5.49928	1.41653	1.25713
13	6	0	1.82010	-0.11253	0.15374
14	6	0	2.46730	0.44078	1.28444
15	6	0	2.61002	-0.54955	-0.92815
16	6	0	3.84111	0.54934	1.32107
17	1	0	1.87314	0.77949	2.12396
18	6	0	3.99177	-0.43896	-0.89342
19	1	0	2.12777	-0.97770	-1.79812
20	6	0	4.61667	0.11338	0.23339
21	1	0	4.34324	0.97155	2.18295
22	1	0	4.57174	-0.78334	-1.73804
23	8	0	5.95258	0.26693	0.36419
24	6	0	6.80380	-0.15140	-0.70886
25	1	0	6.58165	0.40446	-1.62401
26	1	0	7.81742	0.07339	-0.38255
27	1	0	6.70701	-1.22558	-0.88969
28	8	0	-4.59475	-2.29072	0.10780

Energy and Geometry for Transition Structure 31d‡

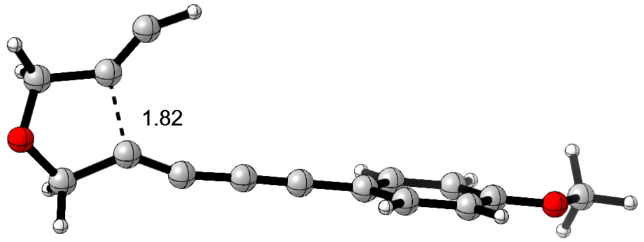

E(UB3LYP): -729.273307938 a.u.

Number of imaginary frequencies: 1

Center Number	Atomic Number	Atomic Type	Coordinates (Ångstroms) X	Y	Z
1	6	0	-5.77908	0.77992	0.01219
2	6	0	-4.33756	1.11378	0.33337
3	6	0	-3.72193	2.09871	0.81306
4	1	0	-2.80515	2.57923	1.09260
5	8	0	-5.93873	-0.62577	0.05019
6	6	0	-4.83215	-1.20498	-0.61611
7	6	0	-3.58847	-0.44997	-0.22999
8	6	0	-2.34509	-0.66390	-0.26558
9	6	0	-1.02602	-0.51743	-0.16243
10	6	0	0.19579	-0.41621	-0.07873
11	6	0	1.59255	-0.29619	0.02165
12	6	0	2.34798	0.26271	-1.02792
13	6	0	2.27633	-0.73284	1.18138
14	6	0	3.72709	0.38537	-0.93410
15	1	0	1.84025	0.60231	-1.92233
16	6	0	3.64709	-0.61222	1.27790
17	1	0	1.71134	-1.16484	1.99807
18	6	0	4.38582	-0.05221	0.22267
19	1	0	4.27646	0.81973	-1.75757
20	1	0	4.17425	-0.94449	2.16405
21	1	0	-4.76964	-2.25286	-0.32409
22	1	0	-4.96004	-1.13883	-1.70640
23	1	0	-6.01770	1.16860	-0.98778
24	1	0	-6.45566	1.21766	0.74359
25	8	0	5.72362	0.02272	0.41387
26	6	0	6.53401	0.59449	-0.61760
27	1	0	7.55605	0.55039	-0.24595
28	1	0	6.45647	0.01730	-1.54341
29	1	0	6.25611	1.63609	-0.80178

Energy and Geometry for 32

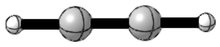

E(UB3LYP): -77.3619484755 a.u.

Number of imaginary frequencies: 0

Center Number	Atomic Number	Atomic Type	Coordinates (Ångstroms)		
			X	Y	Z
1	6	0	0.00000	0.00000	0.60033
2	1	0	0.00000	0.00000	1.66633
3	6	0	0.00000	-0.00000	-0.60033
4	1	0	0.00000	-0.00000	-1.66633

Energy and Geometry for $\mathbf{32}_{dist}$ (from $\mathbf{31a}^{‡}$)

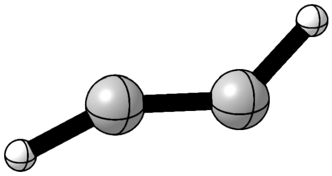

E(UB3LYP): -77.3405555448 a.u.

Center Number	Atomic Number	Atomic Type	Coordinates (Ångstroms) X	Y	Z
1	6	0	-0.62248	-0.14901	-0.00003
2	6	0	0.60154	0.10164	-0.00018
3	1	0	1.64120	-0.15606	0.00076
4	1	0	-1.51558	0.44028	0.00046

Energy and Geometry for 32$_{dist}$ (from 31b‡)

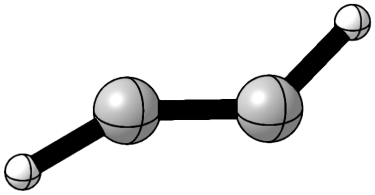

E(UB3LYP): -77.3375907355 a.u.

Center Number	Atomic Number	Atomic Type	Coordinates (Ångstroms) X	Y	Z
1	6	0	0.62206	-0.15452	-0.00084
2	6	0	-0.60396	0.11763	-0.00283
3	1	0	-1.62423	-0.21258	0.01291
4	1	0	1.51564	0.43395	0.00913

Energy and Geometry for 32_{dist} (from 31c‡)

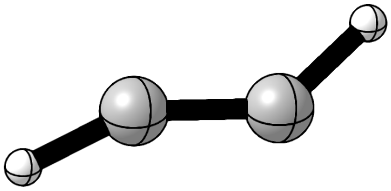

E(UB3LYP): -77.3420241497 a.u.

Center Number	Atomic Number	Atomic Type	Coordinates (Ångstroms) X	Y	Z
1	6	0	-0.62161	-0.14325	-0.00089
2	6	0	0.60409	0.10179	-0.00449
3	1	0	1.64078	-0.16408	0.01968
4	1	0	-1.53566	0.41284	0.01260

Energy and Geometry for 32$_{dist}$ (from 31d‡)

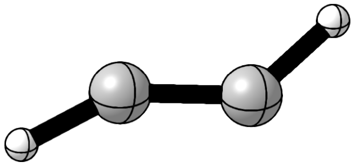

E(UB3LYP): -77.3391990719 a.u.

Center Number	Atomic Number	Atomic Type	Coordinates (Ångstroms) X	Y	Z
1	6	0	0.62247	-0.14909	-0.00116
2	6	0	-0.60625	0.11441	-0.00372
3	1	0	-1.62888	-0.20704	0.01708
4	1	0	1.53155	0.41509	0.01217

Energy and Geometry for 33

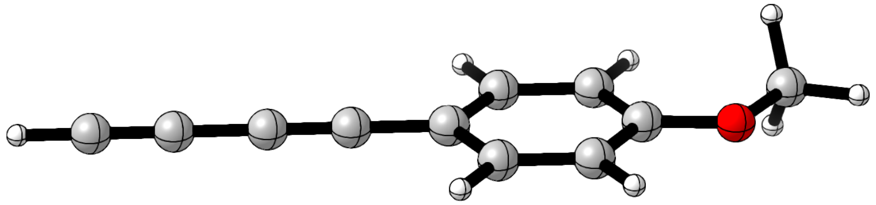

E(UB3LYP): -499.260952084 a.u.

Center Number	Atomic Number	Atomic Type	Coordinates (Ångstroms)		
			X	Y	Z
1	6	0	-0.18641	-1.07979	-0.00001
2	6	0	-1.57420	-1.00274	-0.00005
3	6	0	-2.19851	0.25023	-0.00004
4	6	0	-1.41982	1.41739	0.00001
5	6	0	-0.04087	1.33512	0.00005
6	6	0	0.60414	0.08034	0.00003
7	1	0	0.29449	-2.05029	-0.00002
8	1	0	-2.15412	-1.91519	-0.00006
9	1	0	-1.91991	2.37832	0.00007
10	1	0	0.55479	2.23965	0.00012
11	8	0	-3.54107	0.43617	-0.00018
12	6	0	2.01810	-0.00499	0.00003
13	6	0	3.23131	-0.07271	0.00001
14	6	0	4.58943	-0.14767	-0.00001
15	6	0	5.79803	-0.21345	-0.00001
16	1	0	6.86172	-0.27178	-0.00002
17	6	0	-4.39290	-0.71268	0.00012
18	1	0	-4.23408	-1.31989	0.89590
19	1	0	-5.40978	-0.32427	0.00021
20	1	0	-4.23438	-1.32023	-0.89549

Energy and Geometry for 33_{dist} (from 31a‡)

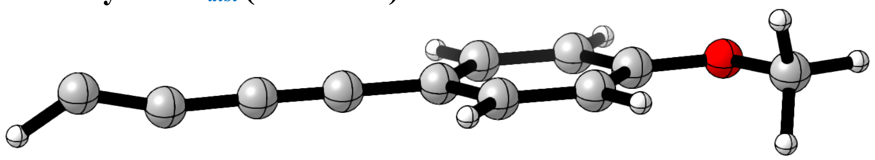

E(UB3LYP): -499.244637543 a.u.

Center Number	Atomic Number	Atomic Type	Coordinates (Ångstroms) X	Y	Z
1	6	0	-5.77837	-0.20832	0.21093
2	6	0	-4.55421	-0.15459	-0.07639
3	6	0	-3.23281	-0.07711	-0.04086
4	6	0	-2.00611	-0.00385	-0.03419
5	6	0	-0.61010	0.08197	-0.02362
6	6	0	0.18128	-1.08509	-0.02299
7	6	0	0.03483	1.34296	-0.01171
8	6	0	1.56392	-1.00625	-0.00939
9	1	0	-0.30208	-2.05396	-0.03397
10	6	0	1.40957	1.42200	0.00163
11	1	0	-0.56270	2.24582	-0.01356
12	6	0	2.18793	0.25055	0.00410
13	1	0	2.14636	-1.91659	-0.00994
14	1	0	1.91256	2.38110	0.01092
15	8	0	3.52262	0.43470	0.01941
16	6	0	4.38239	-0.71256	0.02715
17	1	0	4.23170	-1.31889	-0.86979
18	1	0	5.39583	-0.31655	0.03469
19	1	0	4.21762	-1.31716	0.92297
20	1	0	-6.69006	-0.27967	-0.34462

Energy and Geometry for 33_{dist} (from 31b‡)

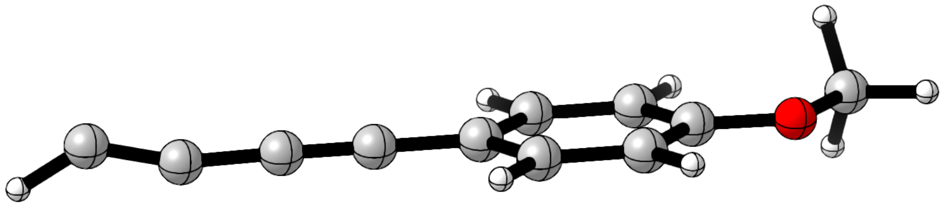

E(UB3LYP): -499.246334369 a.u.

Center Number	Atomic Number	Atomic Type	Coordinates (Ångstroms) X	Y	Z
1	6	0	5.78294	-0.25593	0.20721
2	6	0	4.56562	-0.12529	-0.07404
3	6	0	3.23762	-0.05839	-0.04008
4	6	0	2.01132	0.00598	-0.03658
5	6	0	0.61063	0.08692	-0.02579
6	6	0	-0.18028	-1.07952	-0.02757
7	6	0	-0.04074	1.34385	-0.01022
8	6	0	-1.56462	-1.00544	-0.01426
9	1	0	0.30478	-2.04778	-0.03858
10	6	0	-1.41683	1.42072	0.00401
11	1	0	0.55394	2.24883	-0.00877
12	6	0	-2.19231	0.24846	0.00249
13	1	0	-2.14402	-1.91798	-0.01548
14	1	0	-1.92175	2.37892	0.01713
15	8	0	-3.53041	0.42943	0.01785
16	6	0	-4.38365	-0.72083	0.03225
17	1	0	-4.20889	-1.32538	0.92639
18	1	0	-5.39946	-0.33077	0.04829
19	1	0	-4.23865	-1.32757	-0.86578
20	1	0	6.71903	-0.27685	-0.31067

Energy and Geometry for 33_{dist} (from 31c‡)

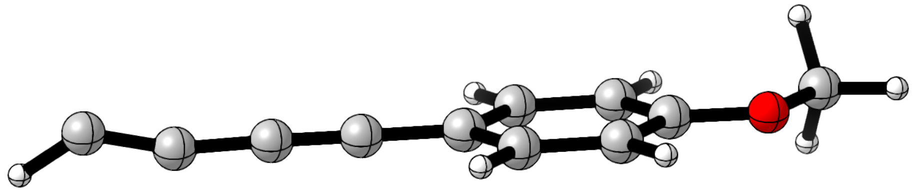

E(UB3LYP): -499.243873375 a.u.

Center Number	Atomic Number	Atomic Type	Coordinates (Ångstroms) X	Y	Z
1	6	0	5.78746	-0.18845	0.21482
2	6	0	4.55964	-0.16991	-0.07810
3	6	0	3.23686	-0.07824	-0.03765
4	6	0	2.01040	-0.00218	-0.03470
5	6	0	0.61058	0.08375	-0.02560
6	6	0	-0.03690	1.34237	-0.01387
7	6	0	-0.18154	-1.08159	-0.02560
8	6	0	-1.41315	1.42133	-0.00096
9	1	0	0.55900	2.24650	-0.01521
10	6	0	-1.56586	-1.00354	-0.01048
11	1	0	0.30107	-2.05096	-0.03766
12	6	0	-2.19109	0.25112	0.00311
13	1	0	-1.91551	2.38092	0.00794
14	1	0	-2.14743	-1.91465	-0.01150
15	8	0	-3.52954	0.43436	0.02077
16	6	0	-4.38355	-0.71523	0.03105
17	1	0	-4.20792	-1.32519	0.92151
18	1	0	-5.39919	-0.32472	0.05096
19	1	0	-4.24041	-1.31764	-0.87038
20	1	0	6.68962	-0.32563	-0.34392

Energy and Geometry for 33$_{dist}$ (from 31d‡)

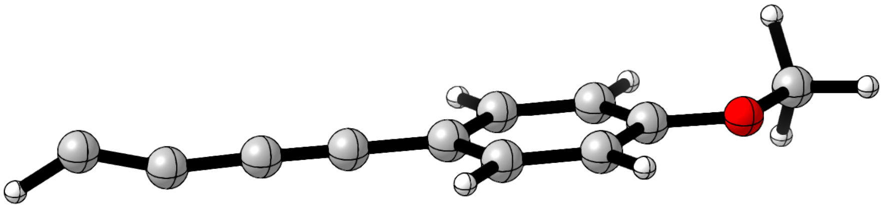

E(UB3LYP): -499.244574731 a.u.

Center Number	Atomic Number	Atomic Type	Coordinates (Ångstroms) X	Y	Z
1	6	0	5.78493	-0.23195	0.23292
2	6	0	4.56999	-0.13992	-0.09646
3	6	0	3.24160	-0.06771	-0.04955
4	6	0	2.01478	0.00257	-0.04219
5	6	0	0.61181	0.08542	-0.02794
6	6	0	-0.18174	-1.07859	-0.02826
7	6	0	-0.04022	1.34152	-0.01142
8	6	0	-1.56733	-1.00332	-0.01261
9	1	0	0.30132	-2.04792	-0.04033
10	6	0	-1.41735	1.41984	0.00432
11	1	0	0.55425	2.24675	-0.01077
12	6	0	-2.19431	0.24973	0.00428
13	1	0	-2.14642	-1.91615	-0.01239
14	1	0	-1.92036	2.37920	0.01772
15	8	0	-3.53533	0.43214	0.02090
16	6	0	-4.38509	-0.71907	0.03436
17	1	0	-5.40244	-0.33252	0.04835
18	1	0	-4.23729	-1.32691	-0.86285
19	1	0	-4.21097	-1.32387	0.92878
20	1	0	6.72215	-0.28680	-0.28042

Energy and Geometry for 34‡ (from 31a‡)

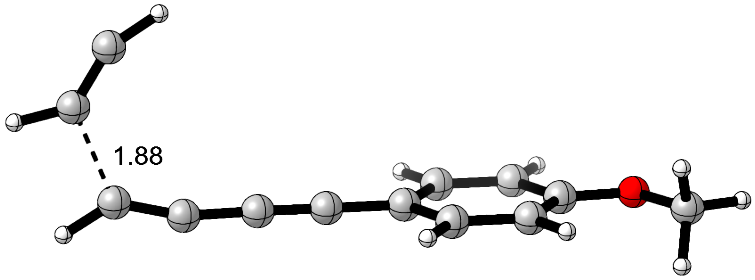

E(UB3LYP): -576.576561040 a.u.

Center Number	Atomic Number	Atomic Type	Coordinates (Ångstroms) X	Y	Z
1	6	0	-5.74195	-0.23382	0.71681
2	6	0	-5.25172	-0.39498	1.85467
3	1	0	-4.44835	-0.48759	2.55704
4	6	0	-4.80646	0.01812	-0.89054
5	6	0	-3.55288	0.08198	-0.98264
6	6	0	-2.25079	0.10335	-0.74294
7	6	0	-1.03811	0.12958	-0.54568
8	6	0	0.34154	0.16126	-0.31783
9	6	0	1.11553	-1.01184	-0.43216
10	6	0	0.98680	1.37215	0.03366
11	6	0	2.48145	-0.98711	-0.20466
12	1	0	0.63205	-1.94231	-0.70226
13	6	0	2.34494	1.39742	0.25982
14	1	0	0.40275	2.27942	0.12291
15	6	0	3.10576	0.22013	0.14453
16	1	0	3.05087	-1.90070	-0.29988
17	1	0	2.84801	2.31791	0.52918
18	8	0	4.42477	0.34952	0.38743
19	6	0	5.26549	-0.80800	0.29371
20	1	0	5.24488	-1.22418	-0.71685
21	1	0	6.26950	-0.45775	0.52440
22	1	0	4.96544	-1.56762	1.02044
23	1	0	-5.62630	0.08821	-1.57453
24	1	0	-6.73458	-0.19100	0.31962

Energy and Geometry for 34‡ (from 31b‡)

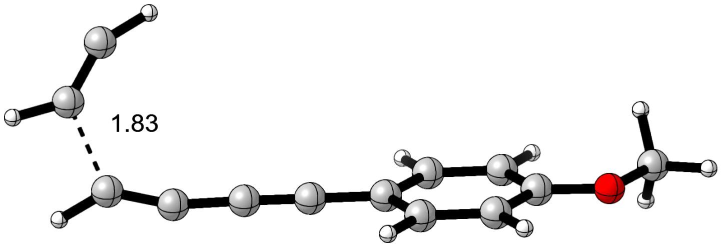

E(UB3LYP): -576.577044530 a.u.

Center Number	Atomic Number	Atomic Type	Coordinates (Ångstroms) X	Y	Z
1	6	0	-5.69421	0.50070	0.58596
2	6	0	-5.21811	1.44599	1.26194
3	1	0	-4.38485	2.06994	1.52000
4	6	0	-4.82556	-0.47957	-0.69866
5	6	0	-3.57603	-0.59854	-0.74940
6	6	0	-2.26488	-0.49344	-0.55180
7	6	0	-1.05004	-0.41250	-0.39184
8	6	0	0.33690	-0.32305	-0.19956
9	6	0	1.06866	0.76595	-0.71408
10	6	0	1.03320	-1.32475	0.51890
11	6	0	2.43879	0.86076	-0.52478
12	1	0	0.54866	1.53992	-1.26500
13	6	0	2.39516	-1.23278	0.70956
14	1	0	0.48419	-2.16754	0.92006
15	6	0	3.11137	-0.14035	0.19074
16	1	0	2.97233	1.70848	-0.93114
17	1	0	2.93406	-1.99487	1.25917
18	8	0	4.44011	-0.14150	0.43146
19	6	0	5.23189	0.94848	-0.05469
20	1	0	4.90511	1.89485	0.38475
21	1	0	6.25092	0.73249	0.26027
22	1	0	5.19057	1.00677	-1.14580
23	1	0	-6.66667	0.06285	0.49937
24	1	0	-5.67805	-0.82241	-1.24697

Energy and Geometry for 34‡ (from 31c‡)

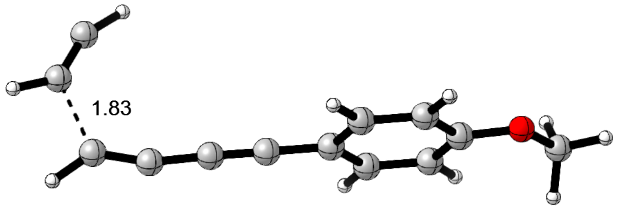

E(UB3LYP): -576.576865282 a.u.

Center Number	Atomic Number	Atomic Type	Coordinates (Ångstroms) X	Y	Z
1	6	0	5.73435	0.17432	0.68768
2	6	0	5.29731	0.13096	1.85795
3	1	0	4.54110	-0.02278	2.59989
4	6	0	4.81311	-0.31224	-0.81621
5	6	0	3.55619	-0.29259	-0.93210
6	6	0	2.25366	-0.17203	-0.71148
7	6	0	1.04094	-0.07187	-0.54030
8	6	0	-0.34237	0.04203	-0.33920
9	6	0	-0.97108	1.31018	-0.33689
10	6	0	-1.13588	-1.10391	-0.13275
11	6	0	-2.33058	1.41697	-0.13482
12	1	0	-0.37409	2.19952	-0.49574
13	6	0	-2.50305	-0.99770	0.07273
14	1	0	-0.66800	-2.08054	-0.13523
15	6	0	-3.10953	0.26621	0.07403
16	1	0	-2.81855	2.38398	-0.13186
17	1	0	-3.08626	-1.89446	0.22776
18	8	0	-4.43008	0.47657	0.26701
19	6	0	-5.28325	-0.65165	0.49113
20	1	0	-4.98684	-1.19316	1.39377
21	1	0	-6.28230	-0.24088	0.62375
22	1	0	-5.27573	-1.32566	-0.37003
23	1	0	6.64767	0.44004	0.19765
24	1	0	5.62472	-0.51057	-1.48468

Energy and Geometry for 34‡ (from 31d‡)

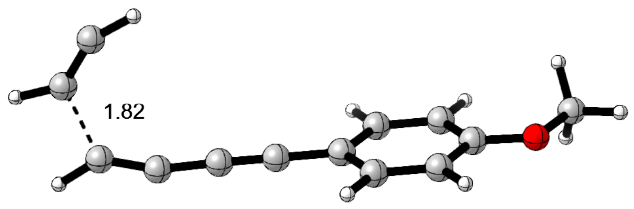

E(UB3LYP): -576.576916529 a.u.

Center Number	Atomic Number	Atomic Type	Coordinates (Ångstroms) X	Y	Z
1	6	0	5.66760	-0.53065	0.60245
2	6	0	5.10580	-1.00683	1.62070
3	1	0	4.21717	-1.22072	2.18116
4	6	0	4.84014	0.32138	-0.78072
5	6	0	3.58845	0.44524	-0.88534
6	6	0	2.27965	0.37424	-0.65288
7	6	0	1.06562	0.32788	-0.46835
8	6	0	-0.32194	0.27446	-0.25104
9	6	0	-1.07996	-0.83144	-0.68354
10	6	0	-0.99356	1.33064	0.40973
11	6	0	-2.44996	-0.89073	-0.47049
12	1	0	-0.58147	-1.64853	-1.19045
13	6	0	-2.35524	1.27476	0.62322
14	1	0	-0.42641	2.18854	0.74912
15	6	0	-3.09666	0.16473	0.18624
16	1	0	-3.00157	-1.75446	-0.81439
17	1	0	-2.87304	2.08064	1.12919
18	8	0	-4.42491	0.20435	0.44332
19	6	0	-5.23682	-0.90111	0.03550
20	1	0	-6.25002	-0.65044	0.34405
21	1	0	-5.20688	-1.03107	-1.05010
22	1	0	-4.92068	-1.82315	0.53147
23	1	0	6.66952	-0.47258	0.23137
24	1	0	5.69388	0.58164	-1.37089

Energy and Geometry for Intermediate Diradical (from 31a‡)

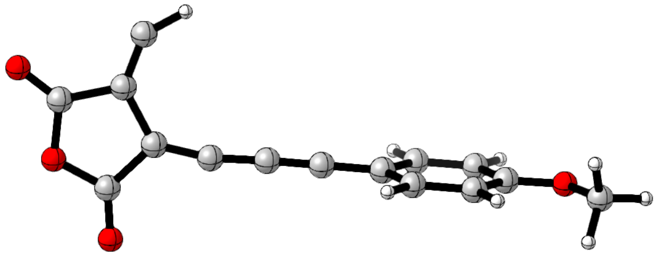

E(UB3LYP): -877.402471443 a.u.

Number of Imaginary Frequencies: 0

Center Number	Atomic Number	Atomic Type	Coordinates (Ångstroms) X	Y	Z
1	6	0	-5.35273	-0.58957	-0.06899
2	6	0	-3.93859	-1.07408	-0.04324
3	6	0	-3.61289	-2.34370	-0.04137
4	1	0	-2.73781	-2.97472	-0.02695
5	8	0	-6.37821	-1.20093	-0.09011
6	8	0	-5.34170	0.79598	-0.06514
7	6	0	-4.04288	1.30101	-0.03881
8	6	0	-3.09626	0.15934	-0.02123
9	6	0	-1.78894	0.29834	0.01126
10	6	0	-0.49664	0.19039	0.04636
11	6	0	0.74724	0.13366	0.08308
12	6	0	2.13602	0.07101	0.12939
13	6	0	2.90164	0.07206	-1.05961
14	6	0	2.81837	0.00373	1.37351
15	6	0	4.28419	0.00678	-1.01639
16	1	0	2.39534	0.12538	-2.01516
17	6	0	4.19267	-0.06027	1.41275
18	1	0	2.24659	0.00399	2.29284
19	6	0	4.94102	-0.06070	0.22147
20	1	0	4.84161	0.01018	-1.94258
21	1	0	4.71967	-0.11099	2.35772
22	8	0	6.28084	-0.12828	0.36993
23	6	0	7.10665	-0.13963	-0.80075
24	1	0	6.97463	0.77739	-1.38115
25	1	0	8.13014	-0.19633	-0.43553
26	1	0	6.89027	-1.01250	-1.42260
27	8	0	-3.84263	2.47915	-0.03355

Energy and Geometry for Intermediate Diradical (from 31b‡)

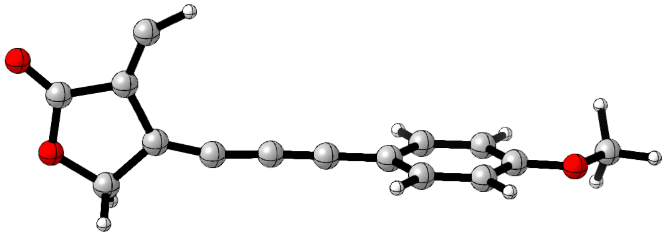

E(UB3LYP): -803.348863528 a.u.

Number of Imaginary Frequencies: 0

Center Number	Atomic Number	Atomic Type	Coordinates (Ångstroms) X	Y	Z
1	6	0	-5.53008	0.37279	-0.05800
2	6	0	-4.09568	0.80571	0.06473
3	6	0	-3.73582	2.05438	0.23918
4	1	0	-2.84029	2.64452	0.35829
5	8	0	-5.57883	-0.96978	-0.21305
6	6	0	-4.26891	-1.57550	-0.20998
7	6	0	-3.27435	-0.43848	-0.04981
8	6	0	-1.97277	-0.56949	-0.02695
9	6	0	-0.67865	-0.40390	0.02702
10	6	0	0.55966	-0.29984	0.07063
11	6	0	1.95030	-0.18572	0.12046
12	6	0	2.70682	0.00708	-1.05745
13	6	0	2.64417	-0.26021	1.35651
14	6	0	4.08794	0.12002	-1.01299
15	1	0	2.19470	0.06718	-2.00976
16	6	0	4.01673	-0.14792	1.40020
17	1	0	2.08256	-0.40749	2.27061
18	6	0	4.75368	0.04285	0.21845
19	1	0	4.63515	0.26644	-1.93391
20	1	0	4.54926	-0.20462	2.34188
21	1	0	-4.22442	-2.28066	0.62150
22	1	0	-4.13943	-2.10665	-1.15383
23	8	0	-6.52292	1.05032	-0.03283
24	8	0	6.09460	0.14011	0.36976
25	6	0	6.90600	0.33125	-0.79351
26	1	0	6.65350	1.26795	-1.29843
27	1	0	7.93130	0.37796	-0.43137
28	1	0	6.80071	-0.50788	-1.48699

Energy and Geometry for Intermediate Diradical (from 31c‡)

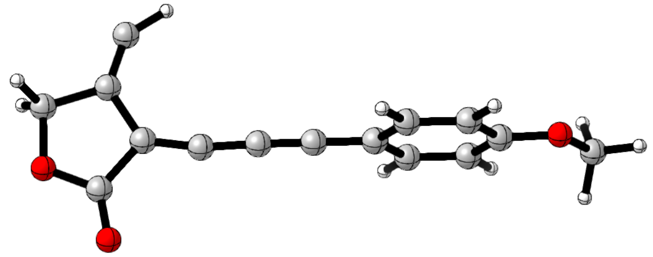

E(UB3LYP): -803.350643684 a.u.

Number of Imaginary Frequencies: 0

Center Number	Atomic Number	Atomic Type	Coordinates (Ångstroms) X	Y	Z
1	6	0	-5.68580	0.79543	-0.09460
2	6	0	-4.23423	1.25393	-0.08529
3	6	0	-3.83508	2.49846	-0.11478
4	1	0	-2.92436	3.07600	-0.11601
5	8	0	-5.65631	-0.64808	-0.05725
6	6	0	-4.39499	-1.13772	-0.02300
7	6	0	-3.42500	-0.00049	-0.03383
8	6	0	-2.12111	-0.16384	0.00145
9	6	0	-0.82480	-0.08804	0.03450
10	6	0	0.41990	-0.06062	0.07137
11	1	0	-6.20739	1.09845	-1.00263
12	1	0	-6.23199	1.14405	0.78216
13	6	0	1.81209	-0.03388	0.12188
14	6	0	2.49183	0.04483	1.36616
15	6	0	2.58426	-0.08528	-1.06096
16	6	0	3.86808	0.06999	1.41205
17	1	0	1.91644	0.08292	2.28257
18	6	0	3.96909	-0.06001	-1.01209
19	1	0	2.08194	-0.14681	-2.01822
20	6	0	4.62198	0.01791	0.22638
21	1	0	4.39116	0.12826	2.35886
22	1	0	4.53008	-0.10197	-1.93537
23	8	0	5.96493	0.04806	0.38150
24	6	0	6.79510	-0.01999	-0.78273
25	1	0	6.61677	0.83399	-1.44208
26	1	0	7.81879	0.01200	-0.41465
27	1	0	6.63057	-0.95431	-1.32669
28	8	0	-4.16737	-2.31958	0.00986

Energy and Geometry for Intermediate Diradical (from 31d‡)

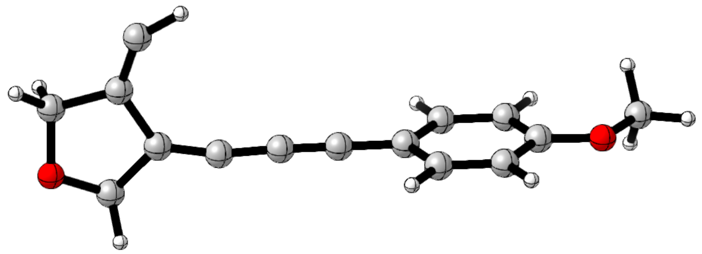

E(UB3LYP): -729.282691709 a.u.

Number of Imaginary Frequencies: 0

Center Number	Atomic Number	Atomic Type	Coordinates (Ångstroms) X	Y	Z
1	6	0	-5.88098	0.56752	-0.06966
2	6	0	-4.42976	0.97937	0.17213
3	6	0	-4.01533	2.15030	0.57185
4	1	0	-3.09674	2.66992	0.79593
5	8	0	-5.88953	-0.85372	0.02877
6	6	0	-4.66106	-1.31918	-0.51817
7	6	0	-3.62592	-0.26007	-0.15006
8	6	0	-2.32913	-0.41653	-0.13960
9	6	0	-1.02957	-0.29710	-0.05448
10	6	0	0.21025	-0.23852	0.01153
11	6	0	1.60456	-0.17165	0.08429
12	6	0	2.37599	0.19031	-1.04252
13	6	0	2.28800	-0.46622	1.29266
14	6	0	3.76002	0.25587	-0.97583
15	1	0	1.87361	0.41990	-1.97415
16	6	0	3.66347	-0.40069	1.35894
17	1	0	1.71583	-0.74552	2.16873
18	6	0	4.41443	-0.04020	0.22737
19	1	0	4.31772	0.53660	-1.85844
20	1	0	4.18667	-0.62610	2.28052
21	1	0	-4.43840	-2.29884	-0.09663
22	1	0	-4.73108	-1.40090	-1.61260
23	1	0	-6.19171	0.88532	-1.07476
24	1	0	-6.57099	0.97233	0.66884
25	8	0	5.75845	-0.00635	0.39620
26	6	0	6.58180	0.33541	-0.72248
27	1	0	7.60606	0.29615	-0.35622
28	1	0	6.45662	-0.38484	-1.53626
29	1	0	6.36053	1.34494	-1.08054

Energy and Geometry for 1,4-Butanediol

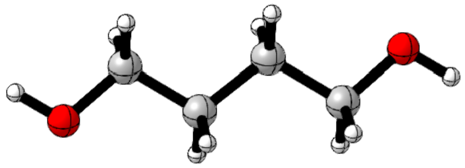

Sum of electronic and thermal enthalpies: -308.477507 a.u.

Number of Imaginary Frequencies: 0

Center Number	Atomic Number	Atomic Type	Coordinates (Ångstroms)		
			X	Y	Z
1	6	0	-0.43044	-1.87936	0.00000
2	6	0	0.42641	-0.63177	0.00000
3	6	0	-0.42641	0.63177	0.00000
4	6	0	0.43044	1.87936	0.00000
5	8	0	-0.42641	3.02099	-0.00000
6	8	0	0.42641	-3.02099	-0.00000
7	1	0	-1.07523	-1.89064	0.88664
8	1	0	-1.07523	-1.89064	-0.88664
9	1	0	1.07594	-0.64472	0.88253
10	1	0	1.07594	-0.64472	-0.88253
11	1	0	-1.07594	0.64472	-0.88253
12	1	0	-1.07594	0.64472	0.88253
13	1	0	1.07523	1.89064	0.88664
14	1	0	1.07523	1.89064	-0.88664
15	1	0	0.11503	3.82013	-0.00000
16	1	0	-0.11503	-3.82013	-0.00000

Energy and Geometry for Dimethyl Ether

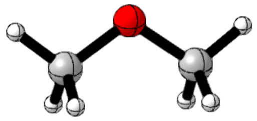

Sum of electronic and thermal enthalpies: -154.801073 a.u.

Number of Imaginary Frequencies: 0

Center Number	Atomic Number	Atomic Type	Coordinates (Ångstroms)		
			X	Y	Z
1	6	0	0.00000	-1.16583	-0.19898
2	1	0	0.89117	-1.19925	-0.83782
3	1	0	-0.00000	-2.03184	0.46242
4	1	0	-0.89117	-1.19925	-0.83782
5	8	0	-0.00000	0.00000	0.60178
6	6	0	0.00000	1.16583	-0.19898
7	1	0	0.00000	2.03184	0.46242
8	1	0	0.89117	1.19925	-0.83782
9	1	0	-0.89117	1.19925	-0.83782

Energy and Geometry for Tetrahydrofuran

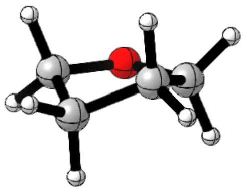

Sum of electronic and thermal enthalpies: -232.113195 a.u.

Number of Imaginary Frequencies: 0

Center Number	Atomic Number	Atomic Type	Coordinates (Ångstroms) X	Y	Z
1	6	0	-0.00000	-1.17398	0.41686
2	6	0	-0.33827	-0.68436	-0.98178
3	1	0	0.02665	-1.35728	-1.75804
4	1	0	-1.42048	-0.56982	-1.09282
5	8	0	-0.00000	-0.00000	1.24514
6	6	0	0.00000	1.17398	0.41686
7	6	0	0.33827	0.68436	-0.98178
8	1	0	-0.02665	1.35728	-1.75804
9	1	0	1.42048	0.56982	-1.09282
10	1	0	-0.99659	1.62887	0.44095
11	1	0	0.72094	1.88801	0.81887
12	1	0	-0.72094	-1.88801	0.81887
13	1	0	0.99659	-1.62887	0.44095

Energy and Geometry for Methanol

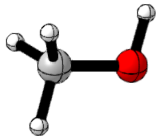

Sum of electronic and thermal enthalpies: -115.579141 a.u.

Number of Imaginary Frequencies: 0

Center Number	Atomic Number	Atomic Type	Coordinates (Ångstroms) X	Y	Z
1	8	0	0.74864	0.12434	0.00000
2	1	0	1.14990	-0.75326	0.00000
3	6	0	-0.66800	-0.01996	0.00000
4	1	0	-1.01577	-0.55122	0.89032
5	1	0	-1.09954	0.98072	-0.00000
6	1	0	-1.01577	-0.55122	-0.89032

Energy and Geometry for 4-Hydroxybutyric Acid

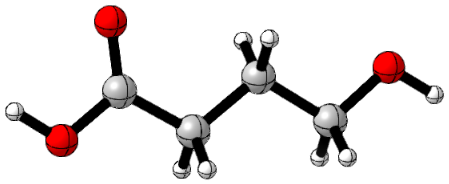

Sum of electronic and thermal enthalpies: -232.113195 a.u.

Number of Imaginary Frequencies: 0

Center Number	Atomic Number	Atomic Type	Coordinates (Ångstroms)		
			X	Y	Z
1	8	0	2.74296	-0.72117	0.00054
2	6	0	1.68501	0.10393	-0.00023
3	8	0	1.81009	1.31371	-0.00032
4	6	0	0.38327	-0.63489	-0.00049
5	6	0	-0.82397	0.28806	0.00017
6	1	0	3.56362	-0.19789	0.00079
7	1	0	0.38284	-1.29324	-0.87571
8	1	0	0.38293	-1.29461	0.87360
9	1	0	-0.80134	0.93404	0.88275
10	6	0	-2.10747	-0.51487	-0.00004
11	1	0	-2.14671	-1.15904	0.88617
12	1	0	-2.14716	-1.15775	-0.88713
13	8	0	-3.20608	0.39375	0.00080
14	1	0	-4.02959	-0.11007	-0.00328
15	1	0	-0.80147	0.93488	-0.88180

Energy and Geometry for Methyl Acetate

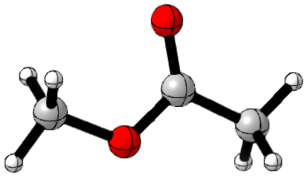

Sum of electronic and thermal enthalpies: -268.074756 a.u.

Number of Imaginary Frequencies: 0

Center Number	Atomic Number	Atomic Type	Coordinates (Ångstroms)		
			X	Y	Z
1	6	0	0.46501	0.16281	-0.00205
2	8	0	0.27963	1.36704	-0.00024
3	6	0	1.79740	-0.50945	0.00073
4	1	0	2.58965	0.23392	-0.04070
5	1	0	1.89607	-1.10919	0.90757
6	8	0	-0.54441	-0.71331	-0.00272
7	6	0	-1.86904	-0.16165	0.00173
8	1	0	-2.54702	-1.01083	0.00017
9	1	0	-2.02640	0.44805	-0.88811
10	1	0	-2.02295	0.44209	0.89623
11	1	0	1.86863	-1.18413	-0.85392

Energy and Geometry for γ-Butyrolactone

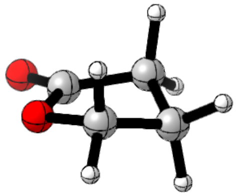

Sum of electronic and thermal enthalpies: -306.142769 a.u.

Number of Imaginary Frequencies: 0

Center Number	Atomic Number	Atomic Type	Coordinates (Ångstroms)		
			X	Y	Z
1	6	0	-0.86873	0.00404	0.00521
2	8	0	-2.07884	-0.03022	-0.07904
3	6	0	0.03115	1.19409	0.18642
4	1	0	-0.34230	2.04995	-0.37195
5	1	0	0.01811	1.44229	1.25321
6	8	0	-0.13378	-1.11924	-0.04986
7	6	0	1.27058	-0.80857	0.14284
8	1	0	1.84607	-1.49300	-0.47528
9	1	0	1.50297	-0.97357	1.19653
10	6	0	1.39002	0.65678	-0.24845
11	1	0	2.23105	1.14302	0.24232
12	1	0	1.50698	0.74901	-1.32974

Energy and Geometry for Acetic Acid

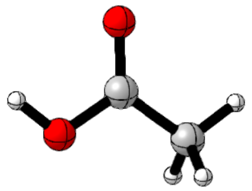

Sum of electronic and thermal enthalpies: -228.853272 a.u.

Number of Imaginary Frequencies: 0

Center Number	Atomic Number	Atomic Type	Coordinates (Ångstroms)		
			X	Y	Z
1	6	0	-0.08324	0.11595	-0.00025
2	8	0	-0.62114	1.20787	0.00010
3	6	0	1.38773	-0.12720	0.00012
4	1	0	1.65726	-0.71593	-0.87892
5	1	0	1.92283	0.81851	-0.00451
6	1	0	1.65615	-0.70866	0.88361
7	8	0	-0.79382	-1.02241	0.00012
8	6	0	-1.74351	-0.81009	-0.00117

Energy and Geometry for Succinic Acid

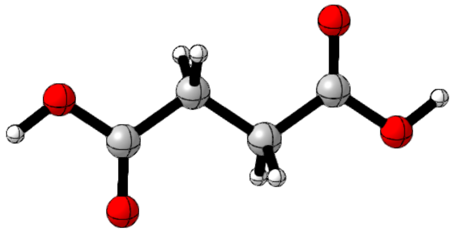

Sum of electronic and thermal enthalpies: -456.534656 a.u.

Number of Imaginary Frequencies: 0

Center Number	Atomic Number	Atomic Type	Coordinates (Ångstroms)		
			X	Y	Z
1	8	0	2.14501	-1.29042	0.00055
2	6	0	1.91319	-0.09682	-0.00013
3	6	0	-1.91319	0.09682	0.00022
4	8	0	-2.14501	1.29042	0.00051
5	6	0	0.54951	0.52185	0.00007
6	6	0	-0.54951	-0.52185	0.00016
7	8	0	2.88783	0.82186	-0.00056
8	8	0	-2.88783	-0.82186	-0.00065
9	1	0	0.48191	1.17468	0.87461
10	1	0	0.48167	1.17474	-0.87442
11	1	0	-0.48178	-1.17467	0.87470
12	1	0	-0.48181	-1.17475	-0.87433
13	1	0	3.75367	0.37709	-0.00048
14	1	0	-3.75367	-0.37709	-0.00082

Energy and Geometry for Acetic Anhydride

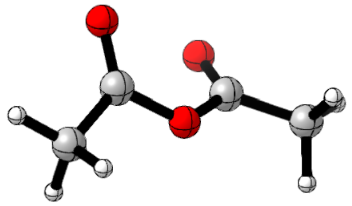

Sum of electronic and thermal enthalpies: -381.325383 a.u.

Number of Imaginary Frequencies: 0

Center Number	Atomic Number	Atomic Type	Coordinates (Ångstroms)		
			X	Y	Z
1	8	0	1.28242	1.21546	-0.52779
2	6	0	1.20780	0.08592	-0.12017
3	8	0	0.00001	-0.57628	0.00006
4	6	0	-1.20781	0.08590	0.12021
5	8	0	-1.28247	1.21546	0.52777
6	6	0	-2.33165	-0.80562	-0.26719
7	6	0	2.33169	-0.80559	0.26714
8	1	0	-2.22732	-1.06583	-1.32283
9	1	0	-2.27644	-1.73076	0.30905
10	1	0	-3.27998	-0.30390	-0.09314
11	1	0	2.22753	-1.06565	1.32283
12	1	0	2.27633	-1.73081	-0.30897
13	1	0	3.28000	-0.30393	0.09285

Energy and Geometry for Succinic Anhydride

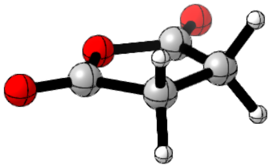

Sum of electronic and thermal enthalpies: -380.156342 a.u.

Number of Imaginary Frequencies: 0

Center Number	Atomic Number	Atomic Type	Coordinates (Ångstroms) X	Y	Z
1	6	0	0.00000	1.13850	0.20302
2	8	0	-0.00015	2.22126	0.71810
3	6	0	0.00026	0.76105	-1.24642
4	1	0	-0.88091	1.20082	-1.71447
5	1	0	0.88214	1.20012	-1.71384
6	8	0	0.00000	0.00000	0.98599
7	6	0	-0.00000	-1.13850	0.20302
8	6	0	-0.00026	-0.76105	-1.24642
9	1	0	0.88091	-1.20082	-1.71447
10	1	0	-0.88214	-1.20012	-1.71384
11	8	0	0.00015	-2.22126	0.71810

VI. X-Ray Crystallographic Data for Compound 15

Data for 15 (CCDC Deposition Number 2353613)

Data collection

A crystal (approximate dimensions 0.150 x 0.070 x 0.030) mm was placed onto the tip of a 0.15 mm MiTeGen loop and mounted on a Bruker Photon-III CPAD diffractometer for a data collection at 100(2) K.[29] A preliminary set of cell constants was calculated from reflections harvested from three sets of frames. These initial sets of frames were oriented such that orthogonal wedges of reciprocal space were surveyed. This produced initial orientation matrices determined from 122 reflections. The data collection was carried out using MoKα radiation (graphite monochromator) with a frame time of 90 seconds and a detector distance of 7.0 cm. A strategy program was used to assure complete coverage of all unique data to a resolution of 0.83 Å. All major sections of frames were collected with 1.2° steps in ω or φ at different detector positions in 2θ. The intensity data were corrected for absorption and decay (SADABS).[30] Final cell constants were calculated from the xyz centroids of 2959 strong reflections from the actual data collection after integration (SAINT).[31] Please refer to Table S1 for additional crystal and refinement information.

Structure solution and refinement

The structure was solved using SHELXT[32] and refined using SHELXL-2019/1[33] The space group $P2_1/c$ was determined based on systematic absences and intensity statistics. A direct-methods solution was calculated which provided most non-hydrogen atoms from the E-map. Full-matrix least squares / difference Fourier cycles were performed which located the remaining non-hydrogen atoms. All non-hydrogen atoms were refined with anisotropic displacement parameters. All hydrogen atoms were placed in ideal positions and refined as riding atoms with relative isotropic displacement parameters. The final full matrix least squares refinement converged to $R1 = 0.0397$ and $wR2 = 0.1032$ (F^2, all data).

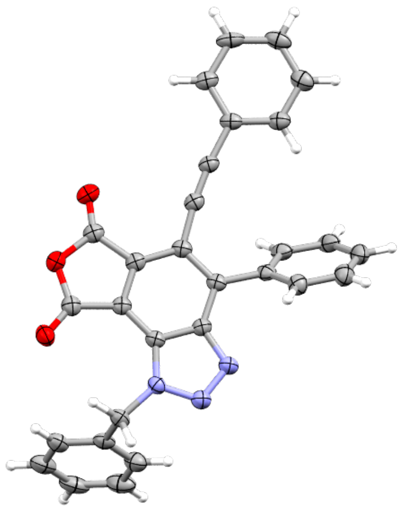

Table S1. Crystal data and structure refinement for 24008.

Ellipsoid contour probability level = 50% (hydrogen atom spheres with fixed 0.15 Å radii)

Identification code	24008z_a	
Empirical formula	$C_{29}H_{17}N_3O_3$	
Formula weight	455.45	
Temperature	100(2) K	
Wavelength	0.71073 Å	
Crystal system	Monoclinic	
Space group	$P2_1/c$	
Unit cell dimensions	*a* = 7.2038(5) Å	α = 90°
	b = 11.3027(7) Å	β = 92.223(2)°
	c = 26.8583(17) Å	γ = 90°
Volume	2185.2(2) $Å^3$	
Z	4	
Density (calculated)	1.384 Mg/m^3	
Absorption coefficient	0.092 mm^{-1}	
F(000)	944	
Crystal color, morphology	yellow, plate	
Crystal size	0.150 x 0.070 x 0.030 mm^3	
Theta range for data collection	1.955 to 25.371°	

Index ranges	$-7 \leq h \leq 8$, $-13 \leq k \leq 12$, $-32 \leq l \leq 28$
Reflections collected	18593
Independent reflections	4013 [R(int) = 0.0318]
Observed reflections	3071
Completeness to theta = 25.242°	100.0%
Absorption correction	multi-scan
Max. and min. transmission	0.7452 and 0.5612
Refinement method	Full-matrix least-squares on F^2
Data / restraints / parameters	4013 / 0 / 316
Goodness-of-fit on F^2	1.026
Final R indices [I>2sigma(I)]	$R1$ = 0.0397, $wR2$ = 0.0944
R indices (all data)	$R1$ = 0.0586, $wR2$ = 0.1032
Extinction coefficient	n/a
Largest diff. peak and hole	0.186 and -0.199 e.Å^{-3}

VII. Supporting Information References

[1] Hoye, T. R.; Hanson, P. R.; Vyvyan, J. R. A Practical Guide to First-Order Multiplet Analysis in ^{1}H NMR Spectroscopy. *J. Org. Chem.* **1994**, *59*, 4096–4103.

[2] Hoye, T. R.; Zhao, H. A Method for Easily Determining Coupling Constant Values: An Addendum to "A Practical Guide to First-Order Multiplet Analysis in ^{1}H NMR Spectroscopy". *J. Org. Chem.* **2002**, *67*, 4014–4016.

[3] Sneddon, D. S.; Hoye, T. R. Arylhydrazine Trapping of Benzynes: Mechanistic Insights and a Route to Azoarenes. *Org. Lett.* **2021**, *23*, 3432–3436.

[4] Smela, M. P.; Hoye, T. R. A Traceless Tether Strategy for Achieving Formal Intermolecular Hexadehydro-Diels–Alder Reactions. *Org. Lett.* **2018**, *20*, 5502–5505.

[5] Gung, B. W.; Kumi, G. Total Synthesis of (*S*)-(-)-(*E*)-15,16-Dihydrominquartynoic Acid: A Highly Potent Anticancer Agent. *J. Org. Chem.* **2004**, *69*, 3488–3492.

[6] Perera, E. L.; Lee, D. Synthesis of 4-Pyrones by Formal Hydration of 1,3-Diynones Promoted by 1,4-Addition of Piperidine. *Org. Lett.* **2022**, *24*, 7042–7046.

[7] Chuchmaerva, M.; Strauch, C.; Schröder, S.; Collong, A.; Niggemann, A. Regio- and Stereoselective N-addition to an Open Bromo Vinyl Cation. *Tetrahedron Lett.* **2021**, *74*, 153173.

[8] Andrade, C. B.; Carvalho, D. B; Trefzger, O. S.; Kassab, N. M.; Guerrero Jr., P. G.; Barbosa, S. L.; Shiguemoto, C. Y. K.; Baroni, A. C. M. One-Pot Synthesis of 1,3-Butadiyne Derivatives and their Application in the Synthesis of Unsymmetrical 2,5-Diarylthiophenes. *Eur. J. Org. Chem.* **2019**, 696–704.

[9] Marino, J. P.; Nguyen, H. N. Bulky Trialkylsilyl Acetylenes in the Cadiot−Chodkiewicz Cross-Coupling Reaction. *J. Org. Chem.* **2002**, *67*, 6841–6844.

[10] Rycek, L.; Mateus, M.; Beytlerová, Kotora, M. Catalytic Cyclotrimerization Pathway for Synthesis of Selaginpulvilins C and D: Scope and Limitations. *Org. Lett.* **2021**, *23*, 4511–4515.

[11] West, K.; Wang, C.; Batsanov, A. S.; Bryce, M. R. Are Terminal Aryl Butadiynes Stable? Synthesis and X-ray Crystal Structures of a Series of Aryl- and Heteroaryl-butadiynes (Ar−C⁝C−C⁝C−H). *J. Org. Chem.* **2006**, *71*, 8541–8544.

[12] Chen, J,; Palani, V.; Hoye, T. R. Reactions of HDDA-Derived Benzynes with Sulfides: Mechanism, Modes, and Three-Component Reactions. *J. Am. Chem. Soc.* **2016**. *138*, 4318–4321.

[13] Jones, G. E.; Kendrick, D. A.; Holmes, A. B. 1,4-Bis(trimethylsilyl)buta-1,3-diyne. *Org. Synth.* **1987**, *65*, 52.

[14] Doak, B. C.; Scanlon, M. J.; Simpson, J. S. Synthesis of Unsymmetrical 1,1′-Disubstituted Bis(1,2,3-triazole)s Using Monosilylbutadiynes. *Org. Lett.* **2011**, *13*, 537–539.

[15] Zhao, G.; Liang, L.; Wen, C.; Tong, R. In Situ Generation of Nitrile Oxides from NaCl–Oxone Oxidation of Various Aldoximes and Their 1,3-Dipolar Cycloaddition. *Org. Lett.* **2019.** *21*, 315–319.

[16] Friedrichsen, W.; Schröer, W.; Debaerdemaeker, T. Reactionen mesoionischer Fünfringheterocyclen mit O-chinoiden Verbindungen, II. Ein Beitrag zum Problem der Ketentautomerie bei mesoionischen 1,3-Oxazolium-5-olaten. *Liebigs. Ann. Chem.* **1980**. 1836–1849.

[17] Spielmann, K.; Xiang, M.; Schwartz, L. A.; Krische M. J. Direct Conversion of Primary Alcohols to 1,2-Amino Alcohols: Enantioselective Iridium-Catalyzed Carbonyl Reductive Coupling of Phthalimido-Allene via Hydrogen Auto-Transfer. *J. Am. Chem. Soc.* **2019**, *141*, 14136–14141.

[18] Muller, D.; Zeltser, I.; Bitan, G.; Gilon, C. Building Units for N-Backbone Cyclic Peptides. 3. Synthesis of Protected N^{α}-(ω-Aminoalkyl)amino Acids and N^{α}-(ω-Carboxyalkyl)amino Acids. *J. Org. Chem.* **1997**, *62*, 411–416.

[19] Verma, A.; Snead, R. F.; Dai, Y.; Slebodnick, C.; Yang, Y.; Yu, H.; Yao, F.; Santos, W. L. Substrate-Assisted, Transition-Metal-Free Diboration of Alkynamides with Mixed Diboron: Regio- and Stereoselective Access to trans-1,2-Vinyldiboronates. *Angew. Chem. Int. Ed.* **2017**, *56*, 5111–5115.

[20] Chalifoux, W. A.; McDonald, R.; Ferguson, M. J.; Tykwinski, R. R. *tert*-Butyl-End-Capped Polyynes: Crystallographic Evidence of Reduced Bond-Length Alternation. *Angew. Chem. Int. Ed.* **2009**. *48*, 7915–7919.

[21] Karmakar, R.; Yun, S. Y.; Chen, J.; Xia, Y.; Lee, D. Benzannulation of Triynes to Generate Functionalized Arenes by Spontaneous Incorporation of Nucleophiles. *Angew. Chem. Int. Ed.* **2015**, *54*, 6582–6586.

[22] Gu, Y.; Dai, L.; Mao, K.; Zhang, J.; Wang, C.; Zhao, L.; Rong, L. Time-Economical Radical Cascade Cyclization/Haloazidation of 1,6-Enynes: Construction of Highly Functional Succinimide Derivatives. *Org. Lett.* **2020**. *22*. 2956–2960.

[23] Deimling, M.; Kirchof, M.; Schwager, B.; Qawasmi, Y.; Savin, A.; Mühlhäuser, T.; Frey, W.; Claasen, B.; Baro, A.; Scottmann, T.; Laschat, S. Asymmetric Catalysis in Liquid Confinement: Probing the Performance of Novel Chiral Rhodium–Diene Complexes in Microemulsions and Conventional Solvents. *Chem. Eur. J.* **2019**. *25*. 9464–9476.

[24] Islam, A.M.; Hassan, E. A.; Hannout, I. B.; Taha, N. M. Benzphthalimides and Related Compounds. Part IV. Synthesis and Reactions of 7-Phenyl-5:6-benzo-3-benzal-2-arylphthalimidines. *Egypt. J. Chem.,* **1974**. *17*, 758.

[25] Hoops, S.; Sahle, S.; Gauge, R.; Lee, C.; Pahle, J.; Simus, N.; Singhal, M.; Xu, L.; Mendes, P.; Kummer, U. COPASI—a COmplex PAthway Simulator. *Bioinformatics* **2006**, *22*, 3067–3074.

[26] Kim, K. H.; Lim, C. H.; Lim, J. W.; Kim, J. N. 2,3-Dichloro-5,6-dicyano-*para*-benzoquinone (DDQ)/Methanesulfonic Acid (MsOH)-Mediated Intramolecular Arene-Alkene Oxidative Coupling. *Adv. Synth. Catal.* **2014**, *356*, 697–704.

[27] Frisch, M. J.; Trucks, G. W.; Schlegel, H. B.; Scuseria, G. E.; Robb, M. A.; Cheeseman, J. R.; Scalmani, G.; Barone, V.; Mennucci, B.; Petersson, G. A.; Nakatsuji, H.; Caricato, M.; Li, X.; Hratchian, H. P.; Izmaylov, A. F.; Bloino, J.; Zheng, G.; Sonnenberg, J. L.; Hada, M.; Ehara,

M.; Toyota, K.; Fukuda, R.; Hasegawa, J.; Ishida, M.; Nakajima, T.; Honda, Y.; Kitao, O.; Nakai, H.; Vreven, T.; Montgomery, J. A.; Peralta, Jr.; J. E.; Ogliaro, F.; Bearpark, M.; Heyd, J. J.; Brothers, E.; Kudin, K. N.; Staroverov, V. N.; Keith, T.; Kobayashi, R.; Normand, J.; Raghavachari, K.; Rendell, A.; Burant, J. C.; Iyengar, S. S.; Tomasi, J.; Cossi, M.; Rega, N.; Millam, J. M.; Klene, M.; Knox, J. E.; Cross, J. B.; Bakken, V.; Adamo, C.; Jaramillo, J.; Gomperts, R.; Stratmann, R. E.; Yazyev, O.; Austin, A. J.; Cammi, R.; Pomelli, C.; Ochterski, J. W.; Martin, R. L.; Morokuma, K.; Zakrzewski, V. G.; Voth, G. A.; Salvador, P.; Dannenberg, J. J.; Dapprich, S.; Daniels, A. D.; Farkas, O.; Foresman, J. B.; Ortiz, J. V.; Cioslowski, J.; Fox, D. J. Gaussian 16, Revision C.01, Gaussian Inc.: Wallingford, CT (2016).

[28] Grimme, S.; Antony, J.; Ehrlich, S.; Krieg, H. A Consistent and Accurate ab initio Parametrization of Density Functional Dispersion Correction (DFT-D) for the 94 Elements H-Pu. *Chem. Phys.* **2010**, *132*, 154104.

[29] APEX4, Bruker Analytical X-ray Systems, Madison, WI (2016).

[30] SADABS, Bruker Analytical X-ray Systems, Madison, WI (2016).

[31] SAINT Bruker Analytical X-ray Systems, Madison, WI (2016).

[32] SHELXTL 2018/2, Bruker Analytical X-Ray Systems, Madison, WI (2016); G. M. Sheldrick, *Acta Cryst.* **A71**, 3-8 (2015).

[33] SHELXL 2019/1; G. M. Sheldrick, *Acta Cryst.* **C71**, 3-8 (2015).

VIII. Copies of 1D and 2D NMR Spectra

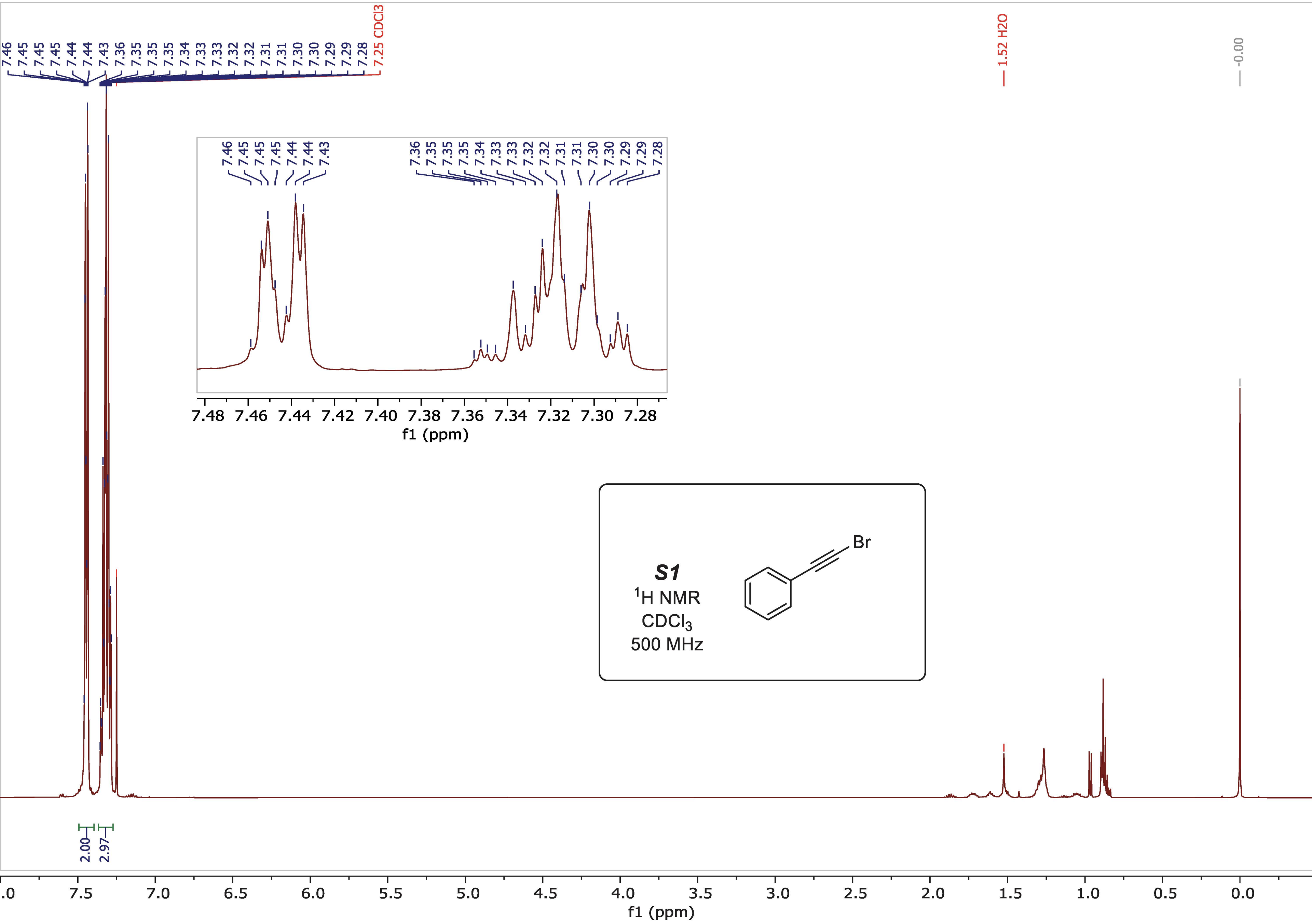
7.46
7.45
7.45
7.45
7.44
7.44
7.43
7.36
7.35
7.35
7.35
7.34
7.33
7.33
7.32
7.32
7.31
7.31
7.30
7.30
7.29
7.29
7.28
7.25 CDCl3
1.52 H2O
-0.00
S1
1H NMR
CDCl3
500 MHz
Br
2.00
2.97
f1 (ppm)

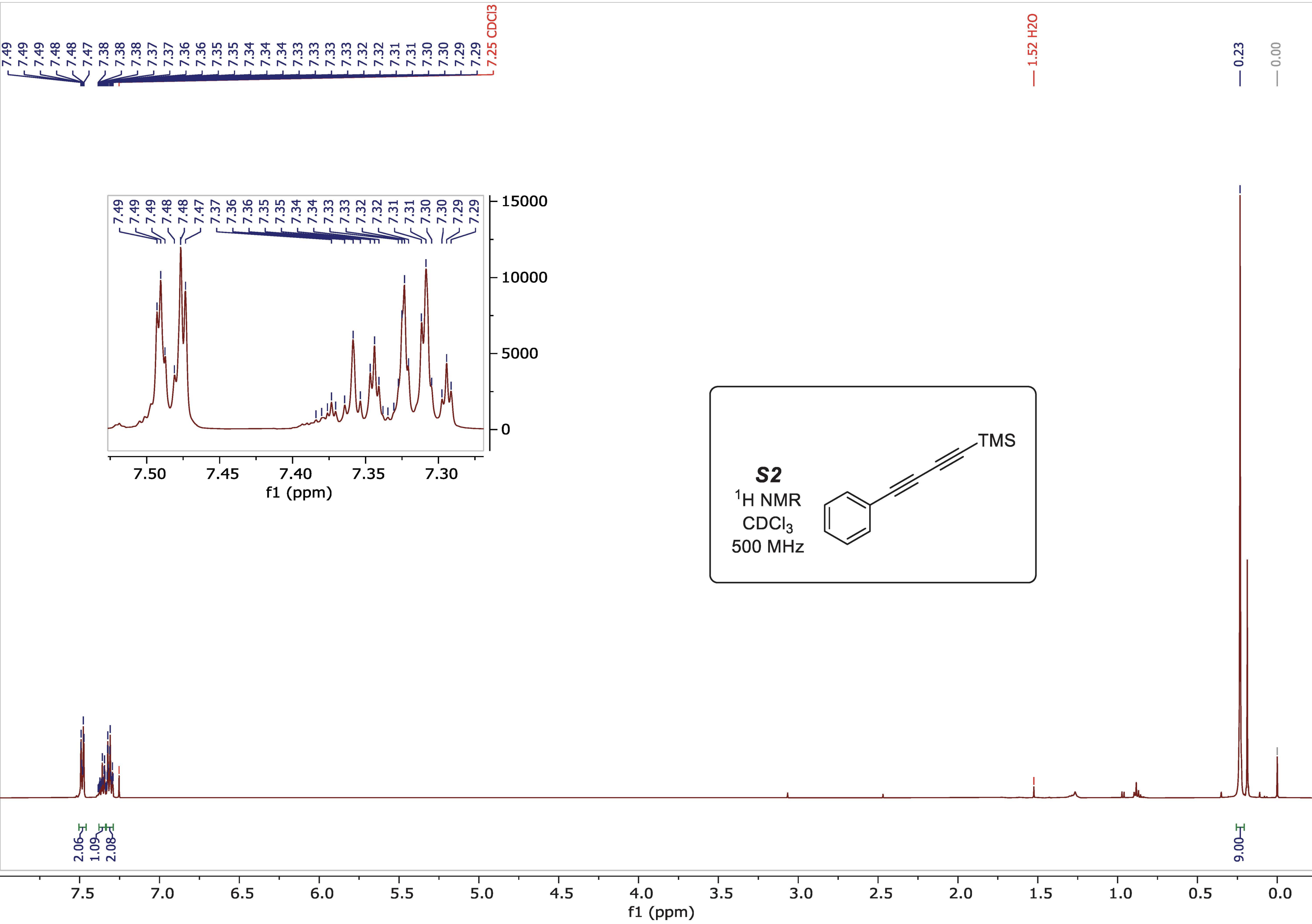
S2
^{1}H NMR
$CDCl_3$
500 MHz
TMS
7.25 CDCl3
1.52 H2O
0.23
0.00
2.06
1.09
2.08
9.00
f1 (ppm)

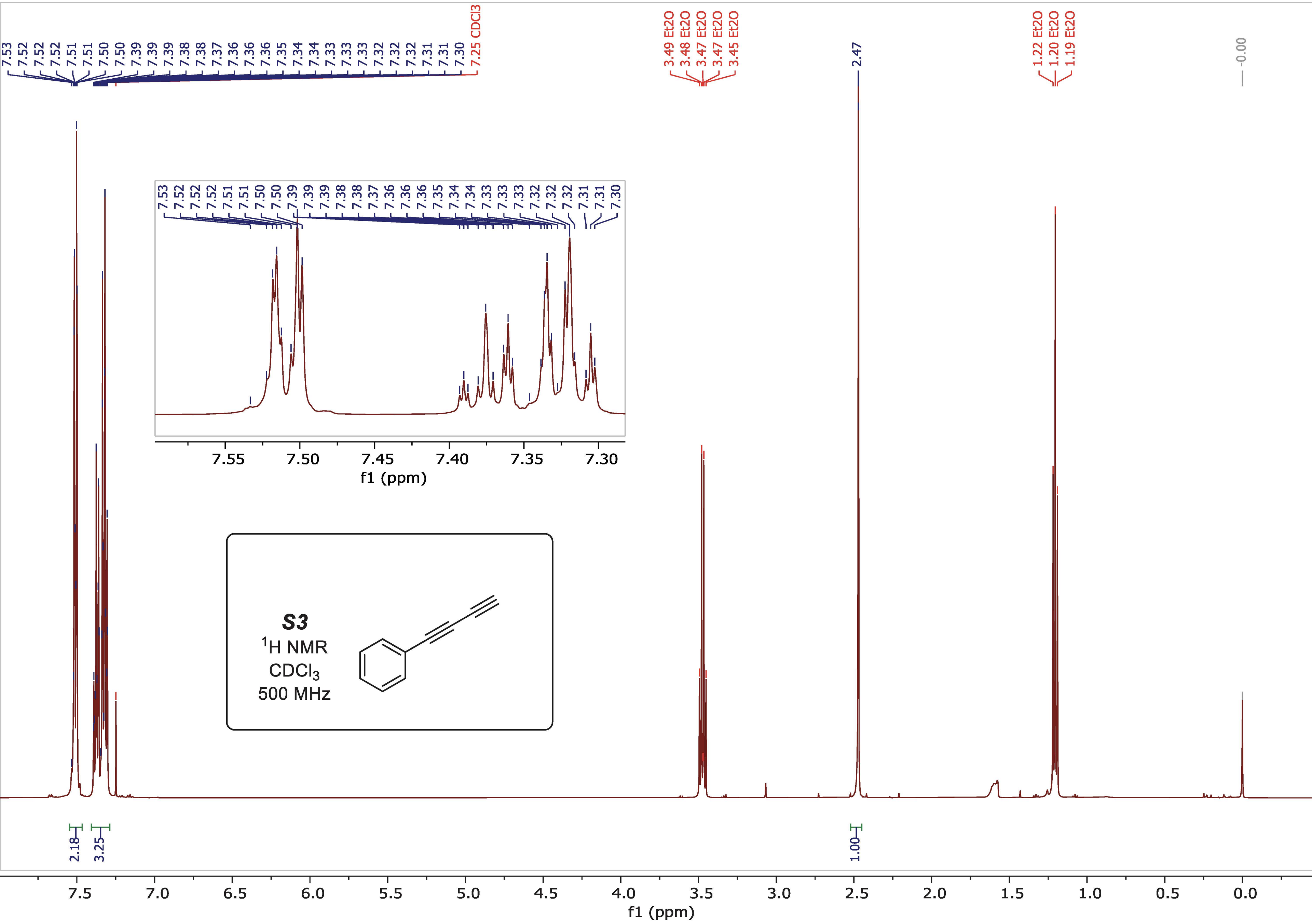
7.53 7.52 7.52 7.52 7.51 7.51 7.50 7.50 7.39 7.39 7.39 7.38 7.38 7.37 7.36 7.36 7.36 7.35 7.34 7.34 7.33 7.33 7.33 7.32 7.32 7.32 7.31 7.31 7.30
7.25 CDCl3
3.49 Et2O 3.48 Et2O 3.47 Et2O 3.47 Et2O 3.45 Et2O
2.47
1.22 Et2O 1.20 Et2O 1.19 Et2O
-0.00
7.55 7.50 7.45 7.40 7.35 7.30
f1 (ppm)
S3
1H NMR
CDCl3
500 MHz
2.18 3.25 1.00
7.5 7.0 6.5 6.0 5.5 5.0 4.5 4.0 3.5 3.0 2.5 2.0 1.5 1.0 0.5 0.0
f1 (ppm)

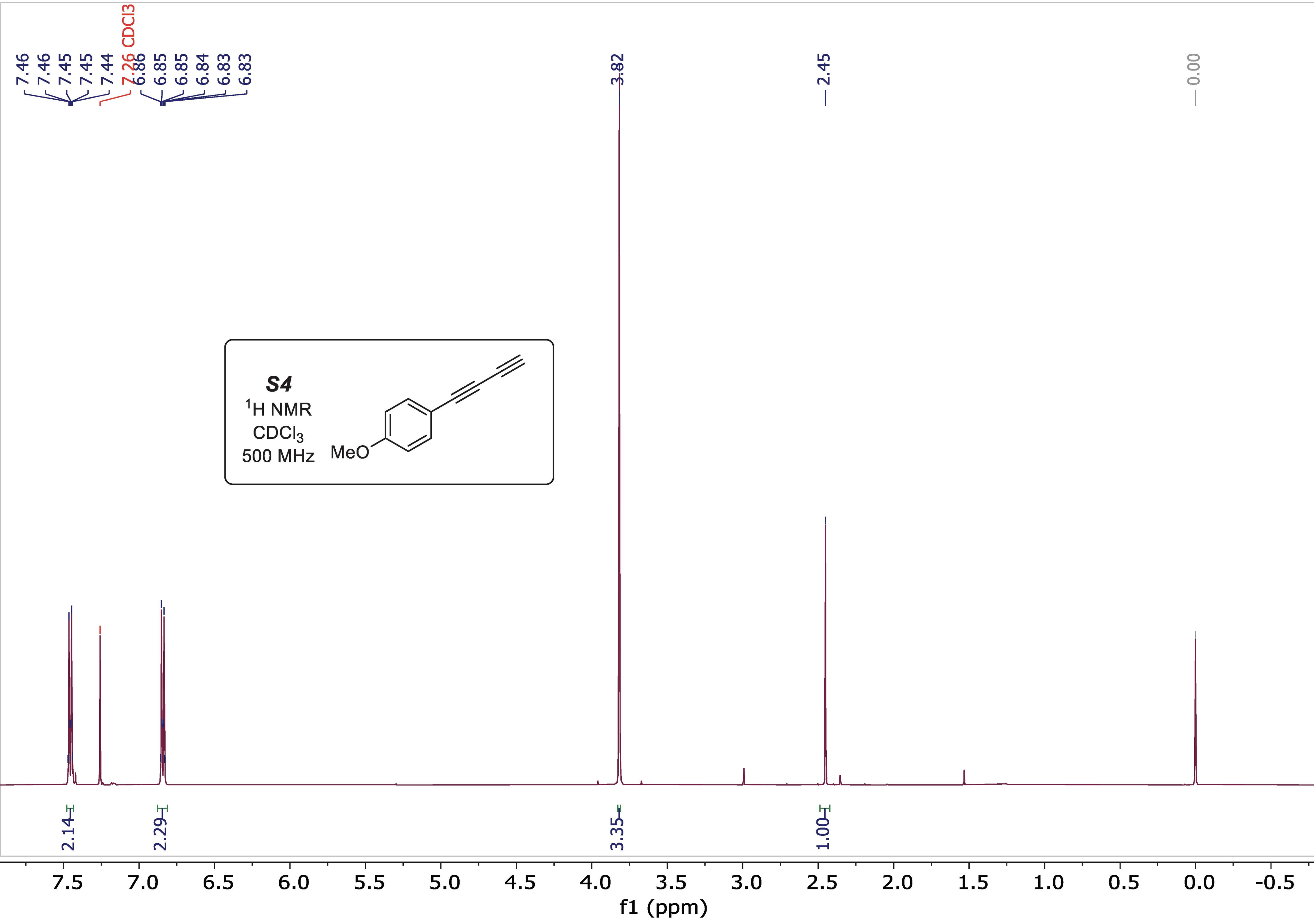
7.46
7.46
7.45
7.45
7.44
7.26 CDCl3
6.86
6.85
6.85
6.84
6.83
6.83
3.82
2.45
0.00
S4
1H NMR
CDCl3
500 MHz
MeO
2.14
2.29
3.35
1.00
7.5 7.0 6.5 6.0 5.5 5.0 4.5 4.0 3.5 3.0 2.5 2.0 1.5 1.0 0.5 0.0 -0.5
f1 (ppm)

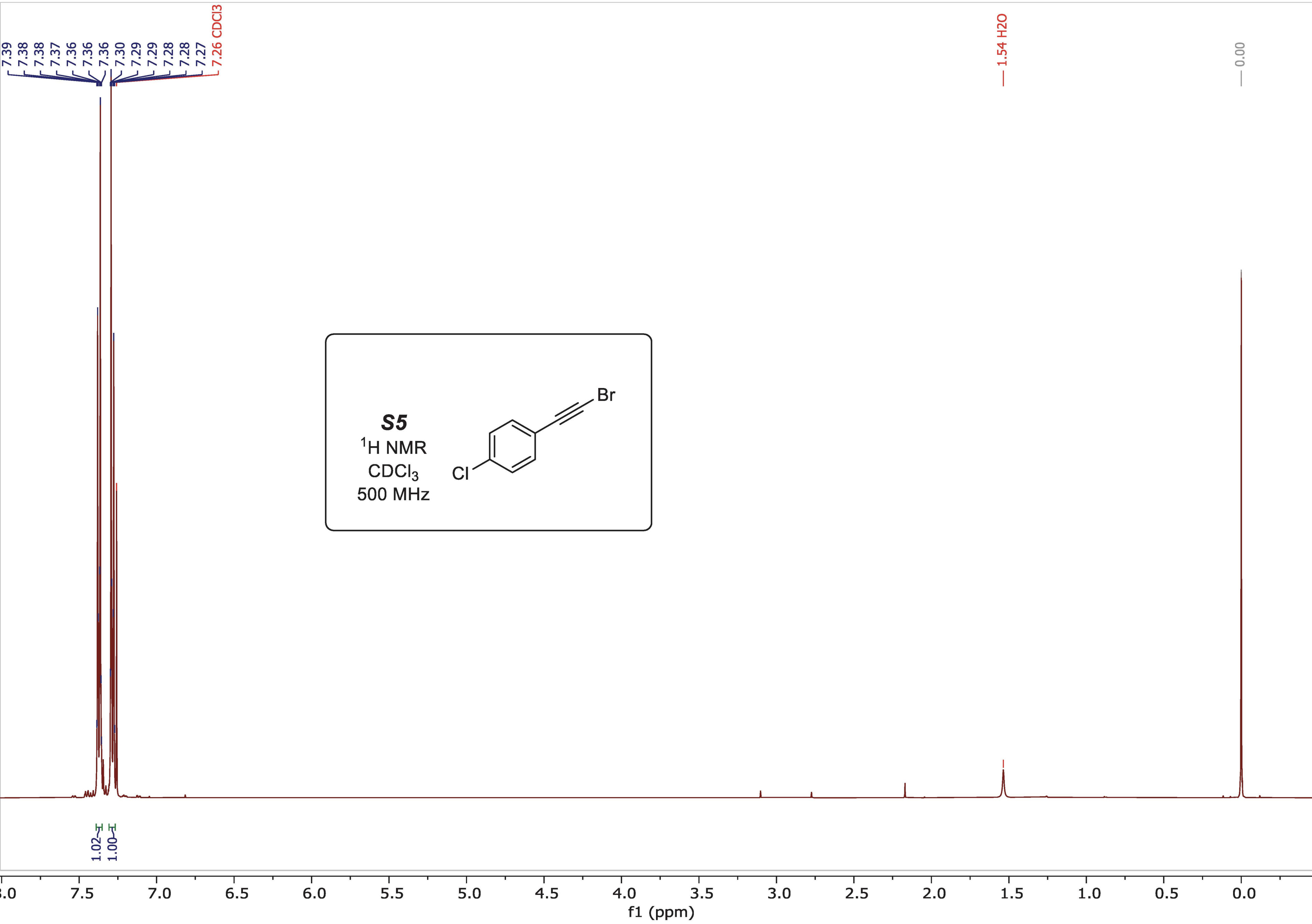
7.39
7.38
7.38
7.37
7.36
7.36
7.36
7.30
7.29
7.29
7.28
7.28
7.27
7.26 CDCl3
1.54 H2O
0.00
S5
1H NMR
CDCl3
500 MHz
Br
Cl
1.02
1.00
7.5
7.0
6.5
6.0
5.5
5.0
4.5
4.0
3.5
3.0
2.5
2.0
1.5
1.0
0.5
0.0
f1 (ppm)

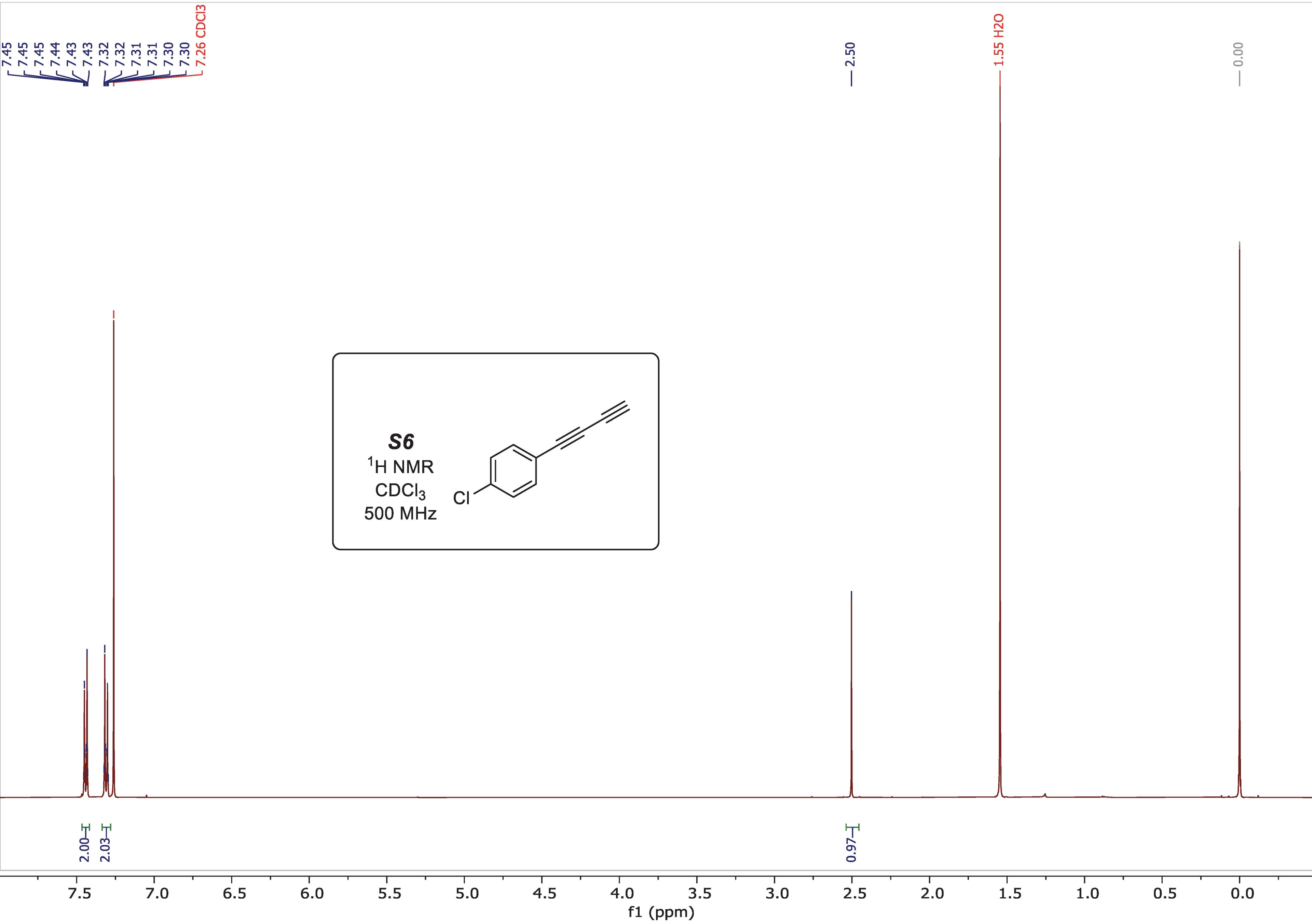
7.45
7.45
7.45
7.44
7.43
7.43
7.32
7.32
7.31
7.31
7.30
7.30
7.26 CDCl3
2.50
1.55 H2O
0.00
S6
1H NMR
CDCl3
500 MHz
Cl
2.00
2.03
0.97
7.5
7.0
6.5
6.0
5.5
5.0
4.5
4.0
3.5
3.0
2.5
2.0
1.5
1.0
0.5
0.0
f1 (ppm)

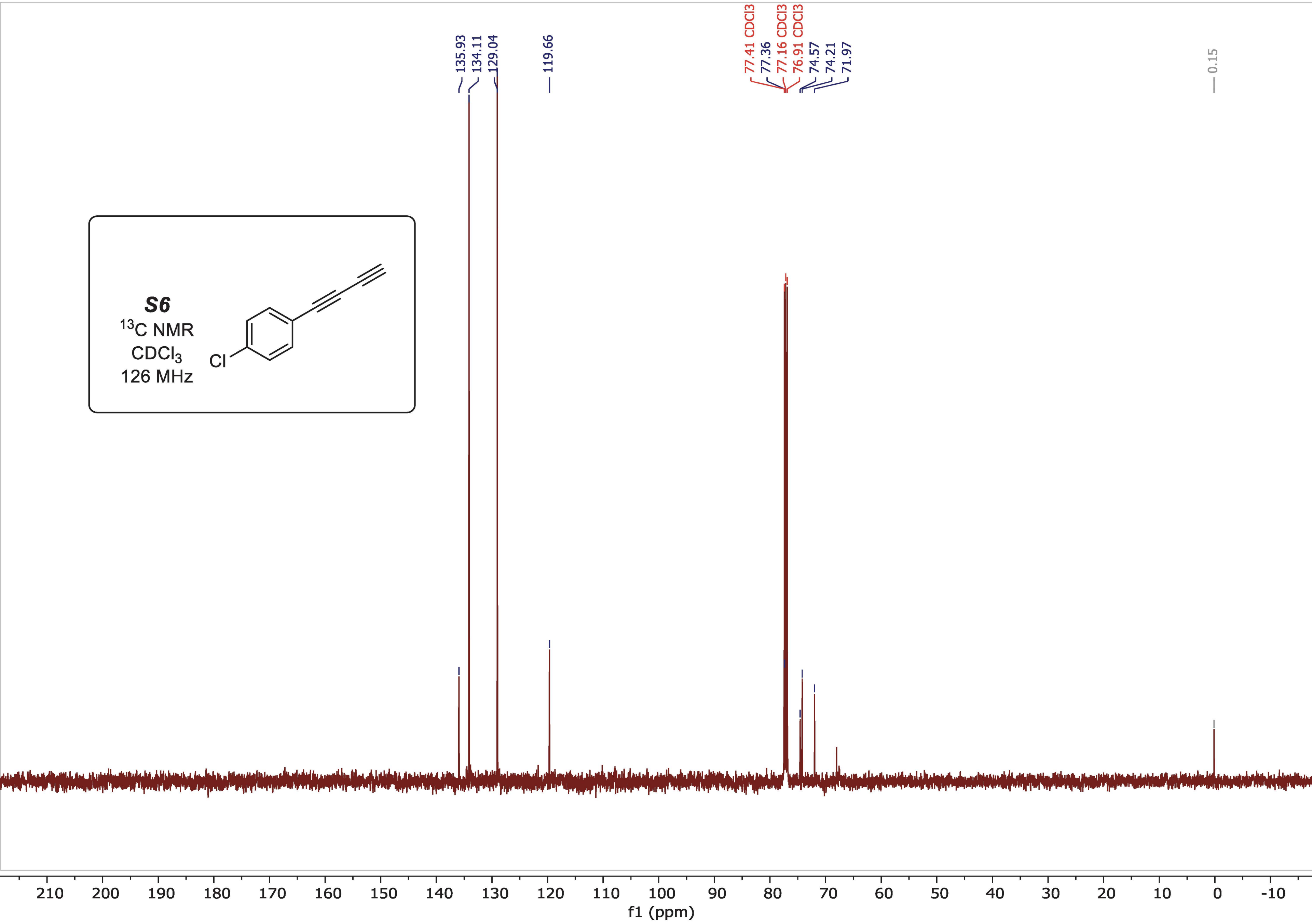
135.93
134.11
129.04
119.66
77.41 CDCl3
77.36
77.16 CDCl3
76.91 CDCl3
74.57
74.21
71.97
0.15
S6
^{13}C NMR
CDCl$_3$
126 MHz
Cl
210 200 190 180 170 160 150 140 130 120 110 100 90 80 70 60 50 40 30 20 10 0 -10
f1 (ppm)

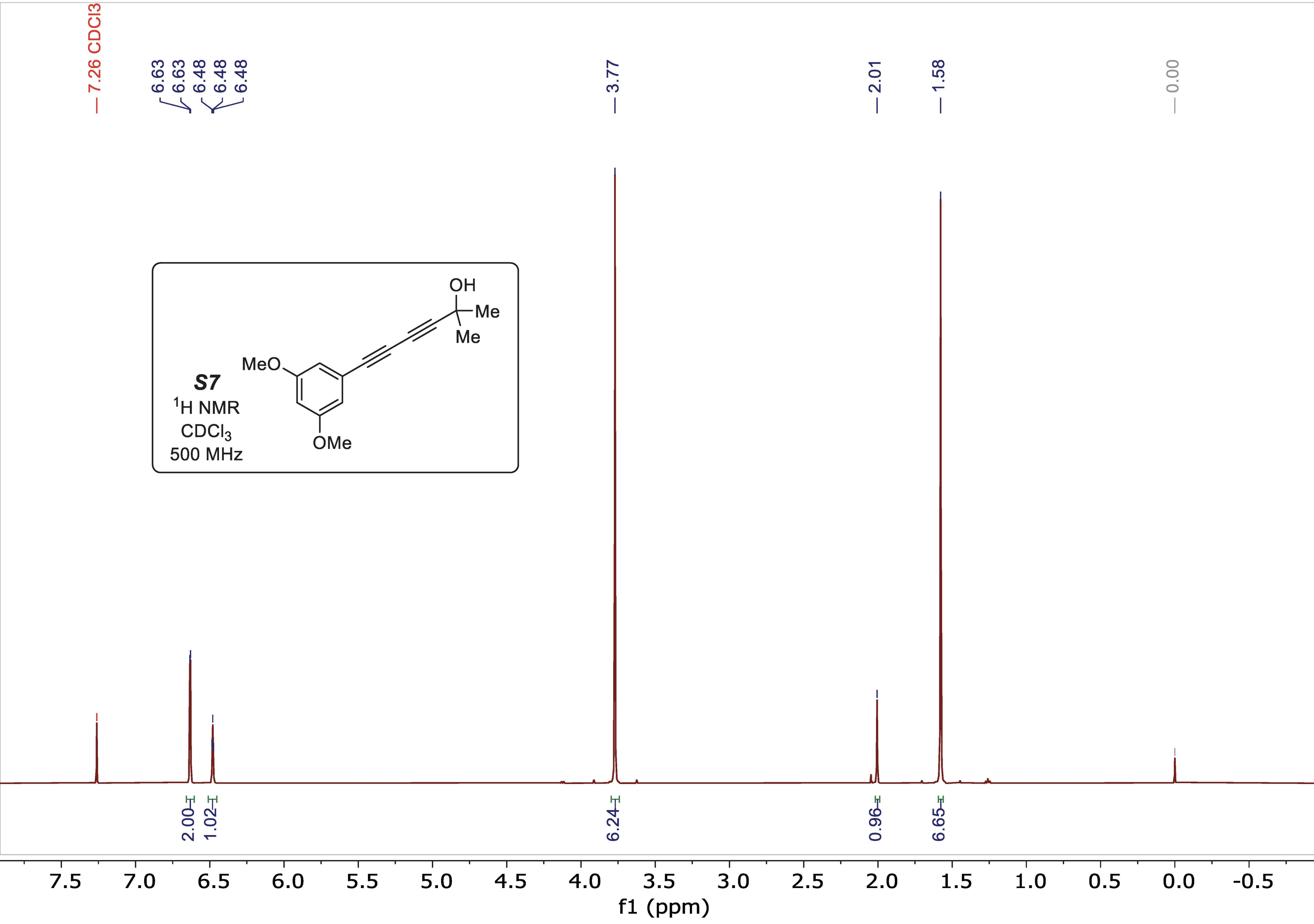
7.26 CDCl3
6.63
6.63
6.48
6.48
6.48
3.77
2.01
1.58
0.00
S7
^{1}H NMR
CDCl$_3$
500 MHz
OH
Me
Me
MeO
OMe
2.00
1.02
6.24
0.96
6.65
7.5
7.0
6.5
6.0
5.5
5.0
4.5
4.0
3.5
3.0
2.5
2.0
1.5
1.0
0.5
0.0
-0.5
f1 (ppm)

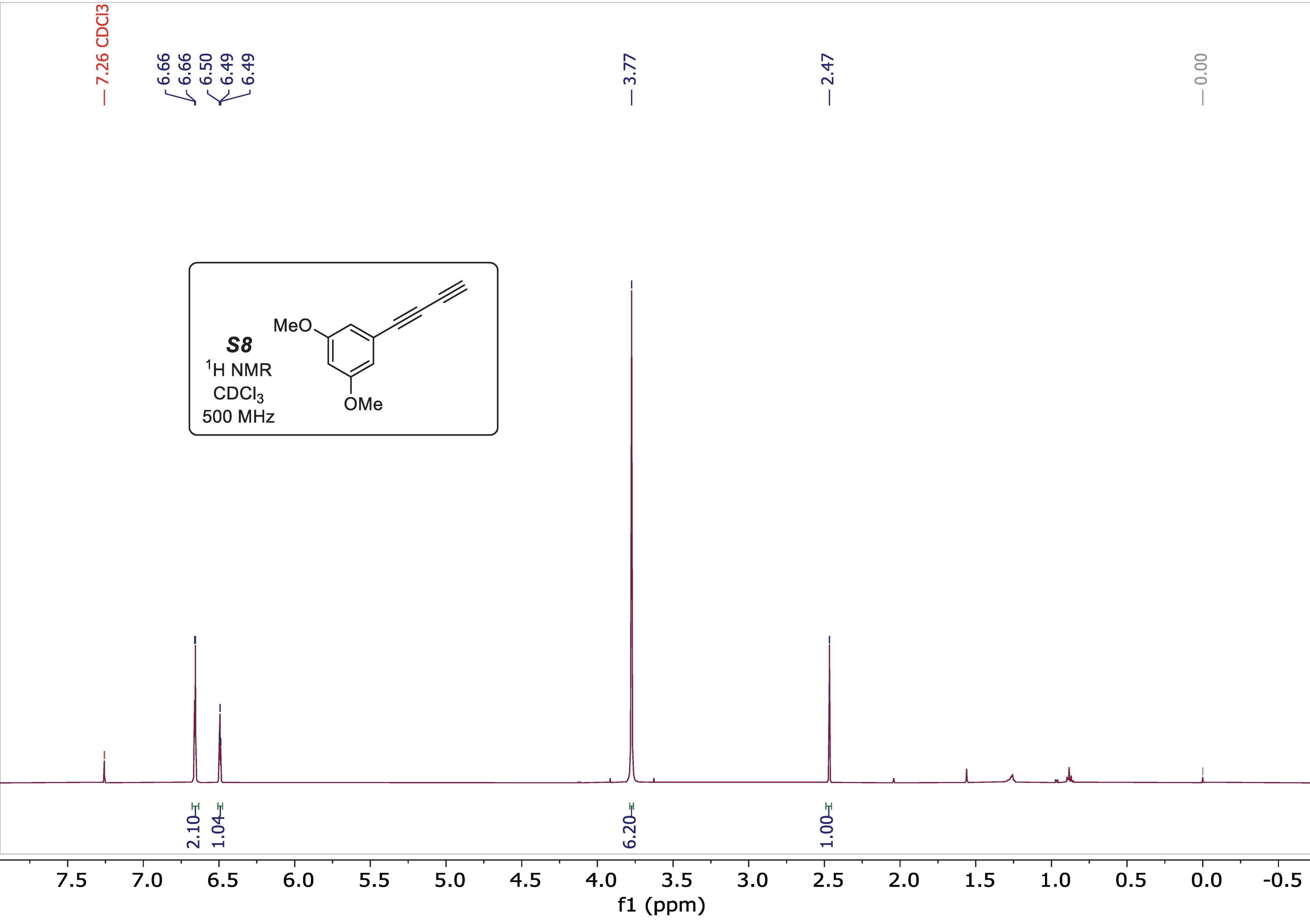
7.26 CDCl3
6.66
6.66
6.50
6.49
6.49
3.77
2.47
0.00
MeO
OMe
S8
1H NMR
CDCl3
500 MHz
2.10
1.04
6.20
1.00
7.5 7.0 6.5 6.0 5.5 5.0 4.5 4.0 3.5 3.0 2.5 2.0 1.5 1.0 0.5 0.0 -0.5
f1 (ppm)

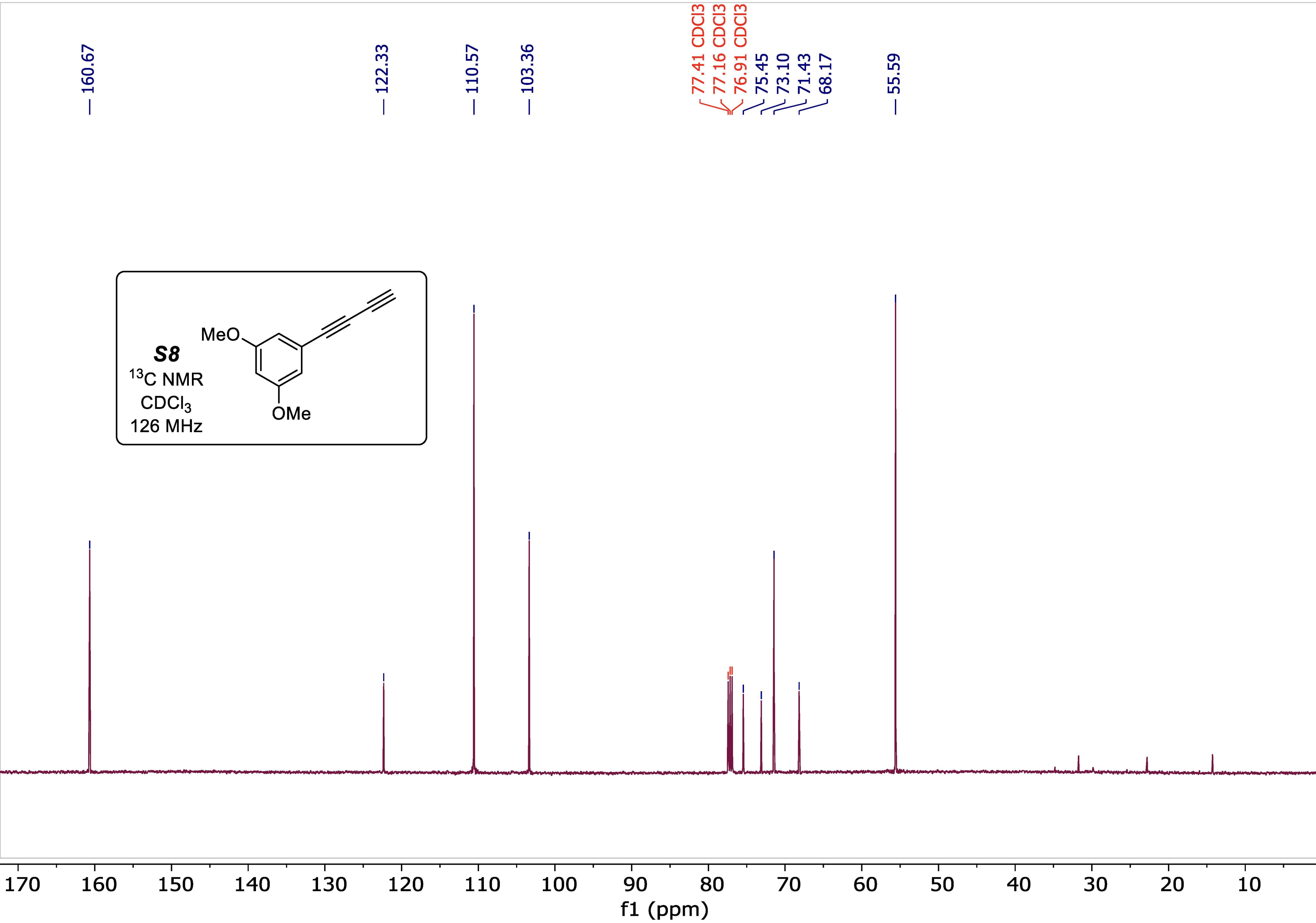
160.67
122.33
110.57
103.36
77.41 CDCl3
77.16 CDCl3
76.91 CDCl3
75.45
73.10
71.43
68.17
55.59
MeO
OMe
S8
13C NMR
CDCl3
126 MHz
170 160 150 140 130 120 110 100 90 80 70 60 50 40 30 20 10
f1 (ppm)

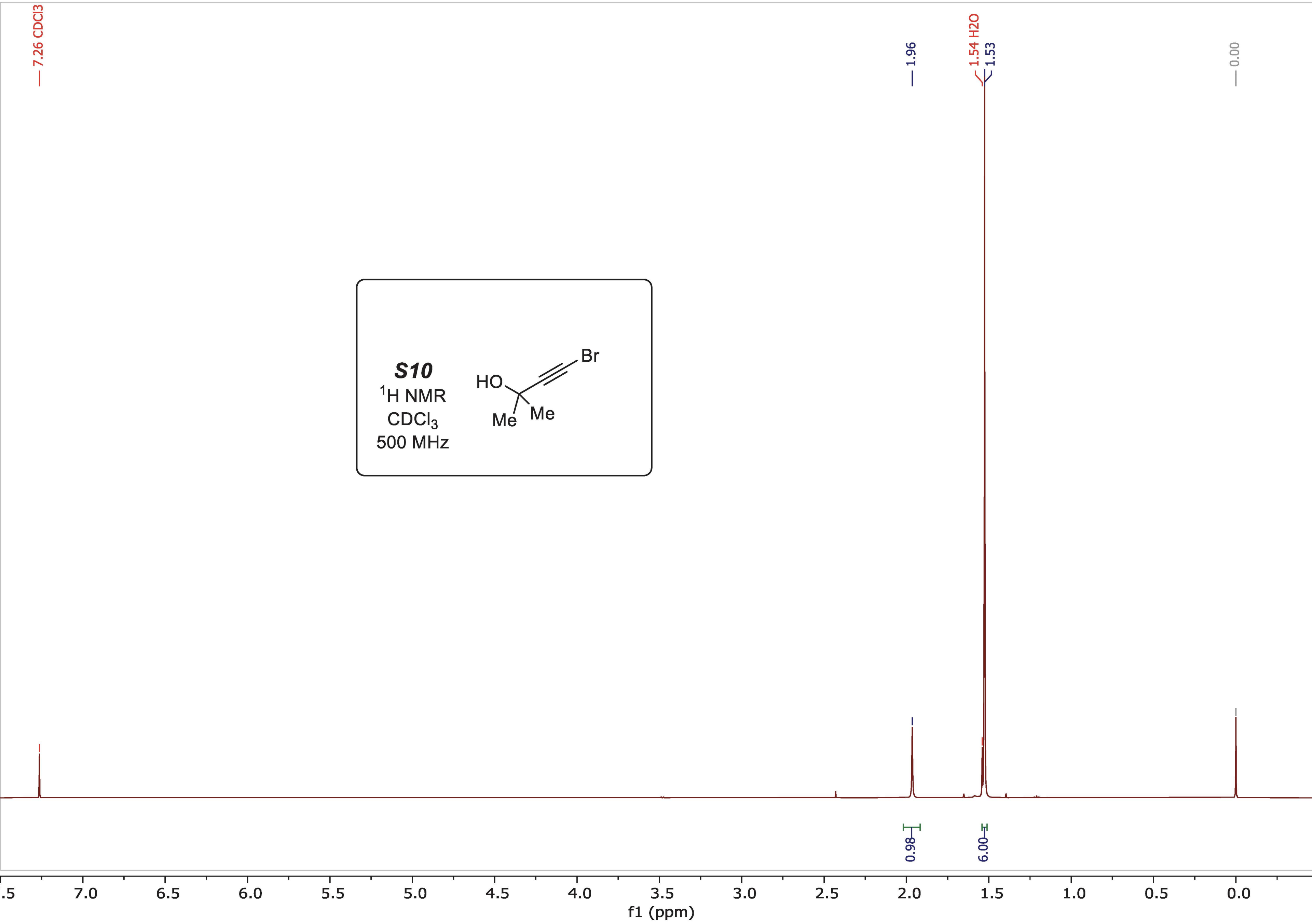
7.26 CDCl3
1.96
1.54 H2O
1.53
0.00
S10
1H NMR
CDCl3
500 MHz
HO
Br
Me
Me
0.98
6.00
7.0
6.5
6.0
5.5
5.0
4.5
4.0
3.5
3.0
2.5
2.0
1.5
1.0
0.5
0.0
f1 (ppm)

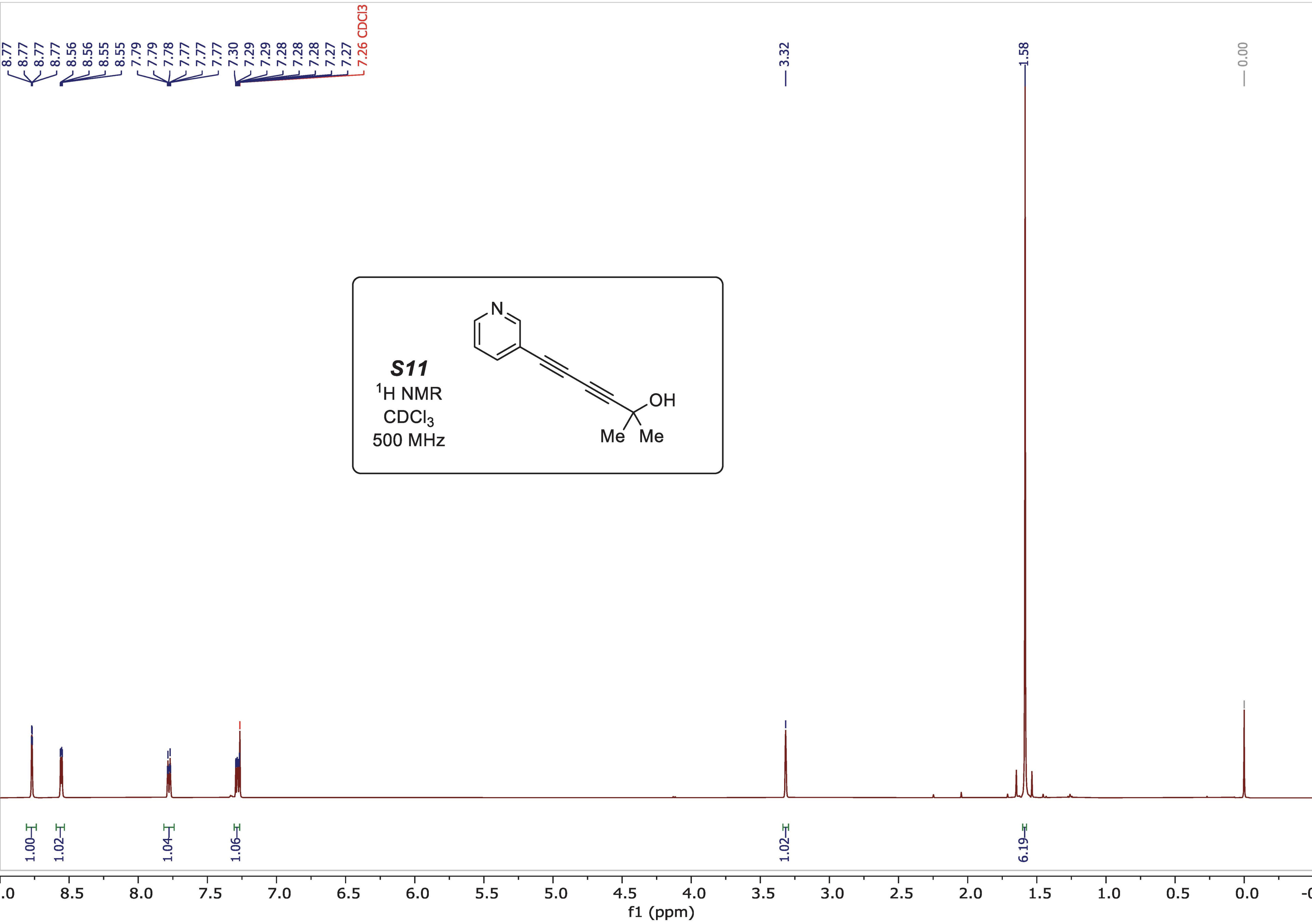
8.77 8.77 8.77 8.77 8.56 8.56 8.55 8.55 7.79 7.79 7.78 7.77 7.77 7.77 7.30 7.29 7.29 7.28 7.28 7.28 7.27 7.27
7.26 CDCl3
3.32
1.58
0.00
S11
1H NMR
CDCl3
500 MHz
N
OH
Me
Me
1.00
1.02
1.04
1.06
1.02
6.19
8.5 8.0 7.5 7.0 6.5 6.0 5.5 5.0 4.5 4.0 3.5 3.0 2.5 2.0 1.5 1.0 0.5 0.0
f1 (ppm)

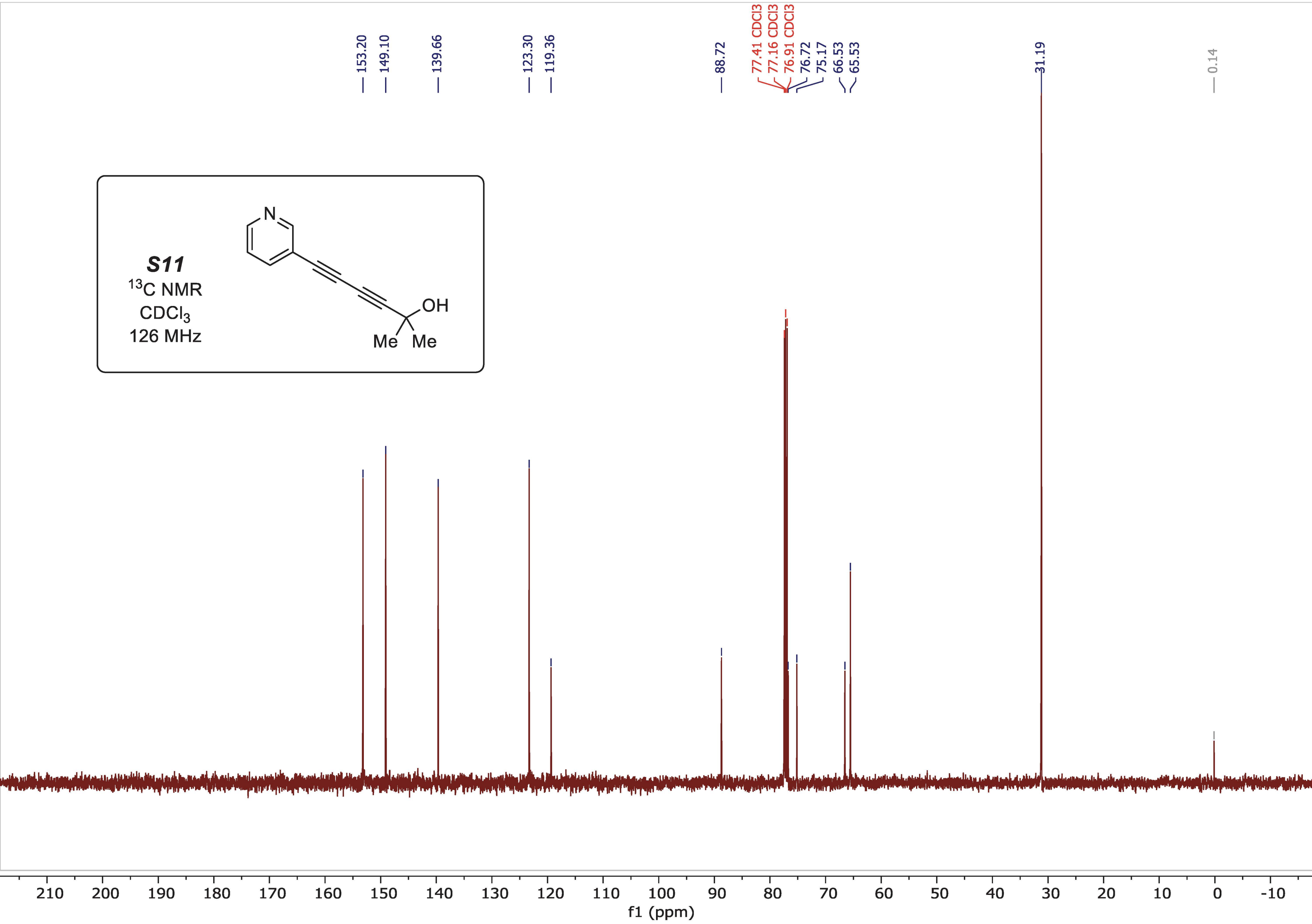
153.20
149.10
139.66
123.30
119.36
88.72
77.41 CDCl3
77.16 CDCl3
76.91 CDCl3
76.72
75.17
66.53
65.53
31.19
0.14
S11
13C NMR
CDCl3
126 MHz
N
OH
Me
Me
f1 (ppm)

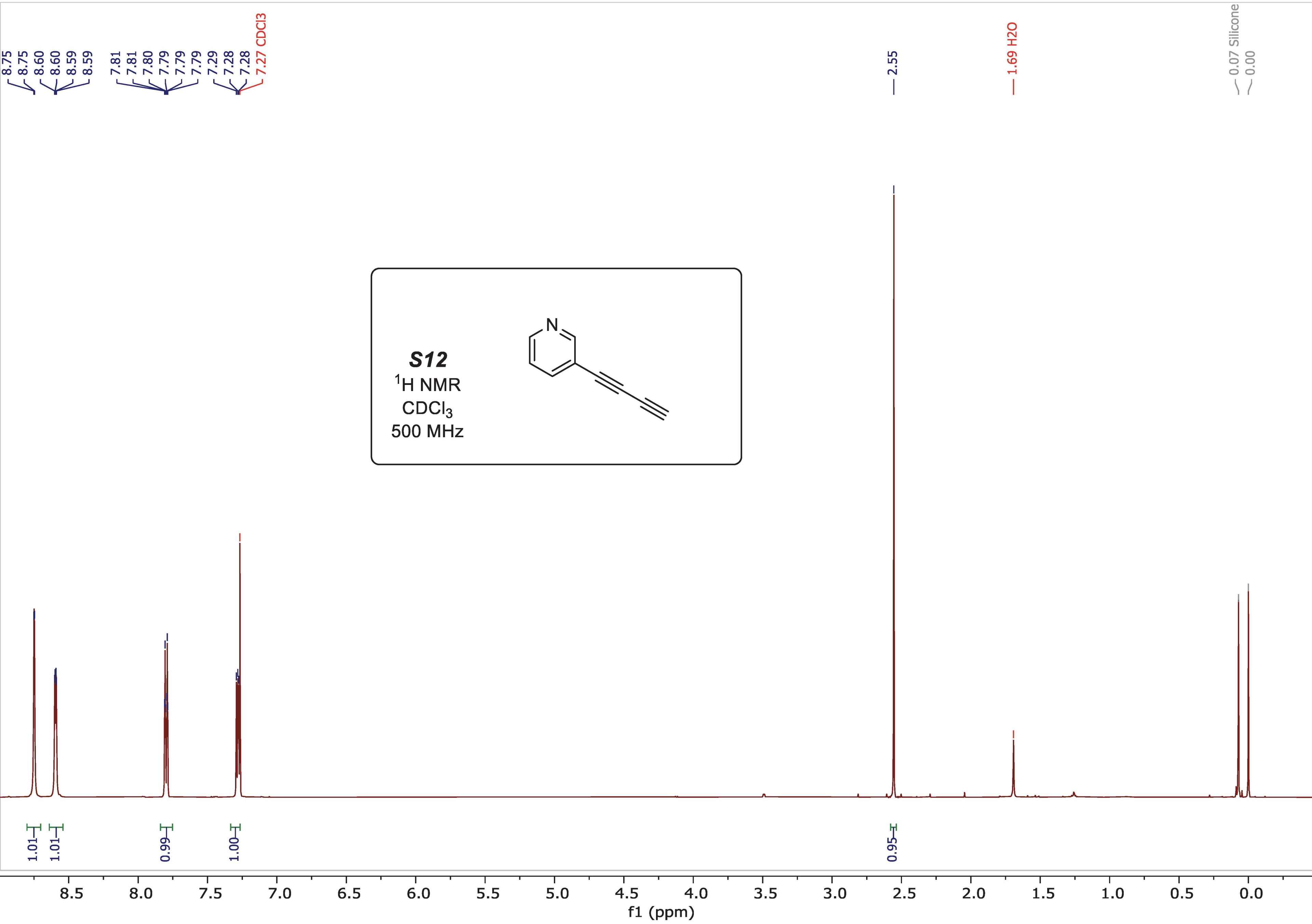
8.75
8.75
8.60
8.60
8.59
8.59
7.81
7.81
7.80
7.79
7.79
7.79
7.29
7.28
7.28
7.27 CDCl3
2.55
1.69 H2O
0.07 Silicone
0.00
S12
1H NMR
CDCl3
500 MHz
N
1.01
1.01
0.99
1.00
0.95
f1 (ppm)

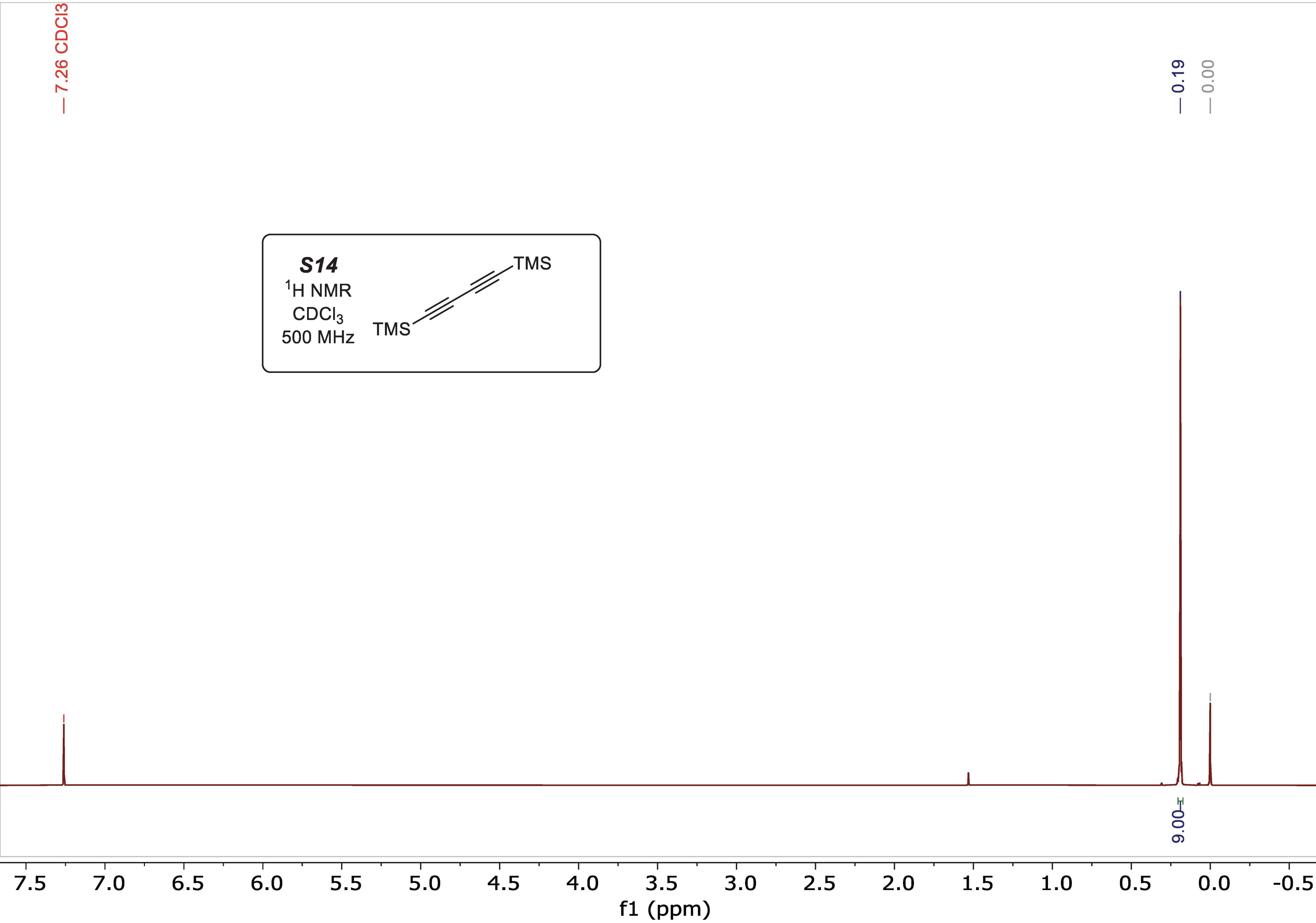

S14
1H NMR
CDCl3
500 MHz
TMS
TMS
7.26 CDCl3
0.19
0.00
9.00
7.5 7.0 6.5 6.0 5.5 5.0 4.5 4.0 3.5 3.0 2.5 2.0 1.5 1.0 0.5 0.0 -0.5
f1 (ppm)

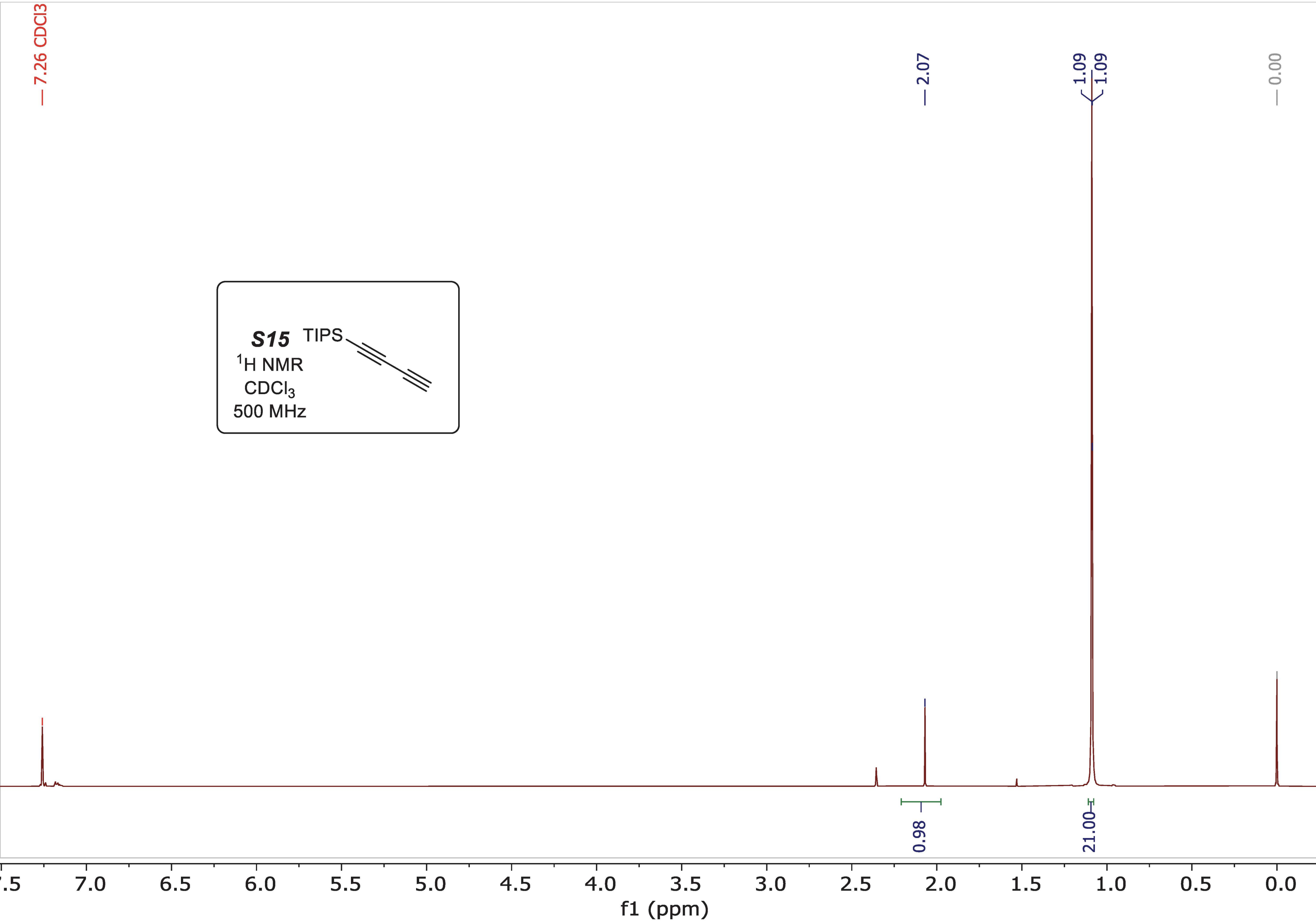
7.26 CDCl3
2.07
1.09
1.09
0.00
S15 TIPS
^{1}H NMR
$CDCl_3$
500 MHz
0.98
21.00
7.0
6.5
6.0
5.5
5.0
4.5
4.0
3.5
3.0
2.5
2.0
1.5
1.0
0.5
0.0
f1 (ppm)

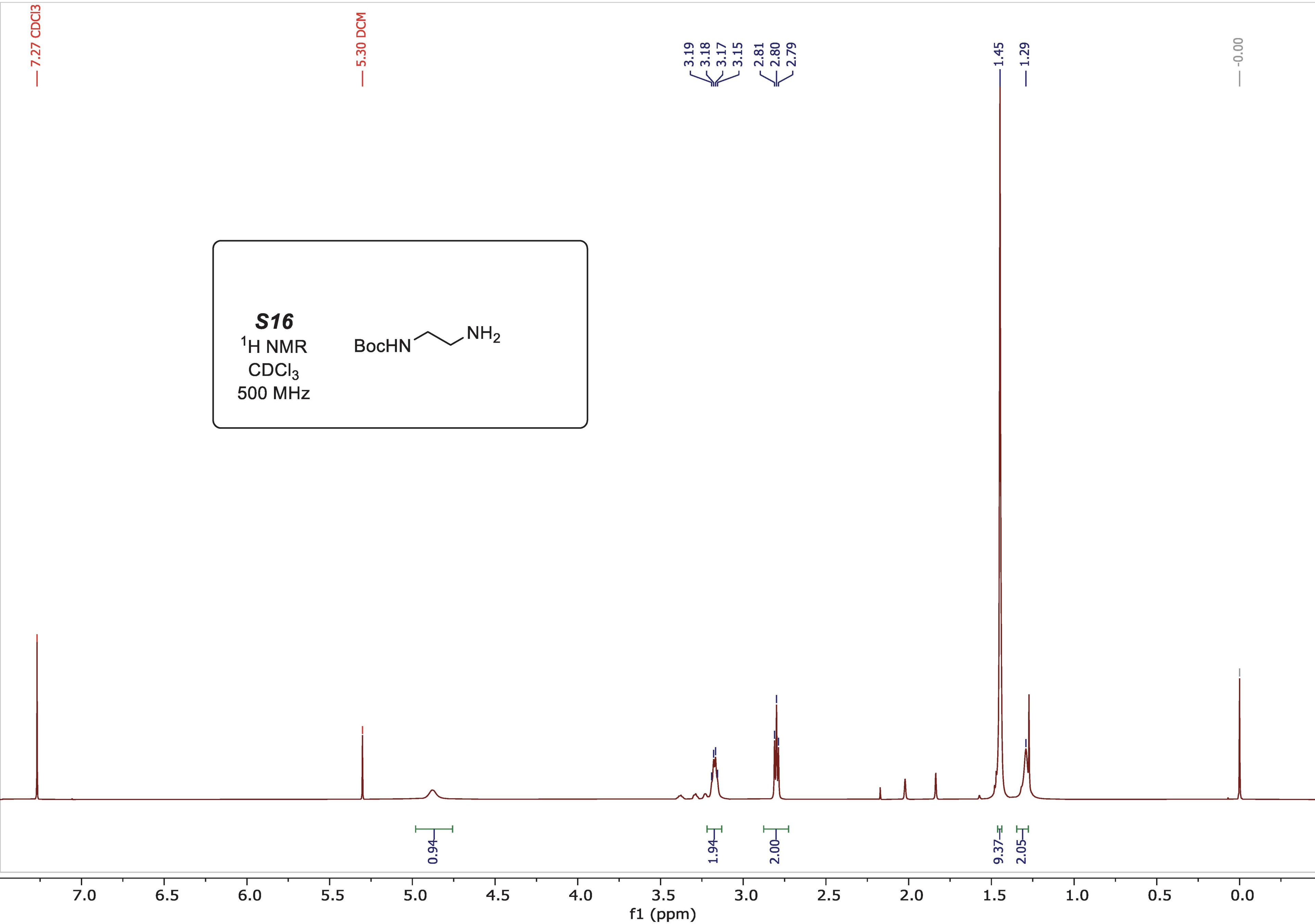
7.27 CDCl3
5.30 DCM
3.19
3.18
3.17
3.15
2.81
2.80
2.79
1.45
1.29
-0.00
S16
1H NMR
CDCl3
500 MHz
BocHN
NH2
0.94
1.94
2.00
9.37
2.05
7.0
6.5
6.0
5.5
5.0
4.5
4.0
3.5
3.0
2.5
2.0
1.5
1.0
0.5
0.0
f1 (ppm)

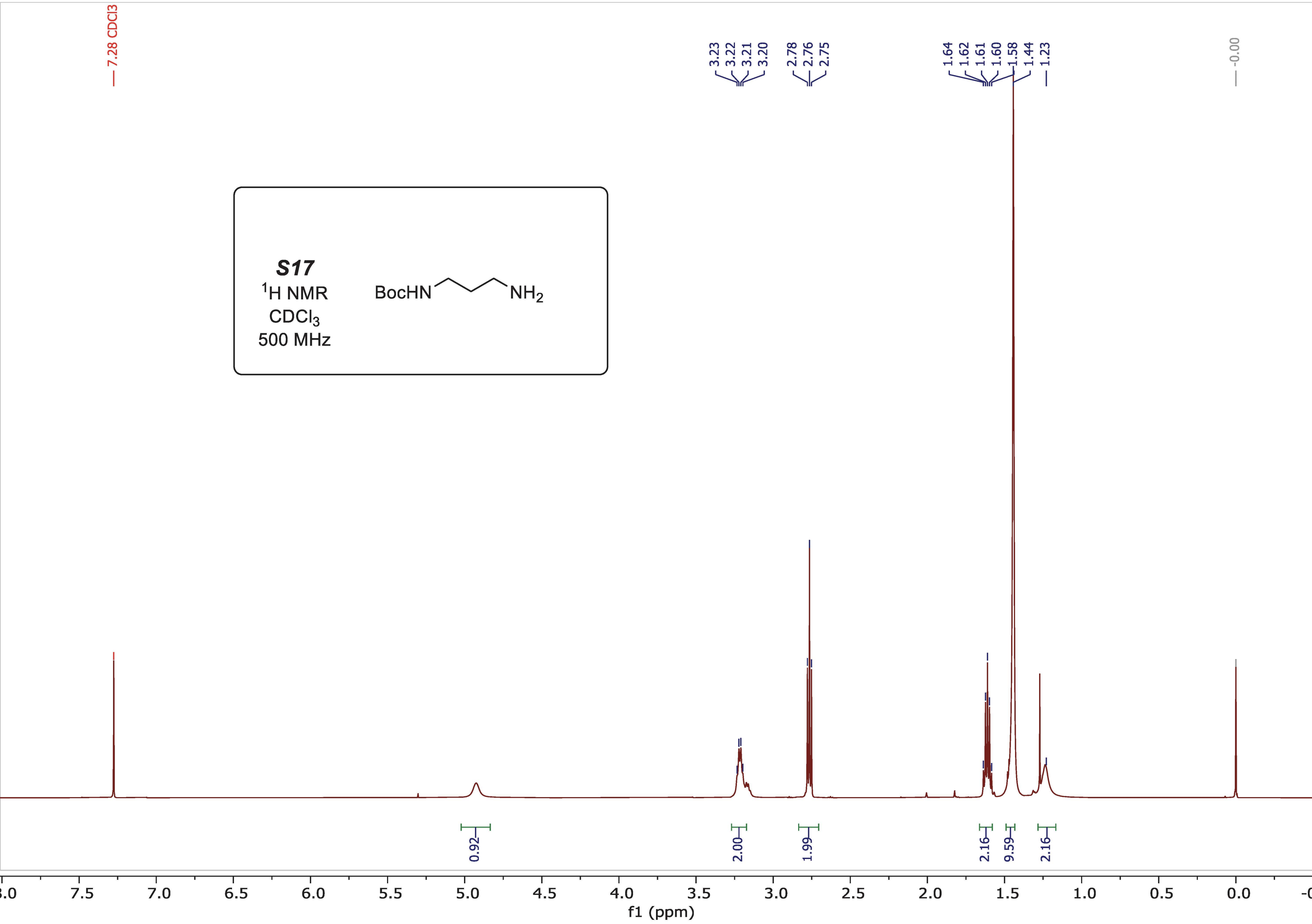
7.28 CDCl3
3.23
3.22
3.21
3.20
2.78
2.76
2.75
1.64
1.62
1.61
1.60
1.58
1.44
1.23
-0.00
S17
1H NMR
CDCl3
500 MHz
BocHN
NH2
0.92
2.00
1.99
2.16
9.59
2.16
f1 (ppm)

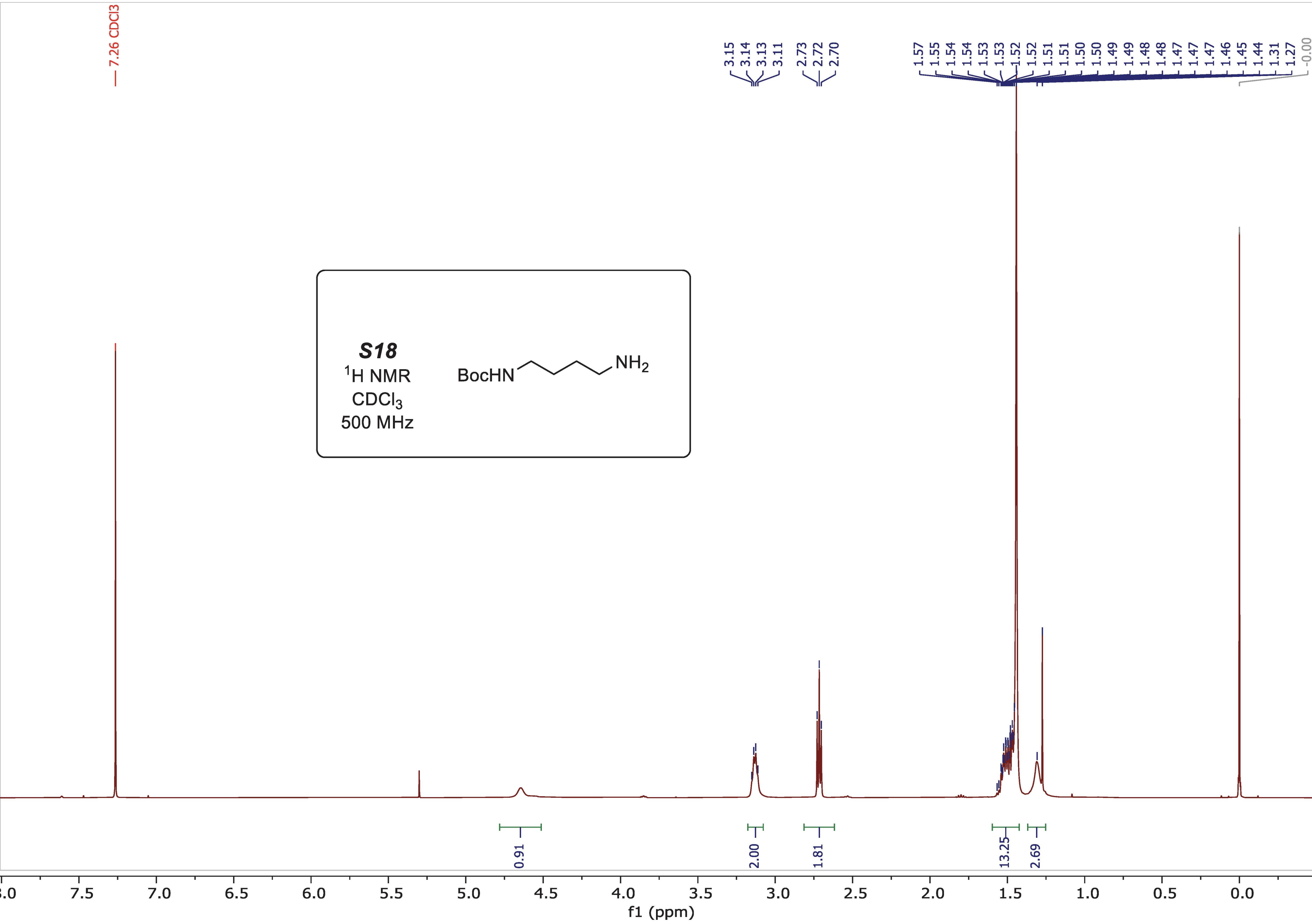
7.26 CDCl3
3.15
3.14
3.13
3.11
2.73
2.72
2.70
1.57
1.55
1.54
1.54
1.53
1.53
1.52
1.52
1.51
1.51
1.50
1.50
1.49
1.49
1.48
1.48
1.47
1.47
1.47
1.46
1.45
1.44
1.31
1.27
-0.00
S18
1H NMR
CDCl3
500 MHz
BocHN
NH2
0.91
2.00
1.81
13.25
2.69
f1 (ppm)

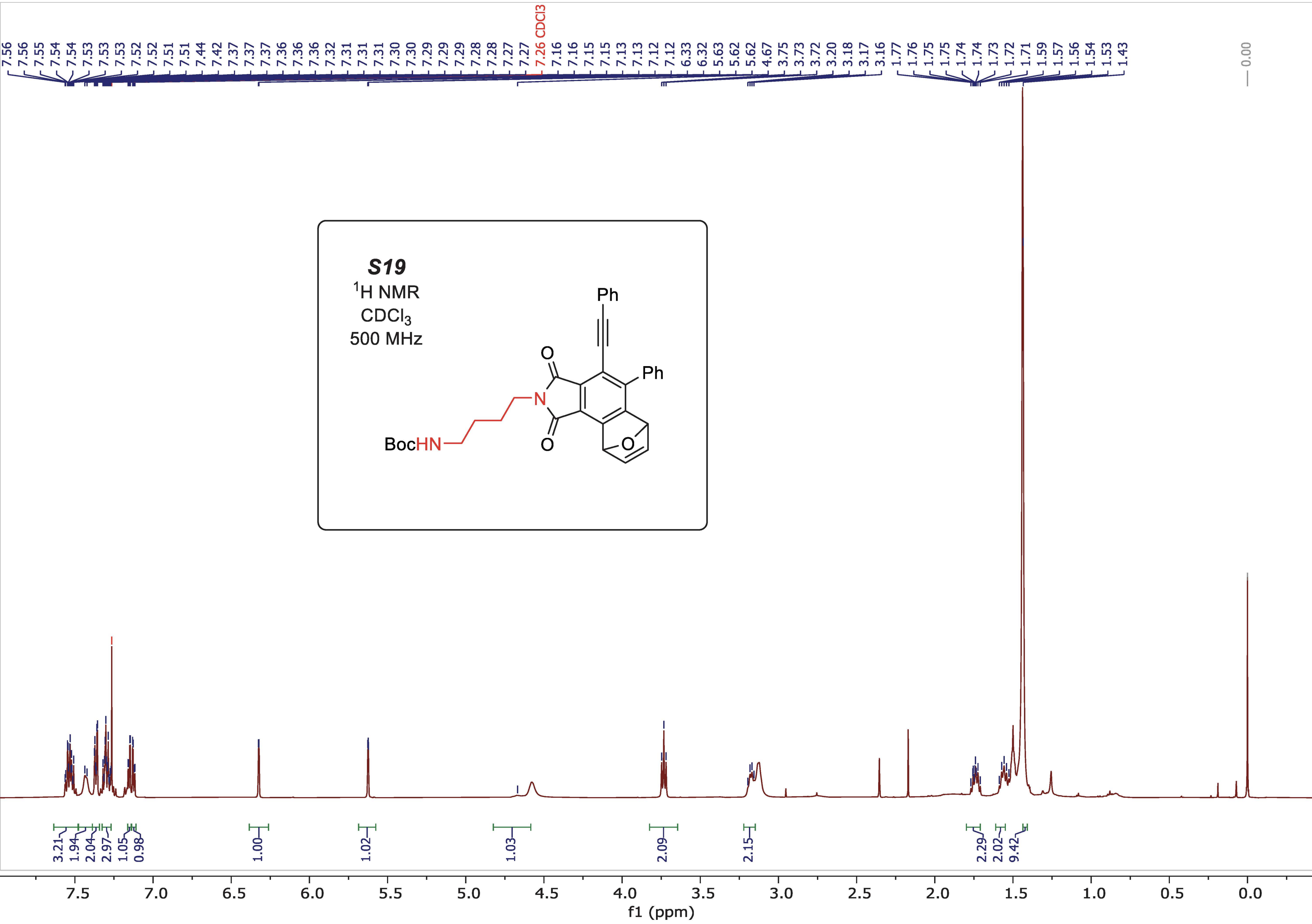
7.56 7.56 7.55 7.54 7.54 7.53 7.53 7.53 7.52 7.52 7.51 7.51 7.44 7.42 7.37 7.37 7.37 7.36 7.36 7.36 7.32 7.31 7.31 7.31 7.30 7.30 7.29 7.29 7.29 7.28 7.28 7.27 7.27 7.26 CDCl3 7.16 7.16 7.15 7.15 7.13 7.13 7.12 7.12 6.33 6.32 5.63 5.62 5.62 4.67 3.75 3.73 3.72 3.20 3.18 3.17 3.16 1.77 1.76 1.75 1.75 1.74 1.74 1.73 1.72 1.71 1.59 1.57 1.56 1.54 1.53 1.43
0.00
S19
1H NMR
CDCl3
500 MHz
Ph
Ph
O
N
O
O
BocHN
3.21 1.94 2.04 2.97 1.05 0.98 1.00 1.02 1.03 2.09 2.15 2.29 2.02 9.42
7.5 7.0 6.5 6.0 5.5 5.0 4.5 4.0 3.5 3.0 2.5 2.0 1.5 1.0 0.5 0.0
f1 (ppm)

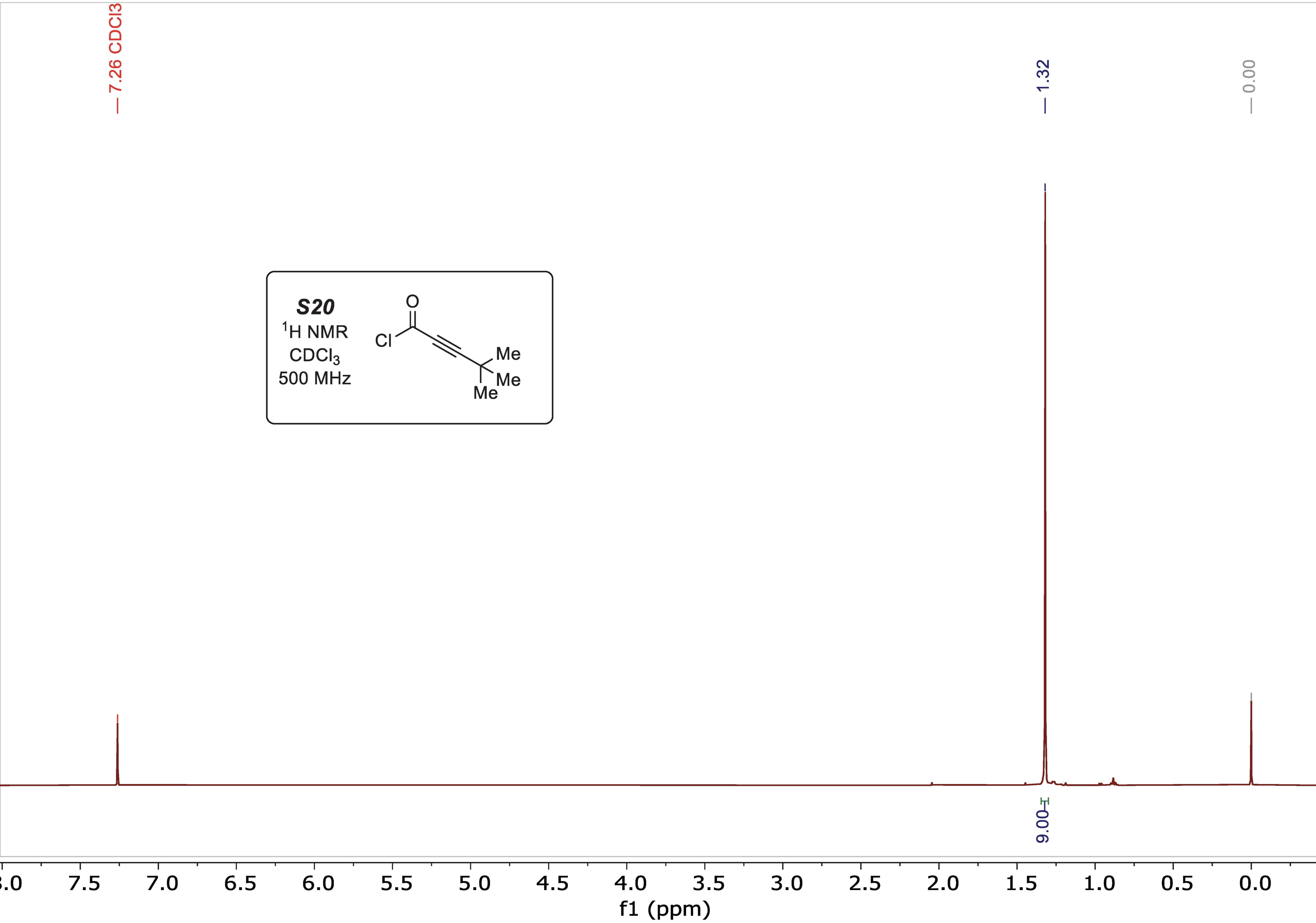

7.26 CDCl3
1.32
0.00
S20
1H NMR
CDCl3
500 MHz
O
Cl
Me
Me
Me
9.00
8.0
7.5
7.0
6.5
6.0
5.5
5.0
4.5
4.0
3.5
3.0
2.5
2.0
1.5
1.0
0.5
0.0
f1 (ppm)

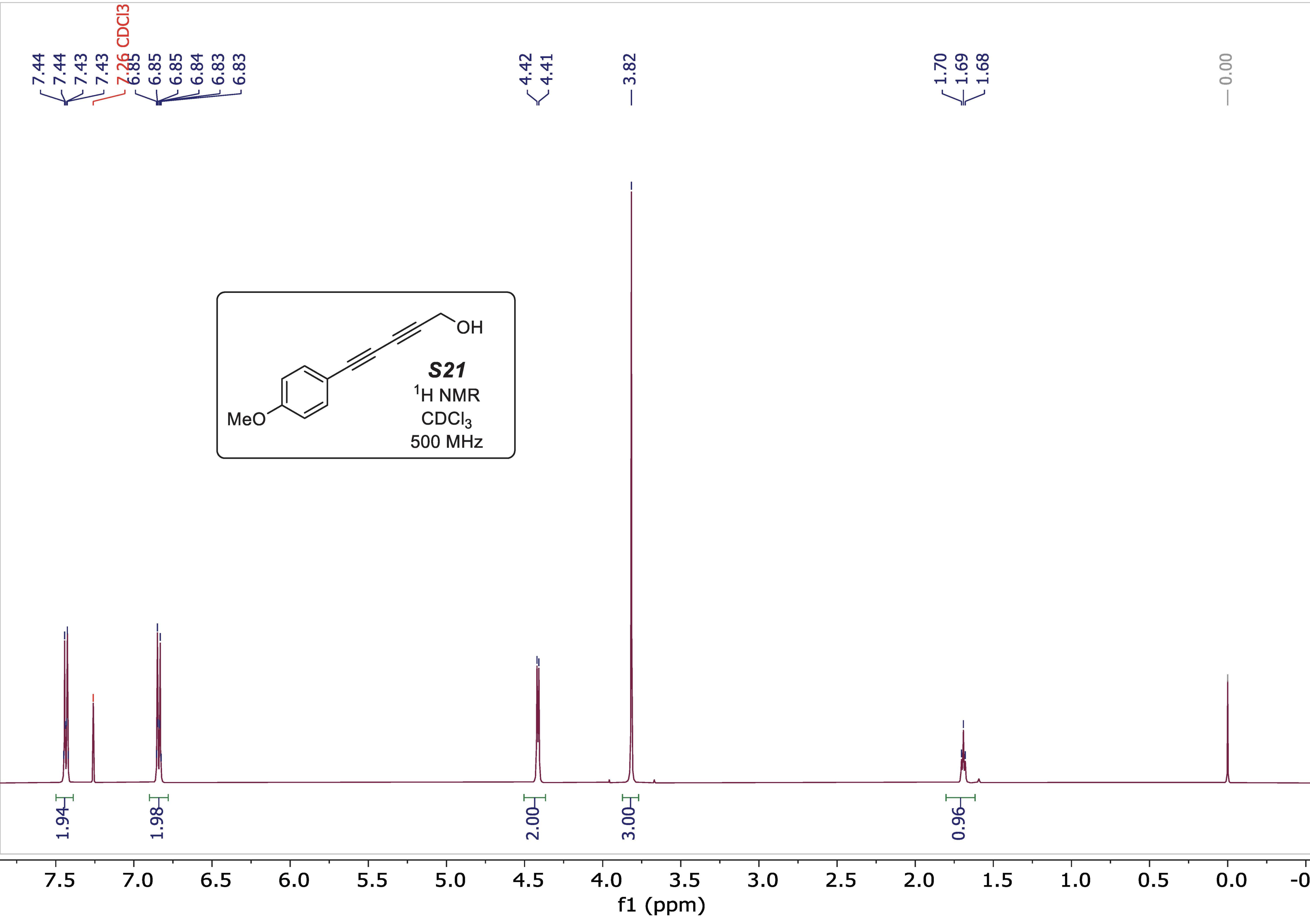
7.44
7.44
7.43
7.43
7.26 CDCl3
6.85
6.85
6.85
6.84
6.83
6.83
4.42
4.41
3.82
1.70
1.69
1.68
0.00
OH
MeO
S21
^{1}H NMR
$CDCl_3$
500 MHz
1.94
1.98
2.00
3.00
0.96
7.5
7.0
6.5
6.0
5.5
5.0
4.5
4.0
3.5
3.0
2.5
2.0
1.5
1.0
0.5
0.0
f1 (ppm)

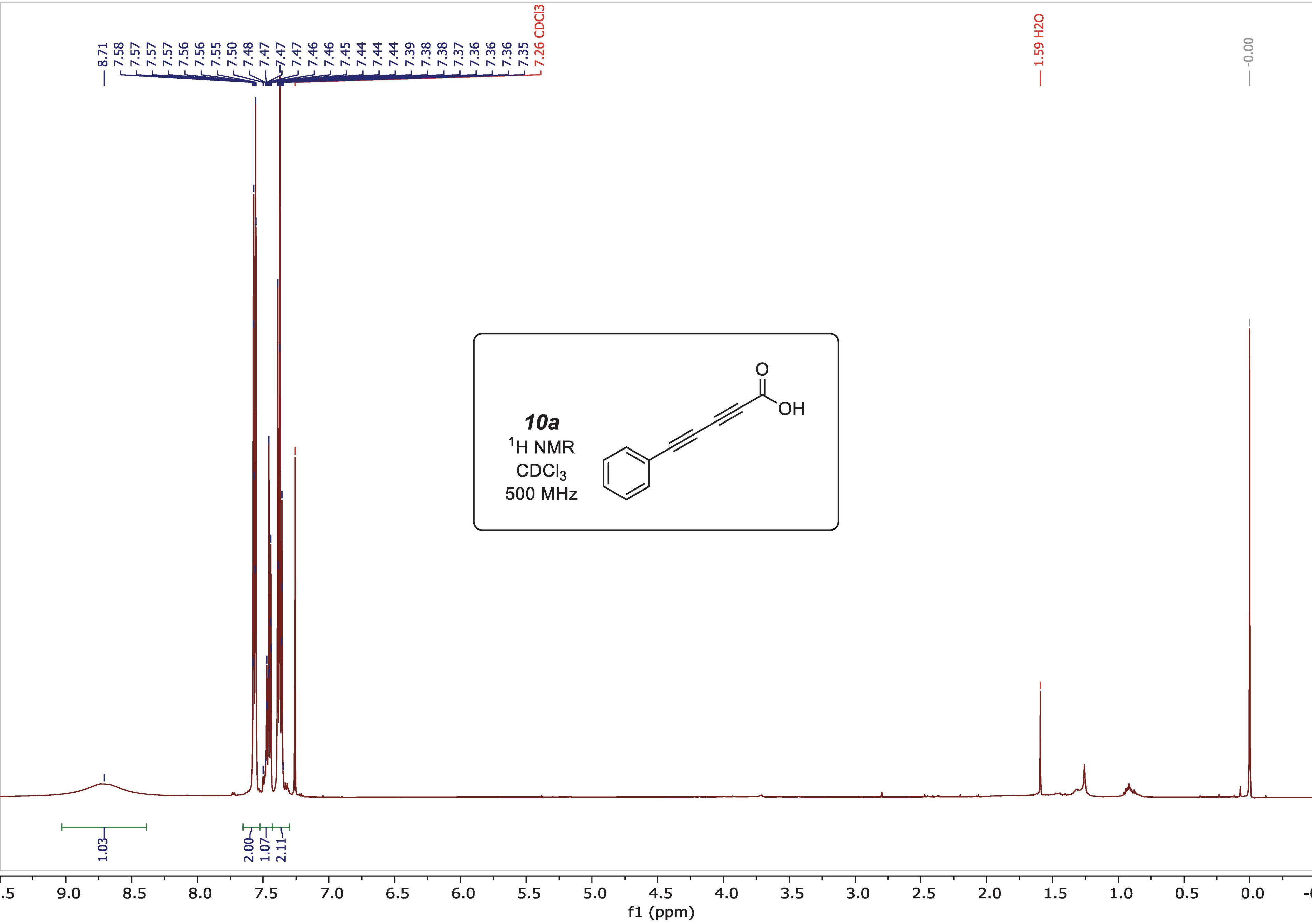
8.71
7.58
7.57
7.57
7.57
7.56
7.56
7.55
7.50
7.48
7.47
7.47
7.47
7.46
7.46
7.45
7.44
7.44
7.44
7.44
7.39
7.38
7.38
7.37
7.36
7.36
7.36
7.35
7.26 CDCl3
1.59 H2O
-0.00
10a
1H NMR
CDCl3
500 MHz
O
OH
1.03
2.00
1.07
2.11
9.0 8.5 8.0 7.5 7.0 6.5 6.0 5.5 5.0 4.5 4.0 3.5 3.0 2.5 2.0 1.5 1.0 0.5 0.0
f1 (ppm)

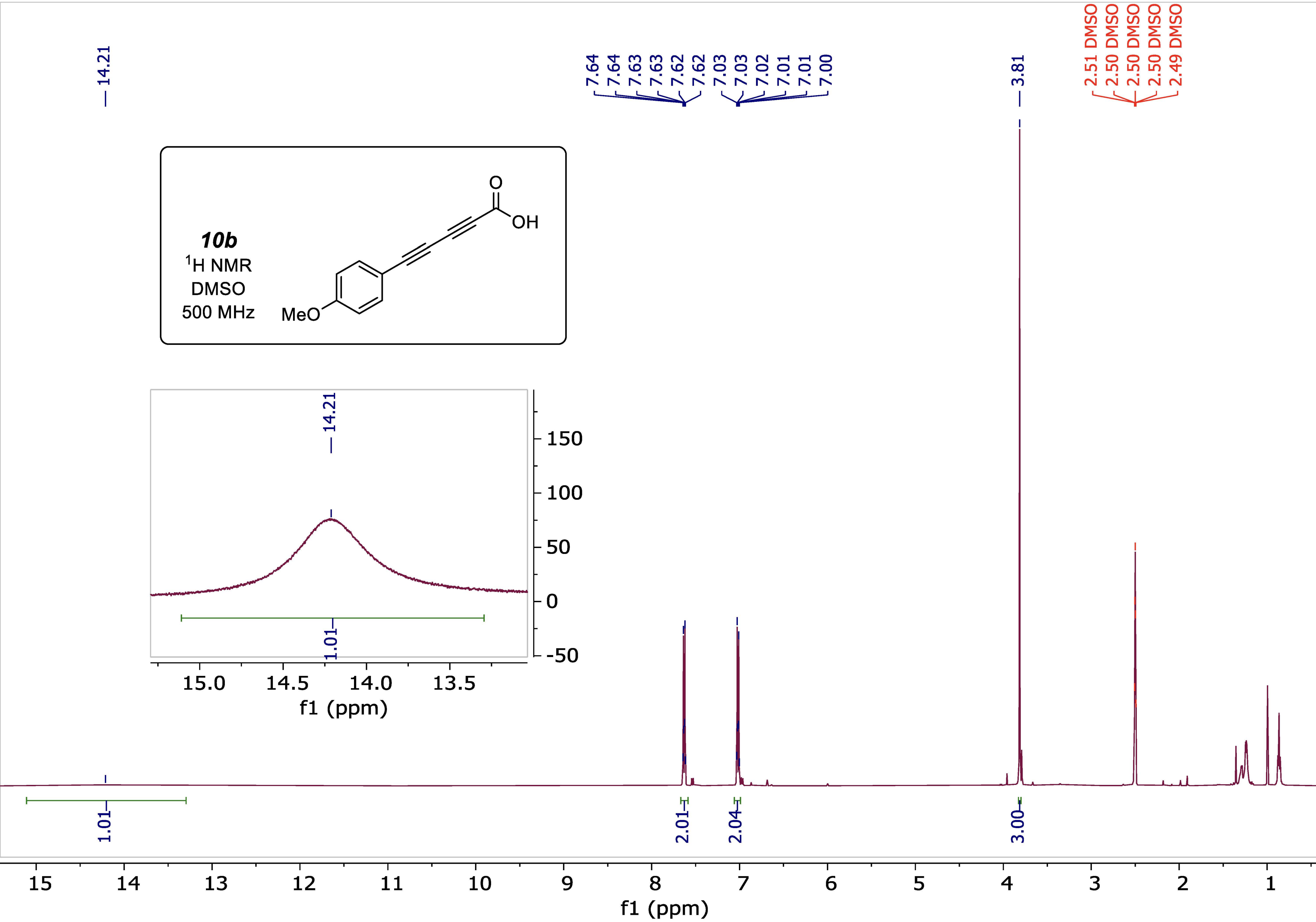

10b
^{1}H NMR
DMSO
500 MHz
MeO
O
OH
14.21
7.64 7.64 7.63 7.63 7.62 7.62 7.03 7.03 7.02 7.01 7.01 7.00
3.81
2.51 DMSO 2.50 DMSO 2.50 DMSO 2.50 DMSO 2.49 DMSO
1.01
2.01
2.04
3.00
f1 (ppm)

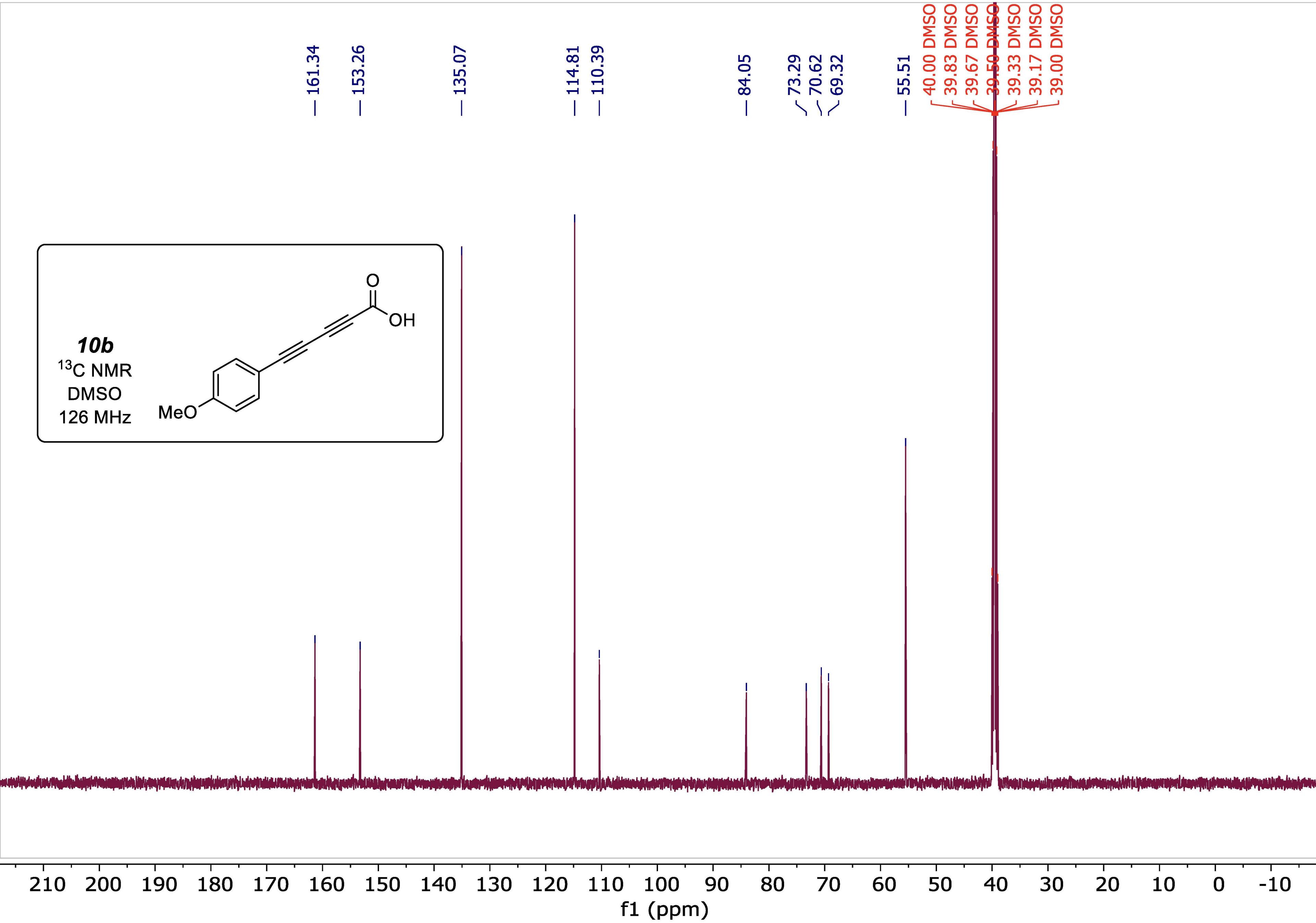
161.34
153.26
135.07
114.81
110.39
84.05
73.29
70.62
69.32
55.51
40.00 DMSO
39.83 DMSO
39.67 DMSO
39.50 DMSO
39.33 DMSO
39.17 DMSO
39.00 DMSO
10b
13C NMR
DMSO
126 MHz
MeO
O
OH
210 200 190 180 170 160 150 140 130 120 110 100 90 80 70 60 50 40 30 20 10 0 -10
f1 (ppm)

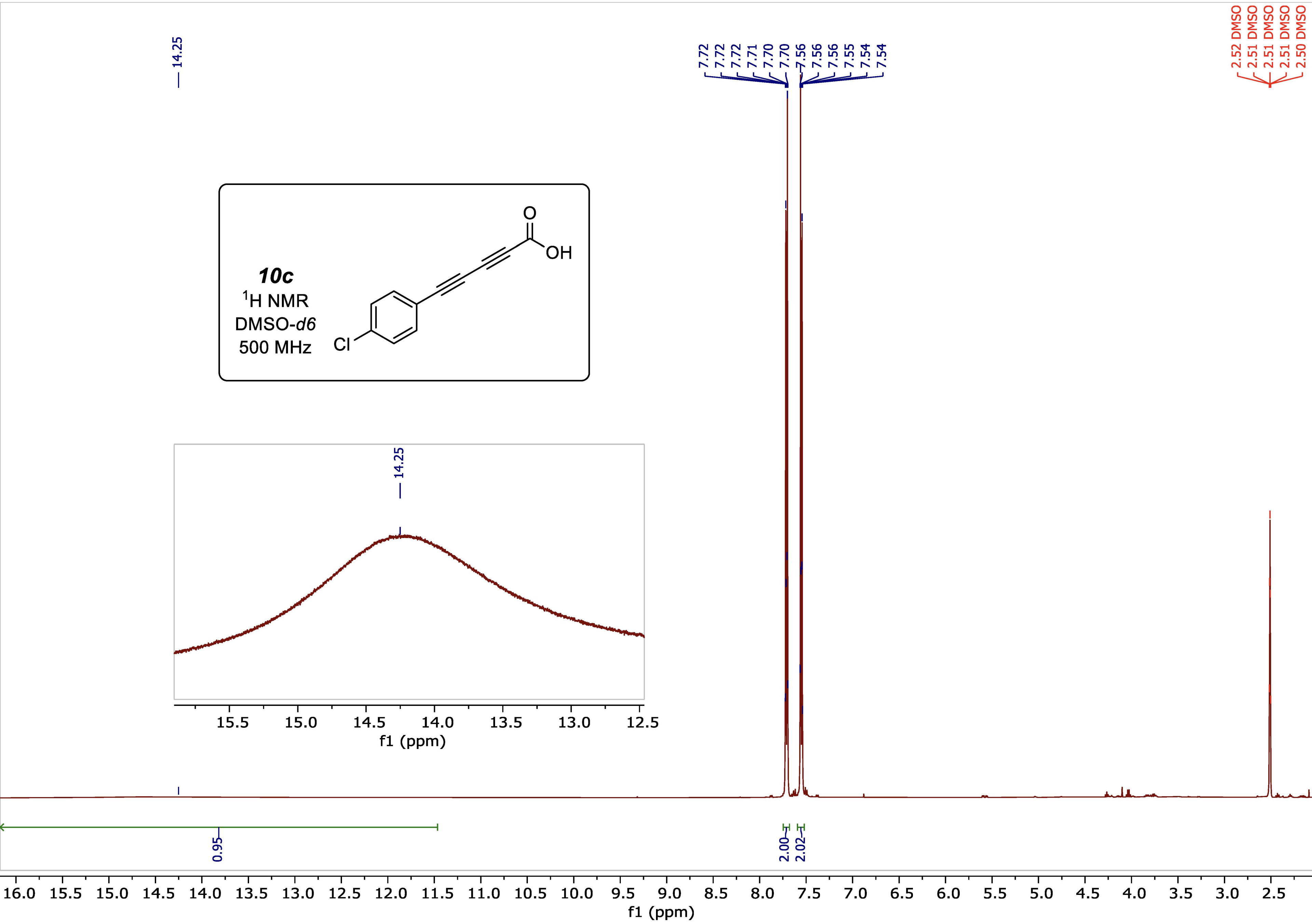

10c
^{1}H NMR
DMSO-d6
500 MHz
O
OH
Cl
14.25
7.72
7.72
7.72
7.71
7.70
7.70
7.56
7.56
7.56
7.55
7.54
7.54
2.52 DMSO
2.51 DMSO
2.51 DMSO
2.51 DMSO
2.50 DMSO
0.95
2.00
2.02
f1 (ppm)

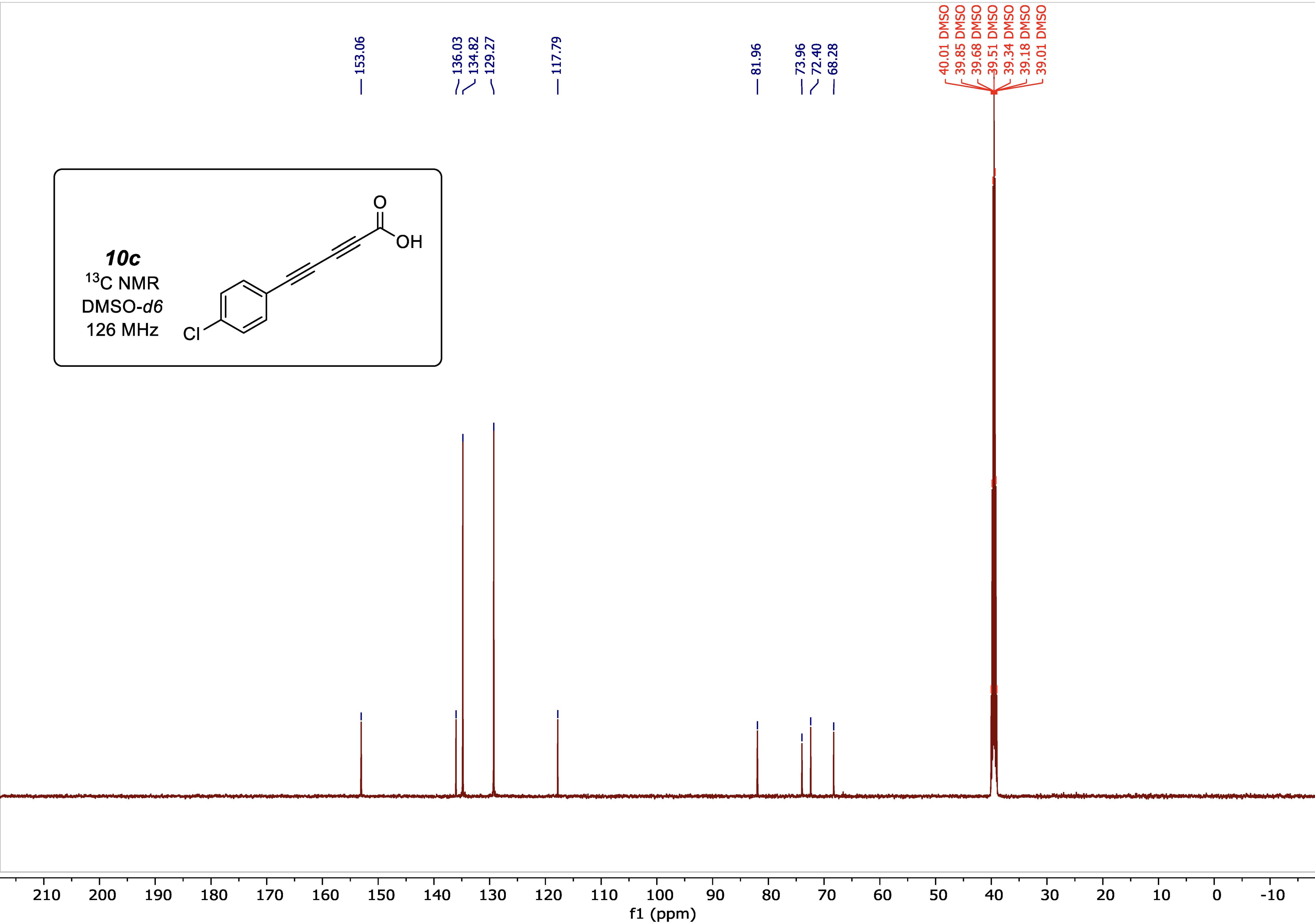
10c
^{13}C NMR
DMSO-d6
126 MHz
O
OH
Cl
153.06
136.03
134.82
129.27
117.79
81.96
73.96
72.40
68.28
40.01 DMSO
39.85 DMSO
39.68 DMSO
39.51 DMSO
39.34 DMSO
39.18 DMSO
39.01 DMSO
210 200 190 180 170 160 150 140 130 120 110 100 90 80 70 60 50 40 30 20 10 0 -10
f1 (ppm)

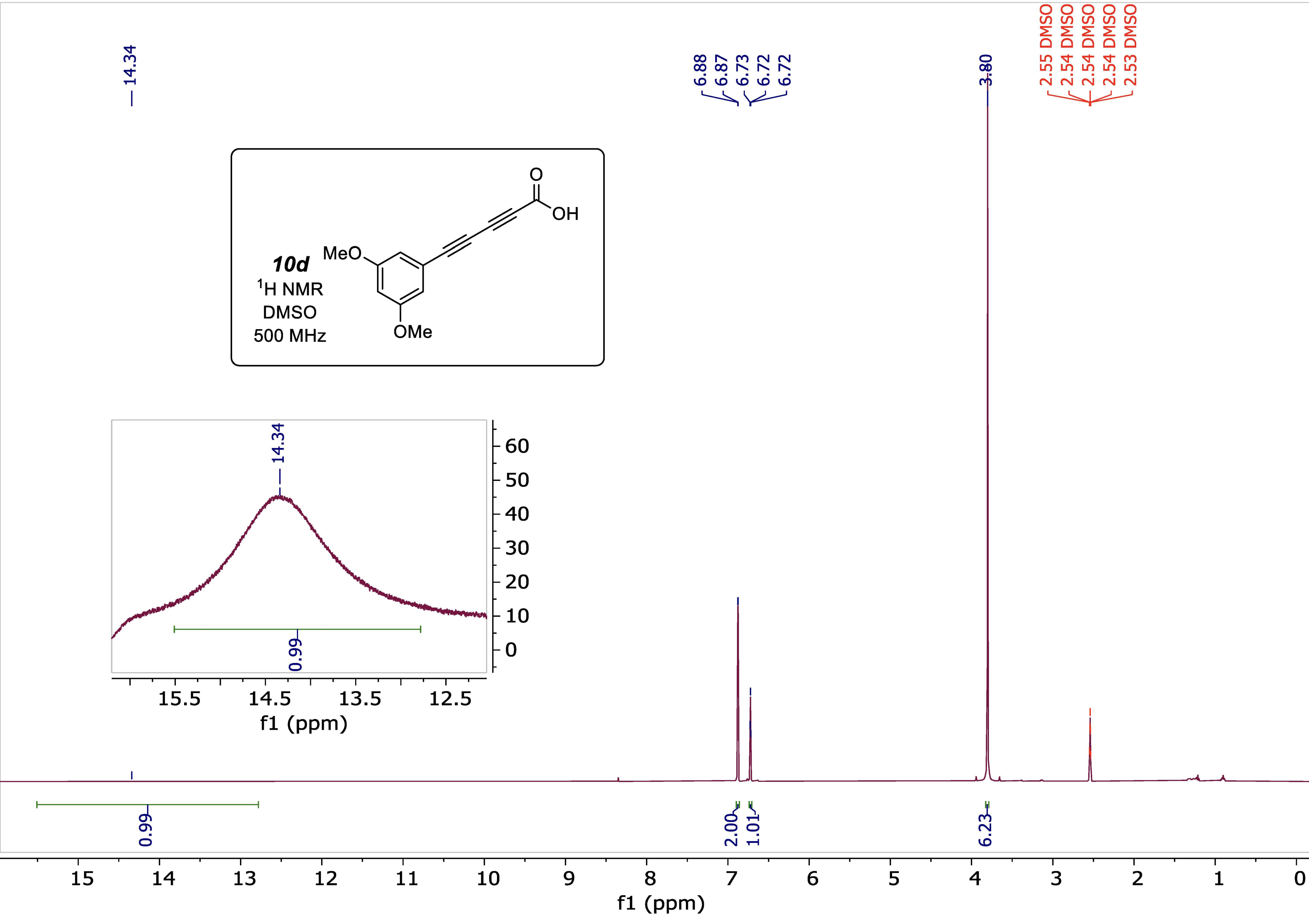
10d
^{1}H NMR
DMSO
500 MHz
MeO
OMe
O
OH
14.34
6.88
6.87
6.73
6.72
6.72
3.80
2.55 DMSO
2.54 DMSO
2.54 DMSO
2.54 DMSO
2.53 DMSO
0.99
2.00
1.01
6.23
f1 (ppm)

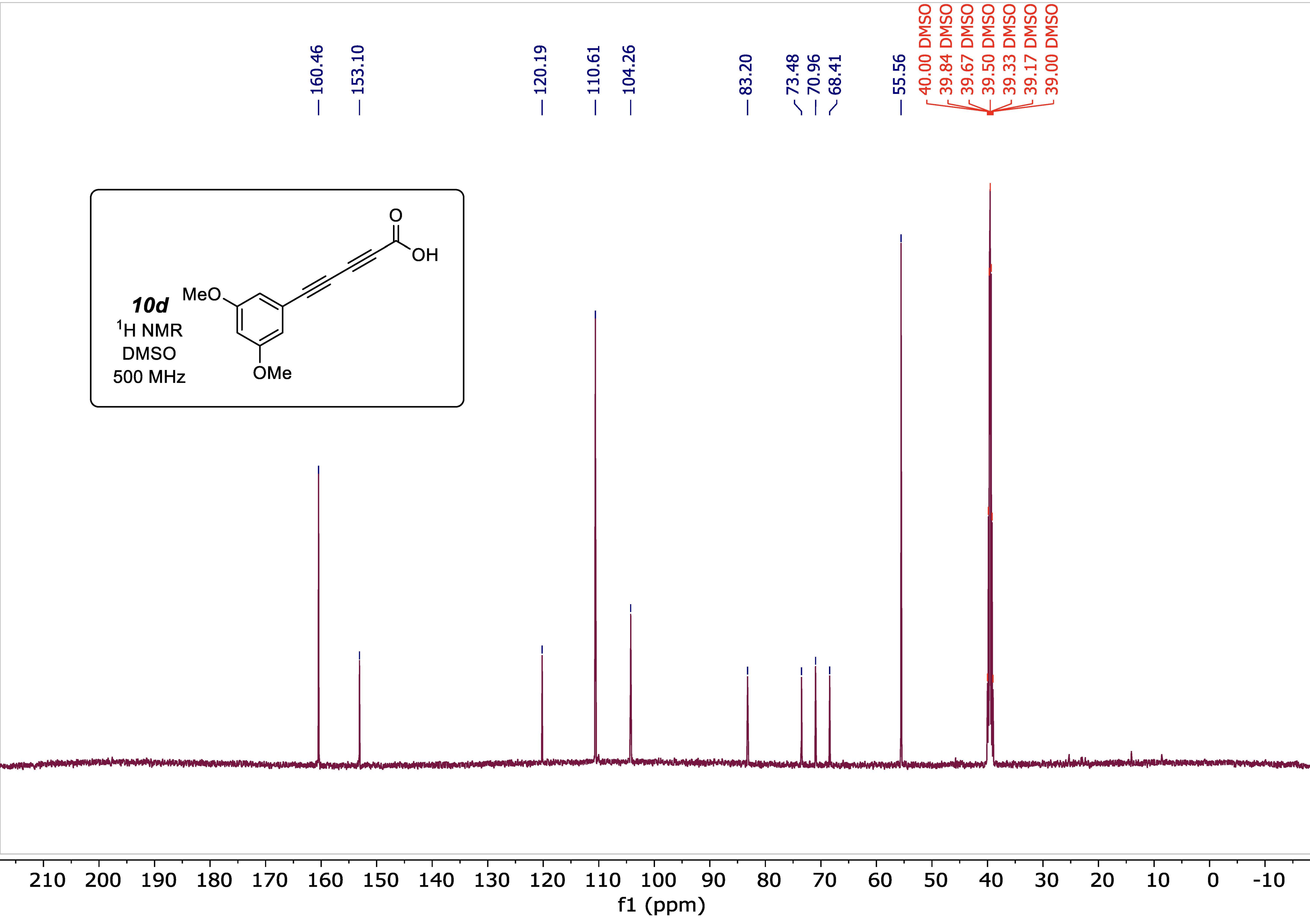
160.46
153.10
120.19
110.61
104.26
83.20
73.48
70.96
68.41
55.56
40.00 DMSO
39.84 DMSO
39.67 DMSO
39.50 DMSO
39.33 DMSO
39.17 DMSO
39.00 DMSO
10d
^{1}H NMR
DMSO
500 MHz
MeO
OMe
O
OH
210 200 190 180 170 160 150 140 130 120 110 100 90 80 70 60 50 40 30 20 10 0 -10
f1 (ppm)

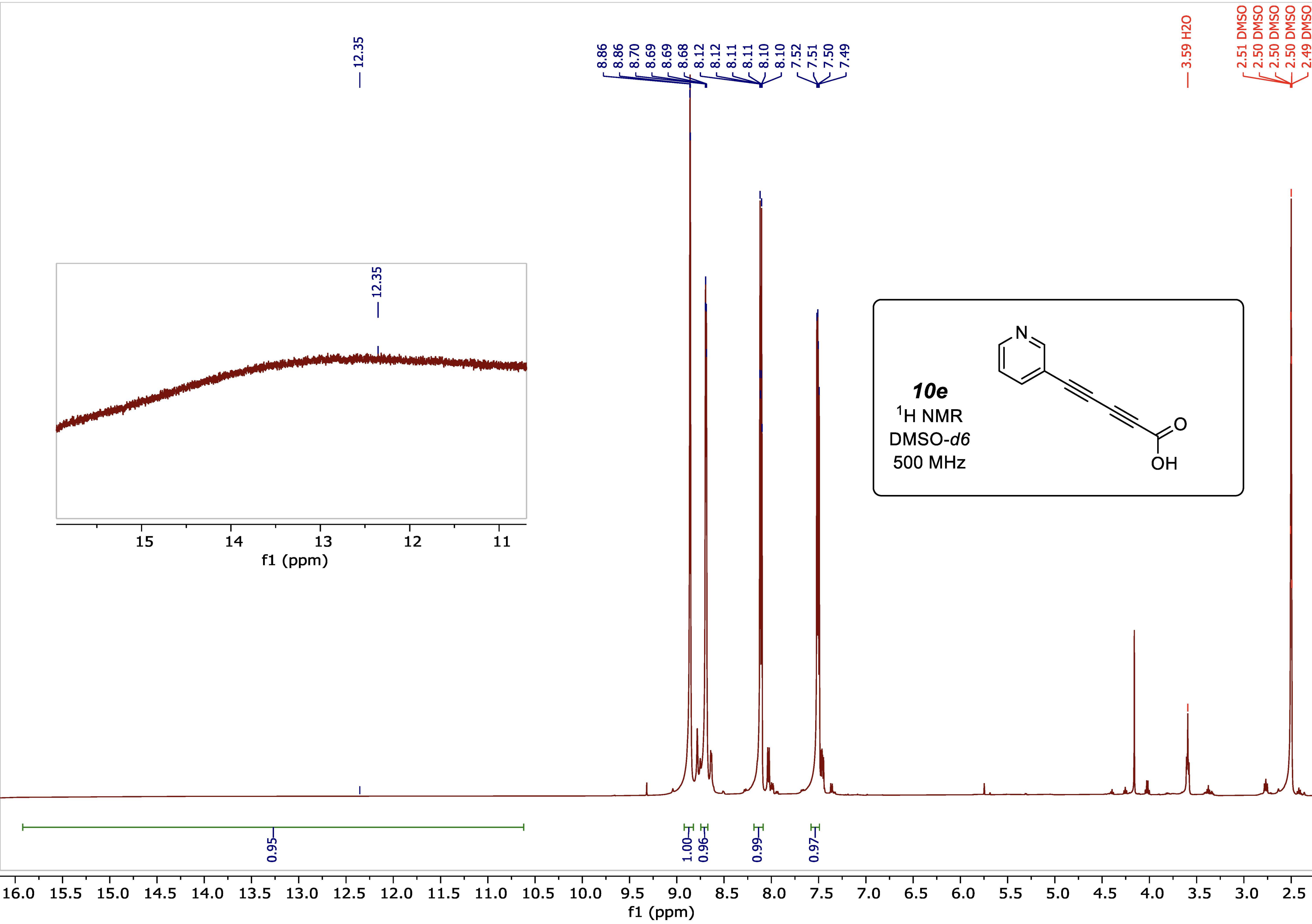
12.35
8.86
8.86
8.70
8.69
8.69
8.68
8.12
8.12
8.11
8.11
8.10
8.10
7.52
7.51
7.50
7.49
3.59 H2O
2.51 DMSO
2.50 DMSO
2.50 DMSO
2.50 DMSO
2.49 DMSO
15
14
13
12
11
f1 (ppm)
10e
1H NMR
DMSO-d6
500 MHz
N
O
OH
0.95
1.00
0.96
0.99
0.97
16.0 15.5 15.0 14.5 14.0 13.5 13.0 12.5 12.0 11.5 11.0 10.5 10.0 9.5 9.0 8.5 8.0 7.5 7.0 6.5 6.0 5.5 5.0 4.5 4.0 3.5 3.0 2.5
f1 (ppm)

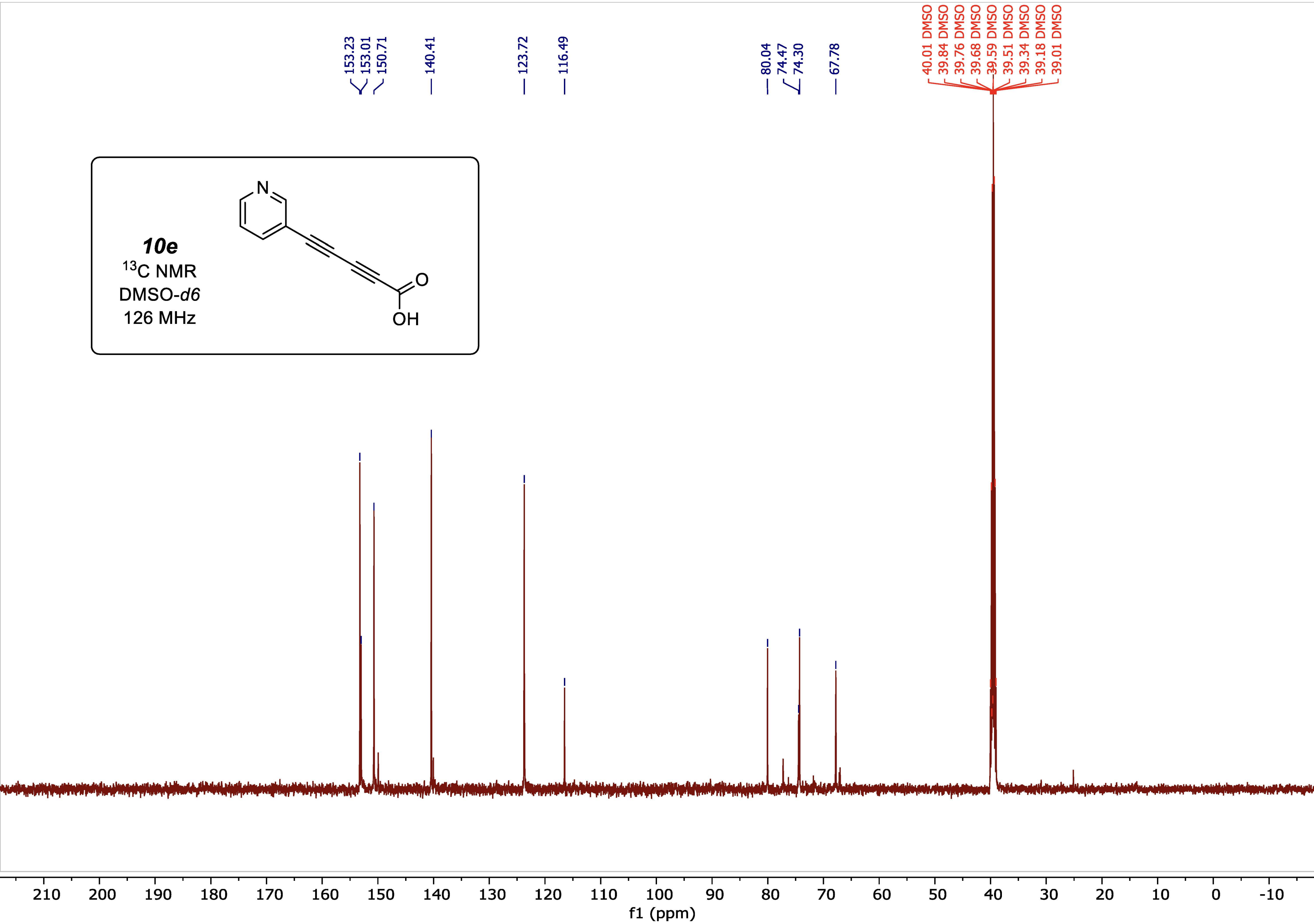
10e
^{13}C NMR
DMSO-d6
126 MHz
N
O
OH
153.23
153.01
150.71
140.41
123.72
116.49
80.04
74.47
74.30
67.78
40.01 DMSO
39.84 DMSO
39.76 DMSO
39.68 DMSO
39.59 DMSO
39.51 DMSO
39.34 DMSO
39.18 DMSO
39.01 DMSO
210 200 190 180 170 160 150 140 130 120 110 100 90 80 70 60 50 40 30 20 10 0 -10
f1 (ppm)

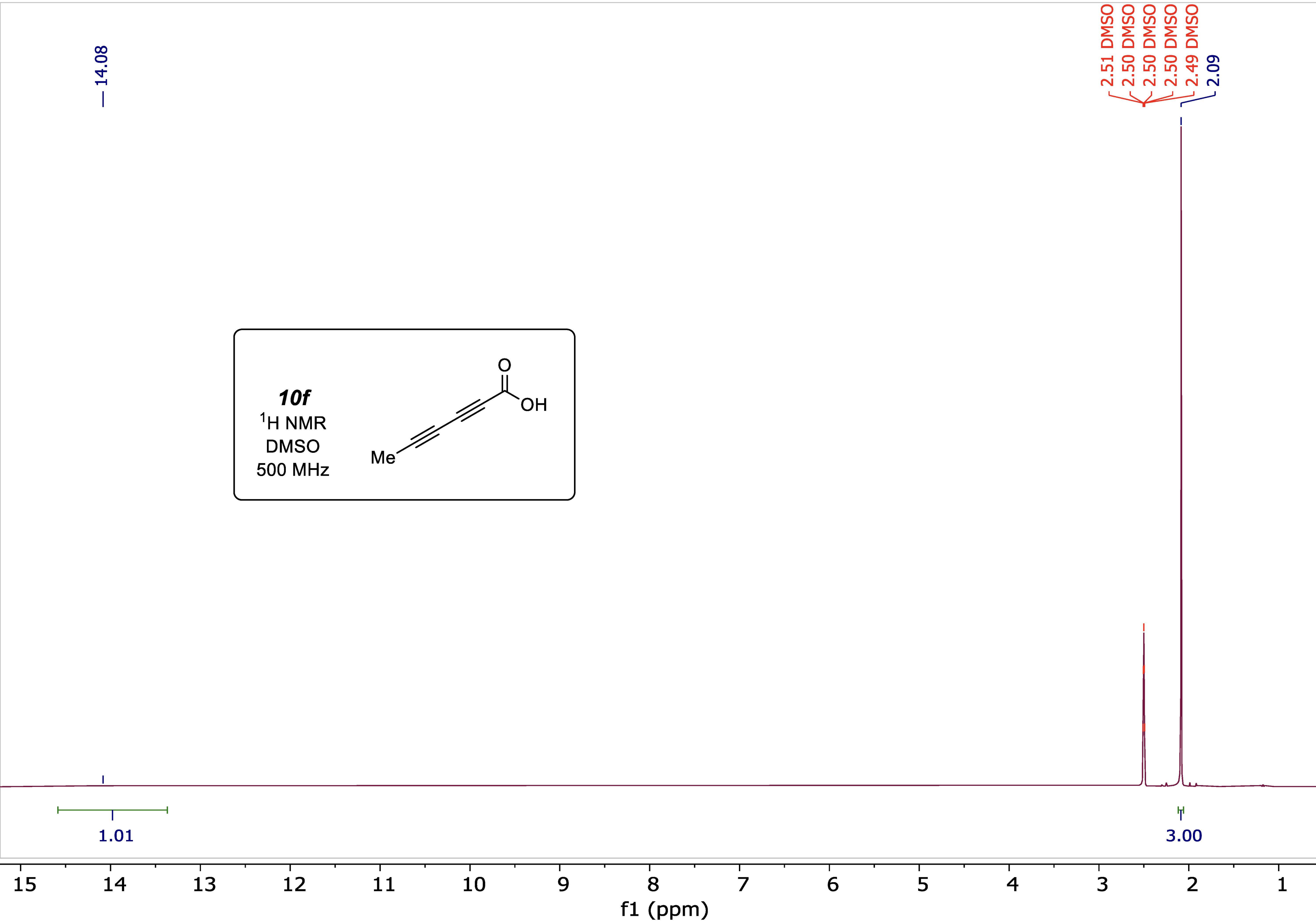
14.08
2.51 DMSO
2.50 DMSO
2.50 DMSO
2.50 DMSO
2.49 DMSO
2.09
10f
1H NMR
DMSO
500 MHz
O
OH
Me
1.01
3.00
15
14
13
12
11
10
9
8
7
6
5
4
3
2
1
f1 (ppm)

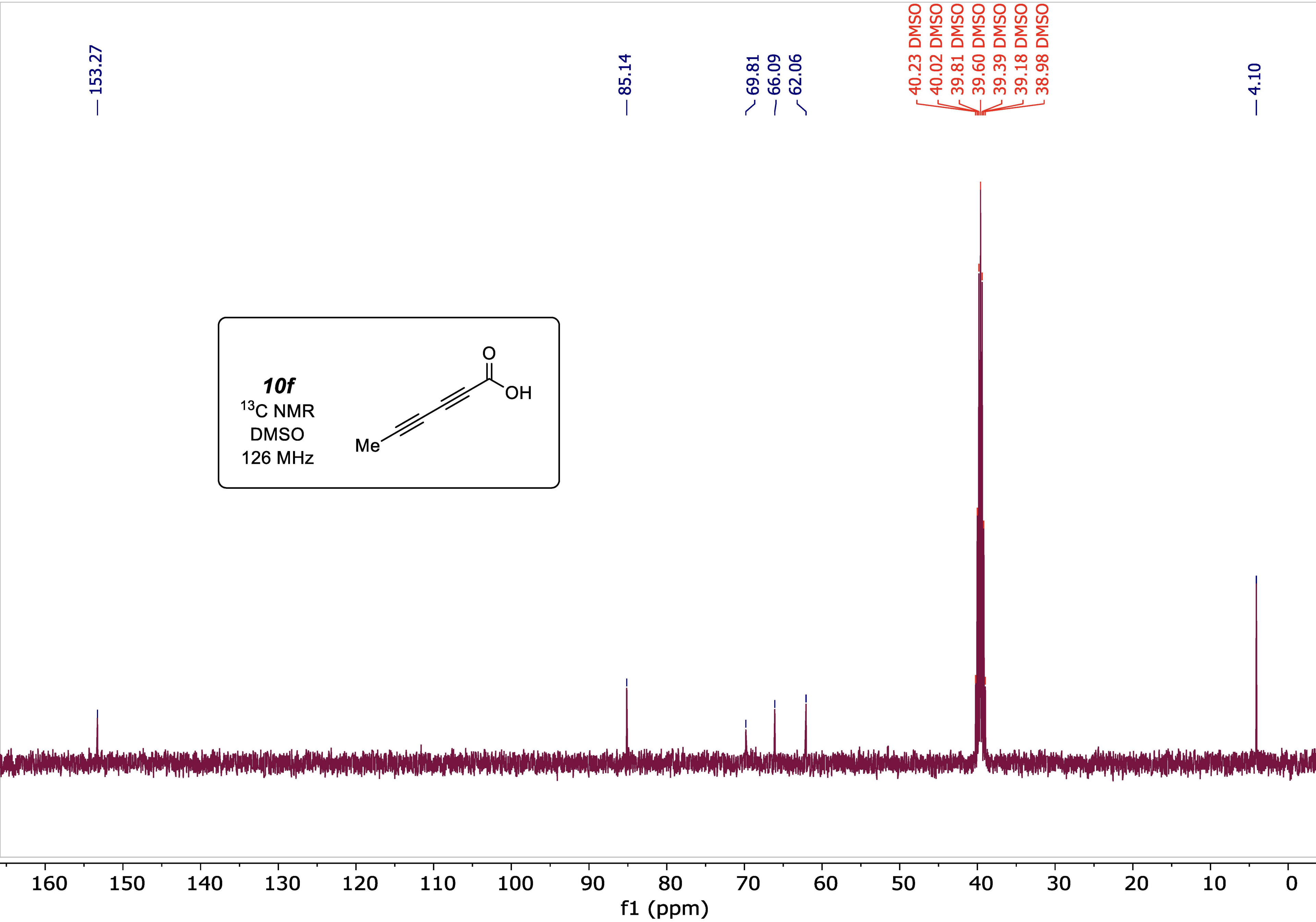
153.27
85.14
69.81
66.09
62.06
40.23 DMSO
40.02 DMSO
39.81 DMSO
39.60 DMSO
39.39 DMSO
39.18 DMSO
38.98 DMSO
4.10
10f
13C NMR
DMSO
126 MHz
O
OH
Me
160
150
140
130
120
110
100
90
80
70
60
50
40
30
20
10
0
f1 (ppm)

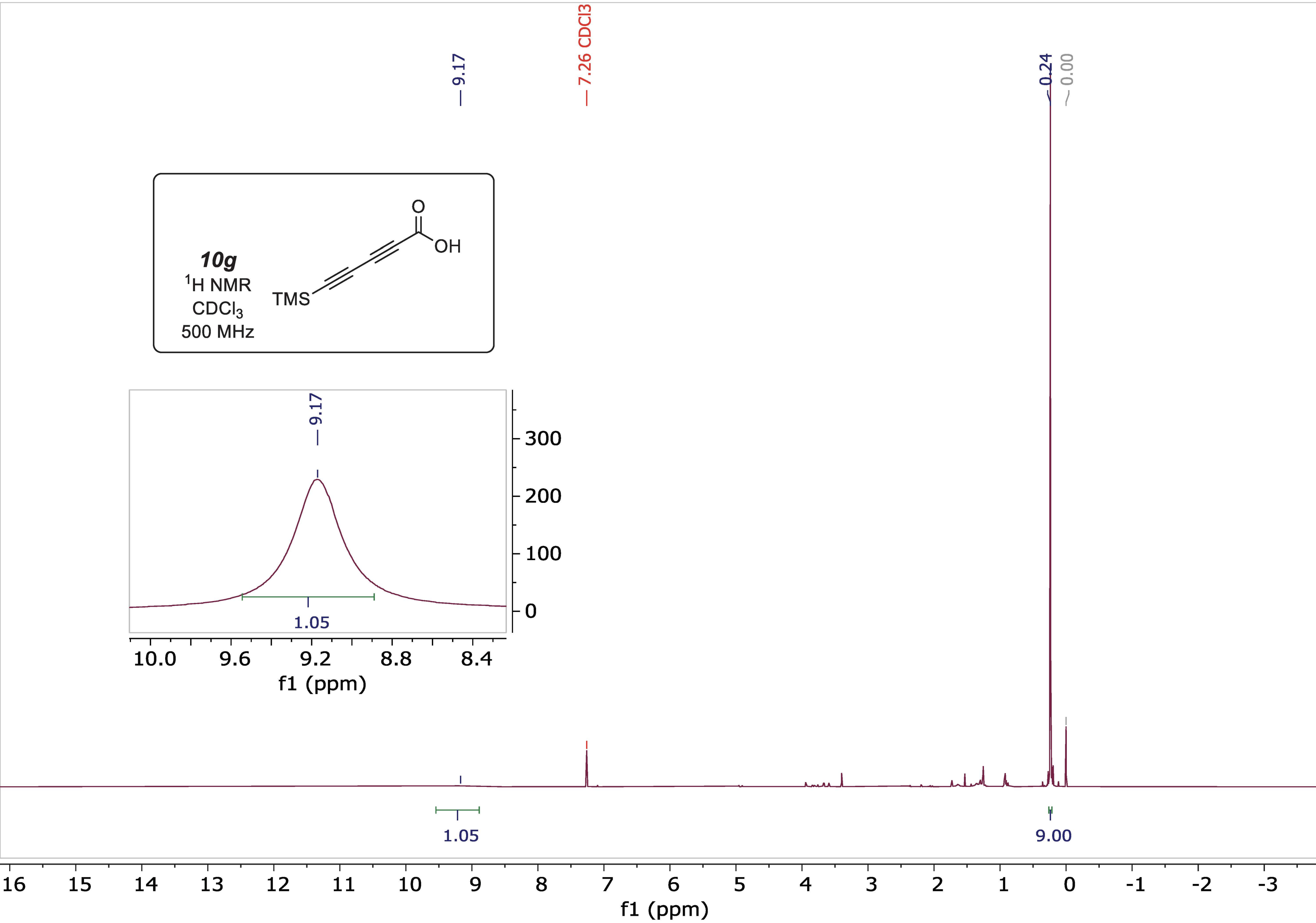
9.17
7.26 CDCl3
0.24
0.00
10g
^{1}H NMR
$CDCl_3$
500 MHz
O
OH
TMS
9.17
300
200
100
0
1.05
10.0
9.6
9.2
8.8
8.4
f1 (ppm)
1.05
9.00
16
15
14
13
12
11
10
9
8
7
6
5
4
3
2
1
0
-1
-2
-3
f1 (ppm)

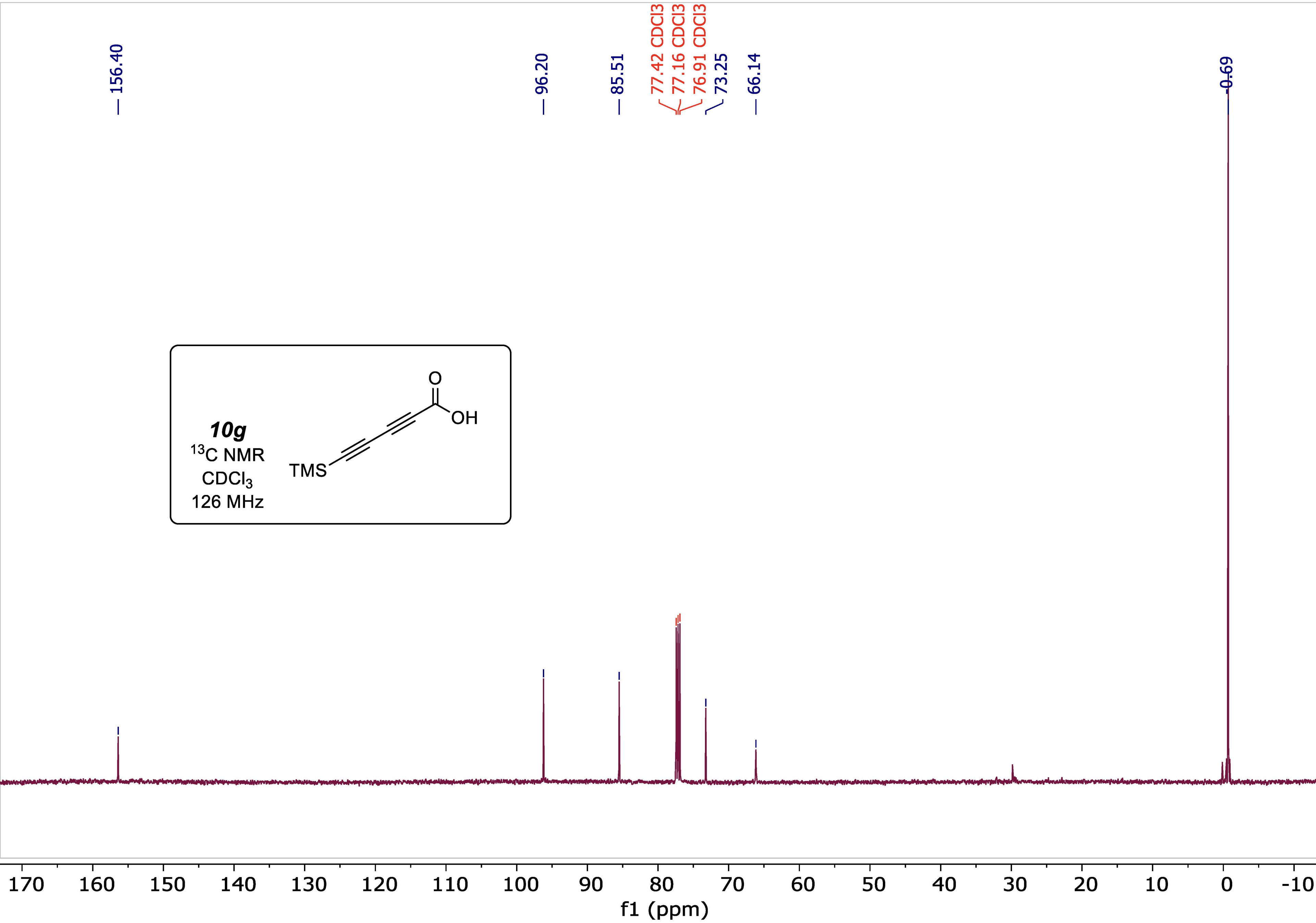
156.40
96.20
85.51
77.42 CDCl3
77.16 CDCl3
76.91 CDCl3
73.25
66.14
-0.69
10g
13C NMR
CDCl3
126 MHz
TMS
O
OH
170 160 150 140 130 120 110 100 90 80 70 60 50 40 30 20 10 0 -10
f1 (ppm)

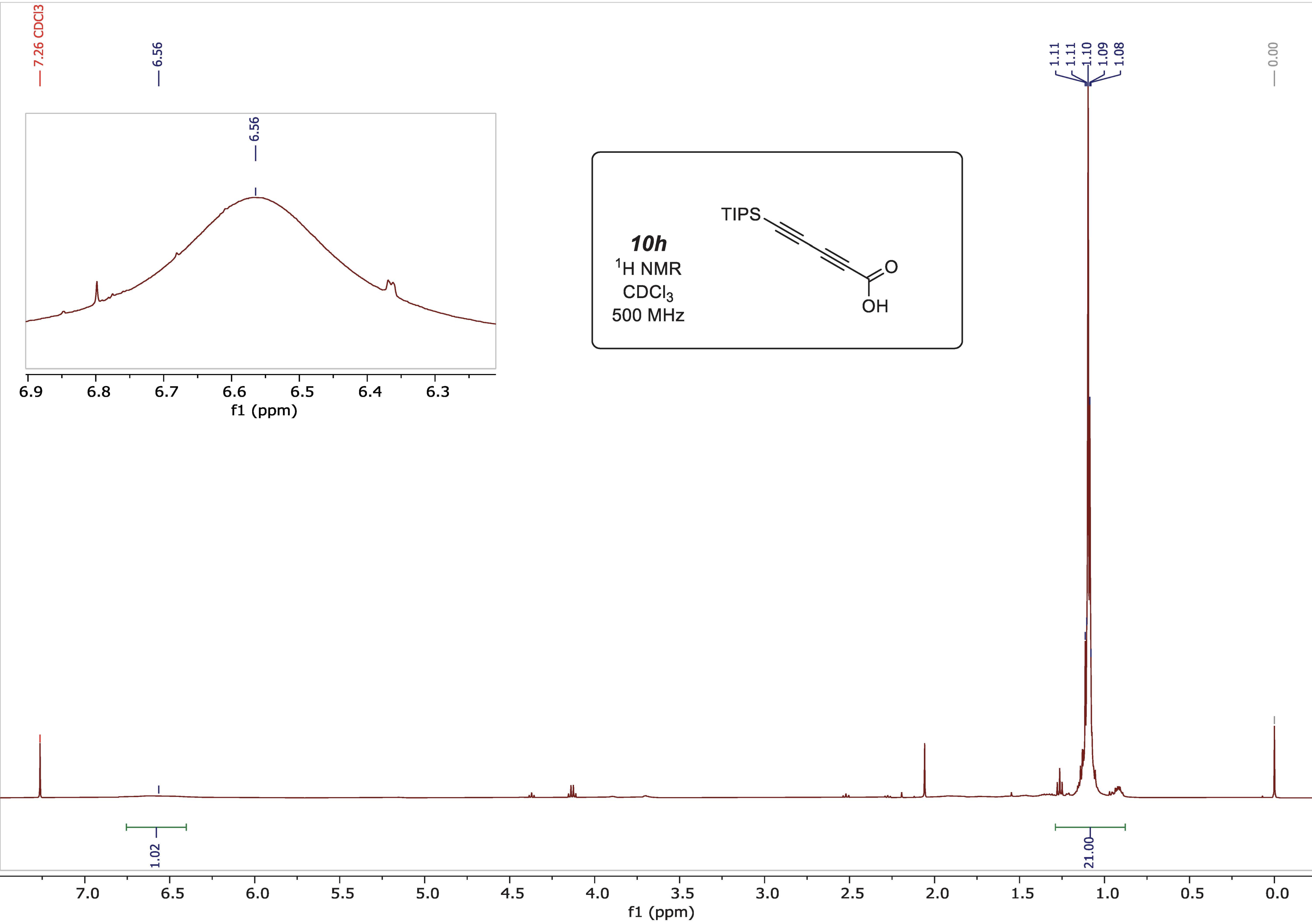

7.26 CDCl3
6.56
1.11
1.11
1.10
1.09
1.08
0.00
6.56
6.9 6.8 6.7 6.6 6.5 6.4 6.3
f1 (ppm)
10h
1H NMR
CDCl3
500 MHz
TIPS
O
OH
1.02
21.00
7.0 6.5 6.0 5.5 5.0 4.5 4.0 3.5 3.0 2.5 2.0 1.5 1.0 0.5 0.0
f1 (ppm)

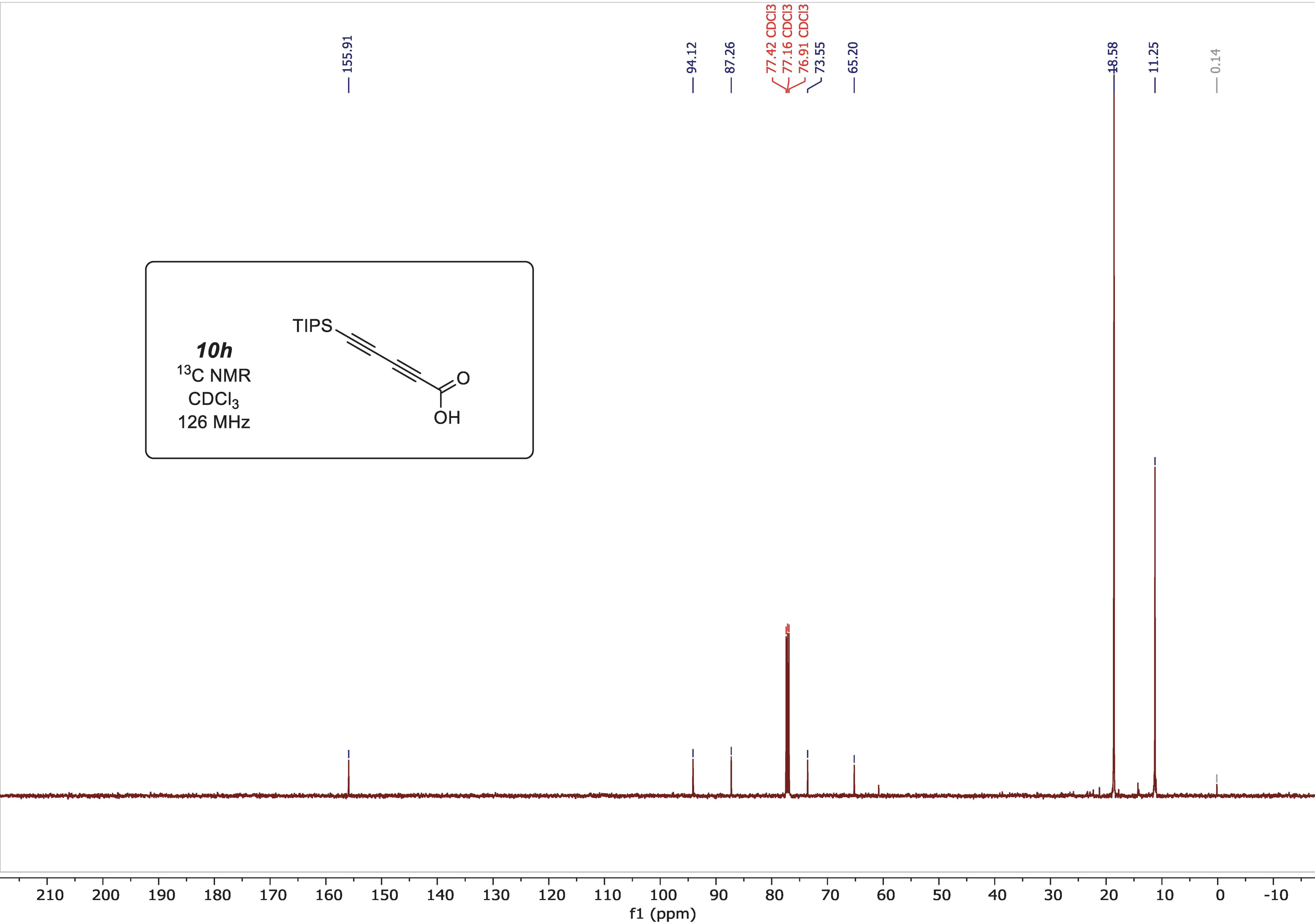

155.91
94.12
87.26
77.42 CDCl3
77.16 CDCl3
76.91 CDCl3
73.55
65.20
18.58
11.25
0.14
10h
13C NMR
CDCl3
126 MHz
TIPS
O
OH
210 200 190 180 170 160 150 140 130 120 110 100 90 80 70 60 50 40 30 20 10 0 -10
f1 (ppm)

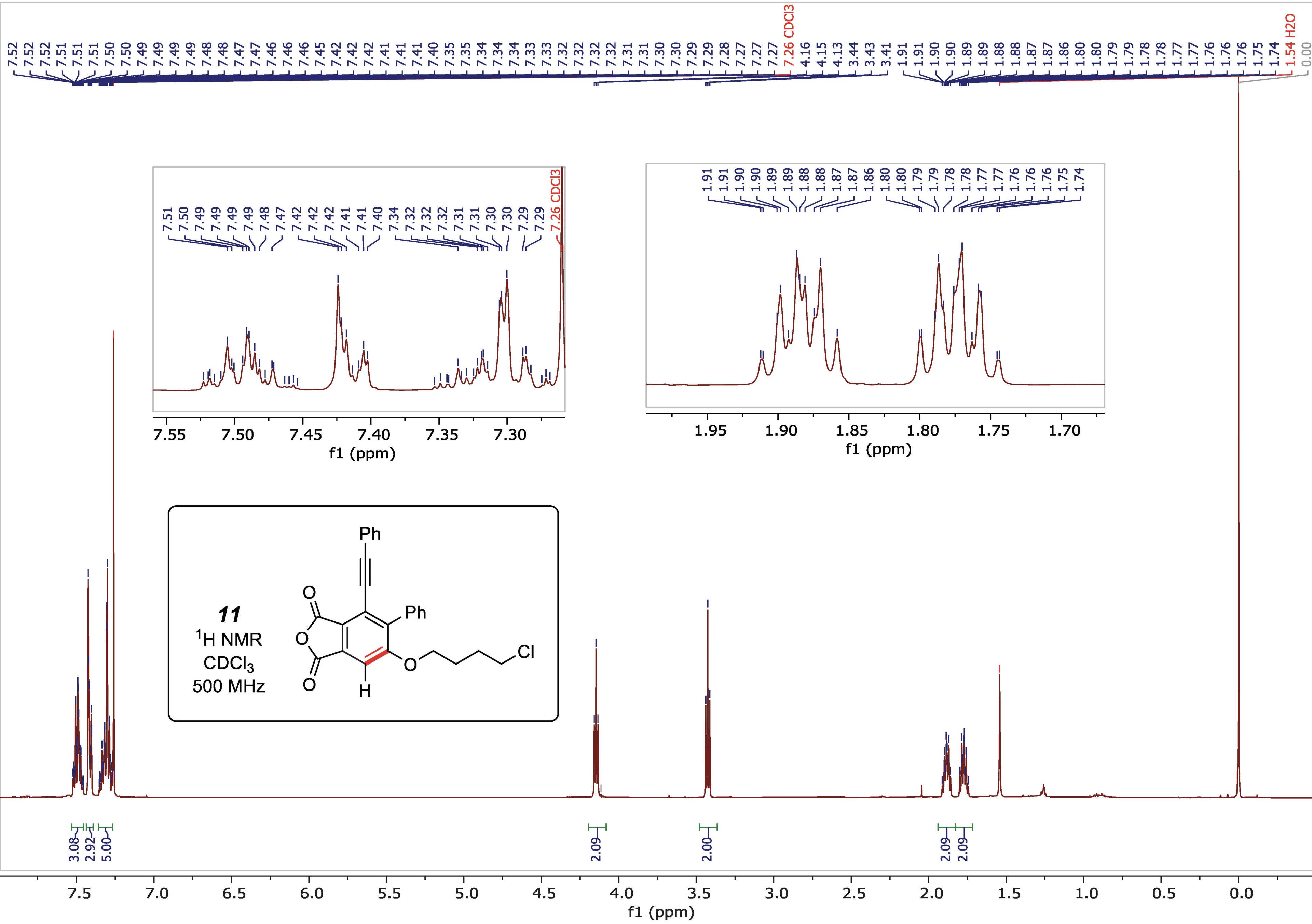
11
^{1}H NMR
$CDCl_3$
500 MHz
Ph
Ph
O
O
O
O
Cl
H
7.26 CDCl3
1.54 H2O
0.00
3.08
2.92
5.00
2.09
2.00
2.09
2.09
f1 (ppm)

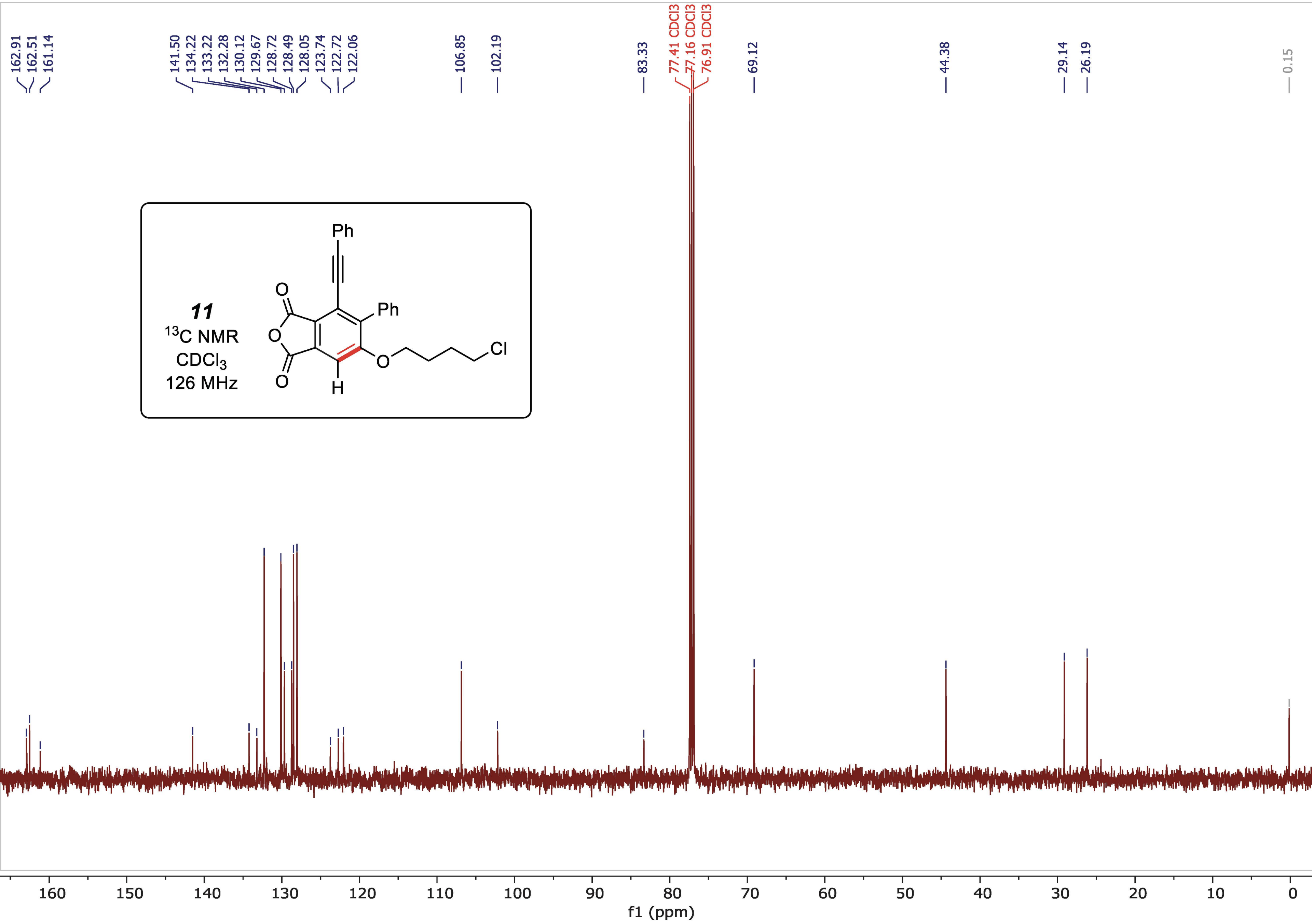
162.91
162.51
161.14
141.50
134.22
133.22
132.28
130.12
129.67
128.72
128.49
128.05
123.74
122.72
122.06
106.85
102.19
83.33
77.41 CDCl3
77.16 CDCl3
76.91 CDCl3
69.12
44.38
29.14
26.19
0.15
11
^{13}C NMR
CDCl$_3$
126 MHz
Ph
O
O
O
Ph
O
Cl
H
f1 (ppm)

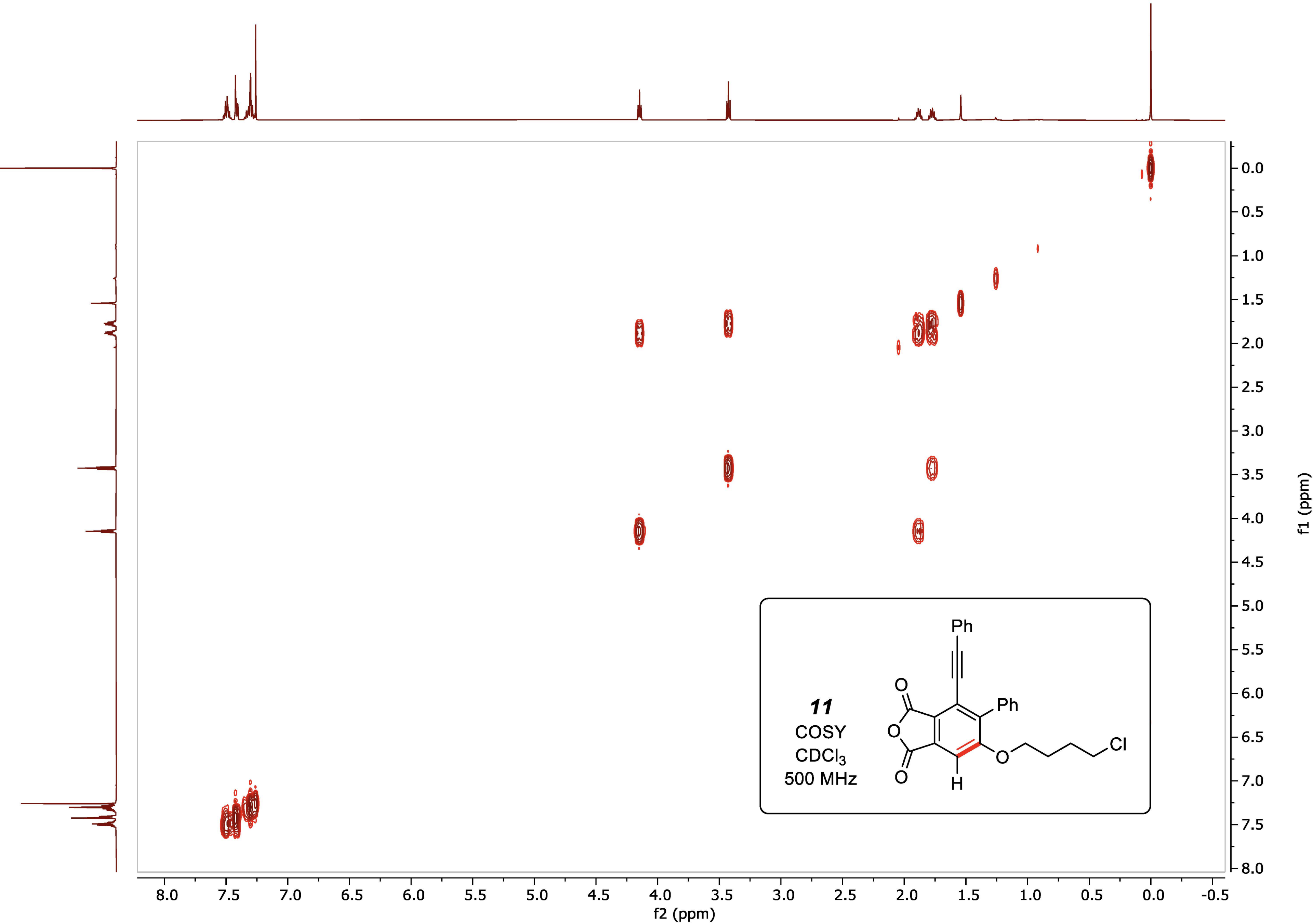
11
COSY
$CDCl_3$
500 MHz
Ph
Ph
O
O
O
O
H
Cl
f2 (ppm)
f1 (ppm)

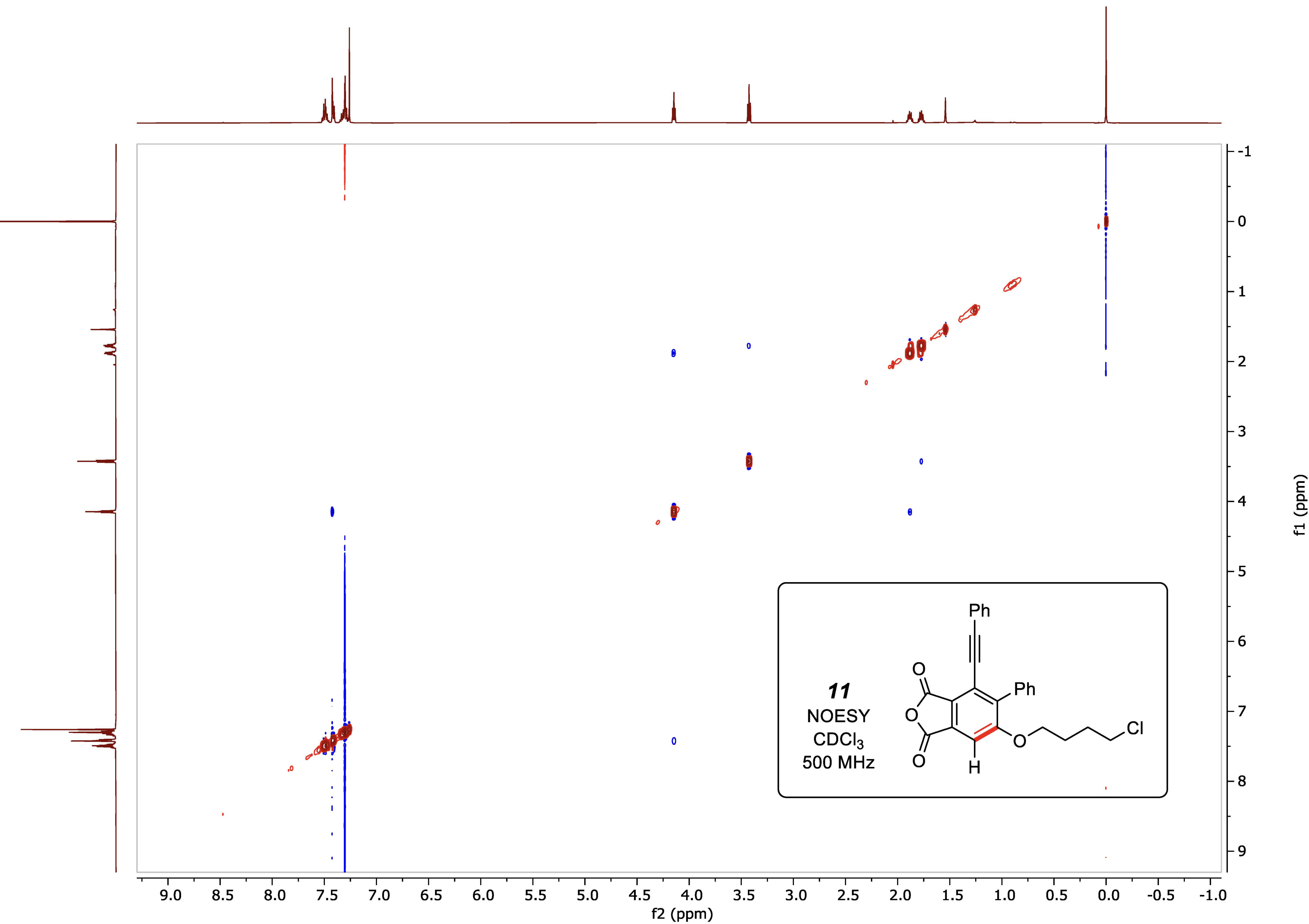
11
NOESY
$CDCl_3$
500 MHz
Ph
Ph
O
O
O
O
Cl
H
f2 (ppm)
f1 (ppm)

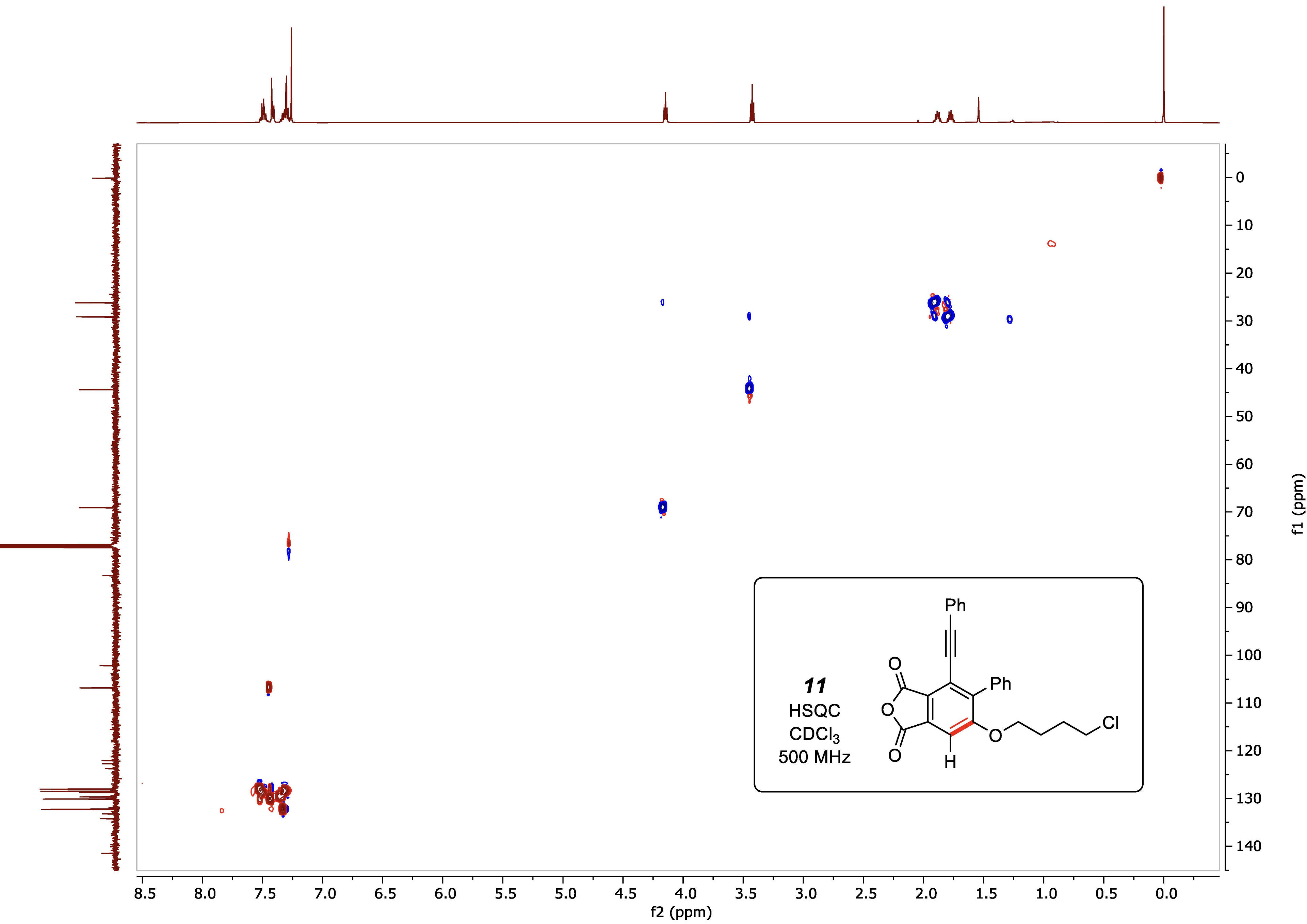
11
HSQC
$CDCl_3$
500 MHz
Ph
Ph
O
O
O
O
Cl
H
f2 (ppm)
f1 (ppm)

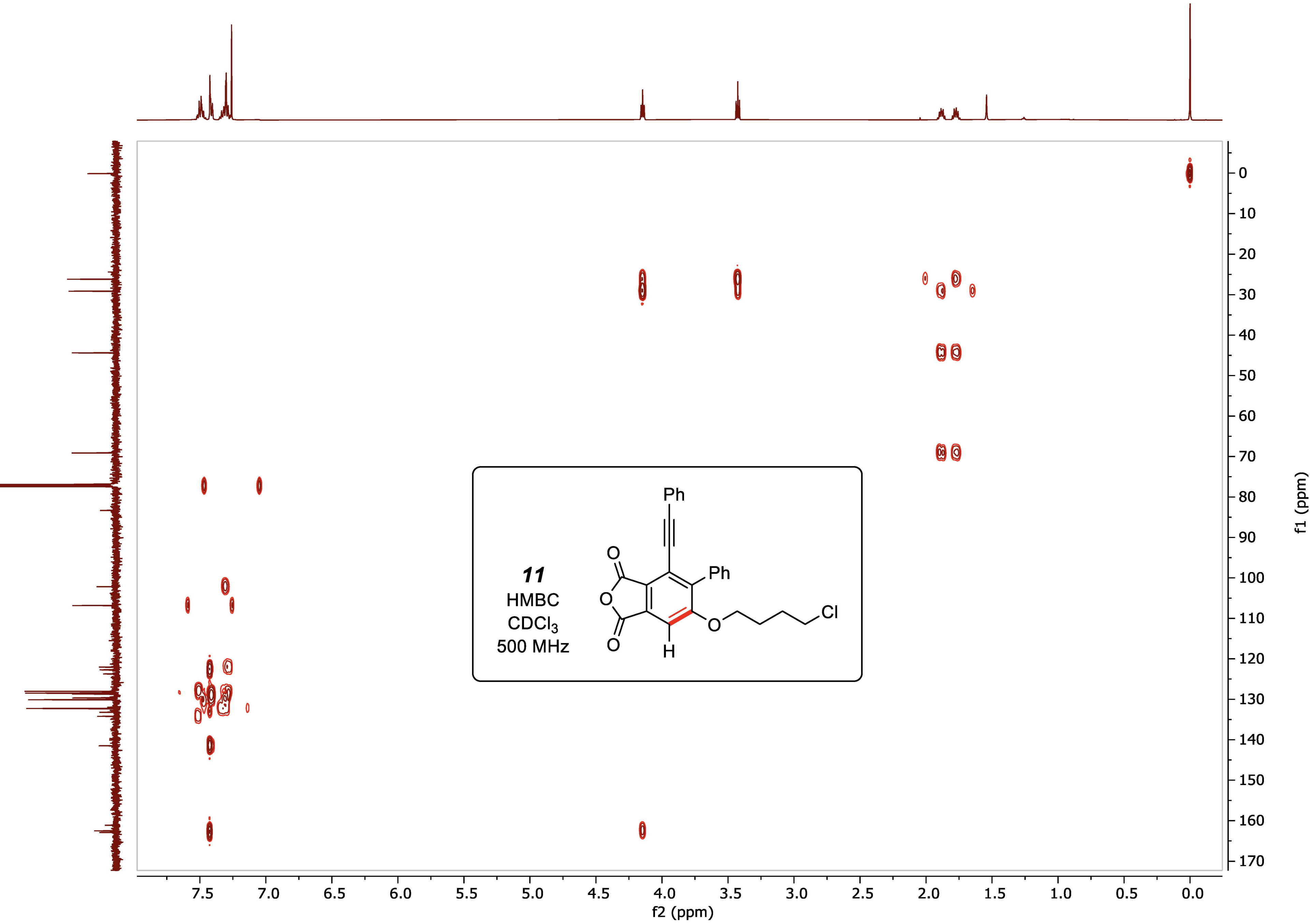

11
HMBC
CDCl3
500 MHz
Ph
O
O
O
Ph
O
Cl
H
f2 (ppm)
f1 (ppm)

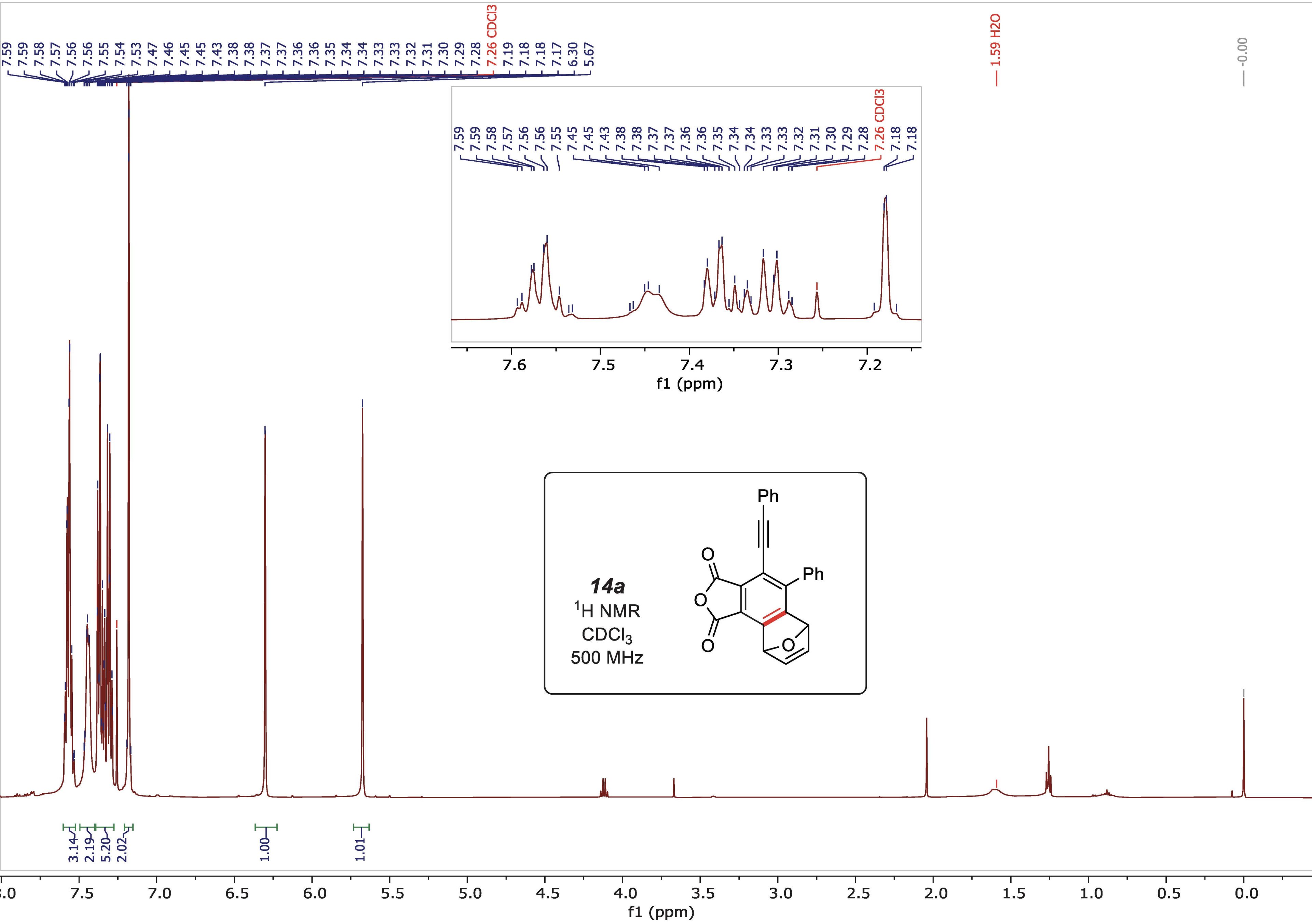
7.59
7.59
7.58
7.57
7.56
7.56
7.55
7.54
7.53
7.47
7.46
7.45
7.45
7.43
7.38
7.38
7.37
7.37
7.36
7.36
7.35
7.34
7.34
7.33
7.33
7.32
7.31
7.30
7.29
7.28
7.26 CDCl3
7.19
7.18
7.18
7.17
6.30
5.67
1.59 H2O
-0.00
7.59
7.59
7.58
7.57
7.56
7.56
7.55
7.45
7.45
7.43
7.38
7.38
7.37
7.37
7.36
7.36
7.35
7.34
7.34
7.33
7.33
7.32
7.31
7.30
7.29
7.28
7.26 CDCl3
7.18
7.18
7.6
7.5
7.4
7.3
7.2
f1 (ppm)
14a
1H NMR
CDCl3
500 MHz
Ph
O
O
O
Ph
O
3.14
2.19
5.20
2.02
1.00
1.01
7.5
7.0
6.5
6.0
5.5
5.0
4.5
4.0
3.5
3.0
2.5
2.0
1.5
1.0
0.5
0.0
f1 (ppm)

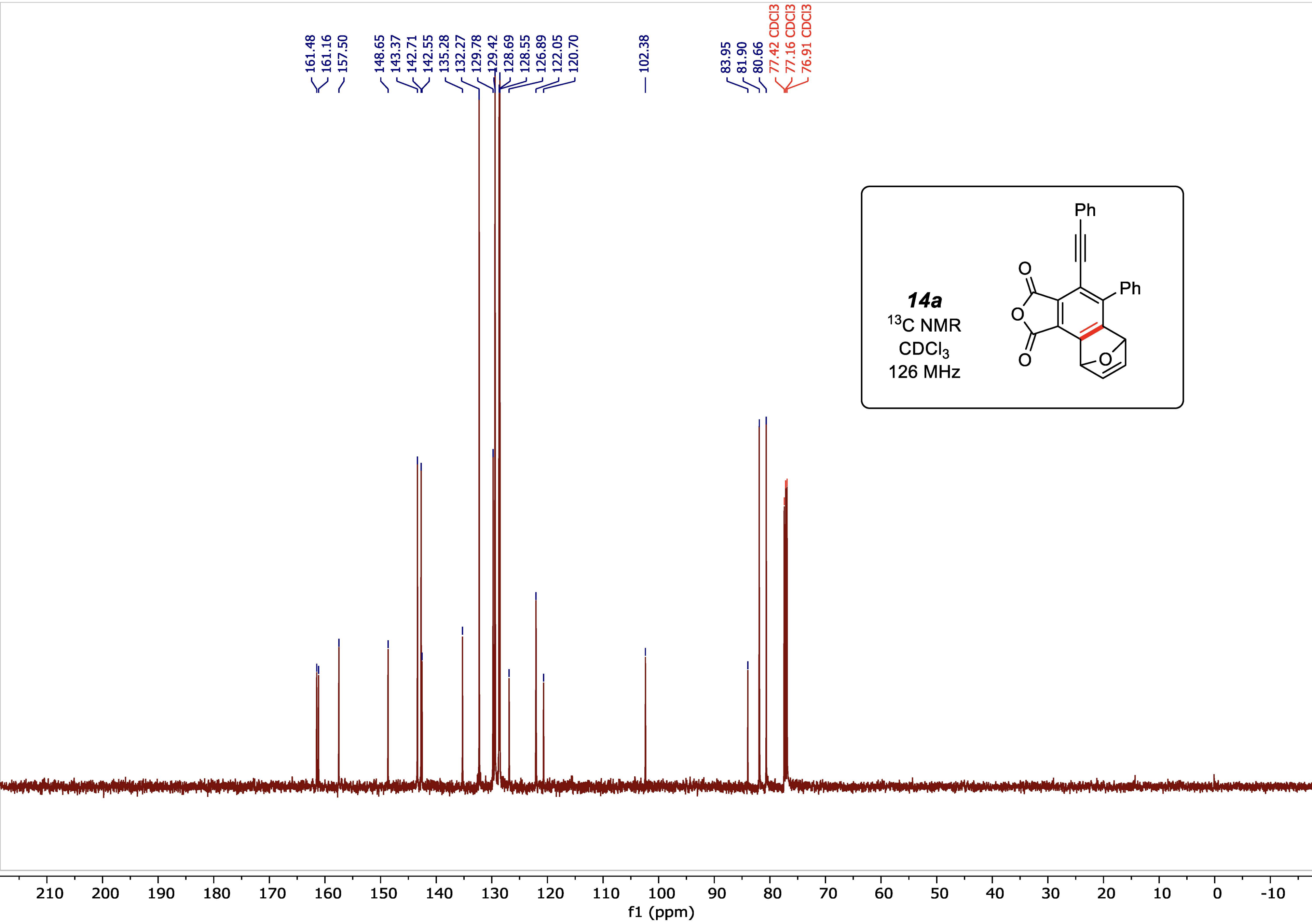

14a

^{13}C NMR

$CDCl_3$

126 MHz

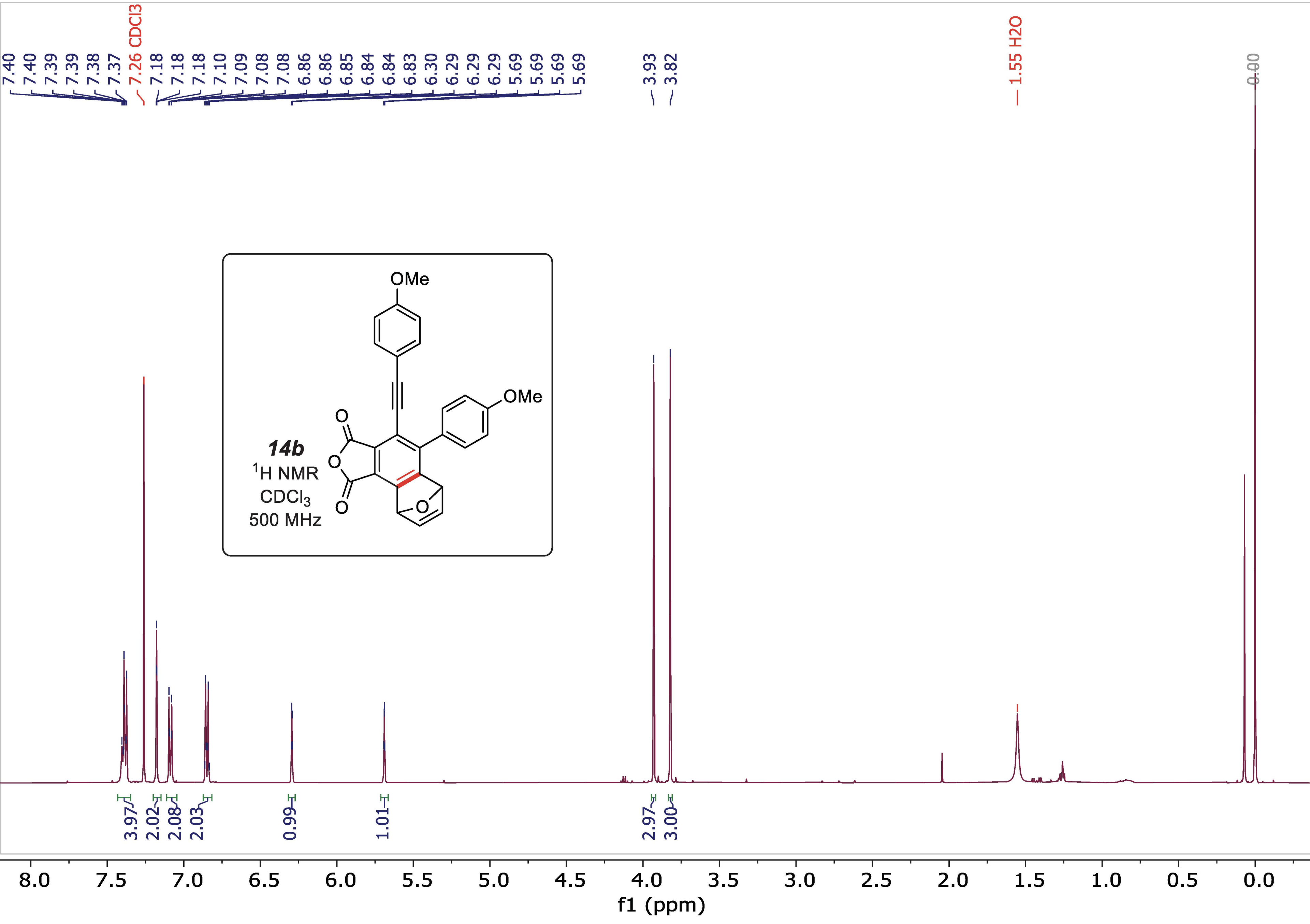
7.40 7.40 7.39 7.39 7.38 7.37 7.26 CDCl3 7.18 7.18 7.18 7.10 7.09 7.08 7.08 6.86 6.86 6.85 6.84 6.84 6.83 6.30 6.29 6.29 6.29 5.69 5.69 5.69 5.69
3.93 3.82
1.55 H2O
0.00
OMe
OMe
O
O
O
O
14b
¹H NMR
CDCl₃
500 MHz
3.97 2.02 2.08 2.03 0.99 1.01 2.97 3.00
8.0 7.5 7.0 6.5 6.0 5.5 5.0 4.5 4.0 3.5 3.0 2.5 2.0 1.5 1.0 0.5 0.0
f1 (ppm)

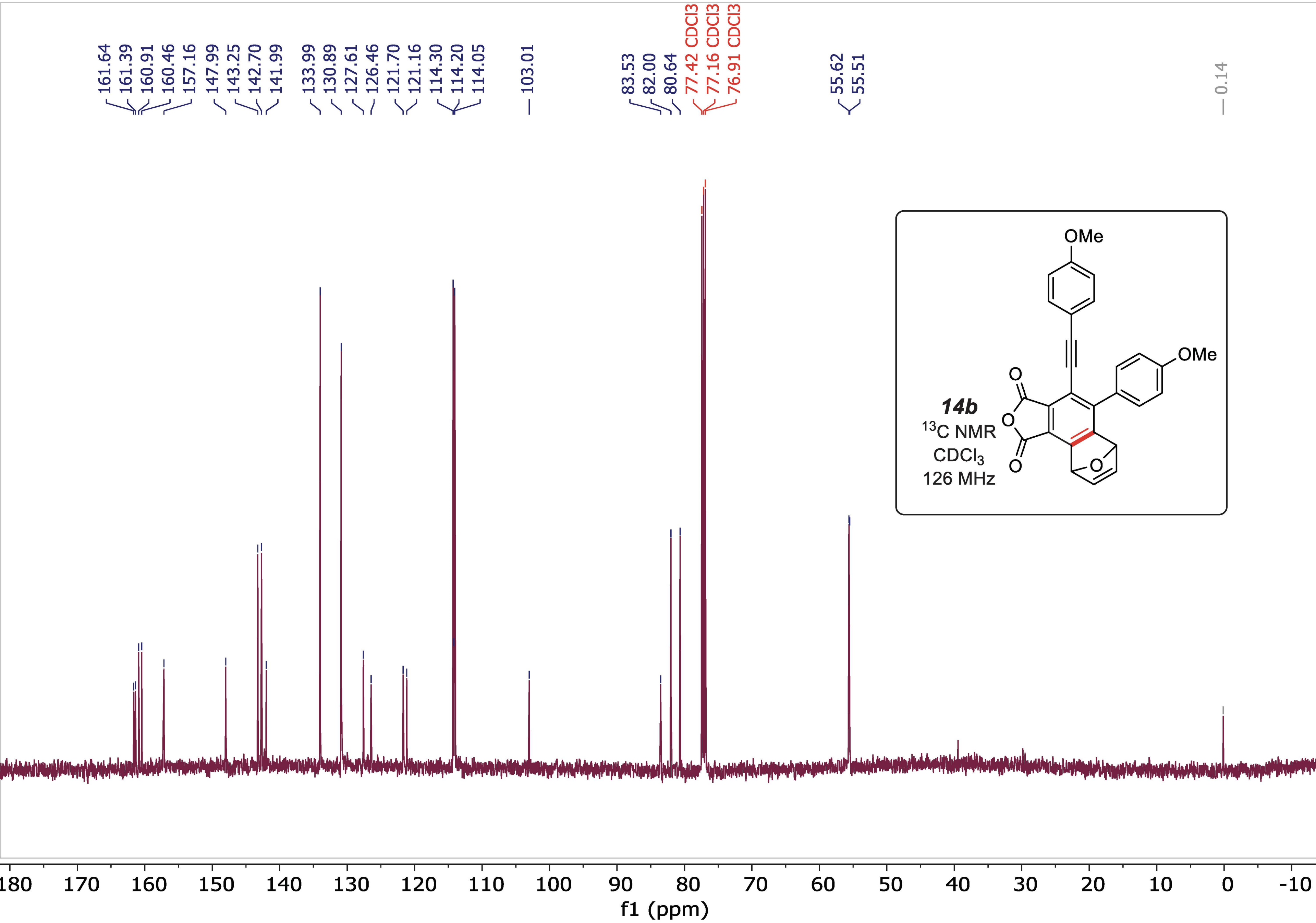
161.64
161.39
160.91
160.46
157.16
147.99
143.25
142.70
141.99
133.99
130.89
127.61
126.46
121.70
121.16
114.30
114.20
114.05
103.01
83.53
82.00
80.64
77.42 CDCl3
77.16 CDCl3
76.91 CDCl3
55.62
55.51
0.14
OMe
OMe
O
O
O
O
14b
13C NMR
CDCl3
126 MHz
180 170 160 150 140 130 120 110 100 90 80 70 60 50 40 30 20 10 0 -10
f1 (ppm)

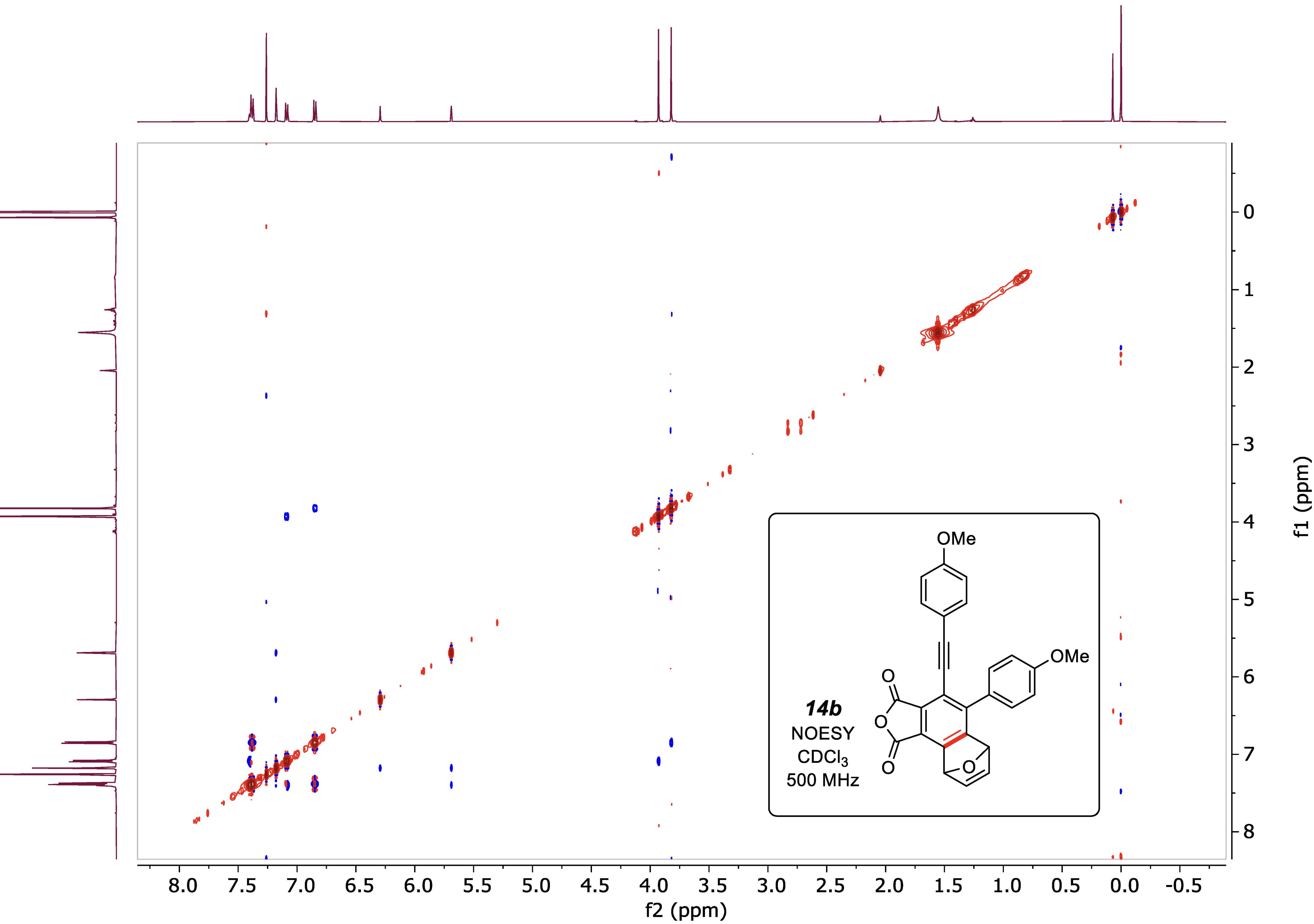

8.0
7.5
7.0
6.5
6.0
5.5
5.0
4.5
4.0
3.5
3.0
2.5
2.0
1.5
1.0
0.5
0.0
-0.5
f2 (ppm)
0
1
2
3
4
5
6
7
8
f1 (ppm)
OMe
OMe
O
O
O
14b
NOESY
CDCl3
500 MHz

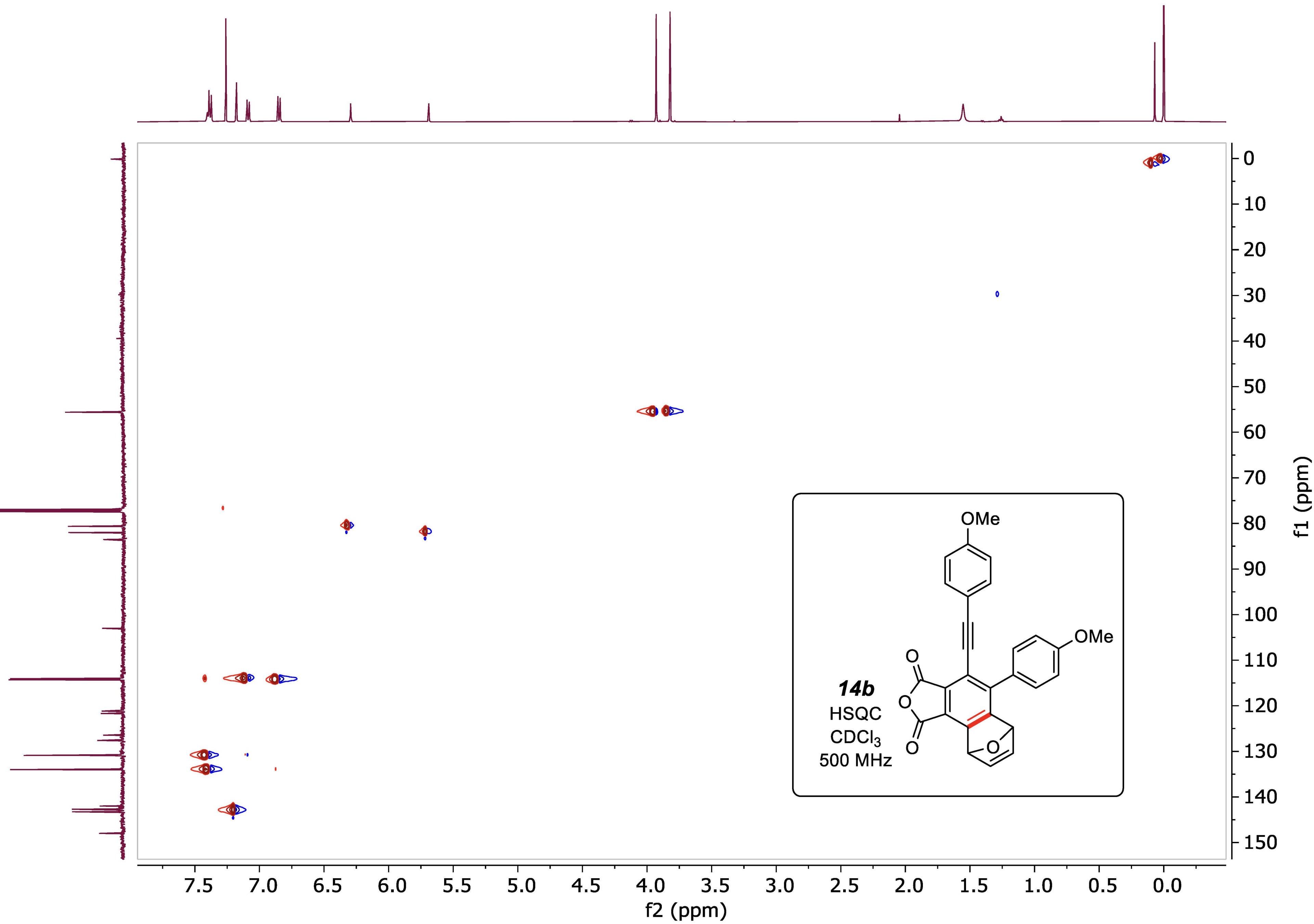
14b
HSQC
CDCl3
500 MHz
OMe
OMe
O
O
O
O
f2 (ppm)
f1 (ppm)

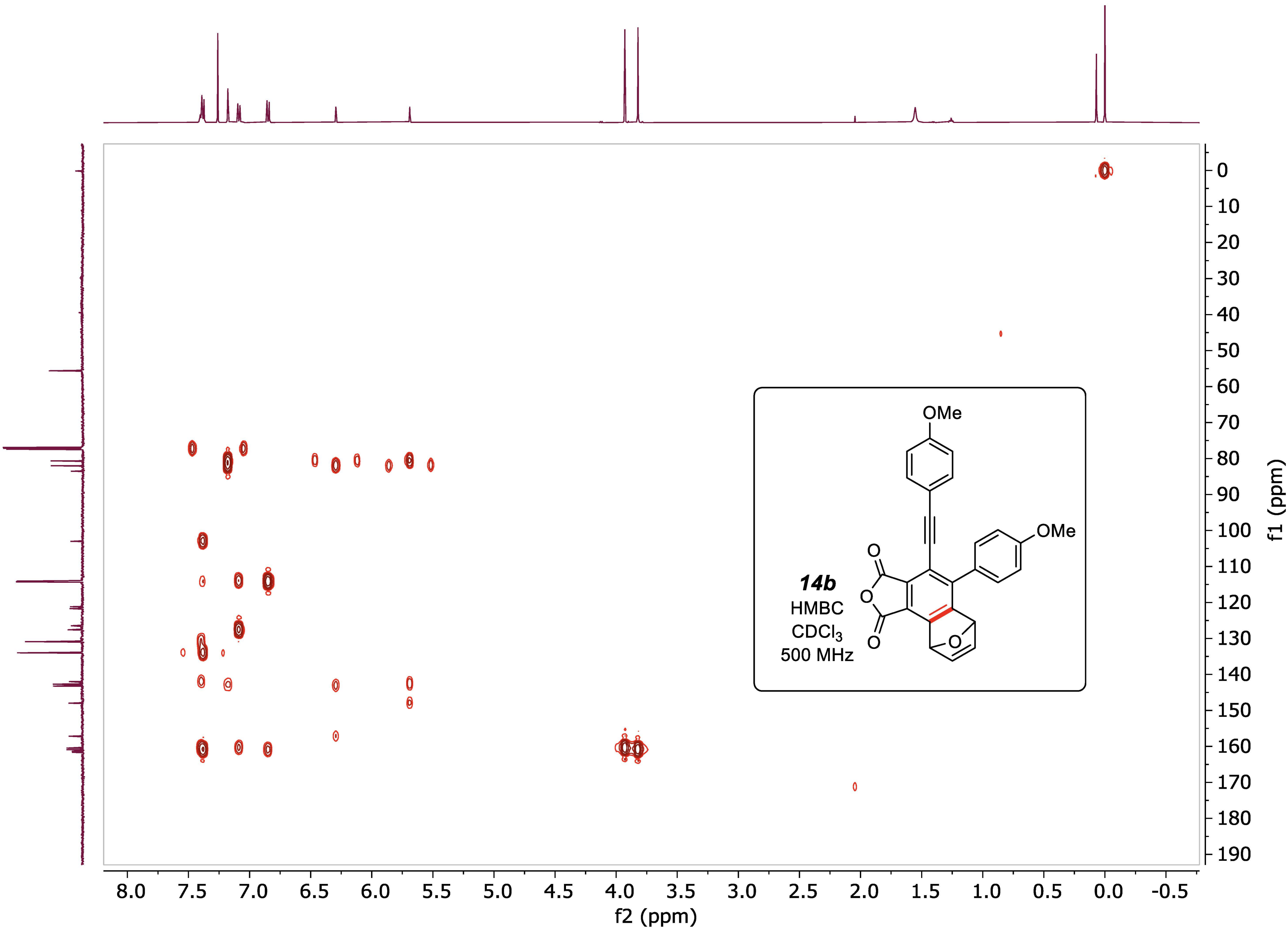

14b
HMBC
CDCl3
500 MHz
OMe
OMe
O
O
O
O
f2 (ppm)
f1 (ppm)

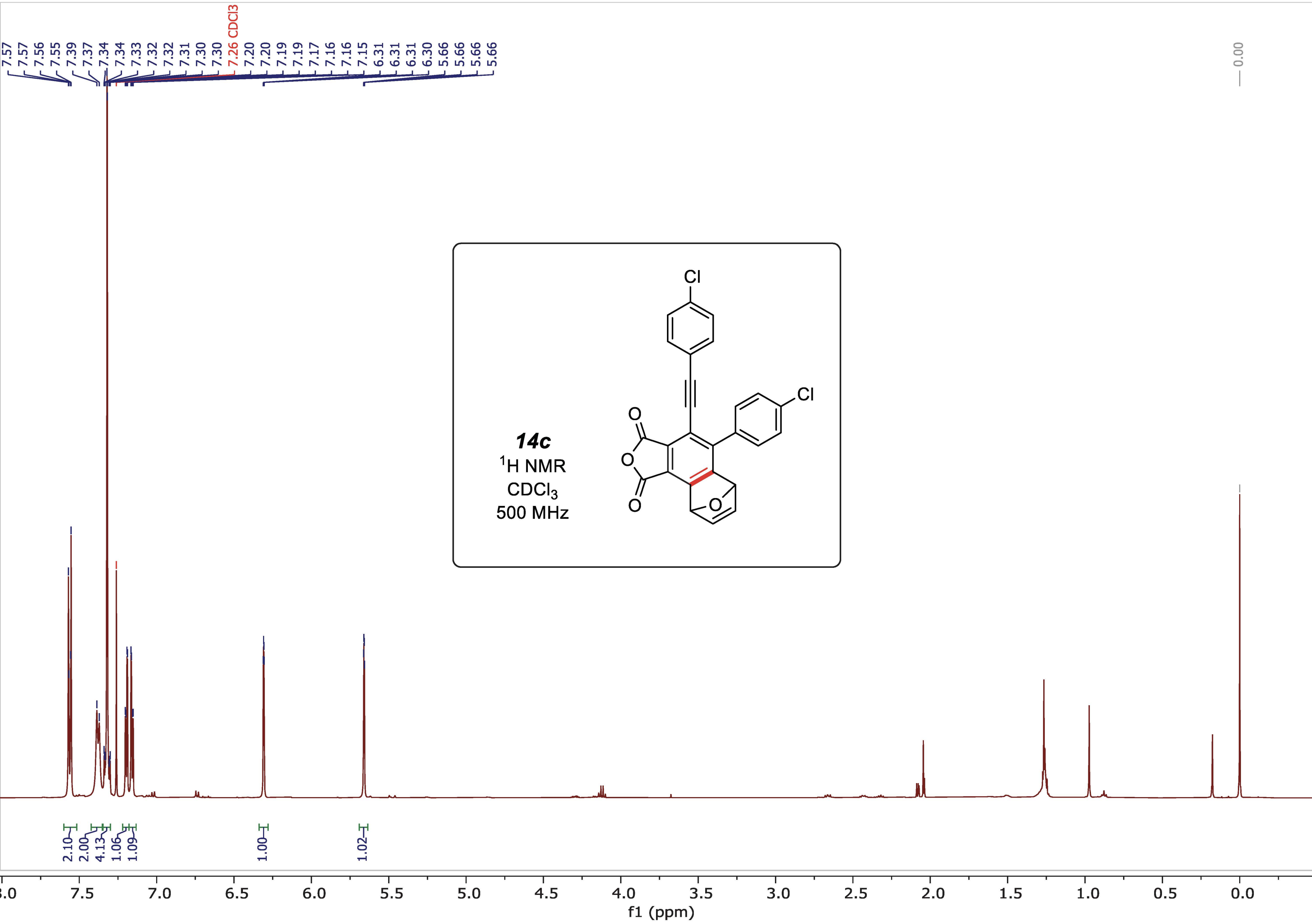
7.57
7.57
7.56
7.55
7.39
7.37
7.34
7.34
7.33
7.32
7.32
7.31
7.30
7.30
7.26 CDCl3
7.20
7.20
7.19
7.19
7.17
7.16
7.16
7.15
6.31
6.31
6.31
6.30
5.66
5.66
5.66
5.66
0.00
14c
1H NMR
CDCl3
500 MHz
Cl
Cl
O
O
O
O
2.10
2.00
4.13
1.06
1.09
1.00
1.02
f1 (ppm)

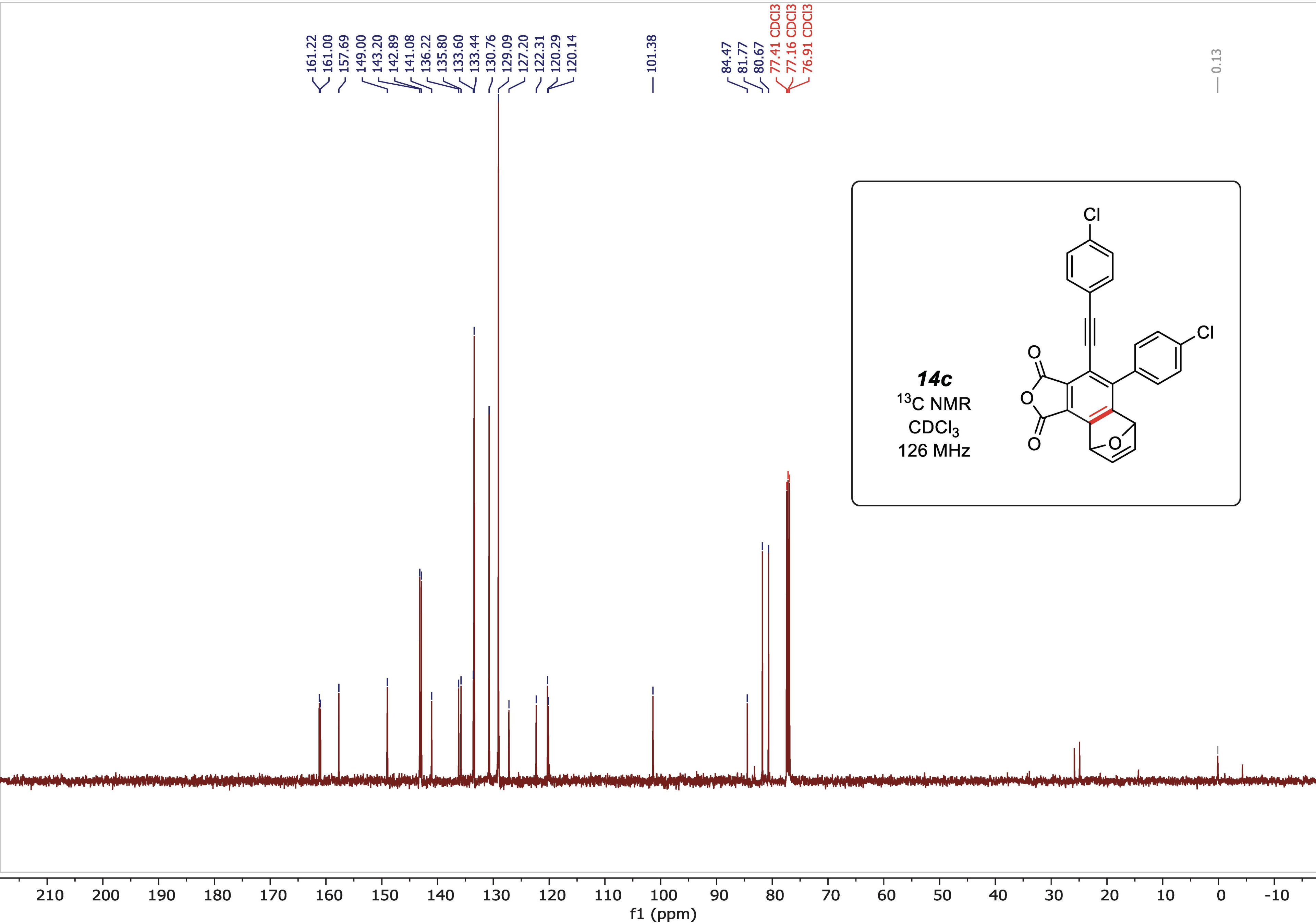
161.22
161.00
157.69
149.00
143.20
142.89
141.08
136.22
135.80
133.60
133.44
130.76
129.09
127.20
122.31
120.29
120.14
101.38
84.47
81.77
80.67
77.41 CDCl3
77.16 CDCl3
76.91 CDCl3
0.13
14c
13C NMR
CDCl3
126 MHz
Cl
Cl
O
O
O
O
210 200 190 180 170 160 150 140 130 120 110 100 90 80 70 60 50 40 30 20 10 0 -10
f1 (ppm)

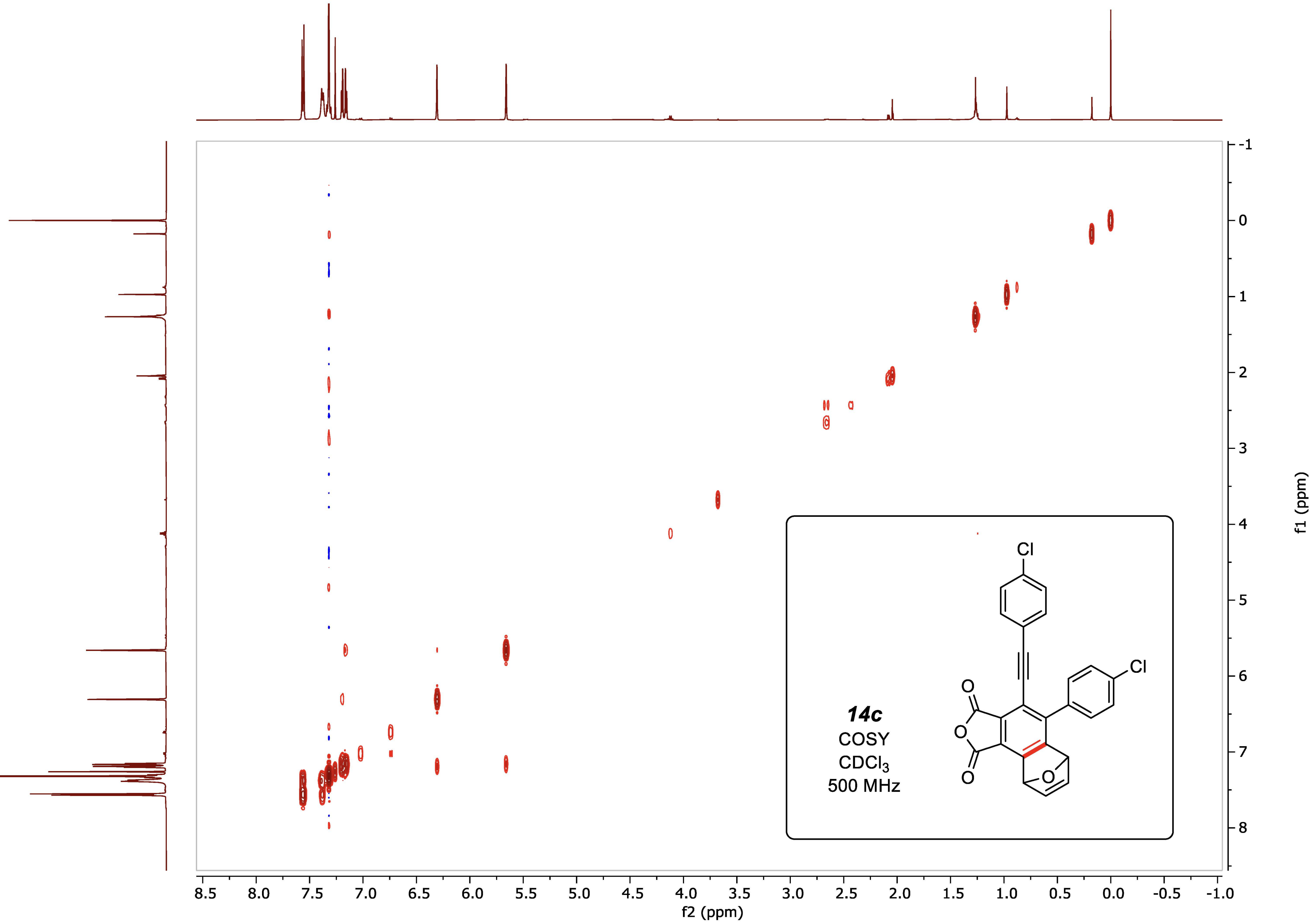
14c
COSY
CDCl3
500 MHz
Cl
Cl
O
O
O
O
f2 (ppm)
f1 (ppm)

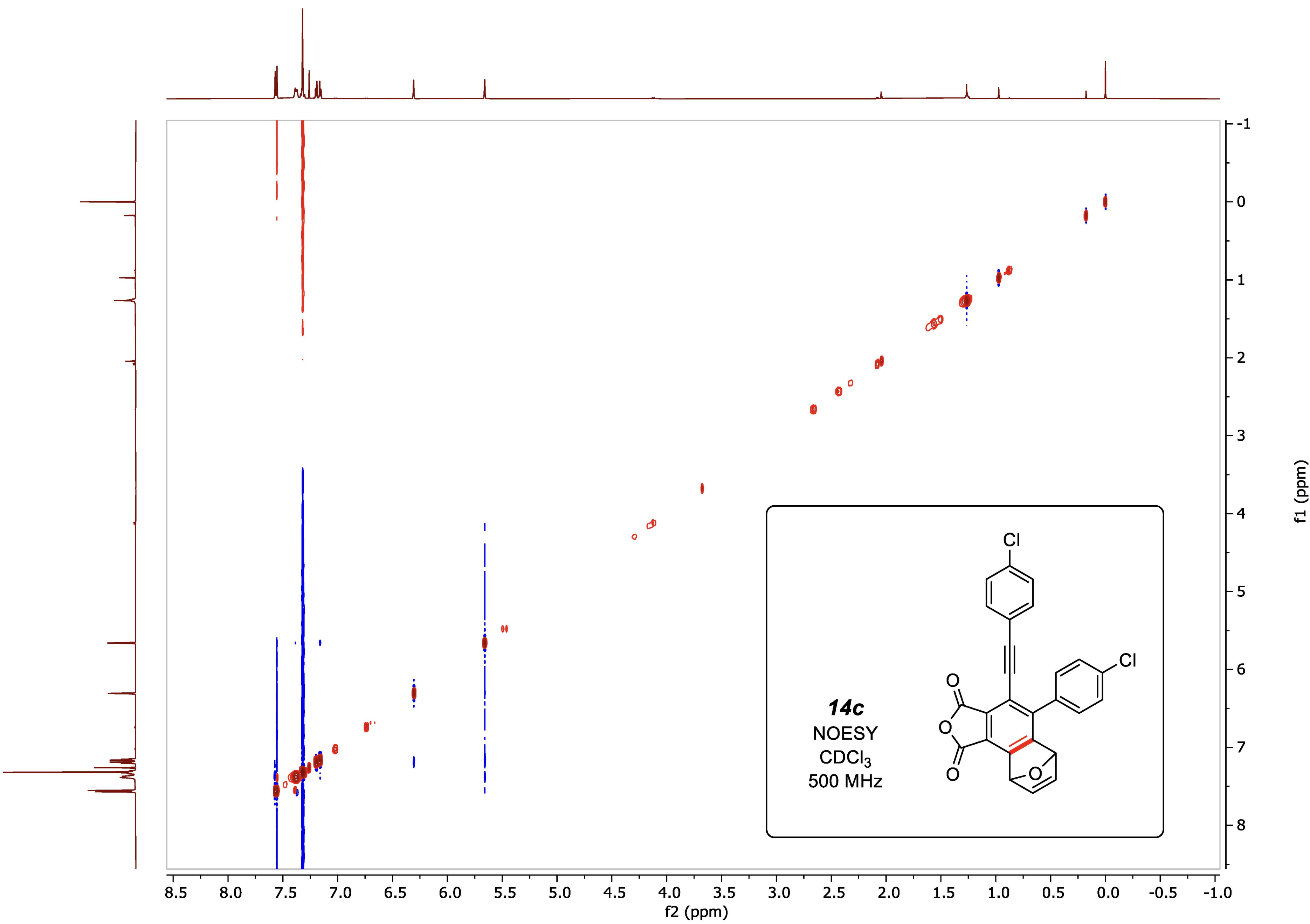

14c
NOESY
$CDCl_3$
500 MHz
Cl
Cl
O
O
O
O
f2 (ppm)
f1 (ppm)

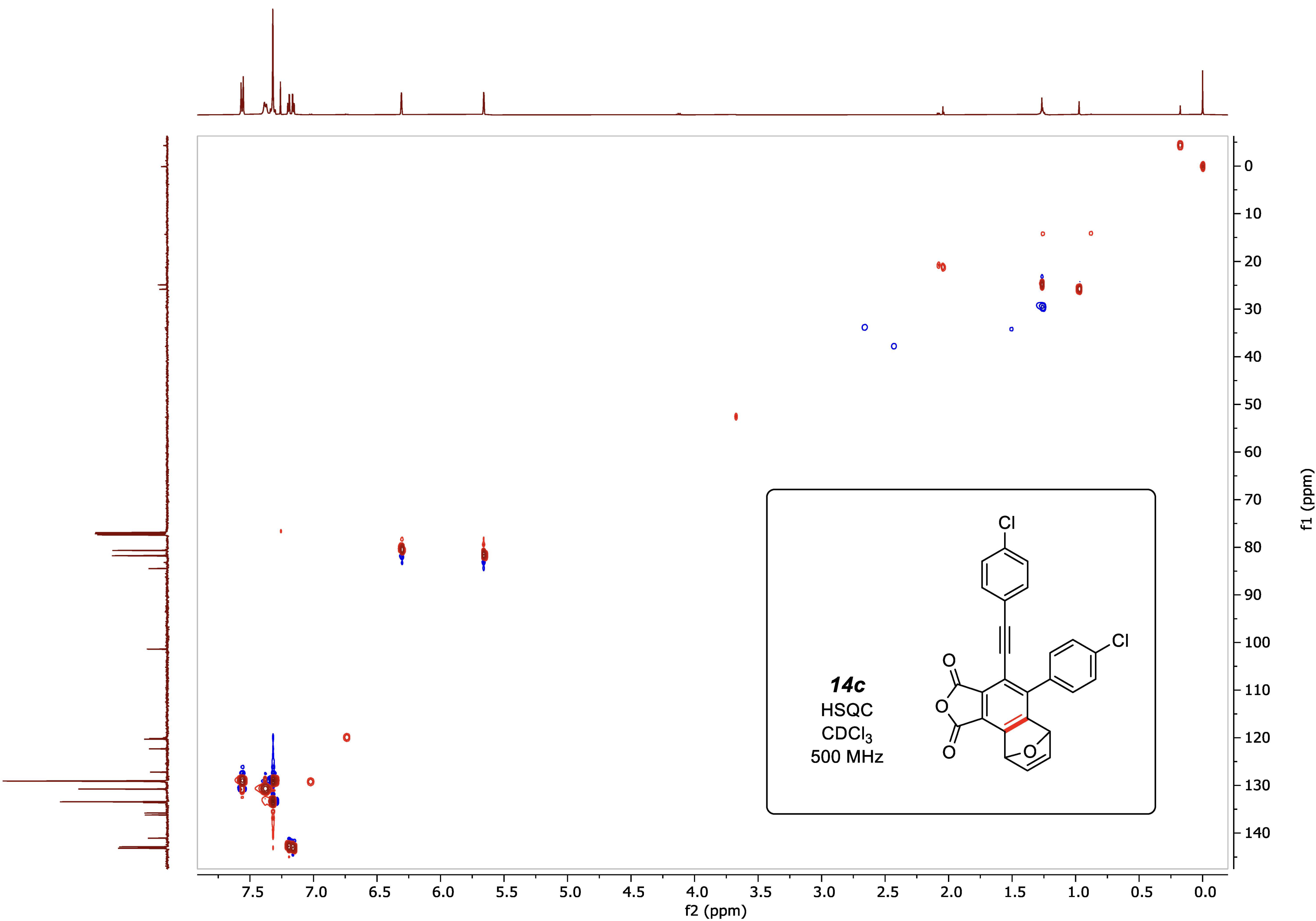
14c
HSQC
CDCl3
500 MHz
Cl
Cl
O
O
O
O
f2 (ppm)
f1 (ppm)

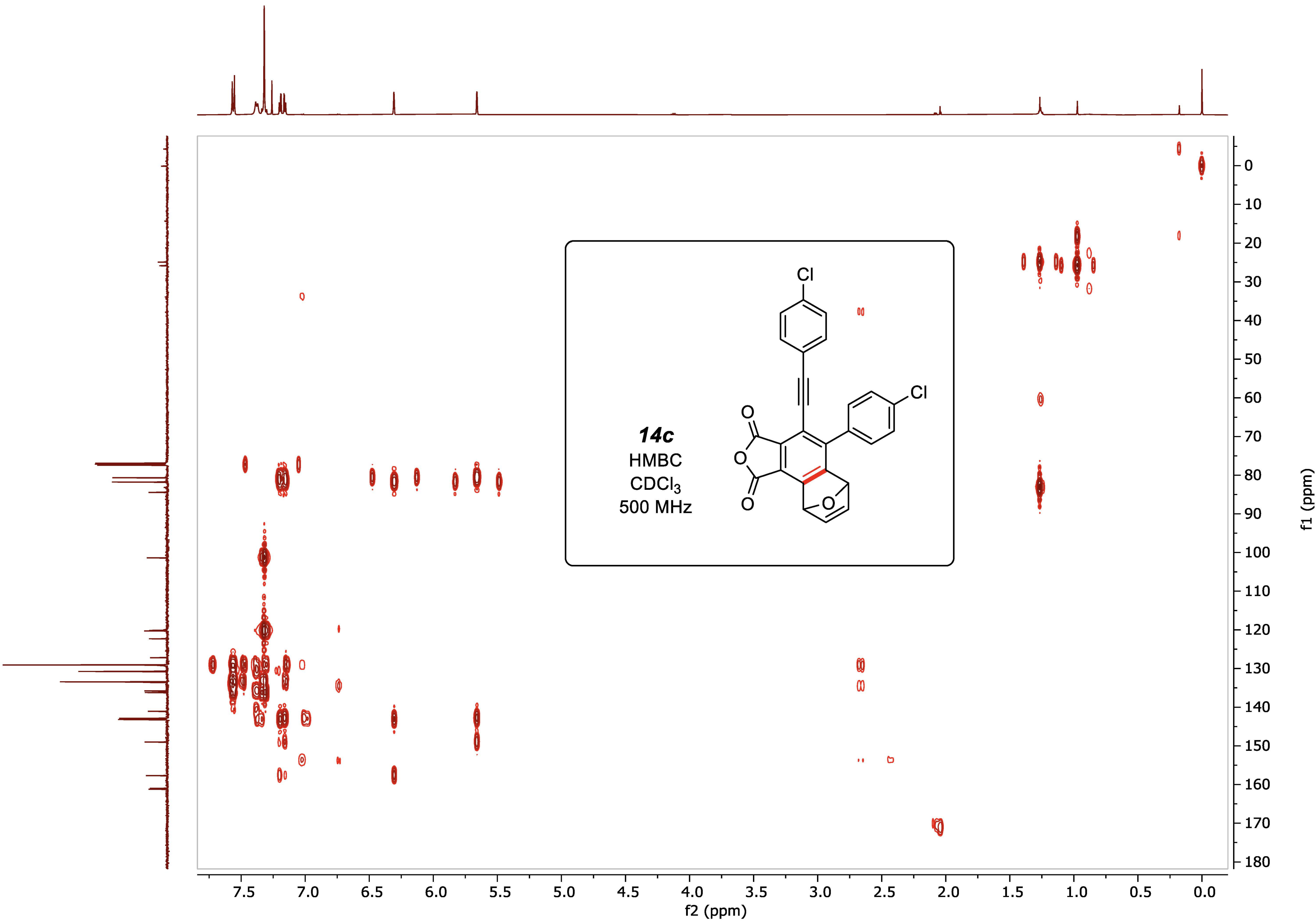
14c
HMBC
CDCl3
500 MHz
Cl
Cl
O
O
O
O
f2 (ppm)
f1 (ppm)

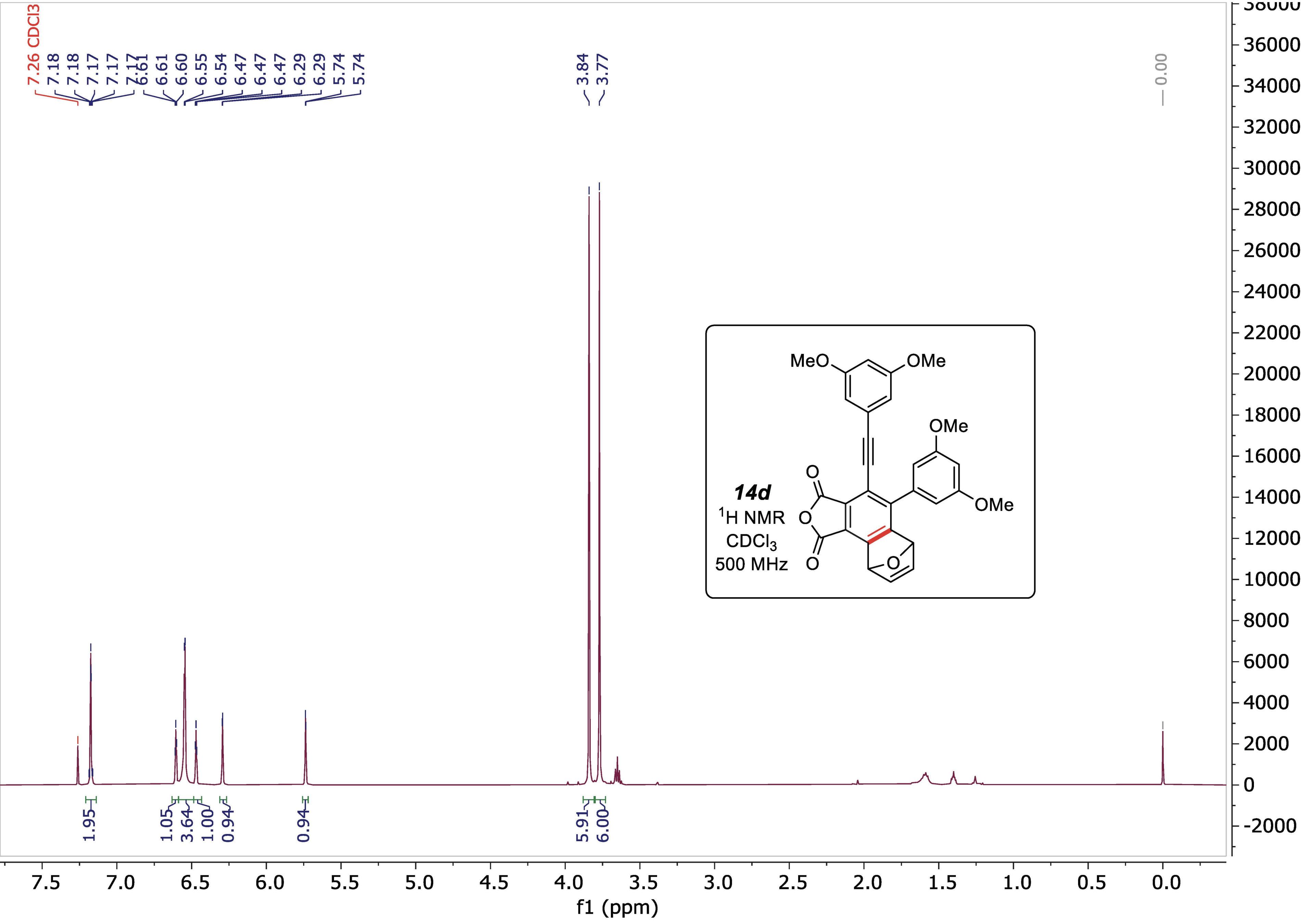
7.26 CDCl3
7.18
7.18
7.17
7.17
7.17
6.61
6.61
6.60
6.55
6.54
6.47
6.47
6.47
6.29
6.29
5.74
5.74
3.84
3.77
0.00
1.95
1.05
3.64
1.00
0.94
0.94
5.91
6.00
f1 (ppm)
14d
^{1}H NMR
$CDCl_3$
500 MHz
MeO
OMe
OMe
OMe
O
O
O
O

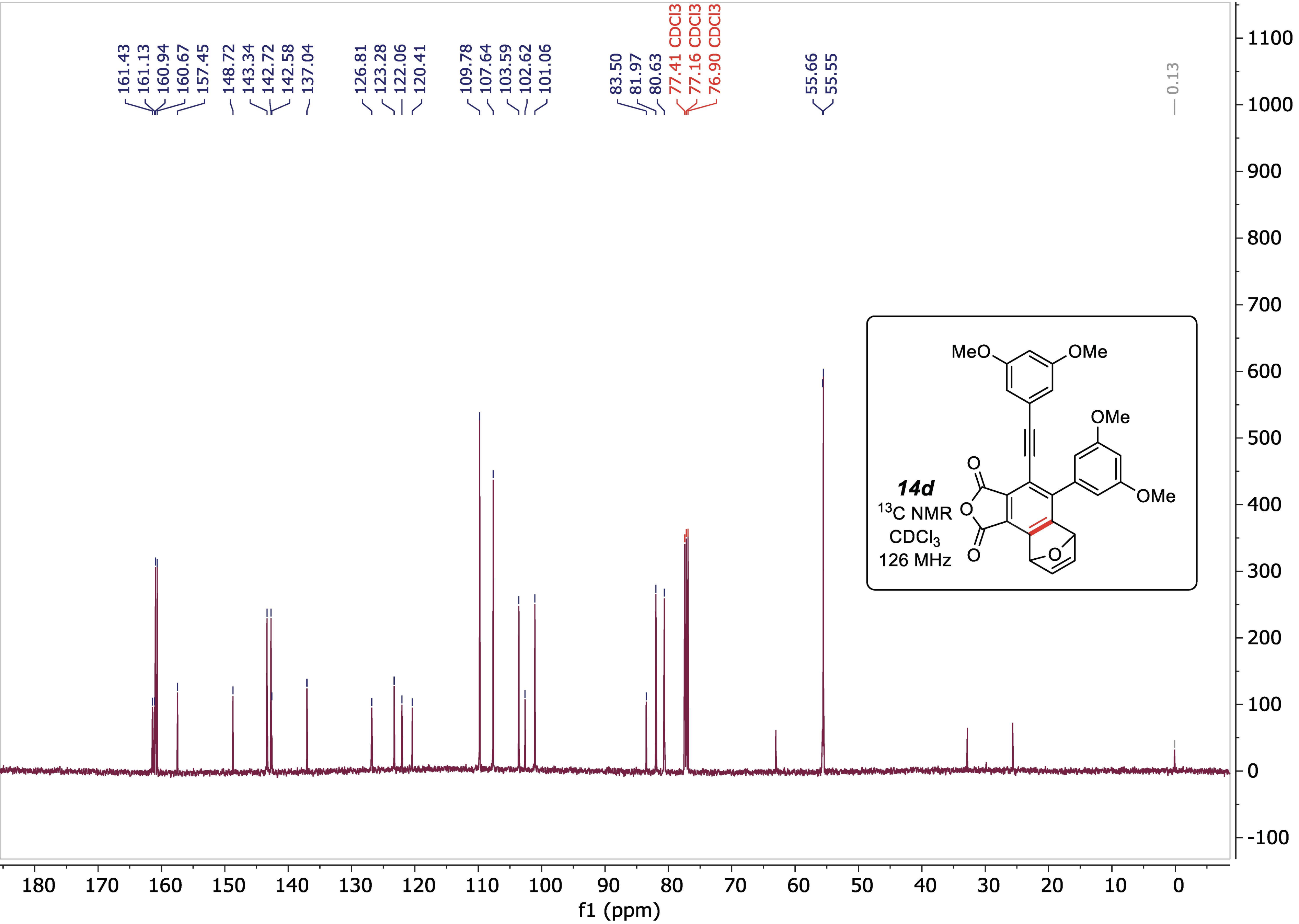
161.43
161.13
160.94
160.67
157.45
148.72
143.34
142.72
142.58
137.04
126.81
123.28
122.06
120.41
109.78
107.64
103.59
102.62
101.06
83.50
81.97
80.63
77.41 CDCl3
77.16 CDCl3
76.90 CDCl3
55.66
55.55
0.13
MeO
OMe
OMe
OMe
O
O
O
O
14d
13C NMR
CDCl3
126 MHz
f1 (ppm)

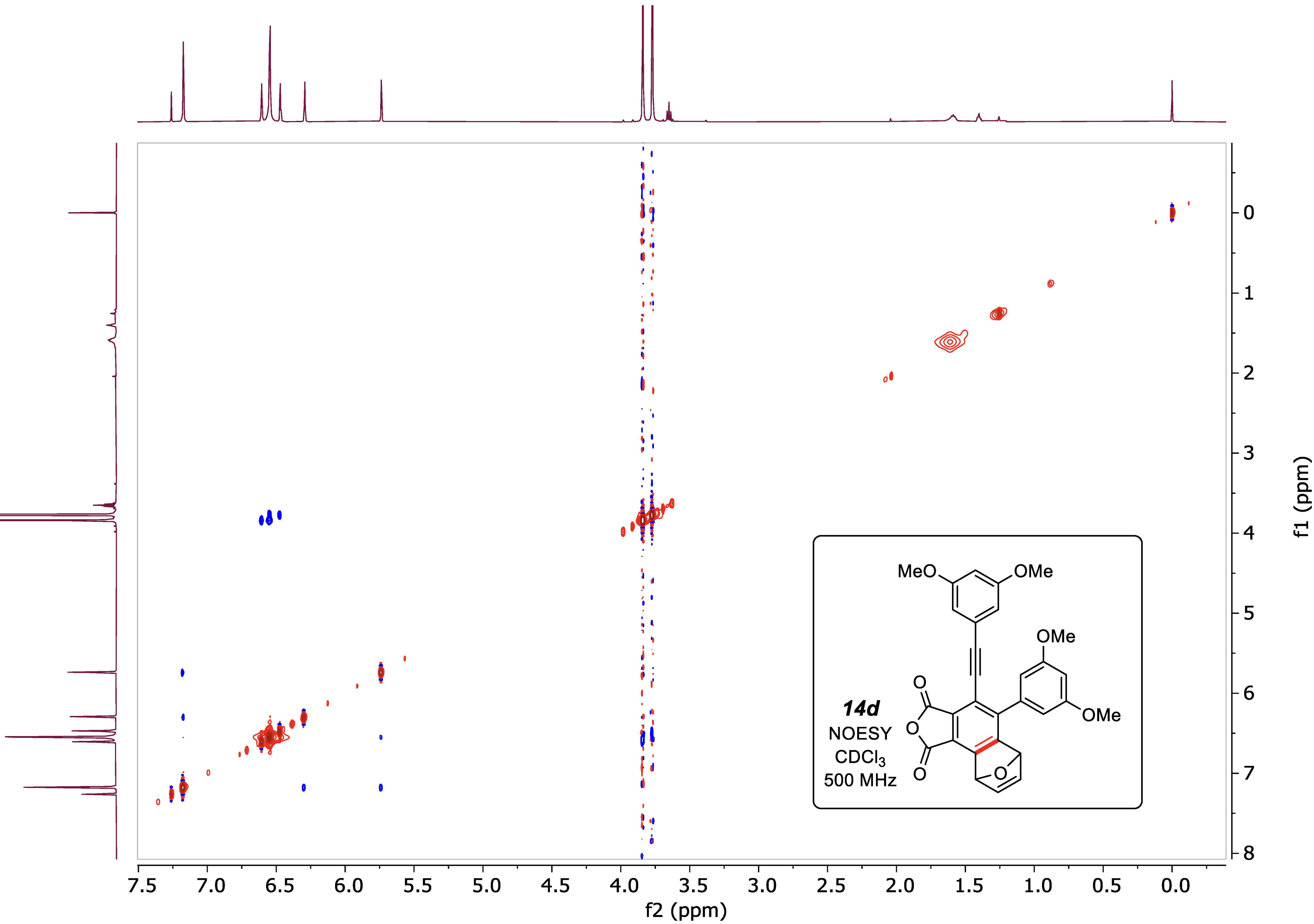
14d
NOESY
$CDCl_3$
500 MHz
MeO
OMe
OMe
OMe
O
O
O
O
f2 (ppm)
f1 (ppm)
7.5 7.0 6.5 6.0 5.5 5.0 4.5 4.0 3.5 3.0 2.5 2.0 1.5 1.0 0.5 0.0
0 1 2 3 4 5 6 7 8

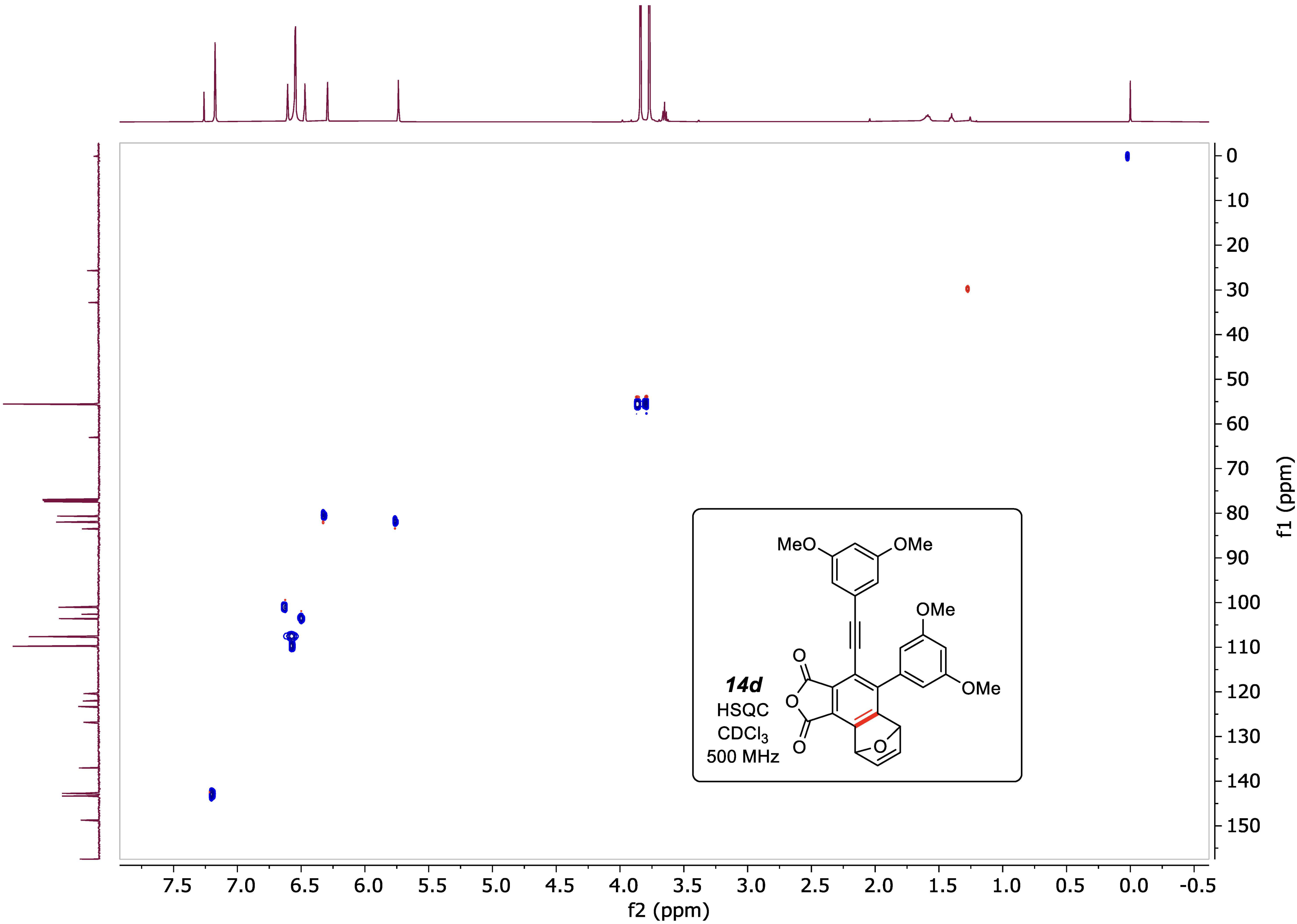

f1 (ppm)
f2 (ppm)
MeO
OMe
OMe
OMe
O
O
O
O
14d
HSQC
CDCl3
500 MHz

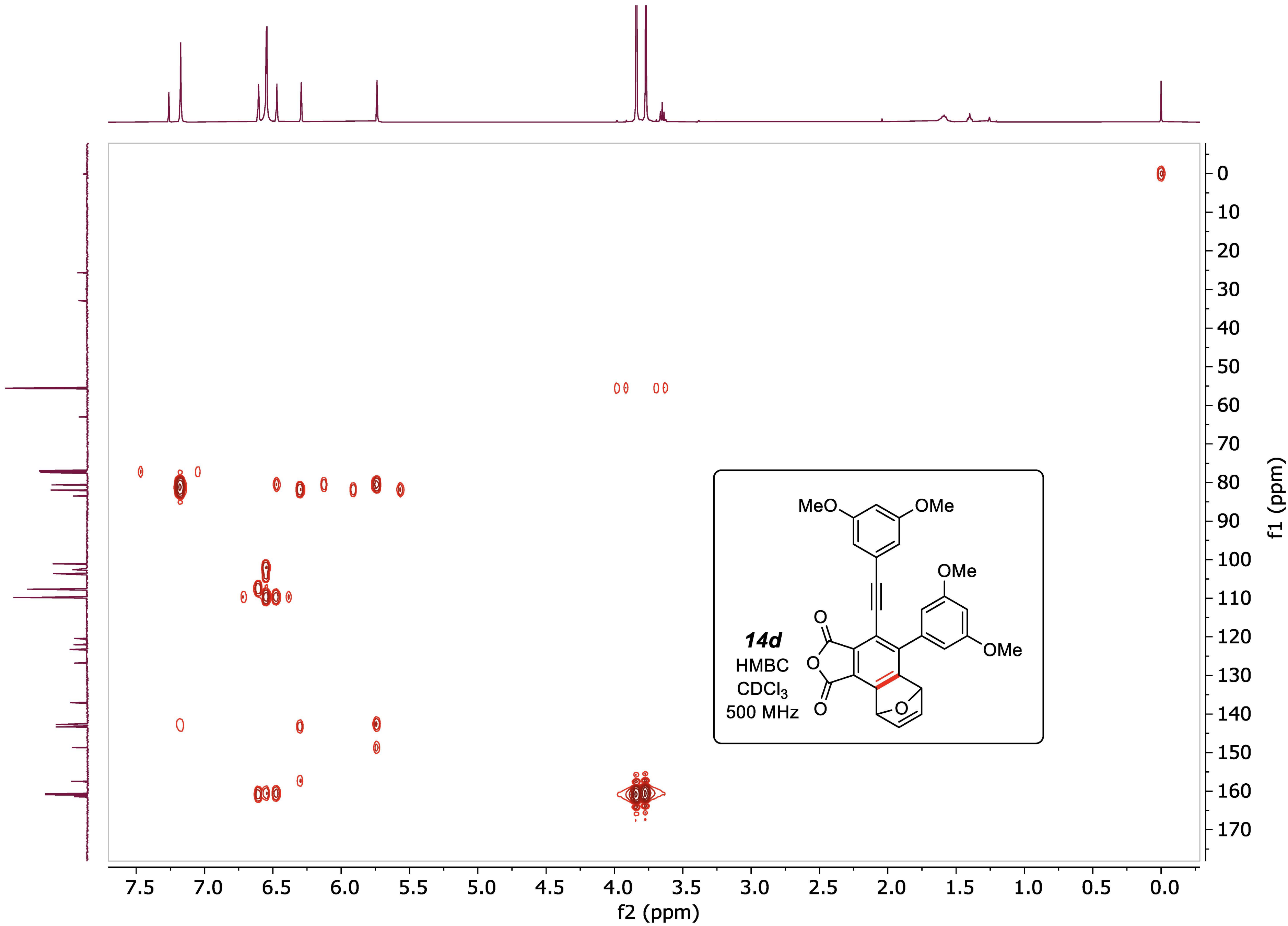
14d
HMBC
CDCl3
500 MHz
MeO
OMe
OMe
OMe
O
O
O
O
f1 (ppm)
f2 (ppm)

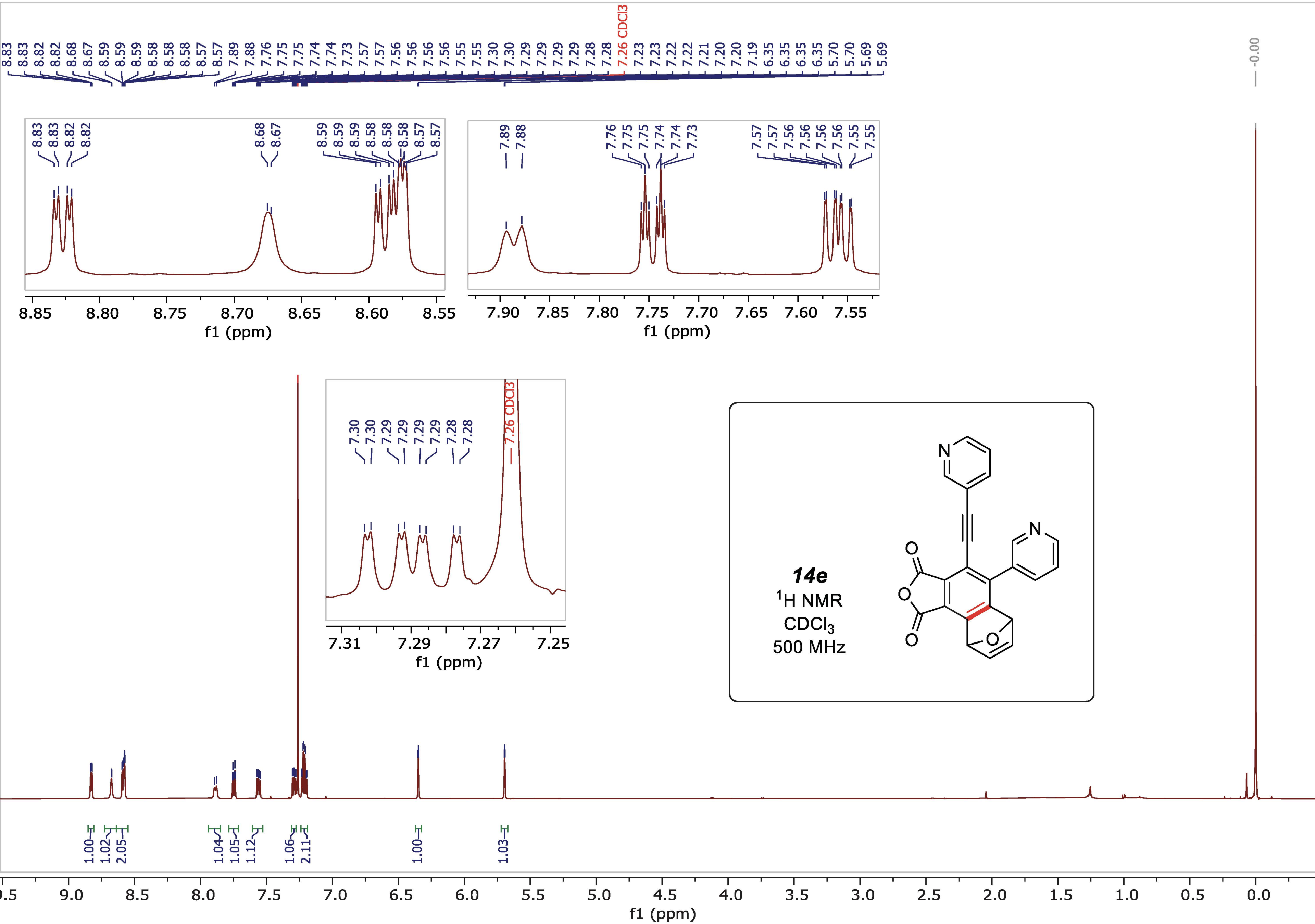
14e
1H NMR
CDCl3
500 MHz
7.26 CDCl3
f1 (ppm)

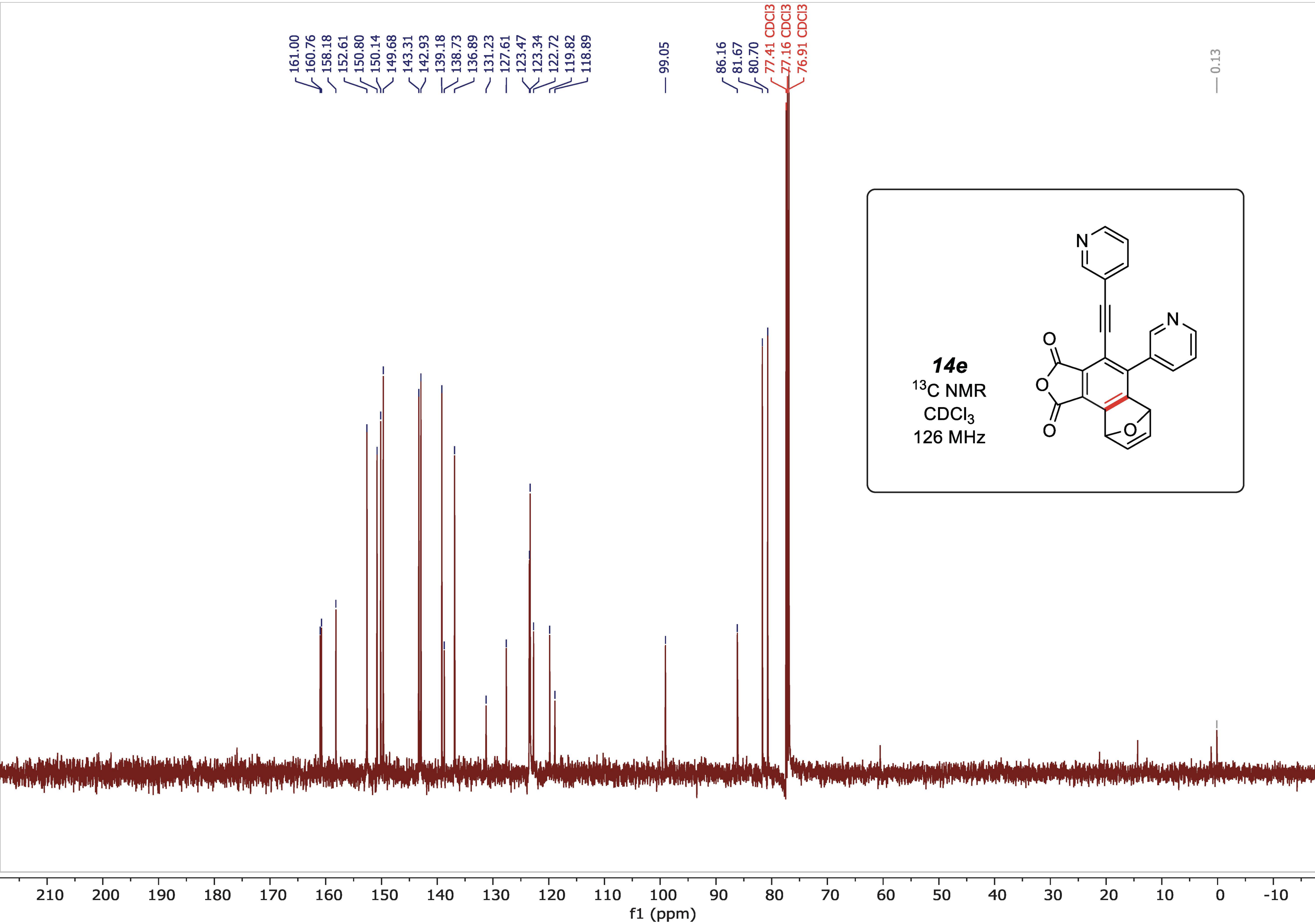
161.00
160.76
158.18
152.61
150.80
150.14
149.68
143.31
142.93
139.18
138.73
136.89
131.23
127.61
123.47
123.34
122.72
119.82
118.89
99.05
86.16
81.67
80.70
77.41 CDCl3
77.16 CDCl3
76.91 CDCl3
0.13
14e
13C NMR
CDCl3
126 MHz
f1 (ppm)

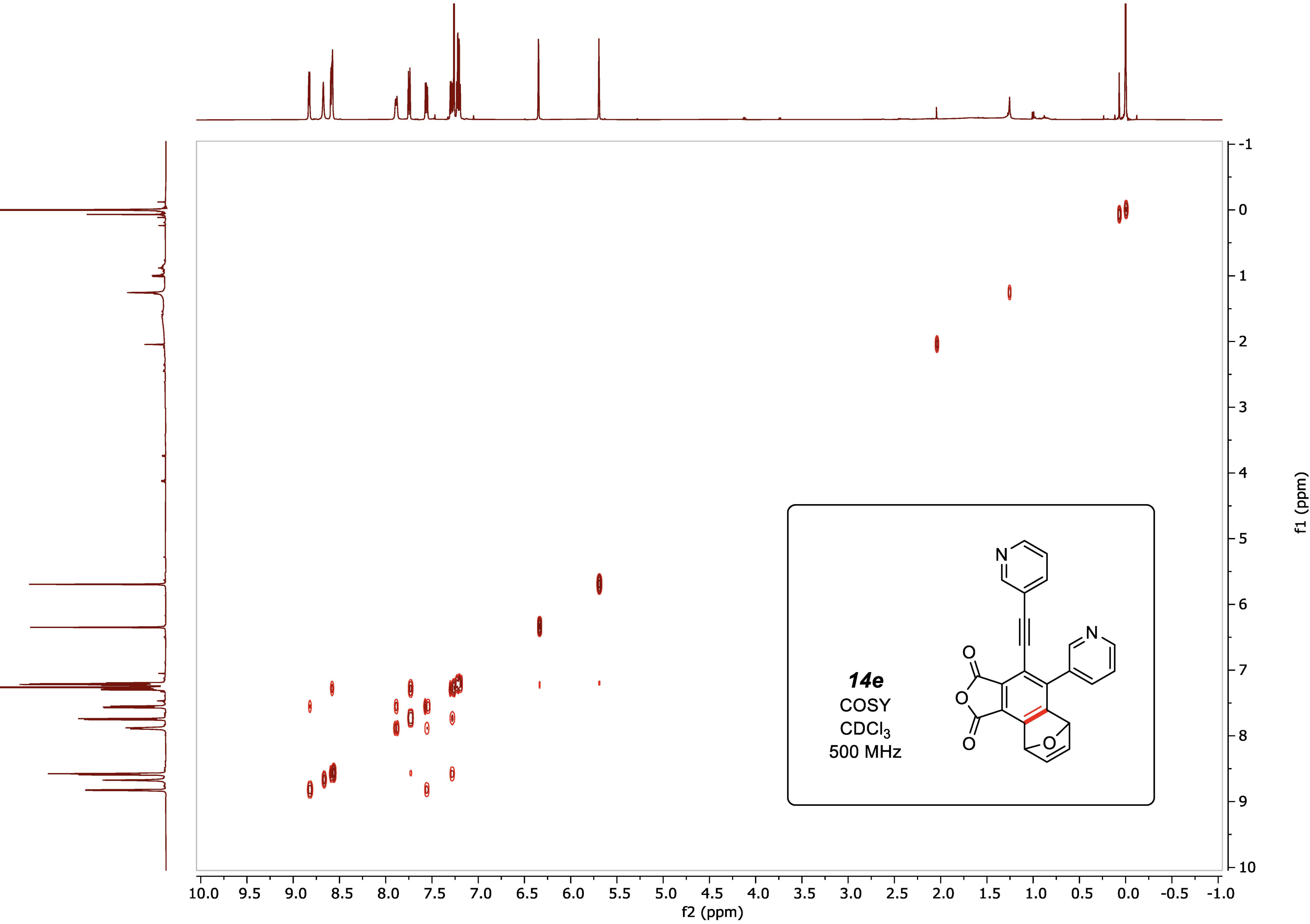
14e
COSY
$CDCl_3$
500 MHz
f2 (ppm)
f1 (ppm)

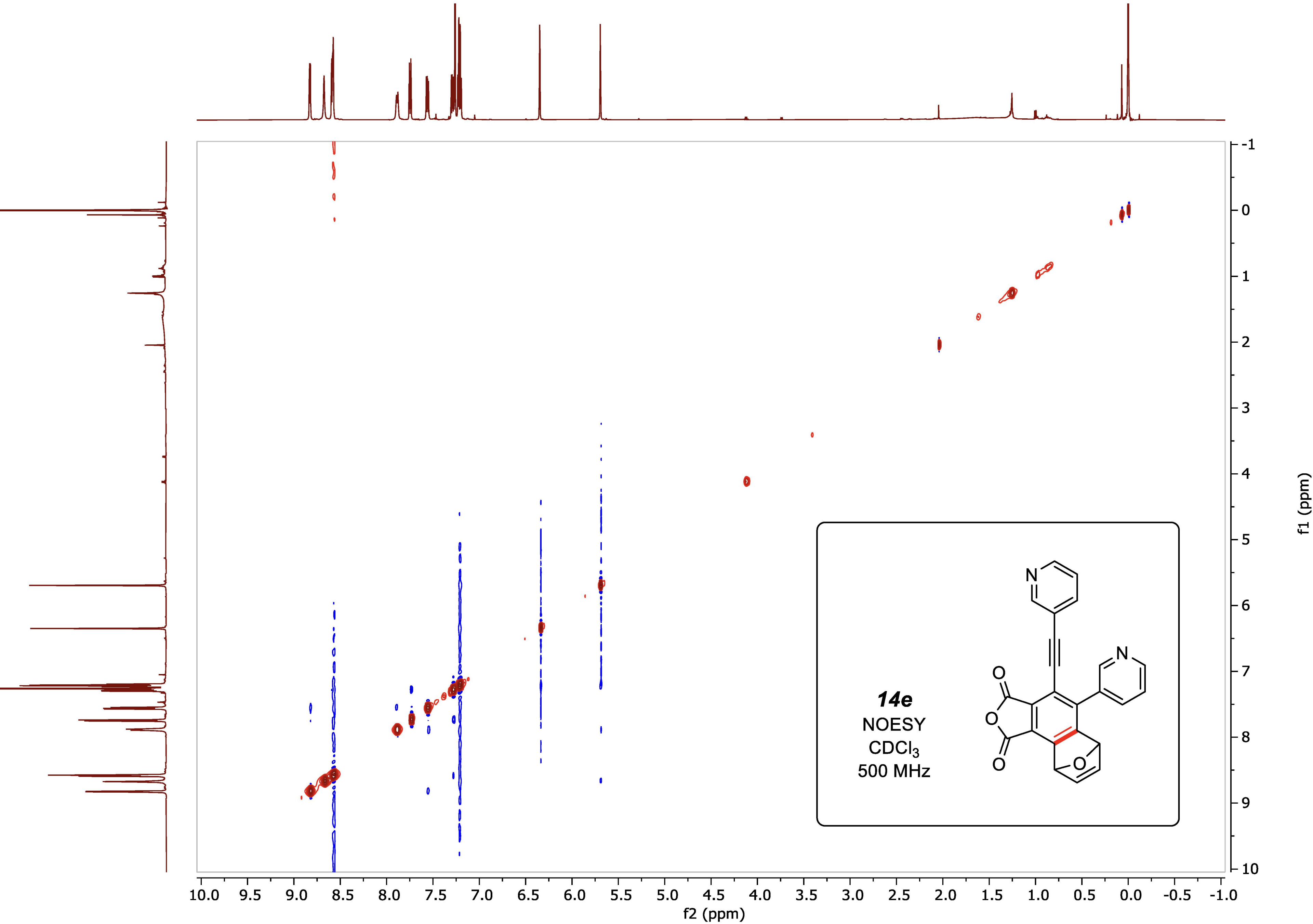
f2 (ppm)
f1 (ppm)
14e
NOESY
CDCl3
500 MHz

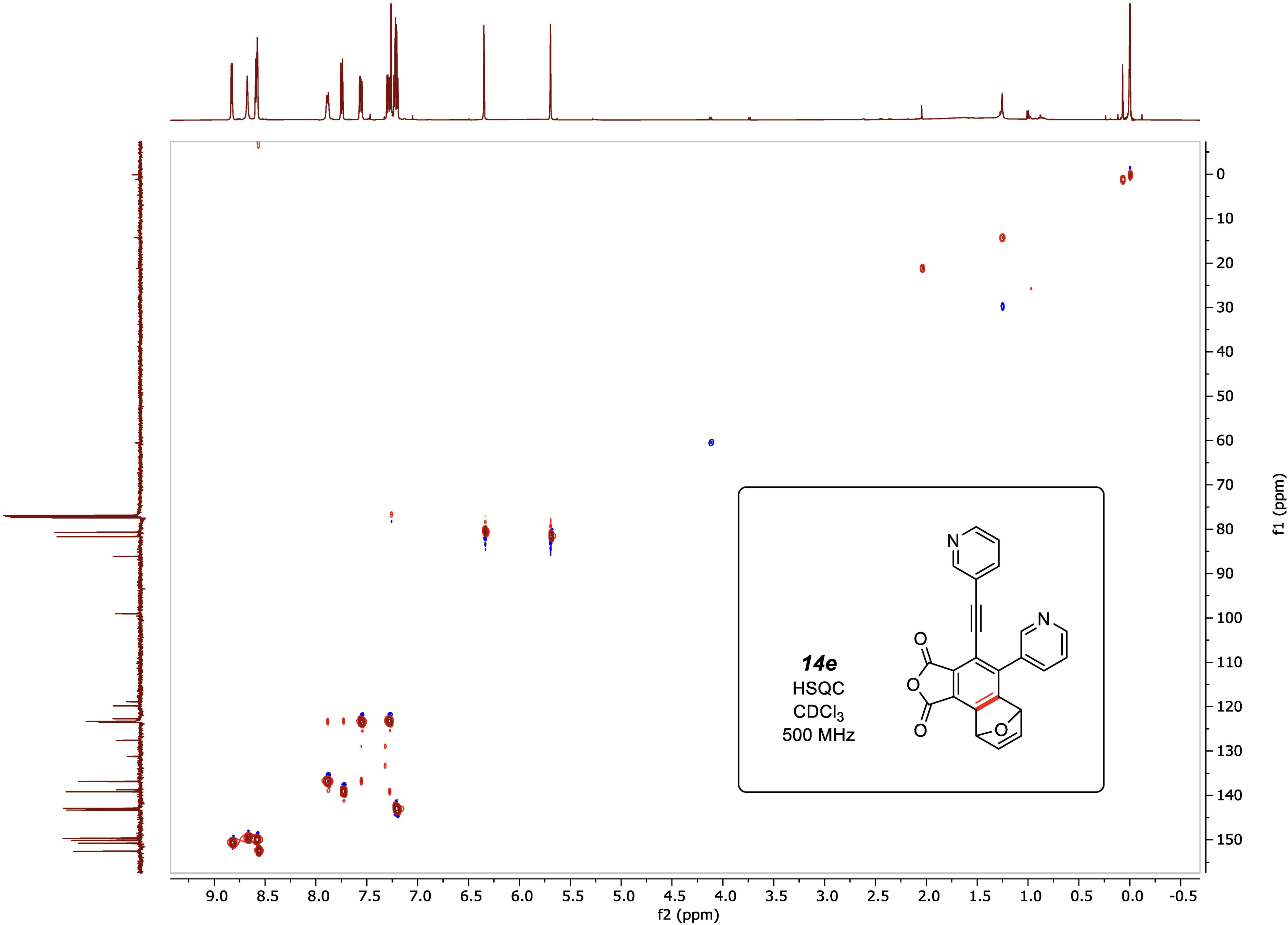
14e
HSQC
CDCl3
500 MHz
N
N
O
O
O
O
f1 (ppm)
f2 (ppm)

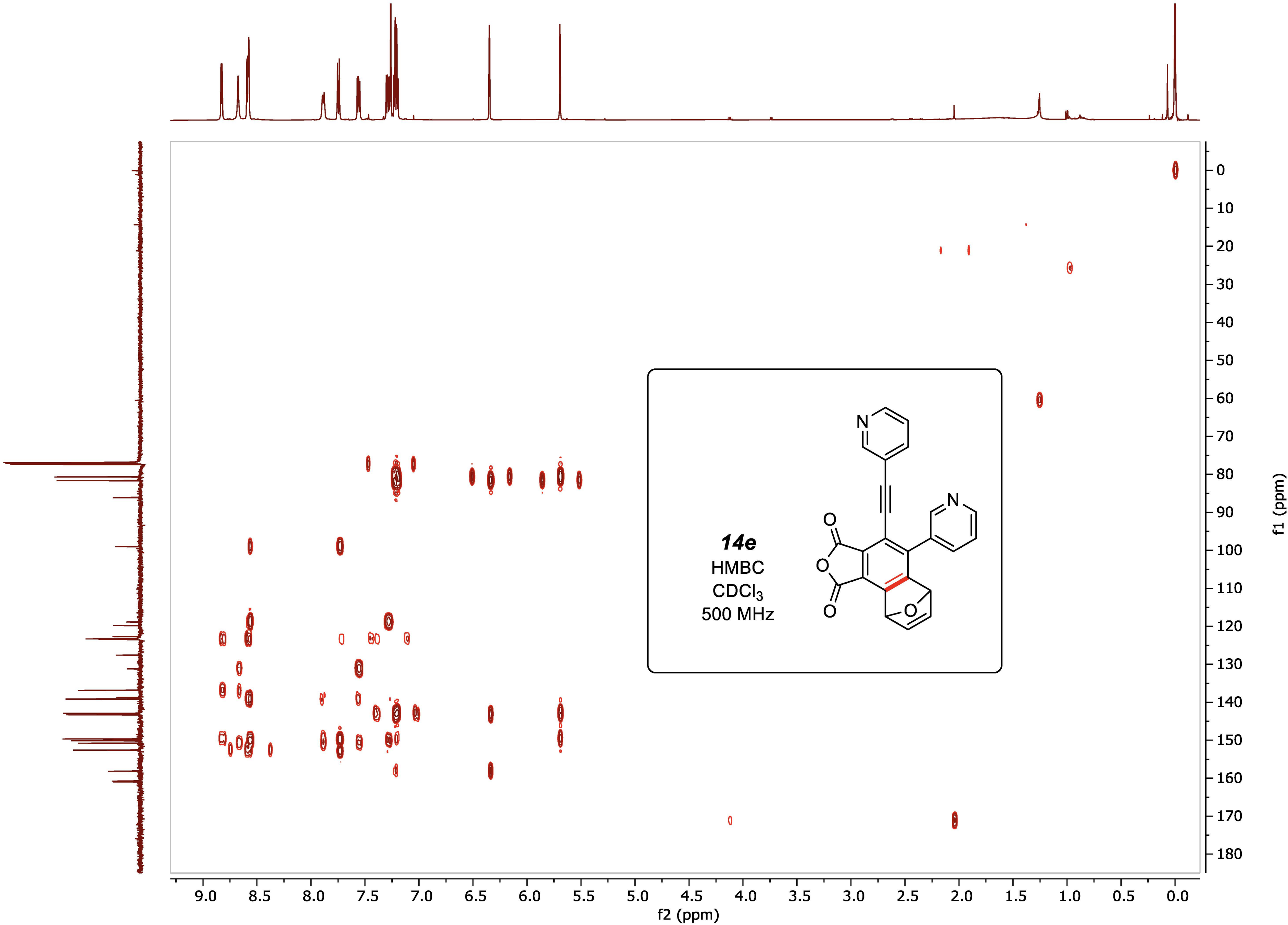
14e
HMBC
CDCl3
500 MHz
f2 (ppm)
f1 (ppm)

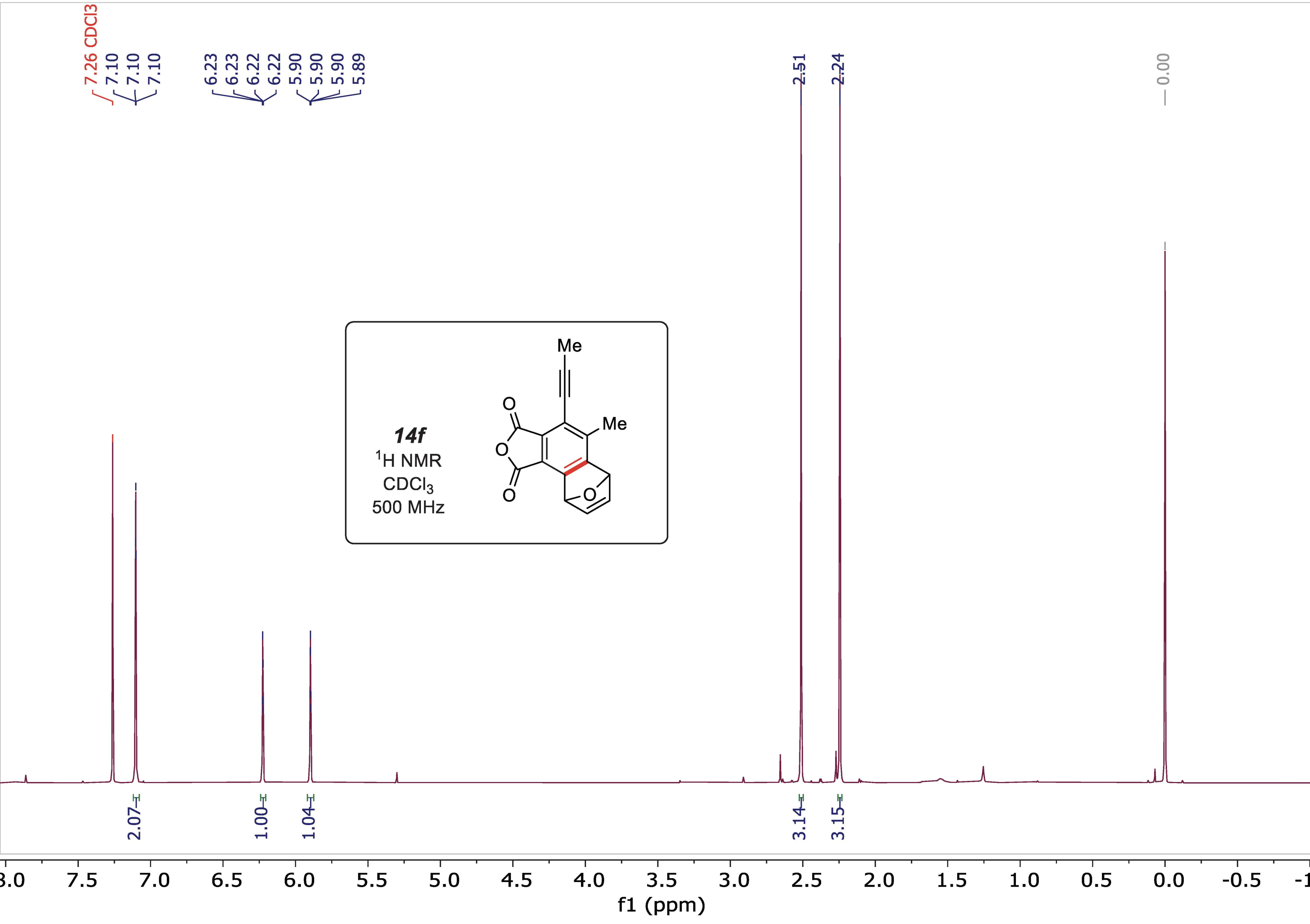
7.26 CDCl3
7.10
7.10
7.10
6.23
6.23
6.22
6.22
5.90
5.90
5.90
5.89
2.51
2.24
0.00
14f
1H NMR
CDCl3
500 MHz
Me
Me
O
O
O
O
2.07
1.00
1.04
3.14
3.15
8.0
7.5
7.0
6.5
6.0
5.5
5.0
4.5
4.0
3.5
3.0
2.5
2.0
1.5
1.0
0.5
0.0
-0.5
f1 (ppm)

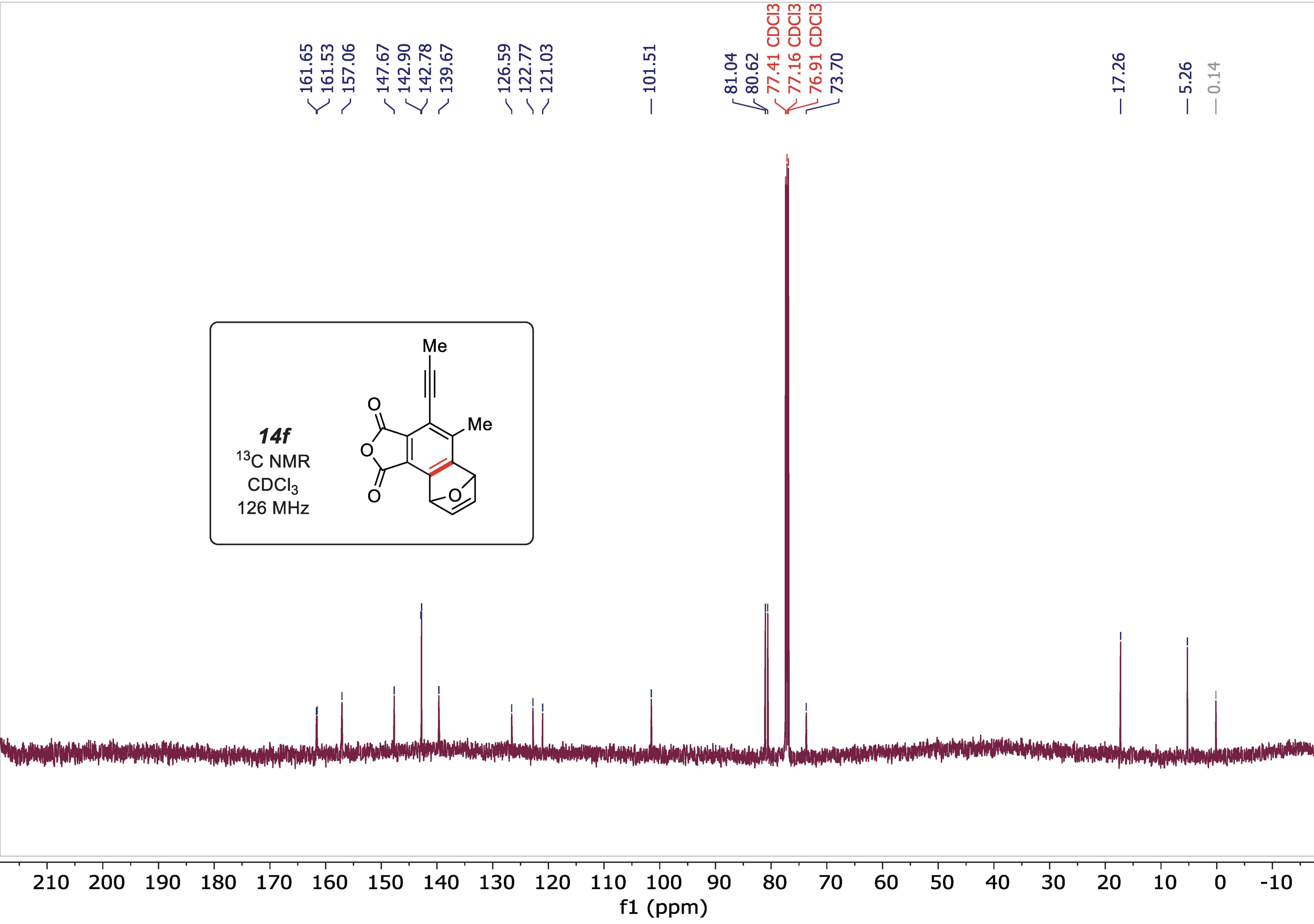
161.65
161.53
157.06
147.67
142.90
142.78
139.67
126.59
122.77
121.03
101.51
81.04
80.62
77.41 CDCl3
77.16 CDCl3
76.91 CDCl3
73.70
17.26
5.26
0.14
14f
13C NMR
CDCl3
126 MHz
Me
O
O
O
Me
O
210 200 190 180 170 160 150 140 130 120 110 100 90 80 70 60 50 40 30 20 10 0 -10
f1 (ppm)

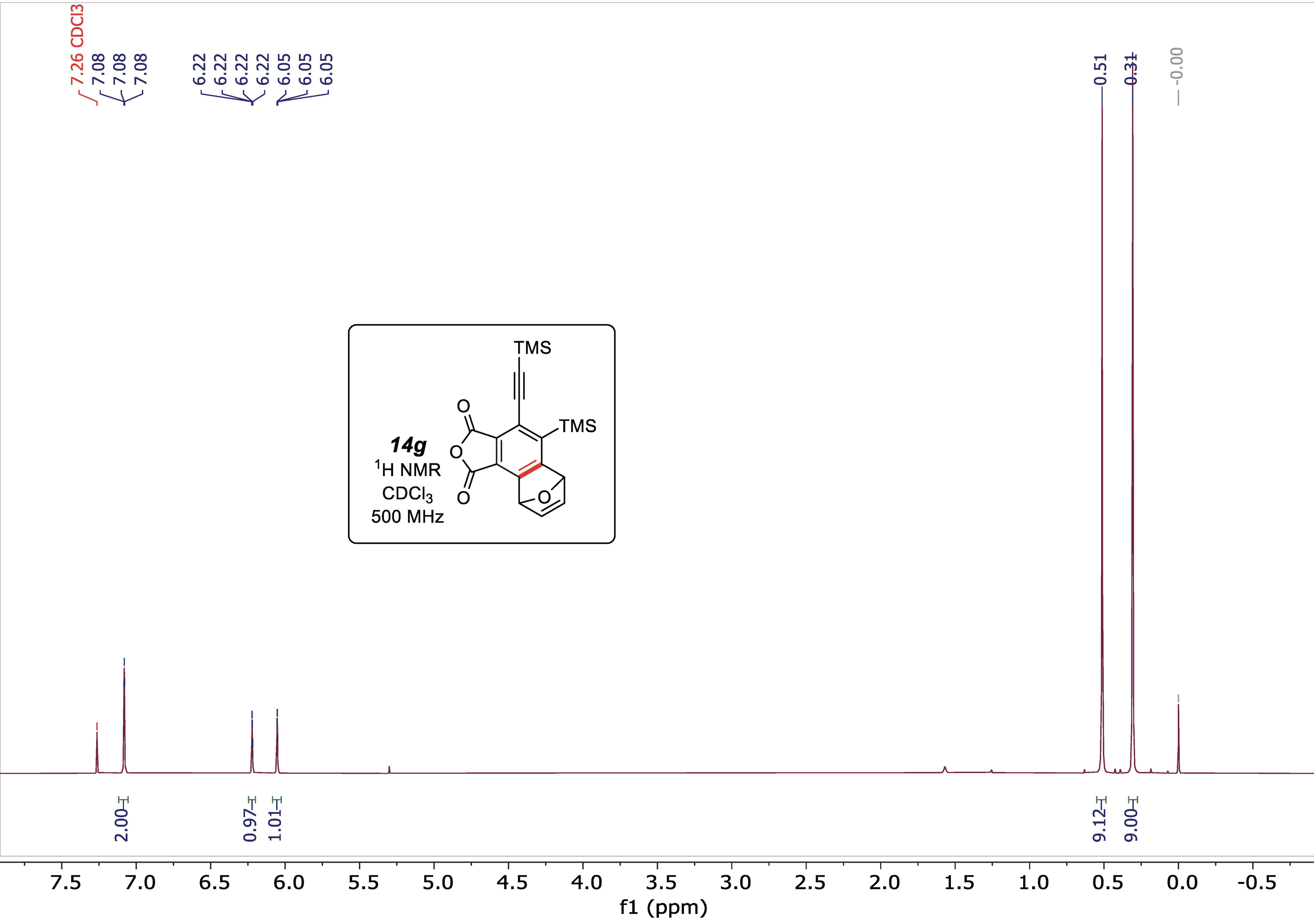
7.26 CDCl3
7.08
7.08
7.08
6.22
6.22
6.22
6.22
6.05
6.05
6.05
0.51
0.31
-0.00
TMS
TMS
O
O
O
O
14g
1H NMR
CDCl3
500 MHz
2.00
0.97
1.01
9.12
9.00
7.5
7.0
6.5
6.0
5.5
5.0
4.5
4.0
3.5
3.0
2.5
2.0
1.5
1.0
0.5
0.0
-0.5
f1 (ppm)

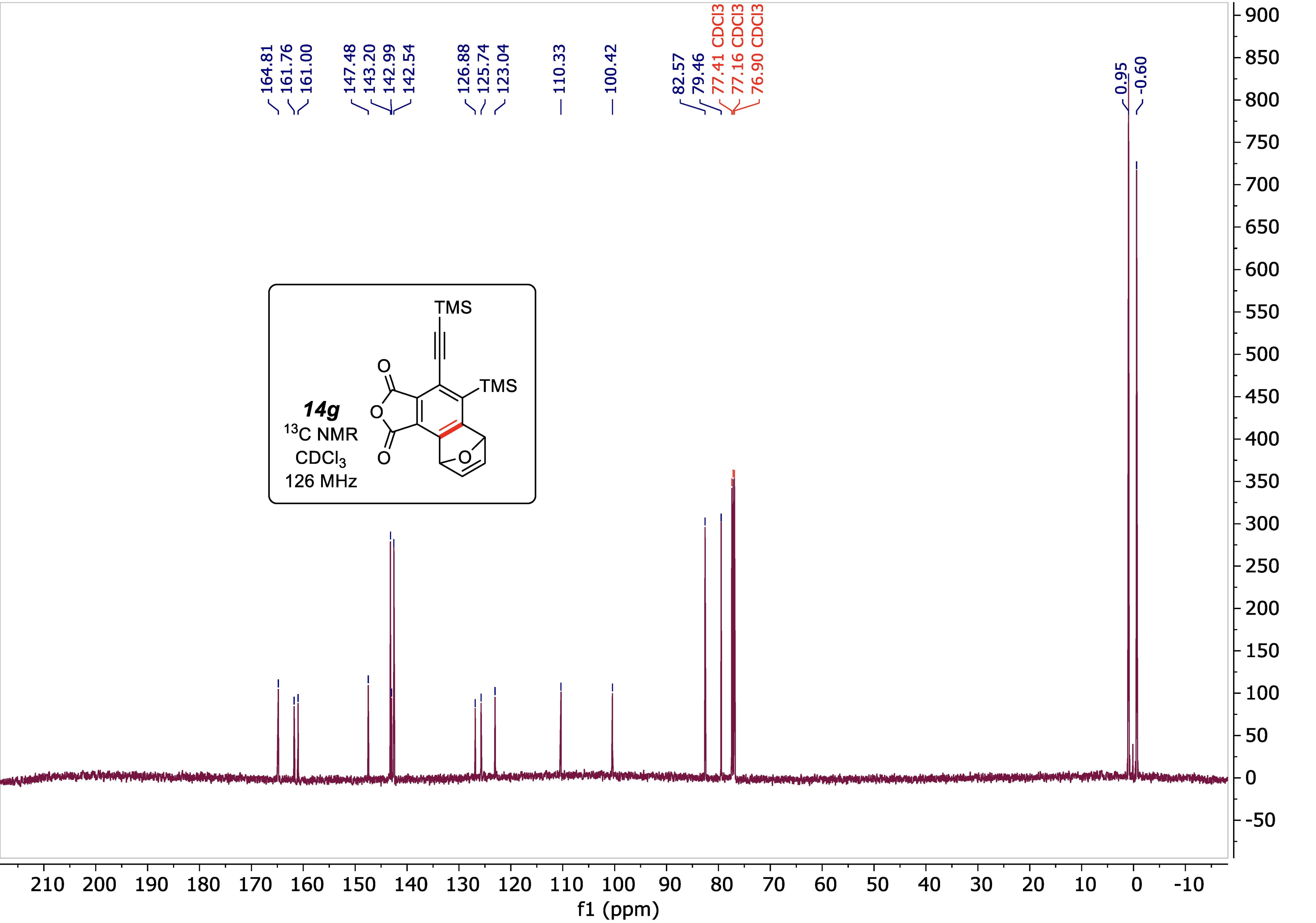

164.81
161.76
161.00
147.48
143.20
142.99
142.54
126.88
125.74
123.04
110.33
100.42
82.57
79.46
77.41 CDCl3
77.16 CDCl3
76.90 CDCl3
0.95
-0.60
TMS
TMS
O
O
O
O
14g
13C NMR
CDCl3
126 MHz
f1 (ppm)

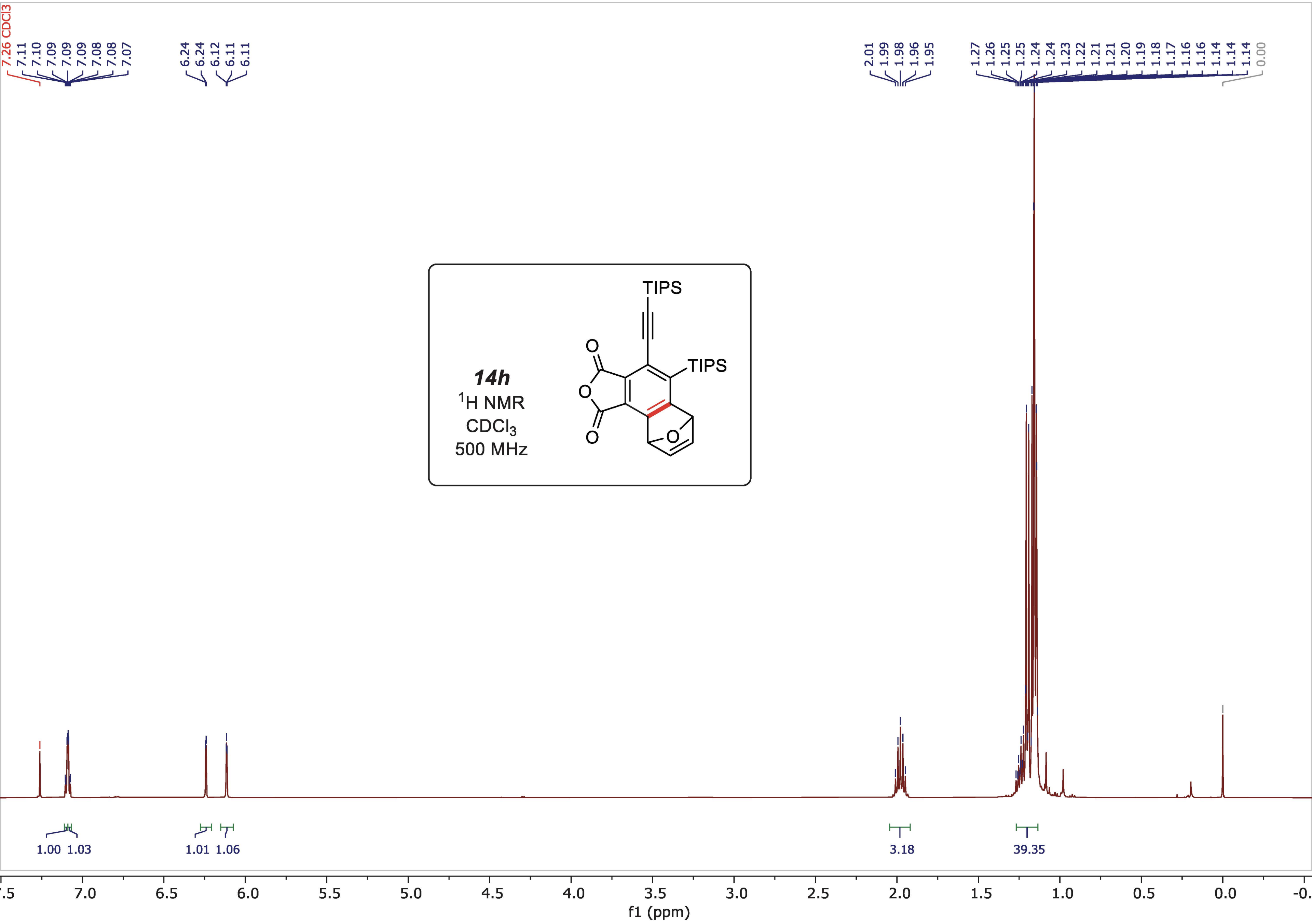
7.26 CDCl3
7.11 7.10 7.09 7.09 7.09 7.08 7.08 7.07
6.24 6.24 6.12 6.11 6.11
2.01 1.99 1.98 1.96 1.95
1.27 1.26 1.25 1.25 1.24 1.24 1.23 1.22 1.21 1.21 1.20 1.19 1.18 1.17 1.16 1.16 1.14 1.14 1.14
0.00
14h
1H NMR
CDCl3
500 MHz
TIPS
TIPS
O
O
O
O
1.00 1.03 1.01 1.06 3.18 39.35
7.0 6.5 6.0 5.5 5.0 4.5 4.0 3.5 3.0 2.5 2.0 1.5 1.0 0.5 0.0
f1 (ppm)

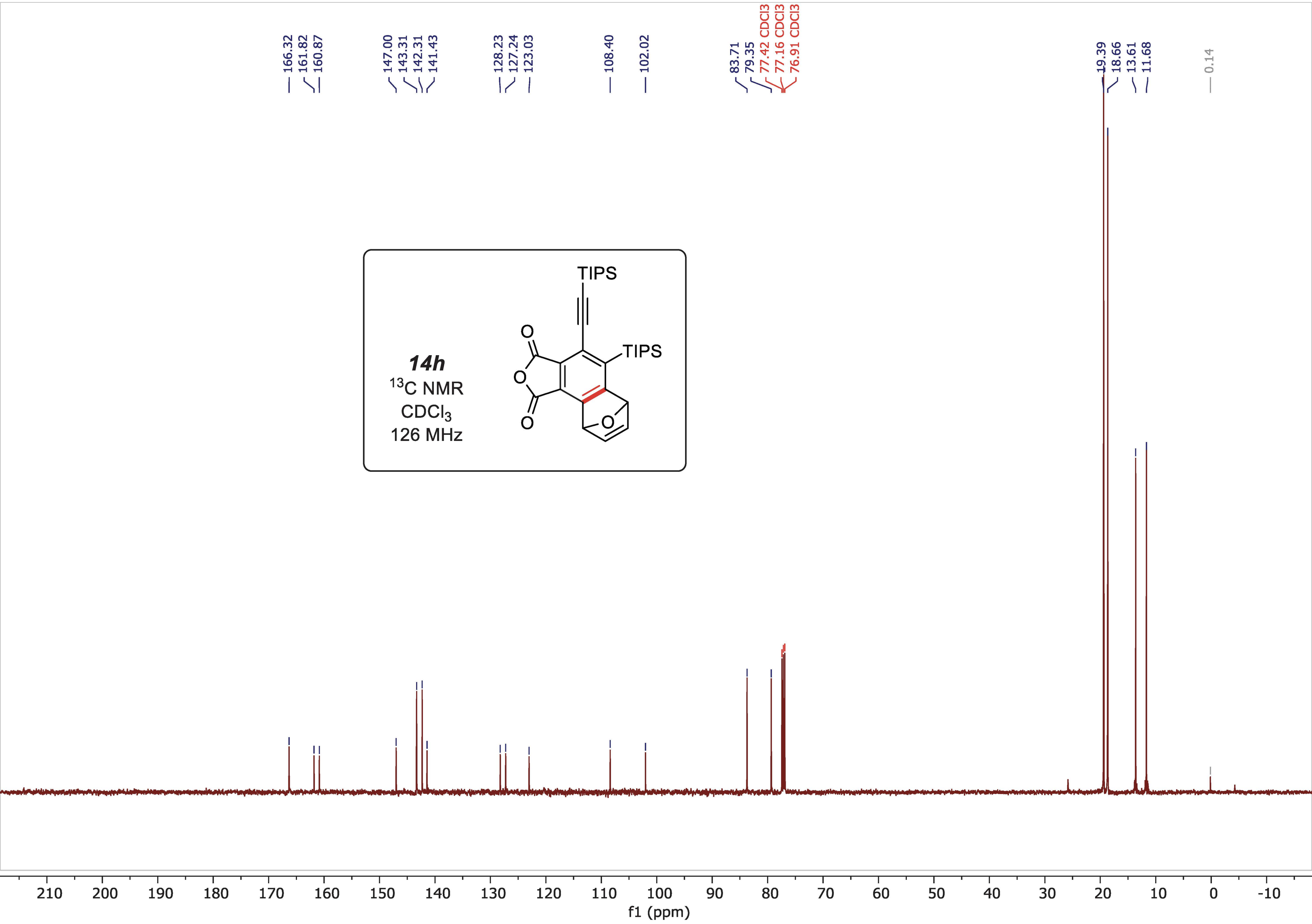

166.32
161.82
160.87
147.00
143.31
142.31
141.43
128.23
127.24
123.03
108.40
102.02
83.71
79.35
77.42 CDCl3
77.16 CDCl3
76.91 CDCl3
19.39
18.66
13.61
11.68
0.14
14h
13C NMR
CDCl3
126 MHz
TIPS
TIPS
O
O
O
O
210 200 190 180 170 160 150 140 130 120 110 100 90 80 70 60 50 40 30 20 10 0 -10
f1 (ppm)

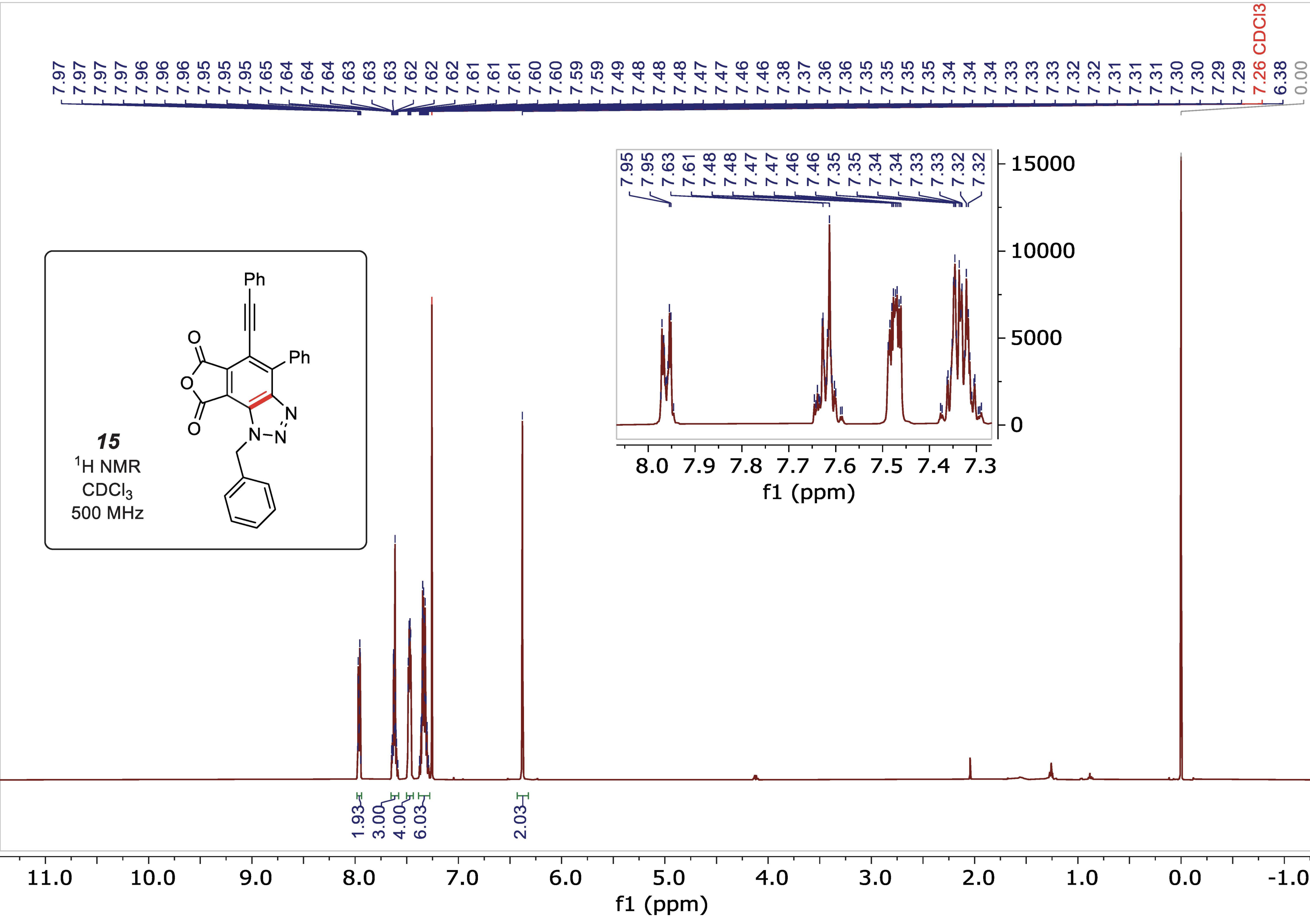
15
1H NMR
CDCl3
500 MHz
Ph
Ph
O
O
O
N
N
N
f1 (ppm)

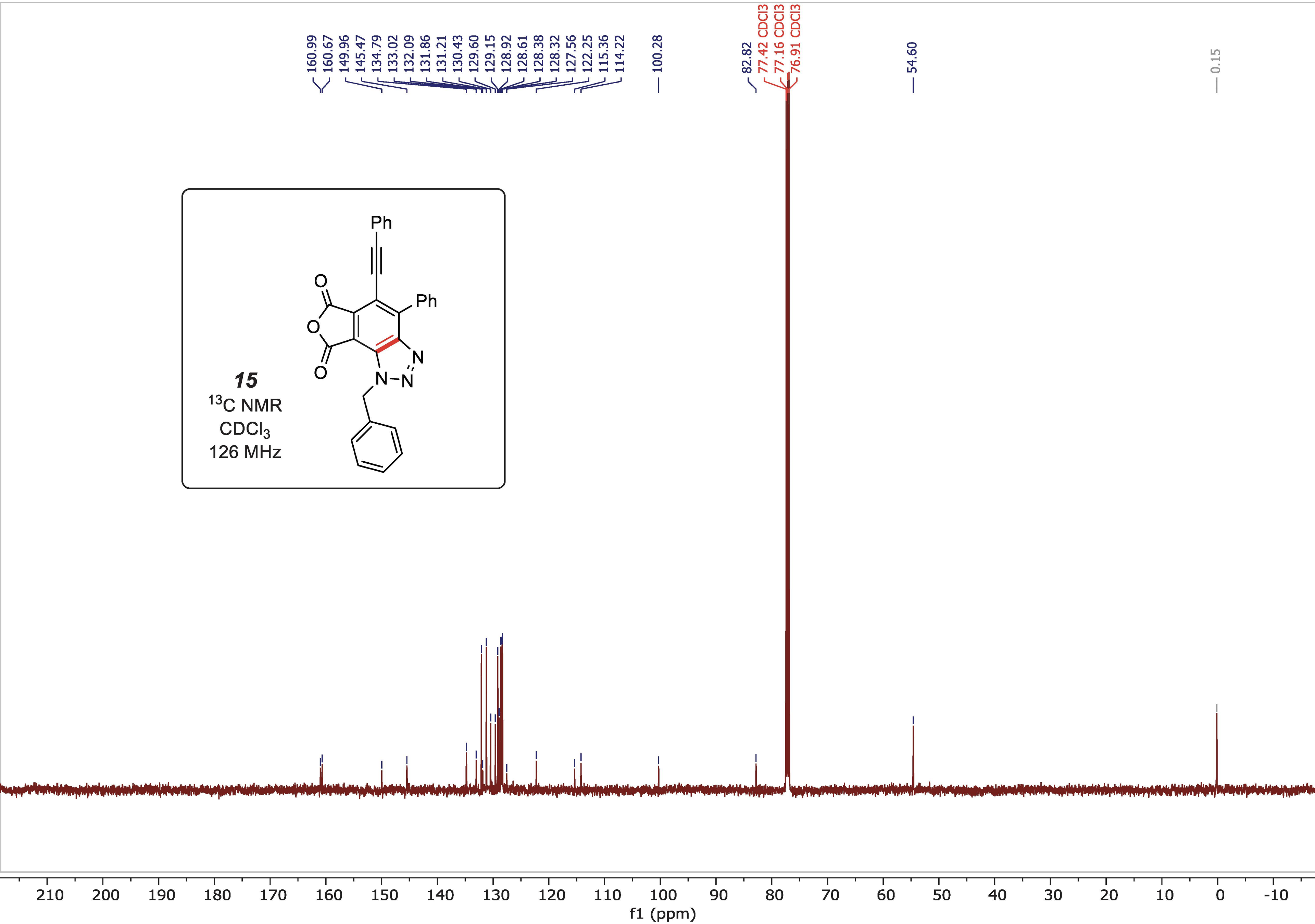
160.99
160.67
149.96
145.47
134.79
133.02
132.09
131.86
131.21
130.43
129.60
129.15
128.92
128.61
128.38
128.32
127.56
122.25
115.36
114.22
100.28
82.82
77.42 CDCl3
77.16 CDCl3
76.91 CDCl3
54.60
0.15
Ph
O
Ph
O
O
N
N
N
15
13C NMR
CDCl3
126 MHz
210 200 190 180 170 160 150 140 130 120 110 100 90 80 70 60 50 40 30 20 10 0 -10
f1 (ppm)

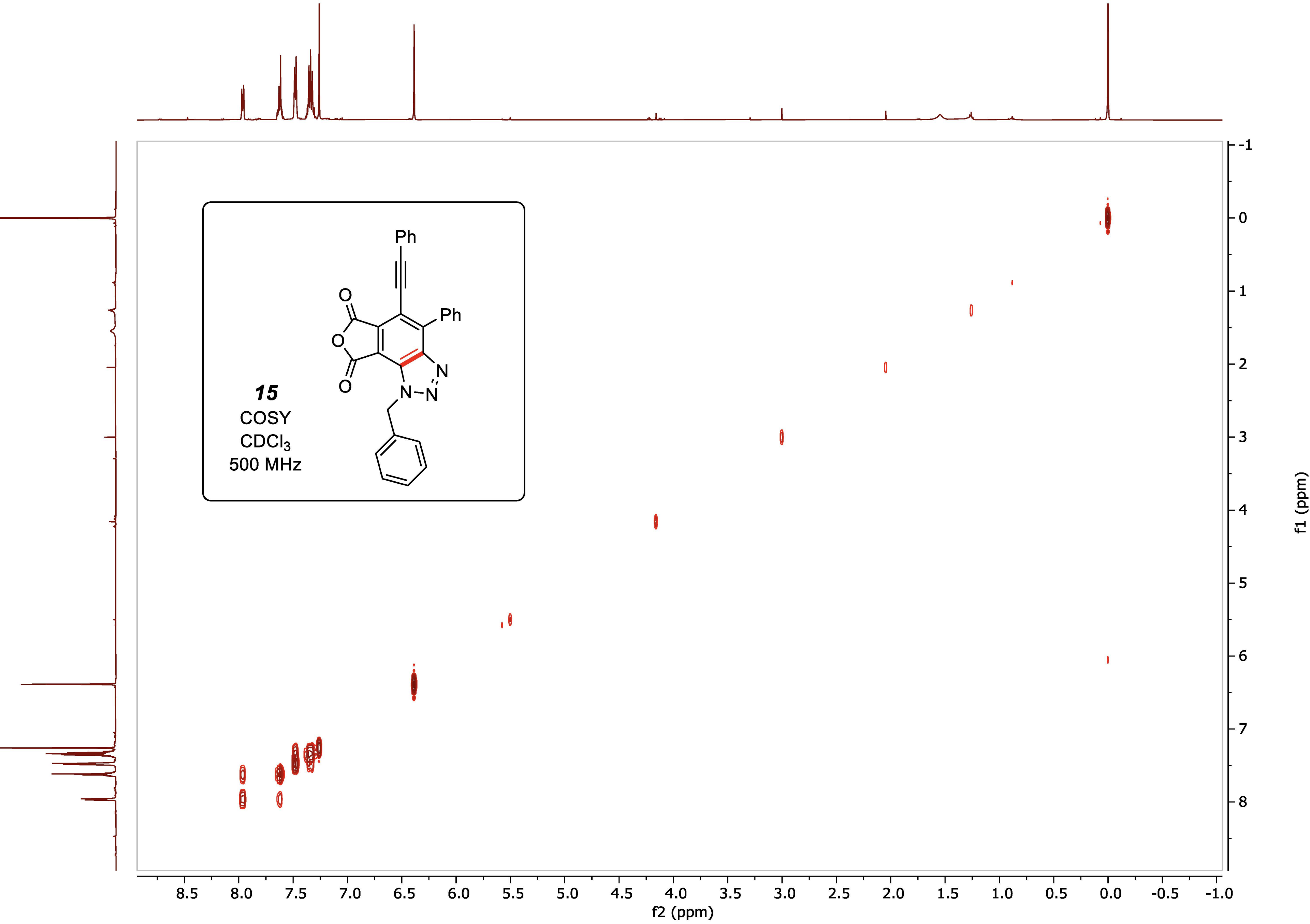
15
COSY
CDCl3
500 MHz
Ph
Ph
O
O
O
N
N
N
f2 (ppm)
f1 (ppm)
8.5 8.0 7.5 7.0 6.5 6.0 5.5 5.0 4.5 4.0 3.5 3.0 2.5 2.0 1.5 1.0 0.5 0.0 -0.5 -1.0
-1 0 1 2 3 4 5 6 7 8

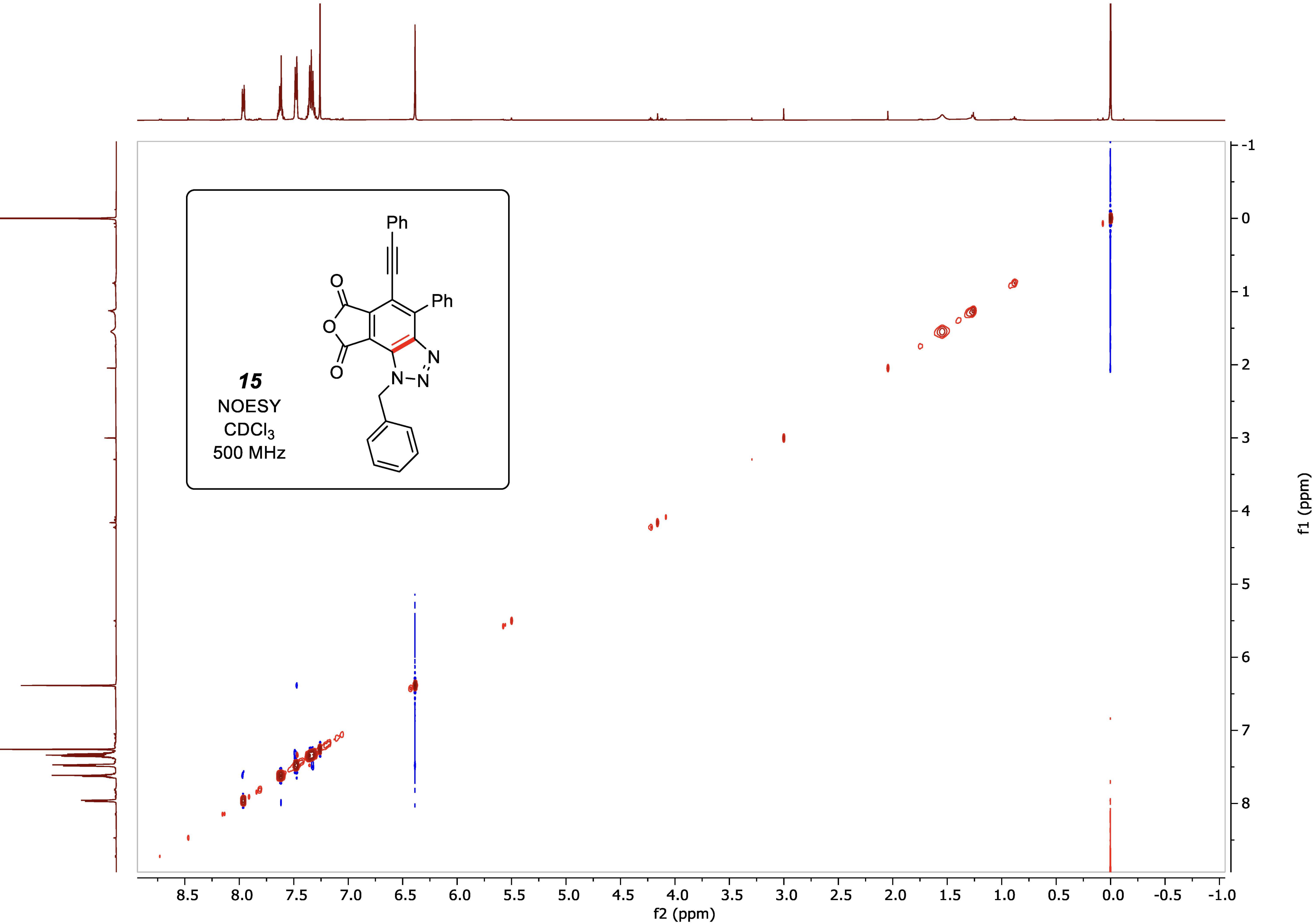
15
NOESY
CDCl3
500 MHz
Ph
Ph
O
O
O
N
N
N
f2 (ppm)
f1 (ppm)

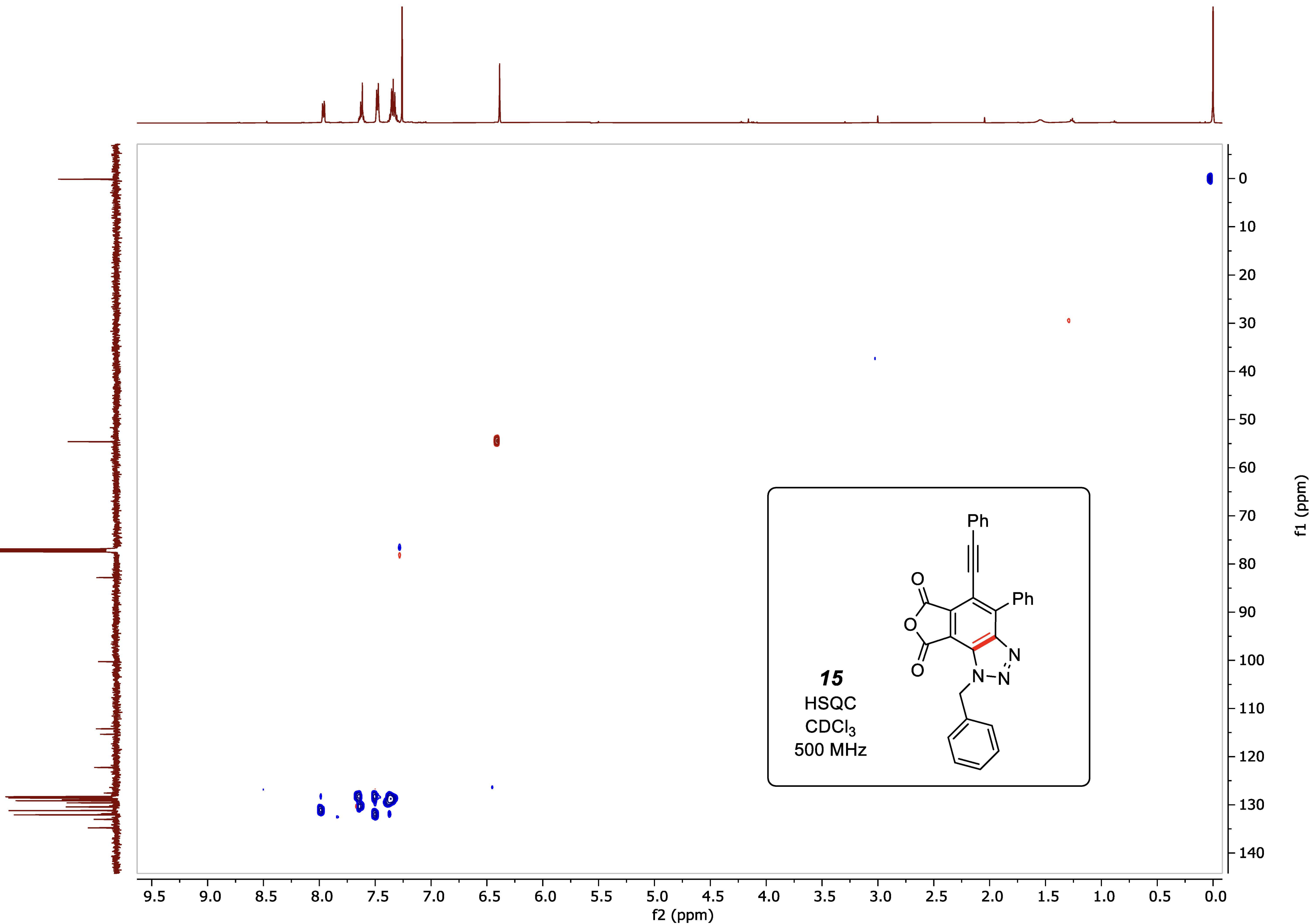
Ph
O
Ph
O
O
N
N
N
15
HSQC
CDCl3
500 MHz
f1 (ppm)
f2 (ppm)

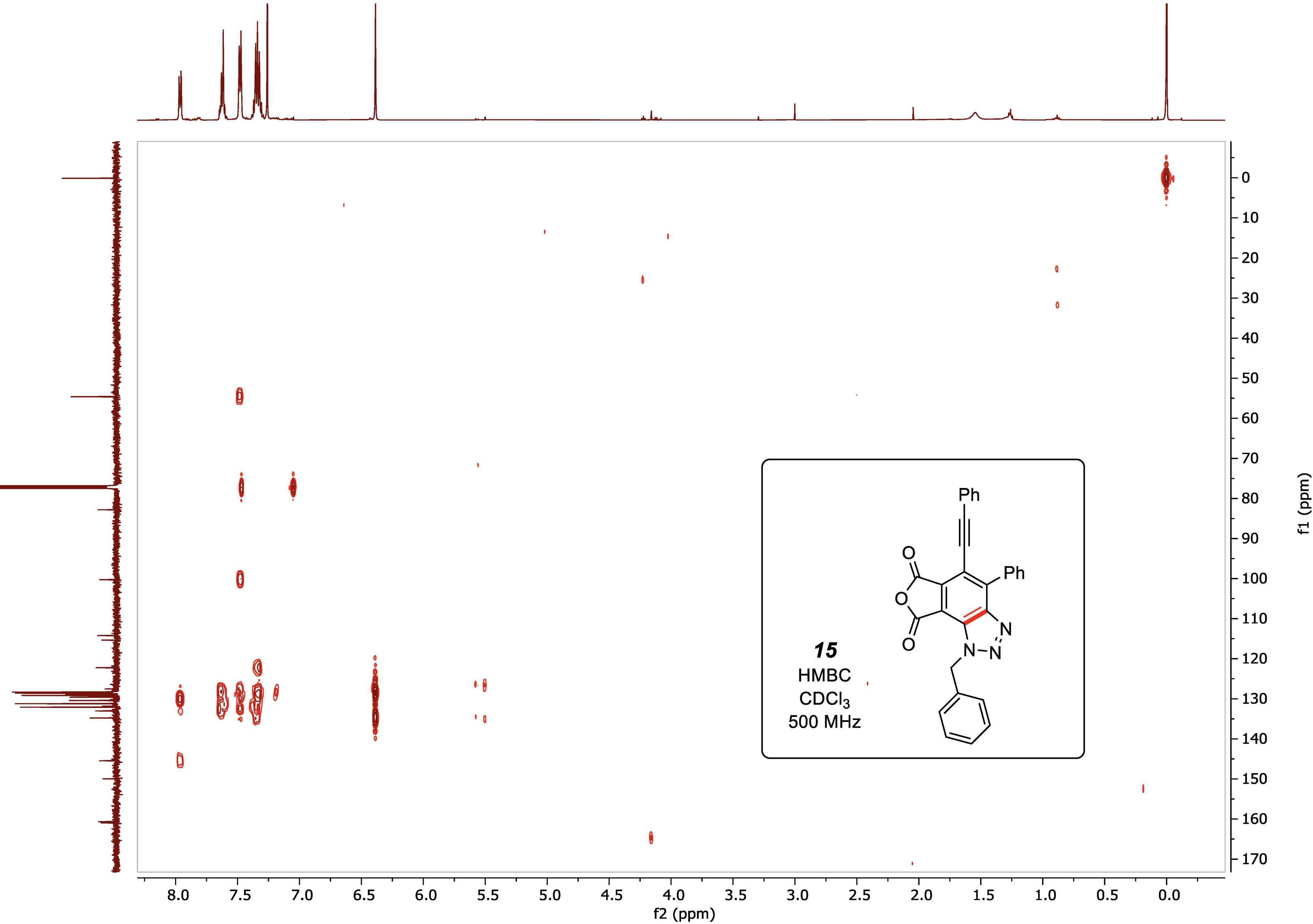
Ph
O
Ph
O
N
N
N
O
15
HMBC
CDCl3
500 MHz
f2 (ppm)
f1 (ppm)

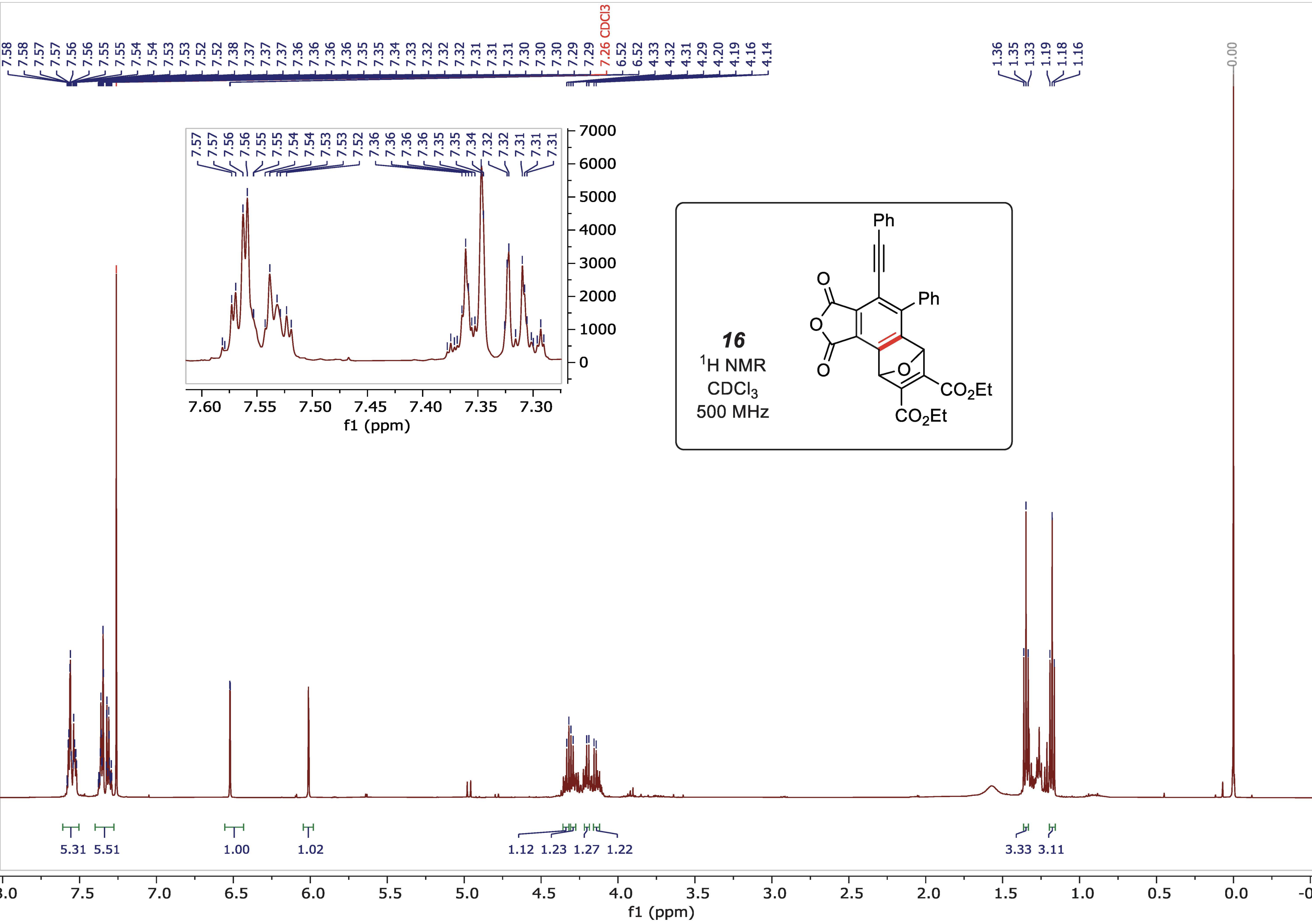
7.58 7.58 7.57 7.57 7.56 7.56 7.55 7.55 7.54 7.54 7.53 7.53 7.52 7.52 7.38 7.37 7.37 7.37 7.36 7.36 7.36 7.35 7.35 7.34 7.33 7.32 7.32 7.32 7.31 7.31 7.31 7.30 7.30 7.30 7.29 7.29
7.26 CDCl3
6.52 6.52 4.33 4.32 4.31 4.29 4.20 4.19 4.16 4.14
1.36 1.35 1.33 1.19 1.18 1.16
0.00
7.57 7.57 7.56 7.56 7.55 7.55 7.54 7.54 7.53 7.53 7.52 7.36 7.36 7.36 7.36 7.35 7.35 7.34 7.32 7.32 7.31 7.31 7.31
7000 6000 5000 4000 3000 2000 1000 0
7.60 7.55 7.50 7.45 7.40 7.35 7.30
f1 (ppm)
16
1H NMR
CDCl3
500 MHz
Ph
Ph
O
O
O
O
CO2Et
CO2Et
5.31 5.51 1.00 1.02 1.12 1.23 1.27 1.22 3.33 3.11
8.0 7.5 7.0 6.5 6.0 5.5 5.0 4.5 4.0 3.5 3.0 2.5 2.0 1.5 1.0 0.5 0.0
f1 (ppm)

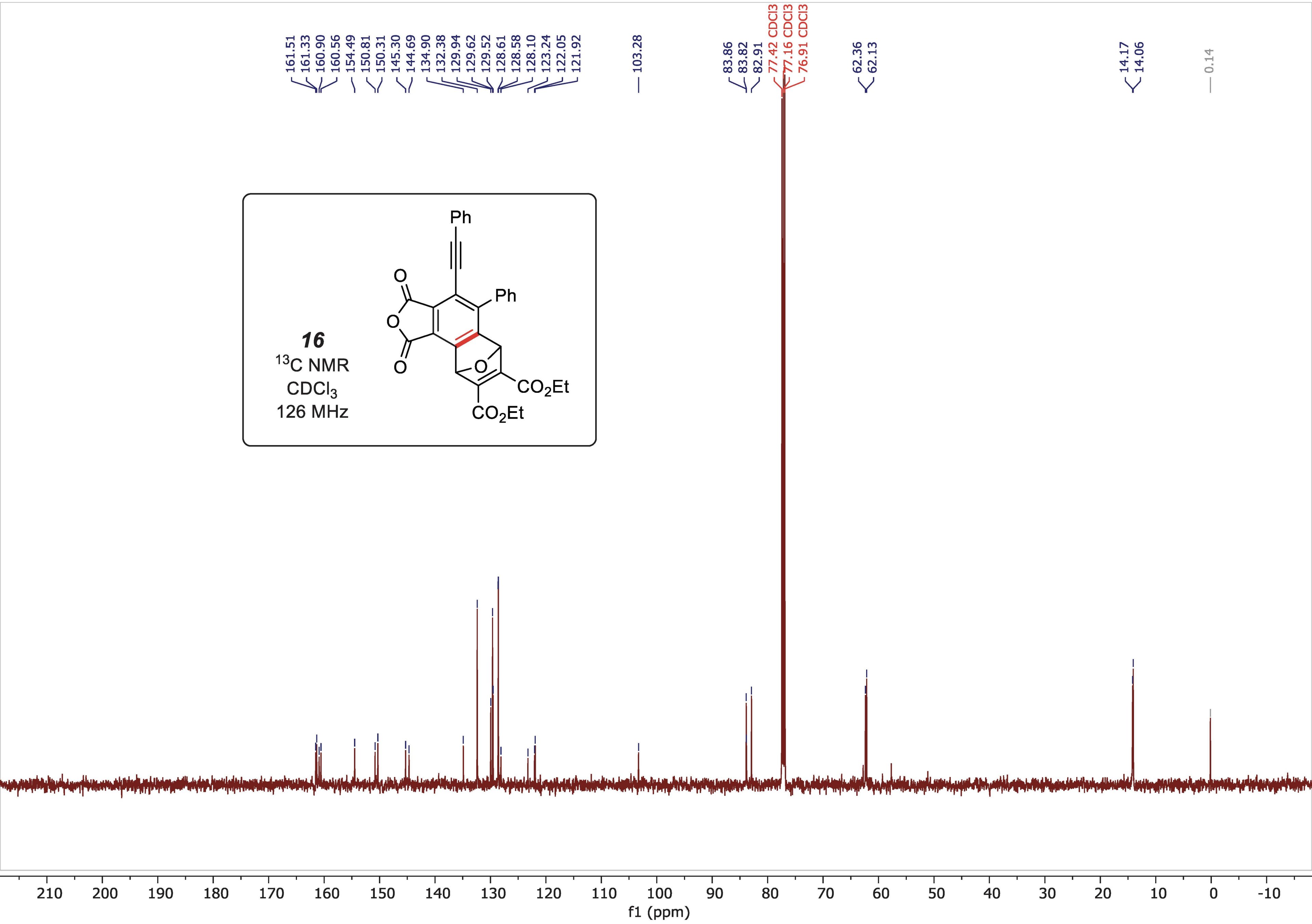

161.51
161.33
160.90
160.56
154.49
150.81
150.31
145.30
144.69
134.90
132.38
129.94
129.62
129.52
128.61
128.58
128.10
123.24
122.05
121.92
103.28
83.86
83.82
82.91
77.42 CDCl3
77.16 CDCl3
76.91 CDCl3
62.36
62.13
14.17
14.06
0.14
16
13C NMR
CDCl3
126 MHz
Ph
O
O
O
Ph
O
CO2Et
CO2Et
210 200 190 180 170 160 150 140 130 120 110 100 90 80 70 60 50 40 30 20 10 0 -10
f1 (ppm)

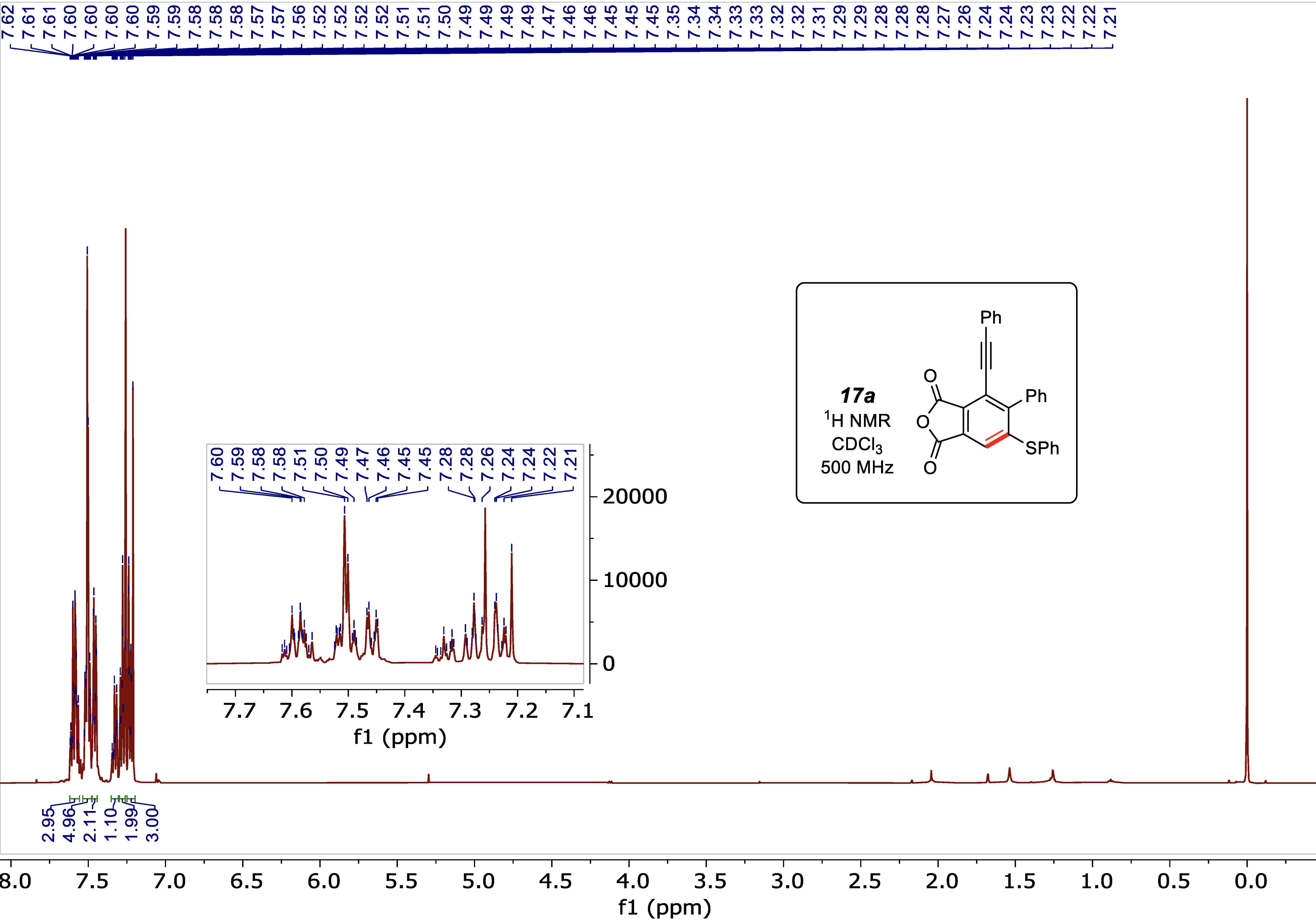

17a
^{1}H NMR
$CDCl_3$
500 MHz
Ph
Ph
SPh
O
O
O
f1 (ppm)

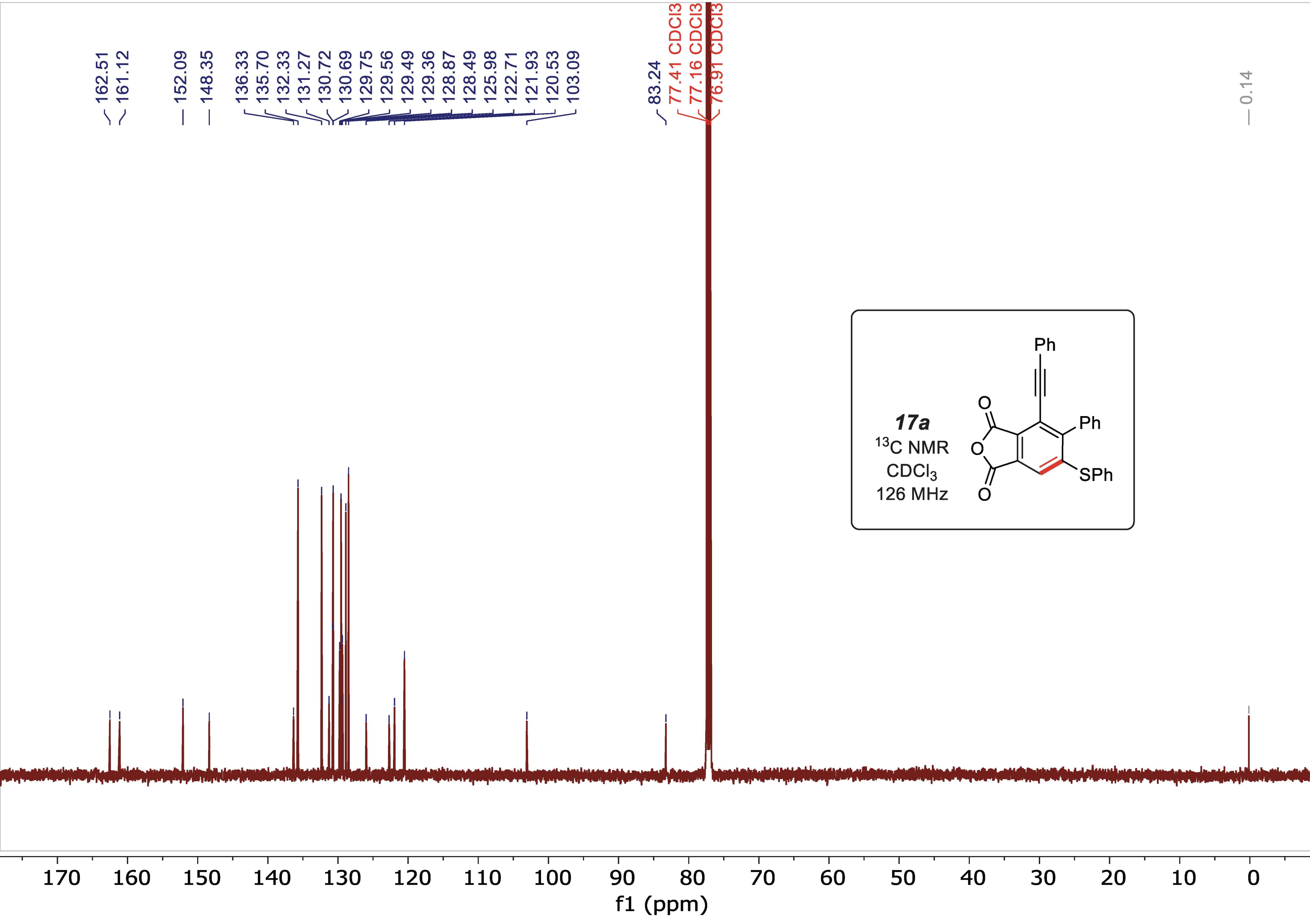
162.51
161.12
152.09
148.35
136.33
135.70
132.33
131.27
130.72
130.69
129.75
129.56
129.49
129.36
128.87
128.49
125.98
122.71
121.93
120.53
103.09
83.24
77.41 CDCl3
77.16 CDCl3
76.91 CDCl3
0.14
17a
^{13}C NMR
CDCl$_3$
126 MHz
Ph
O
Ph
O
SPh
O
170 160 150 140 130 120 110 100 90 80 70 60 50 40 30 20 10 0
f1 (ppm)

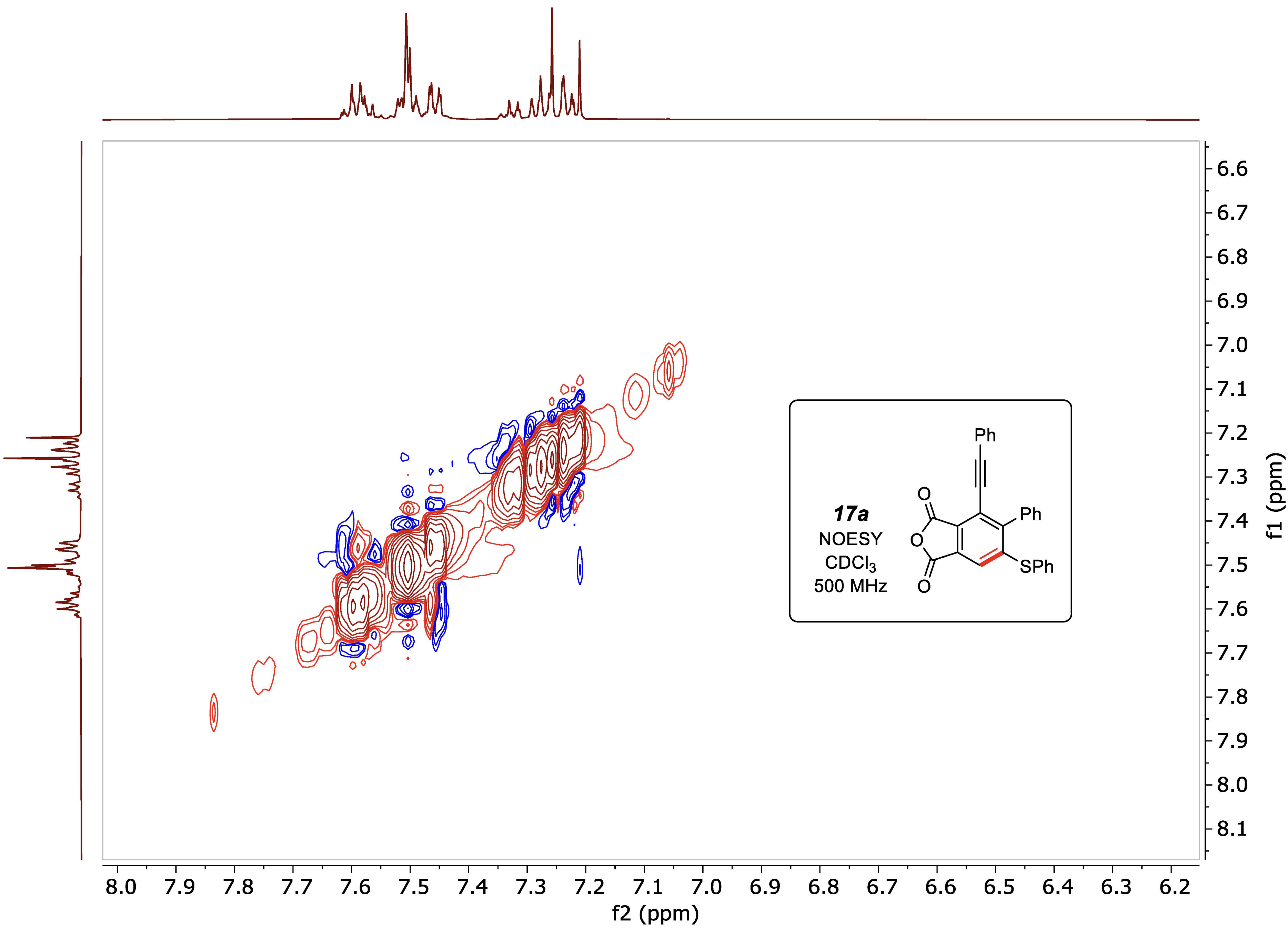

17a
NOESY
CDCl3
500 MHz
Ph
O
Ph
O
SPh
O
f1 (ppm)
f2 (ppm)

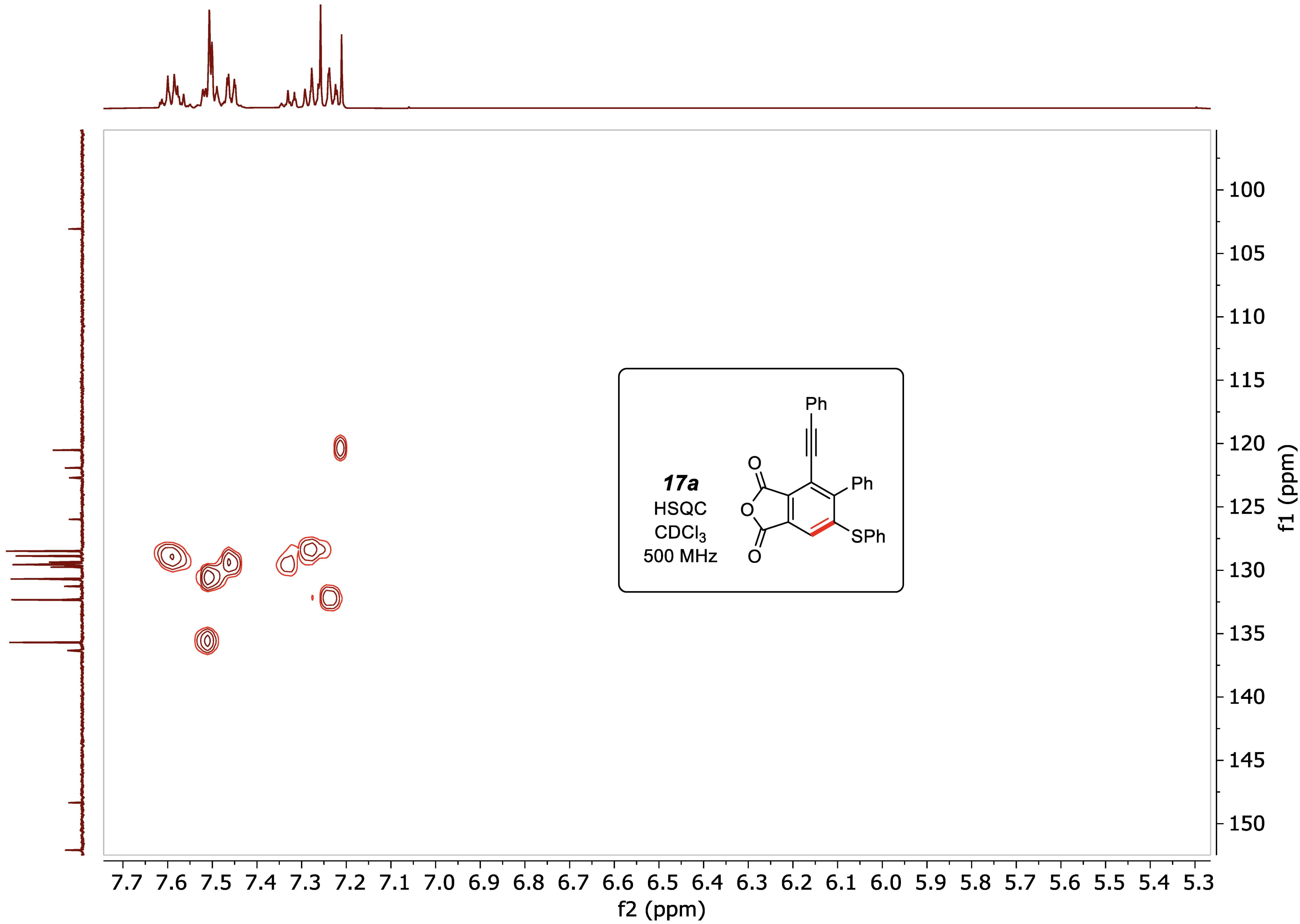
17a
HSQC
$CDCl_3$
500 MHz
Ph
O
O
O
Ph
SPh
f1 (ppm)
f2 (ppm)
100
105
110
115
120
125
130
135
140
145
150
7.7 7.6 7.5 7.4 7.3 7.2 7.1 7.0 6.9 6.8 6.7 6.6 6.5 6.4 6.3 6.2 6.1 6.0 5.9 5.8 5.7 5.6 5.5 5.4 5.3

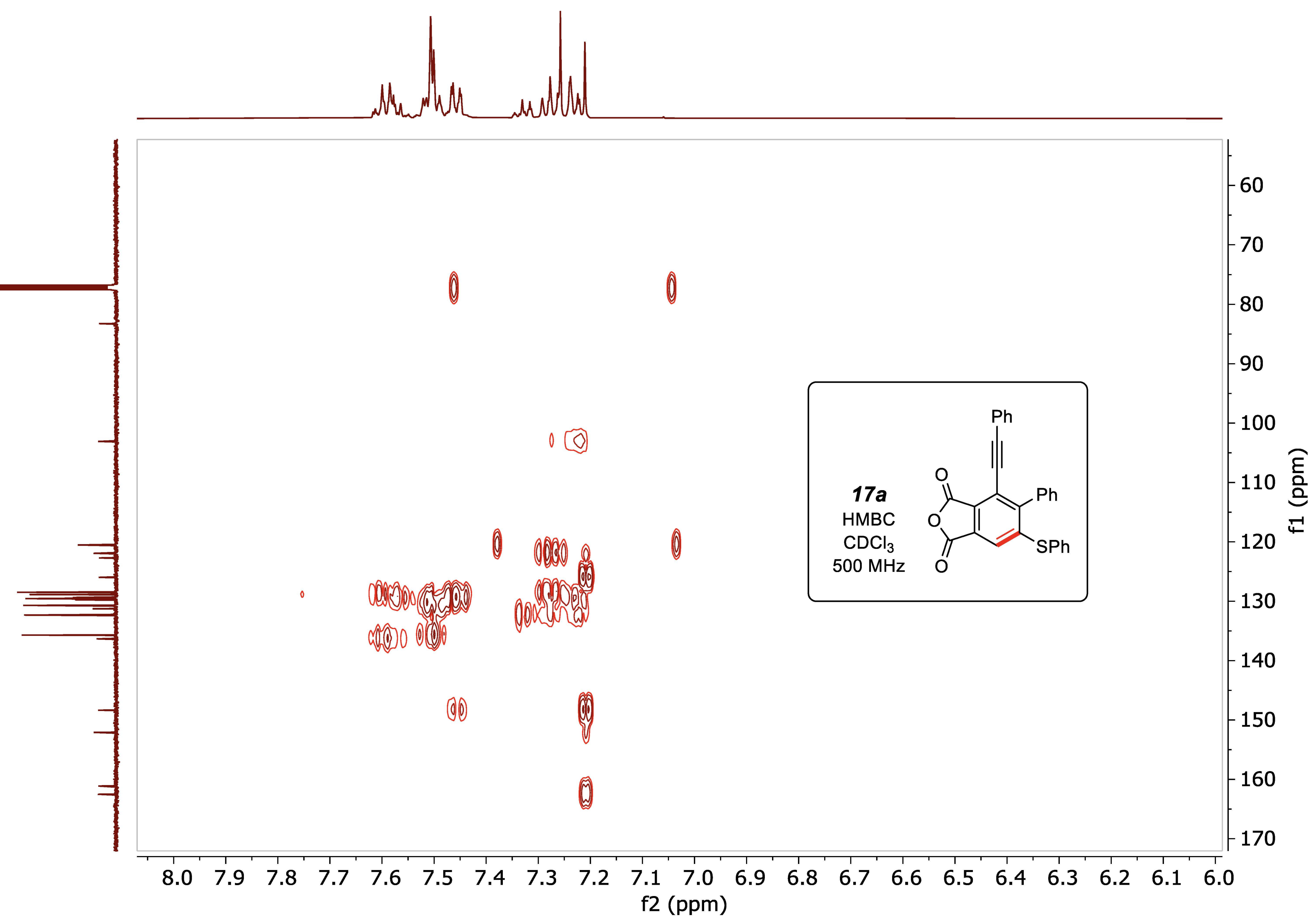
17a
HMBC
$CDCl_3$
500 MHz
Ph
O
O
O
Ph
SPh
f1 (ppm)
f2 (ppm)
60
70
80
90
100
110
120
130
140
150
160
170
8.0
7.9
7.8
7.7
7.6
7.5
7.4
7.3
7.2
7.1
7.0
6.9
6.8
6.7
6.6
6.5
6.4
6.3
6.2
6.1
6.0

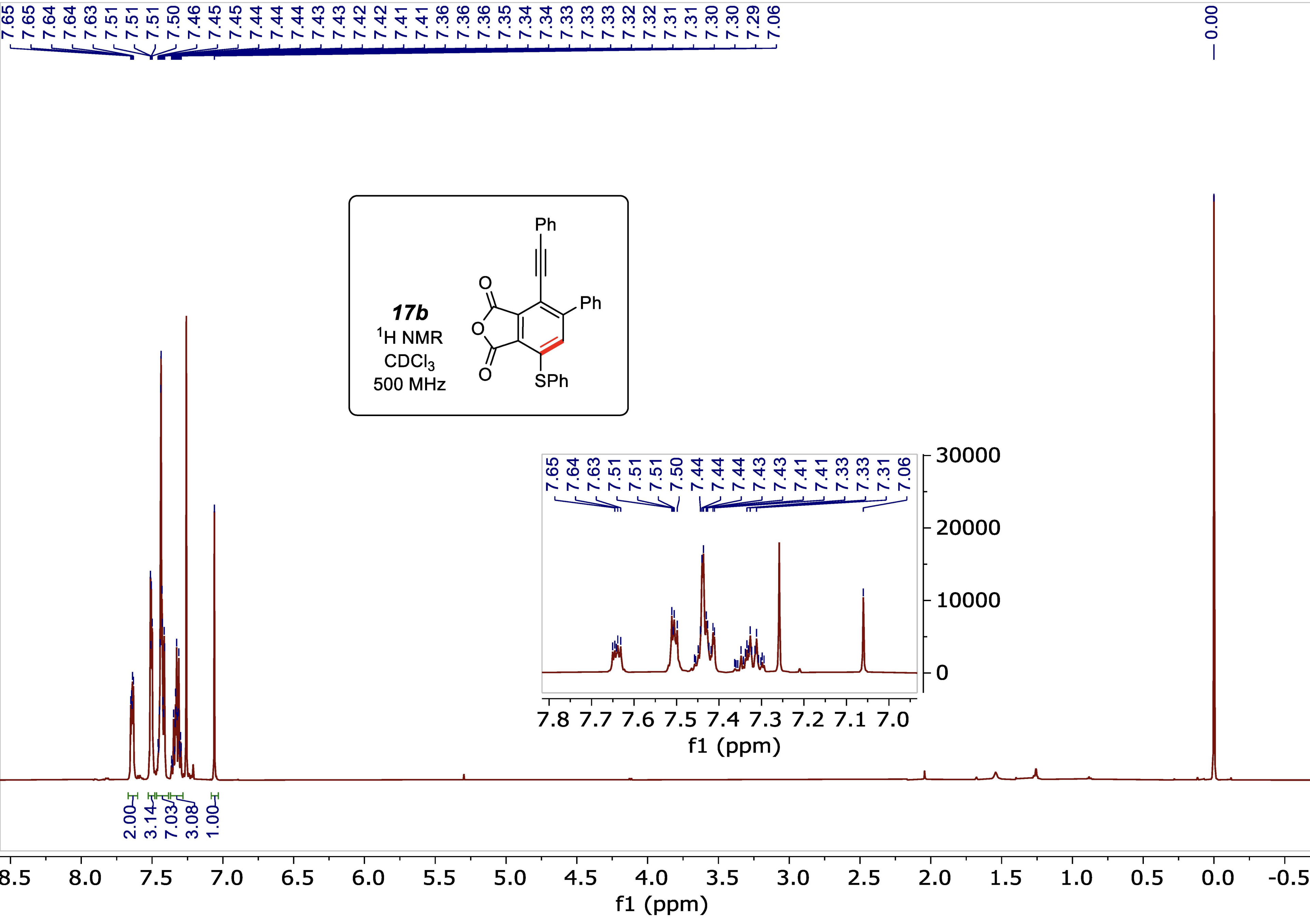
17b
1H NMR
CDCl3
500 MHz
Ph
Ph
SPh
O
O
O
f1 (ppm)

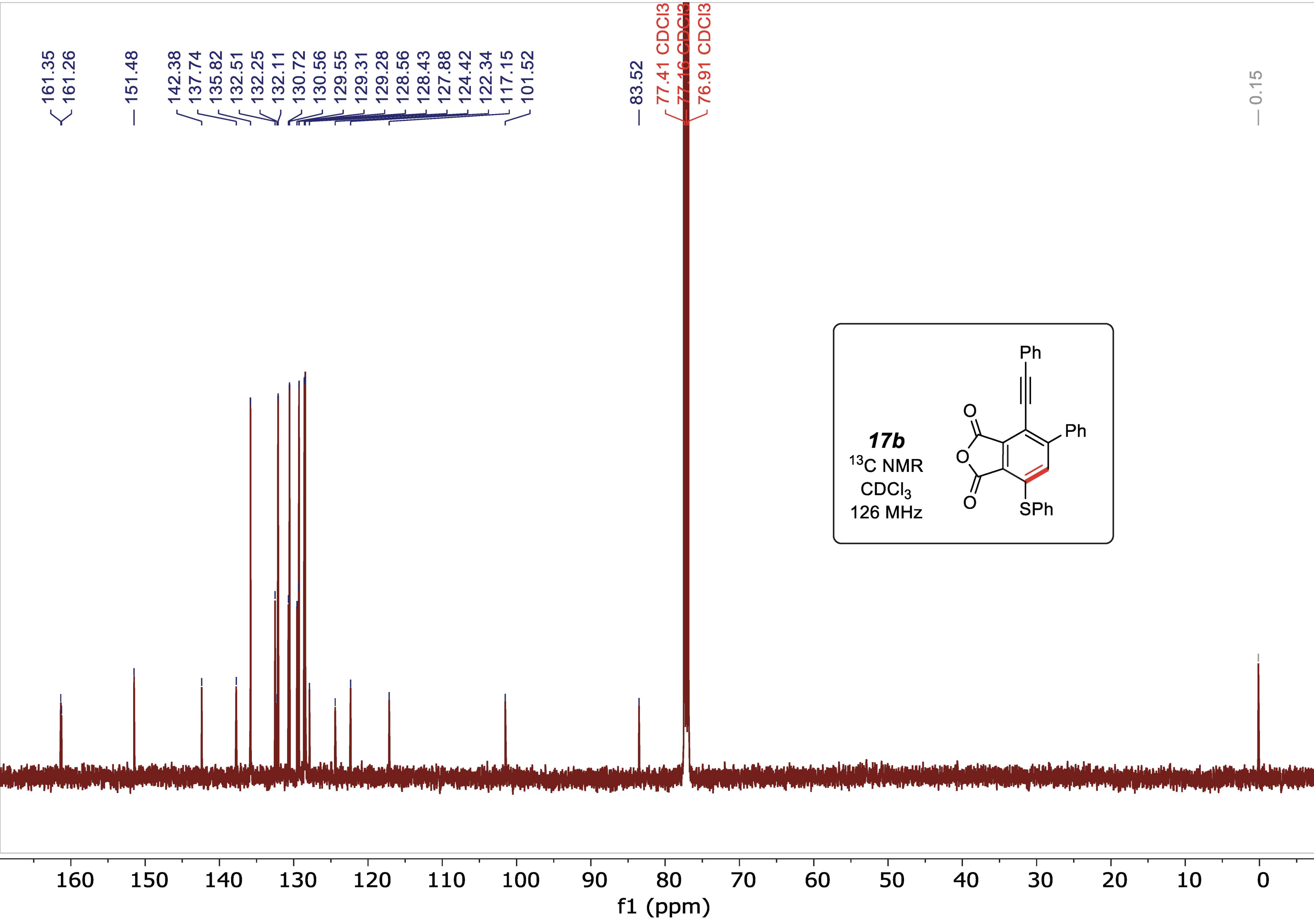
161.35
161.26
151.48
142.38
137.74
135.82
132.51
132.25
132.11
130.72
130.56
129.55
129.31
129.28
128.56
128.43
127.88
124.42
122.34
117.15
101.52
83.52
77.41 CDCl3
77.16 CDCl3
76.91 CDCl3
0.15
17b
^{13}C NMR
$CDCl_3$
126 MHz
Ph
O
O
O
Ph
SPh
160 150 140 130 120 110 100 90 80 70 60 50 40 30 20 10 0
f1 (ppm)

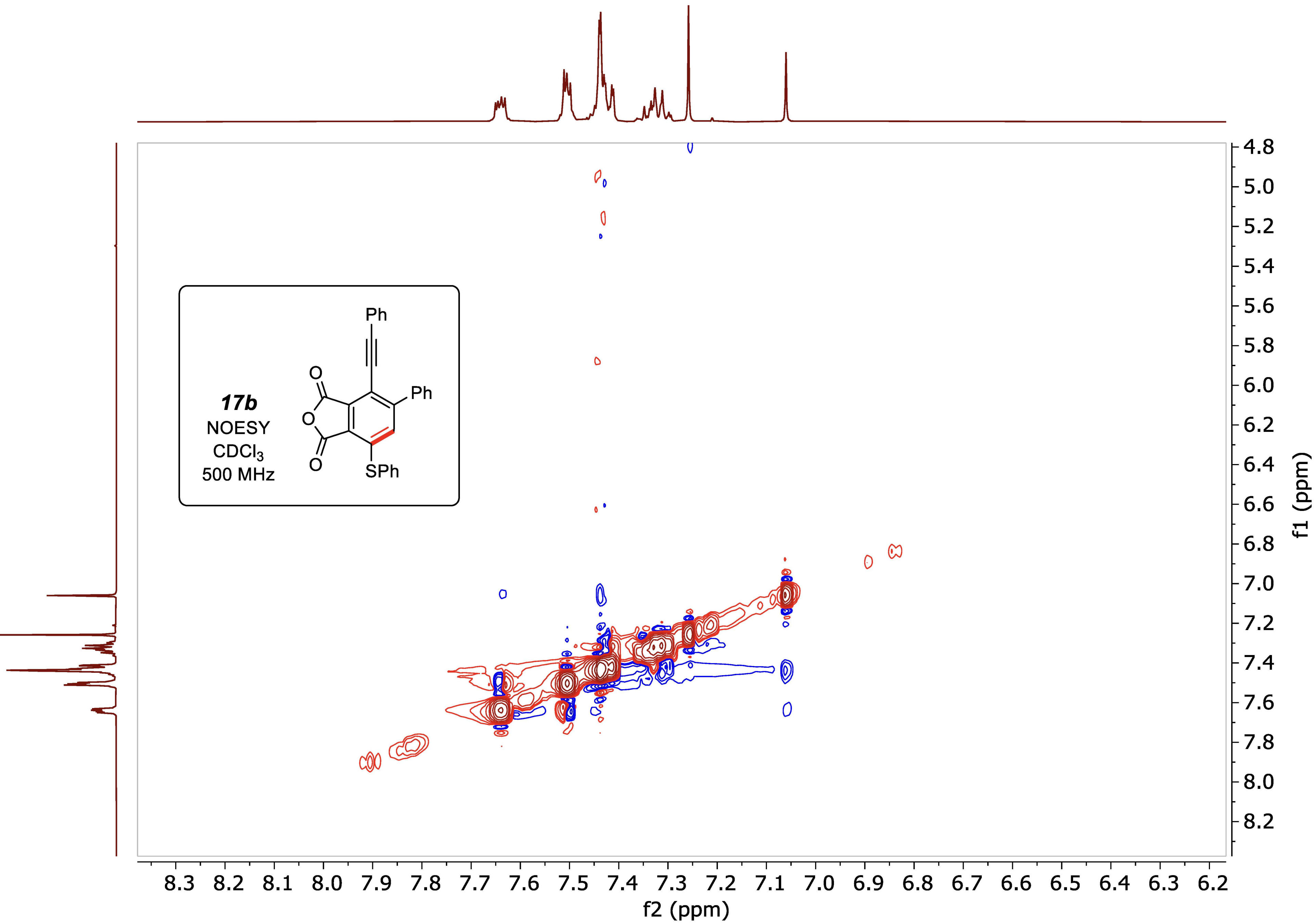
17b
NOESY
CDCl3
500 MHz
Ph
Ph
O
O
O
SPh
f1 (ppm)
f2 (ppm)
4.8
5.0
5.2
5.4
5.6
5.8
6.0
6.2
6.4
6.6
6.8
7.0
7.2
7.4
7.6
7.8
8.0
8.2
8.3 8.2 8.1 8.0 7.9 7.8 7.7 7.6 7.5 7.4 7.3 7.2 7.1 7.0 6.9 6.8 6.7 6.6 6.5 6.4 6.3 6.2

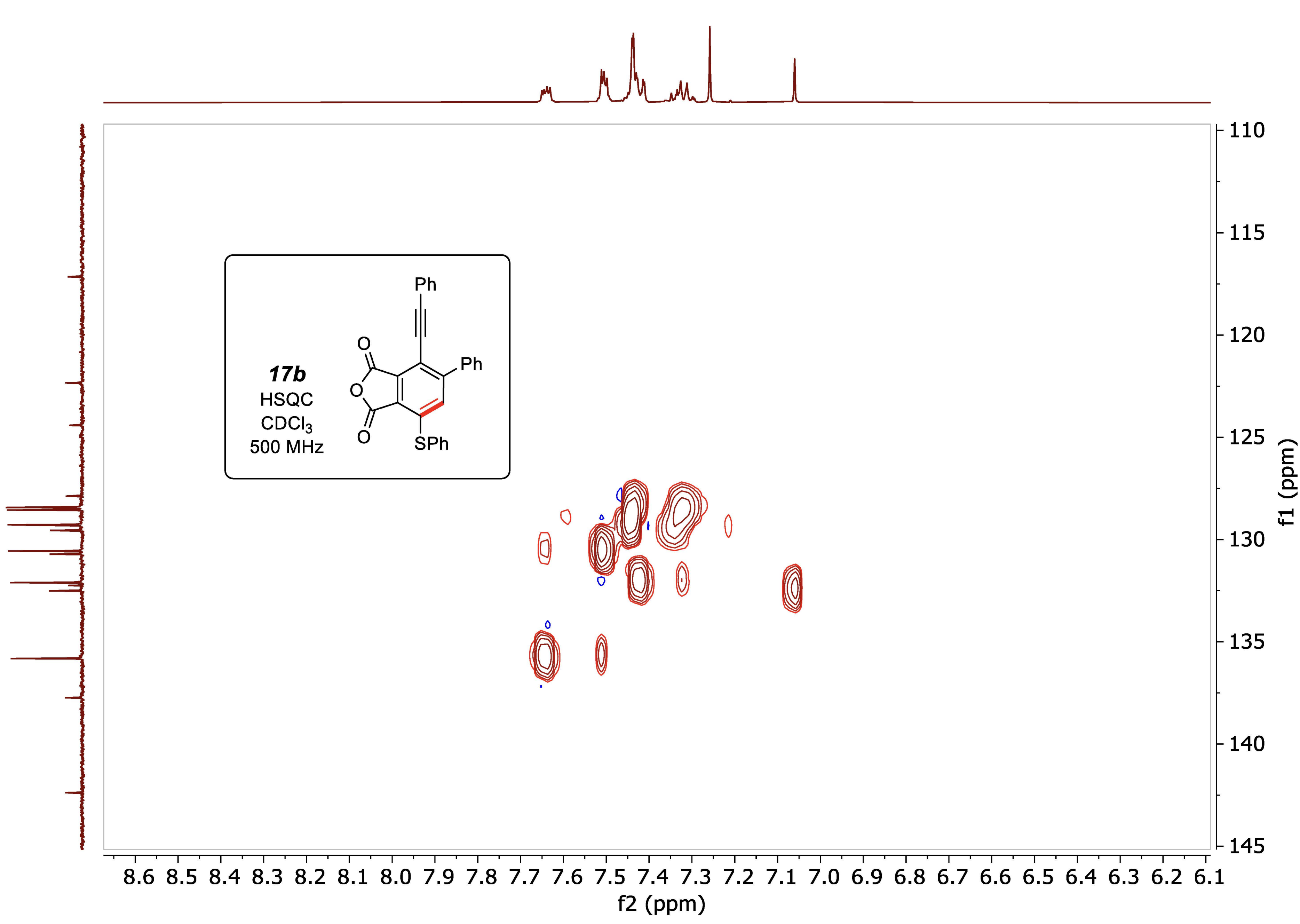
17b
HSQC
CDCl3
500 MHz
Ph
Ph
O
O
O
SPh
f2 (ppm)
f1 (ppm)
8.6 8.5 8.4 8.3 8.2 8.1 8.0 7.9 7.8 7.7 7.6 7.5 7.4 7.3 7.2 7.1 7.0 6.9 6.8 6.7 6.6 6.5 6.4 6.3 6.2 6.1
110 115 120 125 130 135 140 145

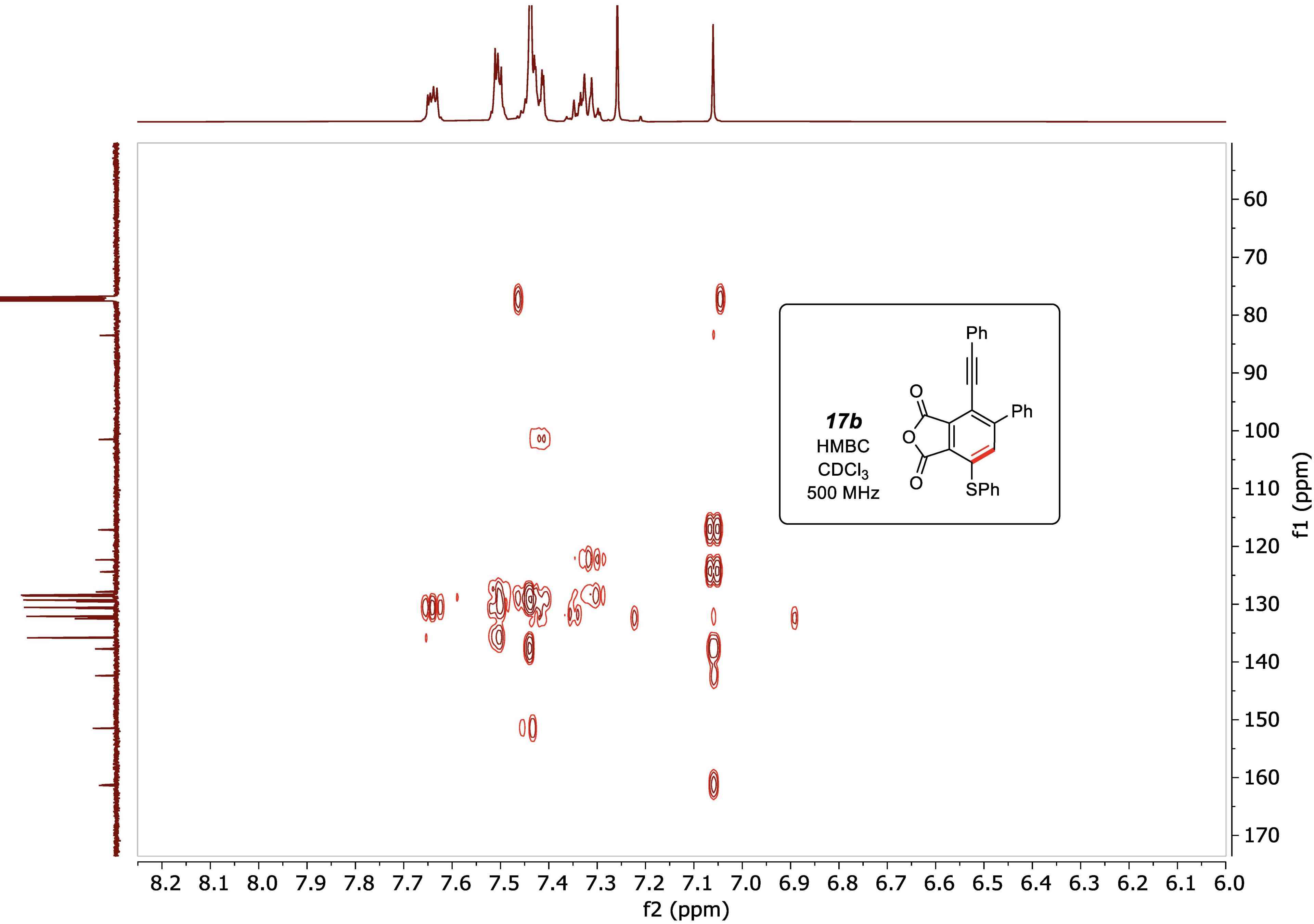
17b
HMBC
$CDCl_3$
500 MHz
Ph
Ph
SPh
O
O
O
f2 (ppm)
f1 (ppm)
8.2 8.1 8.0 7.9 7.8 7.7 7.6 7.5 7.4 7.3 7.2 7.1 7.0 6.9 6.8 6.7 6.6 6.5 6.4 6.3 6.2 6.1 6.0
60 70 80 90 100 110 120 130 140 150 160 170

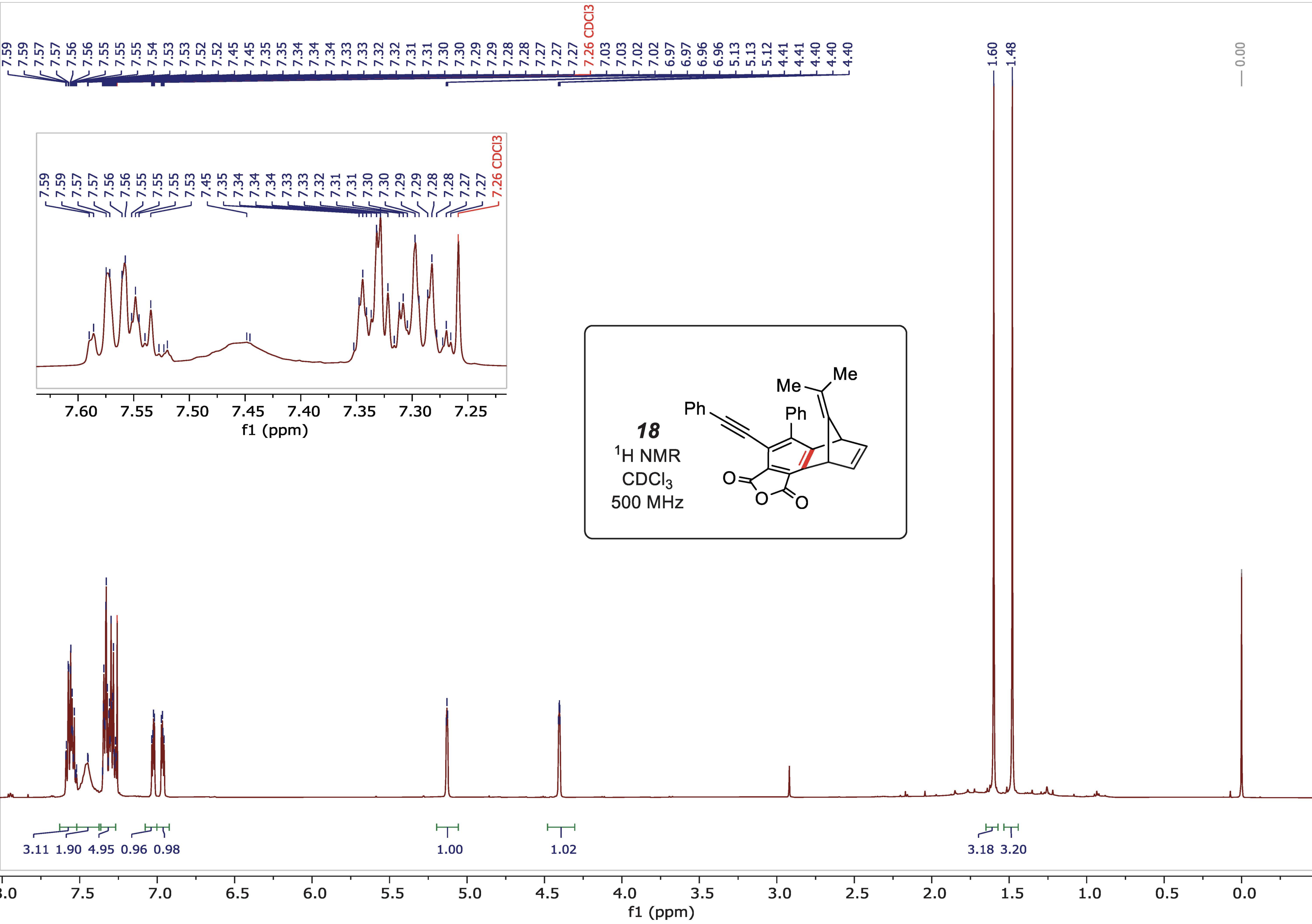
18
^{1}H NMR
$CDCl_3$
500 MHz
Ph
Ph
Me
Me
O
O
O
7.26 CDCl3
3.11 1.90 4.95 0.96 0.98
1.00
1.02
3.18 3.20
f1 (ppm)

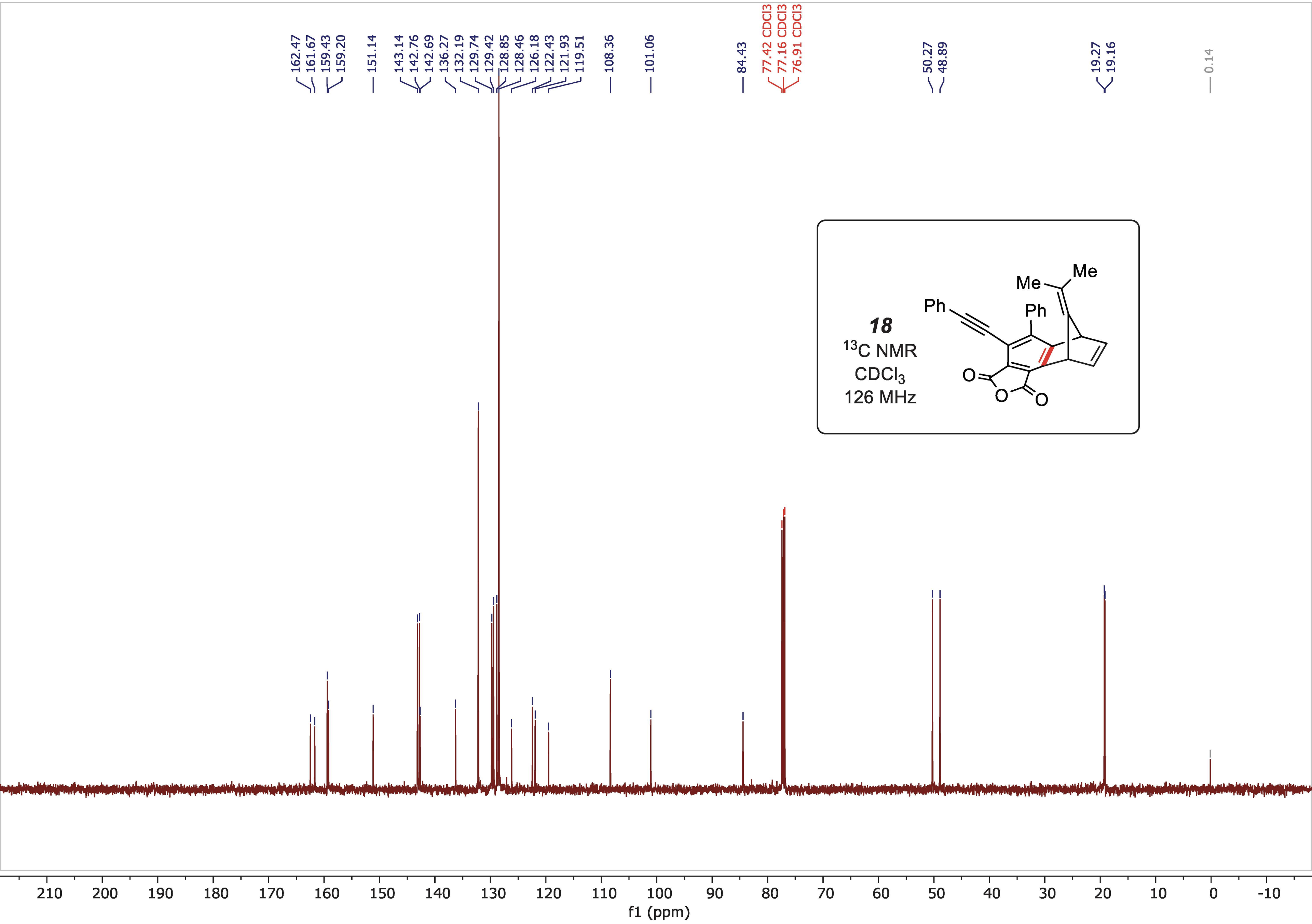
162.47
161.67
159.43
159.20
151.14
143.14
142.76
142.69
136.27
132.19
129.74
129.42
128.85
128.46
126.18
122.43
121.93
119.51
108.36
101.06
84.43
77.42 CDCl3
77.16 CDCl3
76.91 CDCl3
50.27
48.89
19.27
19.16
0.14
18
13C NMR
CDCl3
126 MHz
Ph
Ph
Me
Me
O
O
O
210 200 190 180 170 160 150 140 130 120 110 100 90 80 70 60 50 40 30 20 10 0 -10
f1 (ppm)

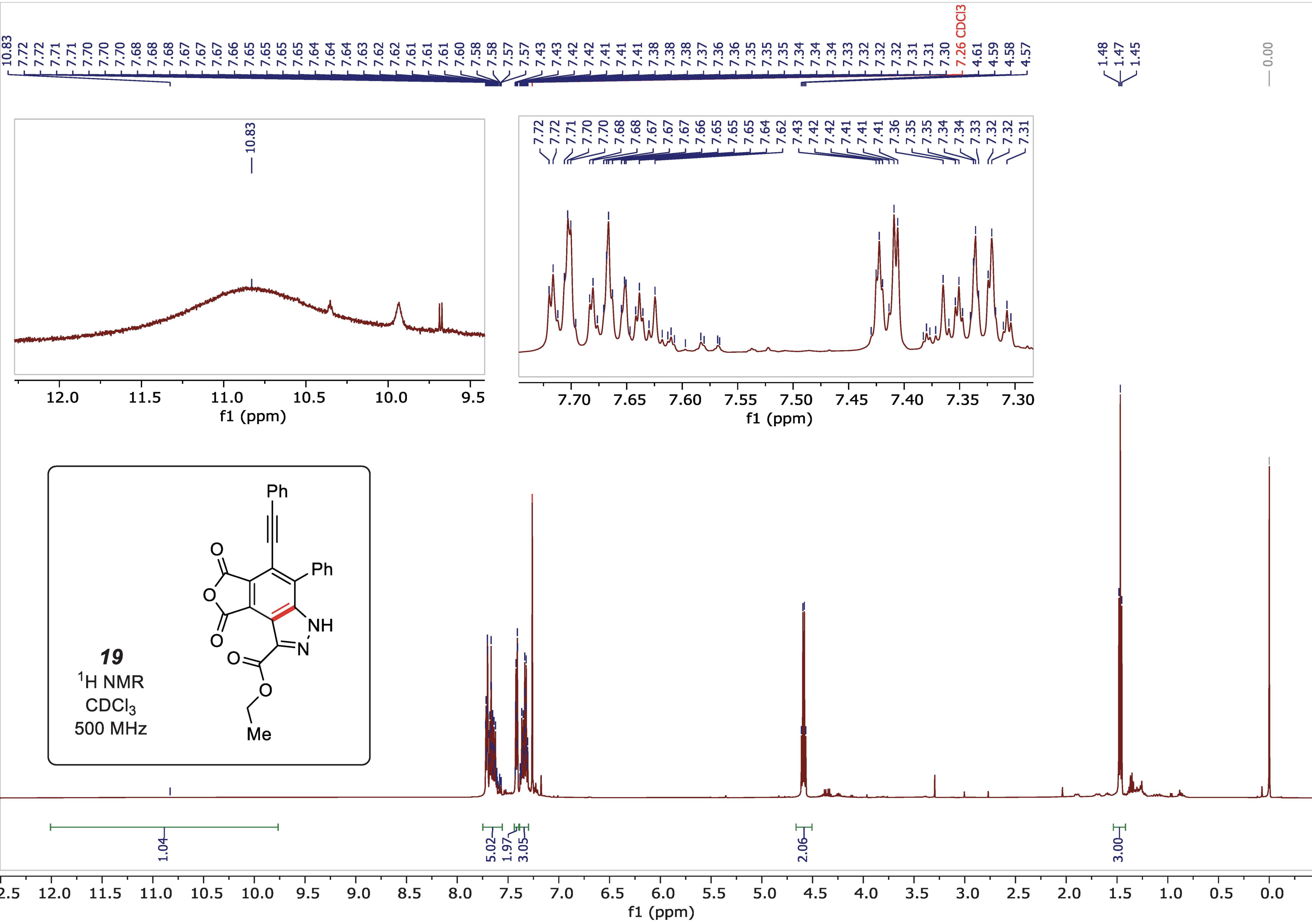
19
^{1}H NMR
$CDCl_3$
500 MHz
Ph
Ph
NH
N
O
O
O
O
O
Me
7.26 CDCl3
f1 (ppm)
1.04
5.02
1.97
3.05
2.06
3.00

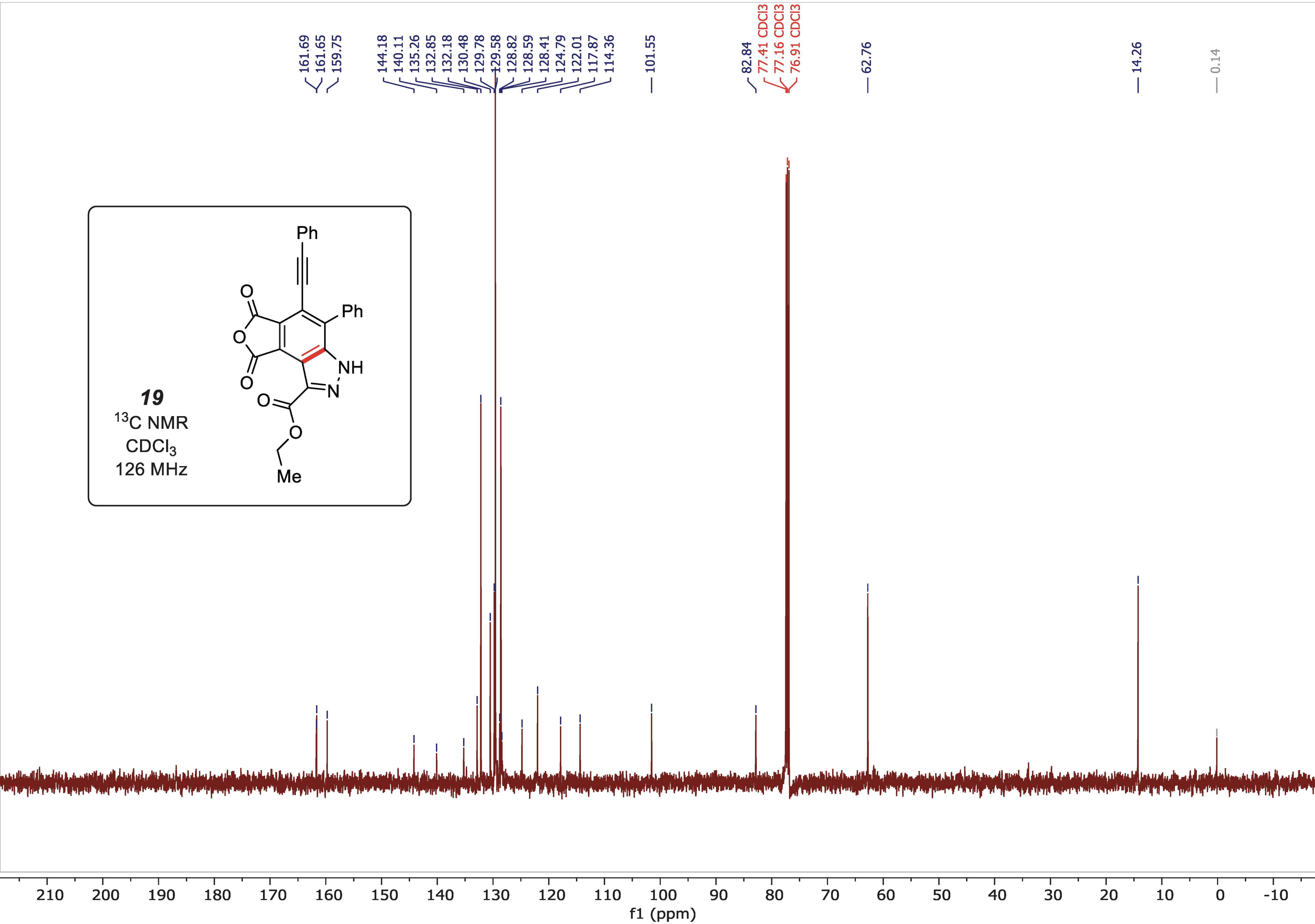
161.69
161.65
159.75
144.18
140.11
135.26
132.85
132.18
130.48
129.78
129.58
128.82
128.59
128.41
124.79
122.01
117.87
114.36
101.55
82.84
77.41 CDCl3
77.16 CDCl3
76.91 CDCl3
62.76
14.26
0.14
Ph
O
Ph
O
NH
O
N
O
O
Me
19
13C NMR
CDCl3
126 MHz
210 200 190 180 170 160 150 140 130 120 110 100 90 80 70 60 50 40 30 20 10 0 -10
f1 (ppm)

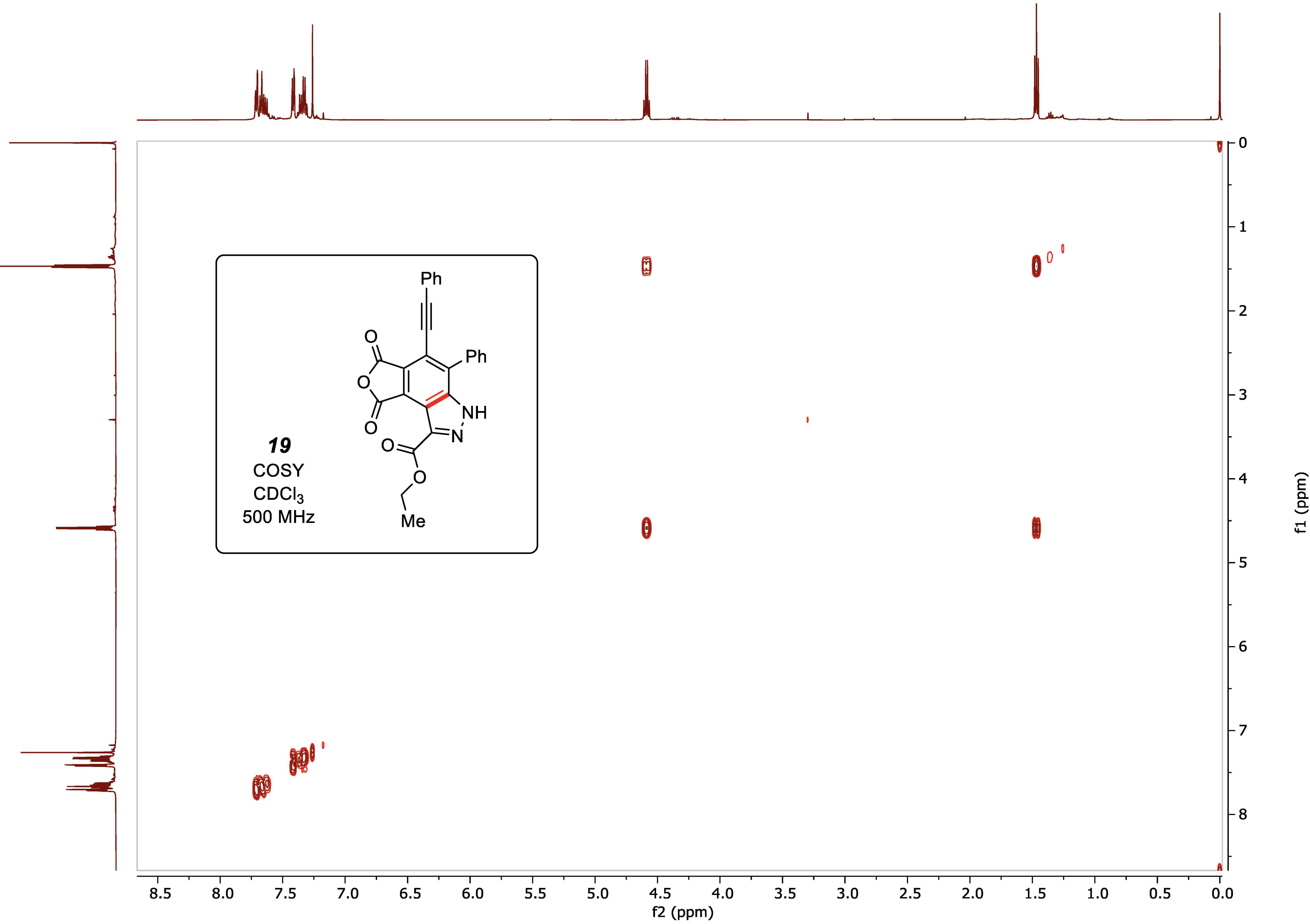
Ph
O
Ph
O
NH
N
O
O
O
Me
19
COSY
CDCl3
500 MHz
f2 (ppm)
f1 (ppm)

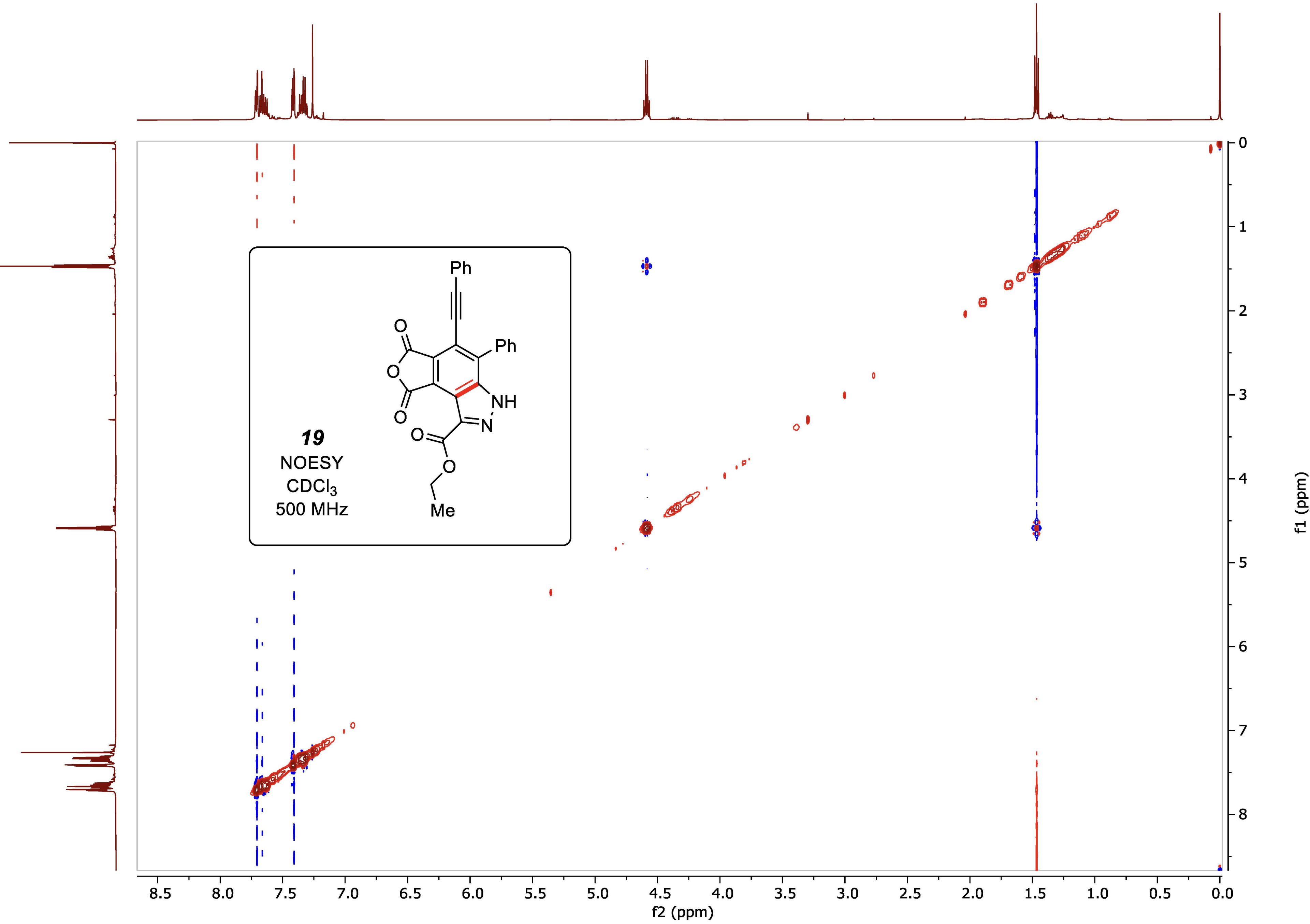
19
NOESY
CDCl3
500 MHz
Ph
Ph
O
O
O
NH
N
O
O
Me
f2 (ppm)
f1 (ppm)
8.5
8.0
7.5
7.0
6.5
6.0
5.5
5.0
4.5
4.0
3.5
3.0
2.5
2.0
1.5
1.0
0.5
0.0
0
1
2
3
4
5
6
7
8

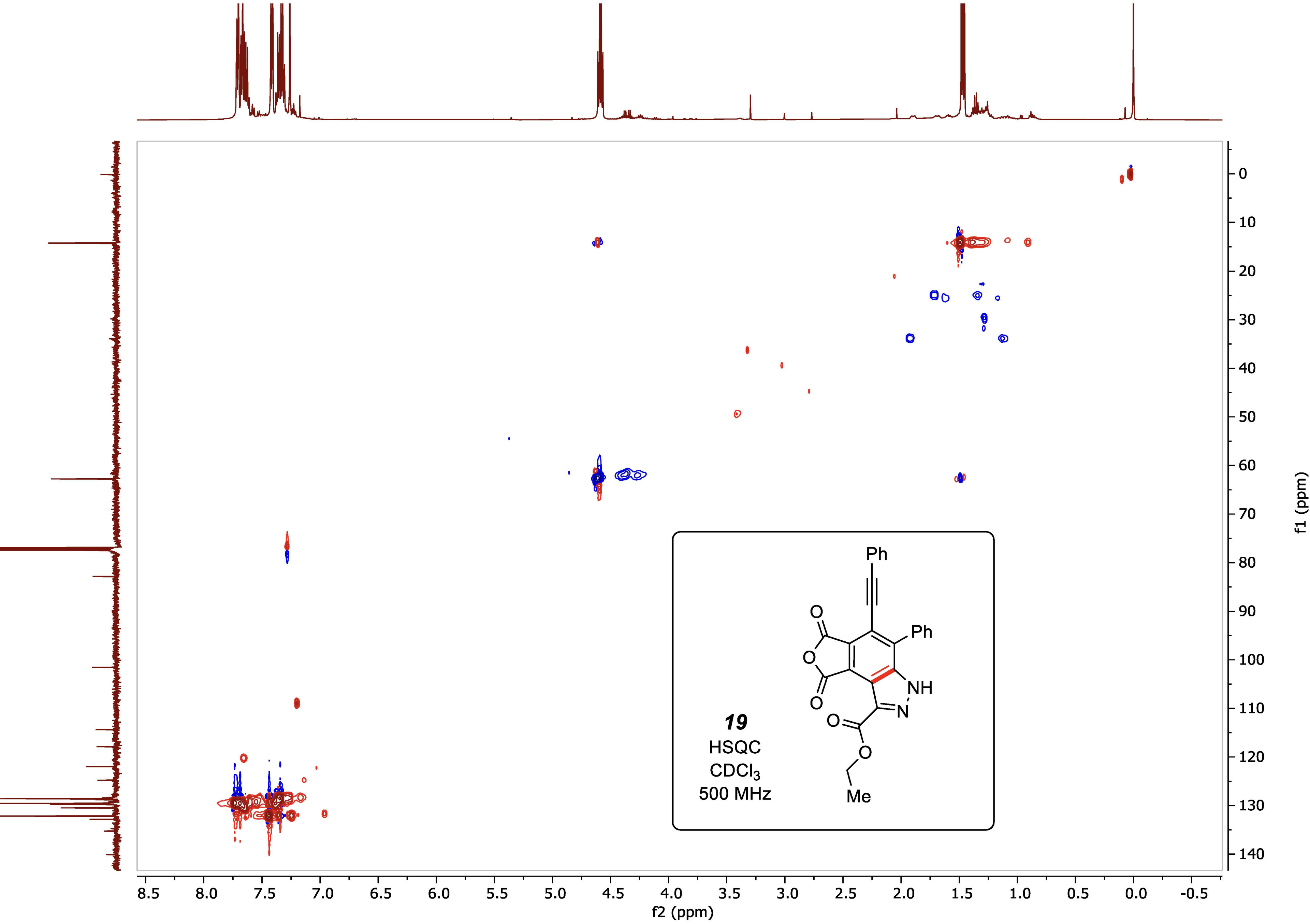
19
HSQC
$CDCl_3$
500 MHz
Ph
Ph
O
O
O
NH
N
O
O
Me
f2 (ppm)
f1 (ppm)
8.5
8.0
7.5
7.0
6.5
6.0
5.5
5.0
4.5
4.0
3.5
3.0
2.5
2.0
1.5
1.0
0.5
0.0
-0.5
0
10
20
30
40
50
60
70
80
90
100
110
120
130
140

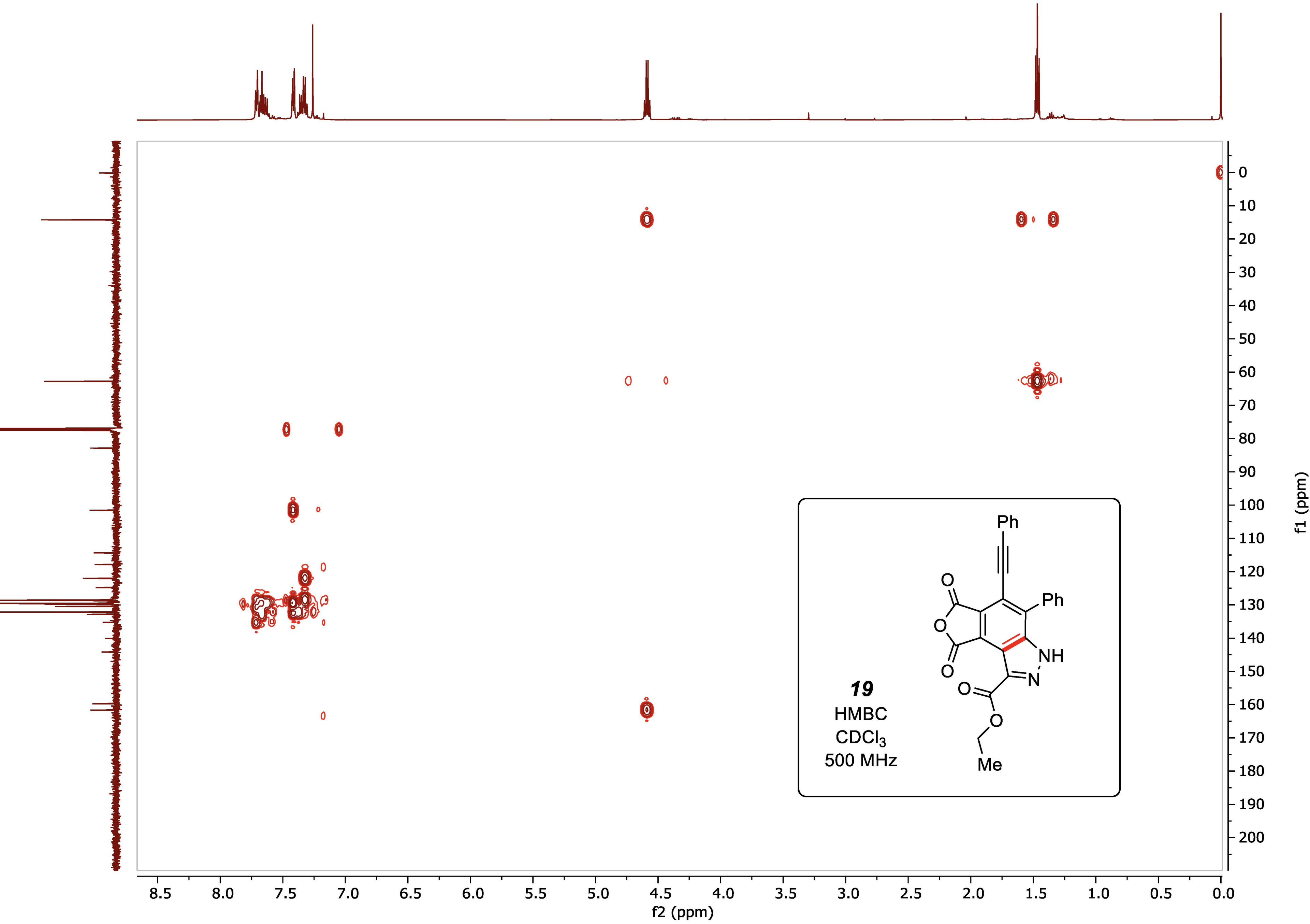

19
HMBC
$CDCl_3$
500 MHz

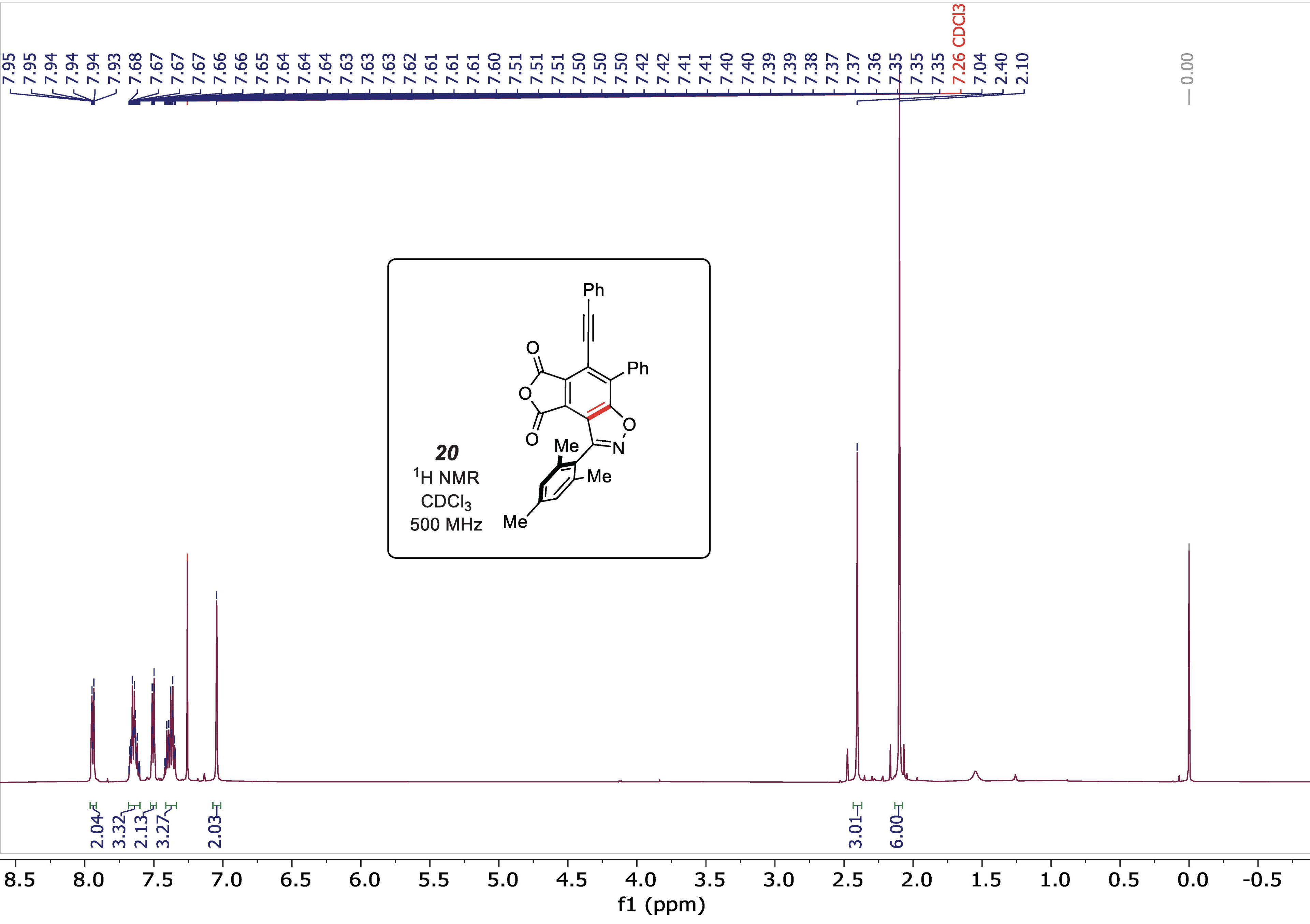
7.95 7.95 7.94 7.94 7.94 7.93 7.68 7.67 7.67 7.67 7.66 7.66 7.65 7.64 7.64 7.64 7.63 7.63 7.63 7.62 7.61 7.61 7.61 7.60 7.51 7.51 7.51 7.50 7.50 7.50 7.42 7.42 7.41 7.41 7.40 7.40 7.39 7.39 7.38 7.37 7.37 7.36 7.35 7.35 7.35 7.26 CDCl3 7.04 2.40 2.10
0.00
20
1H NMR
CDCl3
500 MHz
Ph
O
O
O
Ph
O
N
Me
Me
Me
2.04 3.32 2.13 3.27 2.03 3.01 6.00
8.5 8.0 7.5 7.0 6.5 6.0 5.5 5.0 4.5 4.0 3.5 3.0 2.5 2.0 1.5 1.0 0.5 0.0 -0.5
f1 (ppm)

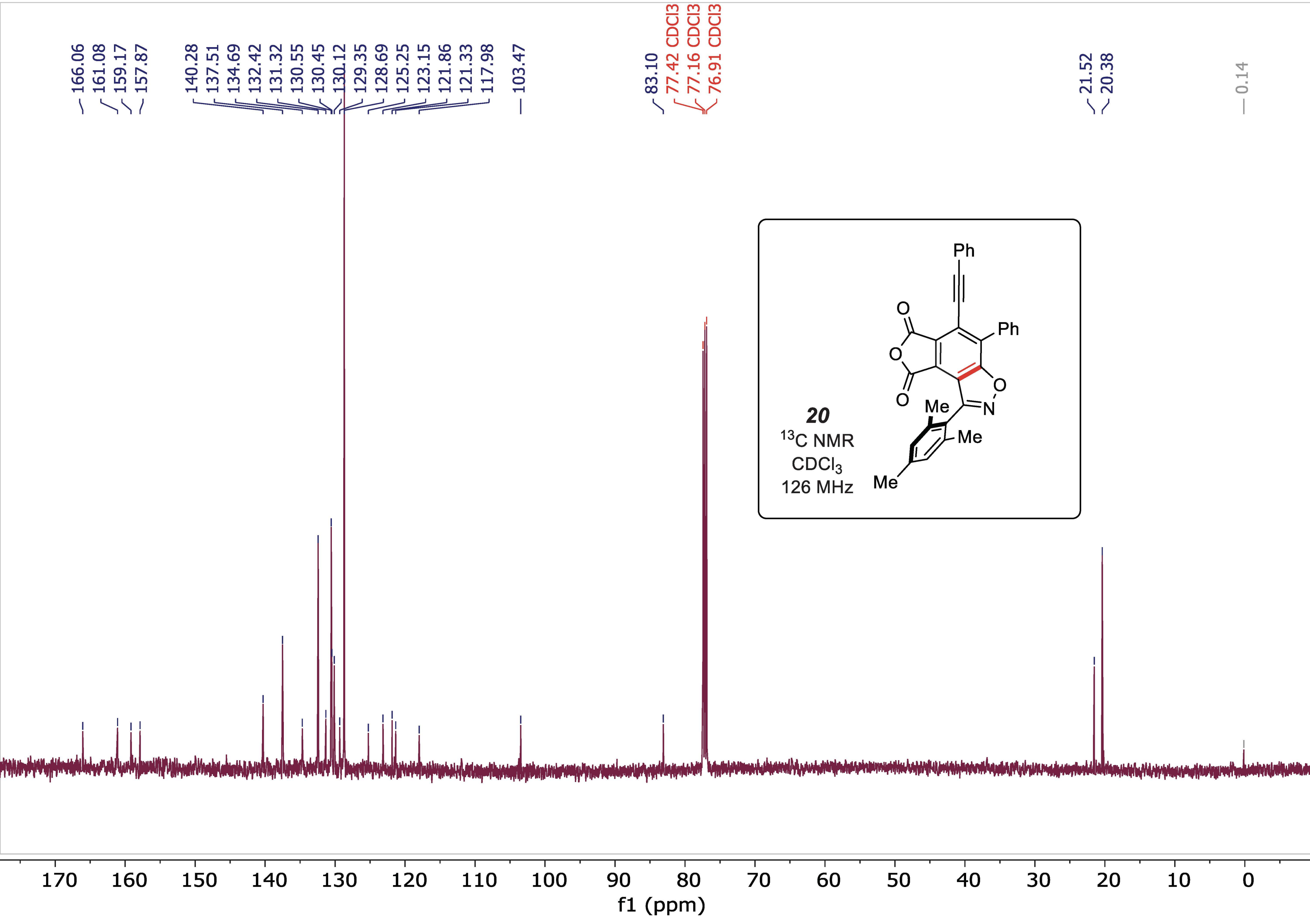
166.06
161.08
159.17
157.87
140.28
137.51
134.69
132.42
131.32
130.55
130.45
130.12
129.35
128.69
125.25
123.15
121.86
121.33
117.98
103.47
83.10
77.42 CDCl3
77.16 CDCl3
76.91 CDCl3
21.52
20.38
0.14
Ph
O
Ph
O
O
O
N
Me
Me
Me
20
^{13}C NMR
$CDCl_3$
126 MHz
f1 (ppm)

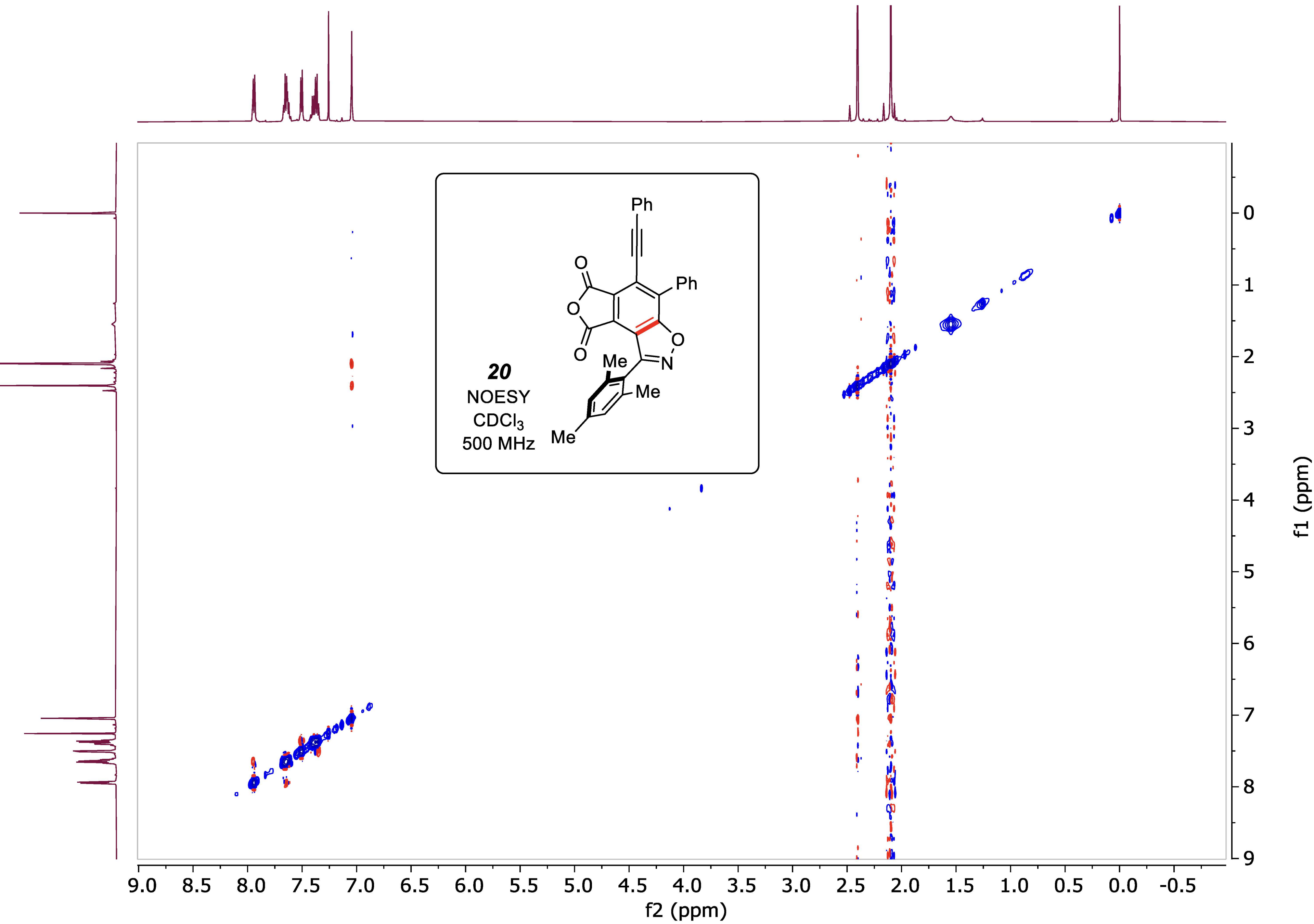
Ph
O
O
O
Ph
O
N
Me
Me
Me
20
NOESY
CDCl3
500 MHz
f1 (ppm)
f2 (ppm)

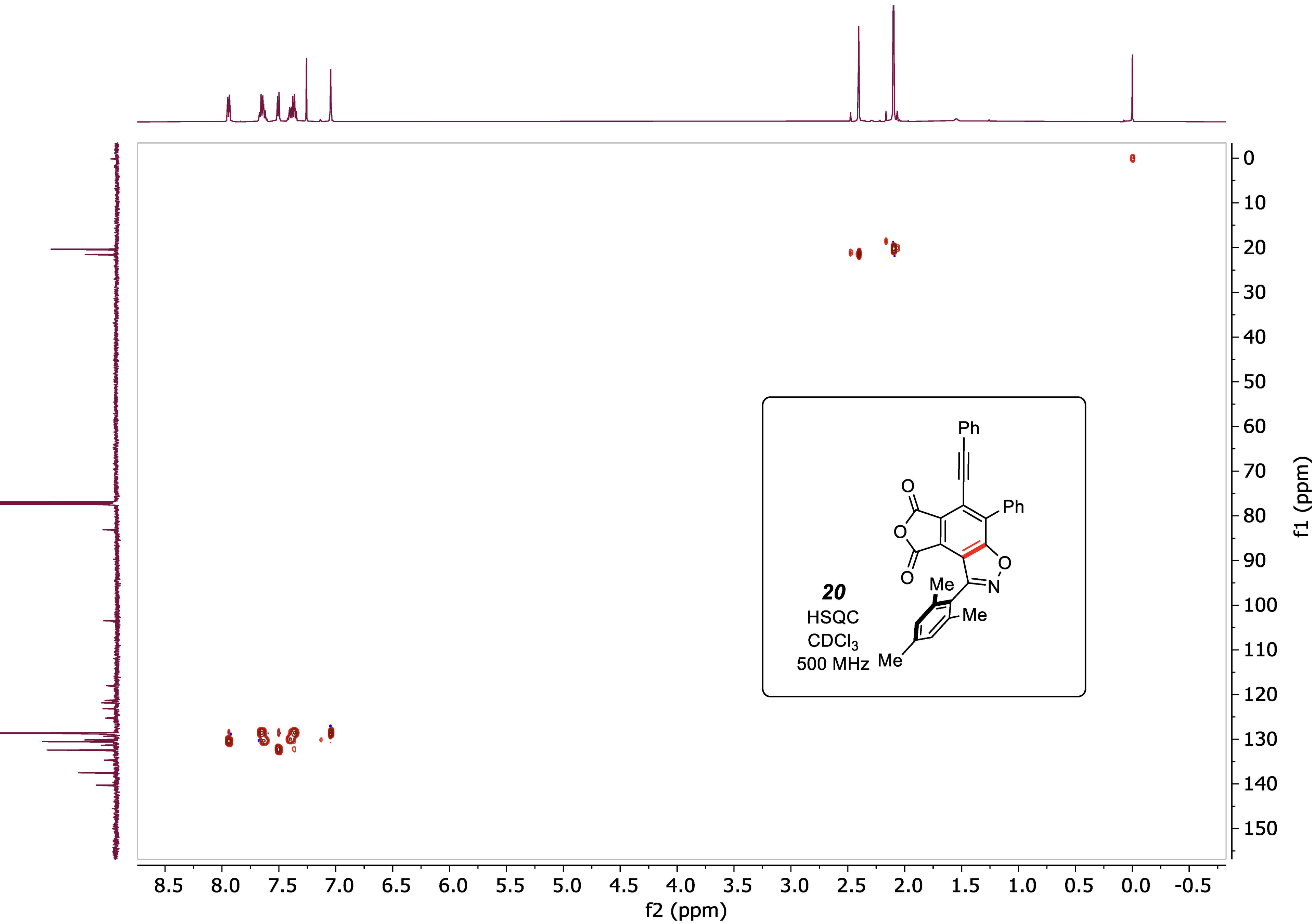
20
HSQC
$CDCl_3$
500 MHz
Ph
Ph
O
O
O
O
N
Me
Me
Me
f1 (ppm)
f2 (ppm)
8.5 8.0 7.5 7.0 6.5 6.0 5.5 5.0 4.5 4.0 3.5 3.0 2.5 2.0 1.5 1.0 0.5 0.0 -0.5
0 10 20 30 40 50 60 70 80 90 100 110 120 130 140 150

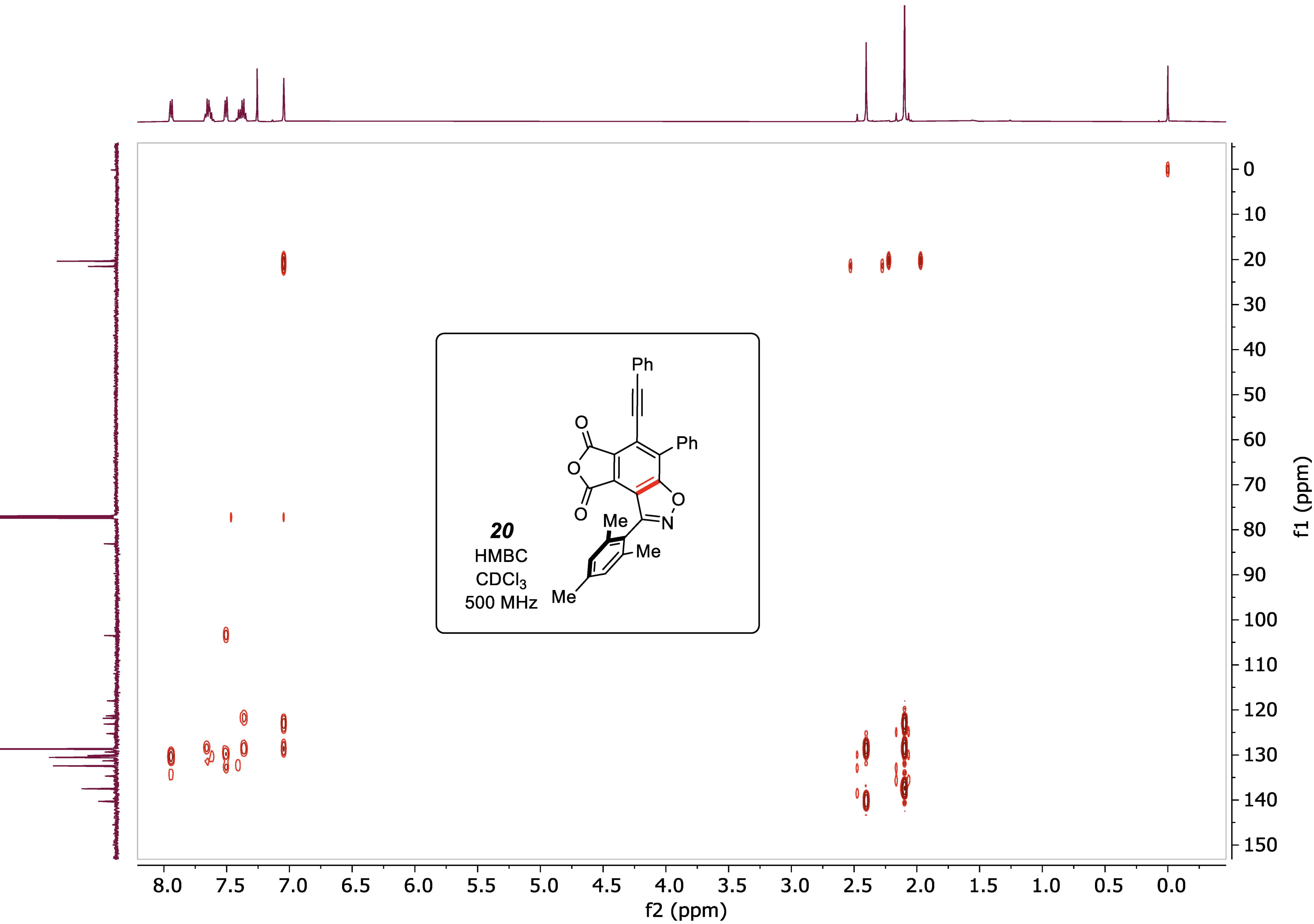

Ph
O
Ph
O
O
O
N
Me
Me
Me
20
HMBC
CDCl3
500 MHz
f1 (ppm)
f2 (ppm)

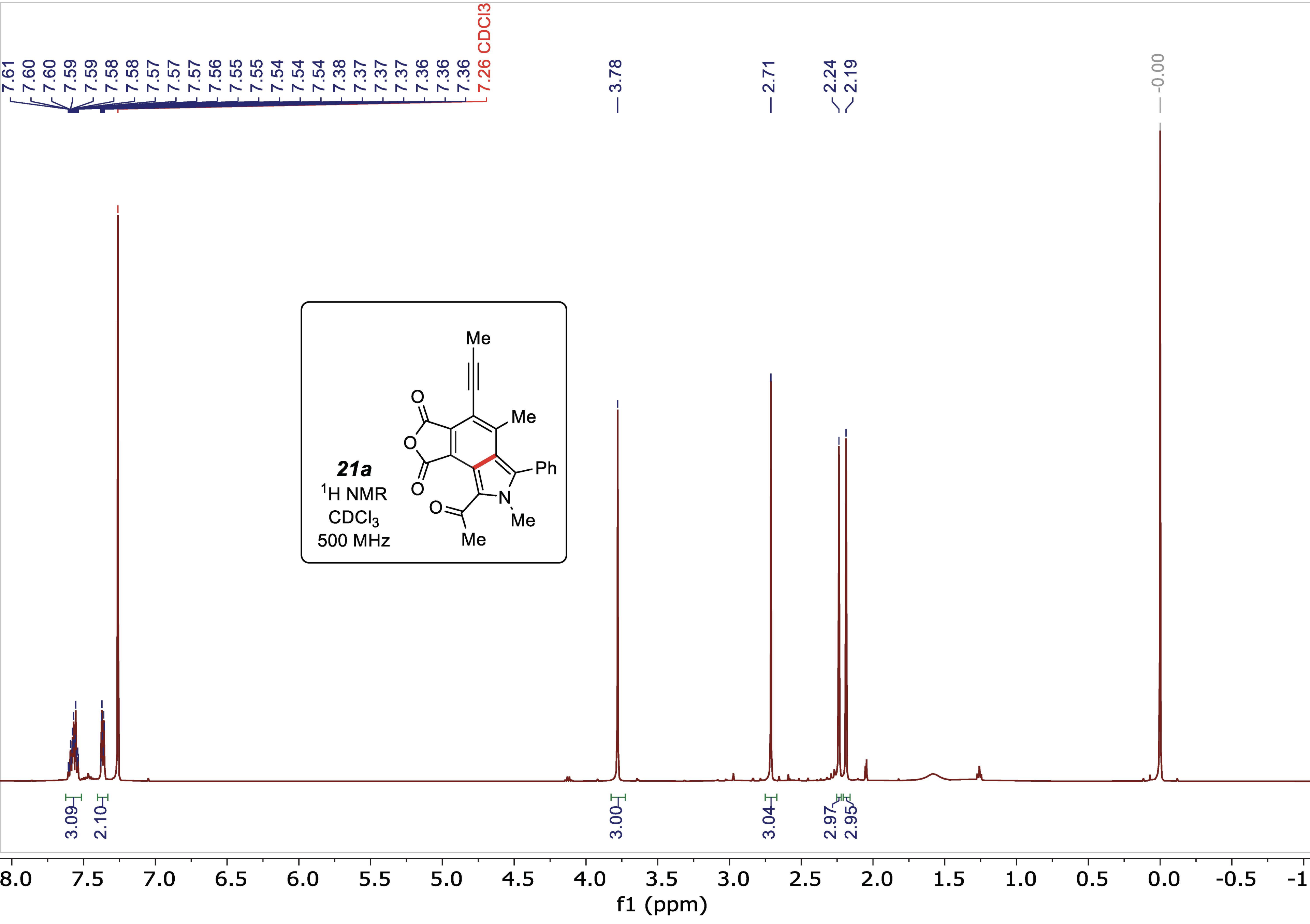
7.61 7.60 7.60 7.59 7.59 7.58 7.58 7.57 7.57 7.57 7.56 7.55 7.55 7.54 7.54 7.54 7.38 7.37 7.37 7.37 7.36 7.36 7.36 7.26 CDCl3
3.78
2.71
2.24 2.19
-0.00
21a
1H NMR
CDCl3
500 MHz
Me
O
Me
O
Ph
O
N
O
Me
Me
3.09 2.10 3.00 3.04 2.97 2.95
8.0 7.5 7.0 6.5 6.0 5.5 5.0 4.5 4.0 3.5 3.0 2.5 2.0 1.5 1.0 0.5 0.0 -0.5 -1
f1 (ppm)

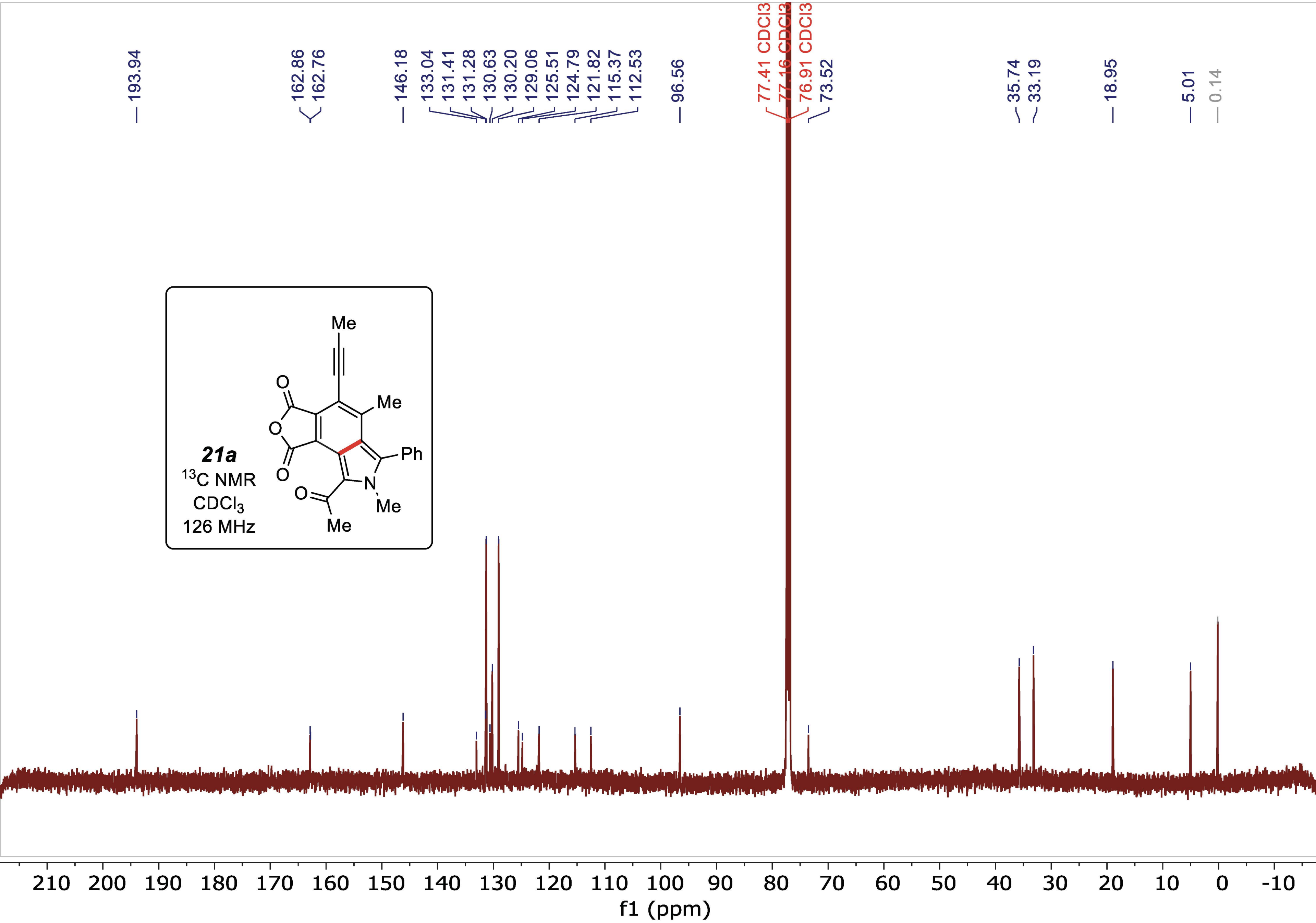
193.94
162.86
162.76
146.18
133.04
131.41
131.28
130.63
130.20
129.06
125.51
124.79
121.82
115.37
112.53
96.56
77.41 CDCl3
77.16 CDCl3
76.91 CDCl3
73.52
35.74
33.19
18.95
5.01
0.14
21a
13C NMR
CDCl3
126 MHz
Me
O
O
O
Me
Ph
N
Me
O
Me
f1 (ppm)

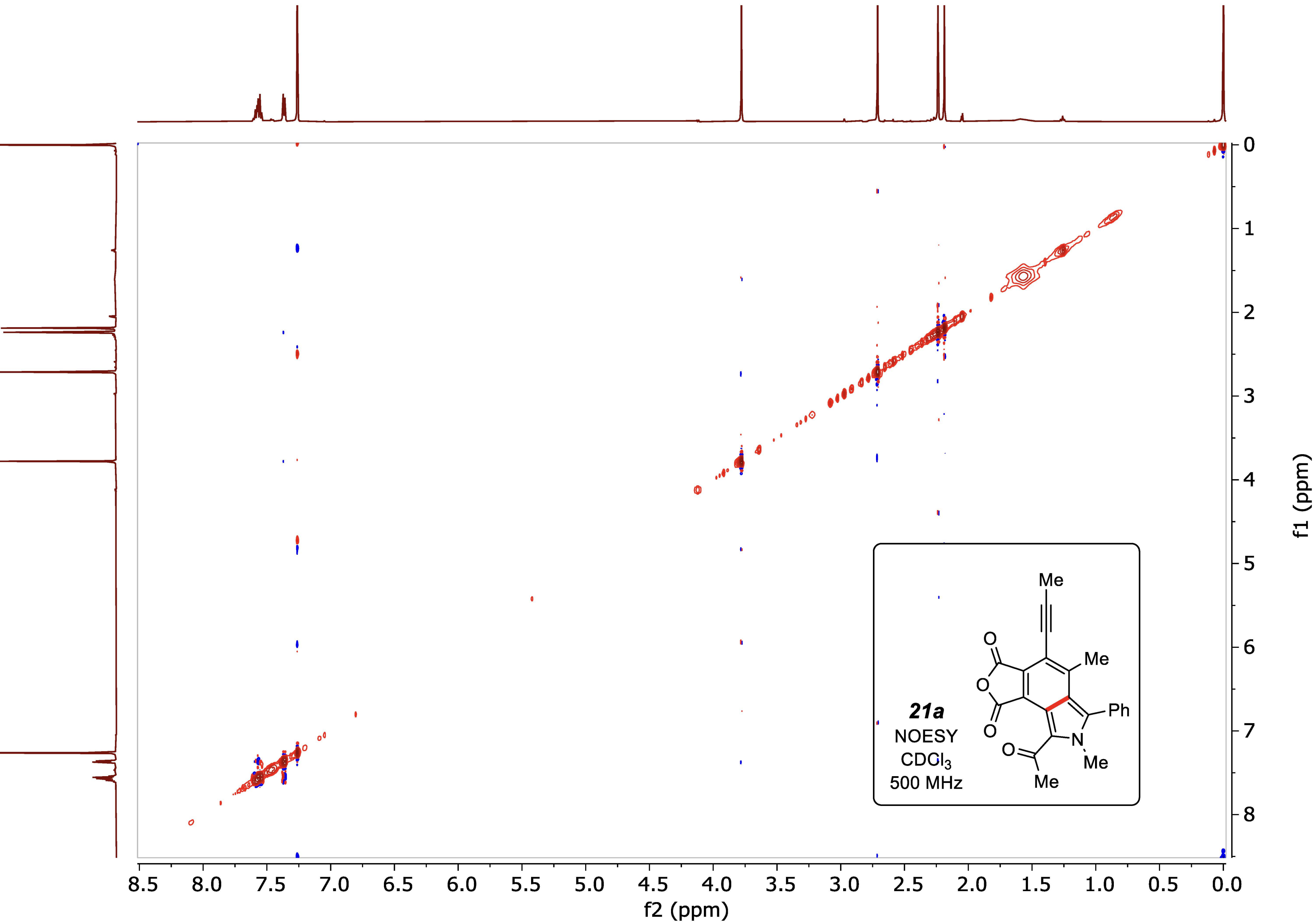
21a
NOESY
$CDCl_3$
500 MHz
Me
O
Me
O
Ph
O
N
O
Me
Me
f1 (ppm)
f2 (ppm)

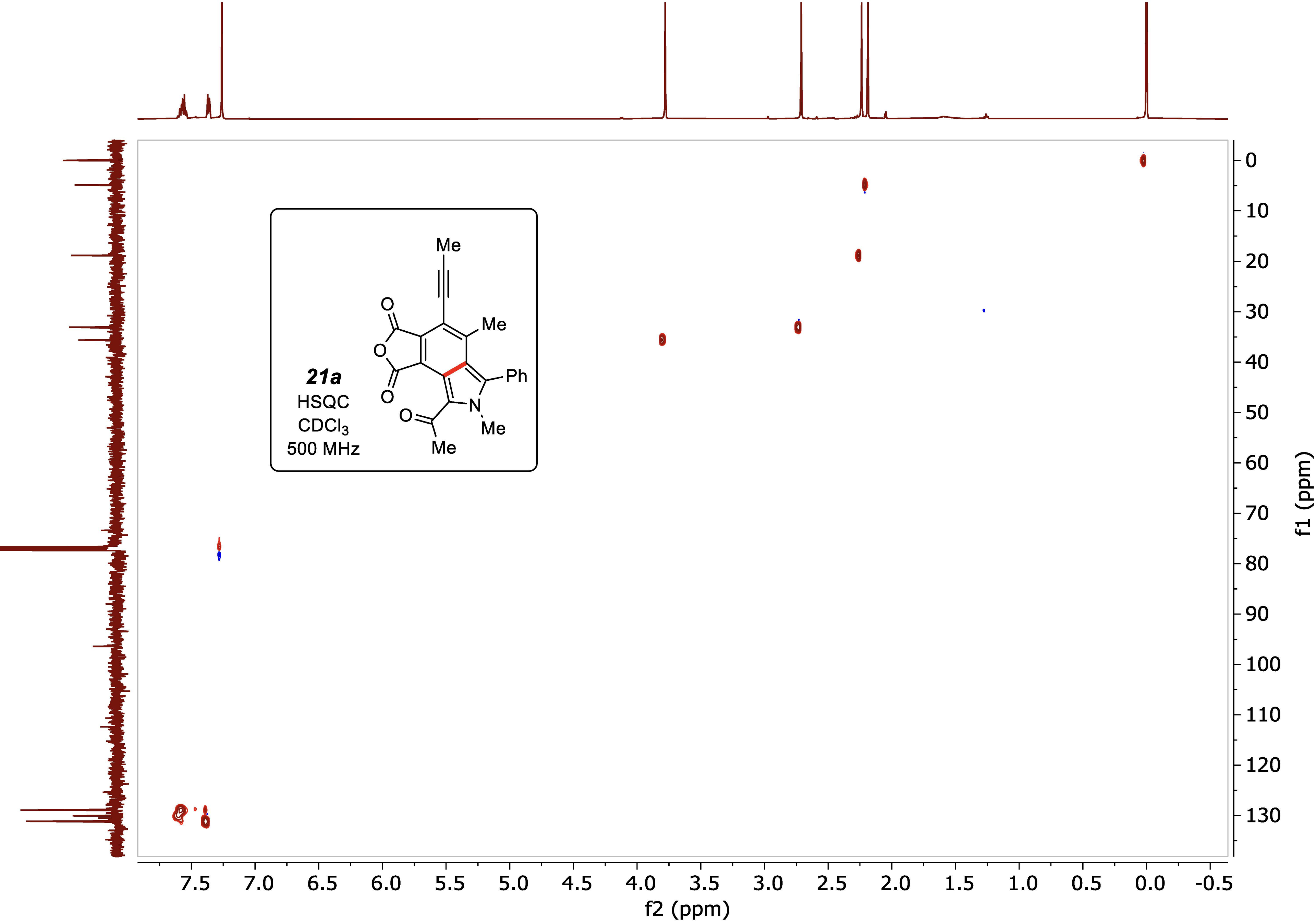
Me
O
Me
O
Ph
O
N
O
Me
Me
21a
HSQC
CDCl3
500 MHz
f1 (ppm)
f2 (ppm)
7.5 7.0 6.5 6.0 5.5 5.0 4.5 4.0 3.5 3.0 2.5 2.0 1.5 1.0 0.5 0.0 -0.5
0 10 20 30 40 50 60 70 80 90 100 110 120 130

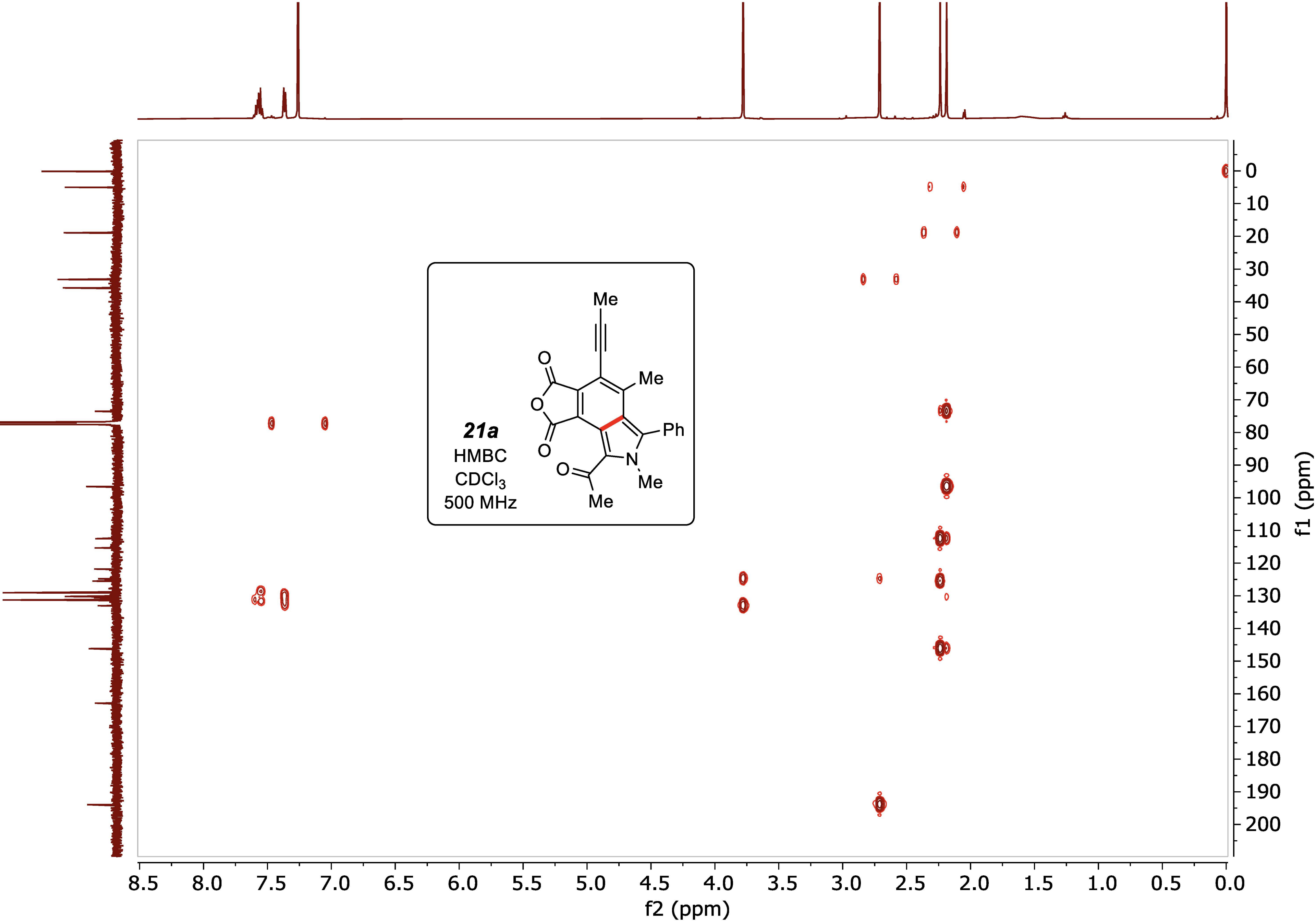
21a
HMBC
CDCl3
500 MHz
Me
O
O
O
Me
Ph
N
Me
O
Me
f1 (ppm)
f2 (ppm)

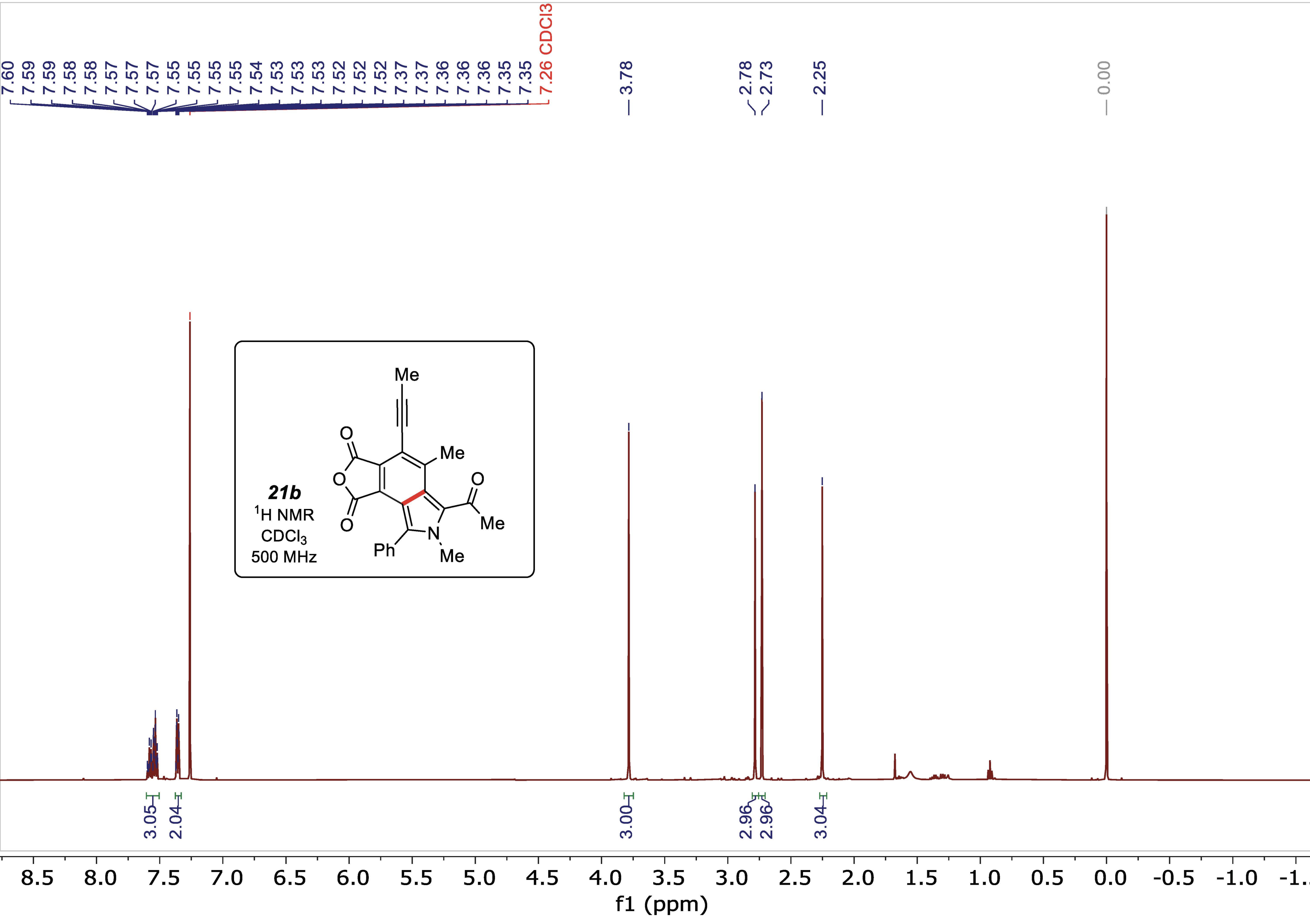
7.60 7.59 7.59 7.58 7.58 7.57 7.57 7.57 7.55 7.55 7.55 7.55 7.54 7.53 7.53 7.53 7.52 7.52 7.52 7.37 7.37 7.36 7.36 7.35 7.35 7.26 CDCl3
3.78
2.78
2.73
2.25
0.00
21b
^{1}H NMR
$CDCl_3$
500 MHz
Me
O
Me
O
O
O
Me
Ph
N
Me
3.05
2.04
3.00
2.96
2.96
3.04
8.5 8.0 7.5 7.0 6.5 6.0 5.5 5.0 4.5 4.0 3.5 3.0 2.5 2.0 1.5 1.0 0.5 0.0 -0.5 -1.0
f1 (ppm)

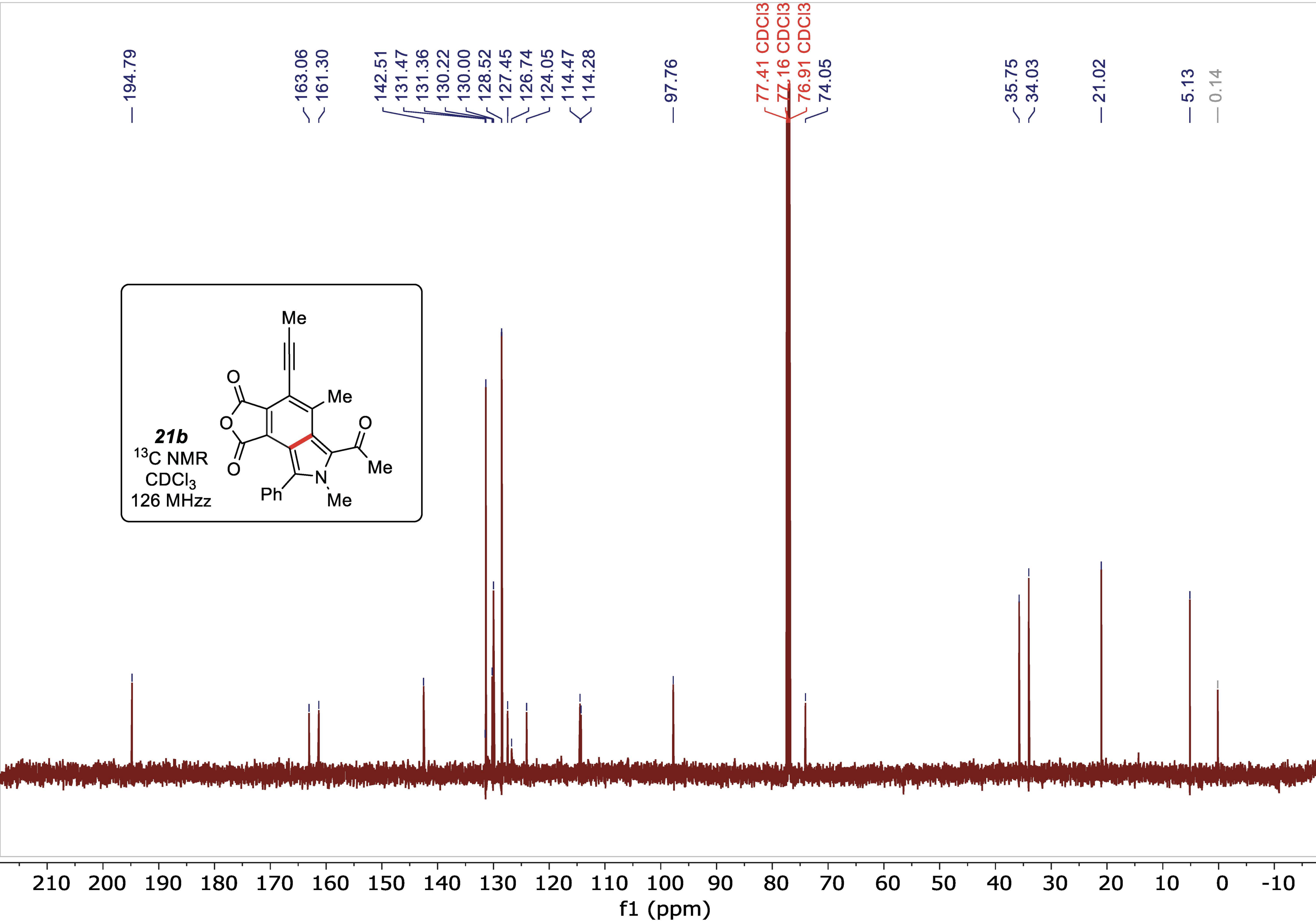
194.79
163.06
161.30
142.51
131.47
131.36
130.22
130.00
128.52
127.45
126.74
124.05
114.47
114.28
97.76
77.41 CDCl3
77.16 CDCl3
76.91 CDCl3
74.05
35.75
34.03
21.02
5.13
0.14
21b
13C NMR
CDCl3
126 MHzz
Me
O
Me
O
O
Me
O
Ph
N
Me
210 200 190 180 170 160 150 140 130 120 110 100 90 80 70 60 50 40 30 20 10 0 -10
f1 (ppm)

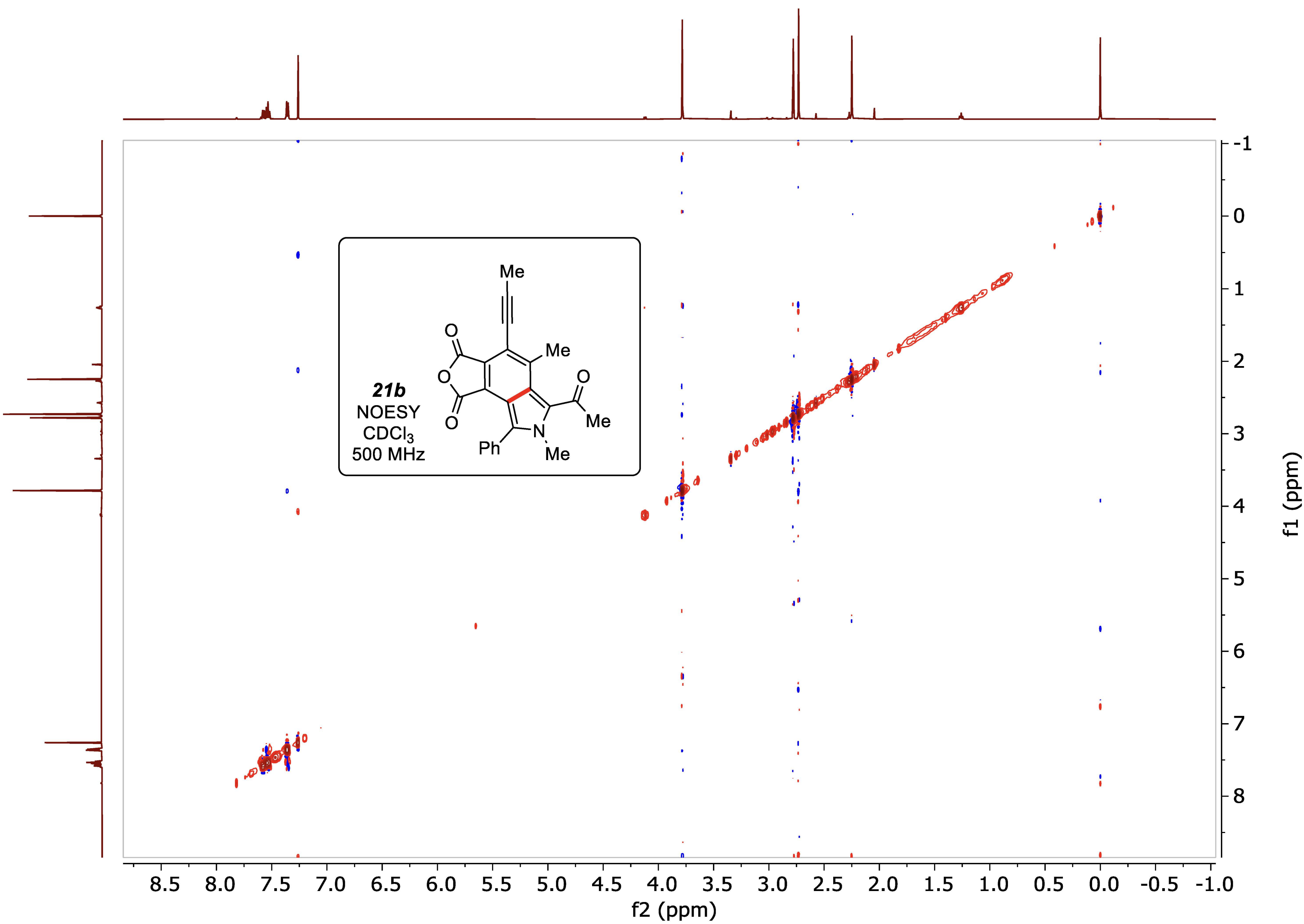
21b
NOESY
CDCl3
500 MHz
Me
O
Me
O
O
O
Me
O
N
Ph
Me
f2 (ppm)
f1 (ppm)
8.5 8.0 7.5 7.0 6.5 6.0 5.5 5.0 4.5 4.0 3.5 3.0 2.5 2.0 1.5 1.0 0.5 0.0 -0.5 -1.0
-1 0 1 2 3 4 5 6 7 8

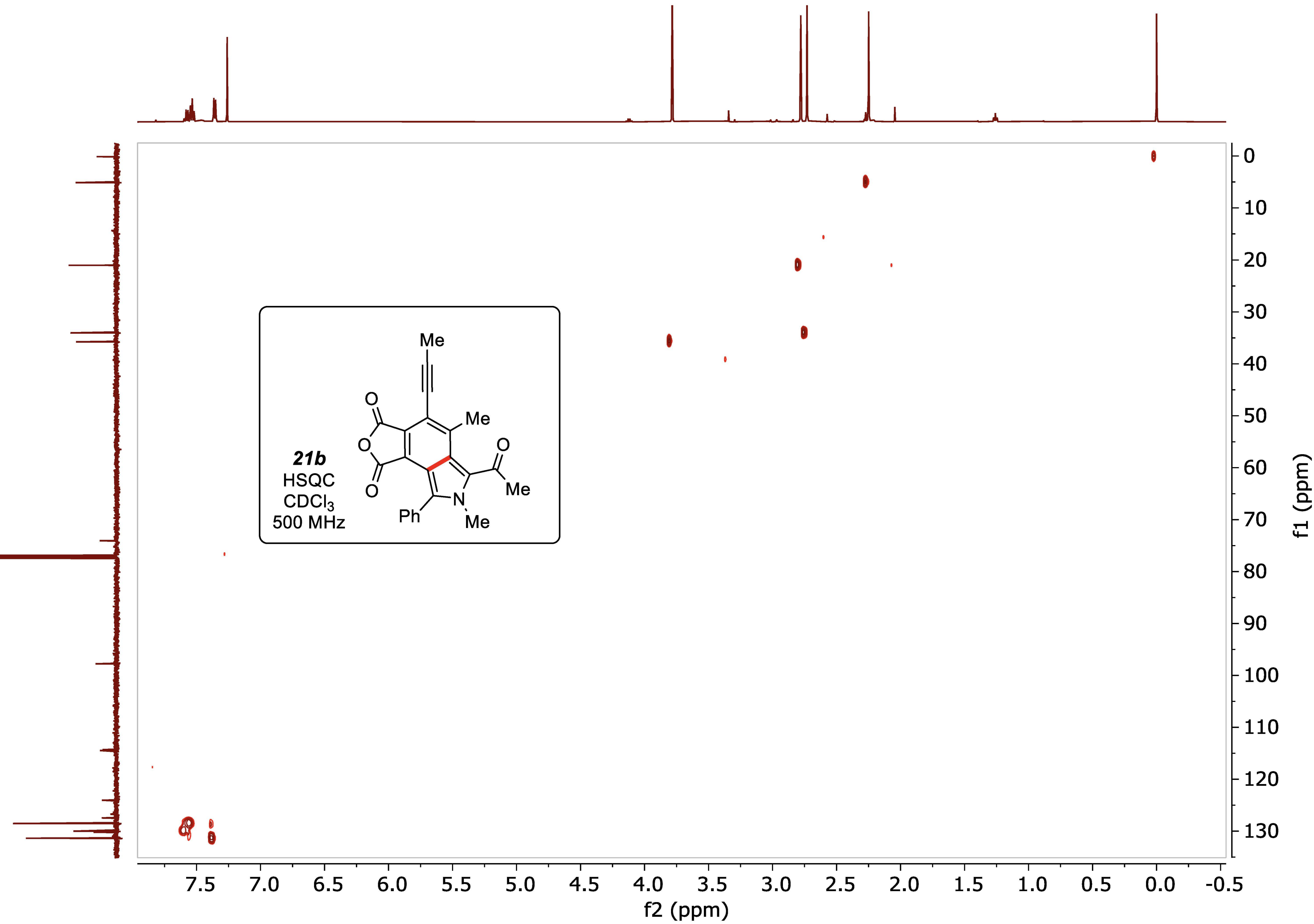
21b
HSQC
$CDCl_3$
500 MHz
Me
O
Me
O
O
Me
O
N
Ph
Me
f2 (ppm)
f1 (ppm)

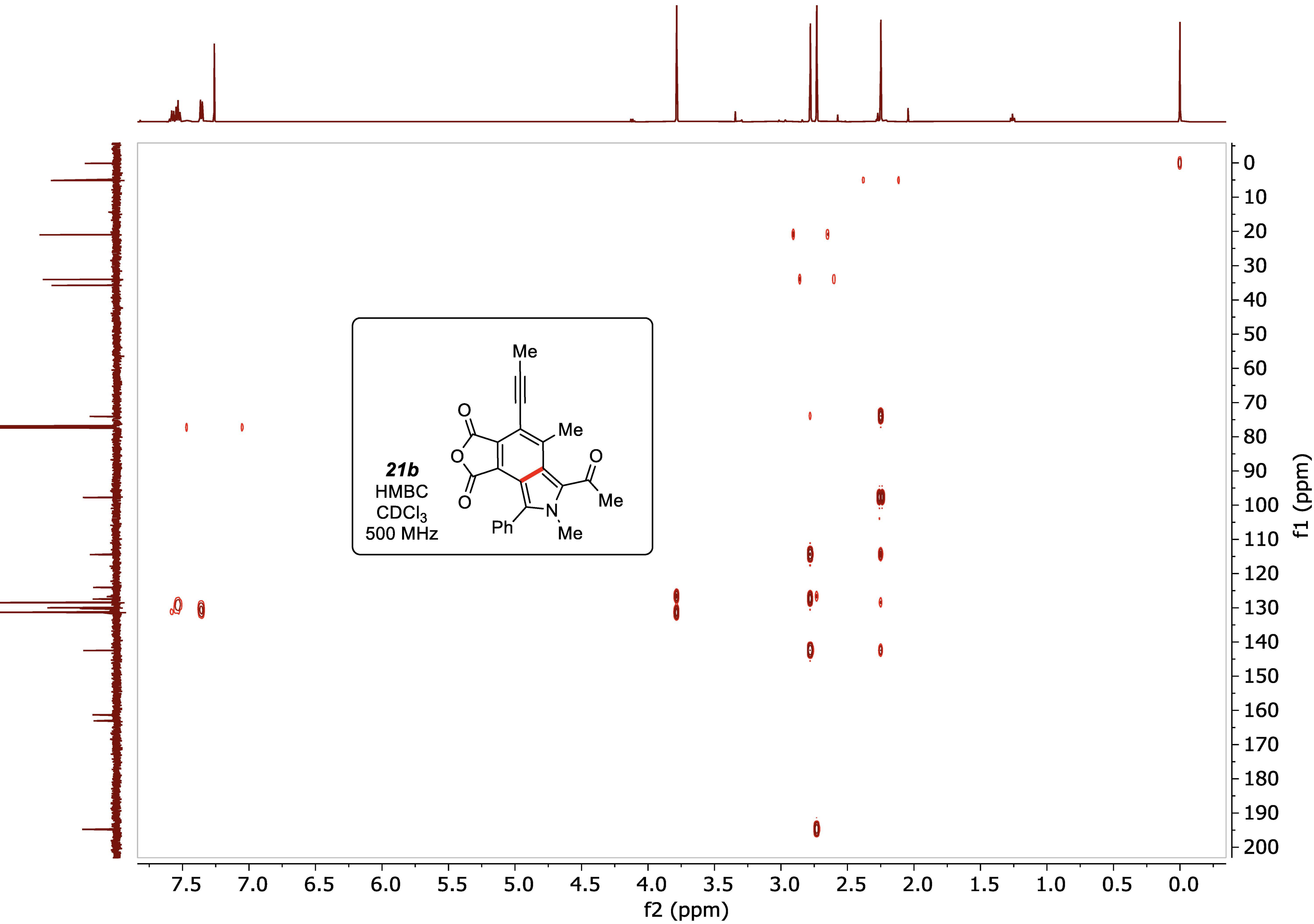
21b
HMBC
CDCl3
500 MHz
Me
O
O
O
Me
O
Me
N
Ph
Me
f2 (ppm)
f1 (ppm)

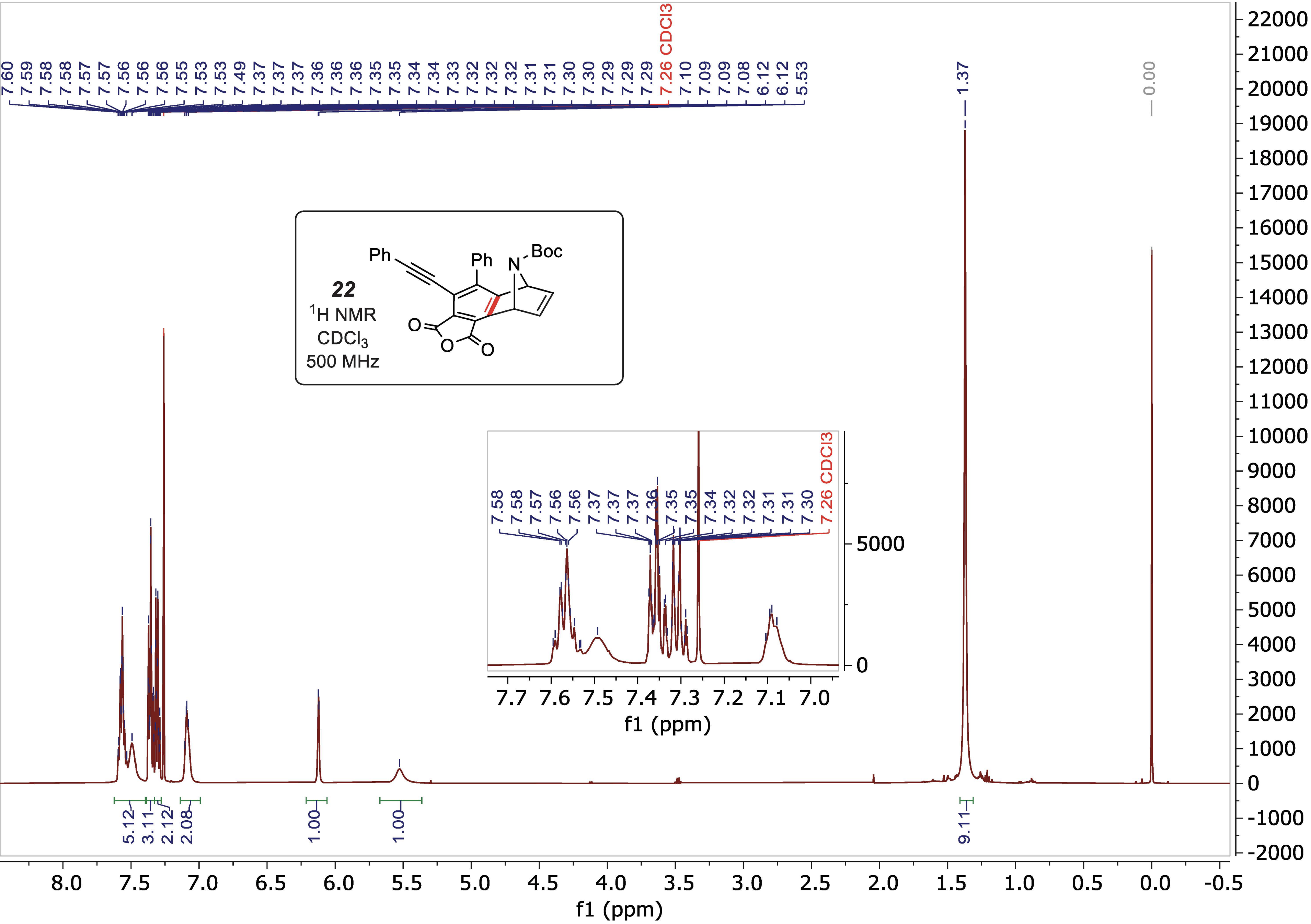

22
^{1}H NMR
$CDCl_3$
500 MHz
Ph
Ph
N
Boc
O
O
O
7.26 CDCl3
1.37
0.00
5.12
3.11
2.12
2.08
1.00
1.00
9.11
f1 (ppm)

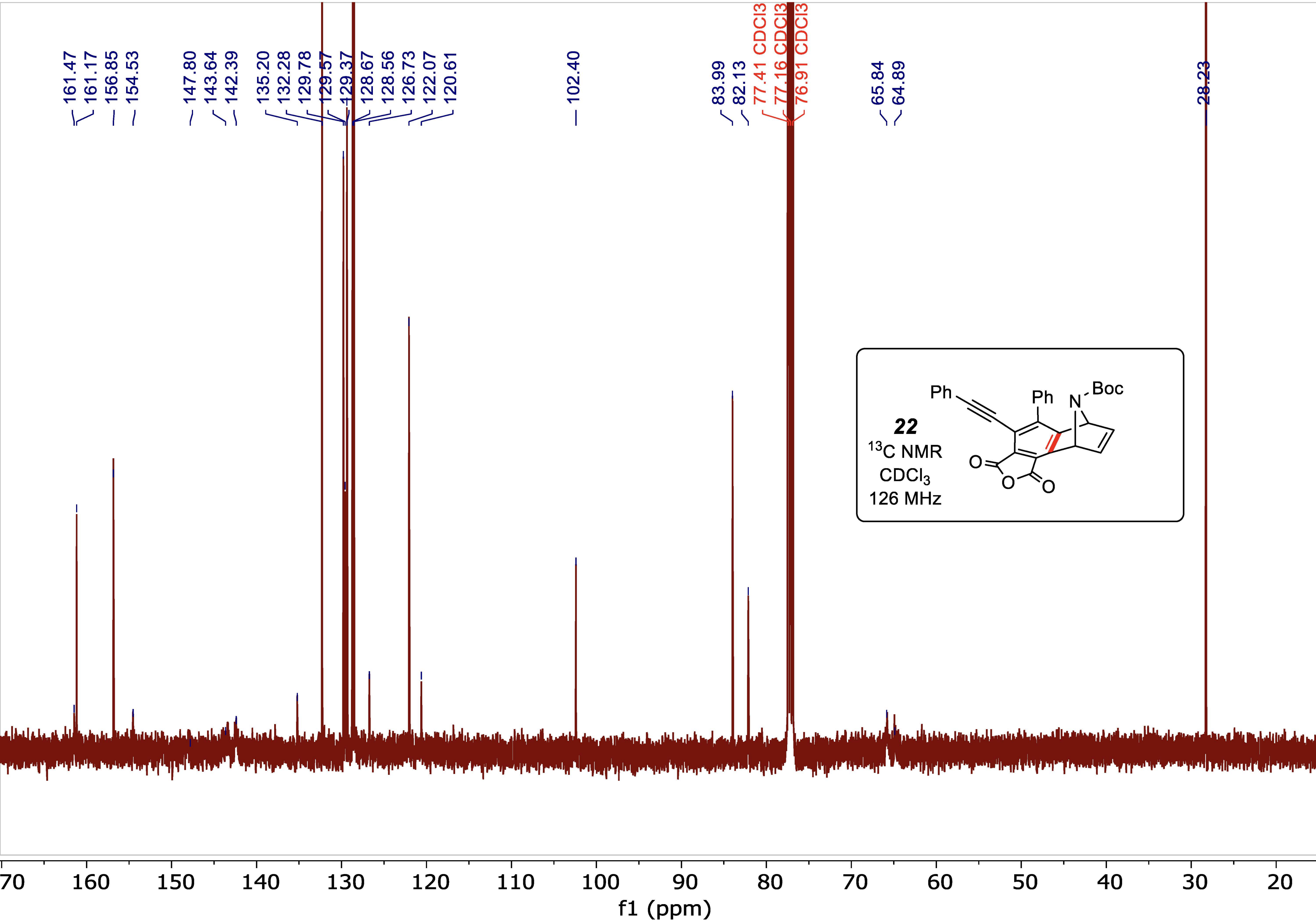
161.47
161.17
156.85
154.53
147.80
143.64
142.39
135.20
132.28
129.78
129.57
129.37
128.67
128.56
126.73
122.07
120.61
102.40
83.99
82.13
77.41 CDCl3
77.16 CDCl3
76.91 CDCl3
65.84
64.89
28.23
22
^{13}C NMR
$CDCl_3$
126 MHz
Ph
Ph
N
Boc
O
O
O
f1 (ppm)
70 160 150 140 130 120 110 100 90 80 70 60 50 40 30 20

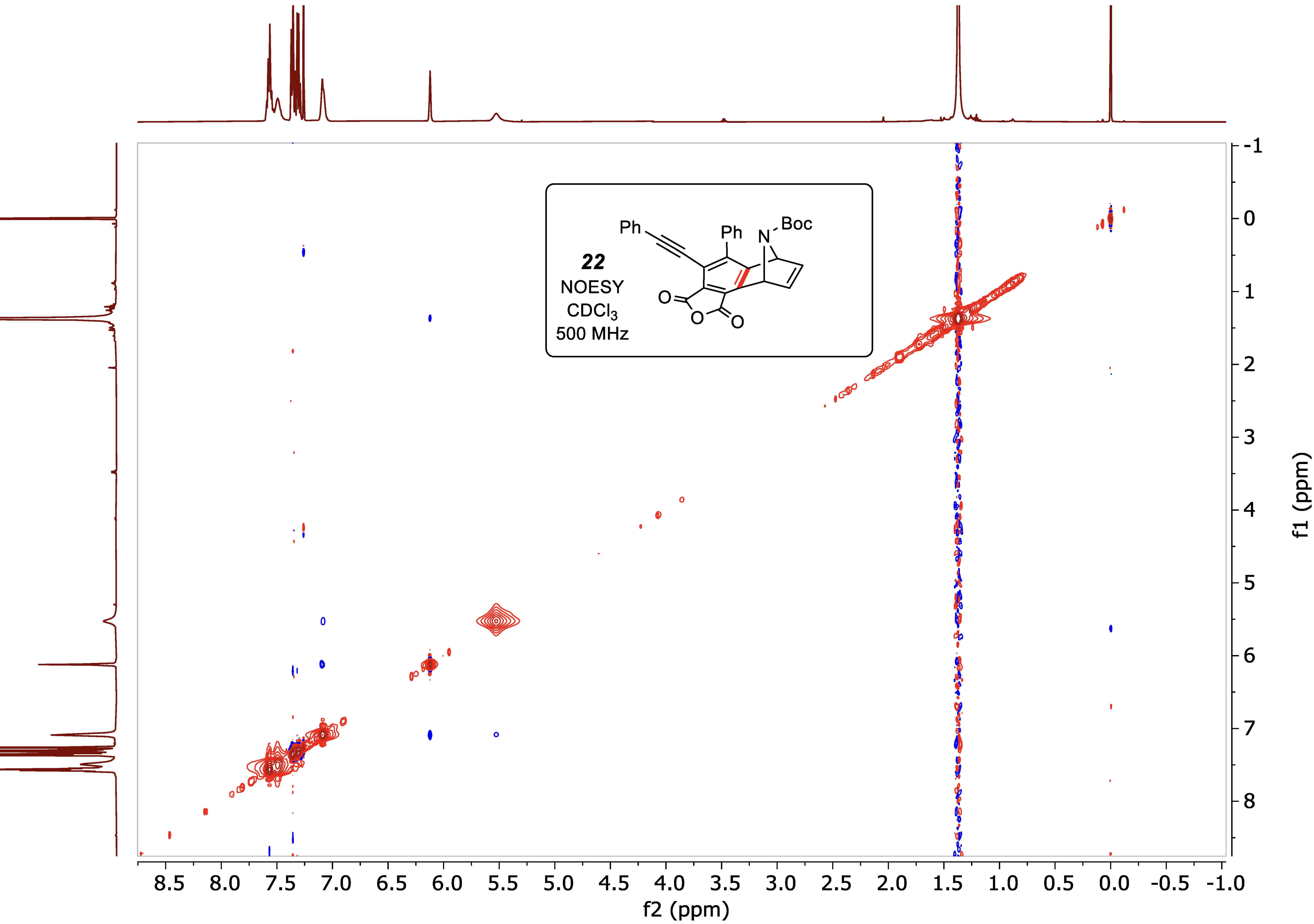

22
NOESY
$CDCl_3$
500 MHz
Ph
Ph
N
Boc
O
O
O
f2 (ppm)
f1 (ppm)

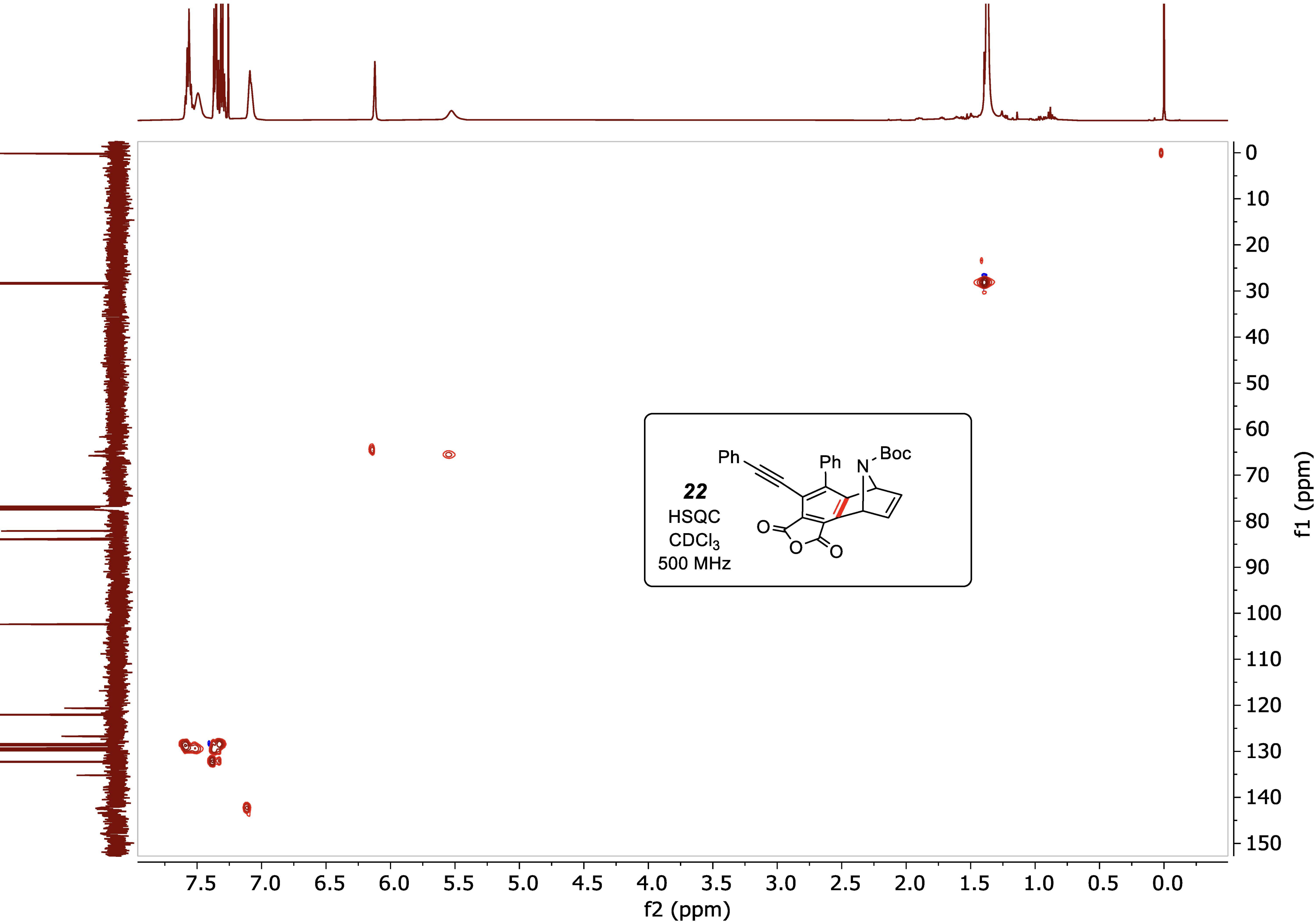
22
HSQC
$CDCl_3$
500 MHz
Ph
Ph
N
Boc
O
O
O
f1 (ppm)
f2 (ppm)

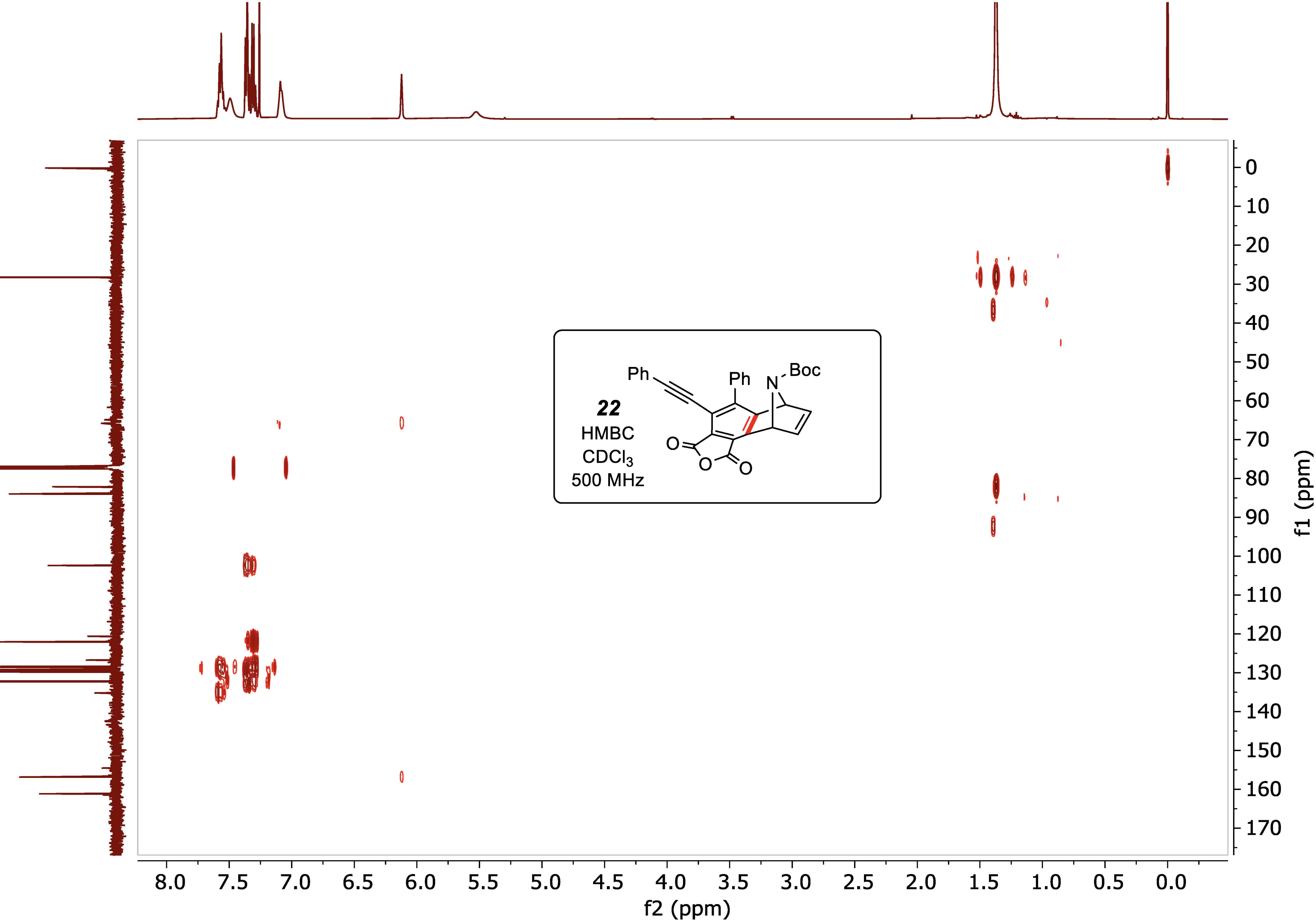
22
HMBC
CDCl3
500 MHz
Ph
Ph
N
Boc
O
O
O
f2 (ppm)
f1 (ppm)

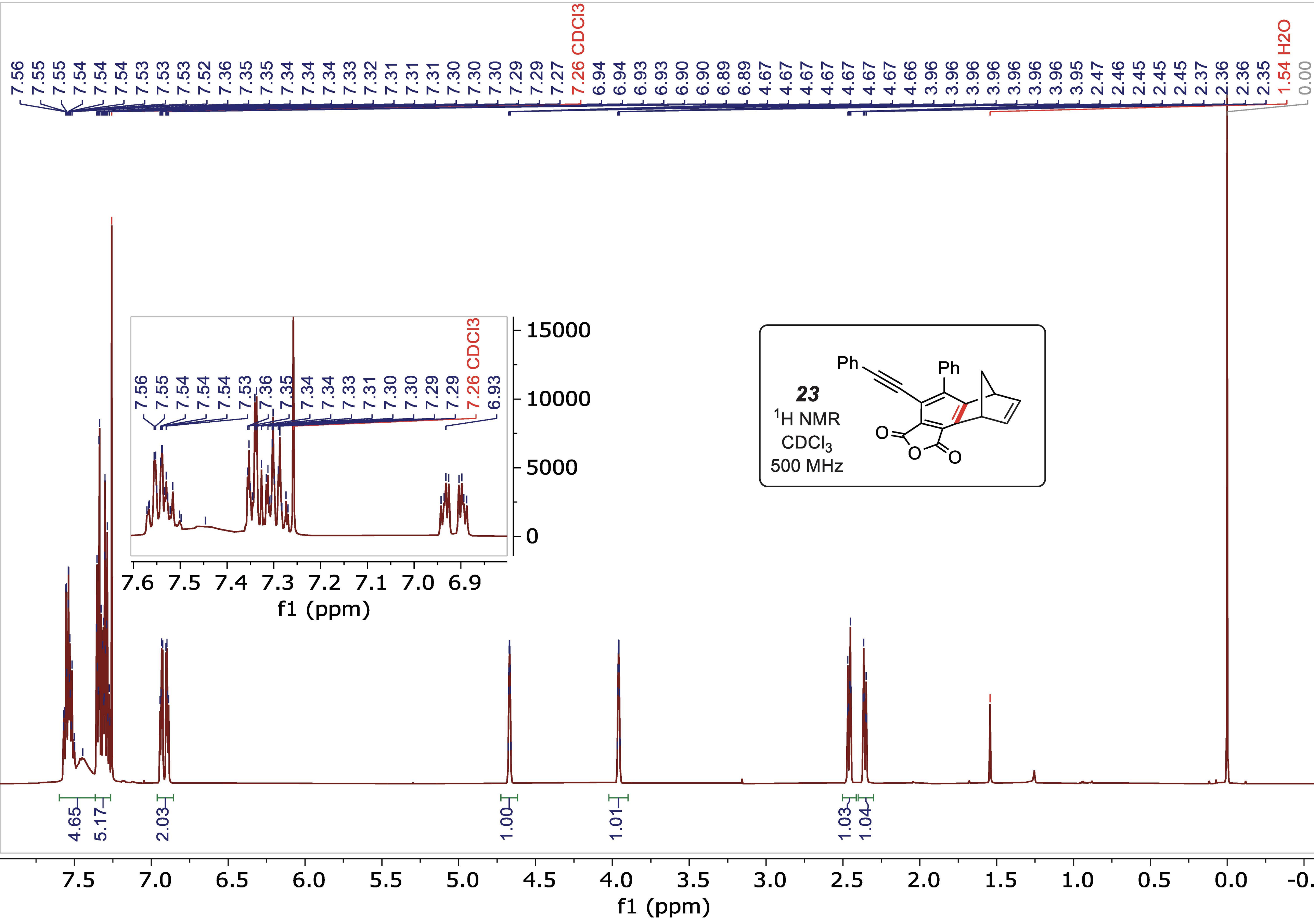
23
1H NMR
CDCl3
500 MHz
Ph
Ph
O
O
O
7.26 CDCl3
1.54 H2O
0.00
4.65
5.17
2.03
1.00
1.01
1.03
1.04
f1 (ppm)
f1 (ppm)

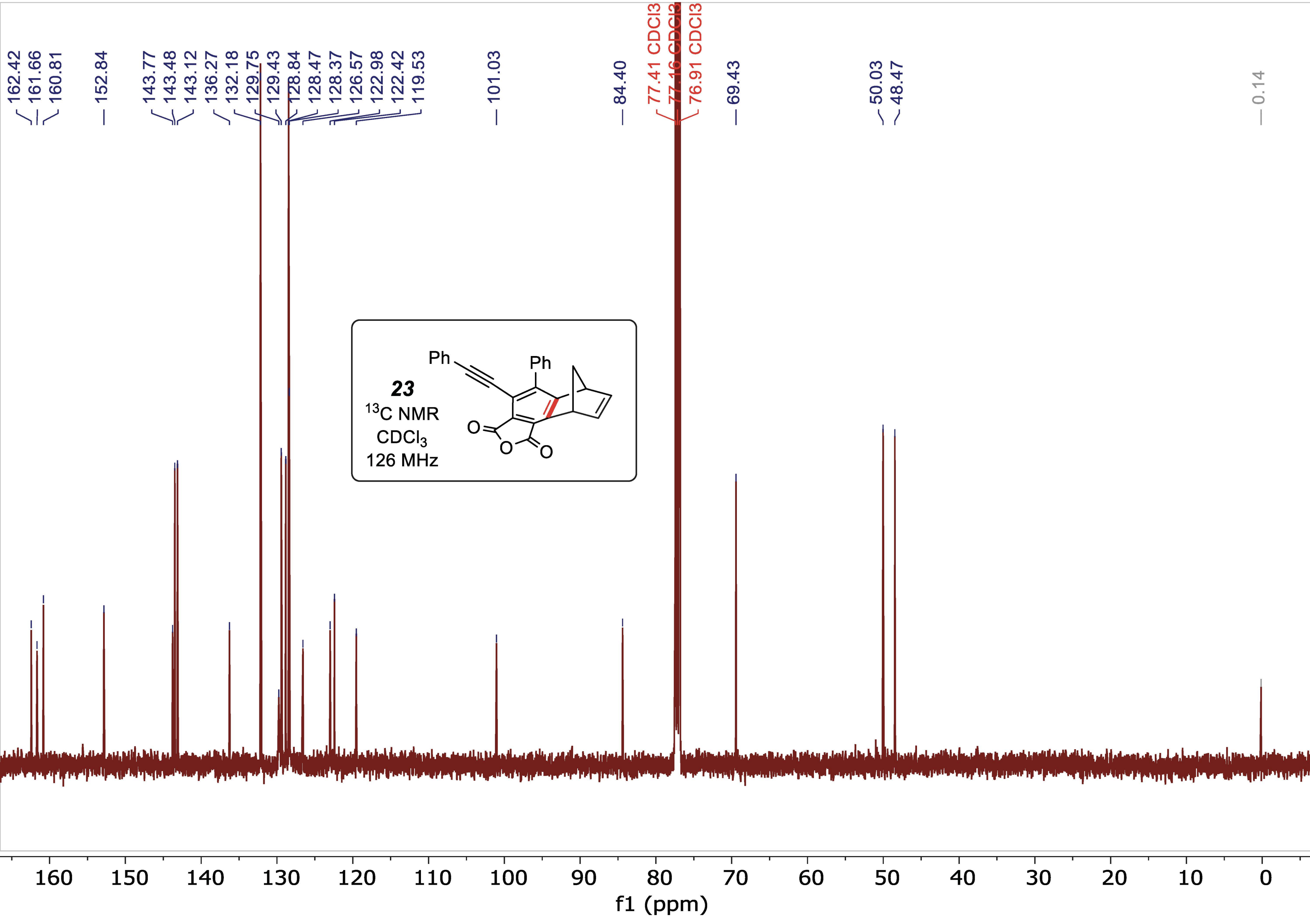
162.42
161.66
160.81
152.84
143.77
143.48
143.12
136.27
132.18
129.75
129.43
128.84
128.47
128.37
126.57
122.98
122.42
119.53
101.03
84.40
77.41 CDCl3
77.16 CDCl3
76.91 CDCl3
69.43
50.03
48.47
0.14
23
13C NMR
CDCl3
126 MHz
Ph
Ph
O
O
O
f1 (ppm)

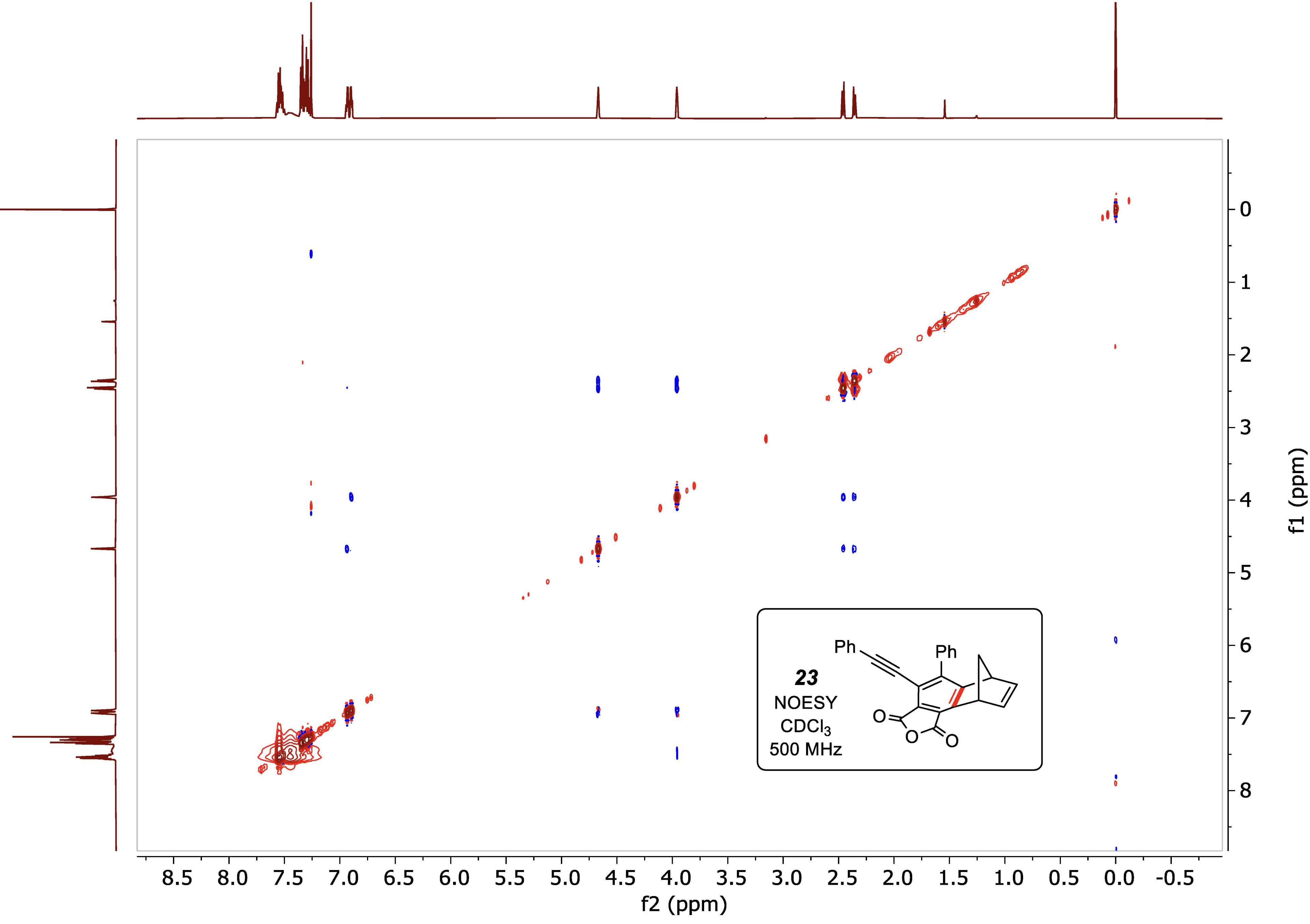

23
NOESY
$CDCl_3$
500 MHz
Ph
Ph
O
O
O
f2 (ppm)
f1 (ppm)
8.5 8.0 7.5 7.0 6.5 6.0 5.5 5.0 4.5 4.0 3.5 3.0 2.5 2.0 1.5 1.0 0.5 0.0 -0.5
0 1 2 3 4 5 6 7 8

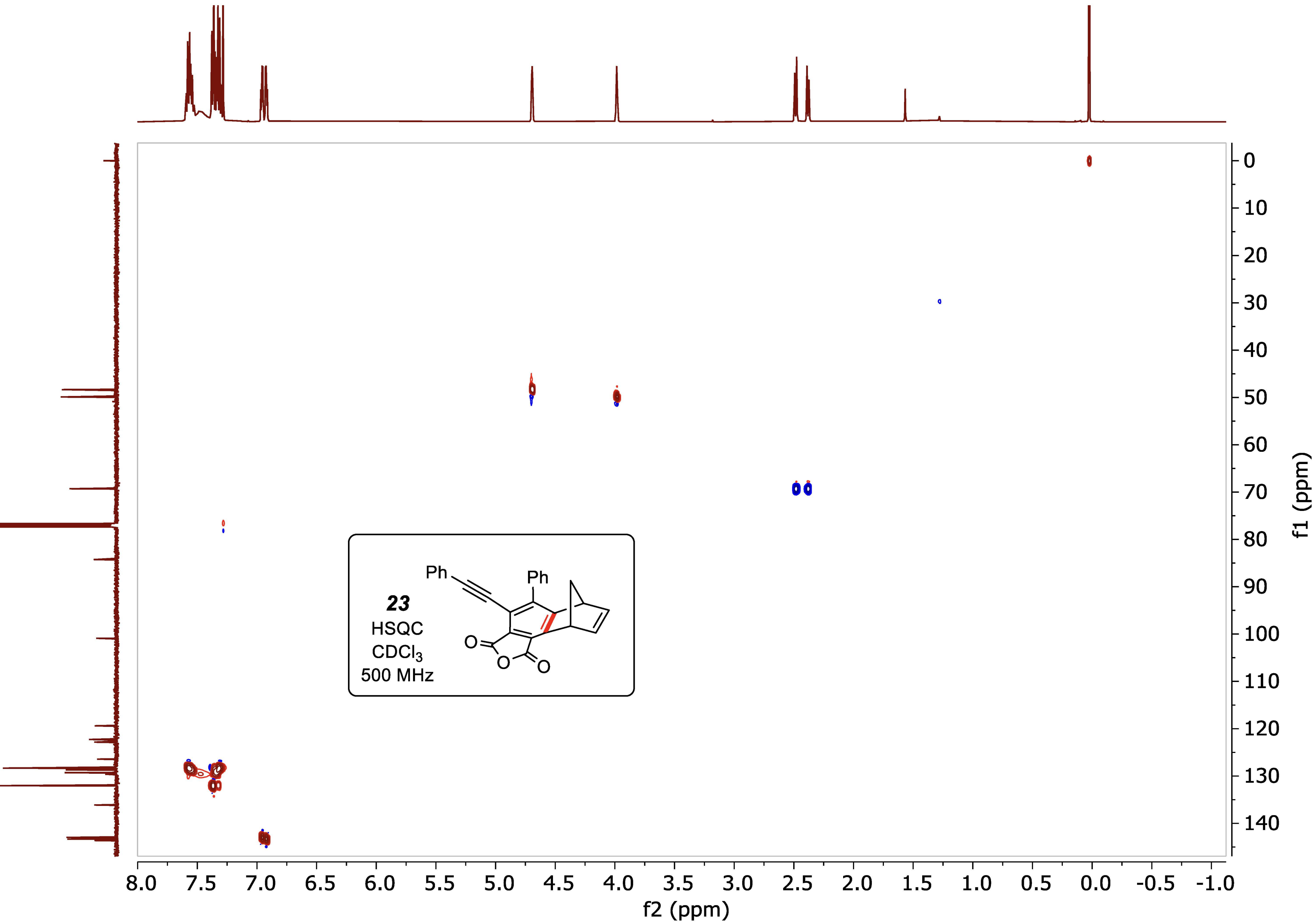

f2 (ppm)
f1 (ppm)
23
HSQC
$CDCl_3$
500 MHz
Ph
Ph
O
O
O

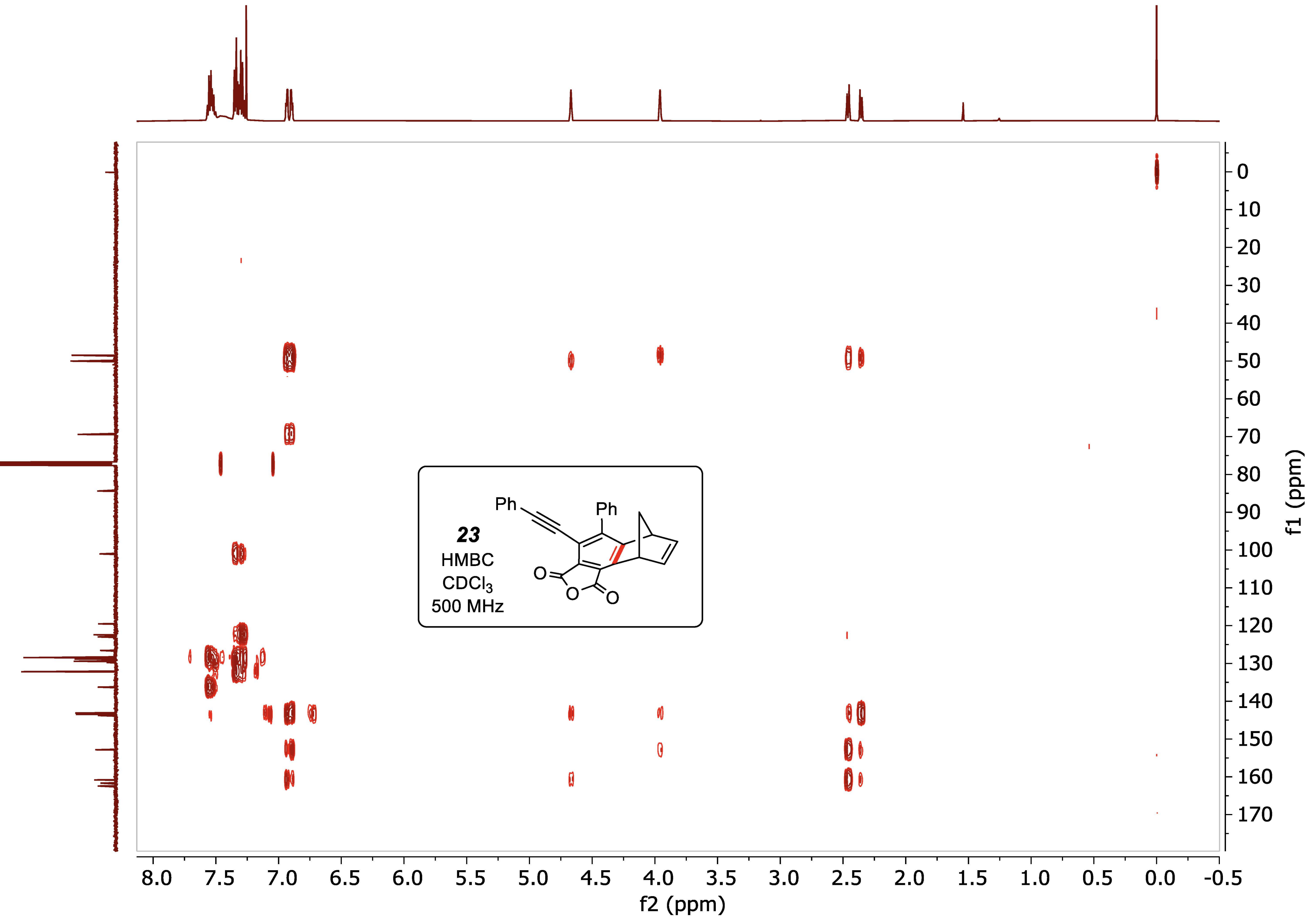
23
HMBC
$CDCl_3$
500 MHz
Ph
Ph
O
O
O
f1 (ppm)
f2 (ppm)

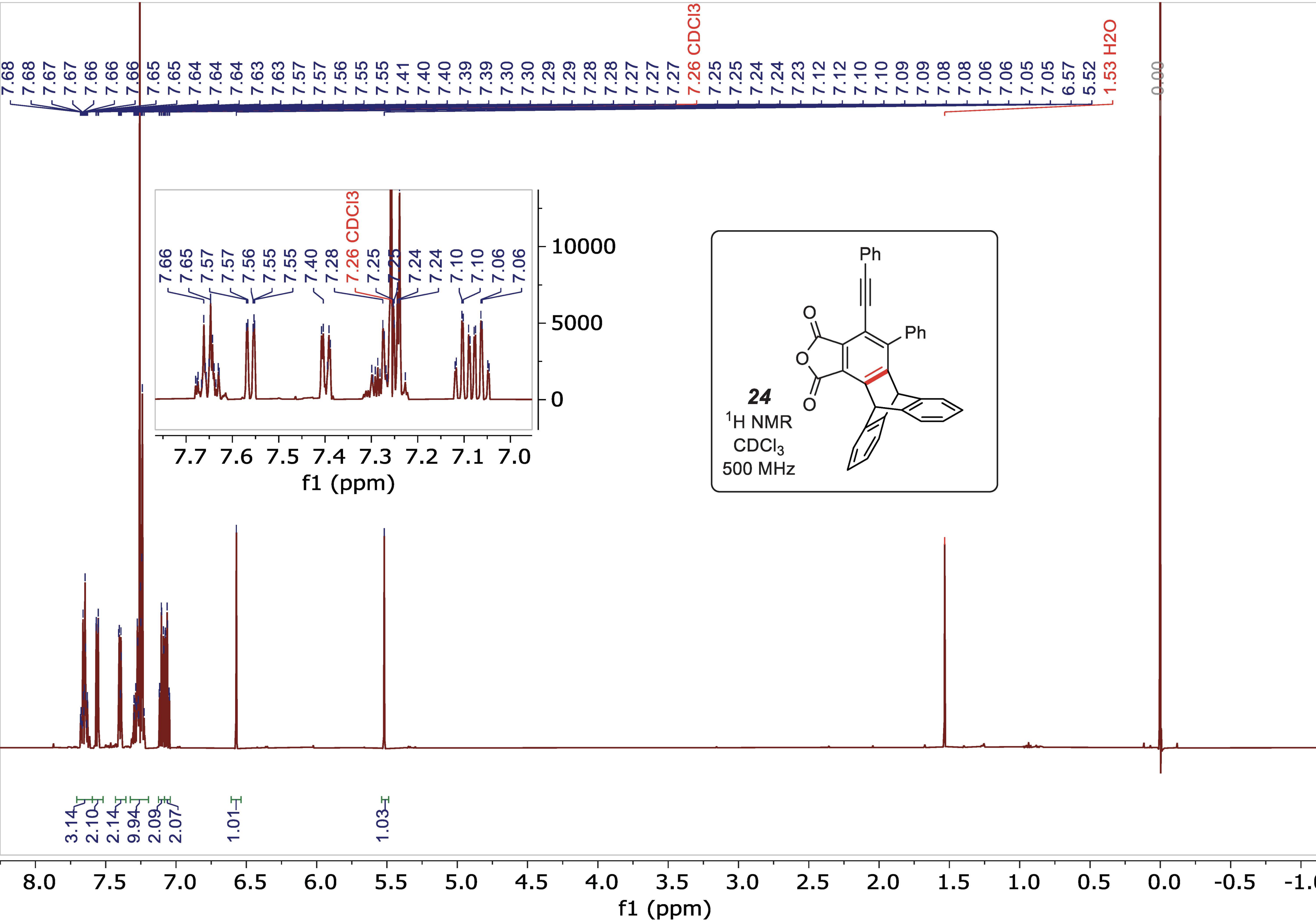
7.26 CDCl3
1.53 H2O
0.00
10000
5000
0
7.7 7.6 7.5 7.4 7.3 7.2 7.1 7.0
f1 (ppm)
Ph
O
O
O
Ph
24
1H NMR
CDCl3
500 MHz
3.14
2.10
2.14
9.94
2.09
2.07
1.01
1.03
8.0 7.5 7.0 6.5 6.0 5.5 5.0 4.5 4.0 3.5 3.0 2.5 2.0 1.5 1.0 0.5 0.0 -0.5
f1 (ppm)

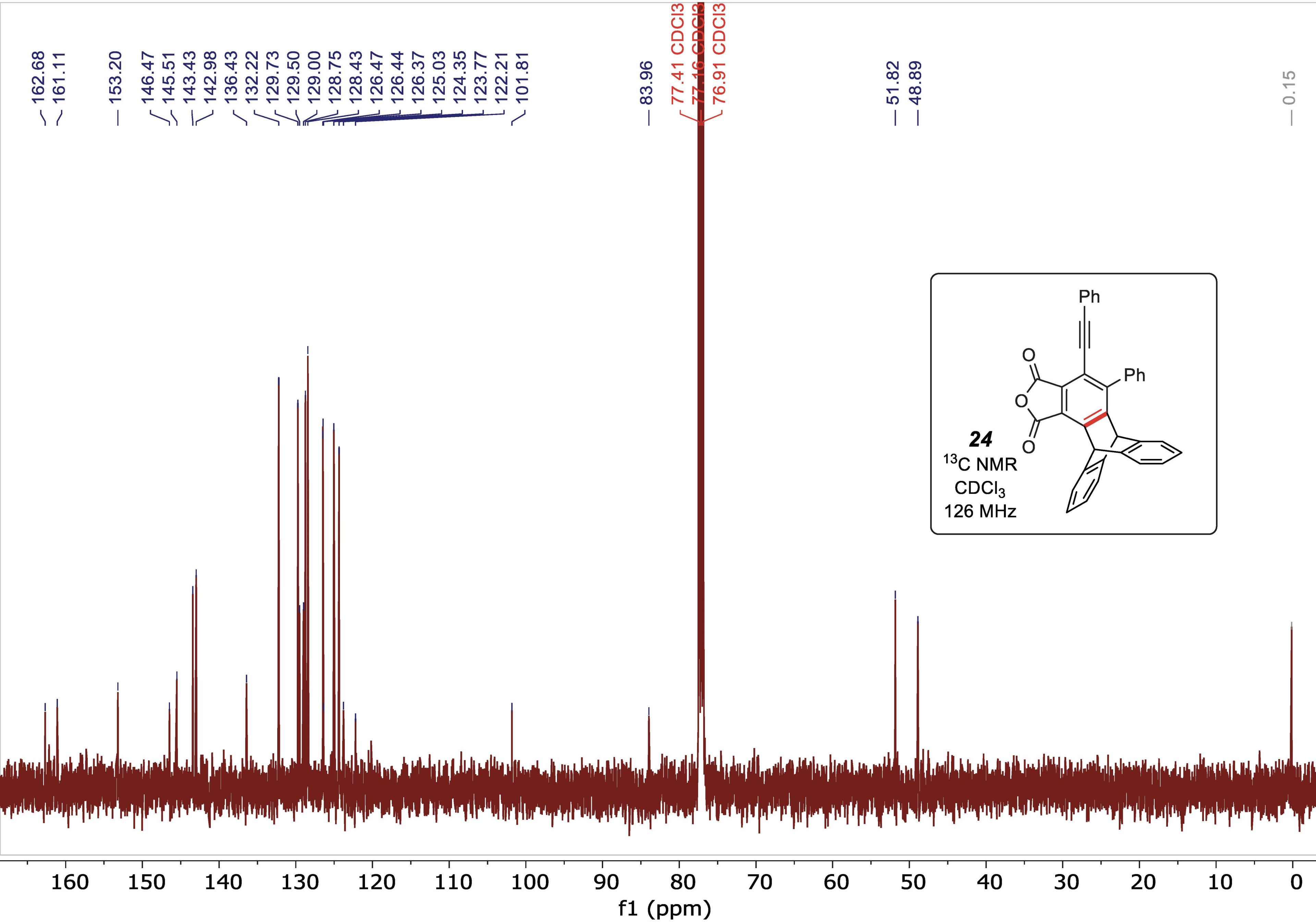
162.68
161.11
153.20
146.47
145.51
143.43
142.98
136.43
132.22
129.73
129.50
129.00
128.75
128.43
126.47
126.44
126.37
125.03
124.35
123.77
122.21
101.81
83.96
77.41 CDCl3
77.16 CDCl3
76.91 CDCl3
51.82
48.89
0.15
Ph
O
O
O
Ph
24
^{13}C NMR
$CDCl_3$
126 MHz
160 150 140 130 120 110 100 90 80 70 60 50 40 30 20 10 0
f1 (ppm)

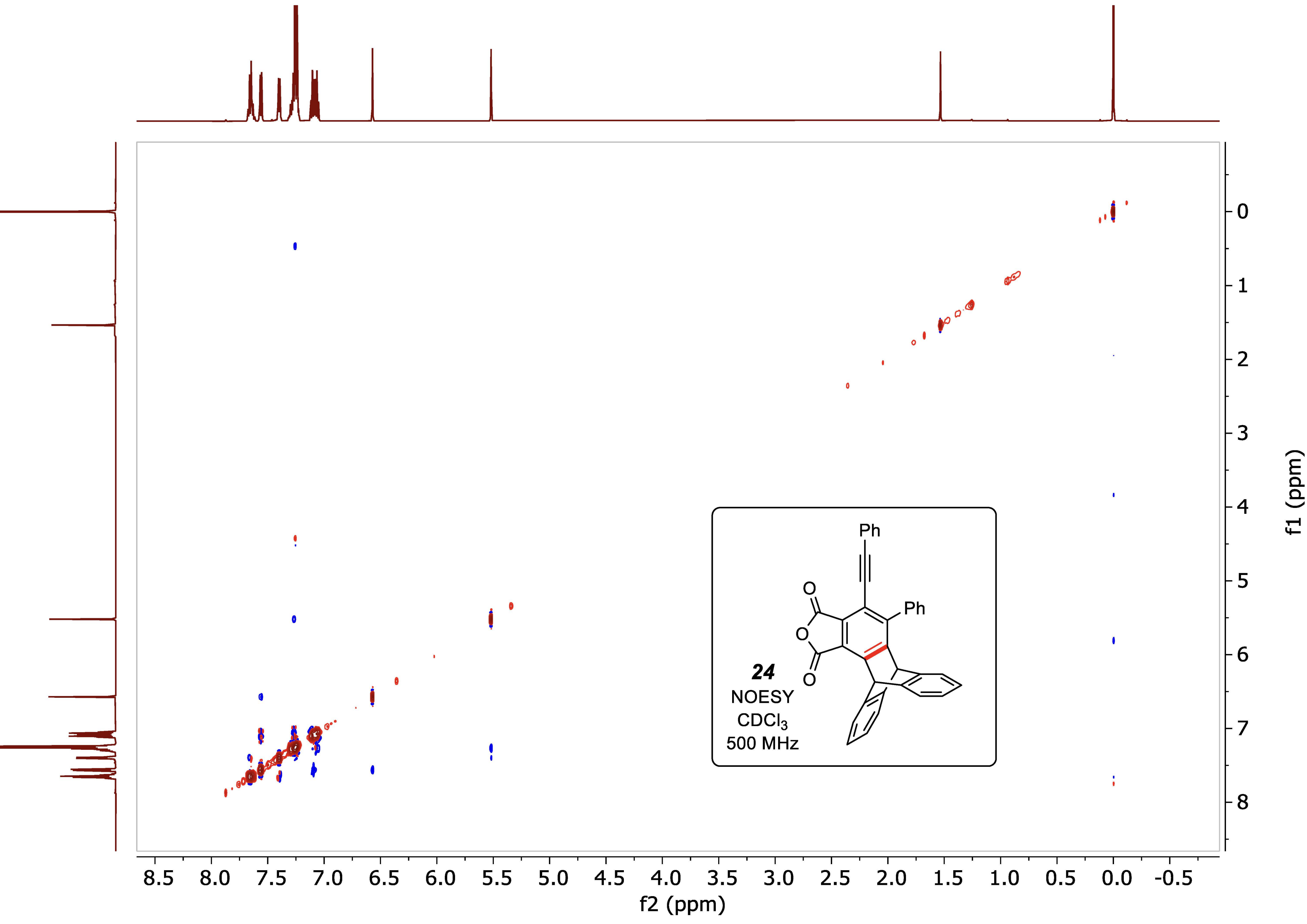
f2 (ppm)
f1 (ppm)
8.5 8.0 7.5 7.0 6.5 6.0 5.5 5.0 4.5 4.0 3.5 3.0 2.5 2.0 1.5 1.0 0.5 0.0 -0.5
0 1 2 3 4 5 6 7 8
Ph
O
Ph
O
O
24
NOESY
$CDCl_3$
500 MHz

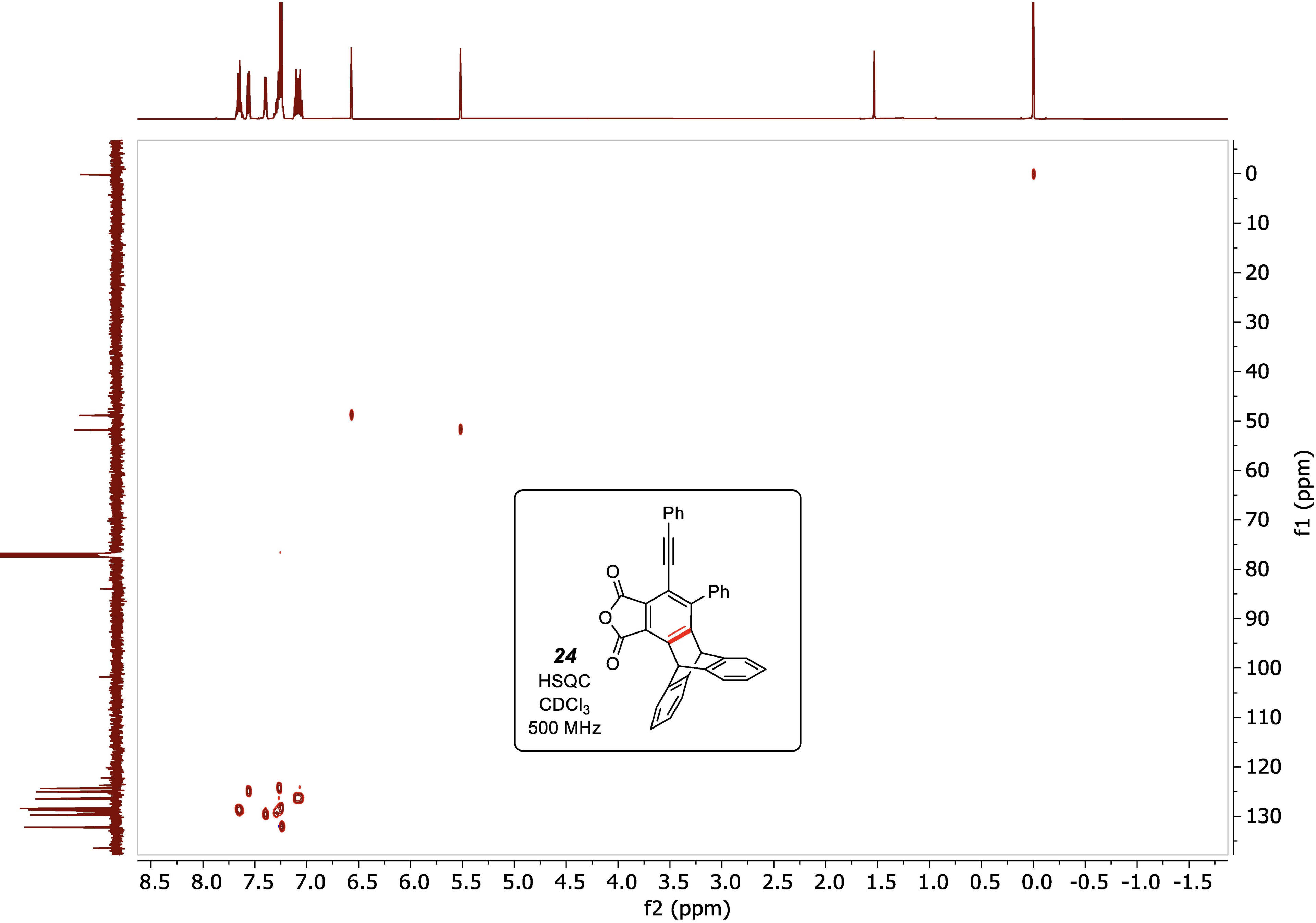
24
HSQC
$CDCl_3$
500 MHz
Ph
O
O
Ph
O
f2 (ppm)
f1 (ppm)

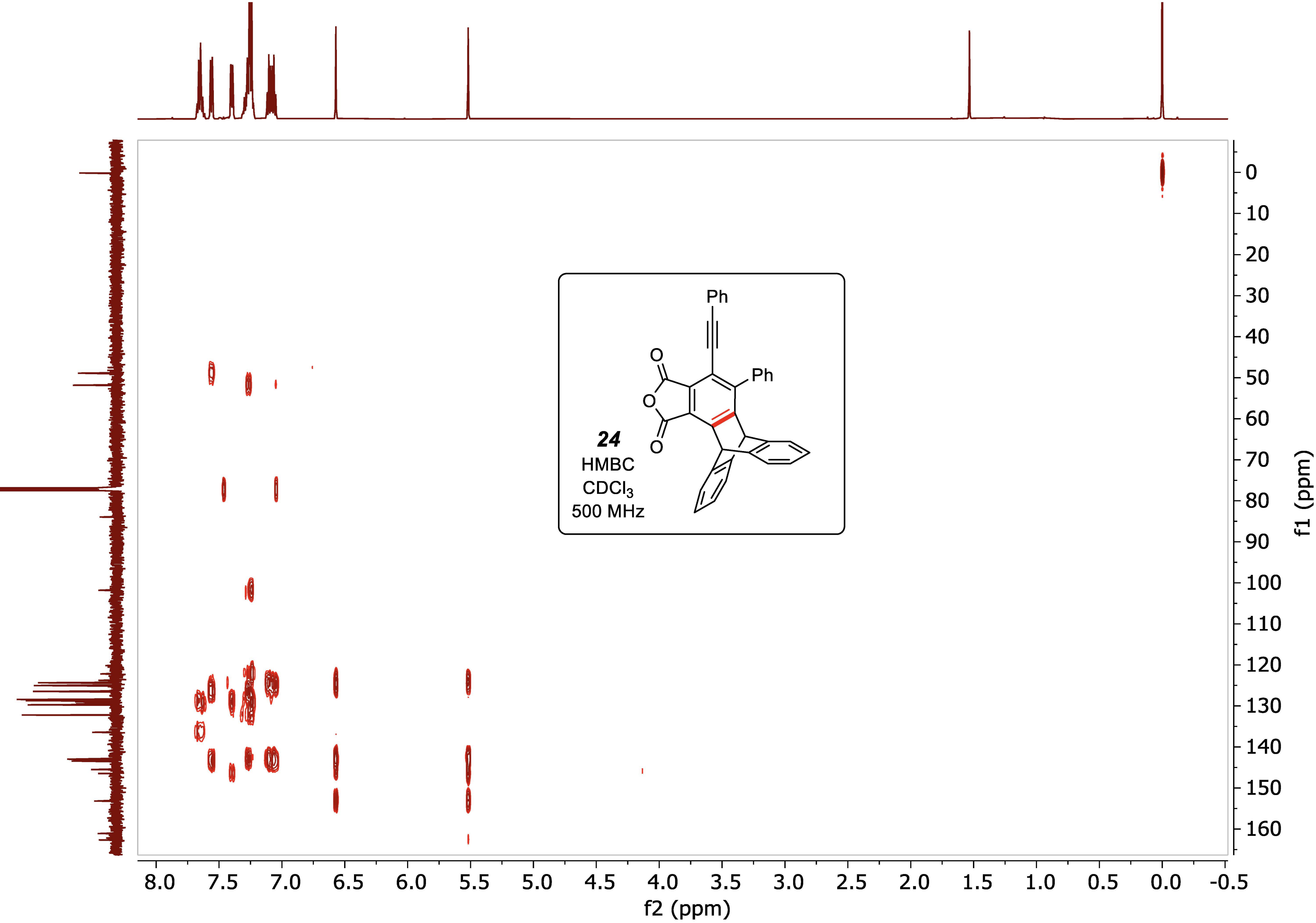
Ph
O
Ph
O
O
24
HMBC
CDCl3
500 MHz
f2 (ppm)
f1 (ppm)

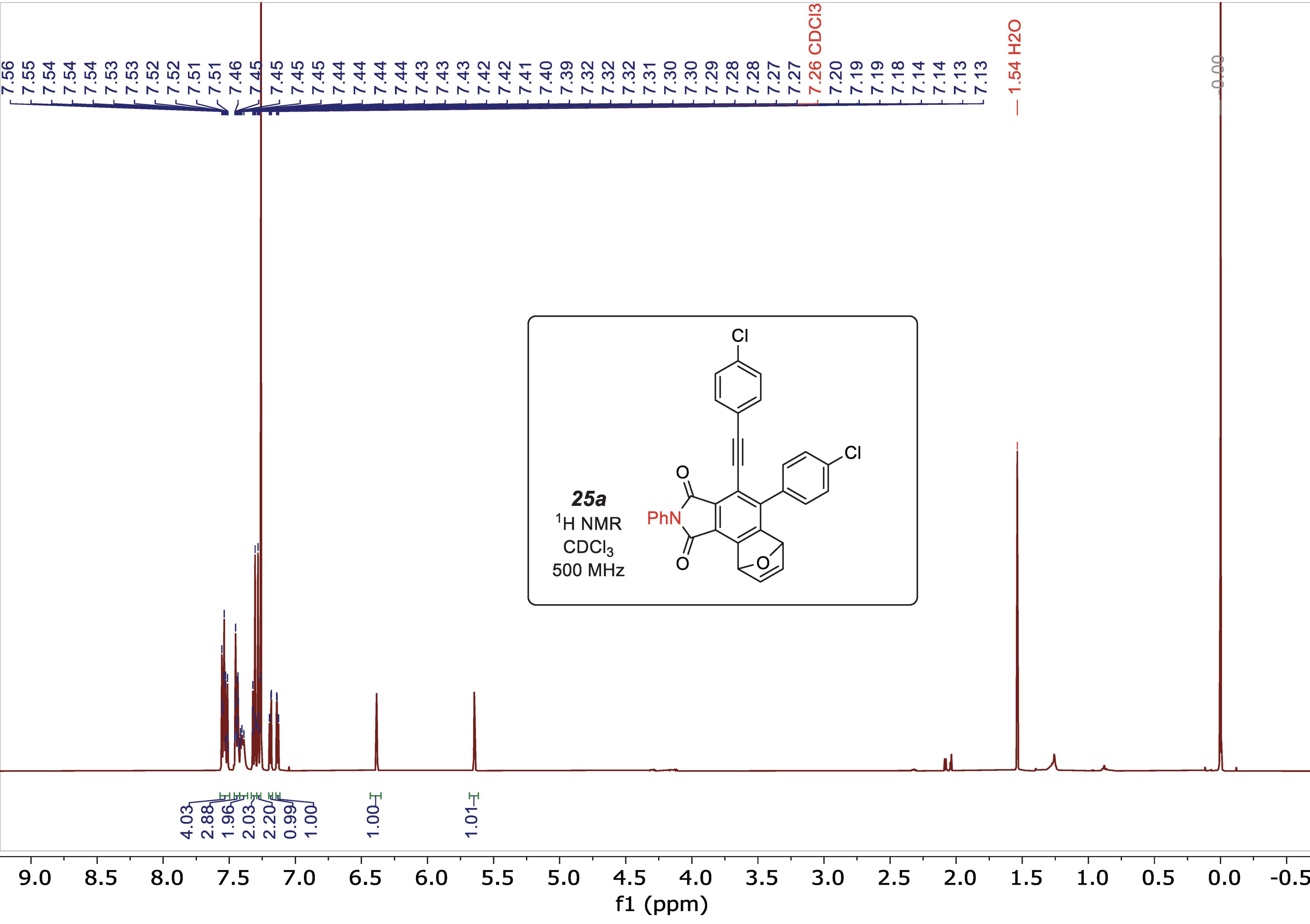
7.56 7.55 7.54 7.54 7.54 7.53 7.53 7.52 7.52 7.51 7.51 7.46 7.45 7.45 7.45 7.45 7.44 7.44 7.44 7.44 7.43 7.43 7.43 7.42 7.42 7.41 7.40 7.39 7.32 7.32 7.32 7.31 7.30 7.30 7.29 7.28 7.28 7.27 7.27
7.26 CDCl3
7.20 7.19 7.19 7.18 7.14 7.14 7.13 7.13
1.54 H2O
0.00
25a
1H NMR
CDCl3
500 MHz
PhN
O
Cl
4.03 2.88 1.96 2.03 2.20 0.99 1.00
1.00
1.01
9.0 8.5 8.0 7.5 7.0 6.5 6.0 5.5 5.0 4.5 4.0 3.5 3.0 2.5 2.0 1.5 1.0 0.5 0.0 -0.5
f1 (ppm)

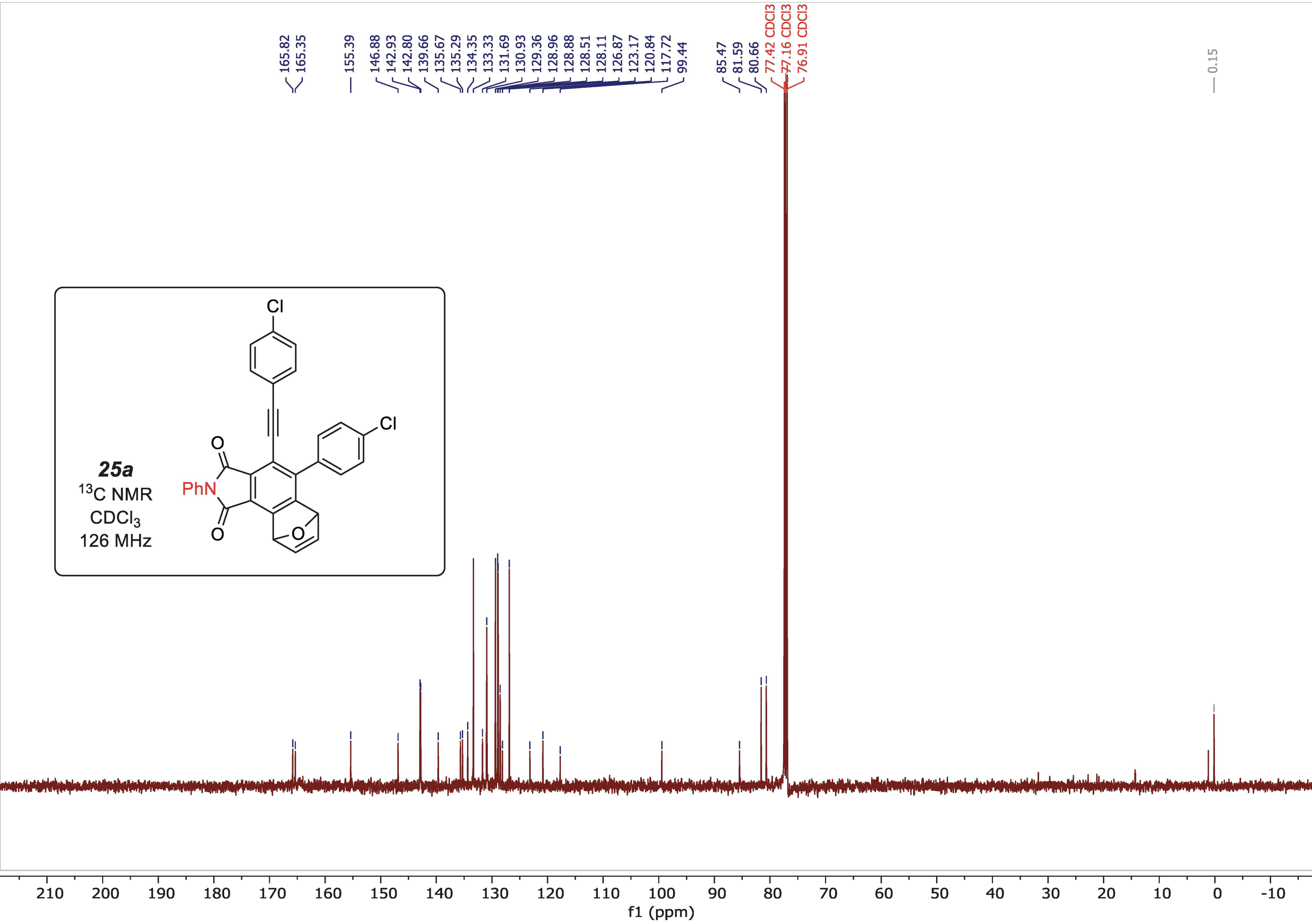
165.82
165.35
155.39
146.88
142.93
142.80
139.66
135.67
135.29
134.35
133.33
131.69
130.93
129.36
128.96
128.88
128.51
128.11
126.87
123.17
120.84
117.72
99.44
85.47
81.59
80.66
77.42 CDCl3
77.16 CDCl3
76.91 CDCl3
0.15
25a
^{13}C NMR
$CDCl_3$
126 MHz
Cl
Cl
O
O
PhN
O
210 200 190 180 170 160 150 140 130 120 110 100 90 80 70 60 50 40 30 20 10 0 -10
f1 (ppm)

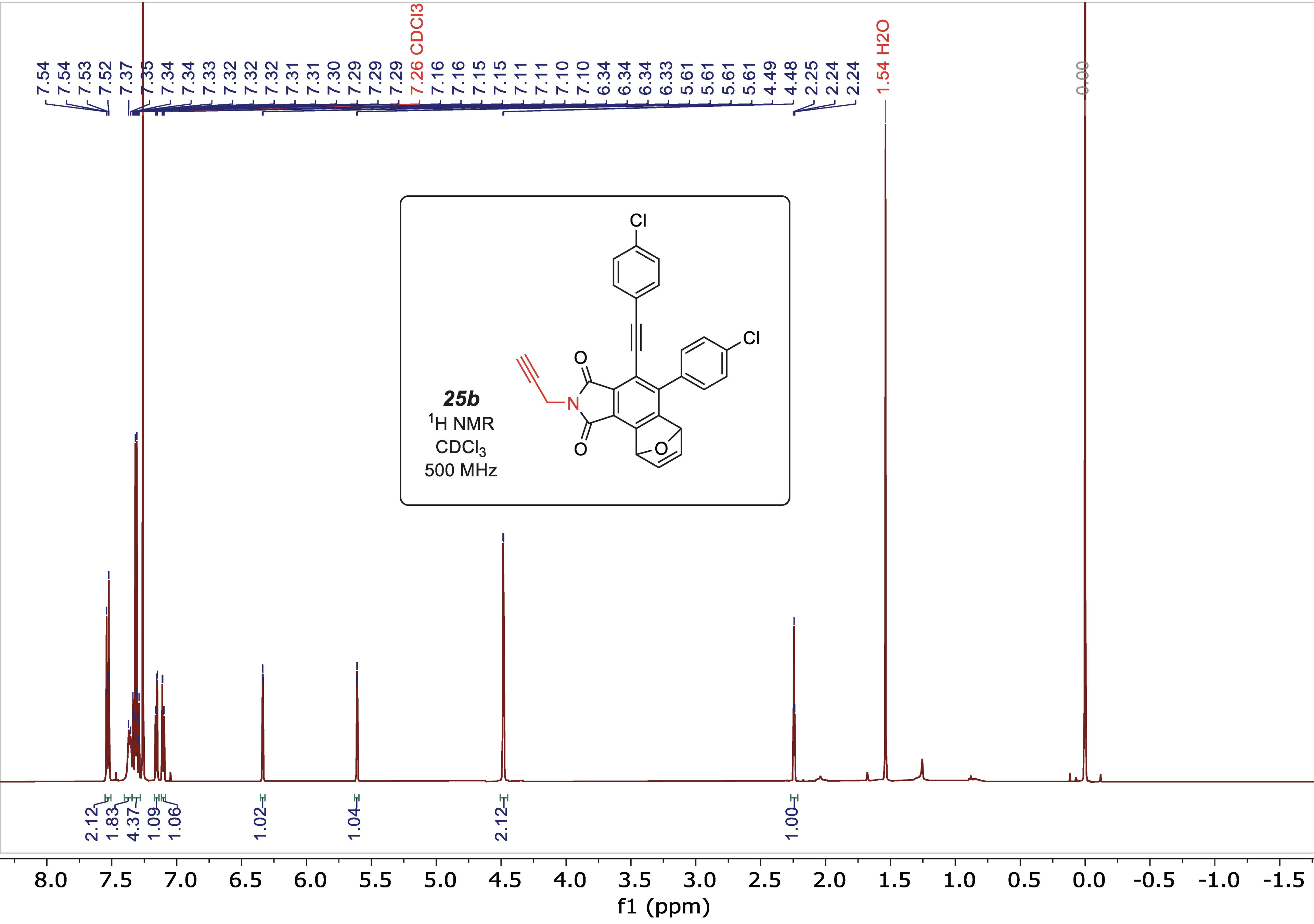
7.54 7.54 7.53 7.52 7.37 7.35 7.34 7.34 7.33 7.32 7.32 7.32 7.31 7.31 7.30 7.29 7.29 7.29 7.26 CDCl3 7.16 7.16 7.15 7.15 7.11 7.11 7.10 7.10 6.34 6.34 6.34 6.33 5.61 5.61 5.61 5.61 4.49 4.48 2.25 2.24 2.24 1.54 H2O 0.00
Cl
Cl
O
N
O
O
25b
1H NMR
CDCl3
500 MHz
2.12 1.83 4.37 1.09 1.06 1.02 1.04 2.12 1.00
8.0 7.5 7.0 6.5 6.0 5.5 5.0 4.5 4.0 3.5 3.0 2.5 2.0 1.5 1.0 0.5 0.0 -0.5 -1.0 -1.5
f1 (ppm)

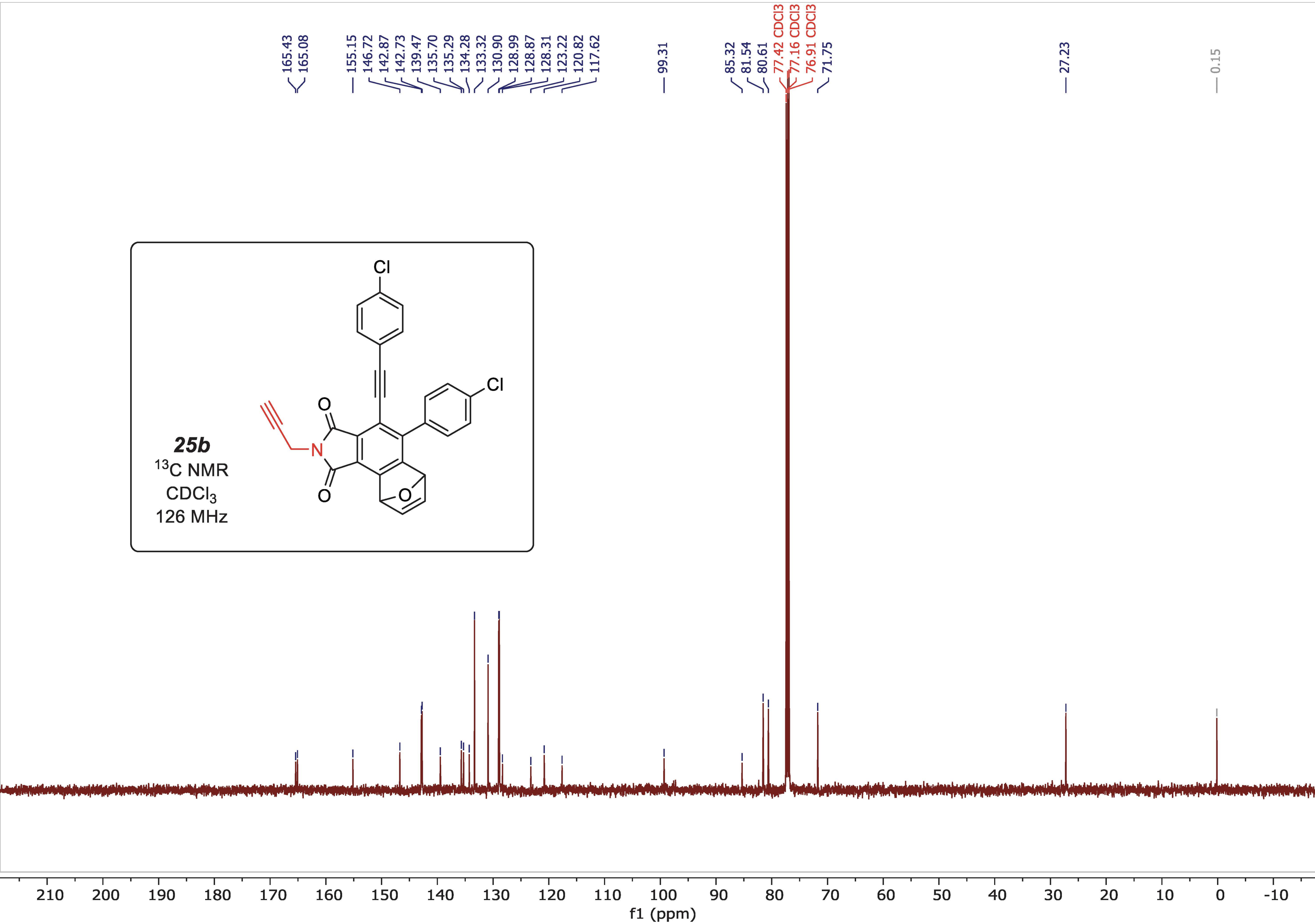
165.43
165.08
155.15
146.72
142.87
142.73
139.47
135.70
135.29
134.28
133.32
130.90
128.99
128.87
128.31
123.22
120.82
117.62
99.31
85.32
81.54
80.61
77.42 CDCl3
77.16 CDCl3
76.91 CDCl3
71.75
27.23
0.15
Cl
Cl
O
O
N
O
25b
13C NMR
CDCl3
126 MHz
210
200
190
180
170
160
150
140
130
120
110
100
90
80
70
60
50
40
30
20
10
0
-10
f1 (ppm)

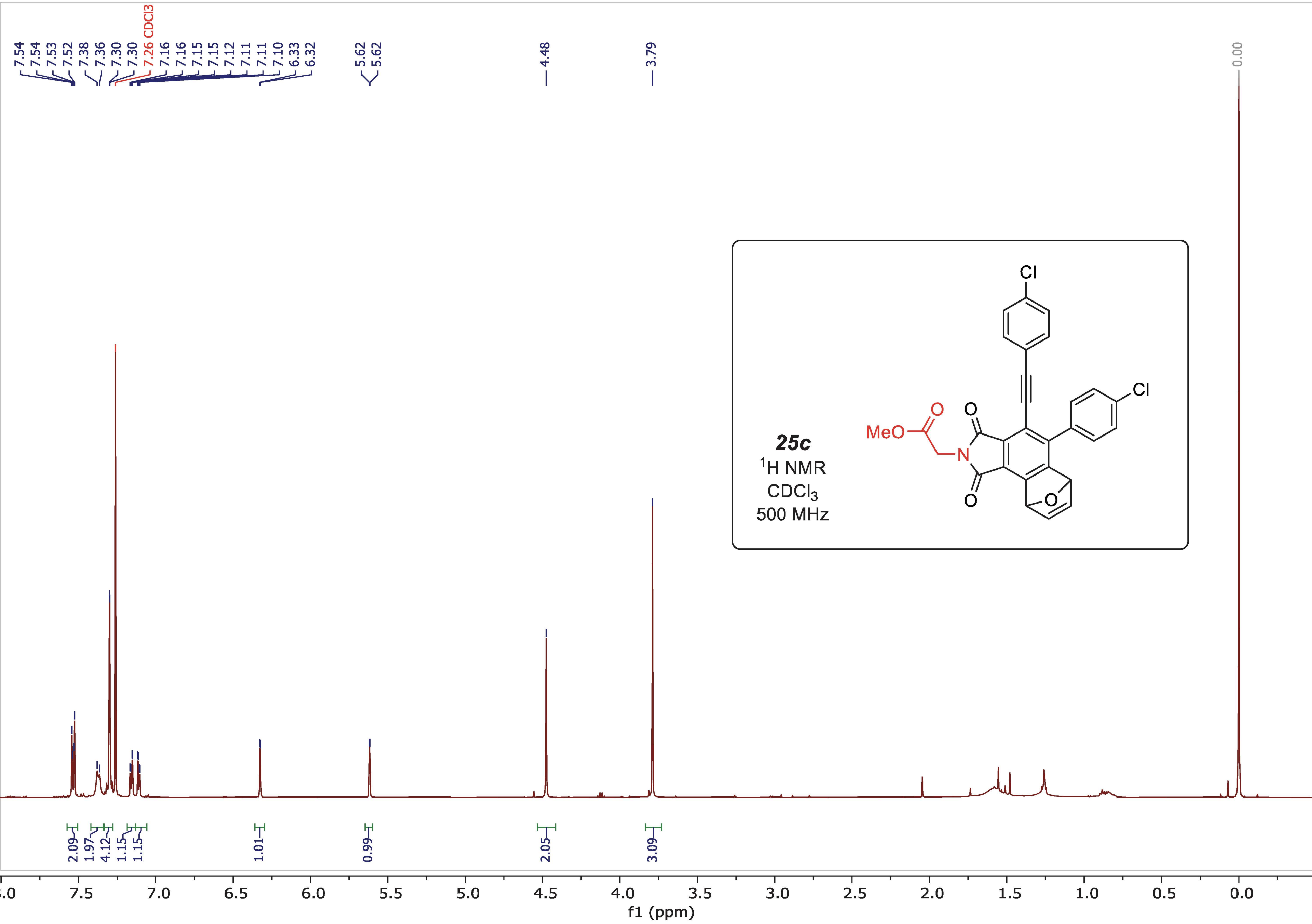
7.54
7.54
7.53
7.52
7.38
7.36
7.30
7.30
7.26 CDCl3
7.16
7.16
7.15
7.15
7.12
7.11
7.11
7.10
6.33
6.32
5.62
5.62
4.48
3.79
0.00
25c
1H NMR
CDCl3
500 MHz
MeO
O
O
N
O
Cl
Cl
O
2.09
1.97
4.12
1.15
1.15
1.01
0.99
2.05
3.09
7.5
7.0
6.5
6.0
5.5
5.0
4.5
4.0
3.5
3.0
2.5
2.0
1.5
1.0
0.5
0.0
f1 (ppm)

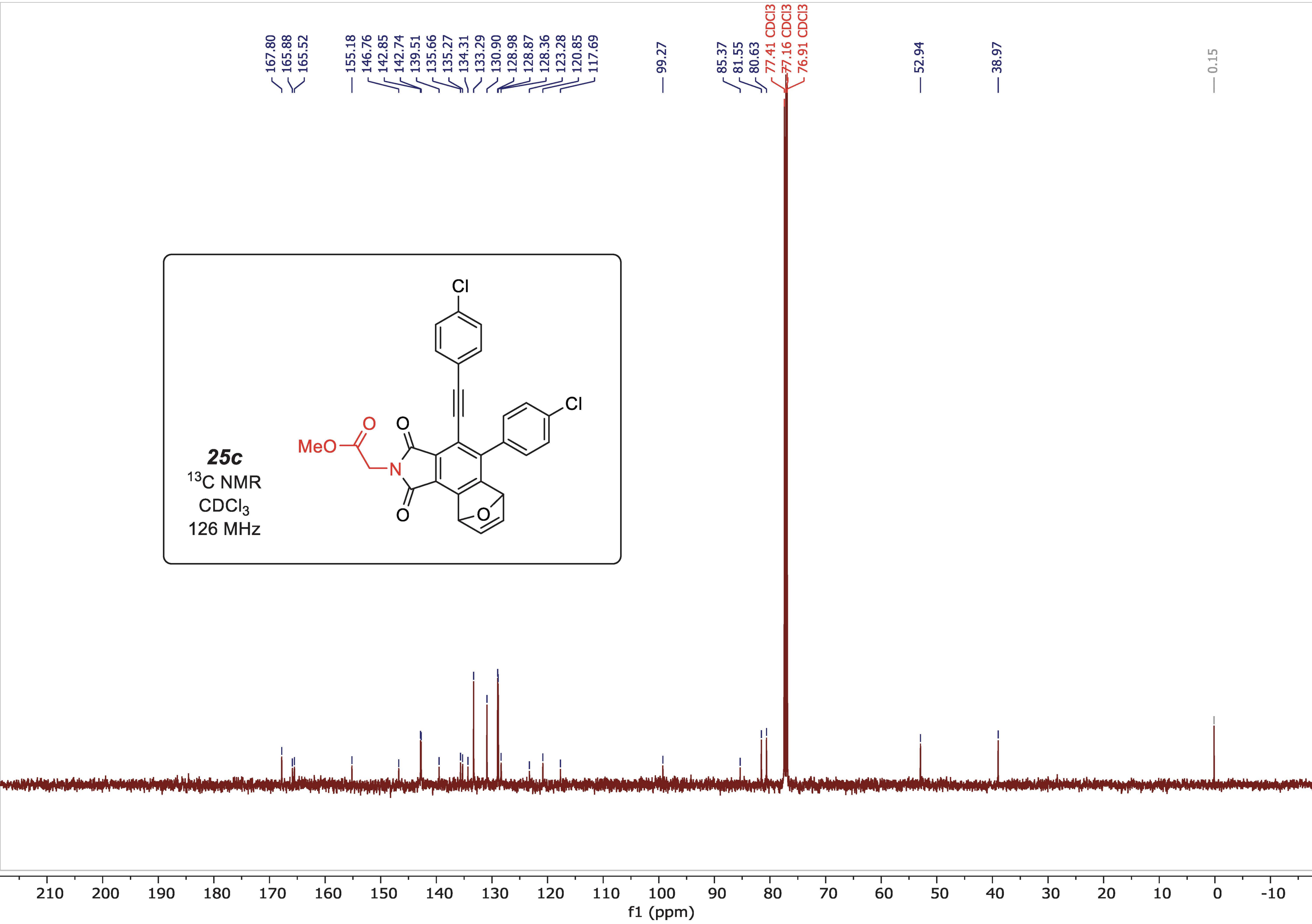
167.80
165.88
165.52
155.18
146.76
142.85
142.74
139.51
135.66
135.27
134.31
133.29
130.90
128.98
128.87
128.36
123.28
120.85
117.69
99.27
85.37
81.55
80.63
77.41 CDCl3
77.16 CDCl3
76.91 CDCl3
52.94
38.97
0.15
Cl
Cl
MeO
O
O
N
O
O
25c
^{13}C NMR
CDCl$_3$
126 MHz
f1 (ppm)

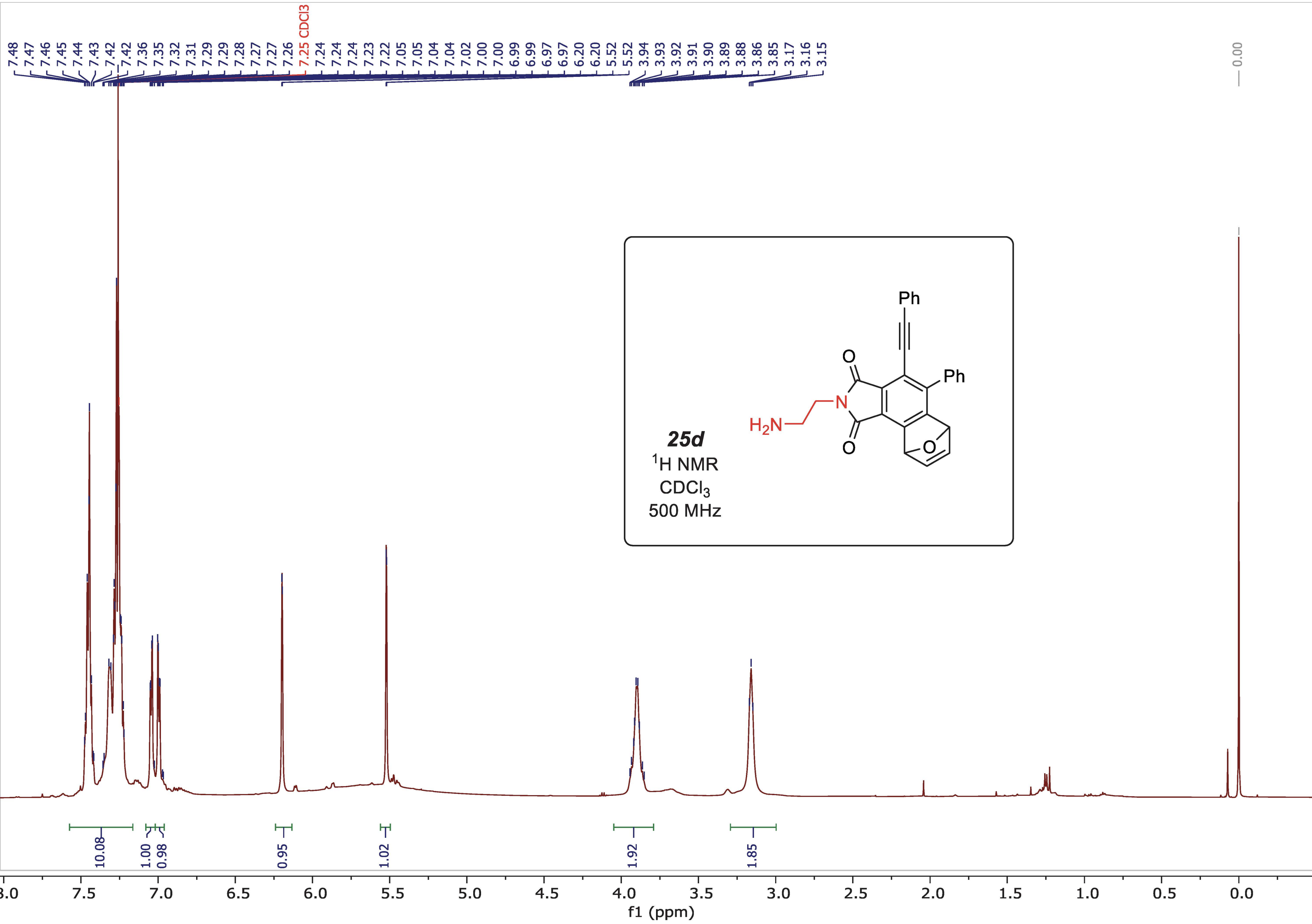
7.48 7.47 7.46 7.45 7.44 7.43 7.42 7.42 7.36 7.35 7.32 7.31 7.29 7.29 7.28 7.27 7.27 7.26 7.25 CDCl3 7.24 7.24 7.24 7.23 7.22 7.05 7.05 7.04 7.04 7.02 7.00 7.00 6.99 6.99 6.97 6.97 6.20 6.20 5.52 5.52 3.94 3.93 3.92 3.91 3.90 3.89 3.88 3.86 3.85 3.17 3.16 3.15
0.00
Ph
O
Ph
H_2N
N
O
O
25d
^1H NMR
$CDCl_3$
500 MHz
10.08
1.00
0.98
0.95
1.02
1.92
1.85
8.0 7.5 7.0 6.5 6.0 5.5 5.0 4.5 4.0 3.5 3.0 2.5 2.0 1.5 1.0 0.5 0.0
f1 (ppm)

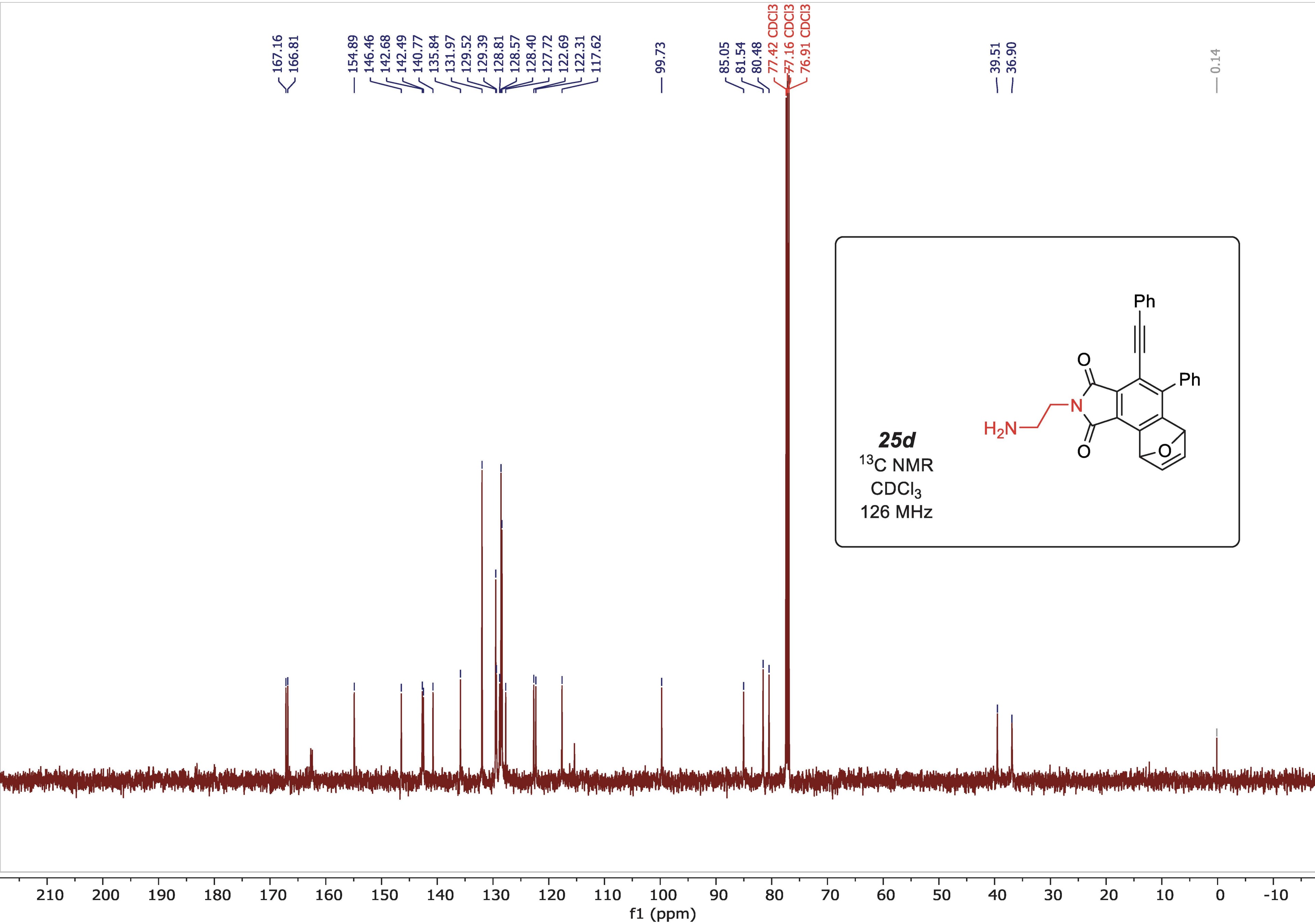
167.16
166.81
154.89
146.46
142.68
142.49
140.77
135.84
131.97
129.52
129.39
128.81
128.57
128.40
127.72
122.69
122.31
117.62
99.73
85.05
81.54
80.48
77.42 CDCl3
77.16 CDCl3
76.91 CDCl3
39.51
36.90
0.14
Ph
O
Ph
H_2N
N
O
O
25d
^{13}C NMR
$CDCl_3$
126 MHz
210 200 190 180 170 160 150 140 130 120 110 100 90 80 70 60 50 40 30 20 10 0 -10
f1 (ppm)

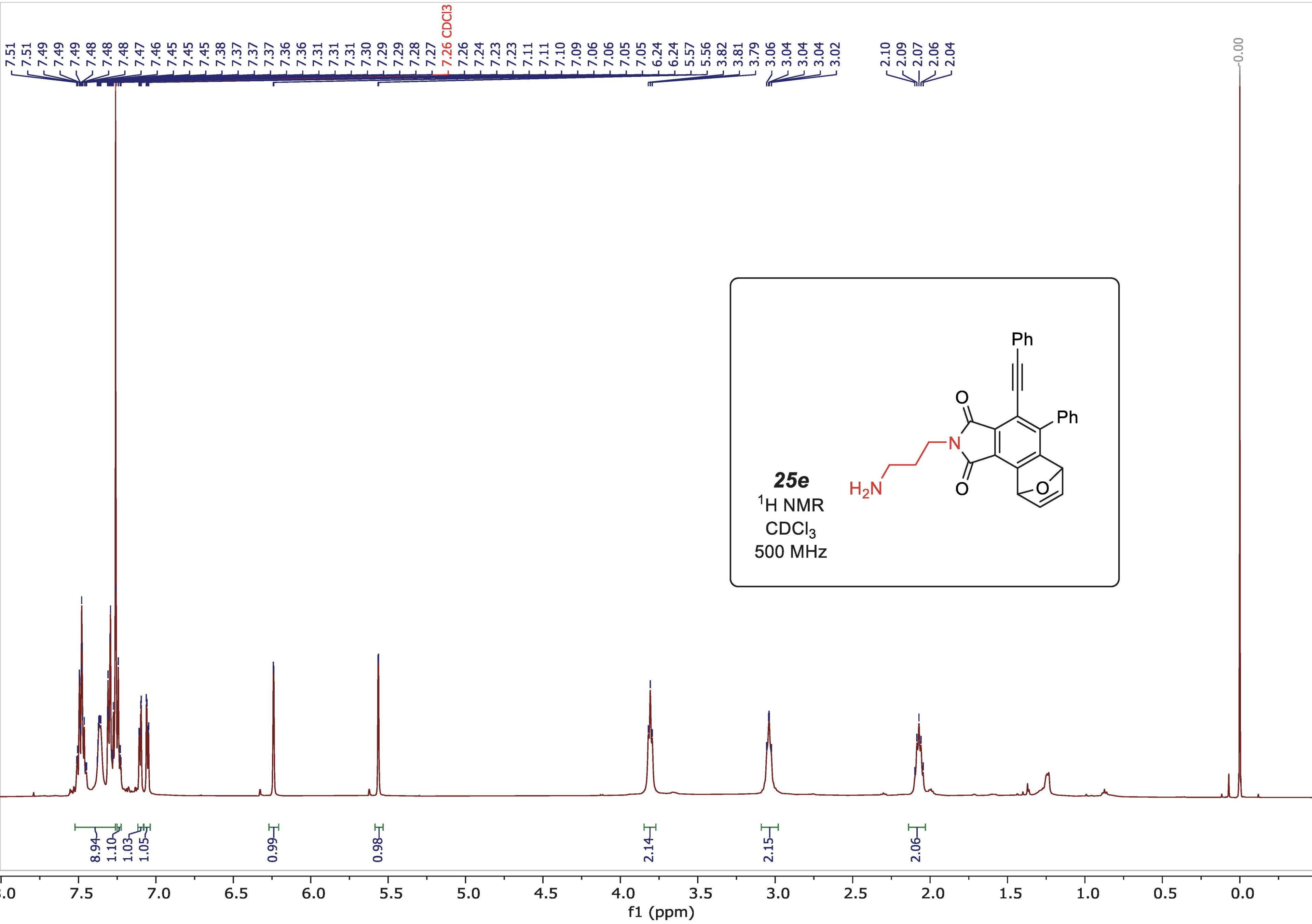
7.51 7.51 7.49 7.49 7.49 7.48 7.48 7.48 7.47 7.46 7.45 7.45 7.45 7.38 7.37 7.37 7.37 7.36 7.36 7.31 7.31 7.31 7.30 7.29 7.29 7.28 7.27 7.26 CDCl3 7.26 7.24 7.23 7.23 7.23 7.11 7.11 7.10 7.09 7.06 7.06 7.05 7.05 6.24 6.24 5.57 5.56 3.82 3.81 3.79 3.06 3.04 3.04 3.04 3.02 2.10 2.09 2.07 2.06 2.04 0.00
Ph
O
Ph
N
H_2N
O
O
25e
^{1}H NMR
$CDCl_3$
500 MHz
8.94 1.10 1.03 1.05 0.99 0.98 2.14 2.15 2.06
7.5 7.0 6.5 6.0 5.5 5.0 4.5 4.0 3.5 3.0 2.5 2.0 1.5 1.0 0.5 0.0
f1 (ppm)

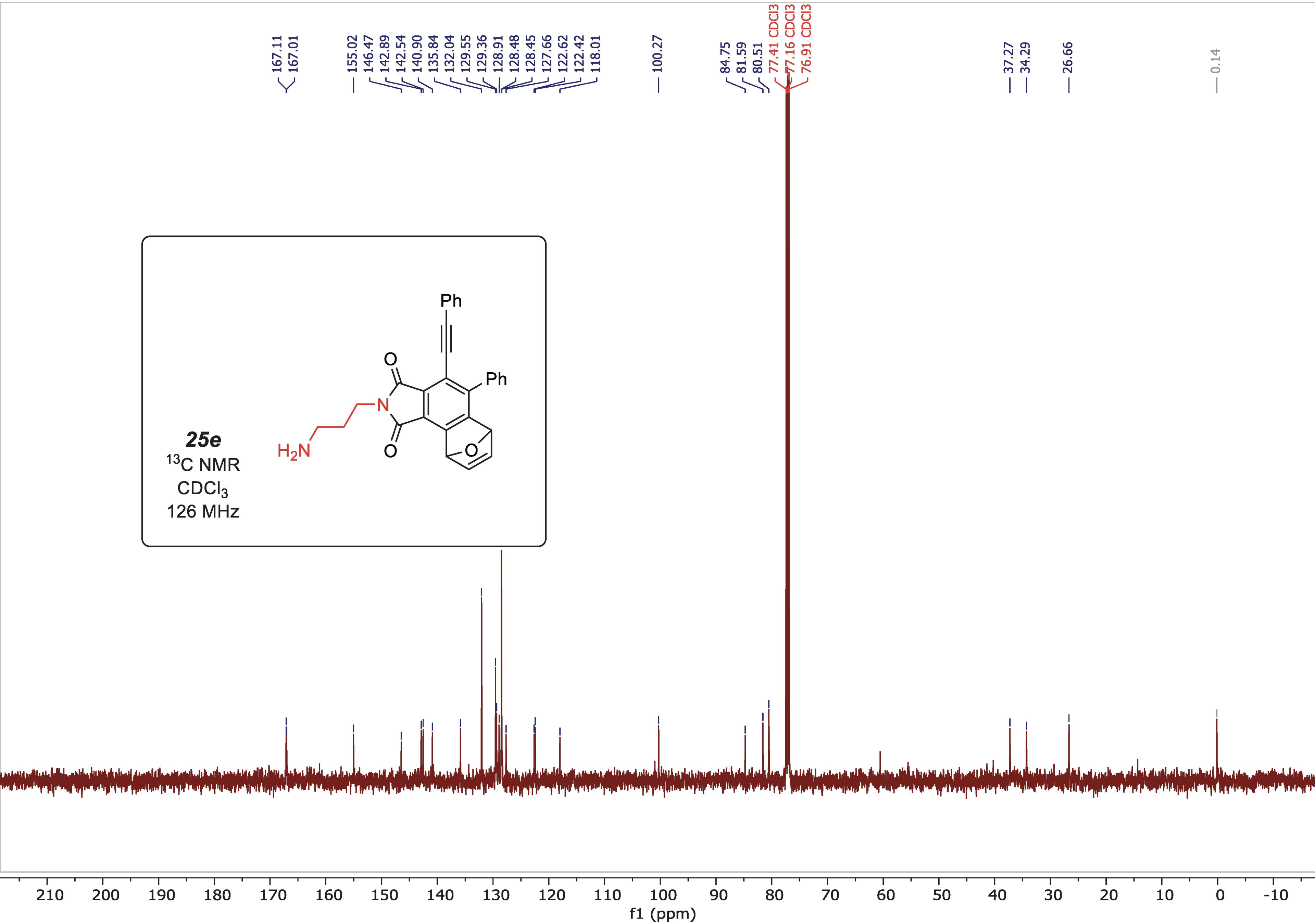
167.11
167.01
155.02
146.47
142.89
142.54
140.90
135.84
132.04
129.55
129.36
128.91
128.48
128.45
127.66
122.62
122.42
118.01
100.27
84.75
81.59
80.51
77.41 CDCl3
77.16 CDCl3
76.91 CDCl3
37.27
34.29
26.66
0.14
Ph
O
Ph
N
H_2N
O
O
25e
^{13}C NMR
$CDCl_3$
126 MHz
210 200 190 180 170 160 150 140 130 120 110 100 90 80 70 60 50 40 30 20 10 0 -10
f1 (ppm)

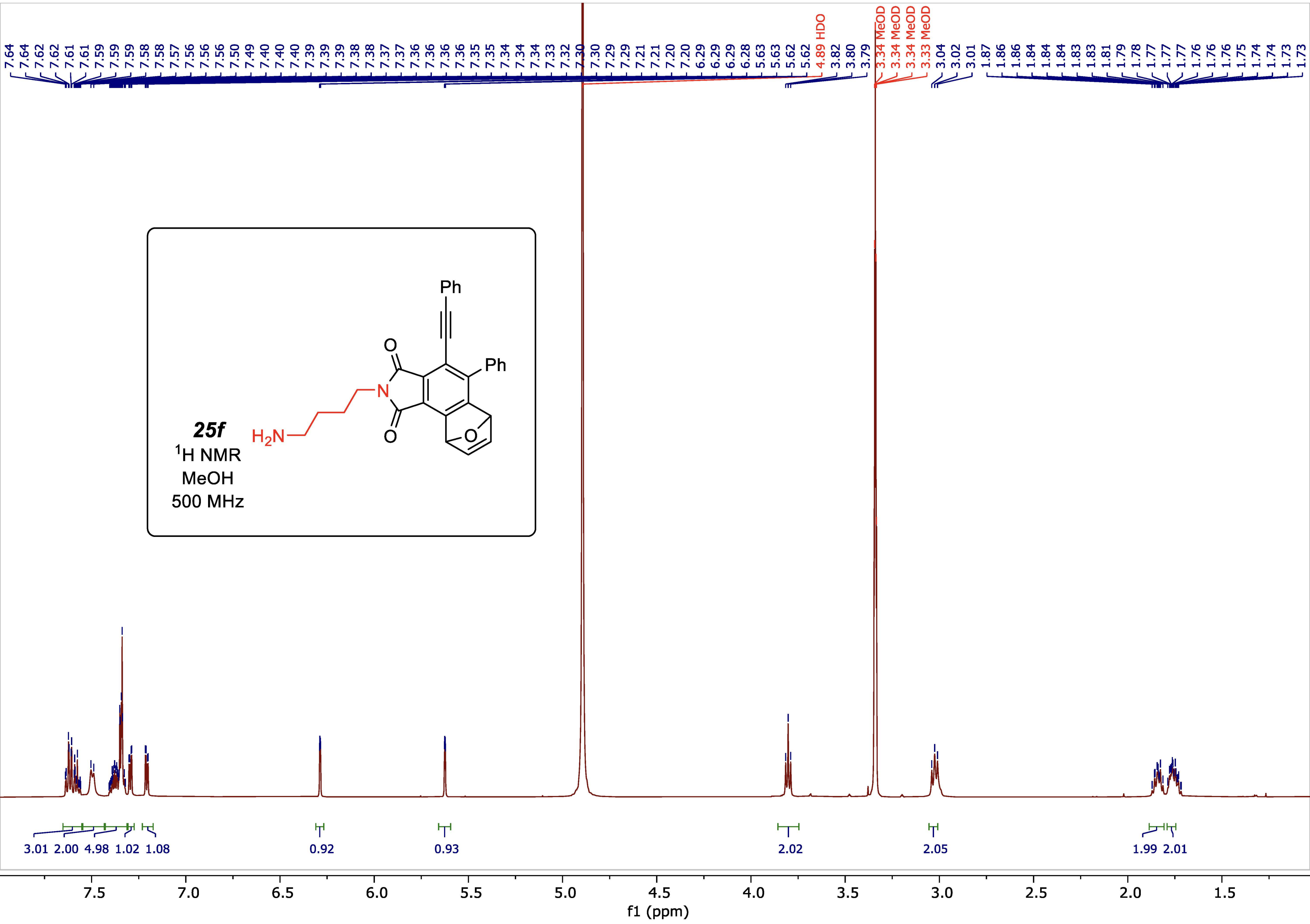
7.64 7.64 7.62 7.62 7.61 7.61 7.59 7.59 7.59 7.58 7.58 7.57 7.56 7.56 7.56 7.50 7.49 7.40 7.40 7.40 7.39 7.39 7.39 7.38 7.38 7.37 7.37 7.36 7.36 7.36 7.36 7.35 7.35 7.34 7.34 7.34 7.33 7.32 7.30 7.30 7.29 7.29 7.21 7.21 7.20 7.20 6.29 6.29 6.29 6.28 5.63 5.63 5.62 5.62
4.89 HDO
3.82 3.80 3.79
3.34 MeOD 3.34 MeOD 3.34 MeOD 3.33 MeOD
3.04 3.02 3.01 1.87 1.86 1.86 1.84 1.84 1.84 1.83 1.83 1.81 1.79 1.78 1.77 1.77 1.77 1.76 1.76 1.76 1.75 1.74 1.74 1.73 1.73
Ph
O
Ph
N
H2N
O
O
25f
1H NMR
MeOH
500 MHz
3.01 2.00 4.98 1.02 1.08 0.92 0.93 2.02 2.05 1.99 2.01
7.5 7.0 6.5 6.0 5.5 5.0 4.5 4.0 3.5 3.0 2.5 2.0 1.5
f1 (ppm)

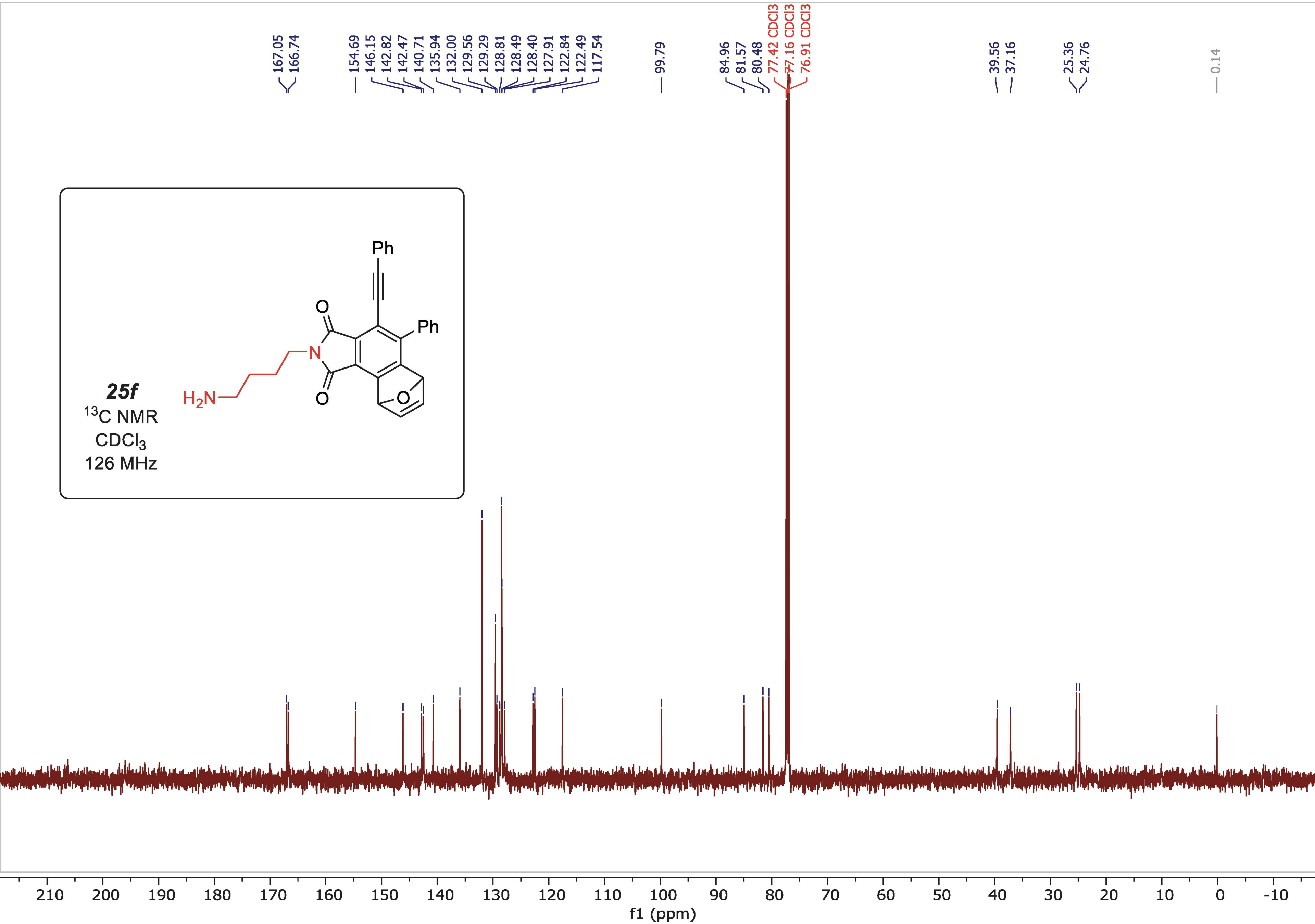
167.05
166.74
154.69
146.15
142.82
142.47
140.71
135.94
132.00
129.56
129.29
128.81
128.49
128.40
127.91
122.84
122.49
117.54
99.79
84.96
81.57
80.48
77.42 CDCl3
77.16 CDCl3
76.91 CDCl3
39.56
37.16
25.36
24.76
0.14
Ph
O
Ph
N
H_2N
O
O
25f
^{13}C NMR
$CDCl_3$
126 MHz
f1 (ppm)

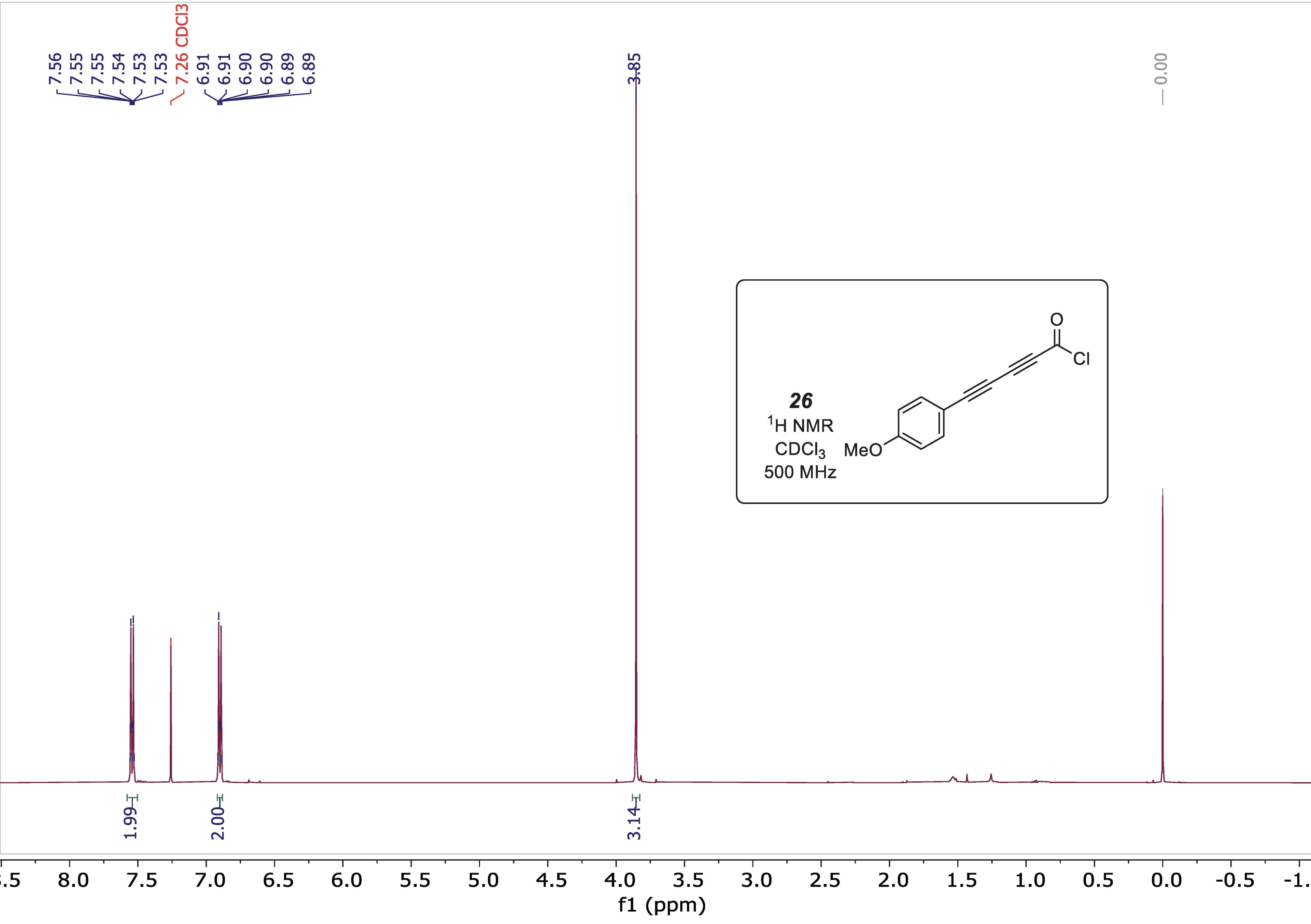
7.56
7.55
7.55
7.54
7.53
7.53
7.26 CDCl3
6.91
6.91
6.90
6.90
6.89
6.89
3.85
0.00
26
1H NMR
CDCl3
500 MHz
MeO
O
Cl
1.99
2.00
3.14
8.0 7.5 7.0 6.5 6.0 5.5 5.0 4.5 4.0 3.5 3.0 2.5 2.0 1.5 1.0 0.5 0.0 -0.5
f1 (ppm)

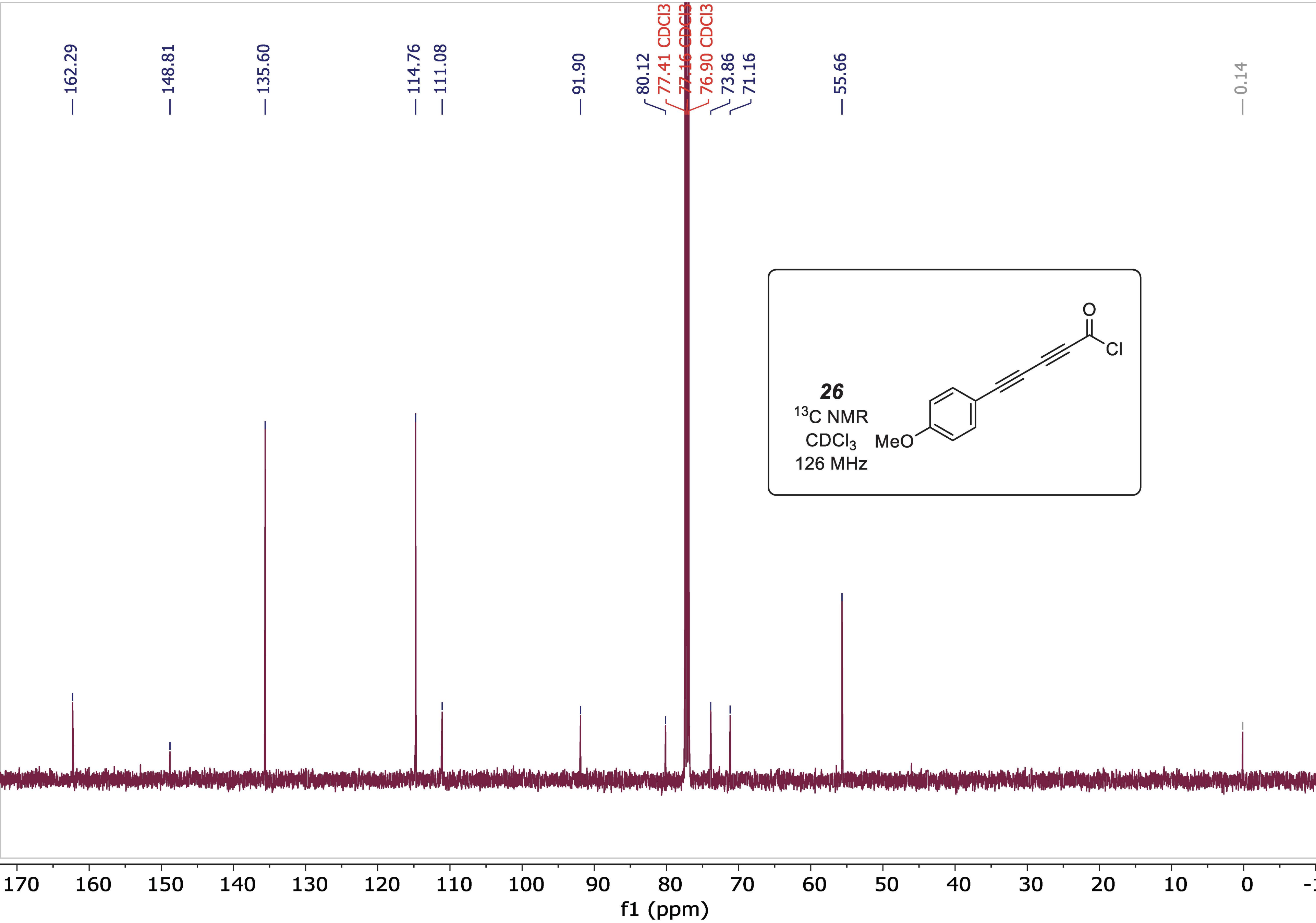
162.29
148.81
135.60
114.76
111.08
91.90
80.12
77.41 CDCl3
77.16 CDCl3
76.90 CDCl3
73.86
71.16
55.66
0.14
26
13C NMR
CDCl3
126 MHz
MeO
O
Cl
170
160
150
140
130
120
110
100
90
80
70
60
50
40
30
20
10
0
f1 (ppm)

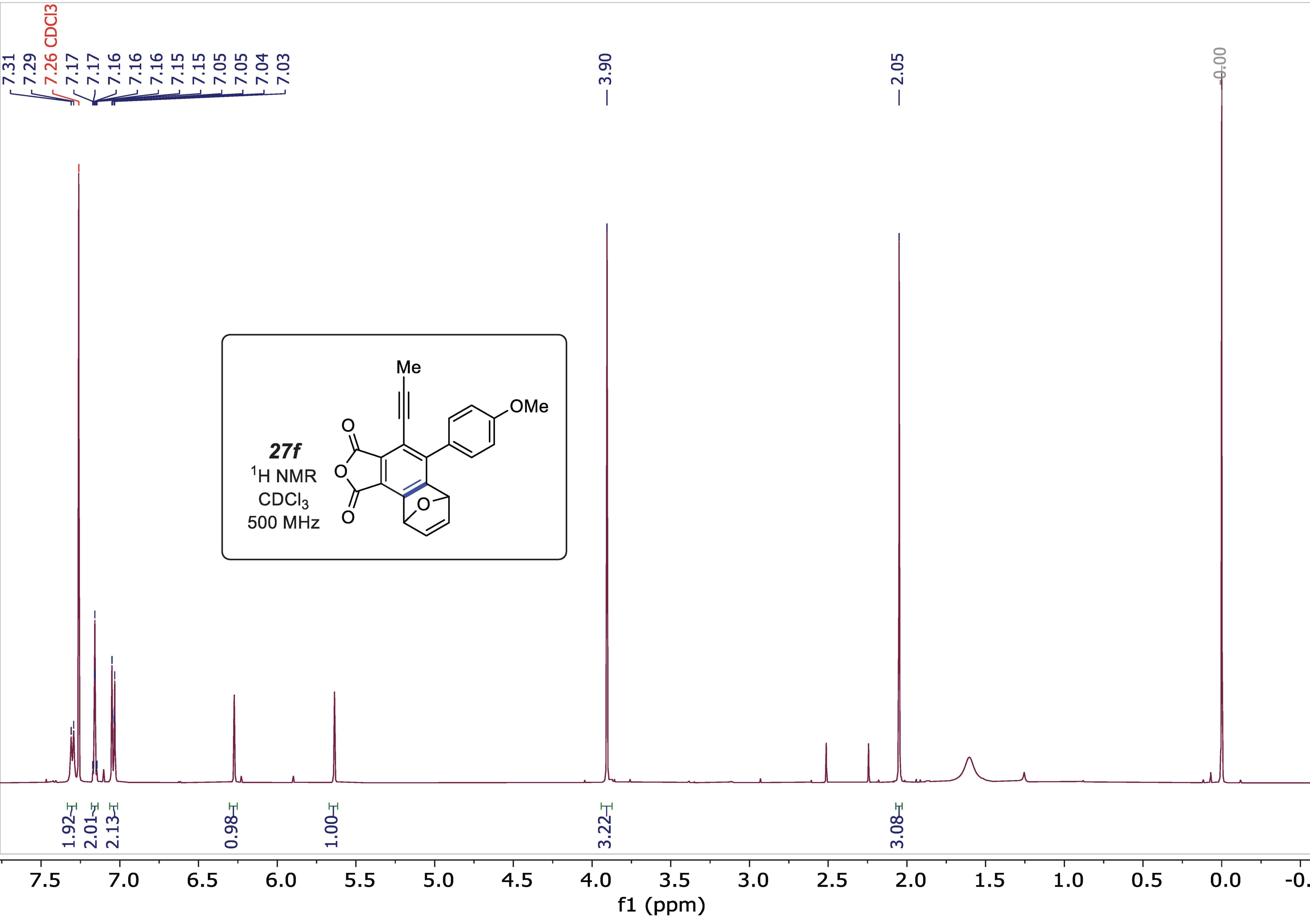
7.31
7.29
7.26 CDCl3
7.17
7.17
7.16
7.16
7.16
7.15
7.15
7.05
7.05
7.04
7.03
3.90
2.05
-0.00
27f
1H NMR
CDCl3
500 MHz
Me
OMe
O
1.92
2.01
2.13
0.98
1.00
3.22
3.08
7.5
7.0
6.5
6.0
5.5
5.0
4.5
4.0
3.5
3.0
2.5
2.0
1.5
1.0
0.5
0.0
f1 (ppm)

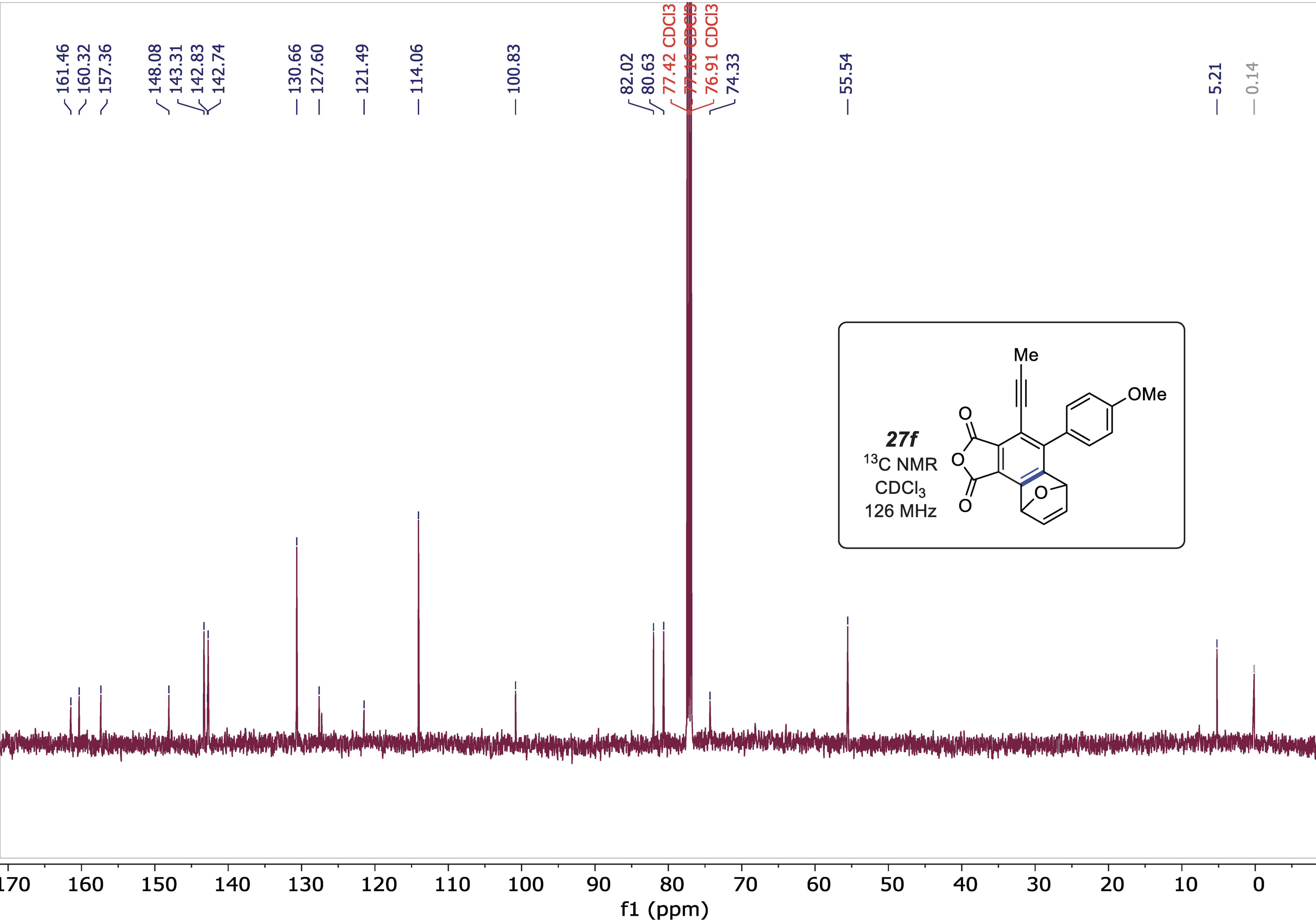
161.46
160.32
157.36
148.08
143.31
142.83
142.74
130.66
127.60
121.49
114.06
100.83
82.02
80.63
77.42 CDCl3
77.16 CDCl3
76.91 CDCl3
74.33
55.54
5.21
0.14
27f
13C NMR
CDCl3
126 MHz
Me
OMe
O
f1 (ppm)

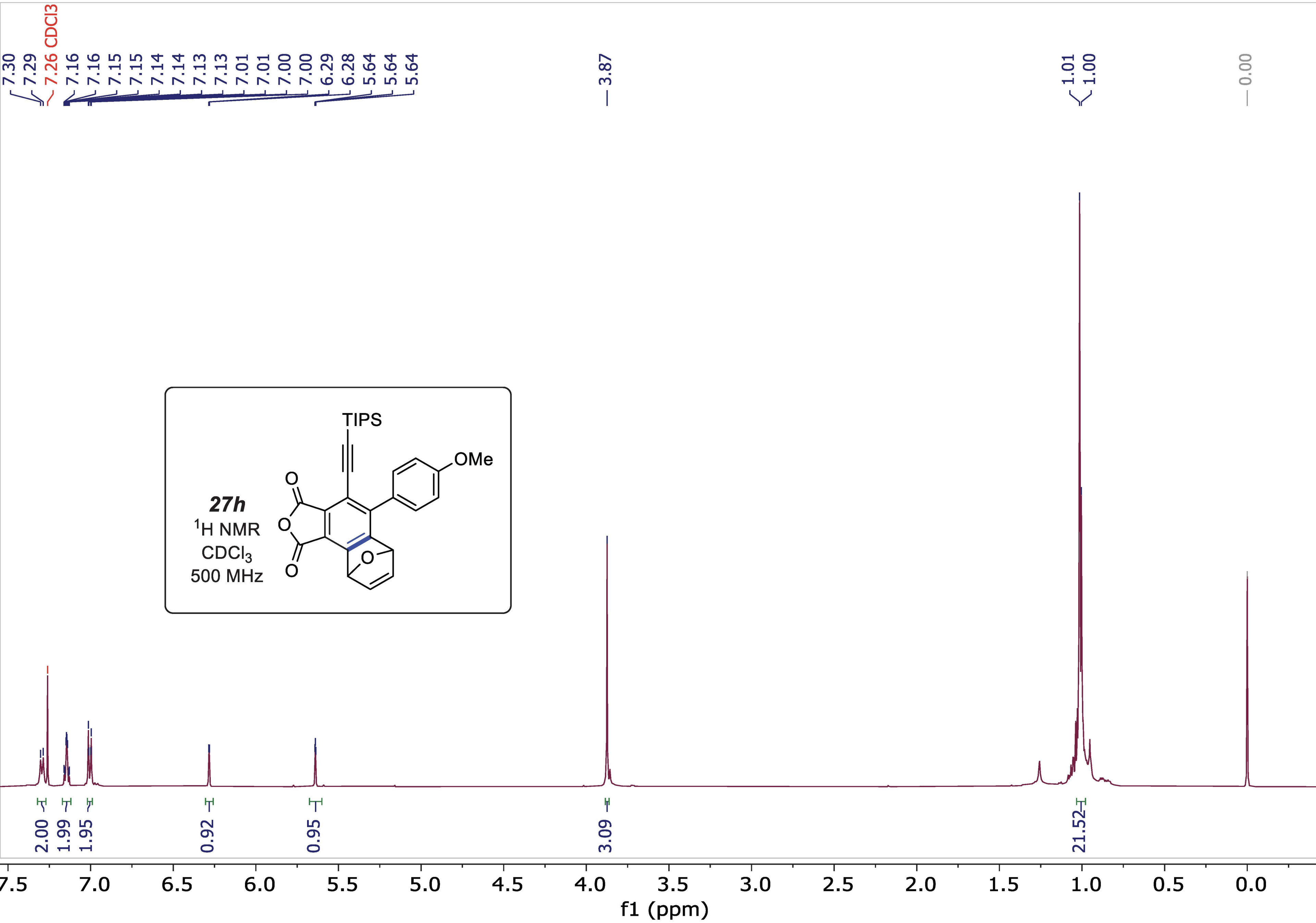
7.30
7.29
7.26 CDCl3
7.16
7.16
7.15
7.15
7.14
7.14
7.13
7.13
7.01
7.01
7.00
7.00
6.29
6.28
5.64
5.64
5.64
3.87
1.01
1.00
0.00
TIPS
OMe
O
O
O
O
27h
1H NMR
CDCl3
500 MHz
2.00
1.99
1.95
0.92
0.95
3.09
21.52
7.5
7.0
6.5
6.0
5.5
5.0
4.5
4.0
3.5
3.0
2.5
2.0
1.5
1.0
0.5
0.0
f1 (ppm)

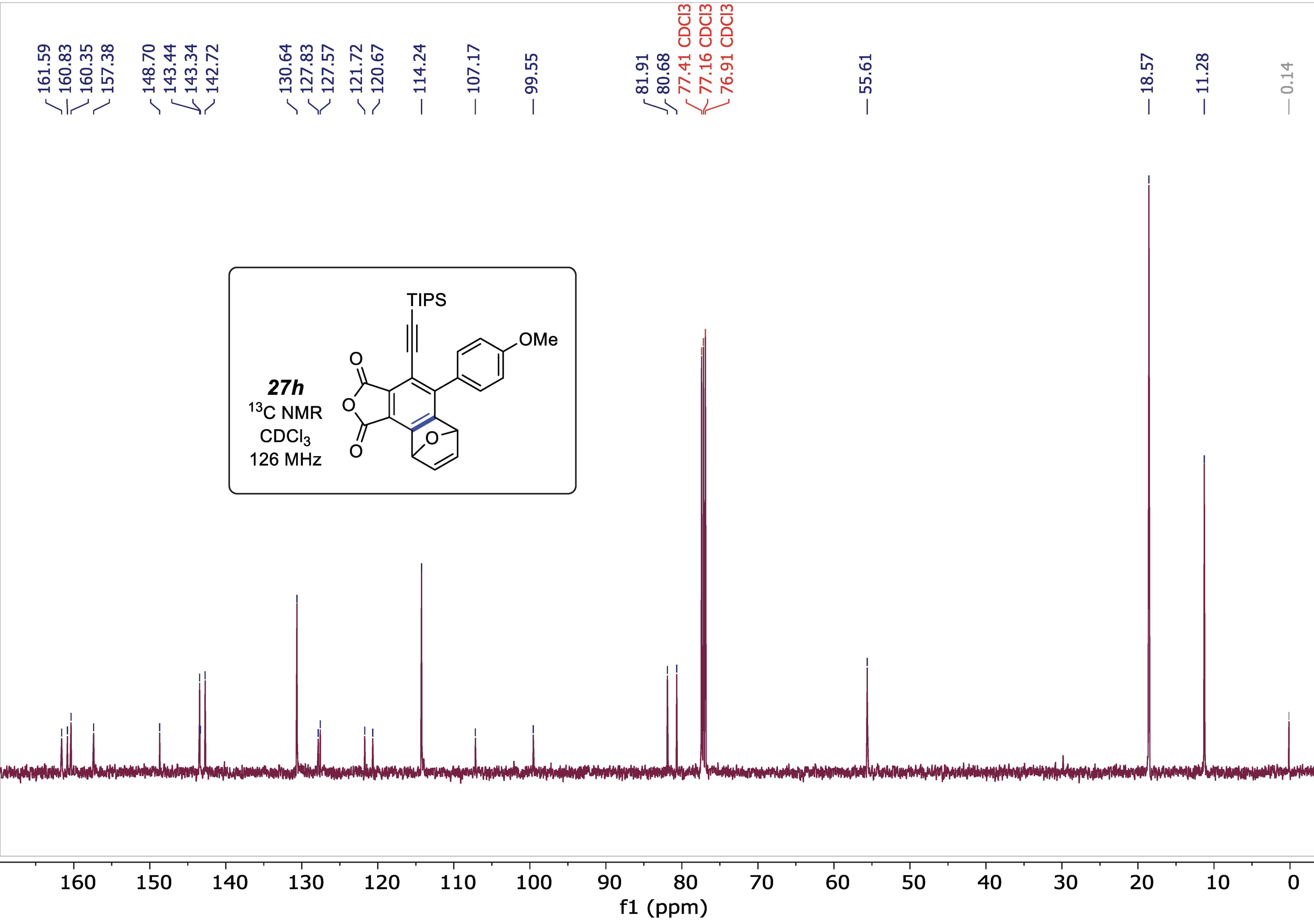
161.59
160.83
160.35
157.38
148.70
143.44
143.34
142.72
130.64
127.83
127.57
121.72
120.67
114.24
107.17
99.55
81.91
80.68
77.41 CDCl3
77.16 CDCl3
76.91 CDCl3
55.61
18.57
11.28
0.14
27h
^{13}C NMR
$CDCl_3$
126 MHz
TIPS
OMe
O
160
150
140
130
120
110
100
90
80
70
60
50
40
30
20
10
0
f1 (ppm)

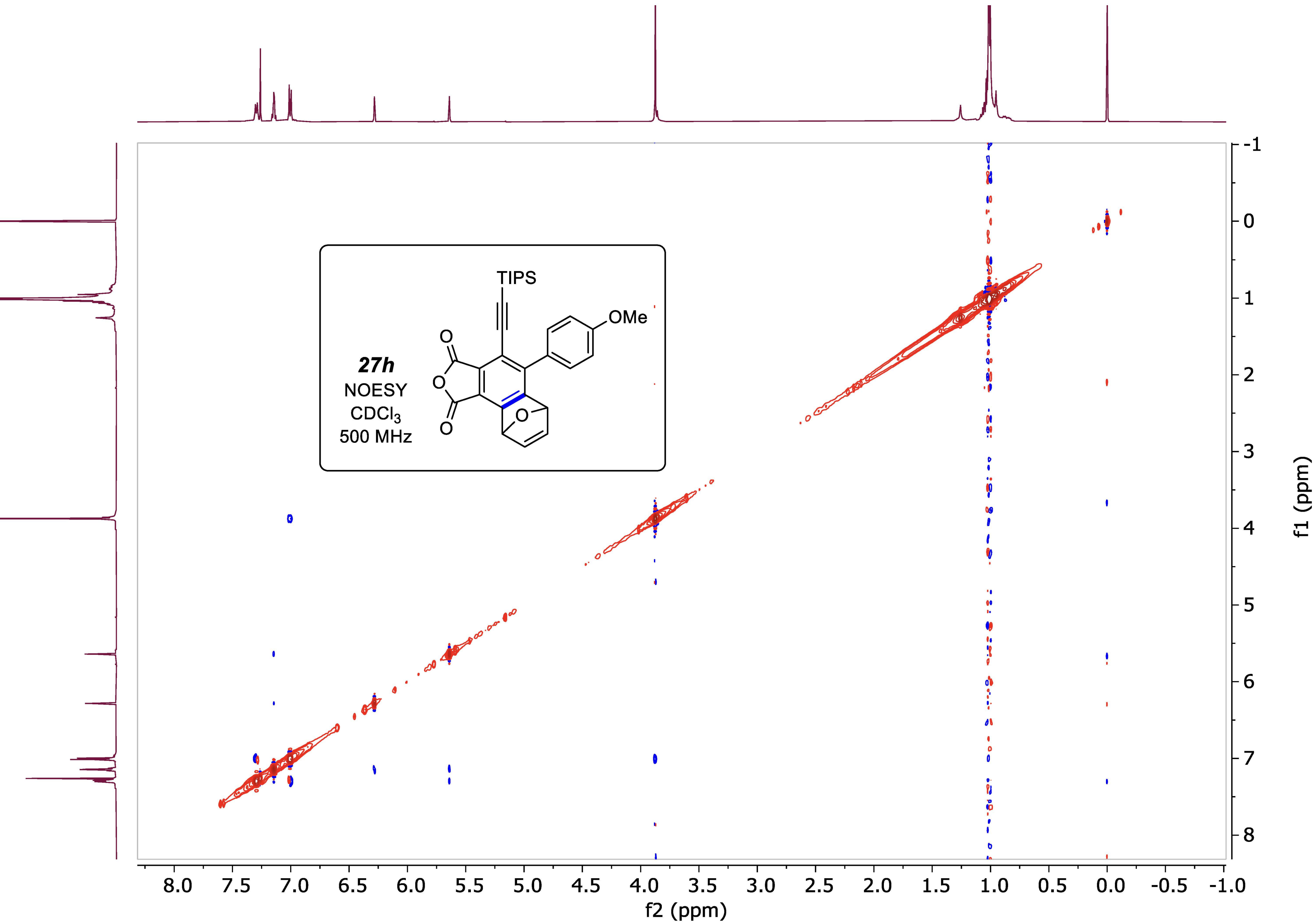
27h
NOESY
CDCl3
500 MHz
TIPS
OMe
O
O
O
O
f1 (ppm)
f2 (ppm)
8.0 7.5 7.0 6.5 6.0 5.5 5.0 4.5 4.0 3.5 3.0 2.5 2.0 1.5 1.0 0.5 0.0 -0.5 -1.0
-1 0 1 2 3 4 5 6 7 8

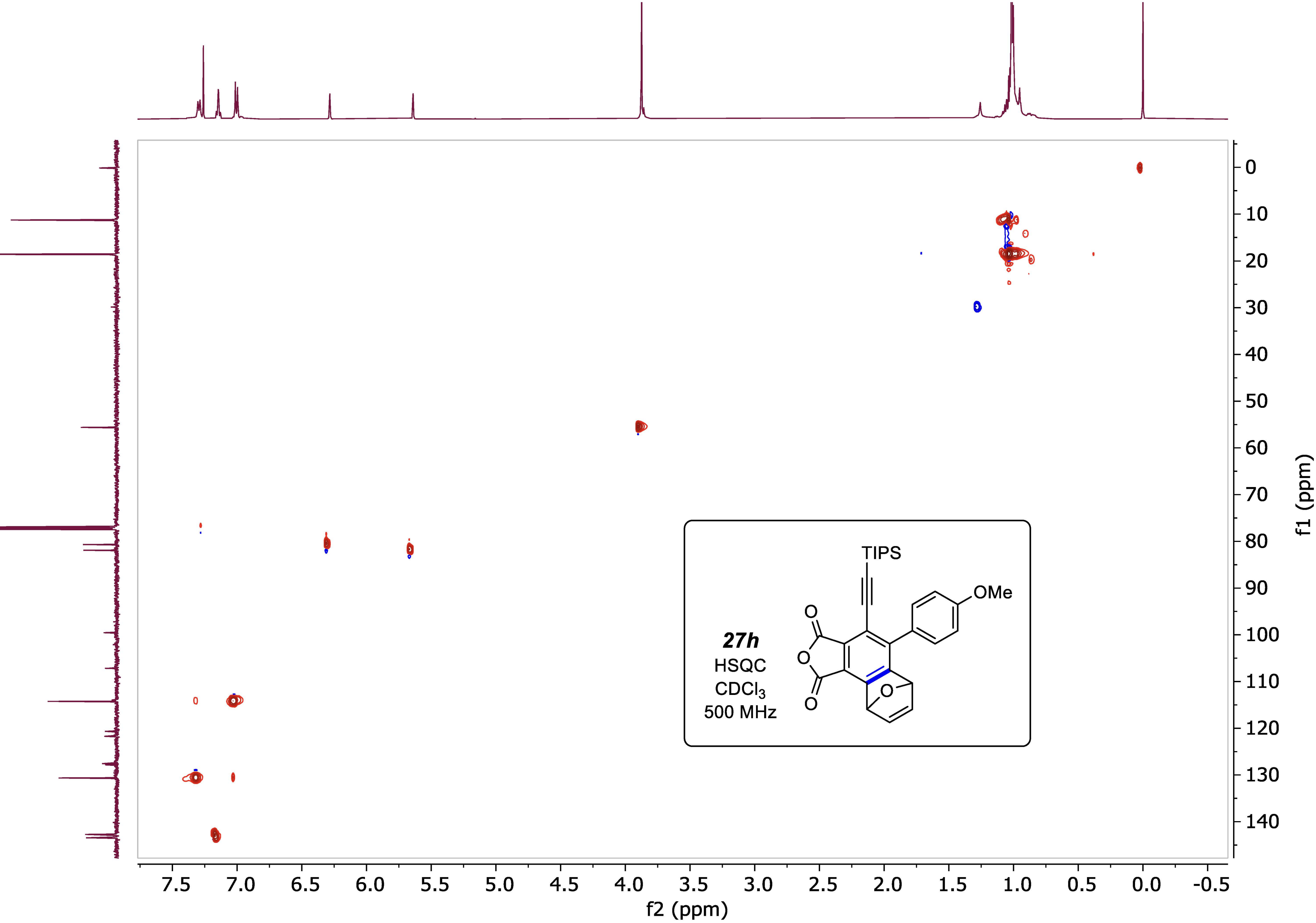
27h
HSQC
CDCl3
500 MHz
TIPS
OMe
O
O
O
O
f1 (ppm)
f2 (ppm)

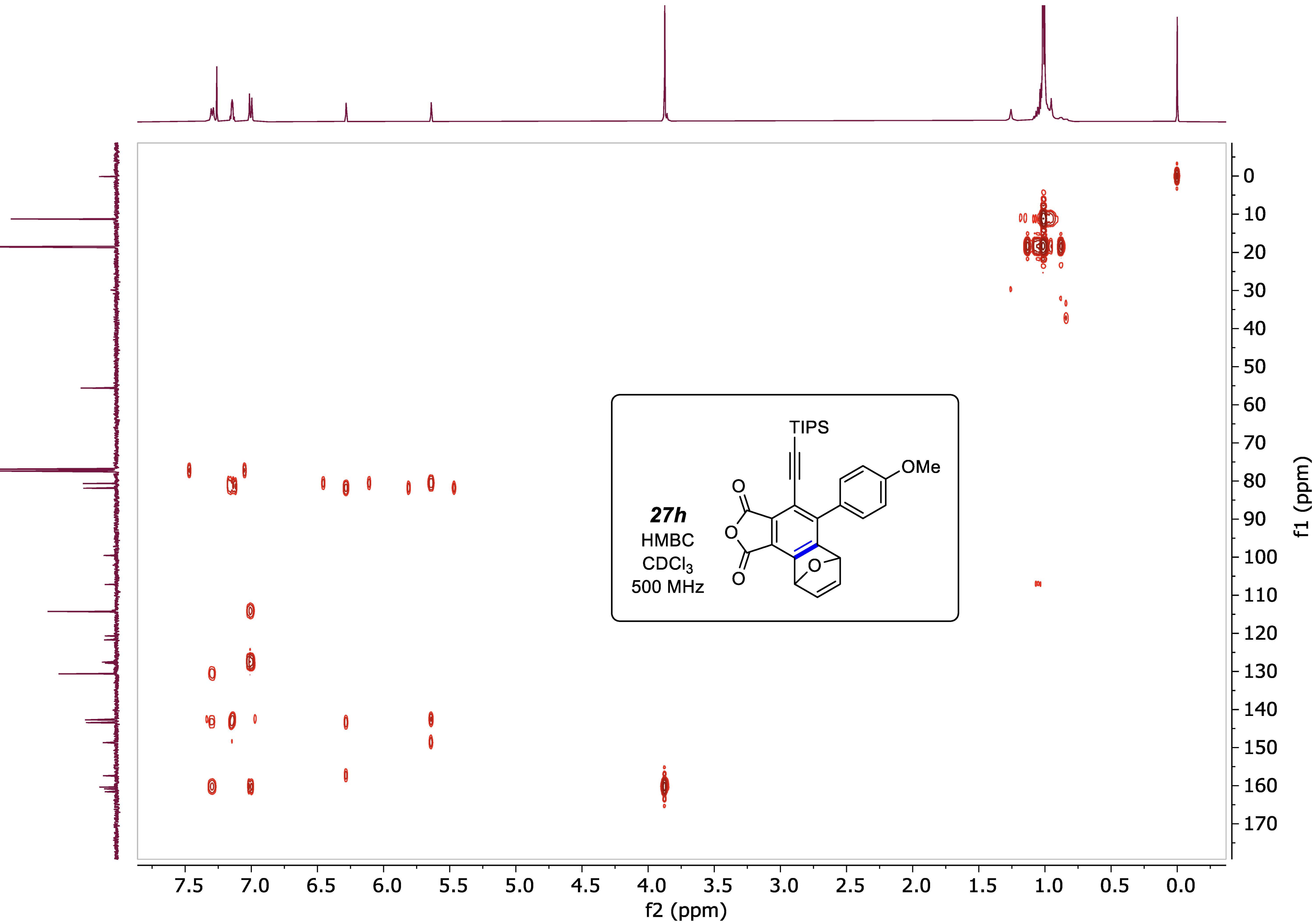
27h
HMBC
CDCl3
500 MHz
TIPS
OMe
f2 (ppm)
f1 (ppm)

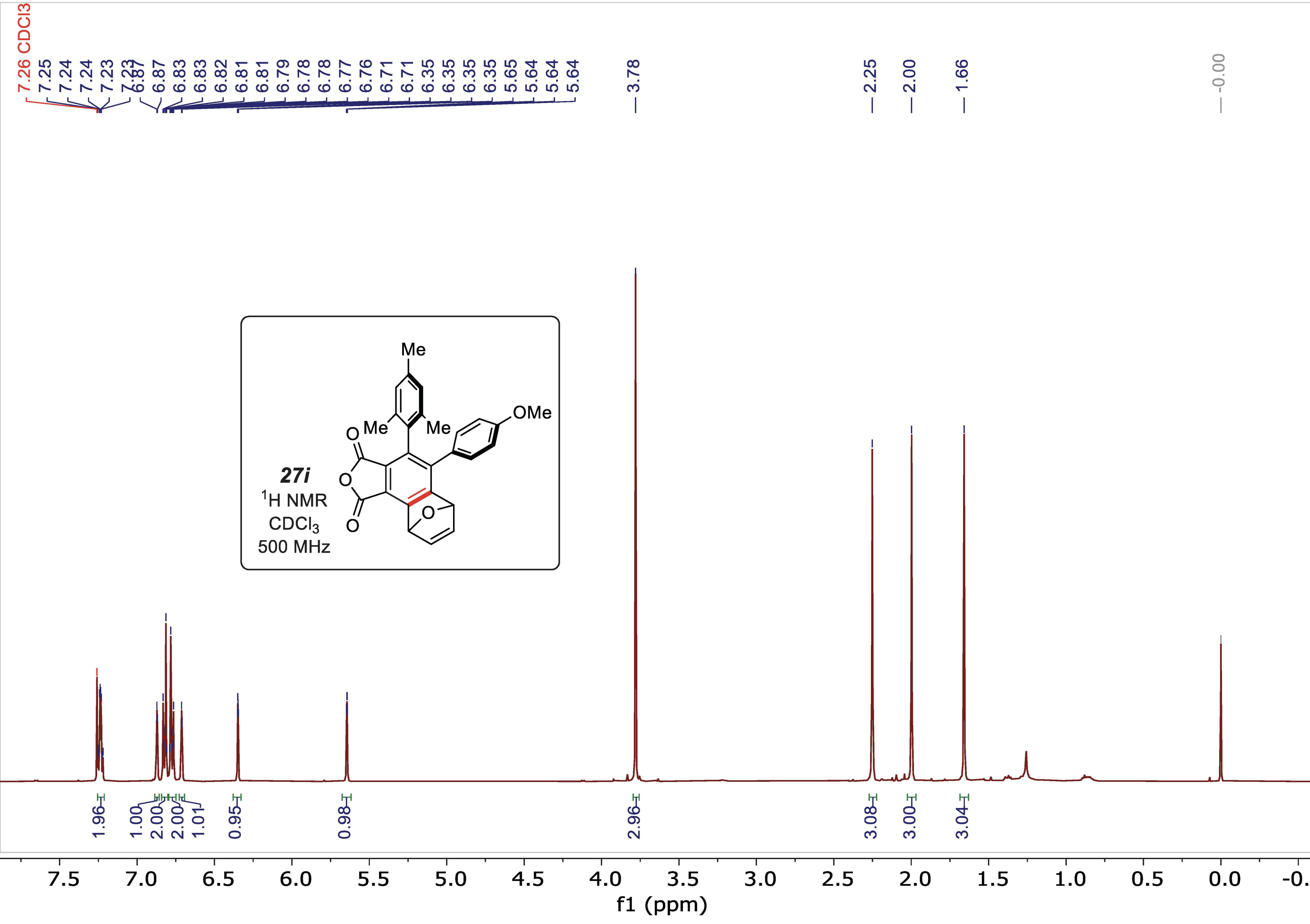
7.26 CDCl3
7.25
7.24
7.24
7.23
7.23
6.87
6.87
6.83
6.83
6.82
6.81
6.81
6.79
6.78
6.78
6.77
6.76
6.71
6.71
6.35
6.35
6.35
6.35
5.65
5.64
5.64
5.64
3.78
2.25
2.00
1.66
-0.00
Me
OMe
O
Me
Me
27i
^{1}H NMR
$CDCl_3$
500 MHz
O
O
O
1.96
1.00
2.00
2.00
1.01
0.95
0.98
2.96
3.08
3.00
3.04
f1 (ppm)

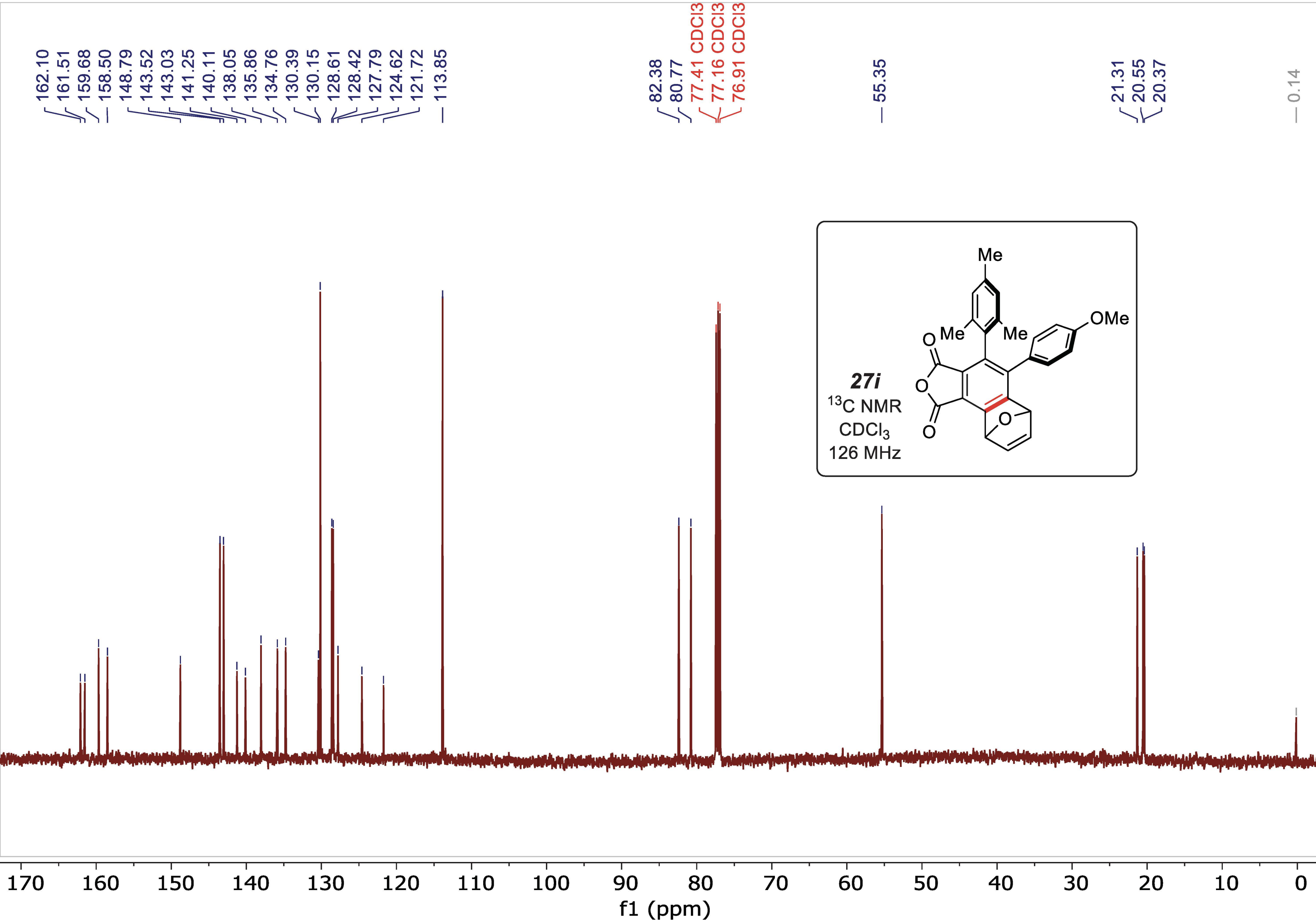
162.10
161.51
159.68
158.50
148.79
143.52
143.03
141.25
140.11
138.05
135.86
134.76
130.39
130.15
128.61
128.42
127.79
124.62
121.72
113.85
82.38
80.77
77.41 CDCl3
77.16 CDCl3
76.91 CDCl3
55.35
21.31
20.55
20.37
0.14
Me
OMe
Me
Me
O
O
O
O
27i
^{13}C NMR
$CDCl_3$
126 MHz
170
160
150
140
130
120
110
100
90
80
70
60
50
40
30
20
10
0
f1 (ppm)

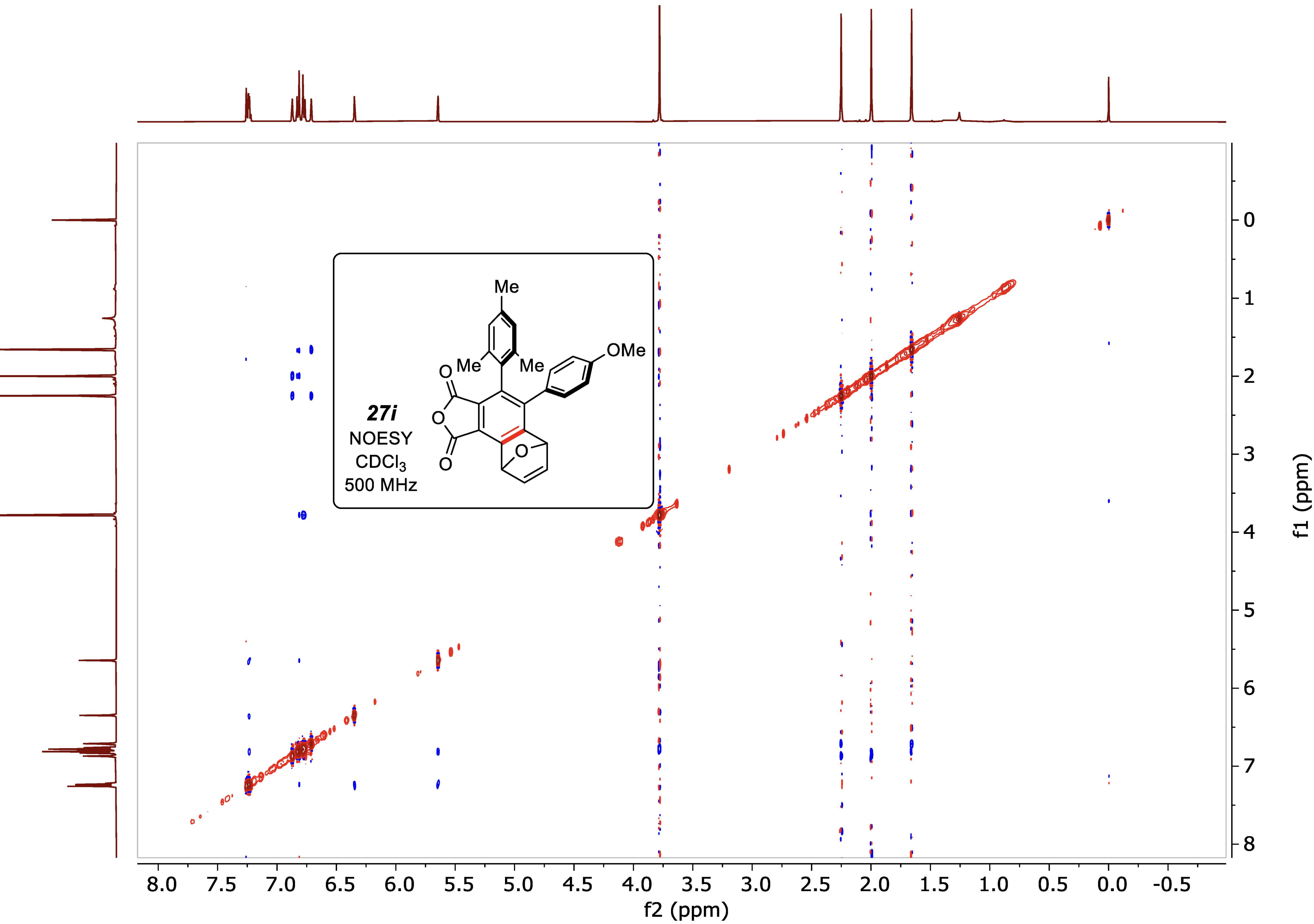
27i
NOESY
CDCl3
500 MHz
Me
Me
Me
OMe
O
O
O
O
f1 (ppm)
f2 (ppm)
8.0 7.5 7.0 6.5 6.0 5.5 5.0 4.5 4.0 3.5 3.0 2.5 2.0 1.5 1.0 0.5 0.0 -0.5
0 1 2 3 4 5 6 7 8

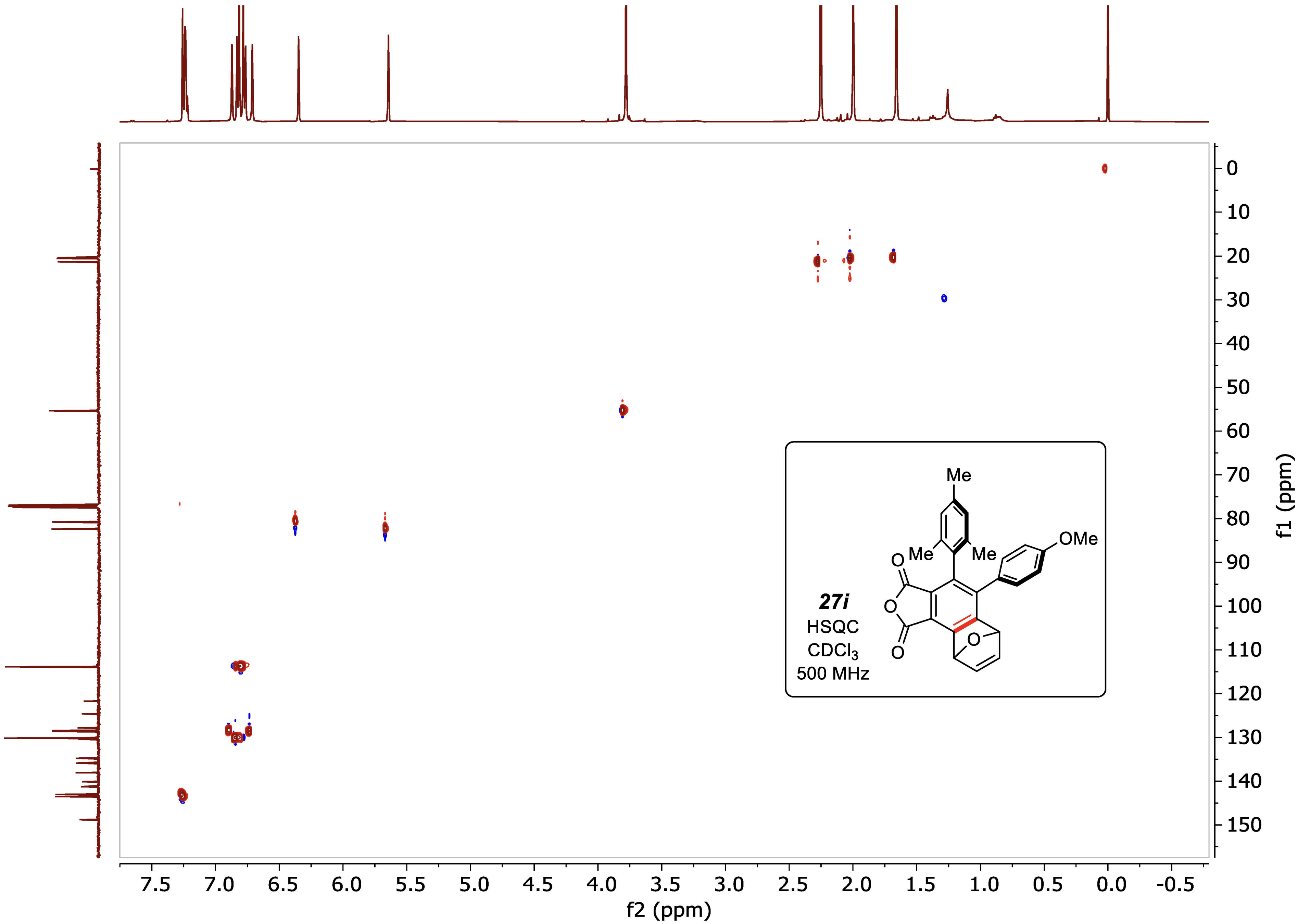
Me
Me
Me
OMe
O
O
O
O
27i
HSQC
CDCl3
500 MHz
f1 (ppm)
f2 (ppm)
7.5 7.0 6.5 6.0 5.5 5.0 4.5 4.0 3.5 3.0 2.5 2.0 1.5 1.0 0.5 0.0 -0.5
0 10 20 30 40 50 60 70 80 90 100 110 120 130 140 150

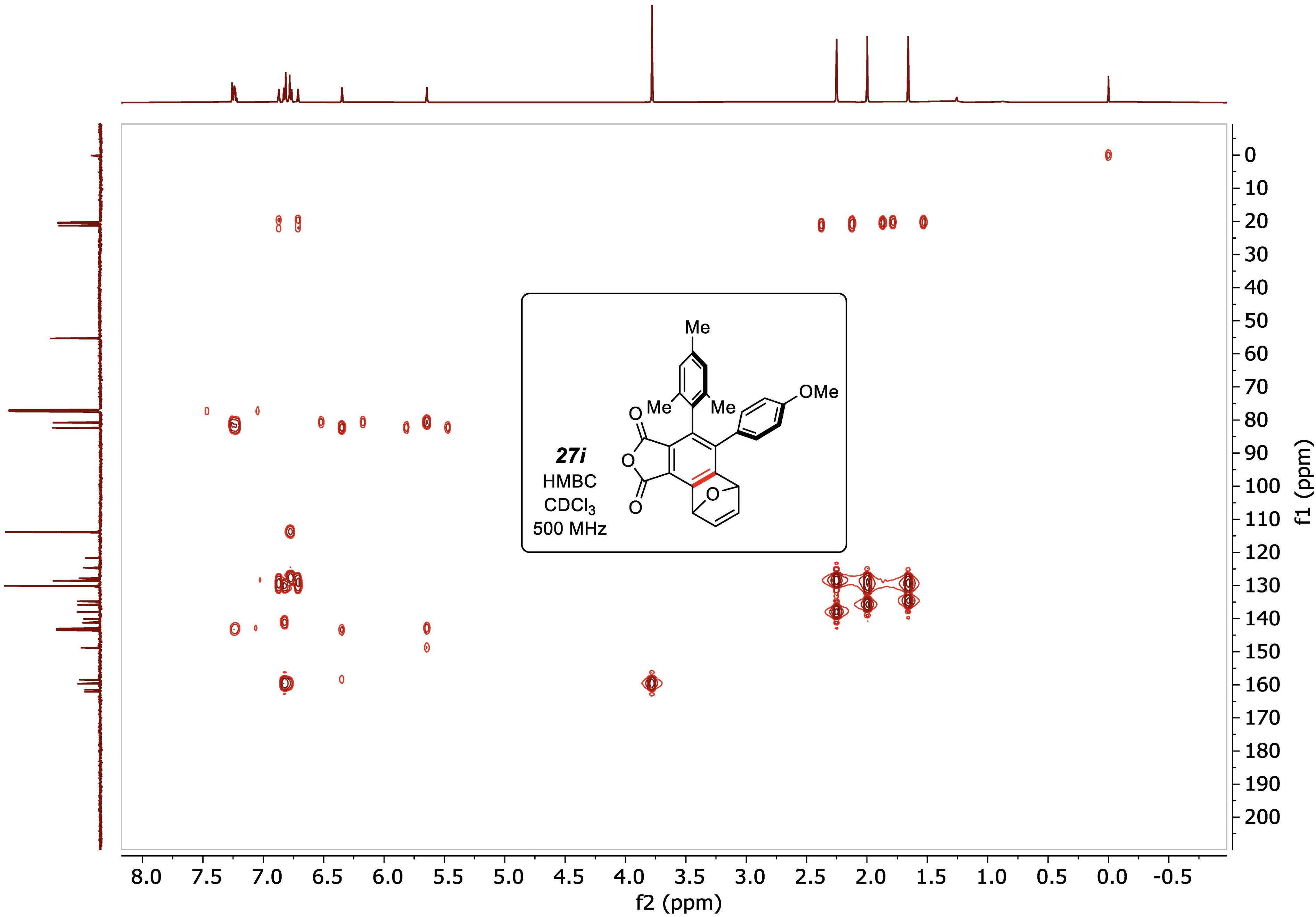
27i
HMBC
CDCl3
500 MHz
Me
Me
Me
OMe
O
O
O
O
f1 (ppm)
f2 (ppm)

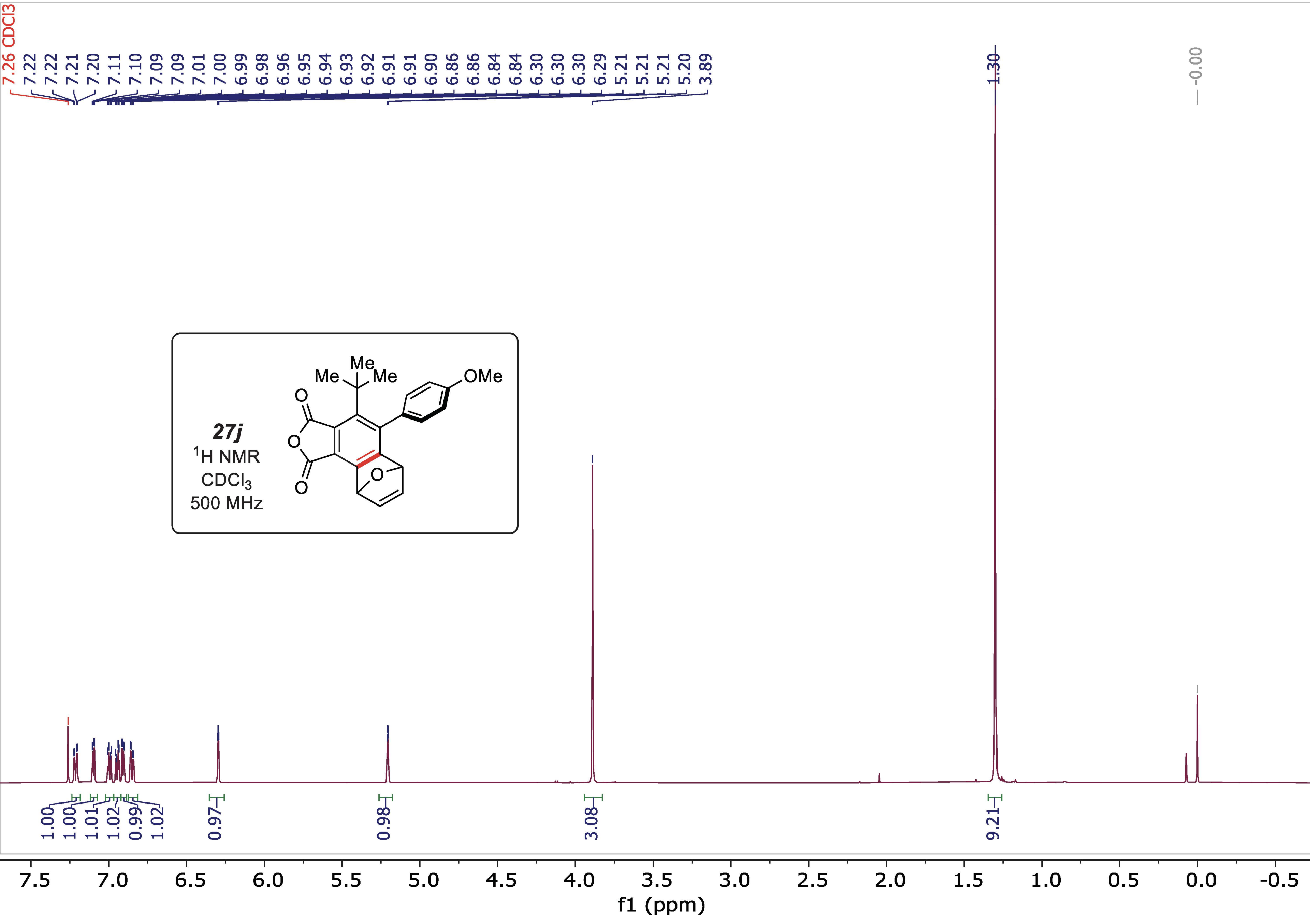
7.26 CDCl3
7.22
7.22
7.21
7.20
7.11
7.10
7.09
7.09
7.01
7.00
6.99
6.98
6.96
6.95
6.94
6.93
6.92
6.91
6.91
6.90
6.86
6.86
6.84
6.84
6.30
6.30
6.30
6.29
5.21
5.21
5.21
5.20
3.89
1.30
-0.00
27j
1H NMR
CDCl3
500 MHz
Me
Me
Me
OMe
O
O
O
O
1.00
1.00
1.01
1.02
0.99
1.02
0.97
0.98
3.08
9.21
7.5
7.0
6.5
6.0
5.5
5.0
4.5
4.0
3.5
3.0
2.5
2.0
1.5
1.0
0.5
0.0
-0.5
f1 (ppm)

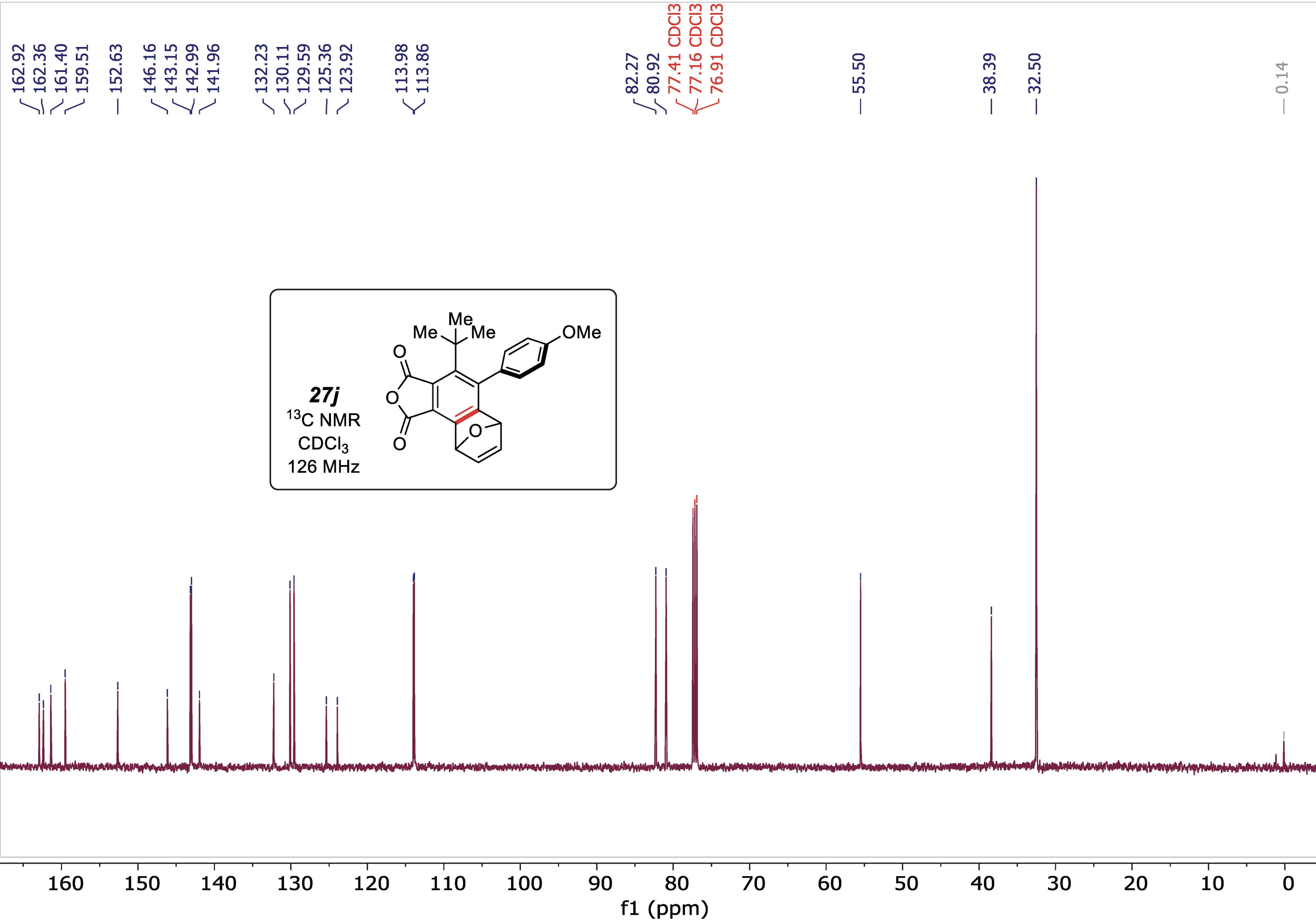
162.92
162.36
161.40
159.51
152.63
146.16
143.15
142.99
141.96
132.23
130.11
129.59
125.36
123.92
113.98
113.86
82.27
80.92
77.41 CDCl3
77.16 CDCl3
76.91 CDCl3
55.50
38.39
32.50
0.14
27j
^{13}C NMR
$CDCl_3$
126 MHz
Me
Me
Me
OMe
O
O
O
O
160 150 140 130 120 110 100 90 80 70 60 50 40 30 20 10 0
f1 (ppm)

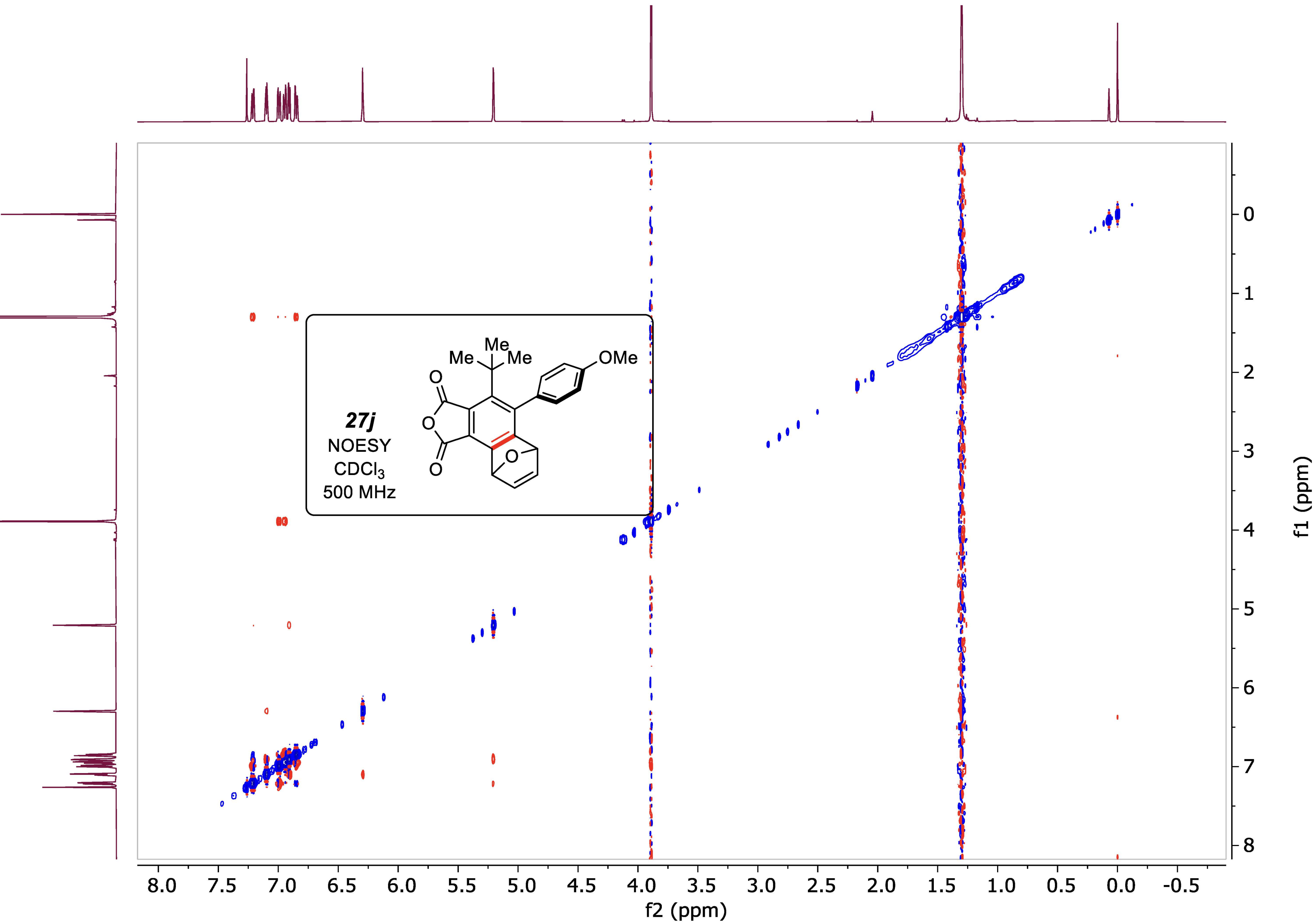
27j
NOESY
$CDCl_3$
500 MHz
Me
Me
Me
OMe
O
O
O
O
f2 (ppm)
f1 (ppm)

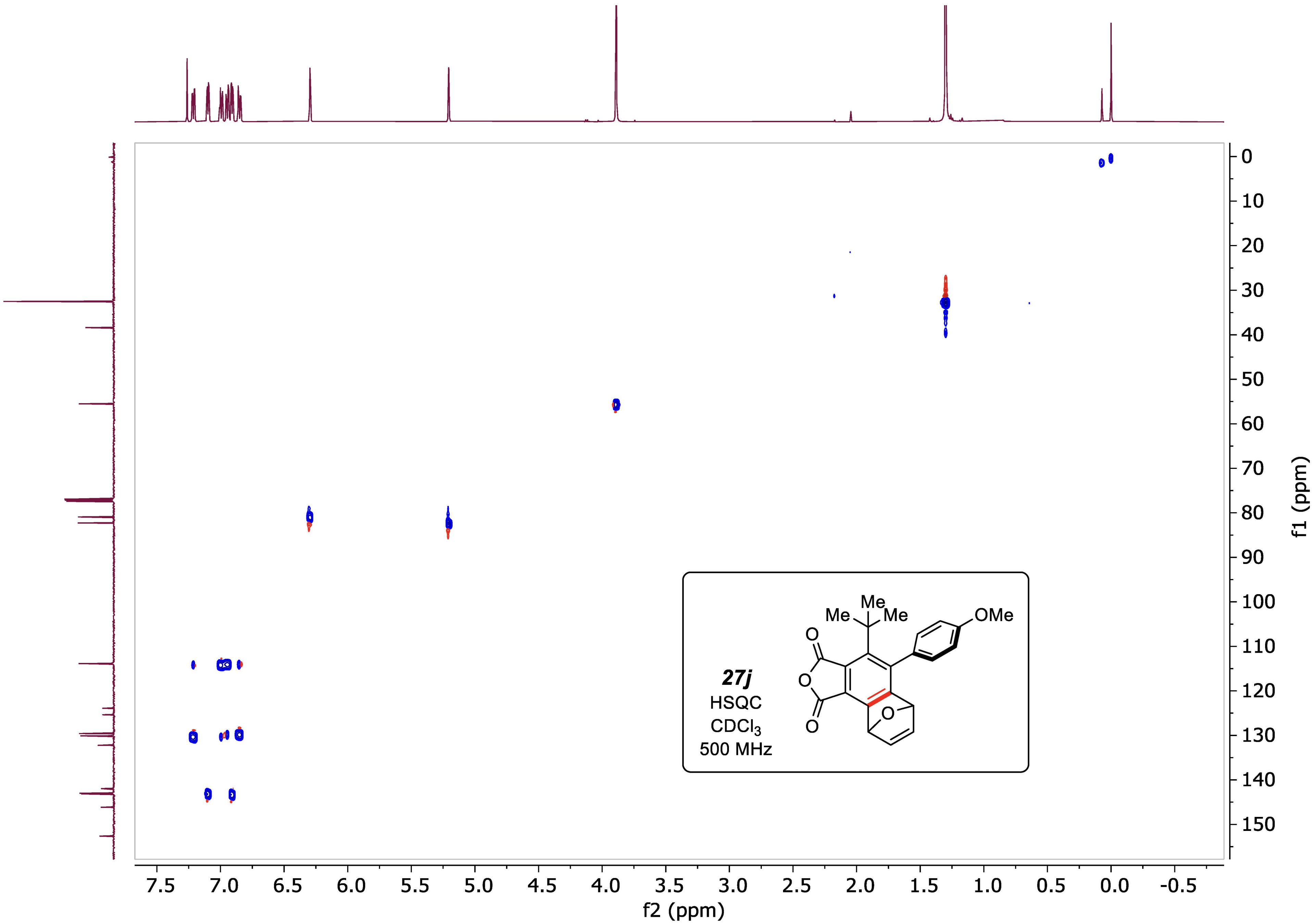
27j
HSQC
CDCl3
500 MHz
Me
Me
Me
OMe
O
O
O
O
f2 (ppm)
f1 (ppm)

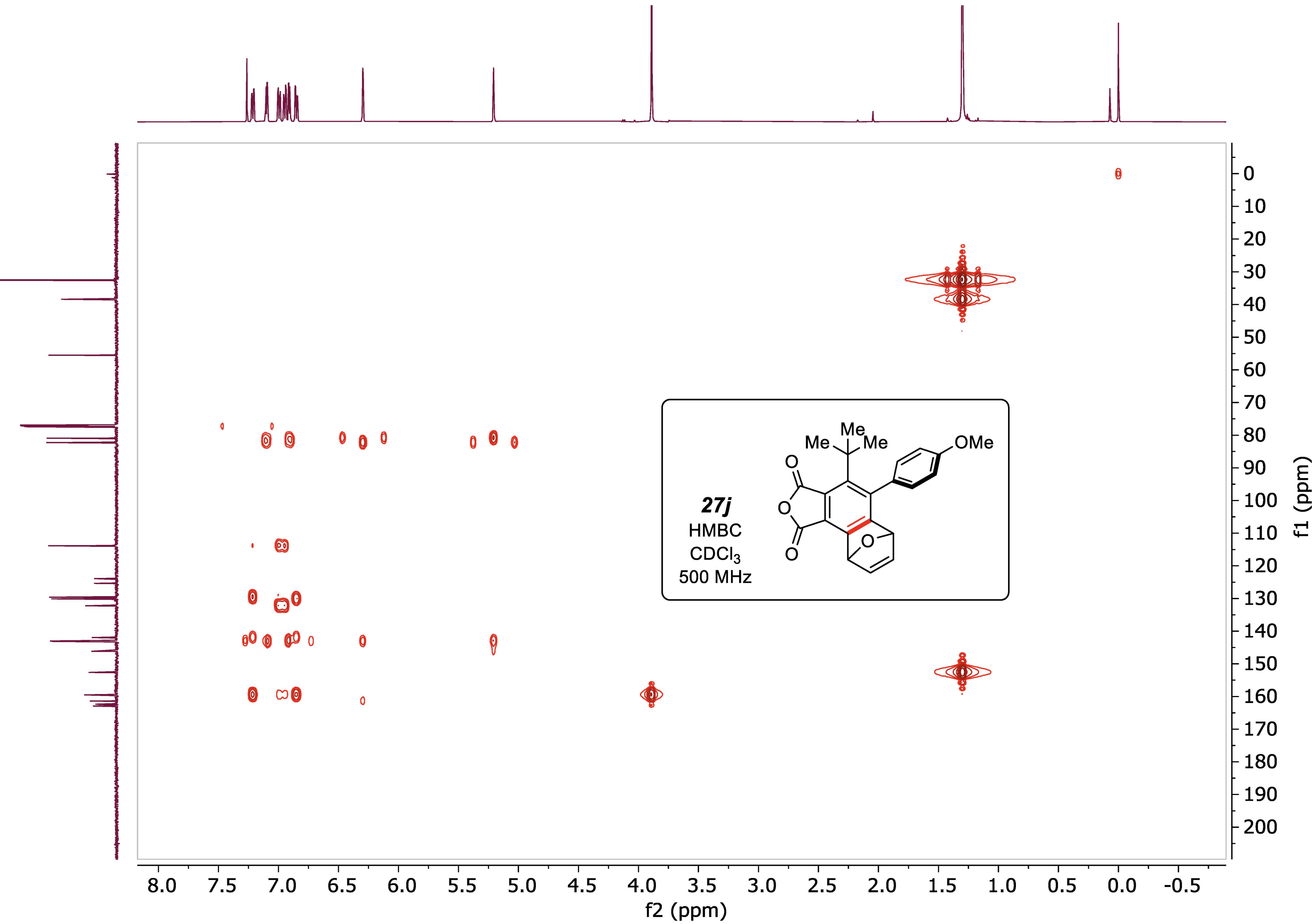
27j
HMBC
CDCl3
500 MHz
Me
Me
Me
OMe
O
O
O
O
f1 (ppm)
f2 (ppm)

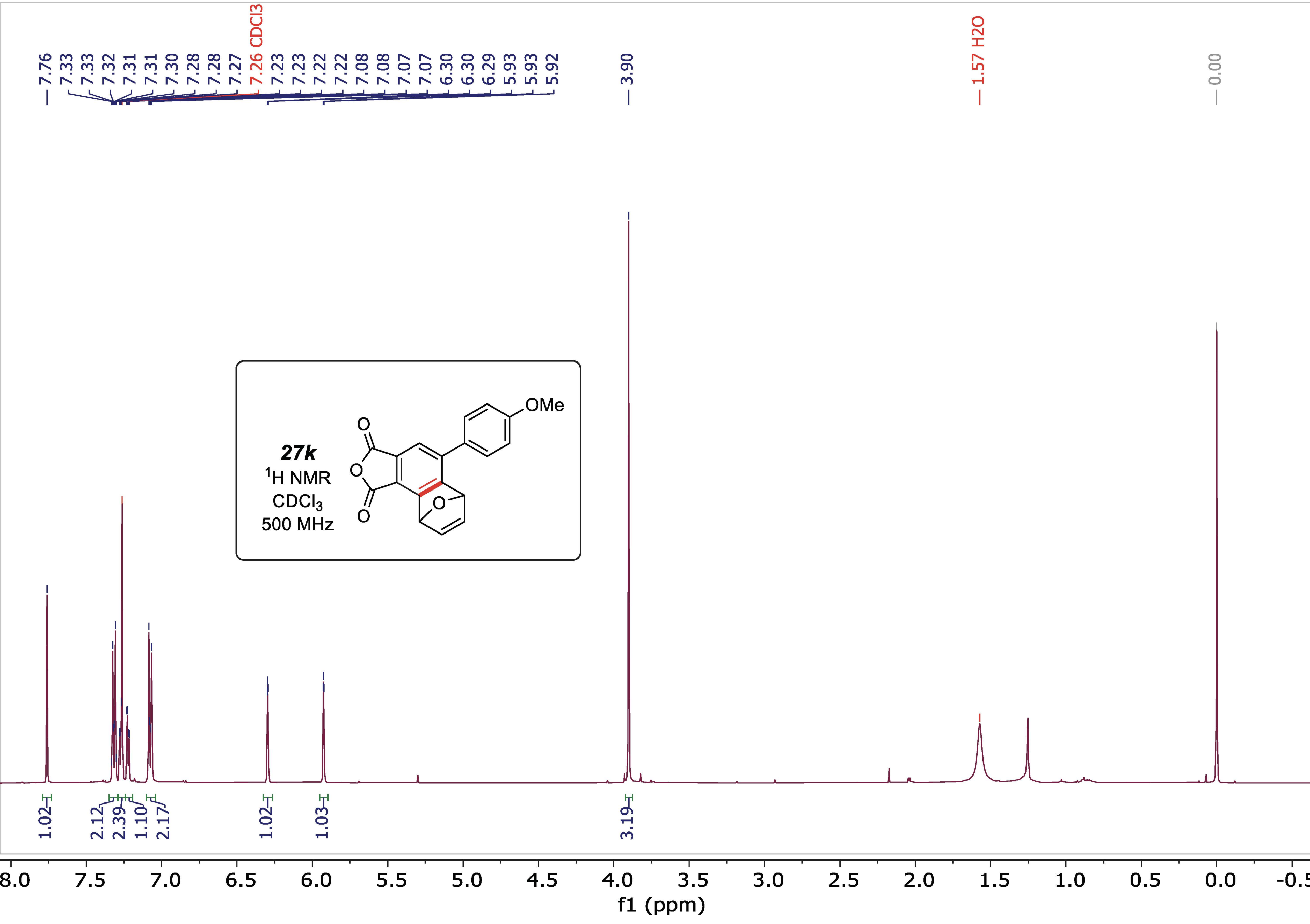
7.76
7.33
7.33
7.32
7.31
7.31
7.30
7.28
7.28
7.27
7.26 CDCl3
7.23
7.23
7.22
7.22
7.08
7.08
7.07
7.07
6.30
6.30
6.29
5.93
5.93
5.92
3.90
1.57 H2O
0.00
27k
1H NMR
CDCl3
500 MHz
O
O
O
O
OMe
1.02
2.12
2.39
1.10
2.17
1.02
1.03
3.19
8.0
7.5
7.0
6.5
6.0
5.5
5.0
4.5
4.0
3.5
3.0
2.5
2.0
1.5
1.0
0.5
0.0
-0.5
f1 (ppm)

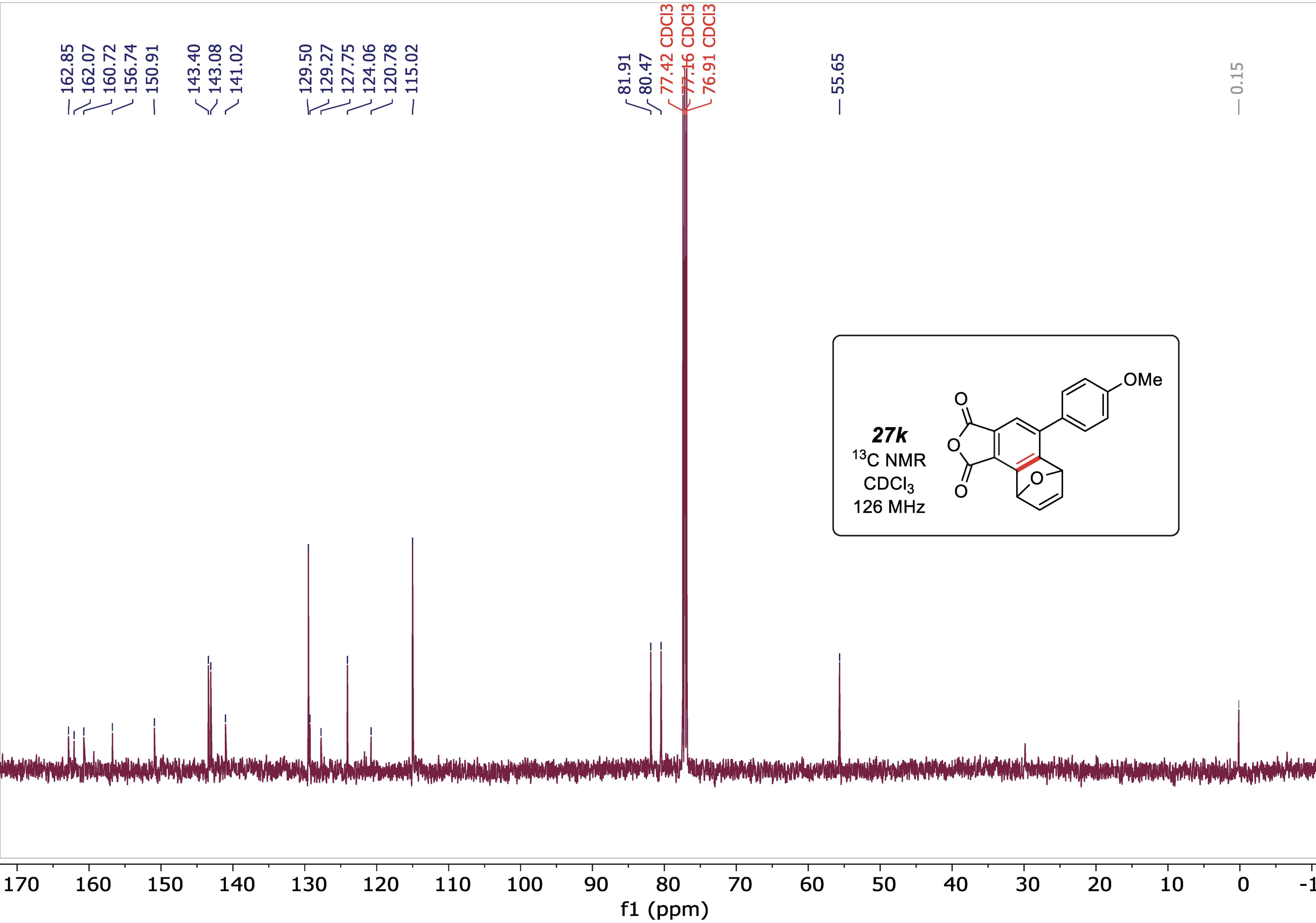
162.85
162.07
160.72
156.74
150.91
143.40
143.08
141.02
129.50
129.27
127.75
124.06
120.78
115.02
81.91
80.47
77.42 CDCl3
77.16 CDCl3
76.91 CDCl3
55.65
0.15
27k
^{13}C NMR
$CDCl_3$
126 MHz
OMe
O
f1 (ppm)
170
160
150
140
130
120
110
100
90
80
70
60
50
40
30
20
10
0
-1

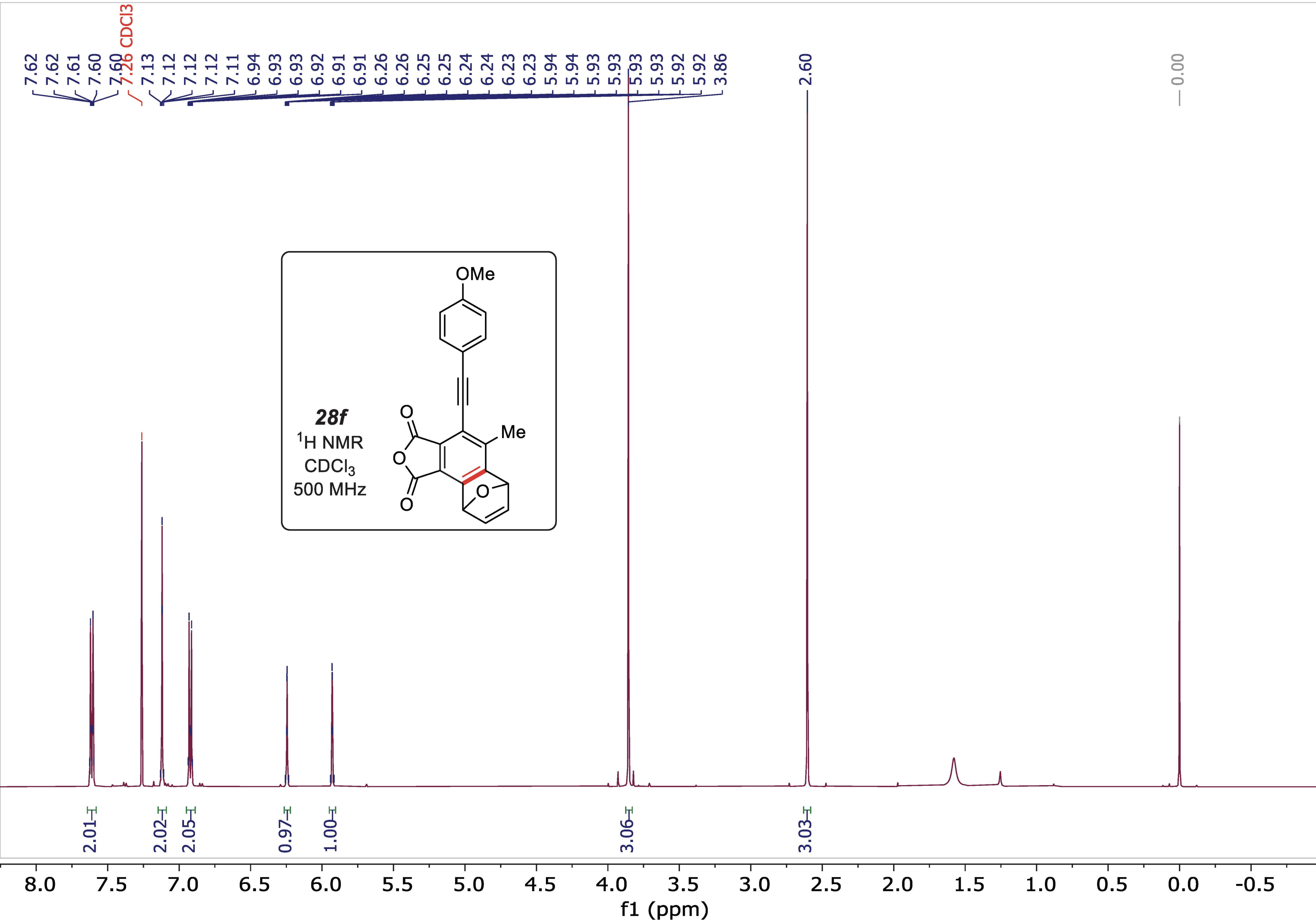
7.62
7.62
7.61
7.60
7.60
7.26 CDCl3
7.13
7.12
7.12
7.12
7.11
6.94
6.93
6.93
6.92
6.91
6.91
6.26
6.26
6.25
6.25
6.24
6.24
6.23
6.23
5.94
5.94
5.93
5.93
5.93
5.93
5.92
5.92
3.86
2.60
0.00
28f
^{1}H NMR
CDCl$_3$
500 MHz
OMe
Me
O
O
O
O
2.01
2.02
2.05
0.97
1.00
3.06
3.03
8.0 7.5 7.0 6.5 6.0 5.5 5.0 4.5 4.0 3.5 3.0 2.5 2.0 1.5 1.0 0.5 0.0 -0.5
f1 (ppm)

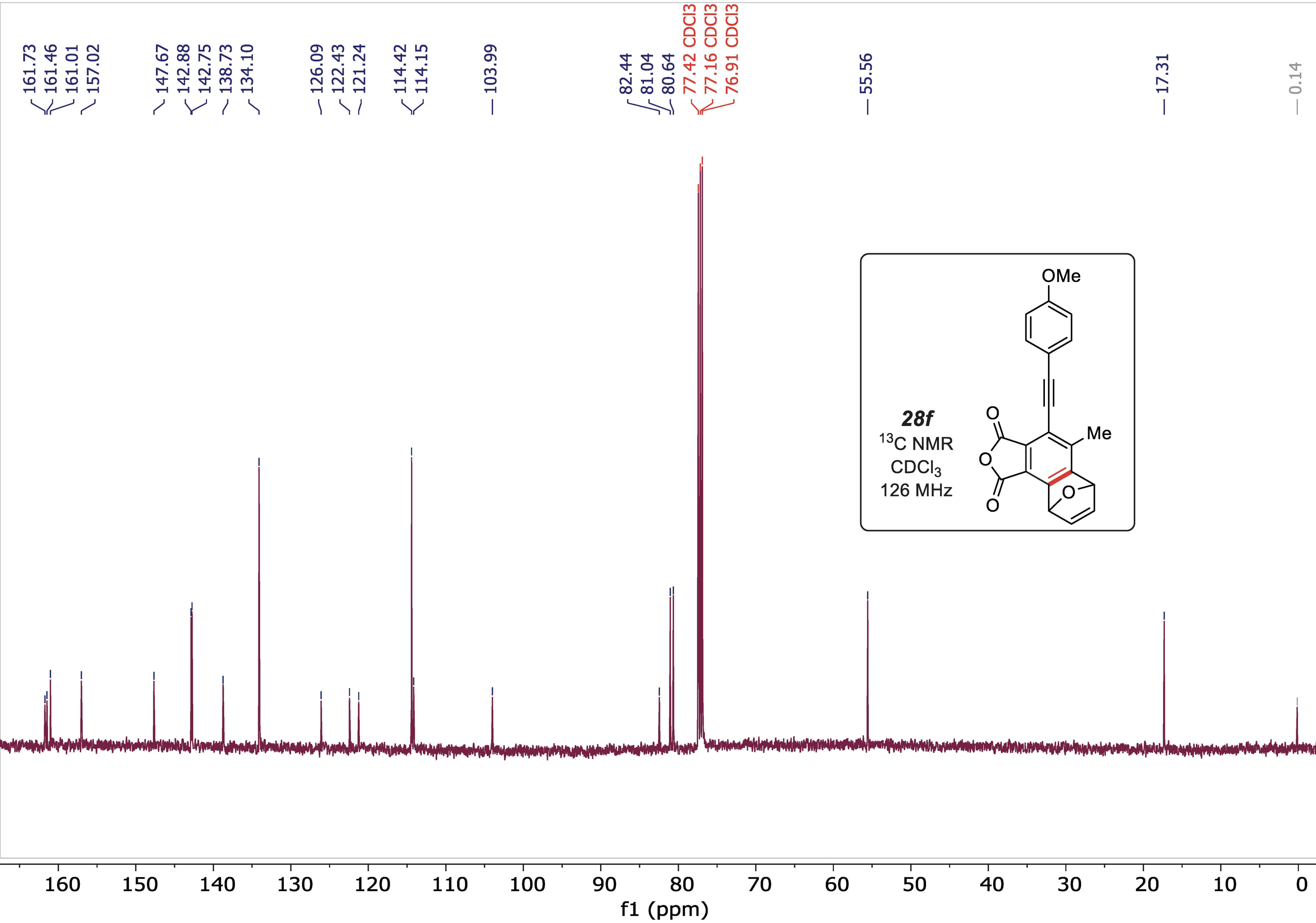
161.73
161.46
161.01
157.02
147.67
142.88
142.75
138.73
134.10
126.09
122.43
121.24
114.42
114.15
103.99
82.44
81.04
80.64
77.42 CDCl3
77.16 CDCl3
76.91 CDCl3
55.56
17.31
0.14
OMe
Me
O
28f
^{13}C NMR
$CDCl_3$
126 MHz
f1 (ppm)

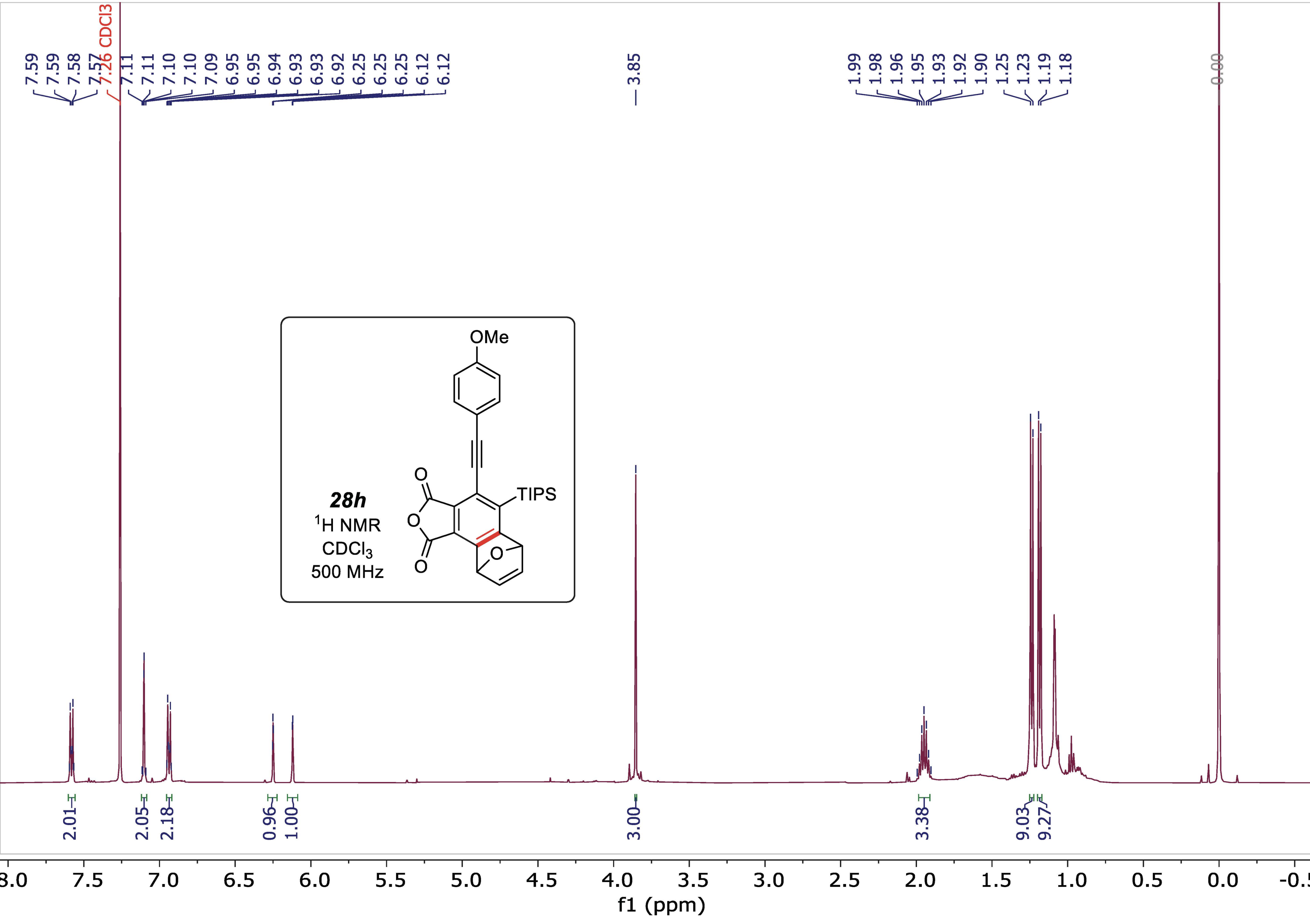
7.59 7.59 7.58 7.57 7.26 CDCl3 7.11 7.11 7.10 7.10 7.09 6.95 6.95 6.94 6.93 6.93 6.92 6.25 6.25 6.25 6.12 6.12
3.85
1.99 1.98 1.96 1.95 1.93 1.92 1.90 1.25 1.23 1.19 1.18
0.00
OMe
TIPS
O
O
O
O
28h
1H NMR
CDCl3
500 MHz
2.01 2.05 2.18 0.96 1.00 3.00 3.38 9.03 9.27
8.0 7.5 7.0 6.5 6.0 5.5 5.0 4.5 4.0 3.5 3.0 2.5 2.0 1.5 1.0 0.5 0.0 -0.5
f1 (ppm)

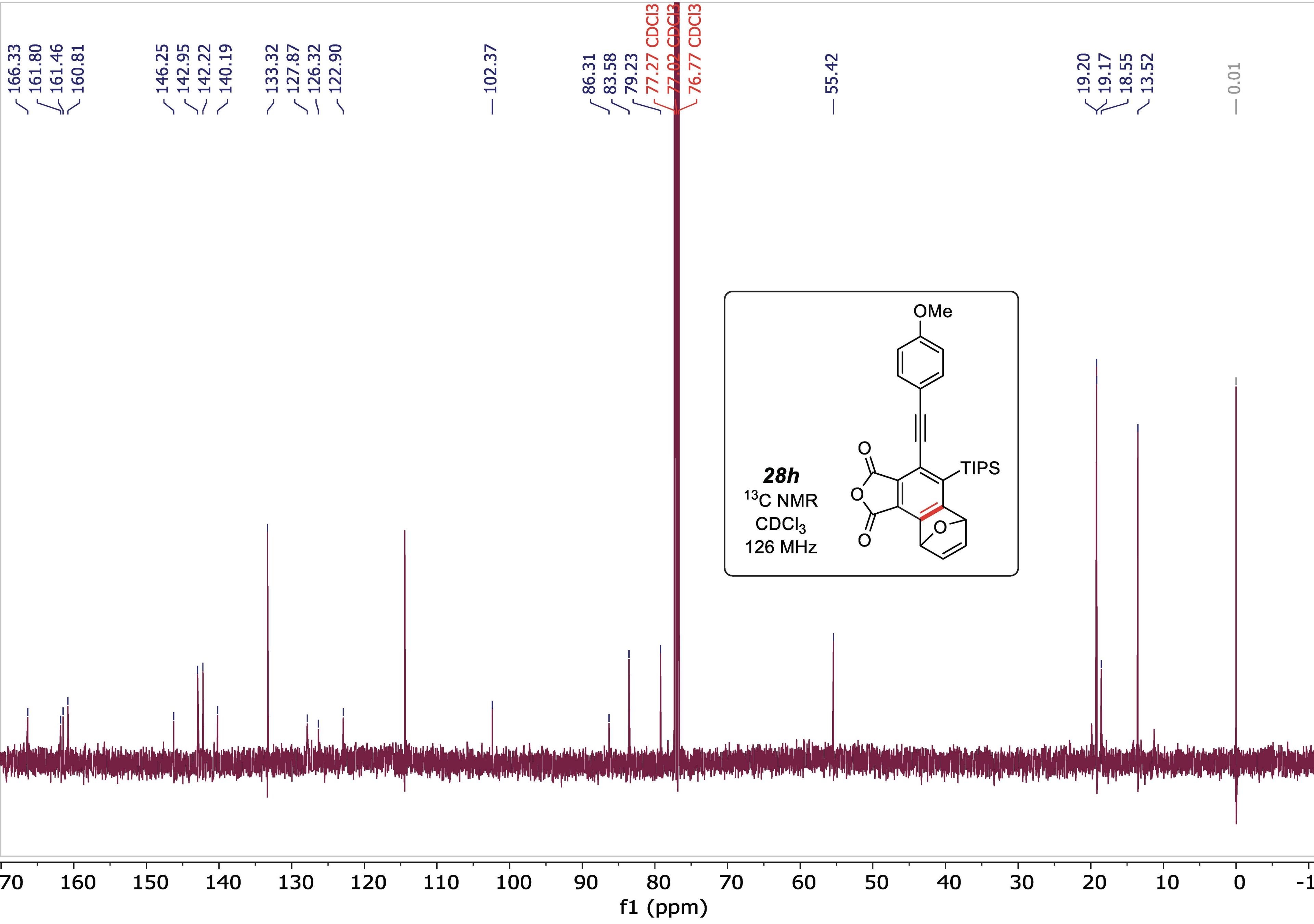
166.33
161.80
161.46
160.81
146.25
142.95
142.22
140.19
133.32
127.87
126.32
122.90
102.37
86.31
83.58
79.23
77.27 CDCl3
77.02 CDCl3
76.77 CDCl3
55.42
19.20
19.17
18.55
13.52
0.01
OMe
TIPS
O
O
O
O
28h
^{13}C NMR
$CDCl_3$
126 MHz
70 160 150 140 130 120 110 100 90 80 70 60 50 40 30 20 10 0 -1
f1 (ppm)

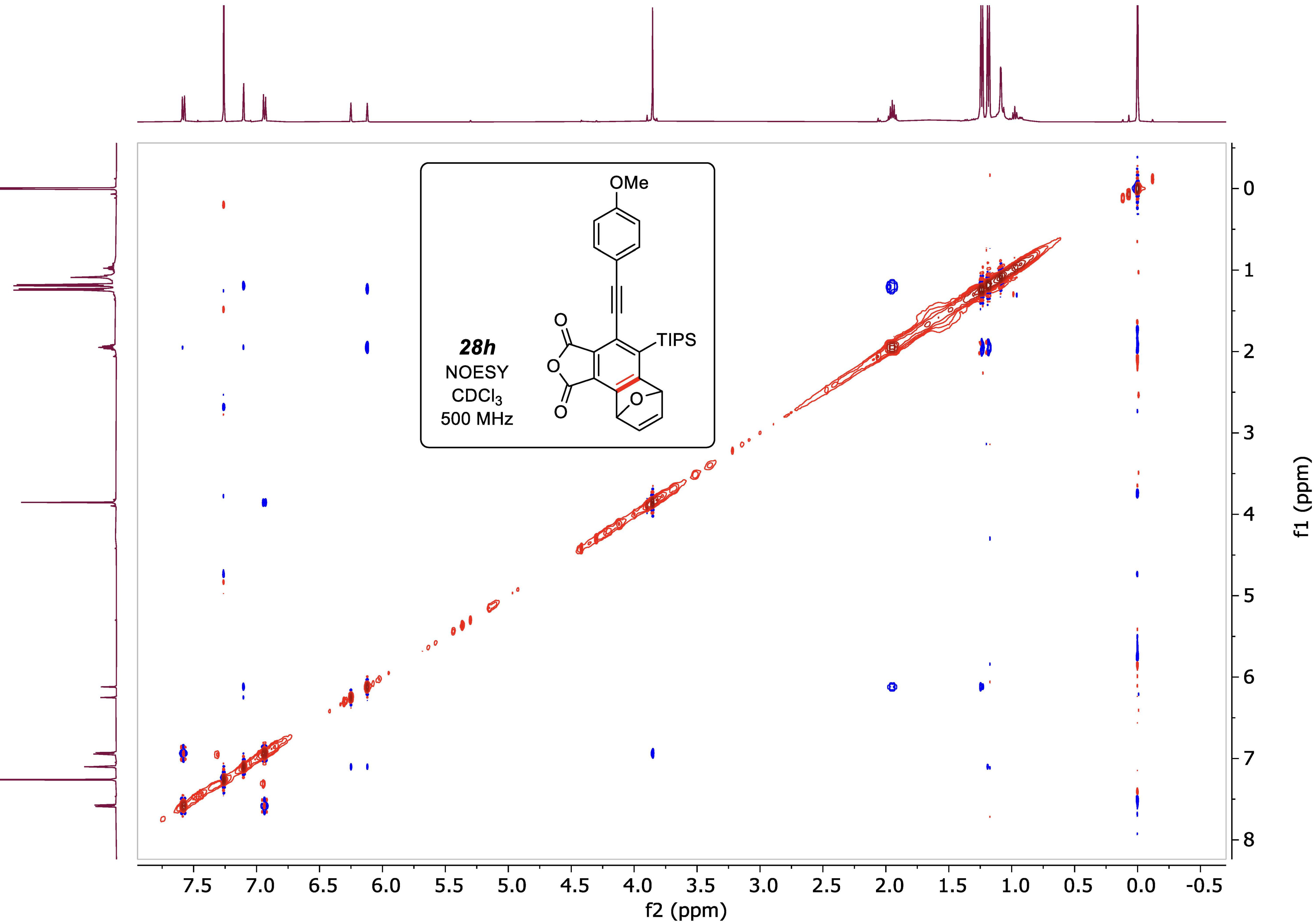
OMe
O
TIPS
O
O
O
28h
NOESY
CDCl3
500 MHz
f1 (ppm)
f2 (ppm)

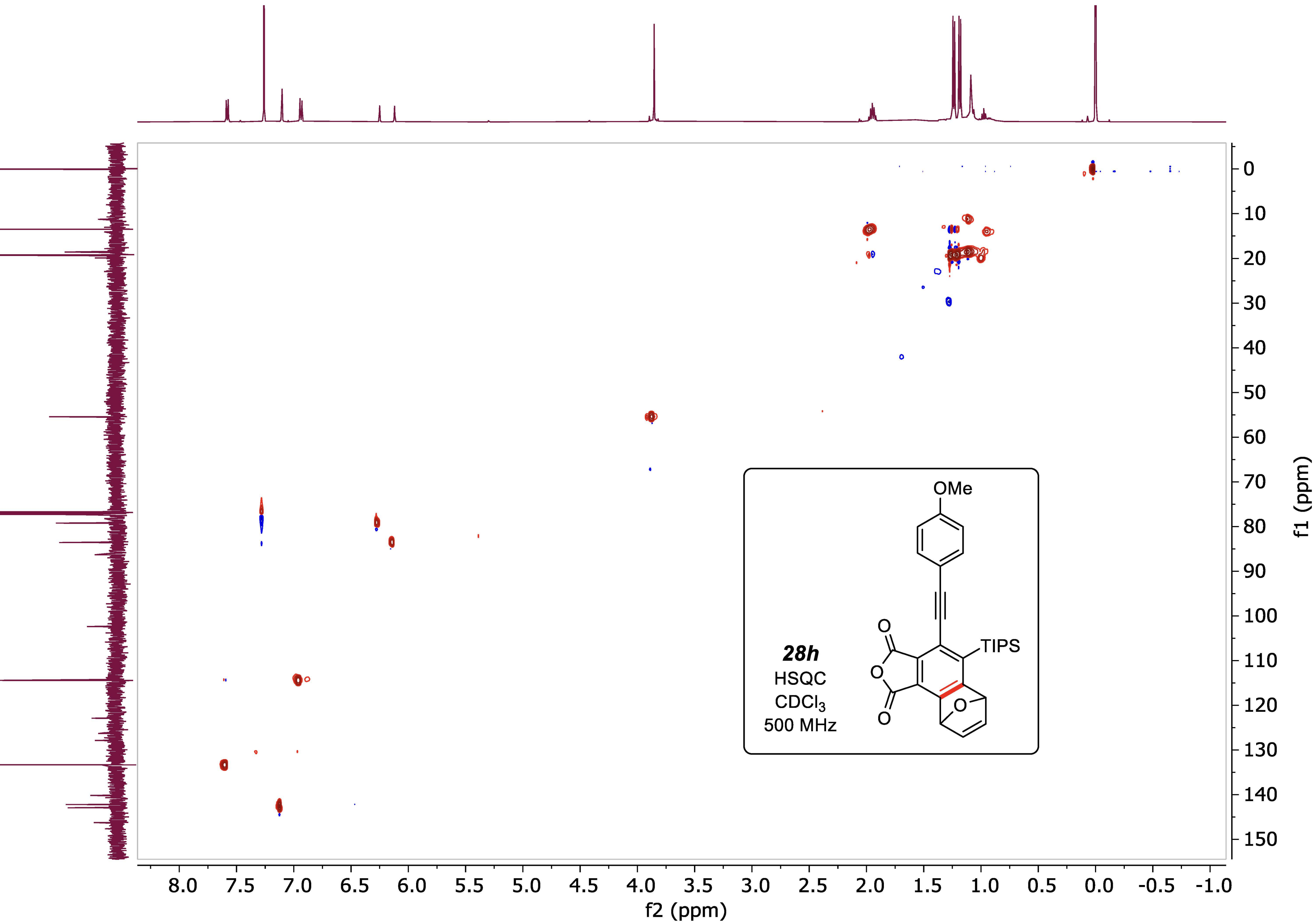

28h
HSQC
$CDCl_3$
500 MHz
OMe
TIPS
O
f1 (ppm)
f2 (ppm)

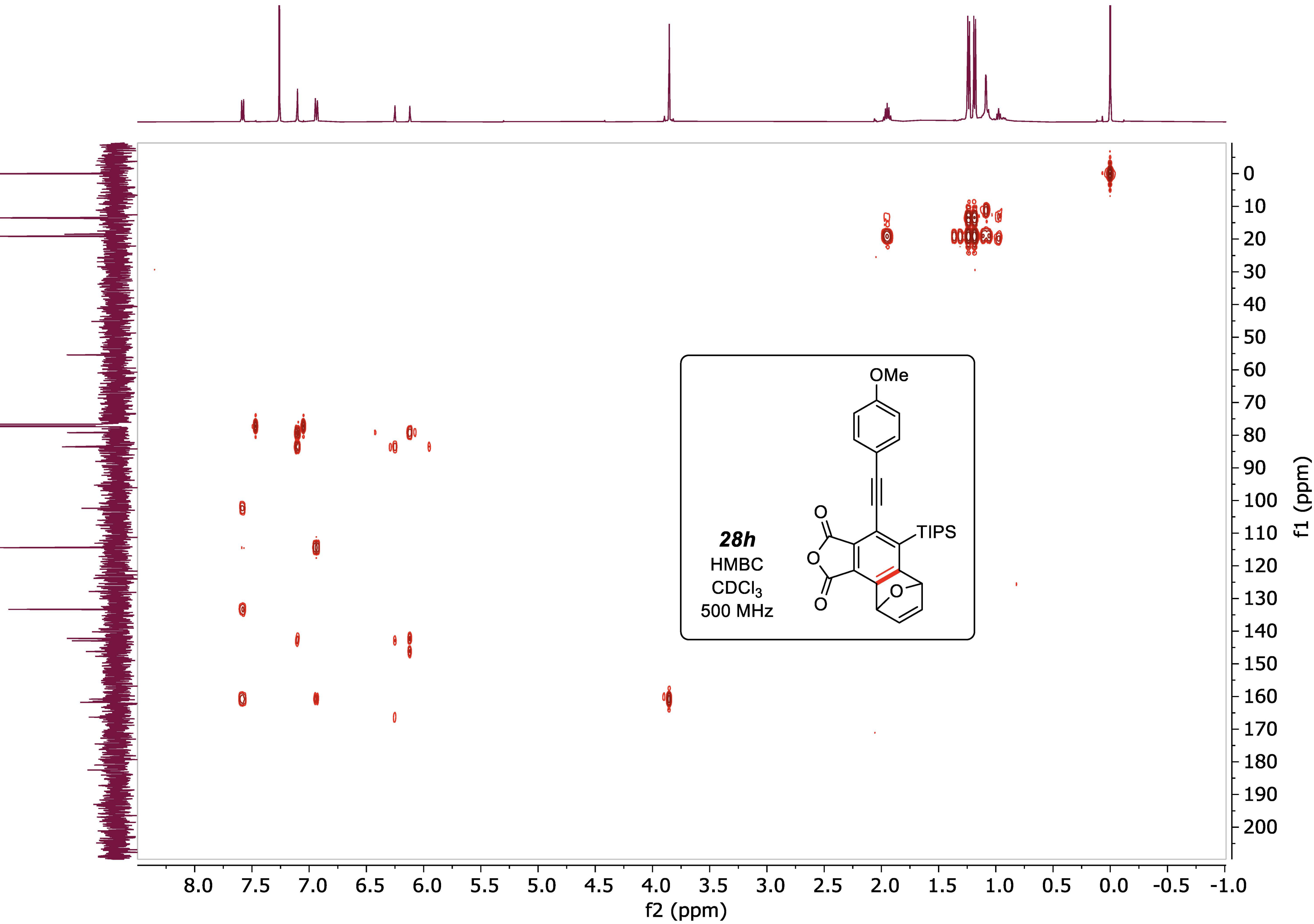
28h
HMBC
CDCl3
500 MHz
OMe
TIPS
O
f1 (ppm)
f2 (ppm)
8.0 7.5 7.0 6.5 6.0 5.5 5.0 4.5 4.0 3.5 3.0 2.5 2.0 1.5 1.0 0.5 0.0 -0.5 -1.0
0 10 20 30 40 50 60 70 80 90 100 110 120 130 140 150 160 170 180 190 200

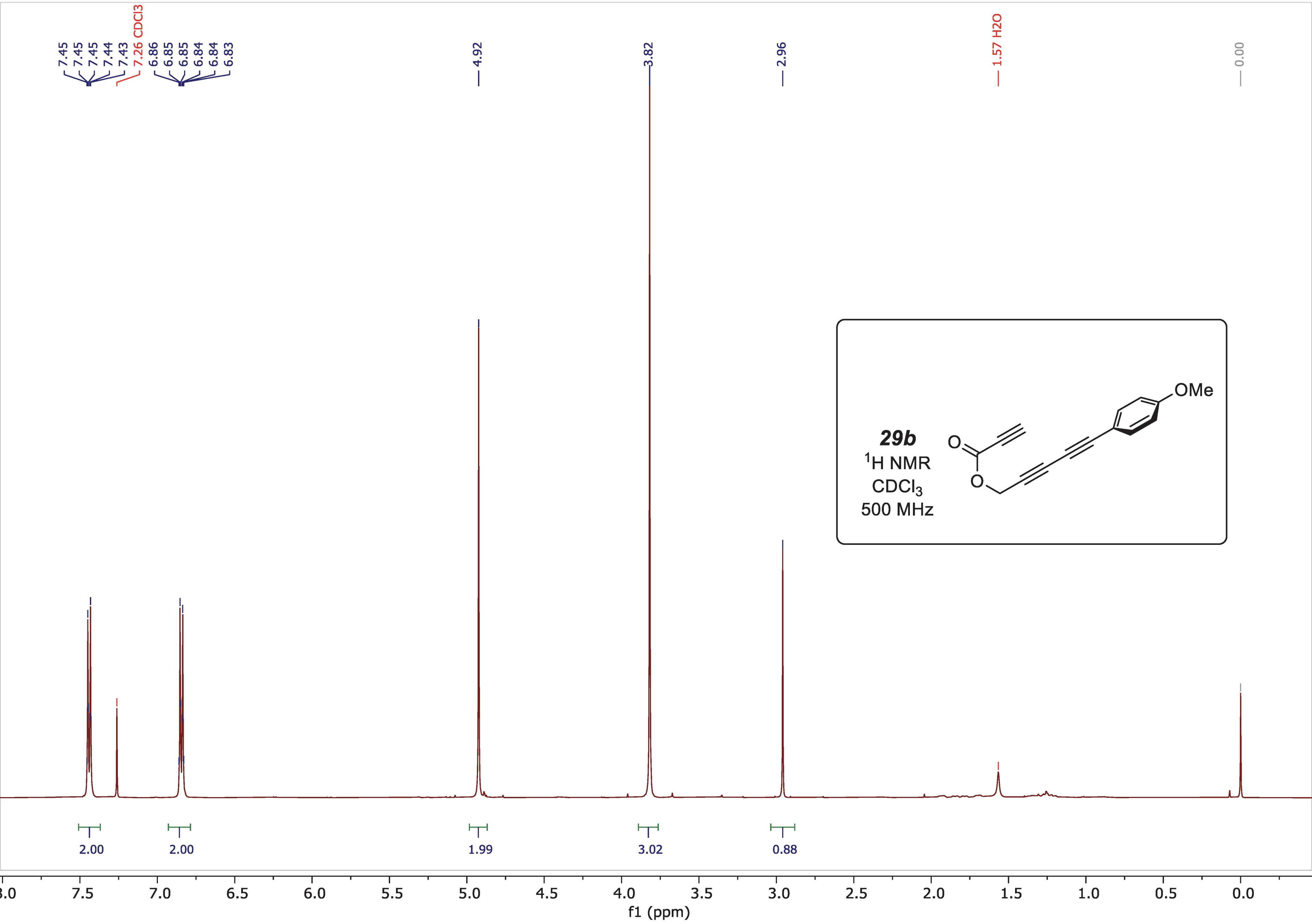
7.45
7.45
7.45
7.44
7.43
7.26 CDCl3
6.86
6.85
6.85
6.84
6.84
6.83
4.92
3.82
2.96
1.57 H2O
0.00
29b
1H NMR
CDCl3
500 MHz
OMe
O
O
2.00
2.00
1.99
3.02
0.88
8.0
7.5
7.0
6.5
6.0
5.5
5.0
4.5
4.0
3.5
3.0
2.5
2.0
1.5
1.0
0.5
0.0
f1 (ppm)

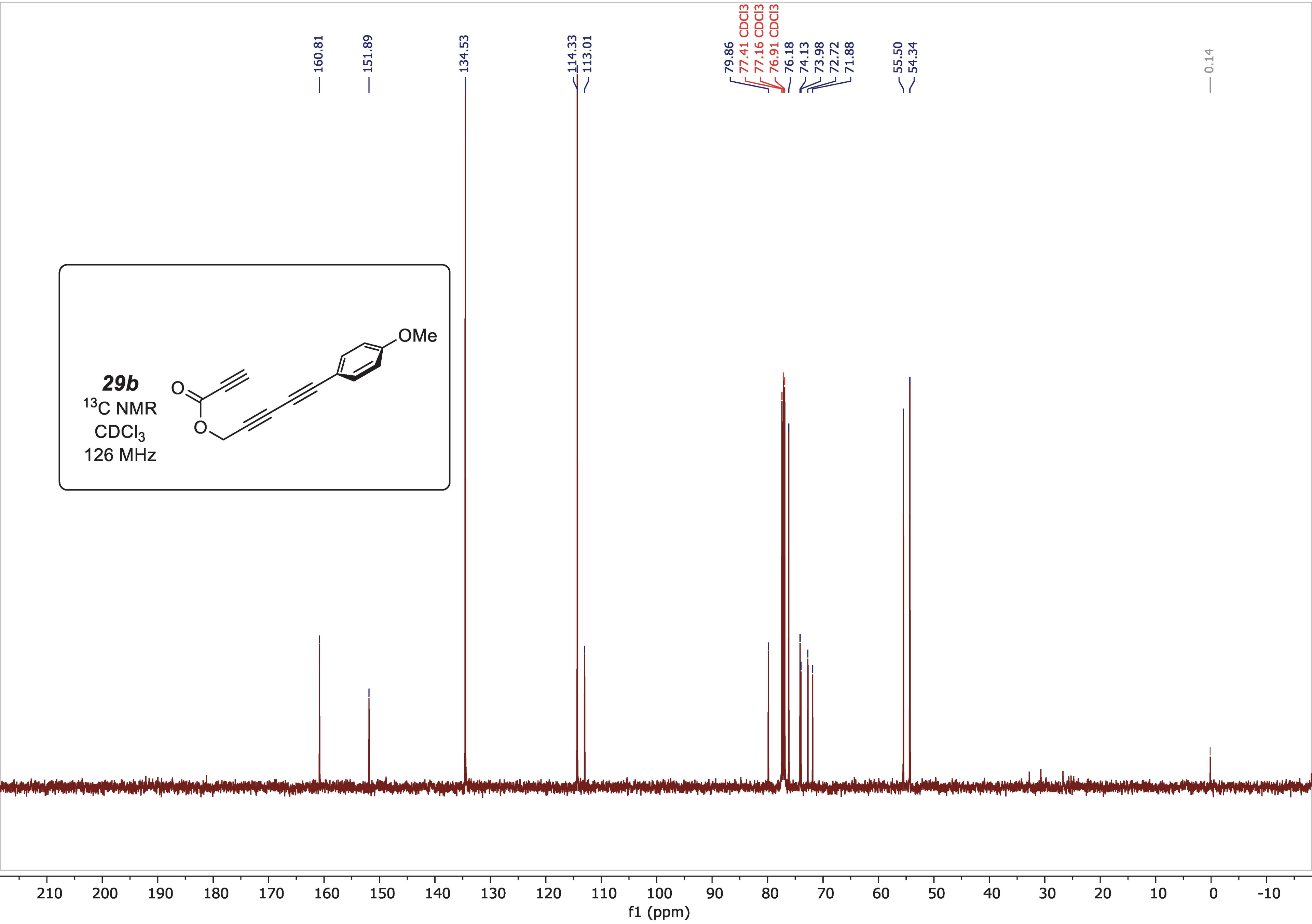
160.81
151.89
134.53
114.33
113.01
79.86
77.41 CDCl3
77.16 CDCl3
76.91 CDCl3
76.18
74.13
73.98
72.72
71.88
55.50
54.34
0.14
29b
^{13}C NMR
$CDCl_3$
126 MHz
O
O
OMe
210 200 190 180 170 160 150 140 130 120 110 100 90 80 70 60 50 40 30 20 10 0 -10
f1 (ppm)

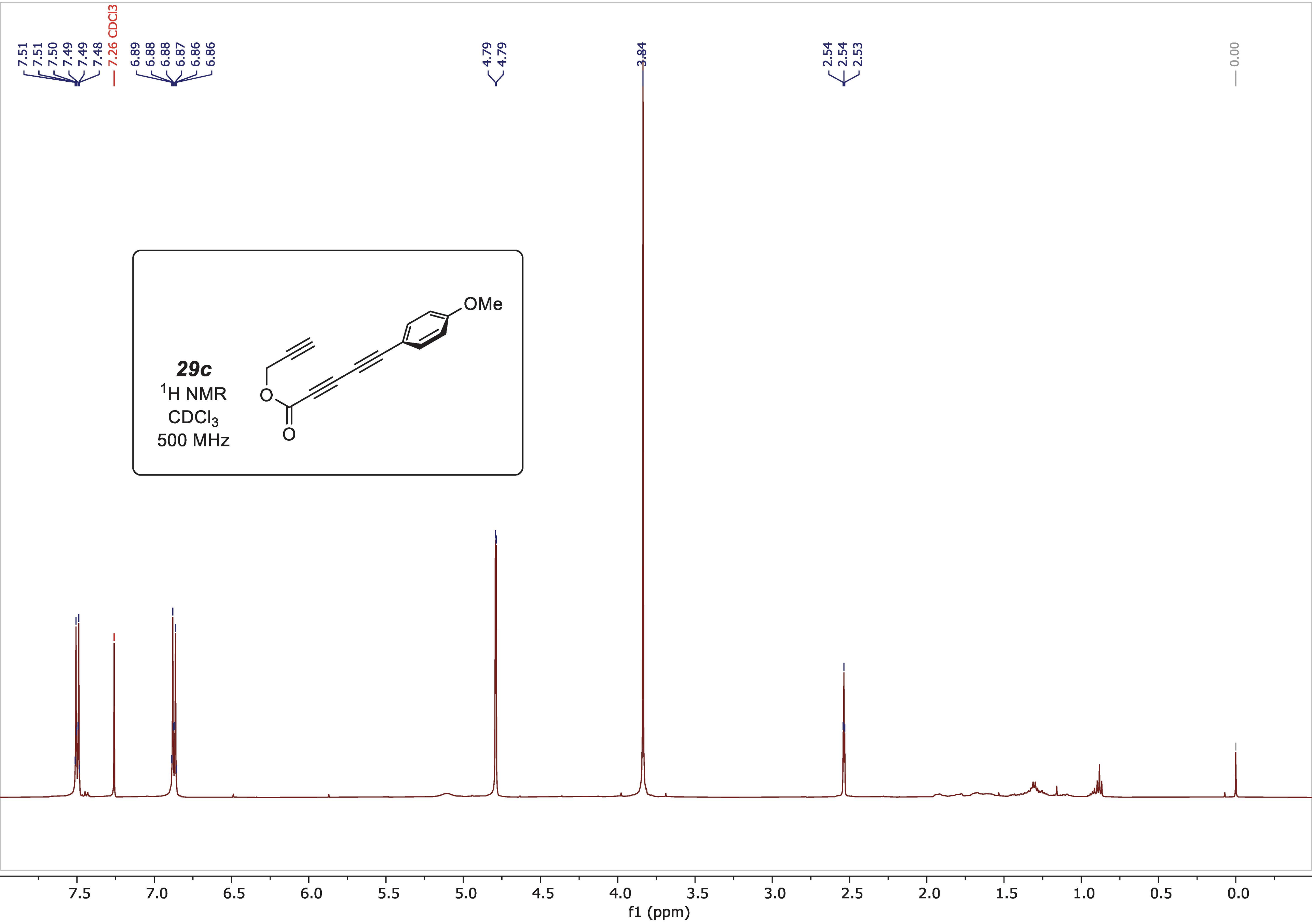
7.51
7.51
7.50
7.49
7.49
7.48
7.26 CDCl3
6.89
6.88
6.88
6.87
6.86
6.86
4.79
4.79
3.84
2.54
2.54
2.53
0.00
29c
1H NMR
CDCl3
500 MHz
OMe
O
O
7.5 7.0 6.5 6.0 5.5 5.0 4.5 4.0 3.5 3.0 2.5 2.0 1.5 1.0 0.5 0.0
f1 (ppm)

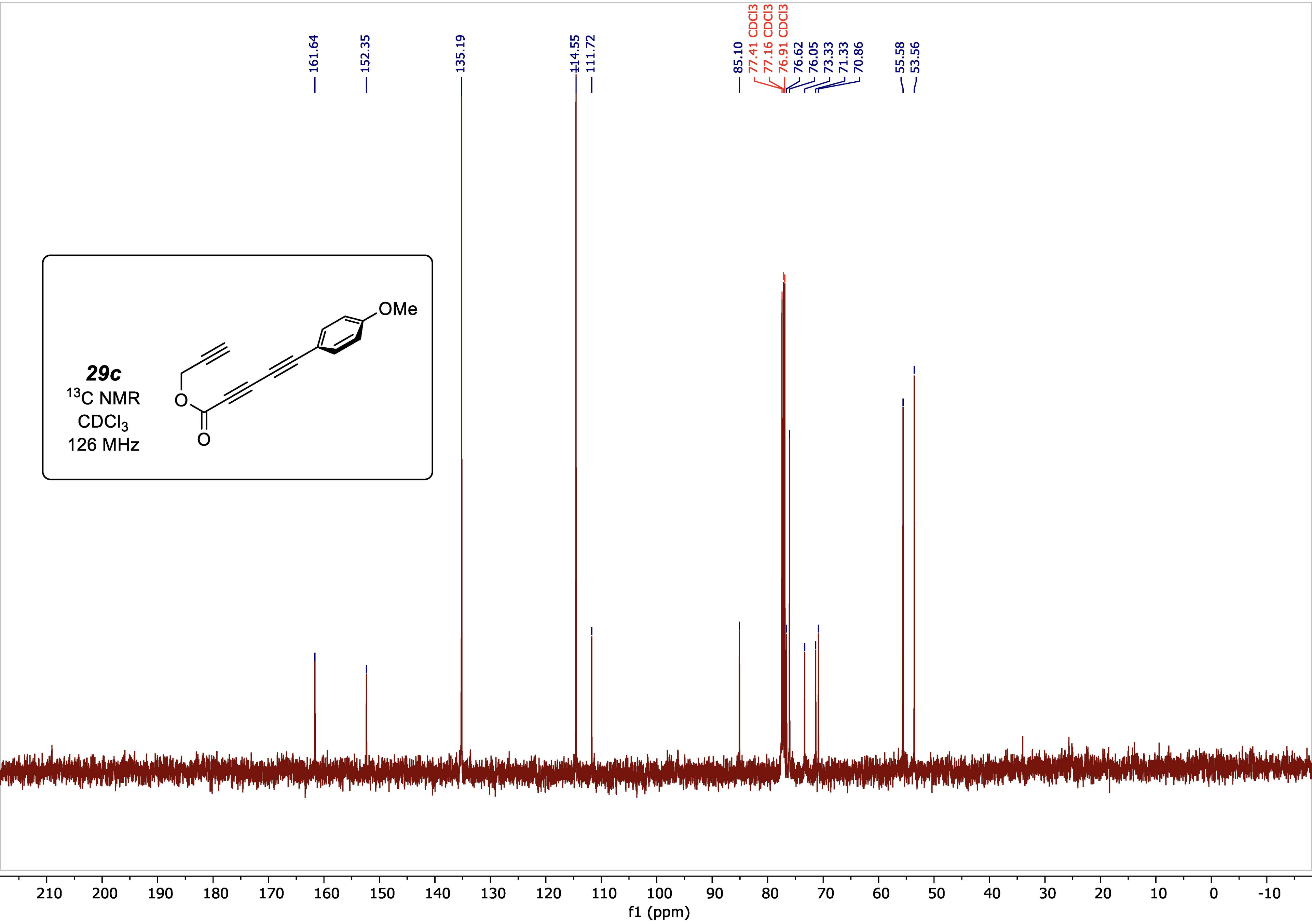
161.64
152.35
135.19
114.55
111.72
85.10
77.41 CDCl3
77.16 CDCl3
76.91 CDCl3
76.62
76.05
73.33
71.33
70.86
55.58
53.56
OMe
O
O
29c
13C NMR
CDCl3
126 MHz
210
200
190
180
170
160
150
140
130
120
110
100
90
80
70
60
50
40
30
20
10
0
-10
f1 (ppm)

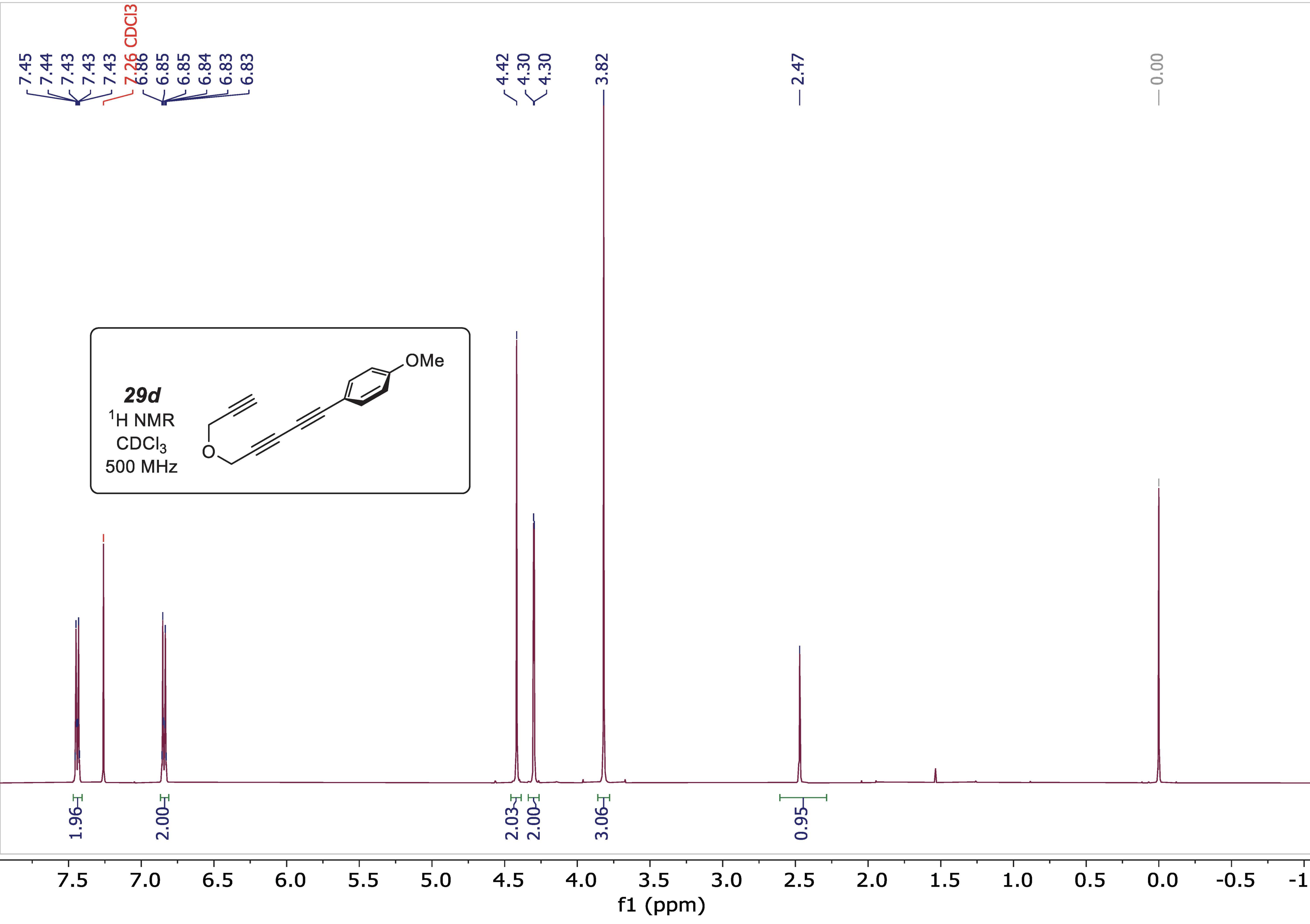
7.45
7.44
7.43
7.43
7.43
7.26 CDCl3
6.86
6.85
6.85
6.84
6.83
6.83
4.42
4.30
4.30
3.82
2.47
0.00
29d
^{1}H NMR
$CDCl_3$
500 MHz
OMe
O
1.96
2.00
2.03
2.00
3.06
0.95
7.5 7.0 6.5 6.0 5.5 5.0 4.5 4.0 3.5 3.0 2.5 2.0 1.5 1.0 0.5 0.0 -0.5 -1
f1 (ppm)

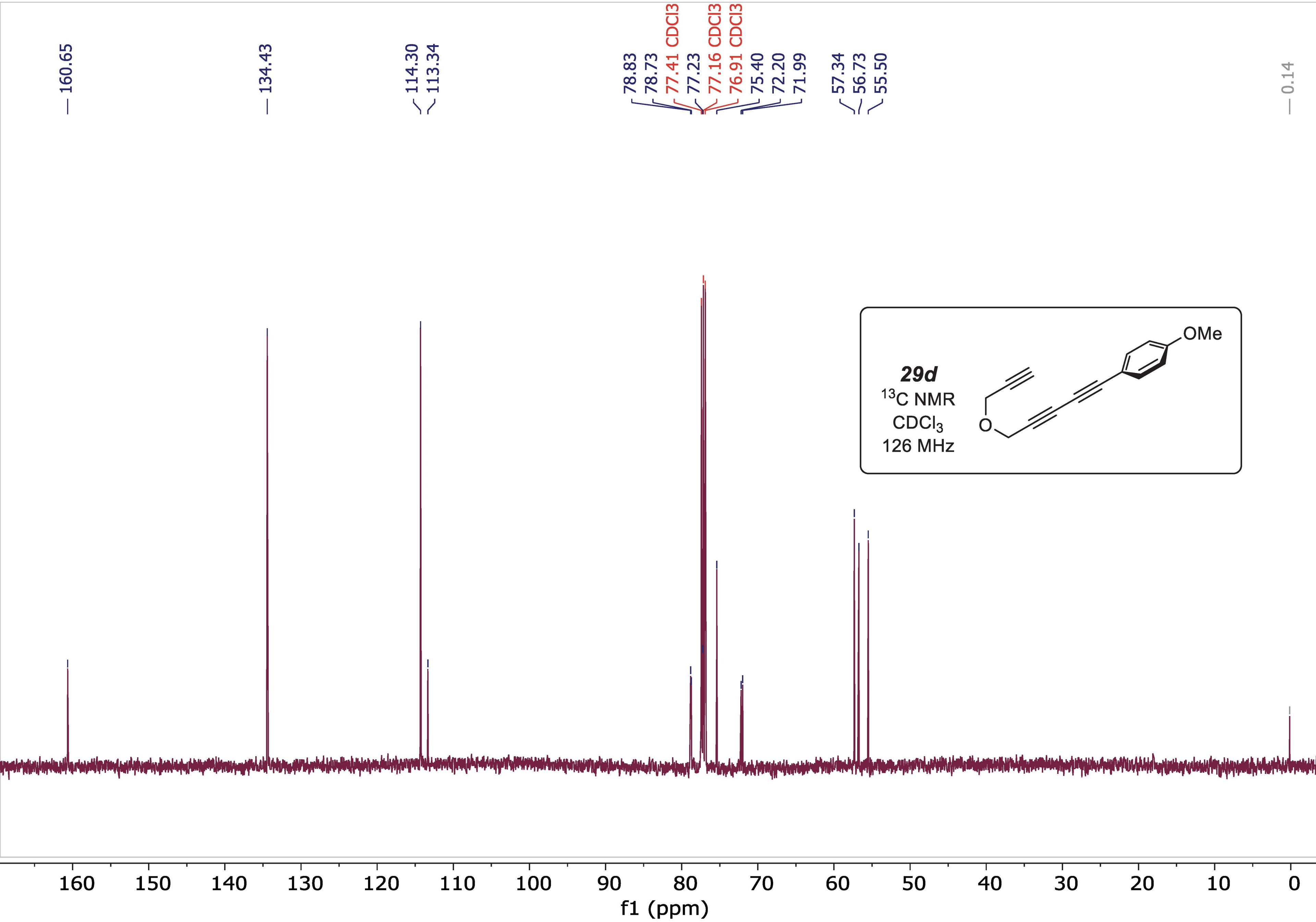
160.65
134.43
114.30
113.34
78.83
78.73
77.41 CDCl3
77.23
77.16 CDCl3
76.91 CDCl3
75.40
72.20
71.99
57.34
56.73
55.50
0.14
29d
13C NMR
CDCl3
126 MHz
OMe
O
160
150
140
130
120
110
100
90
80
70
60
50
40
30
20
10
0
f1 (ppm)

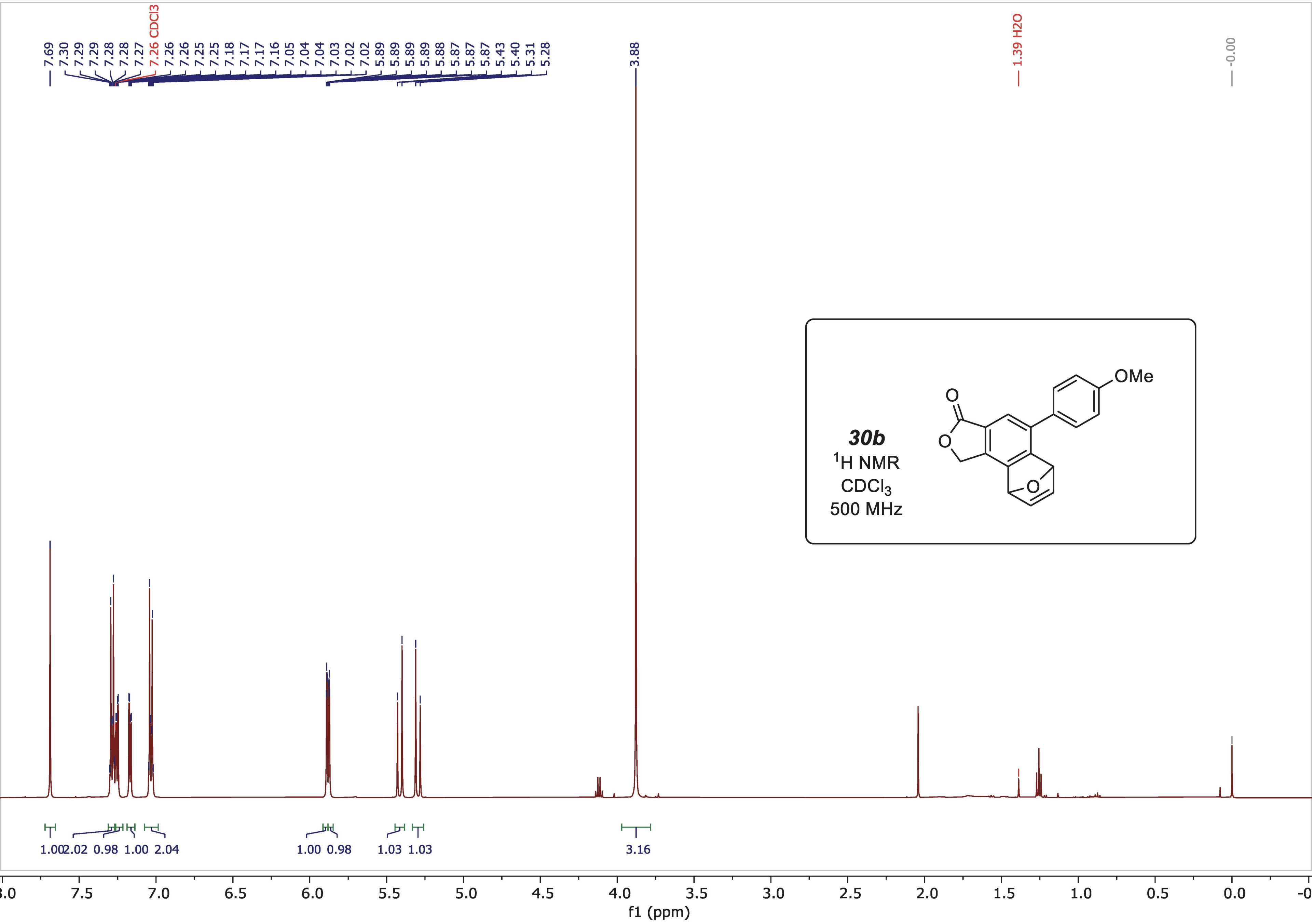
7.69
7.30
7.29
7.29
7.28
7.28
7.27
7.26 CDCl3
7.26
7.26
7.25
7.25
7.18
7.17
7.17
7.16
7.05
7.04
7.04
7.03
7.02
7.02
5.89
5.89
5.89
5.89
5.88
5.87
5.87
5.87
5.43
5.40
5.31
5.28
3.88
1.39 H2O
-0.00
30b
1H NMR
CDCl3
500 MHz
OMe
O
O
O
1.00
2.02
0.98
1.00
2.04
1.00
0.98
1.03
1.03
3.16
f1 (ppm)

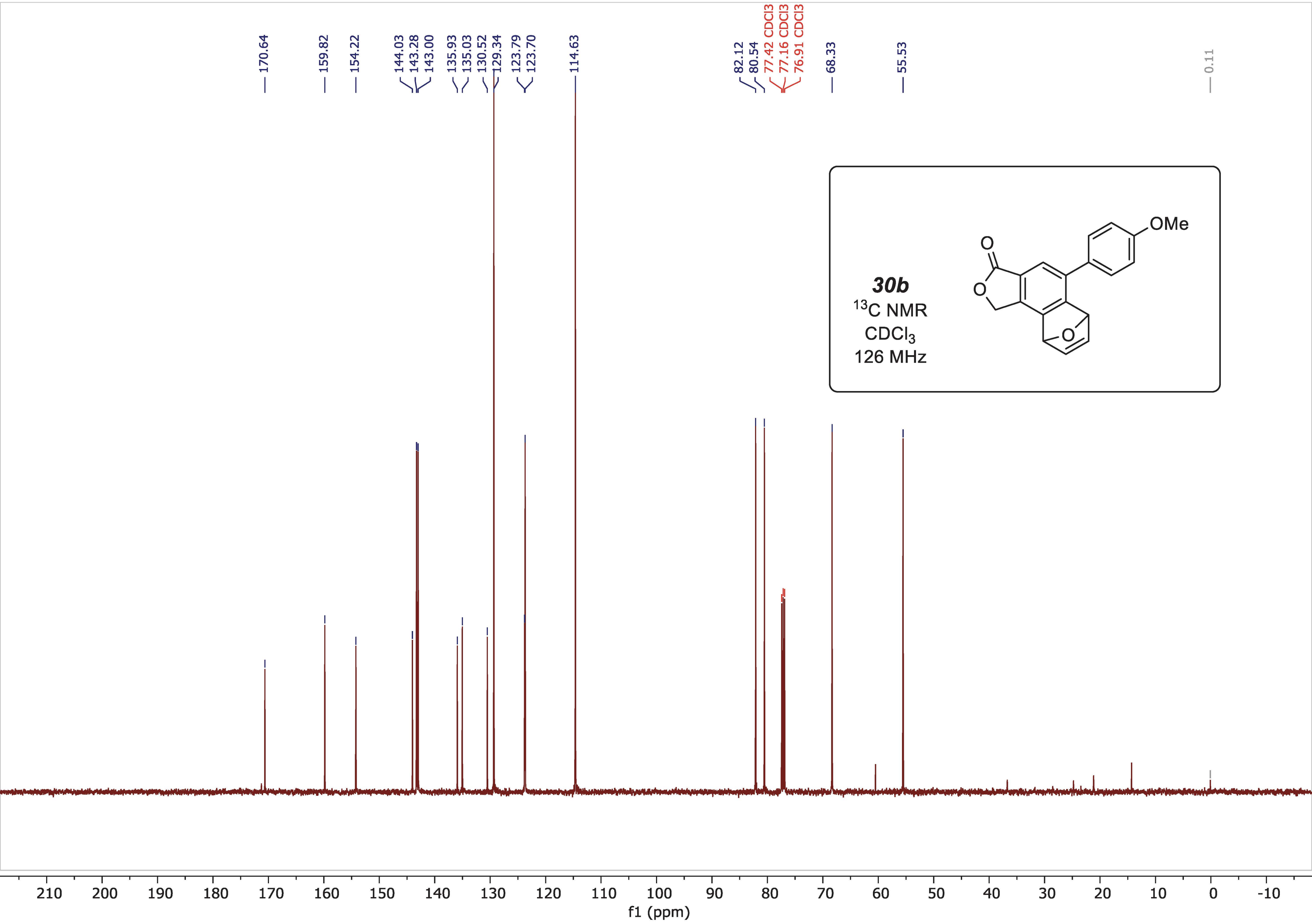
170.64
159.82
154.22
144.03
143.28
143.00
135.93
135.03
130.52
129.34
123.79
123.70
114.63
82.12
80.54
77.42 CDCl3
77.16 CDCl3
76.91 CDCl3
68.33
55.53
0.11
30b
^{13}C NMR
$CDCl_3$
126 MHz
O
O
O
OMe
210 200 190 180 170 160 150 140 130 120 110 100 90 80 70 60 50 40 30 20 10 0 -10
f1 (ppm)

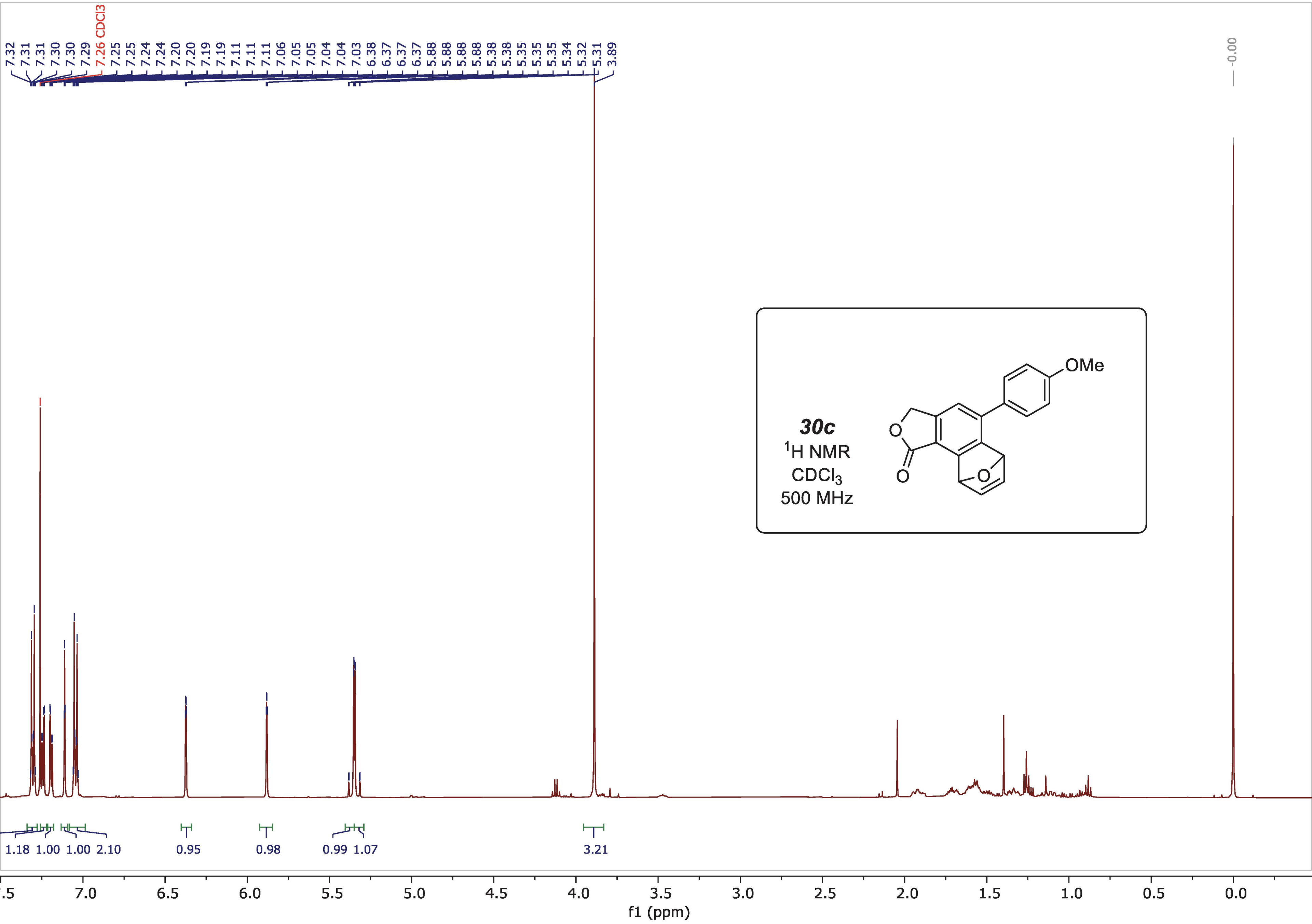
7.32
7.31
7.31
7.30
7.30
7.29
7.26 CDCl3
7.25
7.25
7.24
7.24
7.20
7.20
7.19
7.19
7.11
7.11
7.11
7.06
7.05
7.05
7.04
7.04
7.03
6.38
6.37
6.37
6.37
5.88
5.88
5.88
5.88
5.38
5.38
5.35
5.35
5.35
5.34
5.32
5.31
3.89
-0.00
30c
1H NMR
CDCl3
500 MHz
OMe
O
O
O
1.18 1.00 1.00 2.10
0.95
0.98
0.99 1.07
3.21
7.0
6.5
6.0
5.5
5.0
4.5
4.0
3.5
3.0
2.5
2.0
1.5
1.0
0.5
0.0
f1 (ppm)

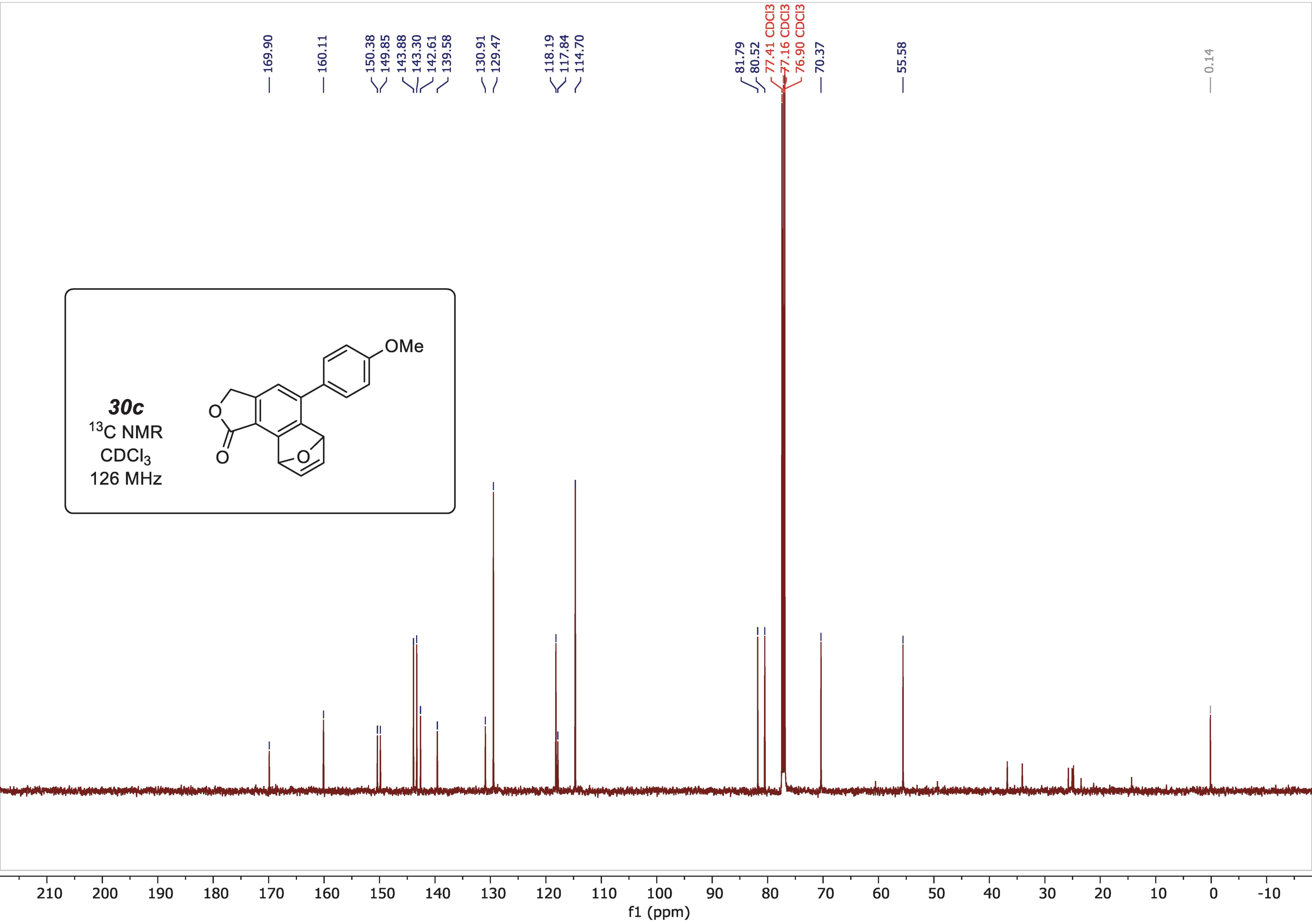
169.90
160.11
150.38
149.85
143.88
143.30
142.61
139.58
130.91
129.47
118.19
117.84
114.70
81.79
80.52
77.41 CDCl3
77.16 CDCl3
76.90 CDCl3
70.37
55.58
0.14
30c
13C NMR
CDCl3
126 MHz
OMe
O
O
O
210 200 190 180 170 160 150 140 130 120 110 100 90 80 70 60 50 40 30 20 10 0 -10
f1 (ppm)

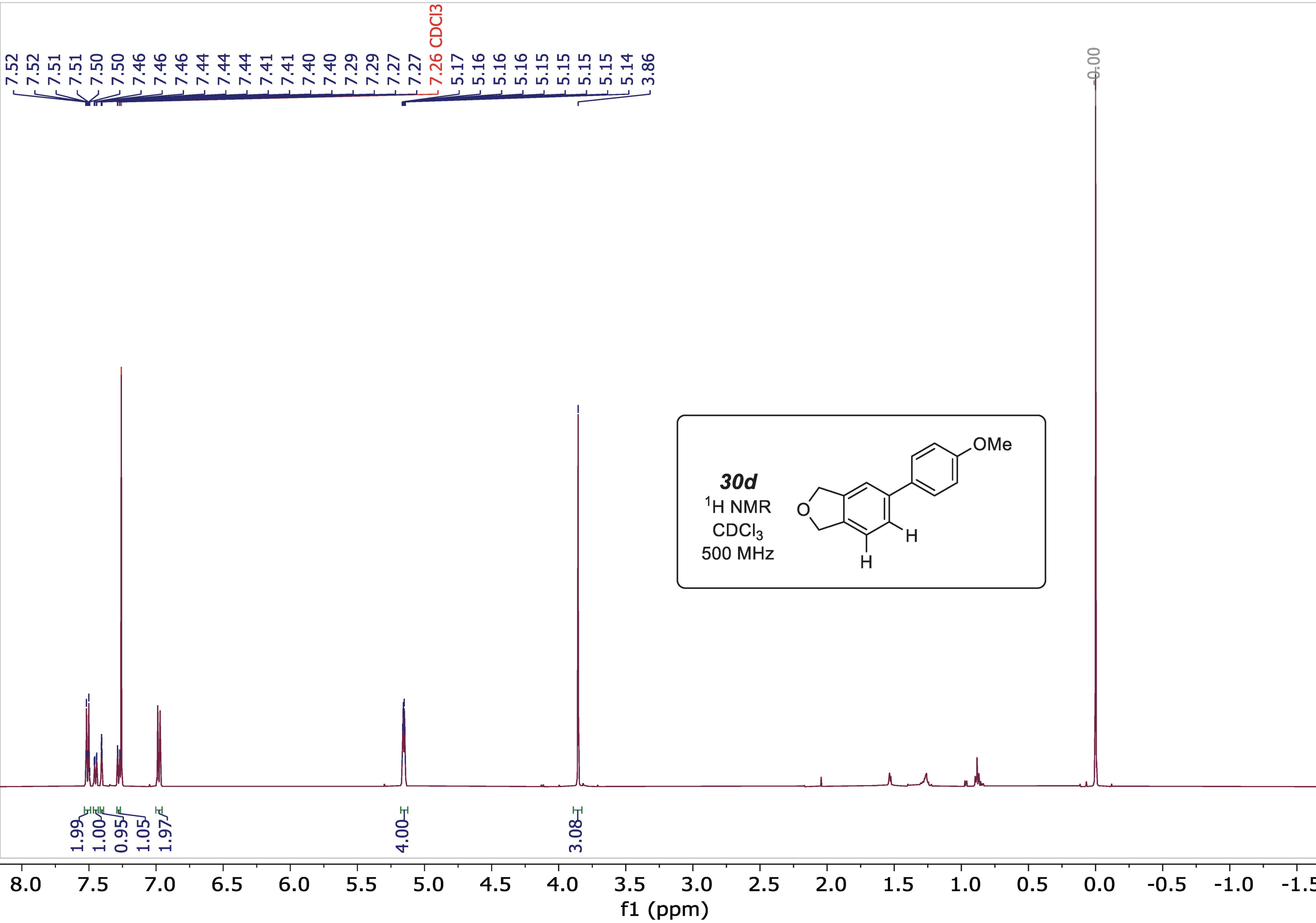
7.52
7.52
7.51
7.51
7.50
7.50
7.46
7.46
7.46
7.44
7.44
7.44
7.41
7.41
7.40
7.40
7.29
7.29
7.27
7.27
7.26 CDCl3
5.17
5.16
5.16
5.16
5.15
5.15
5.15
5.15
5.14
3.86
-0.00
30d
1H NMR
CDCl3
500 MHz
OMe
O
H
H
1.99
1.00
0.95
1.05
1.97
4.00
3.08
8.0
7.5
7.0
6.5
6.0
5.5
5.0
4.5
4.0
3.5
3.0
2.5
2.0
1.5
1.0
0.5
0.0
-0.5
-1.0
f1 (ppm)

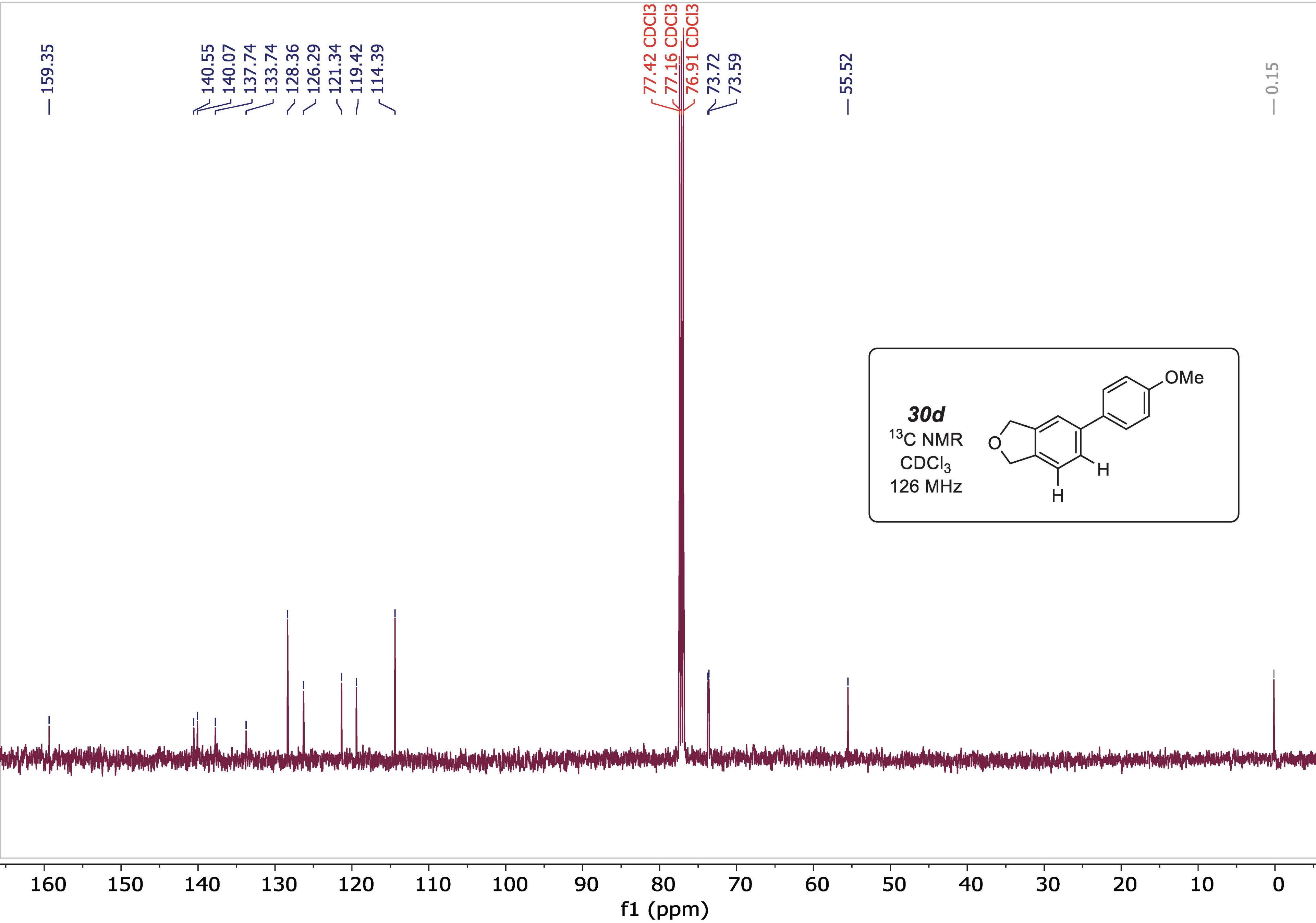
159.35
140.55
140.07
137.74
133.74
128.36
126.29
121.34
119.42
114.39
77.42 CDCl3
77.16 CDCl3
76.91 CDCl3
73.72
73.59
55.52
0.15
30d
13C NMR
CDCl3
126 MHz
OMe
O
H
H
160 150 140 130 120 110 100 90 80 70 60 50 40 30 20 10 0
f1 (ppm)

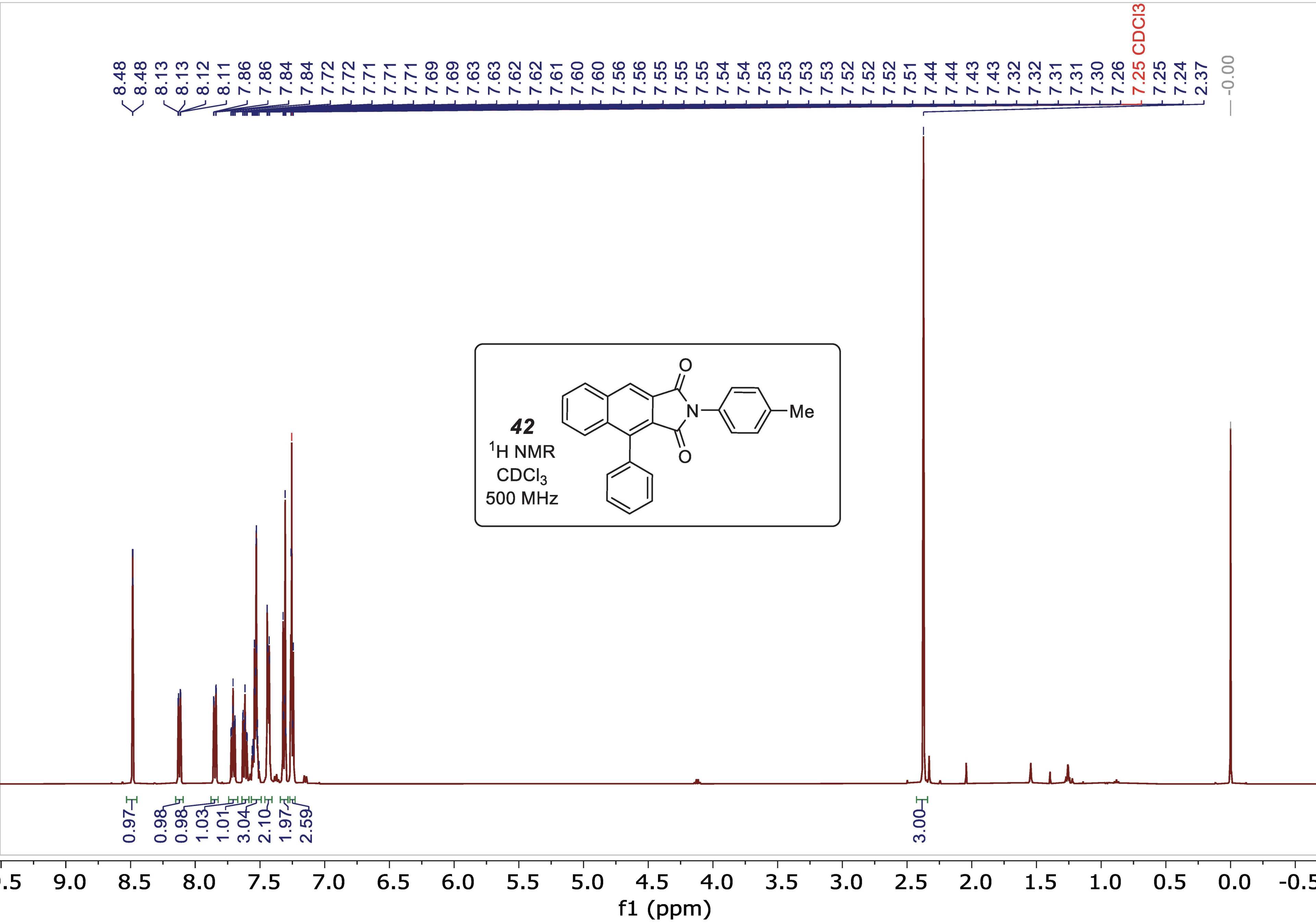
8.48 8.48 8.13 8.13 8.12 8.11 7.86 7.86 7.84 7.84 7.72 7.72 7.71 7.71 7.71 7.69 7.69 7.63 7.63 7.62 7.62 7.61 7.60 7.60 7.56 7.56 7.55 7.55 7.55 7.54 7.54 7.53 7.53 7.53 7.53 7.52 7.52 7.52 7.51 7.44 7.44 7.43 7.43 7.32 7.32 7.31 7.31 7.30 7.26 7.25 CDCl3 7.25 7.24 2.37
-0.00
O
N
Me
O
42
^{1}H NMR
$CDCl_3$
500 MHz
0.97 0.98 0.98 1.03 1.01 3.04 2.10 1.97 2.59
3.00
9.0 8.5 8.0 7.5 7.0 6.5 6.0 5.5 5.0 4.5 4.0 3.5 3.0 2.5 2.0 1.5 1.0 0.5 0.0
f1 (ppm)

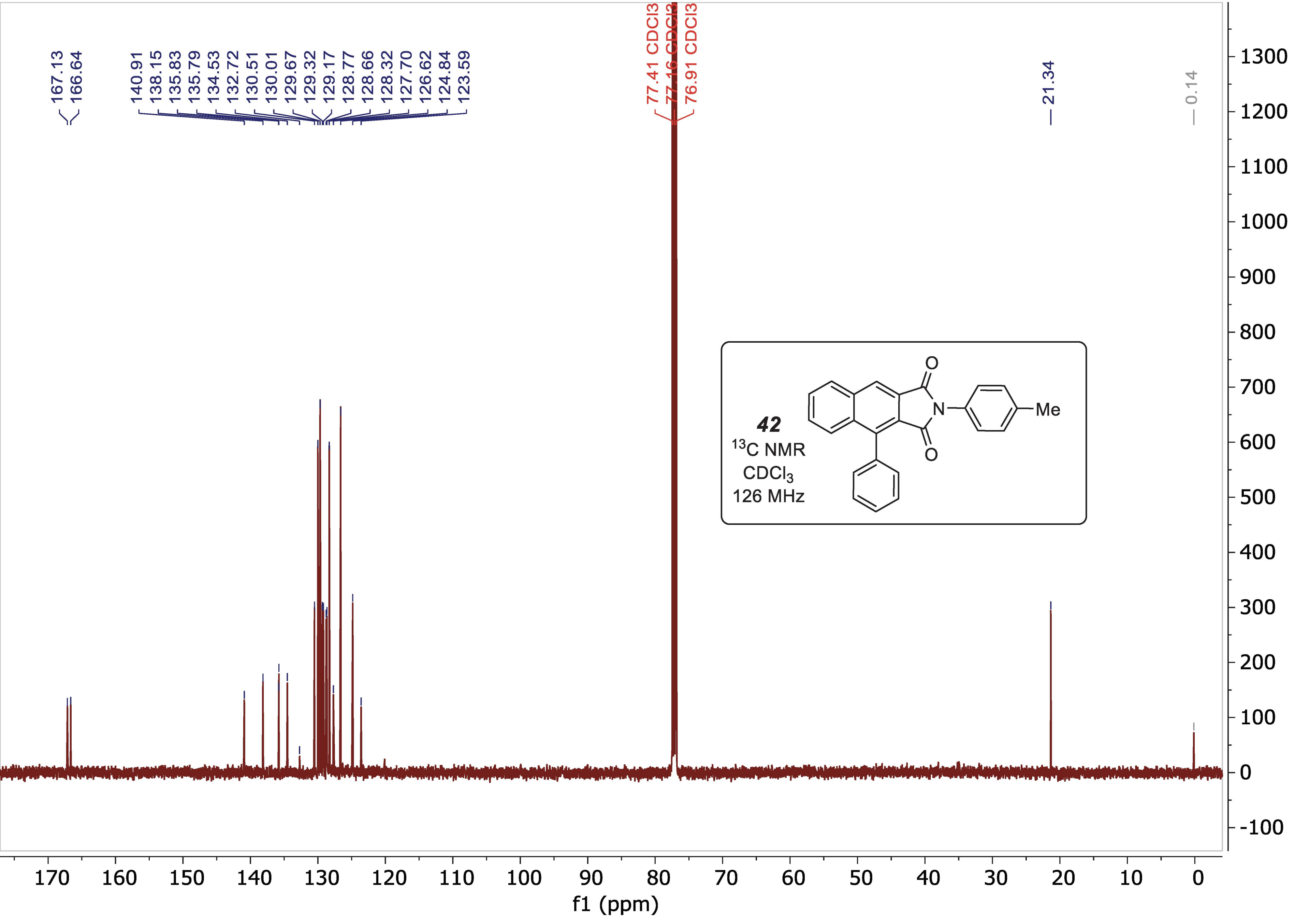
167.13
166.64
140.91
138.15
135.83
135.79
134.53
132.72
130.51
130.01
129.67
129.32
129.17
128.77
128.66
128.32
127.70
126.62
124.84
123.59
77.41 CDCl3
77.16 CDCl3
76.91 CDCl3
21.34
0.14
42
^{13}C NMR
$CDCl_3$
126 MHz
O
N
Me
O
f1 (ppm)

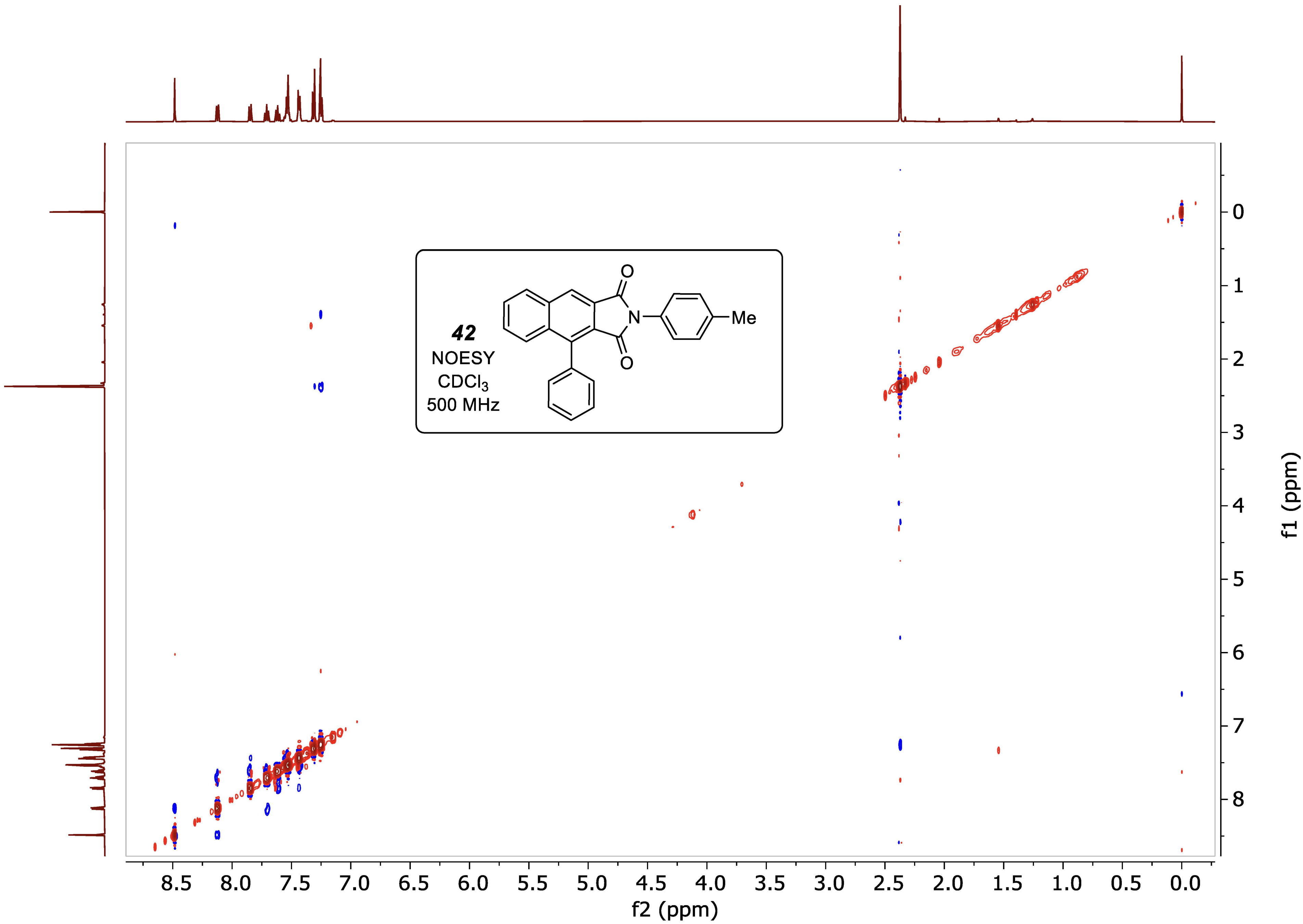
42
NOESY
$CDCl_3$
500 MHz
O
N
Me
O
8.5 8.0 7.5 7.0 6.5 6.0 5.5 5.0 4.5 4.0 3.5 3.0 2.5 2.0 1.5 1.0 0.5 0.0
f2 (ppm)
0 1 2 3 4 5 6 7 8
f1 (ppm)

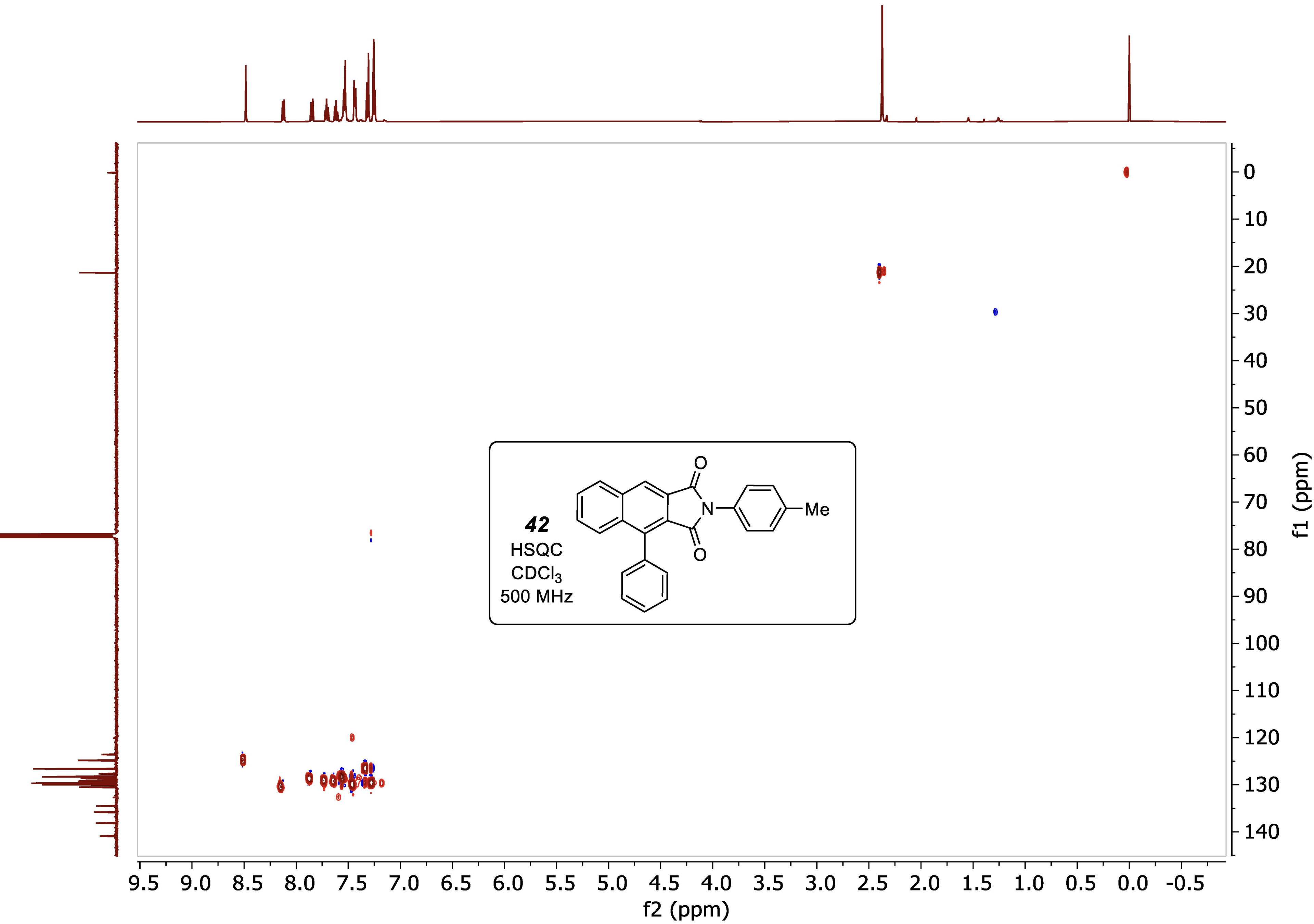
42
HSQC
$CDCl_3$
500 MHz
O
N
Me
O
f2 (ppm)
f1 (ppm)

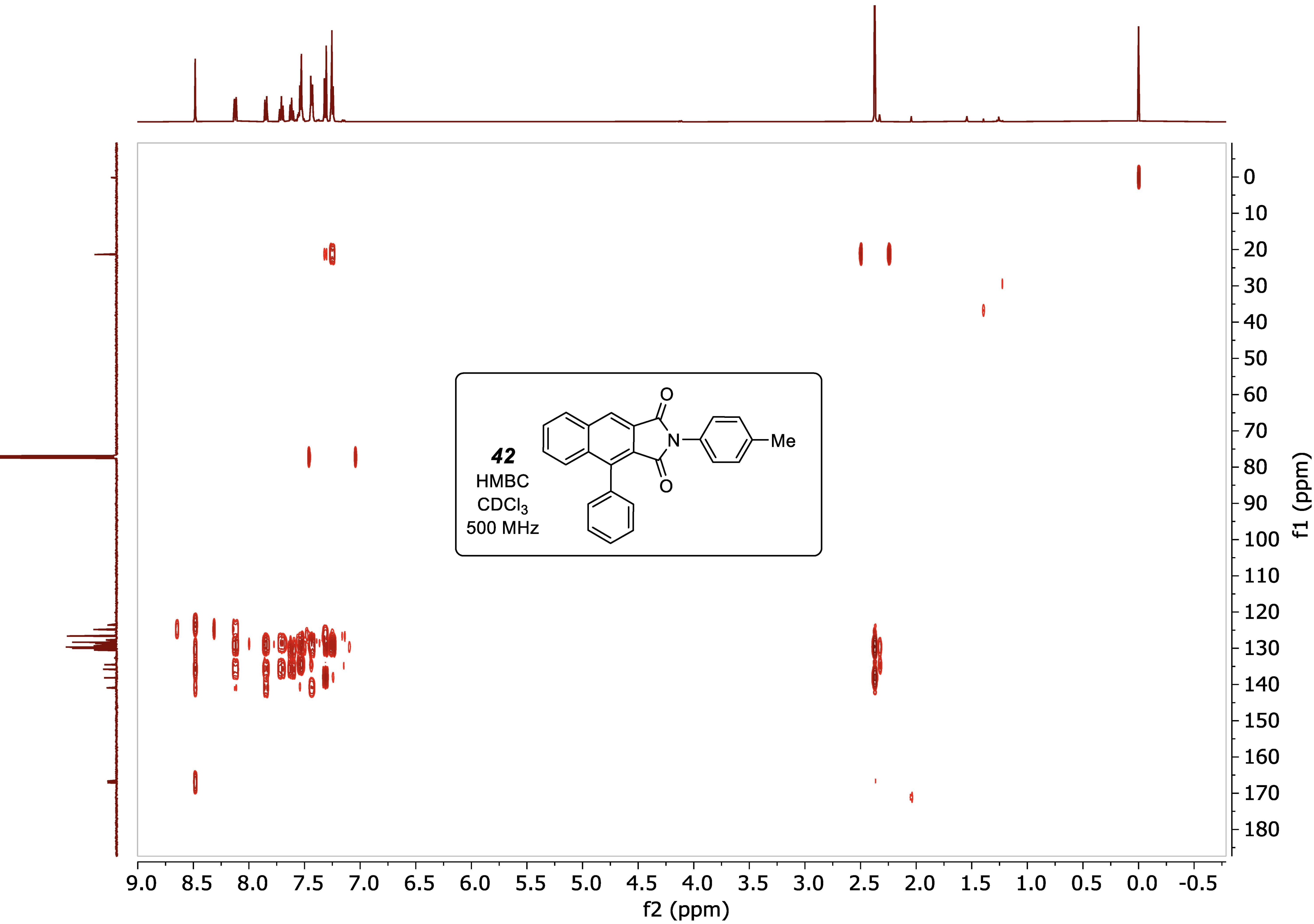
42
HMBC
$CDCl_3$
500 MHz
O
N
Me
O
f1 (ppm)
f2 (ppm)
9.0 8.5 8.0 7.5 7.0 6.5 6.0 5.5 5.0 4.5 4.0 3.5 3.0 2.5 2.0 1.5 1.0 0.5 0.0 -0.5
0 10 20 30 40 50 60 70 80 90 100 110 120 130 140 150 160 170 180
